# Range-Wide Latitudinal and Elevational Temperature Gradients for the World's Terrestrial Birds: Implications under Global Climate Change

**DOI:** 10.1371/journal.pone.0098361

**Published:** 2014-05-22

**Authors:** Frank A. La Sorte, Stuart H. M. Butchart, Walter Jetz, Katrin Böhning-Gaese

**Affiliations:** 1 Cornell Laboratory of Ornithology, Cornell University, Ithaca, New York, United States of America; 2 BirdLife International, Cambridge, United Kingdom; 3 Department of Ecology and Evolutionary Biology, Yale University, New Haven, Connecticut, United States of America; 4 Biodiversity and Climate Research Centre (BiK-F), Senckenberg Gesellschaft für Naturforschung, Frankfurt (Main), Germany; 5 Department of Biological Sciences, Goethe Universität, Frankfurt (Main), Germany; University of Kent, United Kingdom

## Abstract

Species' geographical distributions are tracking latitudinal and elevational surface temperature gradients under global climate change. To evaluate the opportunities to track these gradients across space, we provide a first baseline assessment of the steepness of these gradients for the world's terrestrial birds. Within the breeding ranges of 9,014 bird species, we characterized the spatial gradients in temperature along latitude and elevation for all and a subset of bird species, respectively. We summarized these temperature gradients globally for threatened and non-threatened species and determined how their steepness varied based on species' geography (range size, shape, and orientation) and projected changes in temperature under climate change. Elevational temperature gradients were steepest for species in Africa, western North and South America, and central Asia and shallowest in Australasia, insular IndoMalaya, and the Neotropical lowlands. Latitudinal temperature gradients were steepest for extratropical species, especially in the Northern Hemisphere. Threatened species had shallower elevational gradients whereas latitudinal gradients differed little between threatened and non-threatened species. The strength of elevational gradients was positively correlated with projected changes in temperature. For latitudinal gradients, this relationship only held for extratropical species. The strength of latitudinal gradients was better predicted by species' geography, but primarily for extratropical species. Our findings suggest threatened species are associated with shallower elevational temperature gradients, whereas steep latitudinal gradients are most prevalent outside the tropics where fewer bird species occur year-round. Future modeling and mitigation efforts would benefit from the development of finer grain distributional data to ascertain how these gradients are structured within species' ranges, how and why these gradients vary among species, and the capacity of species to utilize these gradients under climate change.

## Introduction

A variety of responses are available for species as global climate change progresses [1], [2] and changing climatic conditions increasingly impact species' performance and fitness. The two primary options, which are not necessarily independent, are for species' populations to shift their geographic ranges to regions containing suitable climatic conditions, or to remain and adapt phenotypically or genetically [3], [4]. One of the most important immediate responses is for species to track their geographic climatic associations across space. This may be possible provided that opportunities are available for geographic range-shifts, species have the physiological and behavioral capacity to take advantage of them, and they can keep pace with the velocity of climate change [5]. Geographic niche tracking has been observed under climate change over geological time scales [6] and more recently under modern climate change [7], [8].

Two environmental gradients appear particularly relevant when considering geographic niche tracking under past and current climate change: surface latitudinal and elevational temperature gradients. Both gradients represent natural environmental features whose location and general form remain consistent across ecological time-scales, and there is empirical evidence that many taxa including birds are currently tracking these gradients under climate change [7], [9]. Latitudinal temperature gradients are strongest outside the tropics, especially in the Northern Hemisphere where the most extensive land masses are located [10]. Elevational temperature gradients are stronger and less variable than latitudinal temperature gradients but are geographically more restricted [11]. Latitudinal and elevational temperature gradients are not equally available for all species nor are they uniformly distributed within species' geographic ranges. Under current climate change projections, there is no evidence to suggest the strength of elevational temperature gradients are expected to change; whereas there is evidence that the strength of latitudinal temperatures gradients may change over broad geographic regions [2]. Due to latitudinal asymmetry in surface warming, latitudinal temperature gradients are projected to weaken in the Northern Hemisphere [2], [12], which is expected to result in diminished seasonality and increased vegetation growth at the high northern latitudes [13], [14].

Life history, morphological, and physiological traits are known to vary among species' populations at geographic scales [15] and, in some cases, this variation is correlated with latitudinal or elevational gradients [16], [17] and associated temperature gradients [18]. This variation in ecological traits across latitudinal or elevational gradients can in turn affect reproductive potential and rates of population growth, especially at range limits [19], [20]. Under climate change, the structure of latitudinal and elevational temperature gradients can be modified, affecting population growth rates, especially at the leading and trailing edge of the gradient within species' ranges [21], [22]. Specifically, climate change can result in population growth increasing at the leading edge and declining at the trailing edge of the gradient, with the increased likelihood of range shifts at the leading edge and extirpation of populations at the trailing edge.

**Figure 1:**
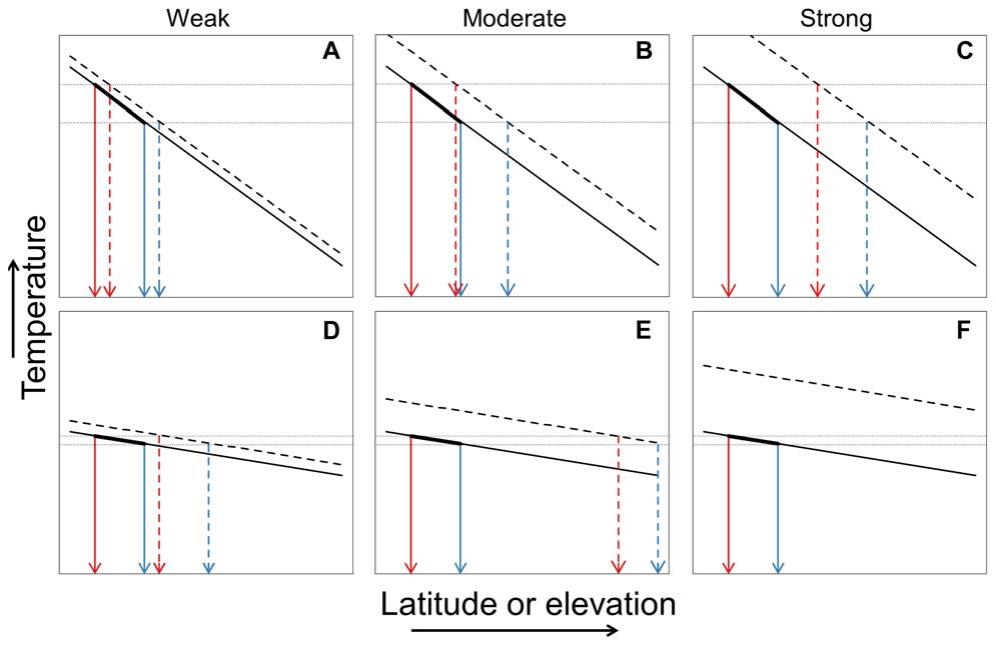
Conceptual figures of six geographic range-shift scenarios for species containing steep (A,B,C) or shallow (D,E,F) latitudinal or elevational temperature gradients within their geographic ranges. The spatial breadth of the latitudinal or elevational gradient within the geographic range is identical in each case. The current temperature gradient (solid line) and the projected temperature gradient under climate change (dashed line) are shown for each scenario. The location of the geographic range of the species along the temperature gradient is depicted with a bold line between the solid blue and red arrows. The species' leading (blue arrow) and trailing (red arrow) range limits are shown based on the current gradient (solid arrows) and the projected gradient under climate change (dashed arrows). Three gradations in projected climate change are shown: weak (A, D), moderate (B, E), and strong climate change (C, F). The horizontal dotted lines identify the boundaries of each species' geographic climatic niche as defined along the temperature gradient. Everything else being equal, species with steeper environmental temperature gradients have broader geographic climatic niches and need to shift their geographic range less in order to track climate change.

The strength of latitudinal and elevational temperature gradients within species current geographic ranges relative to projected changes in temperature under climate change can determine the potential for range-shifts to occur under climate change. For species whose geographic ranges have similar spatial dimensions in relationship to latitudinal or elevational temperature gradients, the steeper the gradient, the broader the species' geographic climatic niche (i.e., species' association with climatic conditions across space) and the greater the likelihood that a larger component of current climatic associations will be retained within the geographic range as temperatures increase (Figure 1A,B,C). In contrast, the shallower the gradient, the narrower the species' geographic climatic niche and the smaller the likelihood that a significant geographic component of current climatic conditions will be retained within the geographic range as temperatures increase (Figure 1D,E,F). One potential consequence of shallow latitudinal or elevational temperature gradients is the formation of range-shift gaps [10]. Here, increasing temperatures can result in the complete loss of current climatic associations within species' current geographic ranges, an outcome that may substantially hinder successful range-shifts. Range-shift gap width is also likely to be negatively correlated with the strength of the temperature gradients (Figure 1), suggesting species' distributions containing very weak gradients, as found with many tropical species, are particularly susceptible to the consequences of climate change [10]. In total, climate change can modify latitudinal or elevational temperature gradients within species' geographic ranges, which in turn can alter population dynamics at range boundaries and range-shift potential based on the strength of those gradients relative to the magnitude of climate change.

Here, we estimate latitudinal and elevational temperature gradients within the geographic ranges of *ca*. 90% of the world's avifauna, numbering 9,014 species. To evaluate whether bird species that are currently threatened with extinction also face particularly high risks under climate change, we structure our analysis to compare species classified as threatened (*n* = 878) vs. non-threatened with extinction (*n* = 8,136) on the IUCN Red List [23]. We additionally examine the ability of species' geography (range size, shape, and orientation relative to the equator) to explain variation in elevational and latitudinal temperature gradients among species. These characteristics of species' ranges tend to be geographically similar across taxa [24] and are determined in part by spatial variation in climate and topography [25]. Lastly, to assess the degree of correspondence between projected warming and the availability of elevational and latitudinal temperature gradients within species' ranges, we consider how gradient strength is related to variation in projected changes in temperature under climate change.

## Materials and Methods

### Data sources and preparation

We acquired range maps for the world's birds from BirdLife International and NatureServe [26] whose data sources are described in Buchanan, Donald and Butchart [27]. We considered breeding/resident ranges only and excluded species that were not well suited for our analysis, specifically those associated with marine environments. This resulted in a total of 9,014 extant species for analysis (see Table S1). These species were classified as either threatened (*n* = 878) or non-threatened with extinction (*n* = 8,136) using the IUCN Red List [23]. Here threatened refers to species identified as vulnerable, endangered, or critically endangered under the IUCN Red List.

Species range data was analyzed using a gridded system having a cylindrical equal-area projection and a cell area of 3,091 km^2^. We defined species' geographic breeding ranges as the spatial combination of equal area cells with ≥50% terrestrial surface area (based on the proportion of the cell that contained non-marine terrain) that intersected each species' geographic range polygon. We placed each species into one of six biogeographical realms [28] based on the realm that contained the greatest proportion of each species' gridded range (Nearctic, Palaearctic, IndoMalaya, Neotropics, Afrotropics, and Australasia). Species whose ranges occurred primarily in Antarctica or Oceania were excluded from analysis. From the 9,014 species, we acquired minimum and maximum elevation associations for 4,978 species from BirdLife International [23]. The distribution of these species across the six realms did not show geographic biases, with the proportion of species in each realm not diverging significantly from expectation (*χ* ^2^ = 0.029, df = 5, *P* = 1.0).

Temperatures within species' geographic ranges were estimated using the annual average of 1950–2000 average-monthly temperatures from WorldClim [29] gridded and analyzed at a 30-arcsec resolution (*ca*. 1 km at the equator). Elevations within species' ranges were estimated using the USGS global digital elevation model (GTOPO30) gridded and analyzed at a 30-arcsec resolution. Tropospheric lapse rates, the association between temperature and elevation within the troposphere, were estimated globally using a gridded model at a resolution of 2.5° (*ca*. 278 km at the equator) for the period 1948–2001 [11].

Broad-scale, expert-based range maps are only coarsely defined estimates of species occurrence and reliably characterize presences only above 100 km grain [30], [31]. Here, we are interested in the form of latitudinal and elevational temperature gradients (i.e., shallow or steep) as defined across the geographic breadth of species' distributions independent of patterns of occurrence. The use of relatively high resolution global estimates of temperature and elevation provided the detail of data necessary to estimate the full structure of these gradients for individual species, especially in situations where geographic ranges were small or temperature gradients were non-linear. For the purpose of this baseline study we assume that low probability of occurrence naturally incurred at the finer spatial grain are not biasing the geographic and species comparisons. We acknowledge that this assumption is not necessarily valid and will require careful follow-up assessments at local scales or at broader scales once more detailed distribution data becomes available.

Projected changes in temperature (temperature anomalies) were estimated using projections averaged over four Atmosphere Ocean General Circulation Models (AOGCMs). The four AOGCMs were CNRMCM3, CSIRO-Mk3.0, ECHam5, and MIROC 3.2. The gridded projections from these models were downscaled to 30 arc-minute resolution using pattern scaling with the MarkSim weather generator [32]. The projections were based on the A2 scenario, which is currently considered to be the most relevant under current greenhouse gas emission rates [33], [34]. The anomalies represent the change in temperature between the twentieth century control 30-year normal (1961–1990) and the 30-year period 2081–2100. The temperature anomalies were bilinearly interpolated to match the resolution of the 3,091 km^2^ equal-area grid.

### Elevational temperature gradients

We estimated latitudinal and elevational temperature gradients separately in order to better represent each gradient's unique characteristics. The presence of elevational temperature gradients is dictated by the presence of mountain systems or orographic features, which show substantial geographic variation (see Figure S1). Mountain systems often contain strong topographic heterogeneity resulting in a mosaic of climatic conditions, which are poorly estimated using interpolated weather station data [35], [36]. Here, we use elevation data, which does not rely on similar interpolation methods, to estimate elevational temperature gradients within species' ranges.

Unlike latitudinal temperature gradients, the strength of elevational temperature gradients are broadly consistent from the equator to the poles. Tropospheric lapse-rate averages 6.2°C km^−1^ over the continents, with a range of 4.5 to 6.5°C km^−1^ from the polar latitudes to the equator, respectively [11]. To account for this geographic variation, we calculated the average tropospheric lapse-rate within each species' geographic range. To account for the high variability in the extent and spatial distribution of orographic features and the decline in terrestrial area with increasing elevation resulting in strongly skewed distributions of elevation within species' ranges, we estimated two components of the elevational temperature gradient for each species: (1) the overall magnitude and (2) the evenness of the gradient. We then combined tropospheric lapse-rate with magnitude and evenness to generate one metric designed to represent the overall strength of elevational temperature gradients within each species' range.

More specifically, for species whose minimum and maximum elevation associations had both been estimated (*n* = 4,978), we excluded elevations within the geographic range below and above these associations. We then identified the actual minimum and maximum elevations within each species' range based on our gridded elevation data, which in combination with species' documented elevation associations were used to calculate each species' realized vertical range extent. Vertical range extent was used to estimate the magnitude of each species' elevational temperature gradient. To assess how evenly the vertical range extent was distributed across each species' range, we first summed the number of 30 arc-second elevation cells in all 100-m elevational bands found within each species range. We then used the Lorenz curve and associated Gini coefficient [37]–[39], explained below, to estimate how evenly the cells were distributed across elevation bands. Here, the 100-m bands were first ranked from lowest to highest elevation. The frequency of cells within these bands was then used to plot the Lorenz curve, i.e. the cumulative proportion of the number of cells in each band (x-axis) against the corresponding cumulative proportion of their elevations (y-axis). Gini coefficients (or Gini ratio) were then calculated, which is defined as twice the area contained between the Lorenz curve and the line of perfect equality (or perfect evenness). The Gini coefficient has a range from 0 to 1, high to low evenness, respectively. To ease interpretation, for each species we took the product of the vertical range extent, average tropospheric lapse rate, and the inverse of evenness (1 – Gini coefficient) to generate a single index for the elevational temperature gradient. We interpret this index as the overall strength of the elevational temperature gradient across each species' range: larger values indicate the presence of a strong and more evenly distributed gradient; lower values indicate the presence of a weak gradient due to either a small vertical range extent or an unevenly distributed vertical range extent.

### Latitudinal temperature gradients

Outside the tropics, average annual temperature declines on average 0.7°C for each degree of latitude in the Northern Hemisphere and on average 0.5°C for each degree of latitude in the Southern Hemisphere (Figure S2). With one degree of latitude equal to approximately 111 km, this translates to a decline of 1°C for every 150 km in the Northern Hemisphere and a decline of 1°C for every 197 km in the Southern Hemisphere.

We estimated the latitudinal temperature gradient within the breeding ranges of 9,014 species while controlling for variation in elevation. For our approach, we first found the average tropospheric lapse rate within each species' range. We then compiled gridded 30 arc-second average annual temperatures and elevations within each species' range. We chose annual average temperature because matching each species' breeding season distribution with their unique breeding season temperatures, especially for migratory species that only spend a few weeks to months on the breeding grounds, was not feasible. A likely consequence of using annual average temperature is that estimates of latitudinal temperature gradients for species that breed outside the tropics are likely to be steeper than they would be if estimates were based on the breeding season alone. Beyond these qualitative differences, using annual average temperature is unlikely to alter our qualitative conclusions. To control for the effect of elevation on temperature, we applied quantile regression to temperature as a function of elevation for 11 quantiles (0.0, 0.1,…0.9, 1.0). The residuals for the quantile whose slope most closely matched the tropospheric lapse rate estimated for each species was retained for further analysis. This approach allowed us to interpret latitudinal temperature gradients as the gradient along a fixed elevation within each species' range.

The relationship between these residuals and latitude was then assessed for each species using segmented linear regression [40]. If species' ranges occurred in both hemispheres, the range was first split in half at the equator and each portion was analyzed separately. We tested if the relationship between temperature and latitude contained one or two segments using Davie's test [41], which tests for a non-zero difference in the slope parameters of the segmented relationship. Relationships with two segments are more likely to occur if species' distributions are intersected by the Tropics of Cancer or Capricorn (Figure S2). When there was statistical evidence for a segmented relationship, we estimated a break point between segments and slope coefficients for each segment. When there was no statistical evidence for multiple segments, a single slope coefficient was estimated. Coefficients estimated south of the equator were multiplied by −1 to allow for comparison between the two hemispheres (i.e., latitudinal temperature gradients north and south of the equator are both negative; Figure S2). For species whose ranges were estimated by one slope coefficient, this coefficient was used to represent the overall gradient. For species whose ranges contained multiple slope coefficients, we used a weighted average to summarize the gradient. Weights were based on the latitudinal extent of the region where each slope coefficient was estimated. Based on this procedure, larger negative values indicate steeper latitudinal temperature gradients and values approaching zero indicate shallower latitudinal temperature gradients.

### Analysis

To summarize how steep elevational and latitudinal temperature gradients are on average within species' ranges globally, we first calculated the average elevational temperature gradient and median latitudinal temperature gradient within each equal-area cell for all species whose geographic ranges intersected that cell. To summarize how gradients differed for threatened and non-threatened species, we quantified how the gradients varied by species' median latitude. This analysis was implemented separately for species in each of the six biogeographical realms. For elevational temperature gradients, we used generalized additive models (GAM; [42]) with a Gaussian error distribution. For latitudinal temperature gradients, we used robust MM-type polynomial regression [43], [44]. GAM was selected because it adjusts automatically to the nonlinear associations observed between elevational temperature gradients and species' median latitude. This feature also supported the use of GAM to examine how species richness, range size, elevational extent, and Gini coefficient varied by species' median latitude for threatened and non-threatened species. Robust polynomial regression was selected for latitudinal temperature gradients because of the presence of many extreme outliers that were poorly modeled with GAM. In addition, the general curvilinear form of the relationship was well represented with a polynomial function. The order of the polynomial function was determined using robust Wald-type or deviance-type tests of nested model pairs. Median latitude was defined as the median of the full latitudinal extent of each species' range.

We used robust multiple regression to examine the ability of geographic and climatic predictors to explain the variability observed in species latitudinal and elevational temperature gradients. The four predictors were estimated within each species' geographic range and included average projected temperature anomaly, and geographic range size, shape, and orientation. Range size was log_10_ transformed before analysis. Range shape and orientation were estimated based on a principle component analysis (PCA) of the latitude and longitude of the grid cells contained within each species geographic range. PCA was conducted using a singular value decomposition of the centered data matrix. The square roots of the two eigenvalues were used to estimate range shape, where the minimum eigenvalue was divided by the maximum eigenvalue. Here, values approaching zero indicating elongated ranges and values approaching one indicating circular ranges. Range orientation was estimated based on the angle of the major axis of the PCA eigenvector from the equator. Values for range orientation were defined from 0° (parallel with the equator) to 90° (perpendicular to the equator). We evaluated our four predictors for multi-collinearity and singularity using variance inflation factors (VIFs) where predictors with VIF>5 indicate cause for concern and VIF>10 indicate the presence of significant collinearity [45]. We retained all four predictors after they were deemed to be statistically independent (VIF≤1.6).

To evaluate differences between threatened and non-threatened species, we included in addition to the four continuous predictors, a species' IUCN Red List status as threatened or non-threatened with extinction. For the analysis of the latitudinal temperature gradient, we also considered if the median latitude of a species' range was located within or outside the tropics. Median latitudes between 23.5°S and 23.5°N latitude were considered tropical. The tropical/extratropical classification was included because it defined a major feature of the gradient (Figure S2). In total, we examined eight robust multiple regression models that consisted of the two gradients, the four continuous predictors, associated categorical predictors, and all pairwise interactions.

All analyses were conducted in R version 3.0.1 [46]. Segmented regression was conducted using the ‘segmented’ library, robust regression using the ‘robust’ and ‘robustbase’ libraries, and GAM using the ‘mgcv’ library [42]. We used the default optimization procedure to estimate the degree of smoothing in the GAMs [42]. The Lorenz curves were estimated using the ‘ineq’ library and the Gini coefficients were estimated using the ‘reldist’ library.

## Results

**Figure 2:**
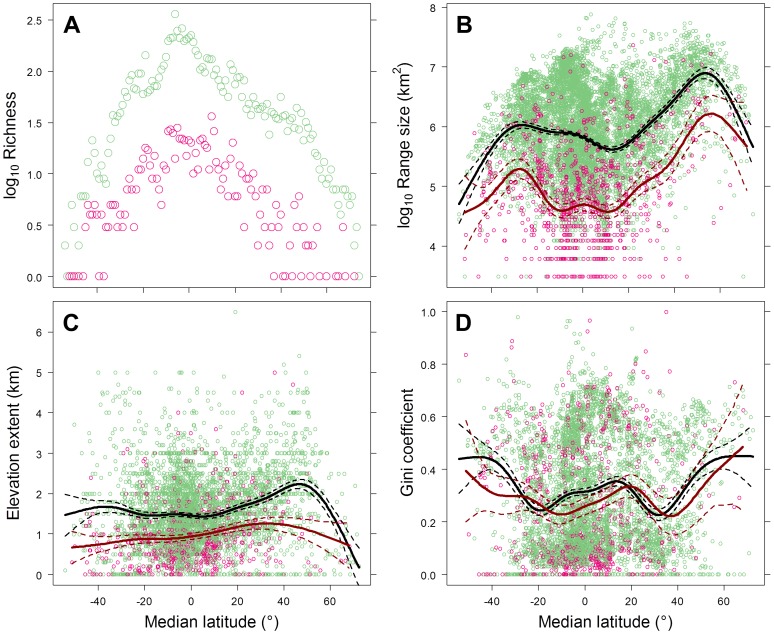
Plots summarizing geographic patterns for species' currently identified as threatened (red points; *n* = 878) and non-threatened (green points; *n* = 8,136) with extinction by the median latitude of each species' range. (A) The number of species' whose median latitudes occur within one degree latitudinal bands; (B) species' range size; (C) the elevational extent within species' ranges for 4,978 species with minimum and maximum elevation associations; and (D) the evenness of the distribution of 100 m elevations bands within species' ranges based on the Gini coefficient (0 =  high evenness; 1 =  low evenness; see Materials and Methods for details) for 4,978 species with minimum and maximum elevation associations. The trend lines are the fits of generalized additive models for species threatened (red) and non-threatened (black) with extinction.

Threatened and non-threatened bird species presented similar latitudinal trends in species richness with peaks for both groups occurring in the tropics (Figure 2A and Figure S3). Range sizes for threatened species were significantly smaller on average except for species located at the most extreme northern and southern latitudes (Figure 2B and Figure S3). For the 4,978 species with documented elevational associations, threatened species had smaller vertical range extents within their geographic ranges (Figure 2C and Figure S3) and threatened species had higher elevational evenness within their geographic ranges in the central tropics and more equivalent elevational evenness elsewhere (Figure 2D and Figure S3). Projected increases in temperature within species' ranges were highest for species located at higher latitudes in the Northern Hemisphere and lowest for species located at lower latitudes in the Southern Hemisphere (Figure S4).

**Figure 3:**
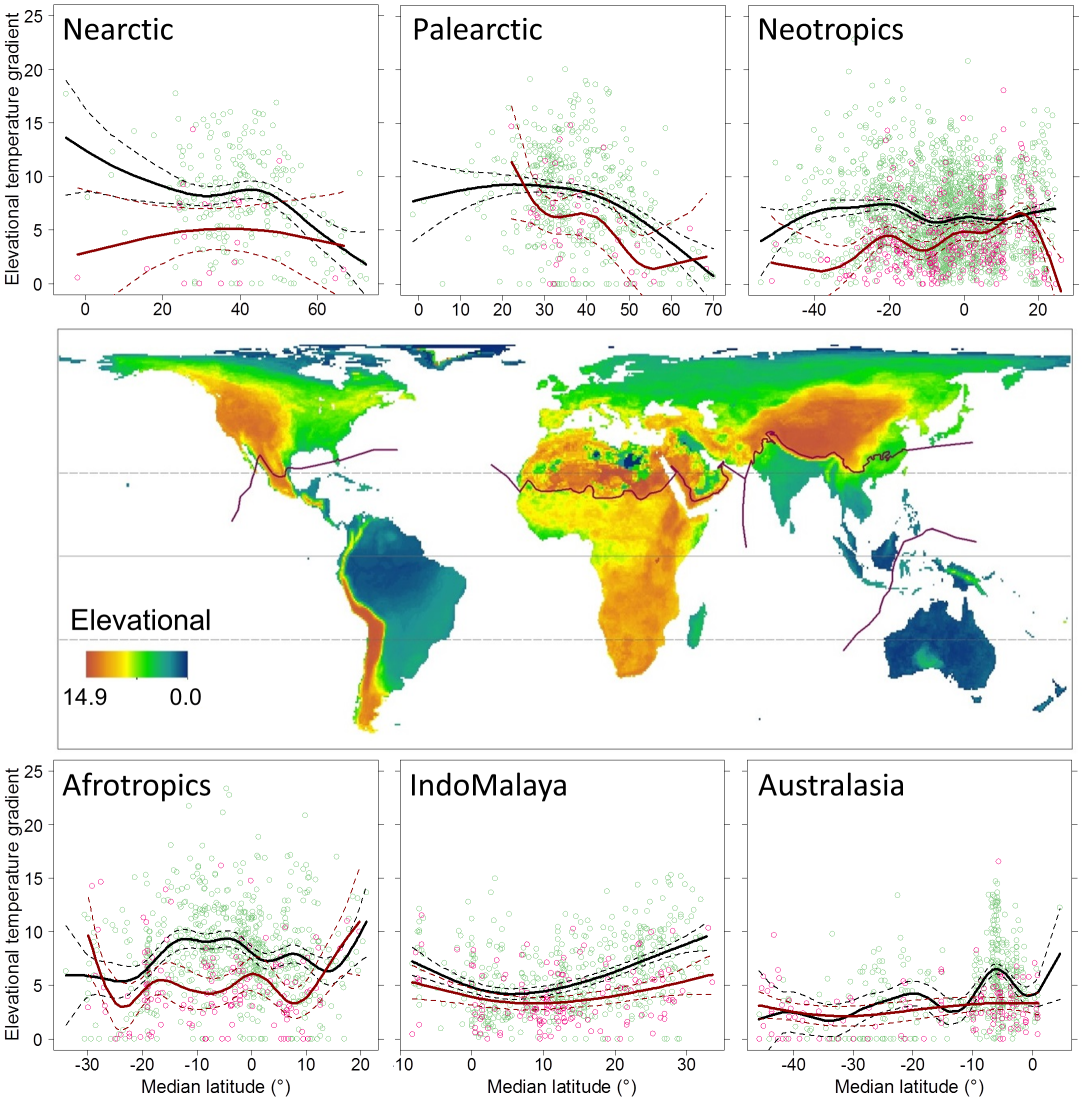
The elevation temperature gradient index summarized across species' geographic ranges (map) and summarized within six biogeographical realms (plots) as a function of the median latitude of each species' range. The temperature gradient was estimated spatially within species' current ranges (see Materials and Methods for details). In the plots, red points are threatened species (*n* = 766) and green points are non-threated species (*n* = 4,212). Trend lines are the fits of generalized additive models for threatened (red) and non-threatened species (black). The solid line is the equator and the dashed lines are the Tropics of Cancer and Capricorn (23.5°N and 23.5°S latitude, respectively).

There was strong geographic variation in the steepness of elevational temperature gradients (Figure 3). When considering the combined effects of elevational extent and elevational evenness (Figure S3) with tropospheric lapse rate, the strongest gradients were found for species located in Africa, the western part of North and South America, and the Tibetan Plateau region. In contrast, species in Australasia, the islands of IndoMalaya and the lowlands of the Neotropics had the weakest elevational temperature gradients (Figure 3). Threatened species were associated with weaker elevational temperature gradients across the six biogeographical realms, with the most significant differences occurring in the Neotropics and Afrotropics (Figure 3).

**Figure 4:**
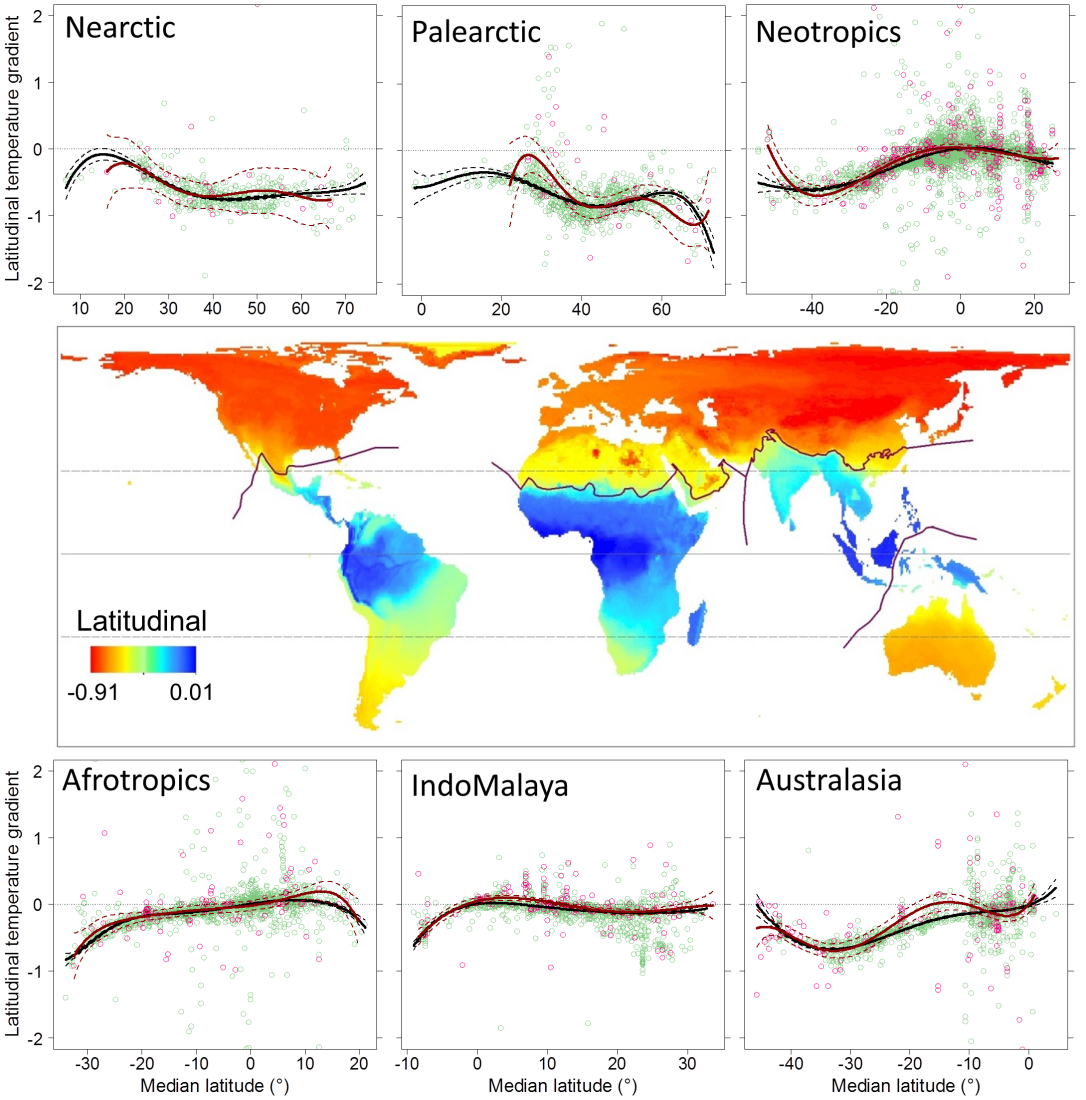
The latitudinal temperature gradient index summarized across species' geographic ranges (map) and summarized within six biogeographical realms (plots) as a function of the median latitude of each species' range. The temperature gradient was estimated spatially within species' current ranges (see Materials and Methods for details). In the plots, red points are threatened species (*n* = 878) and green points species non-threated species (*n* = 8,136). Trend lines are the fits of generalized additive models for threatened (red) and non-threatened species (black). The solid line is the equator and the dashed lines are the Tropics of Cancer and Capricorn (23.5°N and 23.5°S latitude, respectively).

After controlling for elevation, latitudinal temperature gradients were on average shallowest for species in the tropics and steepest for species outside the tropics (Figure 4). The steepest latitudinal temperature gradients occurred in the Northern Hemisphere. Australasia had the strongest gradients in the Southern Hemisphere, which did not match the strength observed in the Northern Hemisphere. There was no evidence that the latitudinal temperature gradients differed between threatened and non-threatened species across the six biogeographical realms (Figure 4).

Variation in elevational temperature gradients among species was poorly explained by our four predictors (Table 1 and Figure S5). Species with higher projected temperature increases and larger geographic ranges had steeper elevational temperature gradients, and this was the case for both threatened and non-threatened species. Species with geographic ranges that were elongated in shape and oriented perpendicular to the equator had stronger gradients, and this was more pronounced for non-threatened species.

**Table 1:** **Coefficients and test statistics from robust linear models examining predictors of elevational temperature gradients as estimated within the geographic ranges of 4,978 bird species.**

Model	Coef.	SE	*t*	*P*
Intercept	3.149	0.380	8.29	<0.001
Anomaly	0.976	0.108	9.05	<0.001
Threat	−3.117	0.895	−3.48	<0.001
Anomaly × Threat	0.344	0.273	1.26	0.2090
(*R* ^2^ = 0.07)				
Intercept	3.600	0.434	8.30	<0.001
Size	0.511	0.074	6.92	<0.001
Threat	−1.872	1.076	−1.74	0.082
Size × Threat	0.002	0.213	0.01	0.993
(*R* ^2^ = 0.06)				
Intercept	6.988	0.134	52.01	<0.001
Shape	−1.155	0.310	−3.73	<0.001
Threat	−2.940	0.295	−9.96	<0.001
Shape × Threat	1.580	0.707	2.23	0.026
(*R* ^2^ = 0.06)				
Intercept	5.845	0.105	55.85	<0.001
Orientation	0.018	0.002	8.18	<0.001
Threat	−1.824	0.280	−6.52	<0.001
Orientation × Threat	−0.014	0.005	−2.68	0.008
(*R* ^2^ = 0.06)				

Predictors include projected temperature Anomaly, geographic range Size, Shape, and Orientation and if the species is threatened with extinction (Threat).

Variation in latitudinal temperature gradients was better explained by our four predictors, with evidence for significant and strong differences between tropical and non-tropical species (Table 2 and Figure S6). Tropical species had weaker relationships for all four predictors with little evidence for differences between threatened and non-threatened species. For threatened and non-threatened extratropical species, species with higher projected temperature increases, larger ranges and ranges that were elongated in shape and oriented parallel with the equator had steeper latitudinal temperature gradients. For threatened extratropical species, gradients were even steeper for species with larger ranges.

**Table 2:** **Coefficients and test statistics from robust linear models examining predictors of latitudinal temperature gradients as estimated within the geographic ranges of 9,014 bird species.**

Model	Coef.	SE	*t*	*P*
Intercept	−0.288	0.021	−13.61	<0.001
Anomaly	−0.092	0.006	−16.52	<0.001
Tropics	0.199	0.028	7.14	<0.001
Threat	0.027	0.047	0.58	0.564
Tropics × Threat	0.028	0.019	1.48	0.138
Anomaly × Tropics	0.094	0.008	12.20	<0.001
Anomaly × Threat	−0.016	0.013	−1.21	0.225
(*R* ^2^ = 0.34)				
Intercept	0.148	0.045	3.32	0.001
Size	−0.120	0.007	−17.09	<0.001
Tropics	0.014	0.048	0.28	0.776
Threat	0.130	0.061	2.12	0.034
Tropics × Threat	−0.034	0.020	−1.66	0.098
Size × Tropics	0.080	0.008	10.38	<0.001
Size × Threat	−0.026	0.011	−2.47	0.014
(*R* ^2^ = 0.34)				
Intercept	−0.655	0.011	−61.97	<0.001
Shape	0.096	0.025	3.82	<0.001
Tropics	0.591	0.012	47.91	<0.001
Threat	−0.005	0.335	−0.02	0.988
Tropics × Threat	−0.032	0.061	−0.52	0.606
Shape × Tropics	−0.134	0.028	−4.70	<0.001
Shape × Threat	0.129	1.018	0.13	0.900
(*R* ^2^ = 0.35)				
Intercept	−0.687	0.006	−107.55	<0.001
Orientation	0.002	0.000	13.52	<0.001
Tropics	0.613	0.008	77.18	<0.001
Threat	−0.027	0.019	−1.42	0.155
Tropics × Threat	−0.026	0.019	−1.34	0.180
Orientation × Tropics	−0.002	0.000	−12.49	<0.001
Orientation × Threat	0.001	0.000	4.49	<0.001
(*R* ^2^ = 0.34)				

Predictors included projected temperature Anomaly and geographic range Size, Shape, and Orientation and if the species is tropical (Tropics) or threatened with extinction (Threat).

## Discussion

Species face unique challenges under climate change, with the availability and strength of range-shift opportunities along latitudinal or elevational temperature gradients playing a significant role in determining species' likelihood of persistence. In this study we provide the first estimation of these gradients for the world's avifauna relative to species' IUCN Red List category, geography, and projected exposure to climate change. Our approach expands upon current methods for estimating species' geographic climatic associations by measuring these two gradients individually using fine-grained environmental data, which more accurately captures their unique spatial structures. The application of this approach has the potential to increase biological realism and predictive quality in current large-scale modeling efforts by removing problematic assumptions and minimizing potential biases. Our findings suggest threatened species are at a distinct disadvantage globally based on their association with shallower elevational temperature gradients. Latitudinal temperature gradients are primarily relevant for species located outside the tropics, a region where few bird species occur year-round, including threatened species whose associations with latitudinal temperature gradients did not differ from non-threatened species. Our results also indicate a degree of correspondence between range-shift opportunities and climate change, in that species in regions that are projected to experience greater warming are more likely to be associated with steeper temperature gradients.

The strength of latitudinal temperature gradients is determined in part by the location, size, shape and orientation of species' geographic ranges. Not surprisingly, the strongest latitudinal temperature gradients were associated with species whose ranges occurred outside the tropics, especially at the higher latitudes in the North Hemisphere where the largest land masses are located. Species in these regions tend to have larger geographic ranges (see Figure 2B) and ranges that tend to be elongated parallel to the equator. As our results indicate, the strength of the latitudinal temperature gradient is of sufficient size within this region that geographic ranges with narrow latitudinal extents are still able to capture a significant gradient. In contrast, the spatial distribution of elevation temperature gradients reflects the form and extent of orographic features and how species' distributions intersect these features. We found that the strength of these gradients varied substantially across the globe and, unlike latitudinal temperature gradients, differed significantly between threatened and non-threatened species. In agreement with other studies [47], [48], species in Australia and the Islands of IndoMalaya stood out as particularly vulnerable, with some of the shallowest elevational gradients, followed by species located in the lowlands of the Neotropics. In contrast to latitudinal gradients, our ability to predict species' elevational temperature gradients was limited, reflecting the greater geographic heterogeneity in the location and structure of these gradients.

The strength of both temperature gradients was positively correlated with projected changes in temperature, but only for species outside the tropics with latitudinal temperature gradients. The greatest increases in temperature under climate change are projected to occur at the high northern latitudes (see Figure S4). Our findings therefore suggest a geographic correspondence with the greatest projected warming occurring in regions with the strongest latitudinal temperature gradients. However, few bird species occur at the high northern latitudes year round. The proportion of migratory species (*ca*. 19% of extant bird species [49]) within breeding communities increasing linearly as you travel north from the equator, approaching 100% at the highest latitudes [50]. Migratory species spend the bulk of their annual cycle in migration or on the non-breeding ranges at lower latitudes. Thus, very few bird species are likely to benefit substantially from this correspondence.

IUCN Red List categories of extinction risk are assigned using the Red List criteria, which have quantitative thresholds relating to population and range size, structure and trends [23]. Only 3.5% of threatened or near threatened bird species (77/2193) are assessed as threatened by climate change so severely that the global population may be declining rapidly or very rapidly over three generations, and hence potentially contributing to their Red List category through the A criterion. Of these, only 26 (1.2%) actually qualify under the A criterion. Our findings suggest this proportion is likely to increase, especially for threatened species associated with shallow elevational temperature gradients. As confirmed in our analysis, threatened species occur primarily in the tropics and their distributions are defined by smaller geographic ranges and elevational extents. Hence, threatened species may be at a geographic disadvantage under climate change with limited associations with elevational or latitudinal temperature gradients, although we found no evidence for the latter. Our findings suggest threatened species that occur in the central tropics have higher elevational evenness across their range, which is a likely consequence of their distributions occurring on steep montane slopes outside of lowland areas, which should result in stronger elevational temperature gradients for these species. However, our analysis suggests that the differences in elevational evenness between threatened and non-threatened species was not substantial enough to overcome the smaller elevational extents contained within threatened species' geographic ranges. Thus, our findings suggest the weaker elevational temperature gradients identified for threatened species is due in large part to a consistent association with smaller elevational extents within their ranges.

When considering both gradients in combination, two regions stand out as having both strong elevational and latitudinal temperature gradients: western North America and Central Asia (see Figures 3–4). These results suggest species in these regions will have greater opportunities to track their climatic niches geographically. Depending on how these temperature gradients intersect spatially within species' ranges, some combination of these gradients could be used. Similarly, two regions stand out as having both shallow elevational and latitudinal temperature gradients: the islands of IndoMalaya and the Amazon Basin in South America (see Figures 3–4). Species in these regions are likely to develop substantial range-shift gaps under climate change [10], resulting in limited opportunities to track their climatic niche geographically. Bird species occurring in the Amazon Basin are also considered to have low adaptive capacity, which may increase their vulnerability to climate change [51].

Species in tropical montane regions are more likely to have restricted lateral and vertical distributions [48], [52] and are also more likely to be threatened with extinction (see Figure S3). Even though in many cases these species occur in some of the most extensive montane systems in the world, our findings suggest that the overall steepness of elevational gradients is constrained by their limited representation within species' geographic ranges. Local climatic heterogeneity, however, may allow for microclimatic adjustments that could compensate for broader climatic trends [53]–[56]. The level of climatic heterogeneity is determined by local topographic variation and also by species' habitat associations [57]. For example, species in forested habitats may have greater opportunities for microclimatic adjustments relative to species in open habitats [58]. In general, however, behavior resulting in microclimatic adjustments might not be sufficient to address the full consequences of climate change [59].

Even if latitudinal and elevational temperature gradients are readily available, dispersal along these gradients is not guaranteed. Dispersal abilities are determined in large part by species' morphological, physiological and behavioral characteristics and adaptations [60], [61], all of which are likely to be altered under rapid climate change [62]. Dispersal under climate change may be constrained by unsuitable habitat, population or habitat fragmentation [58], [63], or interspecific competition [64]. In addition, gene flow that occurs in association with dispersal can both support and hinder range-shift potential [65]. Gene flow among populations at the leading edge of the range has been found to benefit fitness, whereas gene flow from the interior to the leading edge has been identified as maladaptive [66], [67]. Dispersal abilities also vary tremendously among species and between taxa [60]. Even highly vagile taxa such as birds do not necessarily have the full complement of behavioral or physiological characteristics needed to support successful dispersal, which is considered particularly relevant for tropical species [68], [69]. Although there is evidence tropical montane birds have moved upslope under climate change [70], the low annual variation in temperatures within the tropics relative to temperate regions may result in stronger physiological barriers to dispersal for tropical species [52], [71], [72]. When tropical habitats are fragmented through human activities – a process that is occurring at an increasing rate – dispersal can become even more constrained [73], [74]. If species' responses are hindered by dispersal limitations or interspecific competition, as is expected for many tropical montane bird communities, substantial ecological disruptions are likely to occur [75].

In contrast, species that breed outside the tropics that are also migratory [76] or have larger geographic ranges tend to have stronger natal dispersal abilities [77]–[79]. Thus migratory species that are broadly distributed and breed outside the tropics might be in a better position to track elevational or latitudinal temperature gradients. However, exceptions exist [80] and factors that are strongly correlated with dispersal abilities might not be similarly correlated with broad-scale distributional responses under climate change [81]. Nevertheless, current evidence indicates distributional responses for birds along latitudinal [7] and elevational temperature gradients [82] are lagging behind warming trends, suggesting stronger responses could develop as climate change progresses and time lags are overcome [7].

We estimated temperature gradients in this study using relatively high resolution environmental data that was finer than the resolution of the species' range data [30]. This grain mismatch resulted in temperature gradients likely being estimated over fine-grain sections of species' ranges where probability of occurrence is low. Our main results focus on the geographic form of the gradient across the spatial breadth of species' distributions, but future work will benefit from further examination of potential biases this grain mismatch may have on comparisons. How patterns of occurrence are structured within species' ranges, based on proportion of occupancy and level of aggregation [83], may affect the chances of successful dispersal along these gradients. For example, sparse or fragmented patterns of occurrence may diminish the chances of successful dispersal, while more continuous patterns of occurrence may improve them. Alternatively, large contiguous regions with low probabilities of occurrence might contain the steepest gradients with very little dispersal value, as when lowland species' distributions border mountain systems. These remaining uncertainties highlight the need for advancing the spatial grain of our global biodiversity knowledge and for the necessary collaborative data mobilization, integration and quality control to realize this [31].

In addition to temperature, other climate factors and conditions are likely to be important in determining the strength and importance of latitudinal and elevation temperature gradients in defining range-shift potential. Two that have received particular attention are changes in precipitation regimes and changes in climatic variability. In contrast to temperature, precipitation has more complex spatial and temporal variability and more complex associations with latitudinal and elevational gradients. Projected changes in precipitation are also more ambiguous [2], which adds uncertainty to current estimates on how species are likely to respond to climate change [84]. A second factor is climatic variability, which is projected to increase through an increased frequency of extreme climate events [2]. Temporal climatic variability is considered an important factor defining species' distributions limits [85] and increasing climatic variability can have varied effects on species' populations [86] including the potential to hinder range shifts [87] and, especially under rapid climate change, increase extinction risk [88].

Additional research is needed to improve our understanding of how latitudinal and especially elevational temperature gradients are structured within species' ranges and the ability of species, especially tropical, to take advantage of these gradients. In particular, studies using higher resolution climate data (e.g., [22]) for species with varying spatial patterns of occurrence and forms of elevational or latitudinal gradients would be informative. Birds are often used as a model biological system due to high data availability, but less vagile taxa also need to be considered such as plants or vertebrate ectotherms that have alternative modes of dispersal and different physiological and behavior capacities. With birds, examining how these gradients are structured within non-breeding ranges, where migratory birds tend to occur during the majority of the annual cycle and are typically less well studied, would be beneficial [9]. Efforts to better understand the role of precipitation and climate variability in defining range-shift potential along latitudinal and elevational temperature gradients would support more comprehensive inferences.

Current efforts to project species' distributions or to estimate movement corridors under climate change often assume perfect and complete dispersal along temperature gradients (e.g., [89]). Temperature gradients are typically estimated using coarse-grained interpolated weather-station data based on current climatic conditions where the contrasting spatial forms of latitudinal and especially elevational temperature gradients are often poorly summarized [35], [36]. These methods may exaggerate estimated dispersal distances [90], especially at high northern latitudes where latitudinal temperature gradients are projected to weaken under climate change [12]. These considerations in addition to the correlative nature of these models have promoted the development of more mechanistic or process-based methods intended to improve biological realism and minimize bias in current projections [91]. Our findings suggest the quality of model predictions may be improved by considering the spatial form and strength of species' demographic responses [92] to projected latitudinal and elevational temperature gradients within species' ranges and along possible movement corridors

Lastly, in order to maximize range-shift potential under climate change, conservation efforts are needed to maximize the size of areas of contiguous habitat, minimize the extent of habitat fragmentation and promote the permeability of the matrix of habitats around key sites. In particular, efforts to assess and promote connectivity at broad geographic extents are likely to be the most beneficial (e.g., [93], [94]) where existing gradients are represented as continuously as possible across space [95]. The more thoroughly this can be achieved, the greater the potential for species to track changing climatic conditions based on their unique dispersal abilities and ecological and environmental requirements.

## Conclusions

The availability of opportunities for geographic responses under climate change reflects the interplay between each species' geographic distribution and their associated latitudinal and elevational temperature gradients (with the extent to which these opportunities will be utilized depending on each species' dispersal abilities and the consequences of changing gene flow). The distribution of these gradients across the world follows clearly defined geographic patterns, but their availability depends on how they intersect with species' distributions. The location, size, and shape of species' geographic range and projected changes in temperature all contain a degree of relevance in determining the strength of these temperature gradients. However, the strength of latitudinal and elevational temperature gradients varied considerably among species, constraining our ability to make robust predictions for all regions globally. Nevertheless, we found that species that are currently at risk of extinction are consistently at the greatest disadvantage, especially based on the availability of elevational temperature gradients.

## Supporting Information

Figure S1 **Global patterns of elevation based on the USGS global digital elevation model (GTOPO30) gridded at a 30 arc-second resolution (** ***ca*** **. 1 km at the equator).** The solid grey line is the equator and the dashed grey lines are the Tropics of Cancer and Capricorn (23.5°N and 23.5°S latitude, respectively).
(PDF)

Figure S2 **Global terrestrial annual mean temperature for 1950–2000 (WorldClim) averaged within 0.1 degree latitudinal bands.** The dotted lines indicate the Tropics of Cancer and Capricorn (23.5°N and 23.5°S latitude, respectively).
(PDF)

Figure S3 **Maps summarizing features of the global distribution of terrestrial avifauna across an equal area grid (cell area: 3,091 km^2^).** (A) The richness of all species (*n* = 9,014) and (B) the richness of species threatened with extinction (*n* = 878). (C) Geographic range size for all species (*n* = 9,014) and (D) elevational extent for 4,978 species with minimum and maximum elevation associations. (E) The evenness of the distribution of elevation within each species' range based on the Gini coefficient and Lorenz curve (0 =  high evenness; 1 =  low evenness) for 4,978 species with minimum and maximum elevation associations. Maps are displayed using the Behrmann equal-area cylindrical projection and the color ramps use Jenk's natural break classification. The solid grey line is the equator and the dashed grey lines are the Tropics of Cancer and Capricorn (23.5°N and 23.5°S latitude, respectively). Red lines distinguish boundaries between six biogeographical realms.
(PDF)

Figure S4 **Projected temperature anomalies summarized across species' geographic ranges (map) and summarized within six biogeographical realms (plots) as a function of the median latitude of each species' range.** In the plots, red points are threatened species (*n* = 878) and green points species non-threatened species (*n* = 8,136). Trend lines are the fits of generalized additive models for threatened (red) and non-threatened species (black). The solid line is the equator and the dashed lines are the Tropics of Cancer and Capricorn (23.5°N and 23.5°S latitude, respectively).
(PDF)

Figure S5 **Fit of robust linear regression models to four predictors of elevational temperature gradients estimated within the geographic ranges of 4,978 bird species.** Red points and lines are threatened species (*n* = 766) and green points and black line are non-threatened species (*n* = 4,212).
(PDF)

Figure S6 **Fit of robust linear regression models to four predictors of latitudinal temperature gradients estimated within the geographic ranges of 9,014 bird species.** Green points and lines are for tropical species (*n* = 6,720), brown points and lines are for extratropical species (*n* = 2,294). The solid lines are the fits for non-threatened species (*n* = 4,214) and the dashed lines are the fits for threatened species (n = 766).
(PDF)

Table S1 The 9,014 species considered in the analysis and their estimated latitudinal and elevational temperature gradients, designated biogeographical realm and threat status (1 =  threatened and 0 =  non-threatened with extinction), projected temperature anomaly, and geographic range size, shape and orientation.
(PDF)
