# Correction: Range-Wide Latitudinal and Elevational Temperature Gradients for the World's Terrestrial Birds: Implications under Global Climate Change

**DOI:** 10.1371/journal.pone.0109448

**Published:** 2014-09-24

**Authors:** The PLOS ONE Staff

A NSF grant was omitted from this article. Please view the complete funding statement below:

This work was funded through a postdoctoral fellowship at the Cornell Lab of Ornithology, and a NSF grant IIS-1125098 . The funders had no role in study design, data collection and analysis, decision to publish, or preparation of the manuscript.
