# Supplementary material for: Range-Wide Latitudinal and Elevational Temperature Gradients for the World's Terrestrial Birds: Implications under Global Climate Change
Source: PLoS One. 2014 May 22;9(5):e98361. doi: 10.1371/journal.pone.0098361 (PMC4031198; doi:10.1371/journal.pone.0098361)
Supplement: Figure S1 — Global patterns of elevation based on the USGS global digital elevation model (GTOPO30) gridded at a 30 arc-second resolution ( ca . 1 km at the equator). The solid grey line is the equator and the dashed grey lines are the Tropics of Cancer and Capricorn (23.5°N and 23.5°S latitude, respectively). (PDF) [file pone.0098361.s001.pdf]

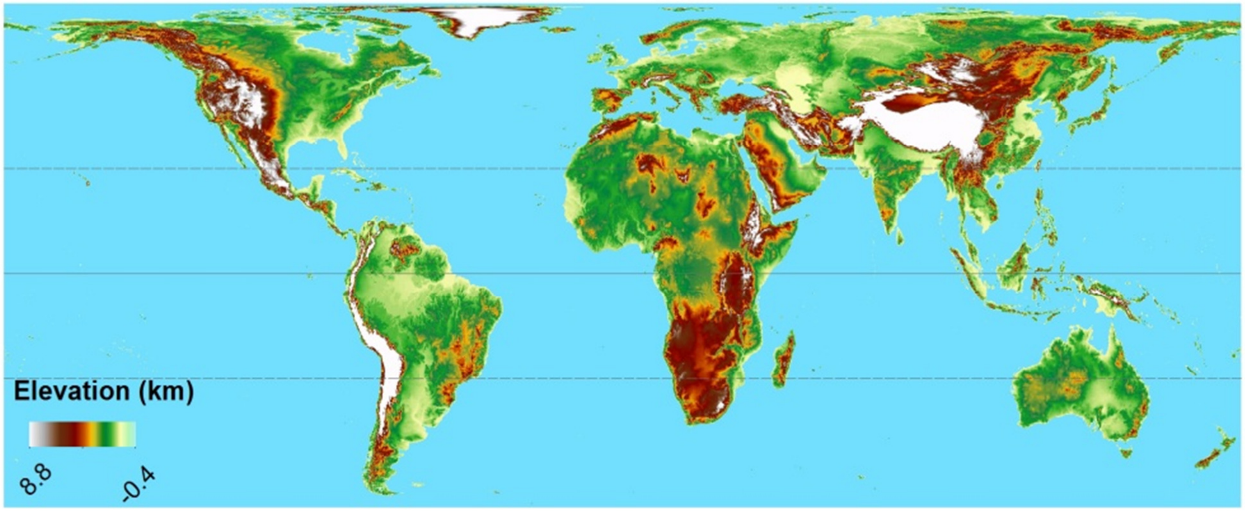


**Figure S1. Global patterns of elevation based on the USGS global digital elevation model (GTOPO30) gridded at a 30 arc-second resolution (*ca*. 1 km at the equator).** The solid grey line is the equator and the dashed grey lines are the Tropics of Cancer and Capricorn (23.5°N and 23.5°S latitude, respectively).
