# Supplementary material for: Range-Wide Latitudinal and Elevational Temperature Gradients for the World's Terrestrial Birds: Implications under Global Climate Change
Source: PLoS One. 2014 May 22;9(5):e98361. doi: 10.1371/journal.pone.0098361 (PMC4031198; doi:10.1371/journal.pone.0098361)
Supplement: Figure S2 — Global terrestrial annual mean temperature for 1950–2000 (WorldClim) averaged within 0.1 degree latitudinal bands. The dotted lines indicate the Tropics of Cancer and Capricorn (23.5°N and 23.5°S latitude, respectively). (PDF) [file pone.0098361.s002.pdf]

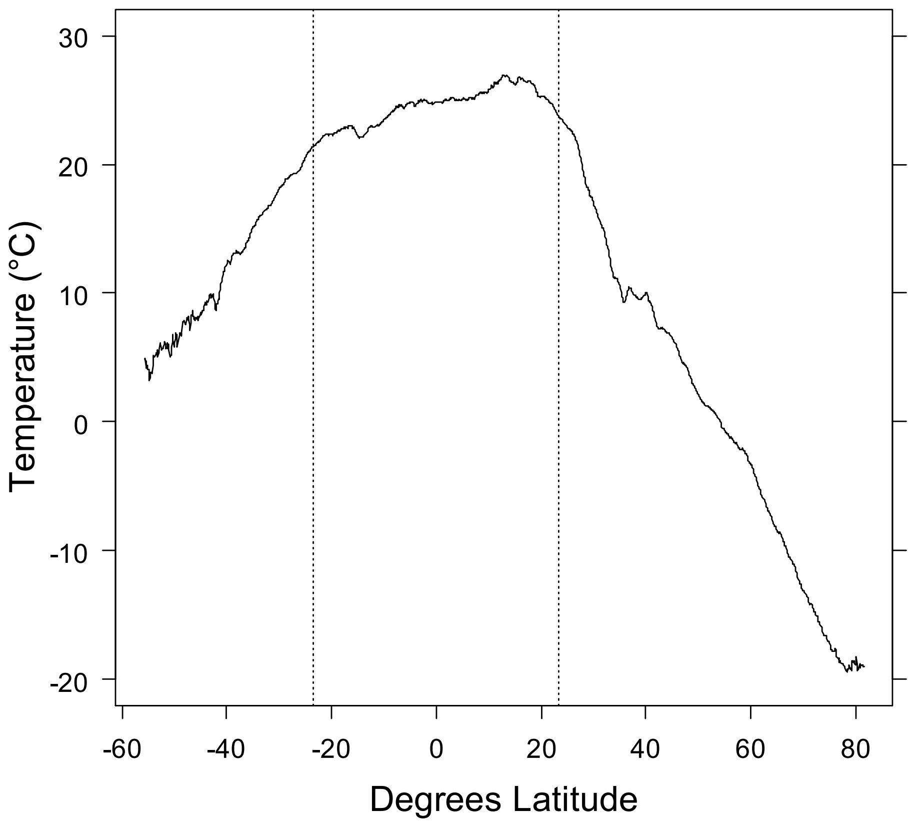

Figure S2. Global terrestrial annual mean temperature for 1950-2000 (WorldClim) averaged within 0.1 degree latitudinal bands. The dotted lines indicate the Tropics of Cancer and Capricorn (23.5°N and 23.5°S latitude, respectively).
