# Supplementary material for: Range-Wide Latitudinal and Elevational Temperature Gradients for the World's Terrestrial Birds: Implications under Global Climate Change
Source: PLoS One. 2014 May 22;9(5):e98361. doi: 10.1371/journal.pone.0098361 (PMC4031198; doi:10.1371/journal.pone.0098361)
Supplement: Figure S3 — Maps summarizing features of the global distribution of terrestrial avifauna across an equal area grid (cell area: 3,091 km2). (A) The richness of all species (n = 9,014) and (B) the richness of species threatened with extinction (n = 878). (C) Geographic range size for all species (n = 9,014) and (D) elevational extent for 4,978 species with minimum and maximum elevation associations. (E) The evenness of the distribution of elevation within each species' range based on the Gini coefficient and Lorenz curve (0 = high evenness; 1 = low evenness) for 4,978 species with minimum and maximum elevation associations. Maps are displayed using the Behrmann equal-area cylindrical projection and the color ramps use Jenk's natural break classification. The solid grey line is the equator and the dashed grey lines are the Tropics of Cancer and Capricorn (23.5°N and 23.5°S latitude, respectively). Red lines distinguish boundaries between six biogeographical realms. (PDF) [file pone.0098361.s003.pdf]

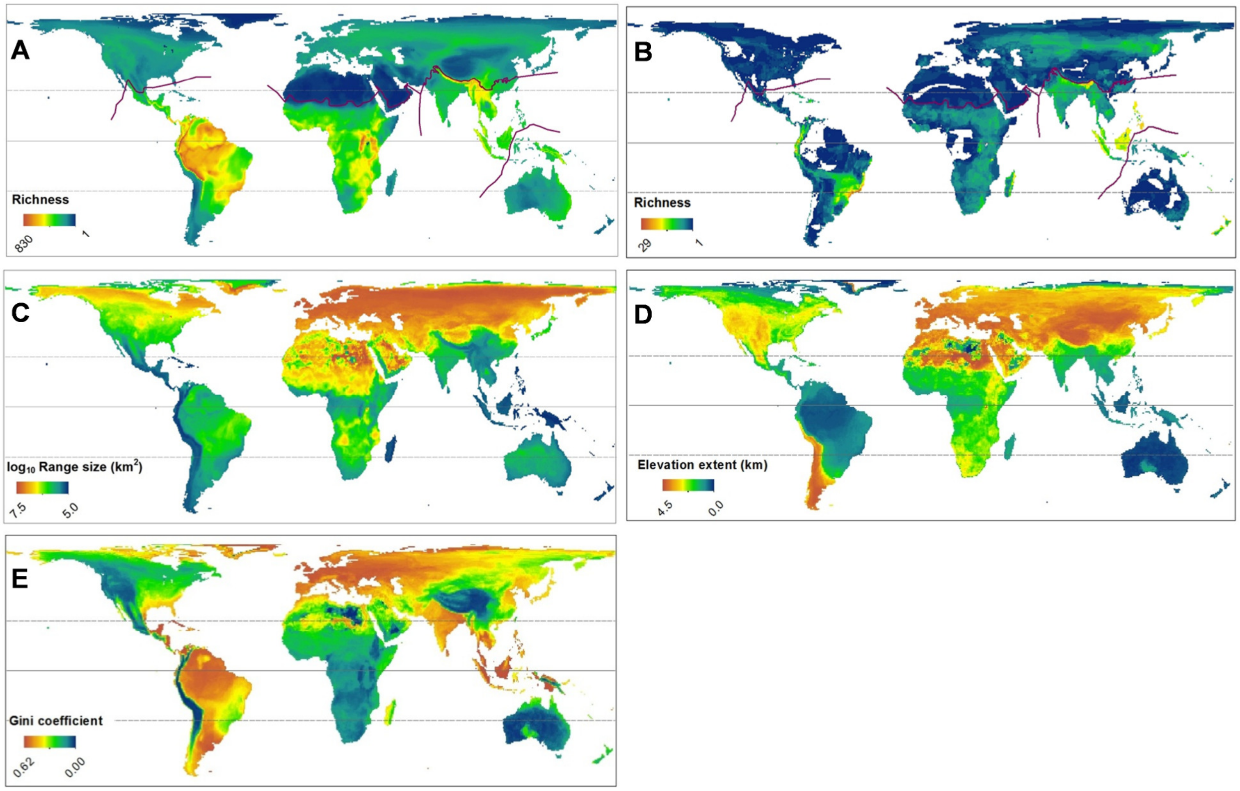

Figure S3. Maps summarizing features of the global distribution of terrestrial avifauna across an equal area grid (cell area: 3,091 km^2). (A) The richness of all species (n = 9,014) and (B) the richness of species threatened with extinction (n = 878). (C) Geographic range size for all species (n = 9,014) and (D) elevational extent for 4,978 species with minimum and maximum elevation associations. (E) The evenness of the distribution of elevation within each species' range based on the Gini coefficient and Lorenz curve (0 = high evenness; 1 = low evenness) for 4,978 species with minimum and maximum elevation associations. Maps are displayed using the Behrmann equal-area cylindrical projection and the color ramps use Jenk's natural break classification. The solid grey line is the equator and the dashed grey lines are the Tropics of Cancer and Capricorn (23.5°N and 23.5°S latitude, respectively). Red lines in A and B distinguish boundaries between six biogeographical realms.
