# Supplementary material for: Range-Wide Latitudinal and Elevational Temperature Gradients for the World's Terrestrial Birds: Implications under Global Climate Change
Source: PLoS One. 2014 May 22;9(5):e98361. doi: 10.1371/journal.pone.0098361 (PMC4031198; doi:10.1371/journal.pone.0098361)
Supplement: Figure S4 — Projected temperature anomalies summarized across species' geographic ranges (map) and summarized within six biogeographical realms (plots) as a function of the median latitude of each species' range. In the plots, red points are threatened species (n = 878) and green points species non-threatened species (n = 8,136). Trend lines are the fits of generalized additive models for threatened (red) and non-threatened species (black). The solid line is the equator and the dashed lines are the Tropics of Cancer and Capricorn (23.5°N and 23.5°S latitude, respectively). (PDF) [file pone.0098361.s004.pdf]

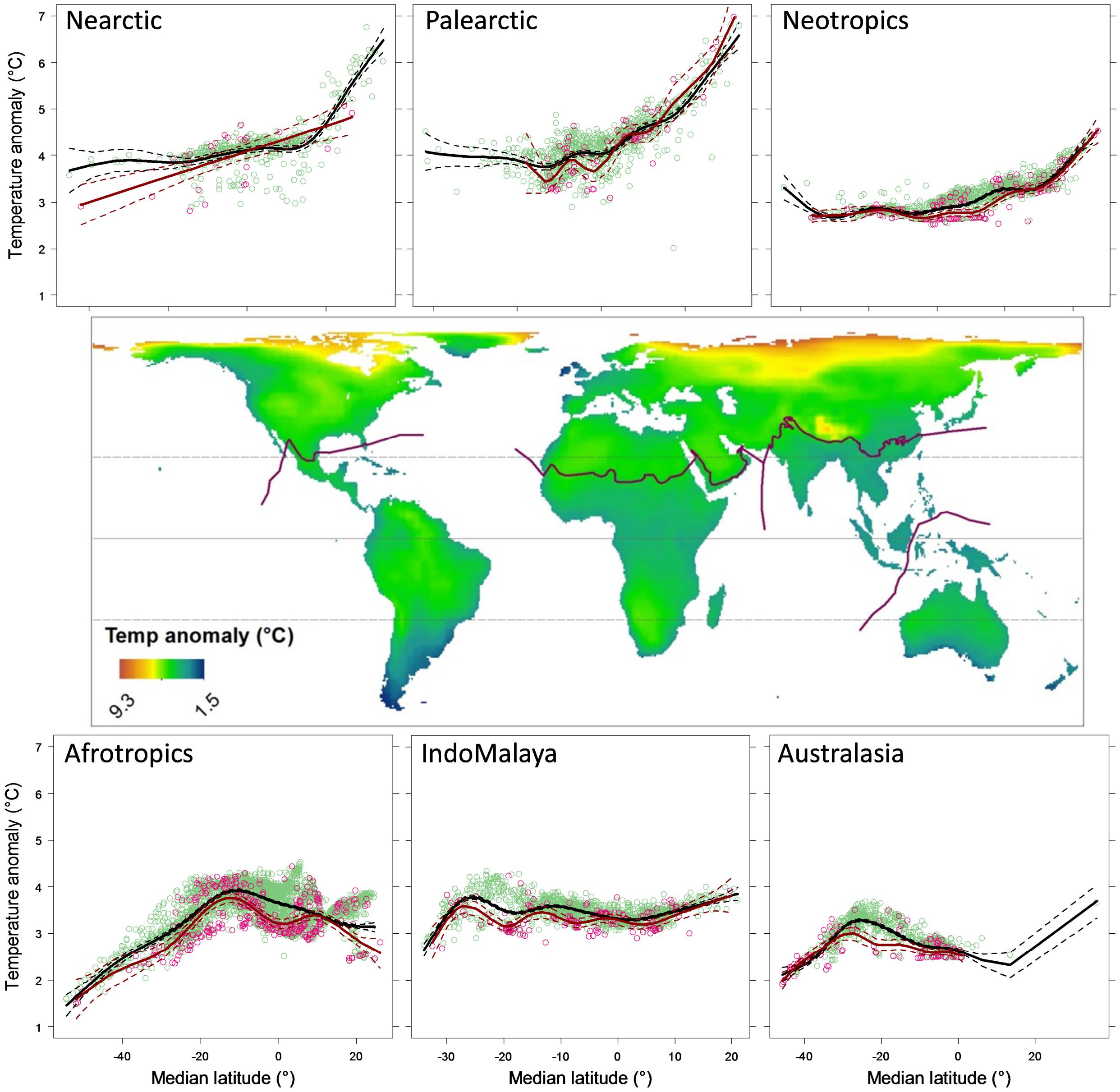

Figure S4. Projected temperature anomalies summarized across species' geographic ranges (map) and summarized within six biogeographical realms (plots) as a function of the median latitude of each species' range. In the plots, red points are threatened species (*n* = 878) and green points species non-threatened species (*n* = 8,136). Trend lines are the fits of generalized additive models for threatened (red) and non-threatened species (black). The solid line is the equator and the dashed lines are the Tropics of Cancer and Capricorn (23.5°N and 23.5°S latitude, respectively).
