# Supplementary material for: Range-Wide Latitudinal and Elevational Temperature Gradients for the World's Terrestrial Birds: Implications under Global Climate Change
Source: PLoS One. 2014 May 22;9(5):e98361. doi: 10.1371/journal.pone.0098361 (PMC4031198; doi:10.1371/journal.pone.0098361)
Supplement: Figure S5 — Fit of robust linear regression models to four predictors of elevational temperature gradients estimated within the geographic ranges of 4,978 bird species. Red points and lines are threatened species (n = 766) and green points and black line are non-threatened species (n = 4,212). (PDF) [file pone.0098361.s005.pdf]

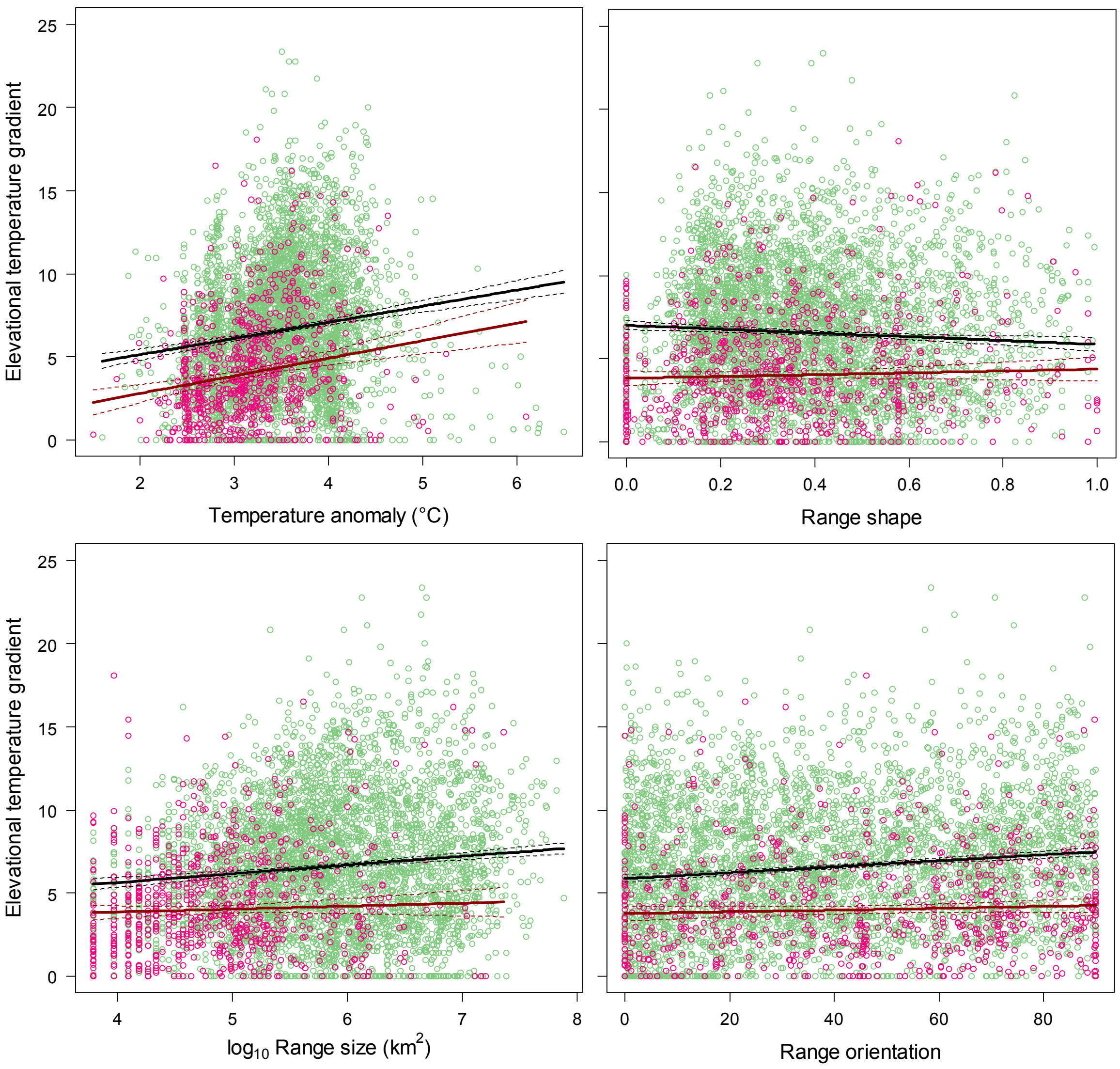

Figure S5. Fit of robust linear regression models to four predictors of elevational temperature gradients estimated within the geographic ranges of 4,978 bird species. Red points and lines are threatened species (n = 766) and green points and black line are non-threatened species (n = 4,212).
