# Supplementary material for: Range-Wide Latitudinal and Elevational Temperature Gradients for the World's Terrestrial Birds: Implications under Global Climate Change
Source: PLoS One. 2014 May 22;9(5):e98361. doi: 10.1371/journal.pone.0098361 (PMC4031198; doi:10.1371/journal.pone.0098361)
Supplement: Figure S6 — Fit of robust linear regression models to four predictors of latitudinal temperature gradients estimated within the geographic ranges of 9,014 bird species. Green points and lines are for tropical species (n = 6,720), brown points and lines are for extratropical species (n = 2,294). The solid lines are the fits for non-threatened species (n = 4,214) and the dashed lines are the fits for threatened species (n = 766). (PDF) [file pone.0098361.s006.pdf]

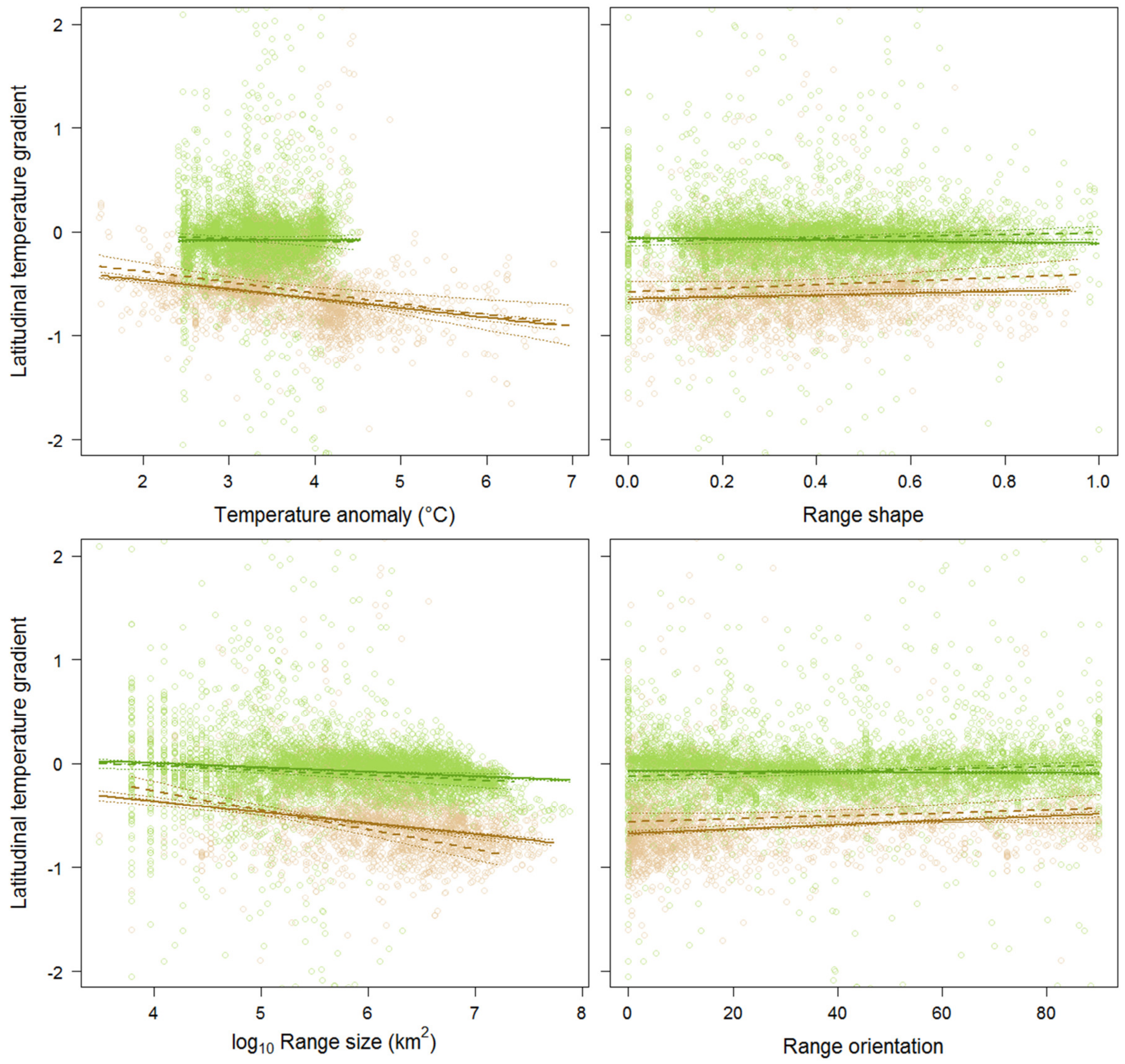

Figure S6. Fit of robust linear regression models to four predictors of latitudinal temperature gradients estimated within the geographic ranges of 9,014 bird species. Green points and lines are for tropical species (*n* = 6,720), brown points and lines are for extratropical species (*n* = 2,294). The solid lines are the fits for non-threatened species (*n* = 4,214) and the dashed lines are the fits for threatened species (n = 766).
