# Supplementary material for: Range-Wide Latitudinal and Elevational Temperature Gradients for the World's Terrestrial Birds: Implications under Global Climate Change
Source: PLoS One. 2014 May 22;9(5):e98361. doi: 10.1371/journal.pone.0098361 (PMC4031198; doi:10.1371/journal.pone.0098361)
Supplement: Table S1 — The 9,014 species considered in the analysis and their estimated latitudinal and elevational temperature gradients, designated biogeographical realm and threat status (1 = threatened and 0 = non-threatened with extinction), projected temperature anomaly, and geographic range size, shape and orientation. (PDF) [file pone.0098361.s007.pdf]

Table S1. The 9,014 species considered in the analysis and their estimated latitudinal and elevational temperature gradients, designated biogeographical realm and threat status (1 = threatened and 0 = non-threatened with extinction), projected temperature anomaly, and geographic range size, shape and orientation.

Family	Species	Realm	Threat	Latitude	Elevation	Anomaly	Size	Shape	Orientation
Acanthisittidae	*Acanthisitta chloris*	Australasia	0	-0.4904	-	2.32	0.7305	0.1841	41.61
Acanthisittidae	*Xenicus gilviventris*	Nearctic	1	-3.7814	-	4.44	0.7586	0.6847	79.20
Acanthizidae	*Acanthiza apicalis*	Australasia	0	-0.6634	-	3.34	0.8221	0.4077	0.65
Acanthizidae	*Acanthiza chrysorrhoa*	Australasia	0	-0.6776	-	3.25	0.8253	0.4032	3.20
Acanthizidae	*Acanthiza ewingii*	Australasia	0	-0.3137	-	2.17	0.6804	0.6686	14.77
Acanthizidae	*Acanthiza inornata*	Australasia	0	-0.9299	-	2.63	0.7172	0.4944	65.20
Acanthizidae	*Acanthiza iredalei*	Australasia	0	-0.8038	-	2.94	0.7844	0.1828	15.28
Acanthizidae	*Acanthiza katherina*	Australasia	0	-0.1076	6.2798	3.20	0.6921	0.6271	87.70
Acanthizidae	*Acanthiza lineata*	Australasia	0	-0.6030	-	3.10	0.7821	0.5078	50.81
Acanthizidae	*Acanthiza murina*	Australasia	0	-0.4254	7.2215	2.85	0.7291	0.1614	24.60
Acanthizidae	*Acanthiza nana*	Australasia	0	-0.5966	-	3.24	0.8015	0.6671	73.87
Acanthizidae	*Acanthiza pusilla*	Australasia	0	-0.5778	3.9924	3.06	0.7837	0.4697	65.46
Acanthizidae	*Acanthiza reguloides*	Australasia	0	-0.5952	-	3.23	0.7987	0.5792	79.13
Acanthizidae	*Acanthiza robustirostris*	Australasia	0	-0.6656	-	3.51	0.8042	0.3316	1.58
Acanthizidae	*Acanthiza uropygialis*	Australasia	0	-0.6508	-	3.33	0.8263	0.3636	4.52
Acanthizidae	*Acanthornis magna*	Australasia	0	-0.3201	-	2.17	0.6784	0.6765	14.38
Acanthizidae	*Aphelocephala leucopsis*	Australasia	0	-0.8268	-	3.25	0.8196	0.3244	8.42
Acanthizidae	*Aphelocephala nigricincta*	Australasia	0	-0.6771	-	3.52	0.7971	0.4333	12.68
Acanthizidae	*Aphelocephala pectoralis*	Australasia	0	-0.6581	-	3.11	0.7367	0.6547	32.81
Acanthizidae	*Calamanthus campestris*	Neotropics	0	0.2228	5.7684	3.31	0.7695	0.4766	75.40
Acanthizidae	*Calamanthus fuliginosus*	Australasia	0	-0.1204	5.1957	2.78	0.7735	0.4377	32.26
Acanthizidae	*Chthonicola sagittatus*	Australasia	0	-0.5342	-	3.37	0.8362	0.5654	7.09
Acanthizidae	*Crateroscelis murina*	Neotropics	0	-3.8620	8.3764	4.10	0.6763	0.2395	25.01
Acanthizidae	*Crateroscelis nigrorufa*	Neotropics	0	6.9733	7.3412	4.02	0.7008	0.2230	33.87
Acanthizidae	*Crateroscelis robusta*	Neotropics	0	-0.3131	11.4017	3.34	0.6891	0.2920	85.38
Acanthizidae	*Gerygone chloronota*	Neotropics	0	-0.5645	12.5179	3.44	0.7775	0.1668	76.38
Acanthizidae	*Gerygone chrysogaster*	Neotropics	0	-11.5251	7.3218	4.03	0.6876	0.4549	43.89
Acanthizidae	*Gerygone cinerea*	Neotropics	0	-0.0916	12.5078	3.98	0.7729	0.2523	50.99
Acanthizidae	*Gerygone fusca*	Neotropics	0	-0.7524	-	3.12	0.5428	-	-
Acanthizidae	*Gerygone levigaster*	Neotropics	0	-0.2217	-	3.64	0.8414	0.6540	59.09
Acanthizidae	*Gerygone mouki*	Nearctic	1	-0.3029	0.0000	2.82	0.6562	0.0883	53.56
Acanthizidae	*Gerygone olivacea*	Neotropics	1	-0.1626	1.1815	3.59	0.6891	0.4891	82.96
Acanthizidae	*Gerygone palpebrosa*	Neotropics	0	-0.0712	6.7506	3.73	0.7291	0.2015	63.29
Acanthizidae	*Gerygone ruficollis*	Neotropics	0	-0.0819	-	3.44	0.7797	0.3064	23.90
Acanthizidae	*Gerygone tenebrosa*	Neotropics	0	0.0717	-	3.14	0.7419	0.2609	66.12
Acanthizidae	*Hylacola cauta*	Afrotropics	0	0.0312	12.6647	3.43	0.8023	0.5100	13.76
Acanthizidae	*Hylacola pyrrhopygia*	Australasia	0	-0.5325	-	3.37	0.8303	0.5432	12.53
Acanthizidae	*Mohoua albicilla*	Australasia	0	-0.0700	8.7055	2.75	0.7517	0.1989	14.39
Acanthizidae	*Mohoua novaeseelandiae*	Australasia	0	-0.0925	2.8016	2.49	0.6370	0.0364	35.57
Acanthizidae	*Mohoua ochrocephala*	Australasia	0	-0.1877	-	2.76	0.7286	0.2118	30.53
Acanthizidae	*Oreoscopus gutturalis*	Neotropics	0	-0.2363	-	3.20	0.7898	0.3841	33.53
Acanthizidae	*Origma solitaria*	Neotropics	1	-0.1821	4.5930	3.04	0.6631	0.4406	55.80
Acanthizidae	*Pycnoptilus floccosus*	Neotropics	1	-0.4414	2.2757	2.31	0.7327	0.2261	7.28
Acanthizidae	*Pyrrholaemus brunneus*	Neotropics	0	-0.2940	5.4681	2.10	0.7435	0.2510	84.59
Acanthizidae	*Sericornis arfakianus*	Neotropics	0	-0.2447	8.3958	3.29	0.6662	0.5542	68.85
Acanthizidae	*Sericornis beccarii*	Neotropics	0	-0.6113	1.2171	4.02	0.7544	0.5509	0.91
Acanthizidae	*Sericornis citreogularis*	Neotropics	0	2.9692	9.0699	3.95	0.7158	0.2834	87.01
Acanthizidae	*Sericornis frontalis*	Neotropics	0	-0.4141	-	3.49	0.8141	0.6858	53.67
Acanthizidae	*Sericornis humilis*	Neotropics	0	-0.1074	6.8870	3.50	0.7816	0.4264	66.00
Acanthizidae	*Sericornis keri*	Neotropics	0	-0.4417	-	3.19	0.7856	0.5207	75.84
Acanthizidae	*Sericornis magnirostra*	Australasia	1	-0.0512	16.5265	2.80	0.7493	0.1468	23.01
Acanthizidae	*Sericornis nouhuysi*	Neotropics	0	0.0318	-	2.99	0.7151	0.4116	80.20
Acanthizidae	*Sericornis papuensis*	Neotropics	0	-0.4246	13.5722	3.52	0.7590	0.2390	85.45
Acanthizidae	*Sericornis perspicillatus*	IndoMalay	1	-0.0533	6.9509	3.32	0.8248	0.4312	14.60
Acanthizidae	*Sericornis rufescens*	IndoMalay	0	-0.0031	-	2.64	0.7327	0.2318	67.52
Acanthizidae	*Sericornis spilodera*	Neotropics	0	-0.1899	-	3.76	0.8558	0.5154	40.77
Acanthizidae	*Sericornis virgatus*	Afrotropics	0	-0.1910	4.3061	3.60	0.8737	0.2658	9.59
Acanthizidae	*Smicrornis brevirostris*	IndoMalay	0	0.1487	15.1966	4.00	0.7480	0.2226	12.48
Accipitridae	*Accipiter albogularis*	Australasia	0	-0.3194	5.5820	2.48	0.6221	0.0384	35.15
Accipitridae	*Accipiter badius*	Afrotropics	0	-0.3480	-	3.59	0.8672	0.4462	21.81
Accipitridae	*Accipiter bicolor*	Neotropics	0	-0.3034	-	3.71	0.8560	0.7186	54.35
Accipitridae	*Accipiter brachyurus*	Australasia	1	-0.3886	5.7974	2.50	0.6716	0.2680	24.42
Accipitridae	*Accipiter brevipes*	Palearctic	0	-0.7661	-	4.37	0.7723	0.1690	12.43
Accipitridae	*Accipiter castanilius*	Afrotropics	0	-0.0652	-	3.30	0.8119	0.6749	18.00
Accipitridae	*Accipiter cirrocephalus*	Australasia	0	-0.4049	7.4739	3.20	0.8311	0.8983	1.58
Accipitridae	*Accipiter collaris*	Neotropics	0	-0.1542	6.2897	3.42	0.6921	0.1251	67.68
Accipitridae	*Accipiter cooperii*	Nearctic	0	-0.7736	11.5642	4.20	0.8414	0.4534	3.55

Family	Species	Realm	Threat	Latitude	Elevation	Anomaly	Size	Shape	Orientation
Accipitridae	*Accipiter erythrauchen*	Australasia	0	-0.2938	4.1372	2.48	0.6690	0.5741	71.12
Accipitridae	*Accipiter erythropus*	Afrotropics	0	0.0373	-	3.29	0.8215	0.2638	19.41
Accipitridae	*Accipiter fasciatus*	Australasia	0	-0.3846	6.9225	3.30	0.8400	0.7433	0.76
Accipitridae	*Accipiter francesiae*	Afrotropics	0	-0.0773	6.5428	3.16	0.7591	0.3890	70.06
Accipitridae	*Accipiter gentilis*	Palearctic	0	-0.8865	8.7011	4.55	0.8774	0.1020	1.21
Accipitridae	*Accipiter griseiceps*	Australasia	0	-0.0283	6.4203	2.71	0.7165	0.5145	57.40
Accipitridae	*Accipiter gularis*	Palearctic	0	-0.6451	5.4598	4.18	0.8310	0.5071	16.56
Accipitridae	*Accipiter gundlachi*	Neotropics	1	-0.3266	0.9520	2.53	0.6974	0.1632	17.43
Accipitridae	*Accipiter haplochrous*	Australasia	0	-0.1968	4.4757	2.58	0.6302	0.1134	34.50
Accipitridae	*Accipiter henicogrammus*	Australasia	0	0.1096	4.0173	2.48	0.6221	0.5774	71.57
Accipitridae	*Accipiter henstii*	Afrotropics	0	-0.0810	5.8799	3.16	0.7586	0.3900	69.95
Accipitridae	*Accipiter imitator*	Australasia	1	-0.1852	1.3738	2.48	0.5985	0.0156	34.57
Accipitridae	*Accipiter luteoschistaceus*	Australasia	1	-0.3482	2.5000	2.50	0.6716	0.2680	24.42
Accipitridae	*Accipiter madagascariensis*	Afrotropics	0	-0.0661	5.1472	3.17	0.7582	0.4160	70.64
Accipitridae	*Accipiter melanochlamys*	Australasia	0	-0.0823	9.8690	2.83	0.7363	0.1601	23.62
Accipitridae	*Accipiter melanoleucus*	Afrotropics	0	-0.0588	-	3.47	0.8445	0.8500	47.86
Accipitridae	*Accipiter meyerianus*	Australasia	0	-0.0592	9.2838	2.75	0.7503	0.1851	16.83
Accipitridae	*Accipiter minullus*	Afrotropics	0	-0.1008	-	3.61	0.8371	0.5108	62.25
Accipitridae	*Accipiter nanus*	Australasia	0	0.1791	7.3988	2.71	0.7019	0.4139	40.46
Accipitridae	*Accipiter nisus*	Palearctic	0	-0.8845	9.9815	4.64	0.8698	0.2201	2.65
Accipitridae	*Accipiter novaehollandiae*	Australasia	0	-0.3583	4.4771	3.05	0.8101	0.5258	67.28
Accipitridae	*Accipiter ovampensis*	Afrotropics	0	-0.0403	-	3.65	0.8406	0.6164	41.89
Accipitridae	*Accipiter poliocephalus*	Australasia	0	-0.0892	3.0919	2.78	0.7701	0.3106	24.22
Accipitridae	*Accipiter poliogaster*	Neotropics	0	-0.2007	1.5950	3.90	0.8383	0.6165	44.32
Accipitridae	*Accipiter princeps*	Australasia	1	-0.3995	3.8777	2.49	0.6631	0.3432	20.37
Accipitridae	*Accipiter rhodogaster*	Australasia	0	-0.0727	6.4527	2.71	0.7165	0.5479	58.43
Accipitridae	*Accipiter rufiventris*	Afrotropics	0	-0.1175	-	3.50	0.7848	0.2462	71.81
Accipitridae	*Accipiter soloensis*	IndoMalay	0	-0.6205	4.0127	3.28	0.7967	0.4875	17.34
Accipitridae	*Accipiter striatus*	Nearctic	0	-0.6333	13.8511	3.92	0.8612	0.3241	47.66
Accipitridae	*Accipiter superciliosus*	Neotropics	0	-0.1798	-	3.87	0.8428	0.5732	38.23
Accipitridae	*Accipiter tachiro*	Afrotropics	0	-0.1087	12.0957	3.44	0.8474	0.6550	33.59
Accipitridae	*Accipiter trinotatus*	Australasia	0	-0.0691	-	2.71	0.7172	0.5758	60.34
Accipitridae	*Accipiter trivirgatus*	IndoMalay	0	-0.0767	-	3.04	0.8216	0.5603	39.81
Accipitridae	*Accipiter virgatus*	IndoMalay	0	-0.1459	8.1538	3.27	0.8266	0.7019	16.91
Accipitridae	*Aegypius monachus*	Palearctic	0	-0.8572	17.2003	4.26	0.8552	0.2748	6.27
Accipitridae	*Aquila adalberti*	Palearctic	1	-0.1540	5.7564	3.87	0.7228	0.7275	38.25
Accipitridae	*Aquila africanus*	Afrotropics	0	-0.0113	0.0000	3.23	0.8033	0.2042	13.25
Accipitridae	*Aquila audax*	Australasia	0	-0.5186	7.2484	3.34	0.8380	0.5846	7.67
Accipitridae	*Aquila chrysaetos*	Palearctic	0	-0.8103	10.8284	4.51	0.8838	0.1300	0.12
Accipitridae	*Aquila clanga*	Palearctic	1	-0.7255	3.6189	4.93	0.8476	0.1670	4.07
Accipitridae	*Aquila fasciatus*	IndoMalay	0	-0.1734	9.2123	3.65	0.8346	0.1592	7.81
Accipitridae	*Aquila gurneyi*	Australasia	0	-0.5804	3.1447	2.76	0.7691	0.2647	21.87
Accipitridae	*Aquila hastata*	IndoMalay	1	-0.1932	2.9676	3.32	0.7908	0.7675	1.96
Accipitridae	*Aquila heliaca*	Palearctic	1	-0.9197	4.0781	4.62	0.8468	0.1737	3.50
Accipitridae	*Aquila nipalensis*	Palearctic	0	-0.9786	9.7799	4.49	0.8369	0.2151	3.11
Accipitridae	*Aquila pomarina*	Palearctic	0	-0.6875	5.1455	4.09	0.8110	0.5564	20.79
Accipitridae	*Aquila rapax*	Afrotropics	0	-0.1737	11.2367	3.65	0.8616	0.6247	16.07
Accipitridae	*Aquila verreauxii*	Afrotropics	0	-0.2479	22.7653	3.58	0.8252	0.3933	70.78
Accipitridae	*Aquila wahlbergi*	Afrotropics	0	-0.0894	-	3.61	0.8529	0.6437	36.09
Accipitridae	*Aviceda cuculoides*	Neotropics	0	0.0608	-	3.99	0.8354	0.6148	31.28
Accipitridae	*Aviceda jerdoni*	Neotropics	0	0.0538	5.2442	3.82	0.8098	0.5503	19.98
Accipitridae	*Aviceda leuphotes*	Neotropics	0	0.4541	4.1107	3.19	0.6221	0.5865	19.14
Accipitridae	*Aviceda madagascariensis*	Neotropics	0	0.0342	-	3.99	0.8231	0.6202	16.46
Accipitridae	*Aviceda subcristata*	Afrotropics	0	-0.1203	-	3.43	0.8482	0.4597	32.92
Accipitridae	*Busarellus nigricollis*	Australasia	0	-0.1076	4.2375	2.56	0.6523	0.1875	30.24
Accipitridae	*Butastur indicus*	Afrotropics	0	-0.0448	0.0000	3.37	0.7228	0.2696	43.62
Accipitridae	*Butastur liventer*	Afrotropics	0	-0.1421	-	3.59	0.8256	0.4834	33.25
Accipitridae	*Butastur rufipennis*	Afrotropics	0	-0.0966	-	3.59	0.8197	0.2694	63.78
Accipitridae	*Butastur teesa*	Neotropics	0	-0.0231	-	3.77	0.7811	0.3190	19.15
Accipitridae	*Buteo albicaudatus*	Afrotropics	0	-0.1908	-	3.63	0.8524	0.8212	57.21
Accipitridae	*Buteo albigula*	Australasia	0	-0.5078	-	3.34	0.8141	0.5811	6.94
Accipitridae	*Buteo albonotatus*	Palearctic	0	-0.5577	-	3.91	0.8574	0.3113	11.24
Accipitridae	*Buteo augur*	Afrotropics	0	-0.2250	-	3.51	0.8380	0.3015	0.70
Accipitridae	*Buteo auguralis*	Neotropics	0	-0.1950	-	3.51	0.7158	0.1406	57.11
Accipitridae	*Buteo brachypterus*	Afrotropics	0	-0.1600	-	3.49	0.8341	0.8517	28.65
Accipitridae	*Buteo brachyurus*	Neotropics	0	-0.1921	-	3.77	0.8529	0.5012	39.88
Accipitridae	*Buteo buteo*	Palearctic	0	-0.9376	5.8523	4.09	0.7982	0.8639	5.35
Accipitridae	*Buteo galapagoensis*	IndoMalay	0	-0.0876	4.1977	3.01	0.7915	0.3108	52.40
Accipitridae	*Buteo hemilasius*	Afrotropics	0	0.0498	-	3.49	0.8401	0.2356	9.80
Accipitridae	*Buteo hemilasius*	Afrotropics	0	0.0498	-	3.49	0.8401	-	-
Accipitridae	*Buteo jamaicensis*	IndoMalay	0	-0.1268	-	3.49	0.8206	0.5542	11.36
Accipitridae	*Buteo lagopus*	Neotropics	0	-0.2192	-	3.57	0.8457	0.5093	48.52
Accipitridae	*Buteo leucorrhous*	Neotropics	0	-0.6355	9.6086	2.73	0.7452	0.1943	85.10
Accipitridae	*Buteo lineatus*	Neotropics	0	-0.1874	-	3.74	0.8397	0.2917	36.77

Family	Species	Realm	Threat	Latitude	Elevation	Anomaly	Size	Shape	Orientation
Accipitridae	*Buteo magnirostris*	Afrotropics	0	-0.0320	23.3731	3.51	0.8230	0.4177	58.58
Accipitridae	*Buteo nitidus*	Afrotropics	0	0.1600	-	3.44	0.8372	0.4642	4.96
Accipitridae	*Buteo oreophilus*	Afrotropics	0	-0.0768	-	3.18	0.7614	0.3874	70.15
Accipitridae	*Buteo platypterus*	Neotropics	0	-0.1629	-	3.81	0.8556	0.3948	36.32
Accipitridae	*Buteo polyosoma*	Palearctic	0	-0.8498	6.3982	4.53	0.8637	0.1898	1.07
Accipitridae	*Buteo ridgwayi*	Palearctic	0	-1.2093	17.0261	4.35	0.8364	0.3588	18.07
Accipitridae	*Buteo rufinus*	Nearctic	0	-0.5644	-	4.12	0.8575	0.5005	21.45
Accipitridae	*Buteo rufofuscus*	Nearctic	0	-0.7286	-	5.32	0.8236	0.0996	0.65
Accipitridae	*Buteo socotraensis*	Neotropics	0	-0.2131	10.3670	3.36	0.7953	0.3688	41.92
Accipitridae	*Buteo solitarius*	Nearctic	0	-0.8820	-	4.08	0.8194	0.4483	13.65
Accipitridae	*Buteo solitarius*	Nearctic	0	-0.8820	-	4.08	0.8194	-	-
Accipitridae	*Buteo swainsoni*	Neotropics	0	-0.2227	-	3.71	0.8568	0.5462	44.82
Accipitridae	*Buteo ventralis*	Neotropics	0	-0.1289	3.7781	3.87	0.8499	0.3572	35.19
Accipitridae	*Buteogallus aequinoctialis*	Afrotropics	0	-0.0762	21.1244	3.34	0.7903	0.2047	74.39
Accipitridae	*Buteogallus anthracinus*	Nearctic	0	-0.7240	-	4.24	0.8280	0.5966	18.72
Accipitridae	*Buteogallus gundlachii*	Neotropics	0	-0.3060	18.5075	3.13	0.8152	0.2355	81.94
Accipitridae	*Buteogallus meridionalis*	Nearctic	0	-0.8870	9.6742	4.36	0.8120	0.9246	4.86
Accipitridae	*Buteogallus urubitinga*	Neotropics	1	0.1826	5.4316	2.47	0.6842	0.3429	7.79
Accipitridae	*Chelictinia riocourii*	Australasia	1	-0.2228	-	2.58	0.6302	0.1134	34.50
Accipitridae	*Chondrohierax uncinatus*	Neotropics	0	-0.0911	5.7666	3.44	0.7674	0.4332	34.92
Accipitridae	*Circaetus beaudouini*	Australasia	0	-0.8111	-	3.21	0.8123	0.4062	7.96
Accipitridae	*Circaetus cinerascens*	Australasia	0	-0.6750	-	3.27	0.7786	0.7679	13.52
Accipitridae	*Circaetus cinereus*	Australasia	0	-0.5986	-	3.06	0.7664	0.3814	68.40
Accipitridae	*Circaetus fasciolatus*	Palearctic	0	-0.6207	-	4.20	0.8482	0.2626	5.31
Accipitridae	*Circaetus gallicus*	Neotropics	0	0.0478	15.2251	3.70	0.7791	0.3937	80.50
Accipitridae	*Circaetus pectoralis*	Nearctic	0	-0.6289	11.8371	3.92	0.8296	0.2545	38.35
Accipitridae	*Circus aeruginosus*	Palearctic	0	-0.8140	-	3.91	0.8458	0.3666	19.28
Accipitridae	*Circus approximans*	Neotropics	1	10.6861	5.9296	3.71	0.7223	0.2784	80.39
Accipitridae	*Circus assimilis*	Neotropics	0	-9.4819	10.2701	4.08	0.7228	0.1637	32.84
Accipitridae	*Circus buffoni*	Neotropics	0	-0.0036	8.9469	3.27	0.7348	0.2146	68.09
Accipitridae	*Circus cinereus*	Neotropics	0	-10.9337	10.0625	3.88	0.7151	0.1490	59.15
Accipitridae	*Circus cyaneus*	Neotropics	0	0.0281	9.3259	3.36	0.7399	0.1740	62.35
Accipitridae	*Circus macrosceles*	Afrotropics	1	-5.6599	4.8212	3.19	0.6562	0.2996	88.58
Accipitridae	*Circus macrourus*	Afrotropics	0	-0.1012	10.2984	3.63	0.8474	0.7038	40.53
Accipitridae	*Circus maurus*	Afrotropics	0	-0.0306	4.2739	3.32	0.7591	0.7238	59.20
Accipitridae	*Circus melanoleucos*	Afrotropics	1	0.0388	8.4716	3.58	0.8072	0.1455	3.08
Accipitridae	*Circus pygargus*	Afrotropics	0	-0.0854	-	3.60	0.8556	0.6867	34.19
Accipitridae	*Circus ranivorus*	Afrotropics	0	-0.0655	-	3.62	0.8510	0.6994	41.43
Accipitridae	*Circus spilonotus*	Afrotropics	0	-0.2203	4.0720	3.22	0.7748	0.2046	74.48
Accipitridae	*Dryotriorchis spectabilis*	Neotropics	0	0.0587	8.7490	3.62	0.7626	-	-
Accipitridae	*Dryotriorchis spectabilis*	Neotropics	0	0.0587	8.7490	3.62	0.7626	0.4607	84.63
Accipitridae	*Elanoides forficatus*	Australasia	0	-0.3916	-	3.02	0.8106	0.6164	19.91
Accipitridae	*Elanus axillaris*	Neotropics	0	-0.3657	0.3225	3.20	0.7647	0.3461	12.04
Accipitridae	*Elanus caeruleus*	Australasia	0	-0.4038	-	2.88	0.8273	0.5896	50.35
Accipitridae	*Elanus leucurus*	Neotropics	0	-0.3333	-	3.83	0.8641	0.3112	46.61
Accipitridae	*Elanus scriptus*	Neotropics	0	-0.4053	-	3.41	0.7957	0.4818	30.01
Accipitridae	*Eutriorchis astur*	Australasia	1	0.0611	7.0738	2.63	0.6716	0.3543	24.58
Accipitridae	*Gampsonyx swainsonii*	Afrotropics	1	-0.1198	10.9066	3.10	0.7335	0.1812	73.43
Accipitridae	*Geranoaetus melanoleucus*	Neotropics	0	-0.1501	11.1054	3.79	0.7314	0.2414	64.24
Accipitridae	*Geranospiza caerulescens*	Neotropics	0	-0.0488	-	3.40	0.7008	0.0678	61.03
Accipitridae	*Gypaetus barbatus*	Palearctic	1	-1.6551	-	6.29	0.7637	0.0244	3.09
Accipitridae	*Gypohierax angolensis*	Palearctic	1	-0.5624	-	4.69	0.7973	0.2655	11.19
Accipitridae	*Gyps africanus*	Palearctic	1	6.0770	13.4912	4.63	0.7800	0.3277	14.41
Accipitridae	*Gyps bengalensis*	Afrotropics	1	-0.5132	10.1970	3.67	0.7783	0.9535	1.77
Accipitridae	*Gyps coprotheres*	Australasia	0	-0.3783	0.0000	3.39	0.8184	0.5699	39.39
Accipitridae	*Gyps fulvus*	Palearctic	1	-0.8547	-	4.39	0.7769	0.2506	1.48
Accipitridae	*Gyps himalayensis*	Palearctic	0	-0.9188	9.4450	4.51	0.8449	0.1855	0.93
Accipitridae	*Gyps indicus*	Australasia	0	-1.3290	-	2.46	0.5788	0.0000	0.00
Accipitridae	*Gyps rueppellii*	Neotropics	1	-0.0685	-	4.06	0.7852	0.2564	10.67
Accipitridae	*Gyps tenuirostris*	Neotropics	1	-0.5445	1.7621	2.76	0.7914	0.7220	39.61
Accipitridae	*Haliaeetus albicilla*	Afrotropics	1	-0.5937	14.6699	3.72	0.7790	0.6030	59.26
Accipitridae	*Haliaeetus leucocephalus*	Palearctic	0	-0.6855	11.8233	4.11	0.8347	0.2752	2.23
Accipitridae	*Haliaeetus leucogaster*	Palearctic	0	-0.7663	16.5084	4.27	0.8171	0.4695	13.51
Accipitridae	*Haliaeetus leucoryphus*	IndoMalay	1	-0.0884	7.4424	3.49	0.7910	0.6708	74.53
Accipitridae	*Haliaeetus pelagicus*	Afrotropics	0	-0.0495	17.1245	3.62	0.8417	0.2777	11.91
Accipitridae	*Haliaeetus sanfordi*	IndoMalay	1	-0.1296	4.7182	3.21	0.7988	0.2470	32.32
Accipitridae	*Haliaeetus vocifer*	IndoMalay	0	-0.2789	7.3518	3.08	0.7416	0.4613	33.36
Accipitridae	*Haliaeetus vociferoides*	Neotropics	1	0.0404	0.0000	2.98	0.5788	0.0000	45.57
Accipitridae	*Haliastur indus*	Neotropics	0	0.4644	5.8873	2.99	0.6690	0.1618	80.83
Accipitridae	*Haliastur sphenurus*	Neotropics	0	0.0478	2.4469	3.37	0.7712	0.2940	30.78
Accipitridae	*Hamirostra melanosternon*	Neotropics	0	-0.2104	3.4161	3.51	0.7120	0.8803	83.90
Accipitridae	*Harpagus bidentatus*	Australasia	0	-0.3090	2.1279	2.94	0.8315	0.3689	40.53
Accipitridae	*Harpagus diodon*	Palearctic	1	-0.7489	14.8101	4.17	0.8504	0.5634	1.18
Accipitridae	*Harpia harpyja*	Palearctic	1	-0.6756	0.2493	4.56	0.7915	0.2834	12.24

Family	Species	Realm	Threat	Latitude	Elevation	Anomaly	Size	Shape	Orientation
Accipitridae	*Harpyhaliaetus coronatus*	Australasia	1	-0.2994	4.7039	2.48	0.6221	0.0384	35.15
Accipitridae	*Harpyhaliaetus solitarius*	Afrotropics	0	-0.1732	-	3.57	0.8619	0.6925	46.27
Accipitridae	*Harpyopsis novaeguineae*	Afrotropics	1	-0.1578	2.9234	3.11	0.7087	0.2680	68.94
Accipitridae	*Helicolestes hamatus*	Neotropics	0	-0.0213	9.0333	3.60	0.7539	0.2991	74.82
Accipitridae	*Henicopernis infuscatus*	Neotropics	0	-78.1727	3.1423	4.16	0.6631	0.7161	32.78
Accipitridae	*Henicopernis longicauda*	Neotropics	0	0.0233	7.6821	4.08	0.6662	0.2905	42.51
Accipitridae	*Hieraaetus ayresii*	Neotropics	0	0.3449	2.1948	3.63	0.7406	0.5754	22.34
Accipitridae	*Hieraaetus morphnoides*	Neotropics	0	7.4571	1.1170	3.76	0.6716	0.3510	0.46
Accipitridae	*Hieraaetus pennatus*	Neotropics	0	-0.2780	5.0435	4.00	0.8031	0.7769	6.04
Accipitridae	*Hieraaetus spilogaster*	Neotropics	0	-28.7357	6.4308	4.10	0.7104	0.4274	46.89
Accipitridae	*Hieraaetus weiskei*	Neotropics	1	0.0000	0.6389	3.77	0.6221	0.3820	31.72
Accipitridae	*Ichthyophaga humilis*	Neotropics	0	0.1350	3.5698	4.05	0.8320	0.6933	8.89
Accipitridae	*Ichthyophaga ichthyaetus*	Neotropics	0	0.0117	1.9081	4.14	0.8125	0.7460	2.86
Accipitridae	*Ictinaetus malayensis*	IndoMalay	0	-0.1095	-	3.19	0.7403	0.3871	79.84
Accipitridae	*Ictinia mississippiensis*	IndoMalay	0	-0.0655	-	2.75	0.7340	0.1598	40.52
Accipitridae	*Ictinia plumbea*	Palearctic	0	-0.6893	17.0779	4.25	0.8286	0.3175	1.49
Accipitridae	*Kaupifalco monogrammicus*	Neotropics	0	0.0126	6.3448	3.63	0.7399	0.2887	73.88
Accipitridae	*Leptodon cayanensis*	Afrotropics	0	-0.1130	-	3.52	0.7277	0.7689	50.18
Accipitridae	*Leptodon forbesi*	Palearctic	0	-1.1339	-	4.77	0.8271	0.2692	2.23
Accipitridae	*Leucopternis albicollis*	Neotropics	0	-0.3188	-	3.53	0.8354	0.4694	48.39
Accipitridae	*Leucopternis kuhli*	Neotropics	0	-0.4454	5.7452	2.72	0.7578	0.6887	36.07
Accipitridae	*Leucopternis lacernulatus*	Neotropics	0	-0.0263	7.2805	3.63	0.7706	0.4598	84.32
Accipitridae	*Leucopternis melanops*	Neotropics	0	-0.1368	14.7603	3.81	0.7552	0.2242	44.75
Accipitridae	*Leucopternis occidentalis*	Neotropics	0	-0.0281	-	3.66	0.7980	0.3936	17.87
Accipitridae	*Leucopternis plumbeus*	Neotropics	0	-0.2858	7.8573	3.44	0.7746	0.5010	52.63
Accipitridae	*Leucopternis polionotus*	Neotropics	0	0.0619	-	3.00	0.7078	0.3294	13.92
Accipitridae	*Leucopternis princeps*	Neotropics	0	-0.1439	2.1845	3.45	0.7461	0.4784	63.96
Accipitridae	*Leucopternis schistaceus*	Neotropics	1	0.1528	0.6295	2.99	0.6221	0.2856	23.69
Accipitridae	*Leucopternis semiplumbeus*	Neotropics	0	-13.3011	5.9606	4.08	0.7305	0.2728	37.16
Accipitridae	*Lophaetus occipitalis*	Afrotropics	0	-0.0177	7.6931	3.31	0.7586	0.3041	24.31
Accipitridae	*Lophoictinia isura*	Palearctic	1	30.4523	8.5072	3.31	0.6562	0.7017	89.34
Accipitridae	*Lophotriorchis kienerii*	Afrotropics	0	-0.0084	6.3434	3.29	0.8225	0.3361	12.10
Accipitridae	*Macheiramphus alcinus*	Australasia	0	0.1030	-	2.48	0.6221	0.5774	71.57
Accipitridae	*Megatriorchis doriae*	IndoMalay	0	-0.1319	-	3.33	0.8056	0.3489	20.27
Accipitridae	*Melierax canorus*	Afrotropics	0	0.0931	6.8375	3.44	0.8246	0.1772	6.73
Accipitridae	*Melierax gabar*	Afrotropics	0	-0.1457	-	3.70	0.8241	0.8904	87.85
Accipitridae	*Melierax metabates*	Australasia	0	-0.0330	-	2.69	0.6824	0.2455	24.56
Accipitridae	*Melierax poliopterus*	Australasia	0	-0.1834	12.7102	2.83	0.7281	0.1885	24.43
Accipitridae	*Milvus migrans*	IndoMalay	0	-0.0744	-	3.17	0.8241	0.2442	26.40
Accipitridae	*Milvus milvus*	Afrotropics	0	-0.0679	-	3.52	0.8563	-	-
Accipitridae	*Milvus milvus*	Afrotropics	0	-0.0679	-	3.52	0.8563	0.6414	29.60
Accipitridae	*Morphnus guianensis*	Afrotropics	0	-0.0514	-	3.55	0.8478	0.5177	35.81
Accipitridae	*Necrosyrtes monachus*	Neotropics	0	0.1240	1.1602	3.89	0.7040	0.5498	50.32
Accipitridae	*Neophron percnopterus*	Afrotropics	0	0.1106	0.0000	3.18	0.7438	0.4642	74.71
Accipitridae	*Nisaetus alboniger*	Australasia	0	-0.6165	-	2.42	0.7240	0.1672	3.23
Accipitridae	*Nisaetus bartelsi*	Australasia	0	-0.5646	-	3.20	0.7632	0.3950	55.56
Accipitridae	*Nisaetus cirrhatus*	Australasia	0	-0.7509	-	2.98	0.7535	0.3068	15.39
Accipitridae	*Nisaetus floris*	Palearctic	1	-0.3700	14.6767	3.92	0.8670	0.3957	8.37
Accipitridae	*Nisaetus lanceolatus*	Neotropics	0	0.0273	2.3865	4.02	0.8172	0.4905	22.56
Accipitridae	*Nisaetus nanus*	Australasia	0	-0.8254	-	3.48	0.8001	0.2348	5.11
Accipitridae	*Nisaetus nipalensis*	Australasia	0	-0.0682	6.4847	2.81	0.7449	0.1466	24.43
Accipitridae	*Nisaetus philippensis*	Australasia	0	-0.2874	12.3630	2.83	0.7363	0.1734	23.55
Accipitridae	*Pandion haliaetus*	Afrotropics	0	0.0577	4.3738	3.23	0.7766	0.1434	13.32
Accipitridae	*Parabuteo unicinctus*	Australasia	0	0.0130	-	2.71	0.7185	0.5623	60.71
Accipitridae	*Pernis apivorus*	Neotropics	0	-0.1719	2.2858	2.54	0.7296	0.4533	7.68
Accipitridae	*Pernis celebensis*	Neotropics	0	-0.3564	10.2969	3.15	0.8080	0.5405	70.03
Accipitridae	*Pernis ptilorhyncus*	Neotropics	0	-0.0665	3.4537	3.39	0.7556	0.2224	31.93
Accipitridae	*Pithecophaga jefferyi*	Neotropics	0	-0.1125	3.9347	3.90	0.7923	0.3201	35.60
Accipitridae	*Polemaetus bellicosus*	Afrotropics	0	0.0592	8.7414	3.33	0.7482	0.2660	14.03
Accipitridae	*Polyboroides radiatus*	Afrotropics	0	-0.0526	6.8575	3.33	0.7988	0.3689	12.55
Accipitridae	*Polyboroides typus*	Afrotropics	0	4.3408	-	3.50	0.7552	0.7205	88.13
Accipitridae	*Rostrhamus sociabilis*	Neotropics	0	-0.0002	2.4088	3.43	0.7406	0.3019	35.53
Accipitridae	*Sagittarius serpentarius*	Australasia	0	-0.3974	-	3.27	0.7810	0.3156	8.58
Accipitridae	*Sarcogyps calvus*	Neotropics	0	-0.3750	4.0231	3.41	0.8346	0.5126	45.39
Accipitridae	*Spilornis cheela*	Australasia	0	-0.5387	4.1548	3.08	0.7466	0.3804	85.58
Accipitridae	*Spilornis holospilus*	Australasia	0	-0.0801	13.6827	2.84	0.7323	0.1522	22.76
Accipitridae	*Spilornis kinabaluensis*	Australasia	0	-0.2836	8.7790	2.84	0.7256	0.1680	21.15
Accipitridae	*Spilornis rufipectus*	Australasia	0	-0.2901	-	2.83	0.7385	0.1836	23.17
Accipitridae	*Spizaetus isidori*	Australasia	0	-0.0440	1.2400	2.65	0.5985	0.5774	45.00
Accipitridae	*Spizaetus melanoleucus*	Australasia	0	-0.4925	-	3.11	0.7128	0.2784	75.67
Accipitridae	*Spizaetus ornatus*	IndoMalay	0	-0.0493	7.9922	3.08	0.7942	0.4582	81.73
Accipitridae	*Spizaetus tyrannus*	Afrotropics	0	-0.4439	-	3.67	0.7725	0.5246	40.65
Accipitridae	*Stephanoaetus coronatus*	Palearctic	0	-0.2041	-	3.45	0.7864	0.8902	79.36
Accipitridae	*Terathopius ecaudatus*	Neotropics	0	0.3510	5.0615	4.03	0.7507	0.2056	13.00

Family	Species	Realm	Threat	Latitude	Elevation	Anomaly	Size	Shape	Orientation
Accipitridae	*Torgos tracheliotos*	Neotropics	0	-0.1626	1.7351	4.20	0.7923	0.4478	7.30
Accipitridae	*Trigonoceps occipitalis*	Neotropics	0	-0.2068	-	3.22	0.6784	0.6109	56.61
Accipitridae	*Urotriorchis macrourus*	Afrotropics	0	-0.0009	-	3.26	0.7874	0.3831	57.48
Aegithalidae	*Aegithalos caudatus*	Palearctic	0	-0.4938	-	4.40	0.8640	0.1918	1.15
Aegithalidae	*Aegithalos concinnus*	IndoMalay	0	-0.3951	-	3.35	0.8168	0.4568	1.90
Aegithalidae	*Aegithalos fuliginosus*	Palearctic	0	-0.9136	8.1083	3.53	0.7422	0.5369	25.41
Aegithalidae	*Aegithalos iouschistos*	Palearctic	0	-0.3803	-	3.86	0.7552	0.2423	6.14
Aegithalidae	*Aegithalos leucogenys*	Palearctic	0	-12.7891	11.9962	4.42	0.7503	0.5047	8.48
Aegithalidae	*Aegithalos niveogularis*	IndoMalay	0	-3.2078	9.5860	4.34	0.7389	0.1549	32.97
Aegithalidae	*Leptopoecile elegans*	Neotropics	0	-0.5969	-	2.37	0.8040	0.4386	53.61
Aegithalidae	*Leptopoecile sophiae*	Palearctic	0	-0.5837	-	3.79	0.7636	0.3363	11.43
Aegithalidae	*Psaltria exilis*	Neotropics	0	-0.2297	3.6045	3.21	0.8077	0.3810	57.70
Aegithalidae	*Psaltriparus minimus*	Australasia	0	-0.6904	-	3.15	0.8060	0.4865	15.85
Aegithinidae	*Aegithina lafresnayei*	IndoMalay	0	-0.1001	-	3.01	0.7950	0.7033	73.61
Aegithinidae	*Aegithina nigrolutea*	IndoMalay	0	-0.1216	-	3.47	0.7732	0.9116	51.80
Aegithinidae	*Aegithina tiphia*	IndoMalay	0	-0.0707	-	3.15	0.8335	0.4410	31.84
Aegithinidae	*Aegithina viridissima*	IndoMalay	0	-0.0036	-	2.84	0.7876	0.4871	7.93
Aegothelidae	*Aegotheles albertisi*	Australasia	0	-0.0810	14.4721	2.81	0.7422	0.1501	24.23
Aegothelidae	*Aegotheles archboldi*	Australasia	0	-0.7169	8.9985	2.89	0.7112	0.1400	15.46
Aegothelidae	*Aegotheles bennettii*	Australasia	0	-0.0676	-	2.78	0.7382	0.3595	35.46
Aegothelidae	*Aegotheles crinifrons*	Australasia	0	0.1966	4.8291	2.48	0.6221	0.5774	71.57
Aegothelidae	*Aegotheles cristatus*	Australasia	0	-0.4840	3.6335	3.33	0.8381	0.5986	6.78
Aegothelidae	*Aegotheles insignis*	Australasia	0	-0.0484	10.1316	2.81	0.7425	0.1527	24.19
Aegothelidae	*Aegotheles savesi*	Australasia	1	-0.0892	2.8169	2.58	0.6302	0.1134	34.50
Aegothelidae	*Aegotheles tatei*	Australasia	0	-0.4991	-	2.94	0.6120	1.0000	0.00
Aegothelidae	*Aegotheles wallacii*	Australasia	0	-0.0829	4.5936	2.83	0.7087	0.2617	22.33
Alaudidae	*Alaemon alaudipes*	Nearctic	0	-0.7506	-	4.16	0.8358	0.4403	5.77
Alaudidae	*Alaemon hamertoni*	Palearctic	0	-0.2593	-	4.03	0.8474	0.2395	2.93
Alaudidae	*Alauda arvensis*	Afrotropics	0	0.1040	-	3.33	0.7310	0.6899	58.21
Alaudidae	*Alauda gulgula*	Palearctic	0	-0.6571	9.0428	4.42	0.8779	0.3946	2.48
Alaudidae	*Alauda japonica*	IndoMalay	0	-0.4826	-	3.67	0.8513	0.4325	7.56
Alaudidae	*Ammomanes cinctura*	Palearctic	0	-0.4655	7.0635	4.08	0.8385	0.2236	2.68
Alaudidae	*Ammomanes deserti*	Palearctic	0	-0.3535	10.9615	4.03	0.8502	0.2768	4.03
Alaudidae	*Ammomanes grayi*	Afrotropics	0	-0.3511	-	3.48	0.7310	0.1996	68.02
Alaudidae	*Ammomanes phoenicura*	IndoMalay	0	-0.1413	-	3.46	0.7987	0.7874	69.92
Alaudidae	*Calandrella acutirostris*	Australasia	0	0.0234	3.0885	2.48	0.6221	0.5774	71.57
Alaudidae	*Calandrella brachydactyla*	IndoMalay	0	-0.1285	-	3.03	0.8273	0.5681	54.33
Alaudidae	*Calandrella cheleensis*	IndoMalay	0	-0.1482	-	3.41	0.8030	0.6860	74.55
Alaudidae	*Calandrella cinerea*	IndoMalay	0	-0.0510	-	2.77	0.7923	0.6518	14.18
Alaudidae	*Calandrella raytal*	IndoMalay	0	-0.0676	-	3.13	0.8317	0.4560	33.99
Alaudidae	*Calandrella rufescens*	Australasia	0	-0.3365	6.2849	3.04	0.8067	0.6106	59.43
Alaudidae	*Calandrella somalica*	Neotropics	0	-0.1882	-	3.76	0.8556	0.4604	40.67
Alaudidae	*Certhilauda albescens*	Afrotropics	0	-0.4686	-	3.71	0.7745	0.5584	8.82
Alaudidae	*Certhilauda barlowi*	Afrotropics	0	0.0602	13.8739	3.49	0.7485	0.2424	79.72
Alaudidae	*Certhilauda burra*	Afrotropics	0	-0.3284	-	3.81	0.7824	0.3107	55.93
Alaudidae	*Certhilauda chuana*	Afrotropics	0	-0.2263	6.3622	3.84	0.8227	0.1550	1.92
Alaudidae	*Certhilauda curvirostris*	Australasia	0	-0.5198	0.0000	2.59	0.7677	0.1924	10.44
Alaudidae	*Certhilauda erythrochlamys*	Nearctic	0	-0.3917	0.5542	3.12	0.7223	0.0531	0.68
Alaudidae	*Certhilauda semitorquata*	Nearctic	0	-0.6752	12.7529	4.07	0.8390	-	-
Alaudidae	*Certhilauda semitorquata*	Nearctic	0	-0.6752	12.7529	4.07	0.8390	0.4979	5.57
Alaudidae	*Certhilauda subcoronata*	Palearctic	0	-0.6525	-	3.65	0.8193	0.4405	4.77
Alaudidae	*Chersomanes albofasciata*	Australasia	0	-0.0571	-	2.75	0.7751	0.2904	17.46
Alaudidae	*Chersophilus duponti*	Australasia	0	-0.0450	6.4616	2.79	0.7582	0.1914	24.12
Alaudidae	*Eremalauda dunni*	Australasia	0	-0.7227	-	2.81	0.7657	0.2376	5.16
Alaudidae	*Eremalauda starki*	Australasia	0	-0.2118	-	2.96	0.7667	0.7166	4.86
Alaudidae	*Eremophila alpestris*	Neotropics	0	0.2853	2.5881	3.88	0.7973	0.8719	77.06
Alaudidae	*Eremophila bilopha*	Neotropics	0	9.2122	0.8934	3.68	0.7136	0.5791	61.12
Alaudidae	*Eremopterix australis*	Neotropics	0	0.1095	2.7834	4.07	0.8101	0.8881	67.32
Alaudidae	*Eremopterix griseus*	Neotropics	0	0.2270	2.4399	4.16	0.8031	0.3730	20.22
Alaudidae	*Eremopterix leucopareia*	Neotropics	0	0.2018	4.6204	4.09	0.8059	0.4883	2.35
Alaudidae	*Eremopterix leucotis*	Neotropics	0	-1.5343	5.6964	3.74	0.7256	0.2279	69.45
Alaudidae	*Eremopterix nigriceps*	Australasia	0	-0.7726	-	3.06	0.8105	0.2507	8.87
Alaudidae	*Eremopterix signatus*	Australasia	0	-0.6311	-	3.41	0.8304	0.4791	5.55
Alaudidae	*Eremopterix verticalis*	Australasia	0	-0.4537	-	3.54	0.8052	0.3887	23.85
Alaudidae	*Galerida cristata*	Palearctic	0	-0.7845	-	5.19	0.8551	0.1380	0.45
Alaudidae	*Galerida magnirostris*	Nearctic	0	-0.7824	-	4.14	0.8484	0.6833	32.71
Alaudidae	*Galerida malabarica*	Neotropics	0	0.0069	15.3654	4.06	0.7688	0.2677	54.88
Alaudidae	*Galerida modesta*	Neotropics	0	-0.5846	-	2.46	0.8100	0.3934	49.02
Alaudidae	*Galerida theklae*	Palearctic	0	-0.5441	-	4.29	0.8732	0.3018	1.36
Alaudidae	*Heteromirafra archeri*	Neotropics	0	19.4092	3.9112	4.07	0.7185	0.1251	27.36
Alaudidae	*Heteromirafra ruddi*	Neotropics	0	-8.6494	4.7010	4.03	0.7087	0.2532	39.38
Alaudidae	*Heteromirafra sidamoensis*	Neotropics	0	0.1029	-	4.10	0.8198	0.6051	2.62
Alaudidae	*Lullula arborea*	Neotropics	0	-0.1152	3.3612	3.98	0.8152	0.5656	43.69
Alaudidae	*Melanocorypha calandra*	Australasia	0	-0.2118	3.4253	2.89	0.7759	0.6628	18.46

Family	Species	Realm	Threat	Latitude	Elevation	Anomaly	Size	Shape	Orientation
Alaudidae	*Melanocorypha maxima*	Neotropics	0	-0.1532	5.3930	3.81	0.8538	0.4412	36.47
Alaudidae	*Melanocorypha mongolica*	Neotropics	0	0.0636	5.9380	3.65	0.7591	0.4220	83.79
Alaudidae	*Melanocorypha yeltoniensis*	Nearctic	0	-0.7372	4.6205	4.22	0.8277	0.6637	1.11
Alaudidae	*Mirafra affinis*	Neotropics	0	3.4001	7.7092	3.19	0.7104	0.1263	65.28
Alaudidae	*Mirafra africana*	IndoMalay	0	-0.0418	-	3.21	0.8141	0.4820	30.33
Alaudidae	*Mirafra africanoides*	Neotropics	0	0.1085	3.4996	4.03	0.7931	0.6877	41.15
Alaudidae	*Mirafra albicauda*	Neotropics	0	1.1024	13.2011	3.99	0.7797	0.4266	74.27
Alaudidae	*Mirafra alopex*	Neotropics	0	0.0938	14.3570	3.97	0.7664	0.1975	42.84
Alaudidae	*Mirafra alopex*	Neotropics	0	0.0938	14.3570	3.97	0.7664	-	-
Alaudidae	*Mirafra angolensis*	Neotropics	0	-0.2851	13.6358	3.63	0.7968	0.2881	84.85
Alaudidae	*Mirafra apiata*	Neotropics	0	16.8570	8.1596	3.92	0.7158	0.2912	70.67
Alaudidae	*Mirafra apiata*	Neotropics	0	16.8570	8.1596	3.92	0.7158	-	-
Alaudidae	*Mirafra ashi*	Neotropics	0	0.2008	-	3.87	0.7693	0.4537	66.55
Alaudidae	*Mirafra assamica*	Neotropics	0	0.0254	-	4.03	0.8233	0.6020	33.03
Alaudidae	*Mirafra cantillans*	Neotropics	0	-0.1078	1.9298	4.09	0.7998	0.4837	32.37
Alaudidae	*Mirafra cheniana*	Neotropics	0	-0.0589	-	3.99	0.8299	0.7001	1.06
Alaudidae	*Mirafra collaris*	Neotropics	1	0.2926	5.1485	3.00	0.7144	0.1906	67.37
Alaudidae	*Mirafra cordofanica*	Neotropics	0	-0.1905	-	3.82	0.8525	0.4894	35.58
Alaudidae	*Mirafra erythrocephala*	Neotropics	0	-0.1620	-	3.82	0.8537	0.4583	35.99
Alaudidae	*Mirafra erythroptera*	Nearctic	0	-0.4476	-	4.01	0.7735	0.5628	29.07
Alaudidae	*Mirafra gilletti*	Neotropics	0	0.0401	-	3.49	0.7737	0.3431	73.13
Alaudidae	*Mirafra hova*	Neotropics	0	0.1433	-	3.95	0.8071	0.3126	10.11
Alaudidae	*Mirafra hypermetra*	Neotropics	0	0.1261	-	4.07	0.7965	0.5043	3.49
Alaudidae	*Mirafra javanica*	Neotropics	0	0.0818	-	3.95	0.8278	0.6053	24.09
Alaudidae	*Mirafra microptera*	Neotropics	0	0.0422	-	3.47	0.7359	0.3084	30.76
Alaudidae	*Mirafra passerina*	Neotropics	0	0.2727	6.7247	4.24	0.7493	0.7678	27.03
Alaudidae	*Mirafra poecilosterna*	Neotropics	0	0.1009	6.0376	3.19	0.7096	0.3636	65.82
Alaudidae	*Mirafra pulpa*	Australasia	0	-0.0812	-	2.78	0.7697	0.3011	24.23
Alaudidae	*Mirafra rufa*	Australasia	0	-0.5682	-	3.27	0.8298	0.6513	4.76
Alaudidae	*Mirafra rufocinnamomea*	Australasia	0	-0.2160	3.6299	3.10	0.7779	0.6008	4.62
Alaudidae	*Mirafra sabota*	Australasia	0	-0.1036	-	2.78	0.7712	0.3092	23.61
Alaudidae	*Mirafra somalica*	Australasia	0	-0.0802	-	2.80	0.7539	0.4324	33.17
Alaudidae	*Pinarocorys erythropygia*	Palearctic	0	2.3227	6.5393	4.47	0.7498	0.2746	31.05
Alaudidae	*Pinarocorys nigricans*	IndoMalay	0	-0.3151	8.8110	3.09	0.7842	0.7054	31.96
Alaudidae	*Pseudalaemon fremantlii*	IndoMalay	0	-0.2512	-	3.62	0.8023	0.6056	32.52
Alaudidae	*Rhamphocoris clotbey*	IndoMalay	0	-0.0250	-	2.83	0.7928	0.6299	16.56
Alaudidae	*Spizocorys conirostris*	Afrotropics	0	0.0510	-	3.32	0.7863	0.3981	57.20
Alaudidae	*Spizocorys fringillaris*	IndoMalay	0	0.0697	9.6416	2.71	0.7179	0.4587	13.44
Alaudidae	*Spizocorys obbiensis*	Afrotropics	1	0.0000	0.6469	3.72	0.6120	1.0000	0.00
Alaudidae	*Spizocorys personata*	Afrotropics	0	-0.2607	-	4.01	0.8030	0.6952	55.35
Alaudidae	*Spizocorys sclateri*	Afrotropics	0	0.0055	0.0000	3.49	0.7687	0.3835	50.98
Alcedinidae	*Actenoides bougainvillei*	Australasia	1	-0.1242	4.8583	2.48	0.6120	0.0400	36.10
Alcedinidae	*Actenoides concretus*	IndoMalay	0	0.0157	-	2.84	0.7879	0.4967	9.99
Alcedinidae	*Actenoides hombroni*	IndoMalay	1	-0.0903	8.8137	2.63	0.6961	0.8096	10.51
Alcedinidae	*Actenoides lindsayi*	IndoMalay	0	-0.0689	2.8357	2.65	0.7087	0.2460	75.53
Alcedinidae	*Actenoides monachus*	Australasia	0	-0.0197	-	2.71	0.7158	0.5270	59.56
Alcedinidae	*Actenoides princeps*	Australasia	0	-0.0540	6.0797	2.71	0.7158	0.5198	57.16
Alcedinidae	*Alcedo argentata*	Palearctic	0	-0.9248	2.8945	3.35	0.7461	0.2858	48.07
Alcedinidae	*Alcedo atthis*	IndoMalay	1	-0.0400	2.7879	2.62	0.7019	0.6235	86.79
Alcedinidae	*Alcedo azurea*	Palearctic	0	-0.4695	-	3.90	0.8708	0.3288	7.73
Alcedinidae	*Alcedo coerulescens*	Australasia	0	-0.3606	4.4763	3.14	0.8173	0.5238	64.52
Alcedinidae	*Alcedo cristata*	IndoMalay	0	-0.1571	-	2.76	0.7331	0.1899	21.10
Alcedinidae	*Alcedo cyanopectus*	Afrotropics	0	-0.0903	0.0000	3.51	0.8564	0.6449	41.24
Alcedinidae	*Alcedo euryzona*	IndoMalay	0	-0.0741	2.3609	2.65	0.7128	0.2680	74.97
Alcedinidae	*Alcedo hercules*	IndoMalay	1	-0.0022	2.4043	2.84	0.7883	0.5299	11.31
Alcedinidae	*Alcedo leucogaster*	IndoMalay	0	-0.0829	4.5263	3.24	0.7857	0.2862	23.95
Alcedinidae	*Alcedo meninting*	Afrotropics	0	0.0367	7.7796	3.28	0.8133	0.2695	15.02
Alcedinidae	*Alcedo pusilla*	IndoMalay	0	-0.0843	2.4423	3.00	0.8202	0.4686	44.23
Alcedinidae	*Alcedo quadribrachys*	Australasia	0	-0.1902	1.8077	2.86	0.7833	0.7290	41.36
Alcedinidae	*Alcedo semitorquata*	Afrotropics	0	0.0189	-	3.32	0.8264	0.3166	20.83
Alcedinidae	*Alcedo vintsioides*	Afrotropics	0	-0.2787	-	3.64	0.8204	0.6223	66.58
Alcedinidae	*Alcedo websteri*	Afrotropics	0	-0.0820	6.0192	3.18	0.7614	0.3874	70.15
Alcedinidae	*Caridonax fulgidus*	Nearctic	0	-0.7792	12.2098	4.07	0.8423	0.4567	8.87
Alcedinidae	*Ceryle rudis*	Palearctic	0	-0.3505	7.0587	4.06	0.7833	0.2102	9.52
Alcedinidae	*Ceyx erithaca*	Australasia	0	-0.6994	-	3.41	0.8278	0.4667	8.12
Alcedinidae	*Ceyx fallax*	Afrotropics	0	-0.2218	-	3.47	0.8693	0.3494	15.84
Alcedinidae	*Ceyx lecontei*	Palearctic	0	-0.7258	8.4924	3.43	0.8041	-	-
Alcedinidae	*Ceyx lecontei*	Palearctic	0	-0.7258	8.4924	3.43	0.8041	0.4922	5.34
Alcedinidae	*Ceyx lepidus*	IndoMalay	0	-0.0077	0.0000	4.09	0.7493	0.1693	3.66
Alcedinidae	*Ceyx madagascariensis*	Palearctic	0	-0.1949	-	3.80	0.7939	0.2649	1.57
Alcedinidae	*Ceyx melanurus*	Palearctic	0	-0.4471	-	3.50	0.7967	0.6338	6.82
Alcedinidae	*Ceyx rufidorsa*	Palearctic	0	-0.8644	4.5177	4.04	0.8376	0.1779	2.09
Alcedinidae	*Chloroceryle aenea*	Australasia	0	-0.0987	-	2.76	0.7389	0.5637	22.08
Alcedinidae	*Chloroceryle amazona*	Australasia	0	-0.5801	-	3.58	0.7949	0.3698	3.97

Family	Species	Realm	Threat	Latitude	Elevation	Anomaly	Size	Shape	Orientation
Alcedinidae	*Chloroceryle americana*	Australasia	0	0.2519	-	2.87	0.7261	0.2314	8.31
Alcedinidae	*Chloroceryle inda*	Australasia	0	-0.5983	-	3.51	0.7989	0.6127	55.87
Alcedinidae	*Cittura cyanotis*	Afrotropics	0	-1.2130	6.8835	3.45	0.6997	0.5917	7.43
Alcedinidae	*Clytoceyx rex*	Australasia	0	-0.0591	-	2.71	0.6961	0.5662	66.33
Alcedinidae	*Dacelo gaudichaud*	IndoMalay	0	0.1030	-	2.84	0.7685	0.6418	46.68
Alcedinidae	*Dacelo leachii*	IndoMalay	0	-0.0577	-	3.24	0.8169	0.4368	17.38
Alcedinidae	*Dacelo novaeguineae*	IndoMalay	0	0.0380	0.6320	2.84	0.7672	0.4439	4.77
Alcedinidae	*Dacelo tyro*	IndoMalay	0	-0.0871	-	3.08	0.8102	0.4903	63.14
Alcedinidae	*Halcyon albiventris*	Australasia	0	-0.0629	6.7044	2.48	0.6120	0.0400	36.10
Alcedinidae	*Halcyon badia*	Neotropics	0	-0.0058	3.7011	3.62	0.7952	0.4669	41.40
Alcedinidae	*Halcyon chelicuti*	Neotropics	0	-0.9754	3.9061	3.79	0.7482	0.4072	79.01
Alcedinidae	*Halcyon coromanda*	Neotropics	0	-0.0089	-	4.08	0.7972	0.5582	13.61
Alcedinidae	*Halcyon cyanoventris*	Neotropics	0	0.1131	-	4.16	0.7922	0.8450	32.15
Alcedinidae	*Halcyon leucocephala*	Australasia	0	-0.5326	-	3.31	0.8353	0.5798	5.22
Alcedinidae	*Halcyon malimbica*	Nearctic	1	-0.5136	7.8239	4.34	0.7933	0.7395	0.55
Alcedinidae	*Halcyon pileata*	Palearctic	0	-0.4165	29.4812	4.15	0.8461	0.4434	15.29
Alcedinidae	*Halcyon senegalensis*	Afrotropics	0	-0.0748	-	3.51	0.8499	0.5318	36.53
Alcedinidae	*Halcyon senegaloides*	Afrotropics	0	-0.0667	13.8500	3.61	0.8503	0.6889	33.10
Alcedinidae	*Halcyon smyrnensis*	IndoMalay	1	-0.0399	4.2503	3.45	0.8327	0.3774	18.41
Alcedinidae	*Lacedo pulchella*	IndoMalay	0	0.0012	-	2.64	0.7310	0.2147	66.44
Alcedinidae	*Megaceryle alcyon*	IndoMalay	0	0.0157	1.8017	2.84	0.7789	0.4532	5.80
Alcedinidae	*Megaceryle lugubris*	IndoMalay	0	0.7379	0.1634	2.51	0.5788	0.0000	45.00
Alcedinidae	*Megaceryle maxima*	Neotropics	0	-4.0821	8.7932	3.71	0.7318	0.3016	61.57
Alcedinidae	*Megaceryle torquata*	Neotropics	0	0.0156	-	3.86	0.8021	0.3483	13.96
Alcedinidae	*Melidora macrorrhina*	Afrotropics	0	4.7556	6.7535	3.45	0.7120	0.4524	82.25
Alcedinidae	*Pelargopsis amauroptera*	Afrotropics	0	0.2228	8.3959	3.25	0.7537	0.7901	56.62
Alcedinidae	*Pelargopsis capensis*	Afrotropics	0	0.0162	9.9203	3.30	0.8080	0.2892	12.06
Alcedinidae	*Pelargopsis melanorhyncha*	Nearctic	0	-0.6946	15.7839	3.98	0.8129	0.4164	44.92
Alcedinidae	*Syma megarhyncha*	IndoMalay	0	0.5690	0.0000	3.17	0.5985	0.5740	49.38
Alcedinidae	*Syma torotoro*	IndoMalay	1	0.0000	6.4553	3.50	0.6562	0.7776	22.41
Alcedinidae	*Tanysiptera danae*	Afrotropics	1	-0.0002	8.4676	3.68	0.7019	0.2276	73.81
Alcedinidae	*Tanysiptera galatea*	Palearctic	0	-0.4808	-	3.67	0.7997	0.3226	7.11
Alcedinidae	*Tanysiptera hydrocharis*	Palearctic	0	-0.6457	-	4.41	0.8566	0.2791	1.33
Alcedinidae	*Tanysiptera nympha*	Palearctic	0	-0.3993	-	3.72	0.7959	0.3576	2.08
Alcedinidae	*Tanysiptera sylvia*	Palearctic	0	-0.8409	5.5177	4.79	0.8605	0.2014	4.06
Alcedinidae	*Todiramphus albonotatus*	Afrotropics	0	-0.1433	10.0237	3.51	0.8567	0.6902	31.83
Alcedinidae	*Todiramphus australasia*	Neotropics	0	-0.1577	5.0845	3.87	0.8437	0.5065	40.02
Alcedinidae	*Todiramphus chloris*	IndoMalay	0	-0.1128	-	3.52	0.8045	0.4658	11.19
Alcedinidae	*Todiramphus diops*	IndoMalay	0	-0.0467	5.9539	3.32	0.8061	0.4739	14.01
Alcedinidae	*Todiramphus farquhari*	IndoMalay	0	-0.5529	-	2.72	0.6891	0.1033	10.47
Alcedinidae	*Todiramphus funebris*	IndoMalay	0	-0.1876	-	3.40	0.8006	0.8688	2.32
Alcedinidae	*Todiramphus lazuli*	IndoMalay	0	-0.5239	10.9695	2.72	0.6859	0.0959	10.58
Alcedinidae	*Todiramphus leucopygius*	Palearctic	0	-0.8763	8.6638	3.92	0.7286	0.5054	13.29
Alcedinidae	*Todiramphus macleayii*	Palearctic	0	-0.7457	-	4.87	0.8370	0.3436	6.51
Alcedinidae	*Todiramphus nigrocyaneus*	Palearctic	0	-0.2469	-	4.96	0.8563	0.1660	2.52
Alcedinidae	*Todiramphus pyrrhopygius*	Palearctic	0	-0.8182	-	5.11	0.8511	0.1687	2.05
Alcedinidae	*Todiramphus sanctus*	Palearctic	0	2.4979	-	4.41	0.7829	0.4722	14.84
Alcedinidae	*Todiramphus saurophaga*	Palearctic	0	-1.0005	13.0396	4.09	0.7621	0.3928	17.98
Alcedinidae	*Todiramphus winchelli*	Palearctic	0	-0.8113	12.4145	3.82	0.7112	0.1797	19.46
Anatidae	*Aix galericulata*	Nearctic	0	-0.5561	-	4.04	0.7968	0.5837	39.09
Anatidae	*Aix sponsa*	Palearctic	0	-0.5918	-	4.03	0.8020	0.1529	2.87
Anatidae	*Alopochen aegyptiaca*	Neotropics	0	-0.1255	4.9046	3.37	0.8238	0.7027	84.22
Anatidae	*Amazonetta brasiliensis*	Neotropics	0	-0.0796	-	2.89	0.7136	0.7146	51.47
Anatidae	*Anas acuta*	Nearctic	0	-0.8200	-	4.44	0.8458	0.2505	10.45
Anatidae	*Anas americana*	Nearctic	0	-0.8833	-	4.28	0.8437	0.2728	13.00
Anatidae	*Anas andium*	Neotropics	0	0.1300	10.0398	3.41	0.7609	0.2686	57.33
Anatidae	*Anas bahamensis*	Neotropics	0	-0.2186	-	3.08	0.8158	0.5606	85.22
Anatidae	*Anas bernieri*	Afrotropics	1	-0.1033	0.5912	3.09	0.7060	0.2299	60.42
Anatidae	*Anas capensis*	Afrotropics	0	-0.1131	-	3.79	0.8243	0.5983	85.03
Anatidae	*Anas castanea*	Australasia	0	-0.6675	0.0000	3.17	0.8150	0.5334	1.04
Anatidae	*Anas chlorotis*	Australasia	1	-0.4656	-	2.35	0.7087	0.2997	45.62
Anatidae	*Anas clypeata*	Nearctic	0	-0.8879	-	4.34	0.8198	0.7285	11.15
Anatidae	*Anas crecca*	Palearctic	0	-0.8232	-	4.78	0.8768	0.0784	1.42
Anatidae	*Anas cyanoptera*	Nearctic	0	-0.3768	17.7053	3.57	0.8367	0.1591	58.71
Anatidae	*Anas discors*	Nearctic	0	-0.6170	-	4.18	0.8446	0.3761	24.24
Anatidae	*Anas erythrorhyncha*	Afrotropics	0	-0.0997	-	3.60	0.8493	0.5388	65.88
Anatidae	*Anas falcata*	Palearctic	0	-0.7688	-	4.67	0.8262	0.3996	4.55
Anatidae	*Anas flavirostris*	Neotropics	0	-0.3853	10.3870	2.95	0.8211	0.5239	84.82
Anatidae	*Anas formosa*	Palearctic	0	-0.6441	0.0000	5.12	0.8194	0.2577	1.72
Anatidae	*Anas fulvigula*	Nearctic	0	-0.6306	-	3.35	0.7367	0.3090	1.34
Anatidae	*Anas georgica*	Neotropics	0	-0.3473	10.6918	2.95	0.8230	0.5194	86.40
Anatidae	*Anas gibberifrons*	IndoMalay	0	-0.0376	-	2.76	0.7713	0.4531	2.25
Anatidae	*Anas gracilis*	Australasia	0	-0.3576	-	3.23	0.8420	0.7167	21.61
Anatidae	*Anas hottentota*	Afrotropics	0	-0.1210	-	3.60	0.8410	0.7137	85.94

Family	Species	Realm	Threat	Latitude	Elevation	Anomaly	Size	Shape	Orientation
Anatidae	*Anas luzonica*	IndoMalay	1	-0.0016	0.9789	2.63	0.7340	0.3306	67.67
Anatidae	*Anas melleri*	Afrotropics	1	-0.1230	8.0672	3.10	0.7340	0.2083	74.00
Anatidae	*Anas penelope*	Palearctic	0	-0.7669	-	5.30	0.8594	0.1434	0.76
Anatidae	*Anas platalea*	Neotropics	0	-0.6374	8.6401	2.76	0.8142	0.4344	76.72
Anatidae	*Anas platyrhynchos*	Palearctic	0	-0.7453	3.0385	4.46	0.8797	0.1081	0.74
Anatidae	*Anas poecilorhyncha*	IndoMalay	0	-0.1403	0.0000	3.46	0.8132	0.8270	2.55
Anatidae	*Anas puna*	Neotropics	0	-2.1266	9.2466	4.13	0.7691	0.2895	52.52
Anatidae	*Anas querquedula*	Palearctic	0	-0.8044	-	4.67	0.8616	0.1565	1.63
Anatidae	*Anas rhynchotis*	Australasia	0	-0.5325	0.0000	3.25	0.8243	0.3764	10.23
Anatidae	*Anas rubripes*	Nearctic	0	-0.8651	-	4.51	0.8231	0.4180	4.77
Anatidae	*Anas sibilatrix*	Neotropics	0	-0.6791	-	2.43	0.8030	0.5148	66.08
Anatidae	*Anas smithii*	Afrotropics	0	-0.2632	-	3.87	0.8073	0.7549	39.26
Anatidae	*Anas sparsa*	Afrotropics	0	-0.1047	-	3.58	0.8448	0.5820	70.74
Anatidae	*Anas strepera*	Palearctic	0	-0.7444	-	4.48	0.8645	0.0707	1.57
Anatidae	*Anas superciliosa*	Australasia	0	-0.3579	-	3.22	0.8419	0.6433	24.65
Anatidae	*Anas undulata*	Afrotropics	0	-0.1278	18.0314	3.59	0.8424	0.4888	68.27
Anatidae	*Anas versicolor*	Neotropics	0	-0.5722	10.4072	2.57	0.8137	0.3853	59.73
Anatidae	*Anas zonorhyncha*	Palearctic	0	-0.7804	0.0000	3.91	0.8421	0.6570	38.45
Anatidae	*Anser albifrons*	Nearctic	0	-1.2632	5.7013	5.13	0.8076	0.1133	1.55
Anatidae	*Anser anser*	Palearctic	0	-0.8301	-	4.59	0.8571	0.3280	7.29
Anatidae	*Anser brachyrhynchus*	Nearctic	0	-1.0216	-	5.26	0.7344	0.4836	5.26
Anatidae	*Anser cygnoides*	Palearctic	1	-1.1264	-	4.40	0.8069	0.1690	1.36
Anatidae	*Anser erythropus*	Palearctic	1	-1.1959	1.4220	6.10	0.8132	0.0476	0.87
Anatidae	*Anser fabalis*	Palearctic	0	-0.6760	0.0000	5.38	0.8549	0.1910	1.35
Anatidae	*Anser indicus*	Palearctic	0	3.7428	-	4.43	0.8087	0.4346	22.63
Anatidae	*Aythya affinis*	IndoMalay	0	-0.2239	4.6628	3.30	0.8147	0.6201	22.86
Anatidae	*Aythya americana*	Afrotropics	0	-0.1226	5.6118	3.10	0.7488	0.4654	70.20
Anatidae	*Aythya australis*	Australasia	0	-0.3109	2.9517	2.98	0.7990	0.5704	49.76
Anatidae	*Aythya baeri*	Neotropics	0	0.0931	0.9243	3.71	0.7739	0.5038	15.42
Anatidae	*Aythya collaris*	Nearctic	0	-0.5692	-	4.18	0.8306	0.3495	23.54
Anatidae	*Aythya ferina*	Nearctic	0	-0.7809	6.9905	4.20	0.8302	0.4802	19.35
Anatidae	*Aythya fuligula*	Australasia	0	-0.5427	0.0000	3.35	0.8352	0.5831	8.89
Anatidae	*Aythya innotata*	Palearctic	1	-0.7094	-	4.44	0.8049	0.6928	10.63
Anatidae	*Aythya novaeseelandiae*	Nearctic	0	-1.0039	-	4.24	0.8305	0.2681	8.10
Anatidae	*Aythya nyroca*	Palearctic	0	-0.8625	-	4.70	0.8608	0.1904	1.53
Anatidae	*Aythya valisineria*	Palearctic	0	-0.7493	-	4.87	0.8656	0.1482	1.39
Anatidae	*Biziura lobata*	Afrotropics	0	-0.0984	-	3.16	0.7554	0.4484	70.65
Anatidae	*Branta bernicla*	IndoMalay	0	-0.3116	5.1811	2.69	0.6523	0.2154	10.94
Anatidae	*Branta canadensis*	IndoMalay	0	0.2238	-	2.76	0.6120	0.6124	0.00
Anatidae	*Branta hutchinsii*	IndoMalay	0	-0.0072	5.3187	2.65	0.6428	0.4267	90.00
Anatidae	*Branta leucopsis*	Afrotropics	1	-0.9154	-	2.73	0.6598	0.1050	14.42
Anatidae	*Branta ruficollis*	Palearctic	0	-0.7379	4.6152	3.92	0.8108	0.5927	50.39
Anatidae	*Branta sandvicensis*	Palearctic	0	-0.6491	10.6778	3.86	0.7881	0.5082	8.57
Anatidae	*Bucephala albeola*	Afrotropics	1	0.2520	6.0000	3.22	0.6804	0.2330	46.41
Anatidae	*Cairina moschata*	Neotropics	0	-15.8028	-	3.63	0.7464	0.5231	74.41
Anatidae	*Cairina scutulata*	Neotropics	0	-0.2136	-	3.85	0.8431	0.8470	11.66
Anatidae	*Callonetta leucophrys*	Palearctic	0	-6.9404	6.2694	4.51	0.7060	0.2212	48.51
Anatidae	*Cereopsis novaehollandiae*	Neotropics	0	-0.2481	2.2404	3.88	0.7558	0.1981	17.86
Anatidae	*Chen caerulescens*	Australasia	0	-0.1347	4.3072	2.48	0.6221	0.0384	35.15
Anatidae	*Chen canagica*	Australasia	0	-0.0540	6.6348	2.48	0.6120	0.0400	36.10
Anatidae	*Chen rossii*	Australasia	0	-0.3723	5.4170	2.93	0.7120	0.2179	23.56
Anatidae	*Chloephaga hybrida*	Palearctic	0	-0.4336	6.1736	3.74	0.7578	0.2923	12.49
Anatidae	*Chloephaga melanoptera*	Neotropics	0	-0.8985	8.2966	4.03	0.7185	0.1631	41.70
Anatidae	*Chloephaga picta*	Neotropics	0	-0.3278	-	3.22	0.7929	0.4601	33.95
Anatidae	*Chloephaga poliocephala*	Neotropics	0	0.1292	-	3.70	0.7572	0.2405	2.78
Anatidae	*Chloephaga rubidiceps*	Neotropics	0	-0.0097	-	3.48	0.7211	0.1557	31.24
Anatidae	*Coscoroba coscoroba*	IndoMalay	0	-0.0956	-	3.46	0.8251	0.5429	13.04
Anatidae	*Cyanochen cyanoptera*	IndoMalay	0	-0.6244	10.2077	3.42	0.8276	0.5560	10.10
Anatidae	*Cygnus atratus*	Neotropics	0	0.0611	7.1296	3.00	0.6370	0.3956	30.13
Anatidae	*Cygnus buccinator*	Neotropics	0	0.1934	6.9990	3.51	0.7256	0.2372	52.45
Anatidae	*Cygnus columbianus*	Neotropics	0	-0.0866	7.0006	3.54	0.7318	0.1304	34.10
Anatidae	*Cygnus cygnus*	Neotropics	1	1.0902	11.6514	3.50	0.6763	0.2056	17.52
Anatidae	*Cygnus melancoryphus*	Neotropics	1	0.0987	10.2970	3.72	0.6598	0.2828	56.12
Anatidae	*Cygnus olor*	Neotropics	0	0.3855	7.5006	2.98	0.6859	0.1424	65.48
Anatidae	*Dendrocygna arborea*	IndoMalay	0	-0.5000	-	3.44	0.8409	0.5624	50.33
Anatidae	*Dendrocygna arcuata*	IndoMalay	0	-0.5885	9.3752	3.45	0.7762	0.6496	23.76
Anatidae	*Dendrocygna autumnalis*	Palearctic	0	-0.2637	12.8820	3.65	0.7645	0.3109	8.17
Anatidae	*Dendrocygna bicolor*	Afrotropics	1	-0.2049	10.0363	3.92	0.7296	0.1184	65.42
Anatidae	*Dendrocygna eytoni*	IndoMalay	0	-5.7144	9.3802	4.33	0.7344	0.1607	29.34
Anatidae	*Dendrocygna guttata*	IndoMalay	0	-0.8315	15.2794	3.76	0.8037	0.3938	22.18
Anatidae	*Dendrocygna javanica*	Palearctic	0	-0.8484	5.4314	3.97	0.7941	0.6263	21.52
Anatidae	*Dendrocygna viduata*	Palearctic	0	-1.0181	8.9268	4.36	0.8102	0.3255	3.53
Anatidae	*Heteronetta atricapilla*	IndoMalay	0	-0.0335	-	3.23	0.7940	0.4678	49.61
Anatidae	*Hymenolaimus*	Neotropics	0	-0.1528	1.5782	3.93	0.8244	0.6859	52.23

Family	Species	Realm	Threat	Latitude	Elevation	Anomaly	Size	Shape	Orientation
	malacorhynchos								
Anatidae	*Lophonetta specularioides*	IndoMalay	0	-1.0333	9.4257	2.61	0.6478	0.3444	72.46
Anatidae	*Malacorhynchus membranaceus*	Neotropics	0	-0.0771	-	3.79	0.8072	0.4973	30.39
Anatidae	*Merganetta armata*	Neotropics	0	-0.4905	9.6240	3.32	0.7770	0.5849	85.22
Anatidae	*Mergellus albellus*	Neotropics	0	-0.2565	6.4241	3.99	0.8117	0.8496	22.45
Anatidae	*Mergus octosetaceus*	IndoMalay	1	0.0411	2.2848	2.84	0.7865	0.4329	0.53
Anatidae	*Mergus squamatus*	Neotropics	0	-0.1977	0.0000	3.08	0.7245	0.5667	46.63
Anatidae	*Neochen jubata*	Afrotropics	0	-0.1706	-	3.45	0.7683	0.5545	78.44
Anatidae	*Netta erythrophthalma*	Afrotropics	0	-0.2345	-	3.81	0.8142	0.7125	9.76
Anatidae	*Netta peposaca*	Afrotropics	0	1.2144	6.3145	3.17	0.6804	0.5718	40.11
Anatidae	*Netta rufina*	Afrotropics	0	-0.0863	-	3.46	0.8349	0.5897	8.18
Anatidae	*Nettapus auritus*	Afrotropics	0	-0.1695	-	3.06	0.7422	0.1091	67.54
Anatidae	*Nettapus coromandelianus*	Afrotropics	0	-0.0435	-	3.32	0.8314	0.3512	18.02
Anatidae	*Nettapus pulchellus*	Afrotropics	0	-0.9247	-	2.88	0.7069	0.3372	8.59
Anatidae	*Nomonyx dominicus*	Neotropics	0	-0.2775	5.1433	3.88	0.8061	0.6343	27.08
Anatidae	*Oxyura australis*	IndoMalay	0	-0.0572	-	3.24	0.8084	0.3966	30.34
Anatidae	*Oxyura jamaicensis*	IndoMalay	0	-0.0181	2.4324	2.83	0.7901	0.6063	13.78
Anatidae	*Oxyura leucocephala*	IndoMalay	0	-0.0994	-	3.26	0.8238	0.4673	8.96
Anatidae	*Oxyura maccoa*	Nearctic	0	-0.2747	7.5064	3.99	0.7495	0.2397	44.82
Anatidae	*Oxyura vittata*	Neotropics	0	0.1451	3.0574	3.29	0.7537	0.6100	42.27
Anatidae	*Plectropterus gambensis*	Neotropics	0	0.0696	10.7708	3.46	0.7590	0.2907	65.64
Anatidae	*Pteronetta hartlaubii*	Neotropics	0	-0.3874	-	3.34	0.7165	0.5784	26.10
Anatidae	*Rhodonessa caryophyllacea*	Afrotropics	0	0.1073	6.2869	3.30	0.8181	0.2992	15.30
Anatidae	*Salvadorina waigiuensis*	Australasia	0	-0.0827	-	2.66	0.7291	0.2490	24.16
Anatidae	*Sarkidiornis melanotos*	Neotropics	0	0.4843	-	3.84	0.7449	0.2465	16.29
Anatidae	*Speculanas specularis*	IndoMalay	0	-0.7051	6.9145	3.29	0.7574	0.4090	25.91
Anatidae	*Stictonetta naevosa*	IndoMalay	1	-0.1098	-5.6100	2.90	0.5788	0.0000	90.00
Anatidae	*Tadorna cana*	Neotropics	0	-0.3225	-	3.27	0.7903	0.2572	55.52
Anatidae	*Tadorna cristata*	Neotropics	0	0.2151	5.1737	4.14	0.7422	0.4029	36.48
Anatidae	*Tadorna ferruginea*	Neotropics	0	0.0325	10.3061	3.69	0.7979	0.2956	44.62
Anatidae	*Tadorna radjah*	Neotropics	0	-0.2733	16.2460	3.84	0.7498	0.2221	42.26
Anatidae	*Tadorna tadorna*	Neotropics	0	-0.1671	-	3.72	0.8425	0.4440	46.61
Anatidae	*Tadorna tadornoides*	Australasia	0	-0.1562	-	2.50	0.5788	0.0000	45.57
Anatidae	*Tadorna variegata*	Australasia	1	0.0163	1.1944	2.00	0.6370	0.4364	43.26
Anatidae	*Thalassornis leuconotus*	Afrotropics	0	-0.3504	3.3475	3.60	0.8806	0.5289	10.81
Anhimidae	*Anhima cornuta*	Neotropics	0	-0.1128	-	3.98	0.8465	0.6312	28.38
Anhimidae	*Chauna chavaria*	Neotropics	0	-0.2677	5.7547	3.88	0.8035	0.5474	27.03
Anhingidae	*Anhinga anhinga*	Neotropics	0	-0.3001	-	3.75	0.8571	0.4188	45.36
Anhingidae	*Anhinga melanogaster*	IndoMalay	0	-0.0546	-	3.17	0.8374	0.3086	28.33
Anhingidae	*Anhinga novaehollandiae*	Australasia	0	-0.3748	0.0000	3.27	0.8362	0.8546	0.42
Anhingidae	*Anhinga rufa*	Afrotropics	0	-0.1688	-	3.53	0.8615	0.7804	49.28
Anseranatidae	*Anseranas semipalmata*	Australasia	0	-0.4899	0.0000	3.04	0.7413	0.5157	50.08
Apodidae	*Aeronautes andecolus*	Neotropics	0	-0.1989	10.9241	3.69	0.7838	0.2952	72.52
Apodidae	*Aeronautes montivagus*	Neotropics	0	-0.0780	9.7451	3.93	0.7816	0.6080	76.03
Apodidae	*Aeronautes saxatalis*	Nearctic	0	-0.4611	9.1595	4.04	0.8227	0.3369	52.30
Apodidae	*Apus acuticauda*	IndoMalay	1	-0.0503	5.5227	3.37	0.6562	0.1659	62.25
Apodidae	*Apus affinis*	Afrotropics	0	-0.2247	-	3.56	0.8674	0.6124	19.22
Apodidae	*Apus apus*	Palearctic	0	-0.7257	-	4.40	0.8698	0.2596	1.80
Apodidae	*Apus balstoni*	Afrotropics	0	-0.0774	-	3.18	0.7614	0.3874	70.15
Apodidae	*Apus barbatus*	Afrotropics	0	-0.2474	10.6851	3.45	0.7895	0.7099	86.70
Apodidae	*Apus batesi*	Afrotropics	0	0.0130	5.0454	3.20	0.7261	0.1044	10.59
Apodidae	*Apus berliozi*	Afrotropics	0	-0.0586	-	3.13	0.7069	0.2523	60.13
Apodidae	*Apus bradfieldi*	Afrotropics	0	-0.2212	-	3.99	0.7785	0.3544	54.61
Apodidae	*Apus caffer*	Afrotropics	0	-0.2710	9.9134	3.51	0.8530	0.7302	46.88
Apodidae	*Apus horus*	Afrotropics	0	-0.1503	9.2475	3.58	0.8079	0.4658	80.48
Apodidae	*Apus niansae*	Afrotropics	0	0.0538	-	3.41	0.7613	0.5186	60.49
Apodidae	*Apus nipalensis*	IndoMalay	0	-0.2725	-	3.09	0.8314	0.8267	62.67
Apodidae	*Apus pacificus*	Palearctic	0	-0.8674	12.5293	4.20	0.8565	0.5538	23.96
Apodidae	*Apus pallidus*	Palearctic	0	-0.6132	-	3.81	0.8081	0.3149	10.32
Apodidae	*Apus sladeniae*	Afrotropics	0	-151.9491	-	3.58	0.6631	0.0513	72.58
Apodidae	*Apus toulsoni*	Afrotropics	0	-0.2280	-	3.17	0.6763	0.0682	88.80
Apodidae	*Chaetura brachyura*	Afrotropics	0	-0.1530	-	3.30	0.8311	0.4511	25.80
Apodidae	*Chaetura chapmani*	IndoMalay	0	-0.1007	2.7150	2.95	0.8152	0.5023	42.56
Apodidae	*Chaetura cinereiventris*	Australasia	0	-0.0708	-	2.70	0.7165	0.5531	61.27
Apodidae	*Chaetura egregia*	Afrotropics	0	0.0386	-	3.24	0.8068	0.2542	10.14
Apodidae	*Chaetura fumosa*	Australasia	0	-0.0033	-	2.73	0.7793	0.2627	30.79
Apodidae	*Chaetura meridionalis*	Afrotropics	0	-0.0706	5.1029	3.17	0.7570	0.4271	70.48
Apodidae	*Chaetura pelagica*	IndoMalay	1	-0.0169	1.9356	2.64	0.7256	0.2114	67.51
Apodidae	*Chaetura spinicaudus*	Afrotropics	0	-0.1005	-	3.47	0.8552	0.5655	29.66
Apodidae	*Chaetura vauxi*	IndoMalay	0	-0.0433	-	2.81	0.7934	0.6420	4.31
Apodidae	*Chaetura viridipennis*	Neotropics	0	0.3215	6.5501	3.25	0.6302	0.6246	0.00
Apodidae	*Collocalia amelis*	Neotropics	0	-0.3299	-	3.39	0.8366	0.3944	46.86
Apodidae	*Collocalia brevirostris*	Neotropics	0	-0.4982	2.2454	2.40	0.7567	0.1771	83.76

Family	Species	Realm	Threat	Latitude	Elevation	Anomaly	Size	Shape	Orientation
Apodidae	*Collocalia esculenta*	Neotropics	0	-0.0068	-	3.96	0.8195	0.8060	77.09
Apodidae	*Collocalia fuciphaga*	Neotropics	0	0.0673	14.6191	3.66	0.7700	0.3978	78.02
Apodidae	*Collocalia germani*	Neotropics	0	-0.1015	8.3815	3.72	0.8133	0.5155	43.18
Apodidae	*Collocalia hirundinacea*	Neotropics	0	-0.1361	17.2792	4.03	0.7821	0.2553	51.13
Apodidae	*Collocalia infuscata*	Neotropics	0	-0.0668	15.1616	3.80	0.7890	0.4422	77.36
Apodidae	*Collocalia linchi*	Neotropics	0	-0.0550	9.5338	3.88	0.7962	0.7810	33.29
Apodidae	*Collocalia maxima*	Neotropics	0	-0.2778	6.6261	3.72	0.8082	0.4658	7.23
Apodidae	*Collocalia mearnsi*	Neotropics	0	-0.1274	9.9276	3.67	0.7899	0.3710	45.42
Apodidae	*Collocalia nuditarsus*	Neotropics	0	-0.0304	-	3.79	0.8031	0.3250	13.24
Apodidae	*Collocalia orientalis*	Neotropics	0	-0.0592	-	2.88	0.7120	0.3293	51.17
Apodidae	*Collocalia palawanensis*	Nearctic	0	-0.5488	6.5847	4.06	0.8258	0.7471	7.95
Apodidae	*Collocalia papuensis*	Afrotropics	0	-0.1806	8.9588	3.52	0.7449	0.6031	74.52
Apodidae	*Collocalia rogersi*	Afrotropics	0	-0.4376	-	3.92	0.7957	0.5854	46.38
Apodidae	*Collocalia salangana*	Afrotropics	0	-0.2872	-	3.16	0.7631	0.4238	38.30
Apodidae	*Collocalia spodiopygia*	Afrotropics	0	-0.0899	11.7080	3.42	0.8415	0.7551	83.69
Apodidae	*Collocalia troglodytes*	IndoMalay	0	-0.0030	-	2.64	0.7305	0.3116	67.95
Apodidae	*Collocalia unicolor*	Palearctic	0	-0.6108	15.0879	3.50	0.8063	0.4068	1.87
Apodidae	*Collocalia vanikorensis*	IndoMalay	0	-0.0676	9.0924	2.77	0.8086	0.3137	11.78
Apodidae	*Collocalia vulcanorum*	IndoMalay	0	0.0191	5.4744	2.82	0.7887	0.5692	6.13
Apodidae	*Collocalia whiteheadi*	IndoMalay	0	-0.1843	-	2.79	0.7474	0.5667	4.10
Apodidae	*Cypseloides cherriei*	IndoMalay	0	-0.3747	-	3.10	0.7941	0.7048	25.56
Apodidae	*Cypseloides cryptus*	IndoMalay	0	-0.0102	4.2311	2.66	0.6997	0.4736	75.00
Apodidae	*Cypseloides fumigatus*	Australasia	0	-0.1194	-	2.77	0.6842	0.7102	61.42
Apodidae	*Cypseloides lemosi*	Australasia	0	-0.1153	-	2.56	0.6523	0.1875	30.24
Apodidae	*Cypseloides niger*	IndoMalay	0	0.1888	-	2.52	0.5985	0.0024	45.41
Apodidae	*Cypseloides rothschildi*	IndoMalay	0	0.2337	0.0000	3.62	0.7363	0.3645	5.19
Apodidae	*Cypseloides senex*	Australasia	0	-0.0362	-	2.71	0.7185	0.5623	60.71
Apodidae	*Cypseloides storeri*	IndoMalay	0	-0.0299	7.0721	3.05	0.7060	0.1449	63.74
Apodidae	*Cypsiurus balasiensis*	IndoMalay	0	-0.1918	-	3.26	0.7696	0.4572	10.22
Apodidae	*Cypsiurus parvus*	IndoMalay	0	-0.2419	-	3.35	0.8074	0.6614	17.71
Apodidae	*Hirundapus celebensis*	Australasia	0	-0.3797	-	3.51	0.7901	0.3177	7.96
Apodidae	*Hirundapus cochinchinensis*	Australasia	0	-0.2126	9.3187	2.80	0.7425	0.1601	23.69
Apodidae	*Hirundapus giganteus*	Neotropics	0	-0.6129	-	2.82	0.8021	0.6927	54.22
Apodidae	*Hydrochous gigas*	Neotropics	0	-0.1938	12.6470	3.67	0.8307	0.7320	48.33
Apodidae	*Mearnsia novaeguineae*	Afrotropics	0	-0.1312	-	3.53	0.8416	0.5997	32.73
Apodidae	*Mearnsia picina*	Afrotropics	0	0.0458	4.4692	3.21	0.8064	0.2206	9.66
Apodidae	*Neafrapus boehmi*	Neotropics	0	0.0019	-	4.09	0.8297	0.8759	10.27
Apodidae	*Neafrapus cassini*	Neotropics	0	0.1961	-	4.00	0.7567	0.1334	3.82
Apodidae	*Panyptila cayennensis*	IndoMalay	0	0.0153	6.9657	2.66	0.6906	0.4993	77.84
Apodidae	*Panyptila sanctihieronymi*	Australasia	0	0.0633	2.9081	2.55	0.7050	0.4844	31.68
Apodidae	*Rhaphidura leucopygialis*	IndoMalay	0	-0.0572	-	2.83	0.7912	0.5799	8.42
Apodidae	*Rhaphidura sabini*	Palearctic	0	-0.7054	-	3.37	0.8100	0.7796	39.43
Apodidae	*Schoutedenapus myoptilus*	IndoMalay	0	-0.6210	8.2978	2.72	0.6859	0.0948	10.27
Apodidae	*Schoutedenapus schoutedeni*	Australasia	0	-0.0842	-	2.78	0.7653	0.2422	23.46
Apodidae	*Streptoprocne biscutata*	Afrotropics	0	3.4478	-	3.32	0.7607	0.5163	3.32
Apodidae	*Streptoprocne phelpsi*	Afrotropics	0	0.0179	-	3.34	0.7735	0.4968	31.11
Apodidae	*Streptoprocne rutila*	Australasia	0	-0.3847	1.8438	3.24	0.7833	0.4035	34.10
Apodidae	*Streptoprocne semicollaris*	Australasia	0	-0.1035	0.4703	2.56	0.6523	0.1875	30.24
Apodidae	*Streptoprocne zonaris*	Afrotropics	1	0.1205	-	2.68	0.6804	0.3531	39.90
Apodidae	*Tachornis furcata*	Australasia	0	-0.7451	0.0000	3.14	0.8029	0.4206	0.86
Apodidae	*Tachornis phoenicobia*	IndoMalay	0	-0.3502	5.9467	3.21	0.8494	0.5372	35.83
Apodidae	*Tachornis squamata*	Afrotropics	0	-0.2890	10.8616	3.71	0.8710	0.5851	23.21
Apodidae	*Tachymarptis aequatorialis*	Afrotropics	0	-0.1291	-	3.61	0.8418	0.5332	26.73
Apodidae	*Tachymarptis melba*	Afrotropics	0	0.1074	-	3.34	0.7406	0.6239	34.40
Apodidae	*Telacanthura melanopygia*	Neotropics	0	-0.2064	13.8669	3.67	0.7736	0.3330	66.60
Apodidae	*Telacanthura ussheri*	Neotropics	0	-0.6245	0.0000	4.25	0.6824	0.1887	21.98
Apodidae	*Zoonavena grandidieri*	Palearctic	0	-0.6020	-	4.58	0.8634	0.1902	2.15
Apodidae	*Zoonavena sylvatica*	Afrotropics	0	-0.0744	-	3.17	0.7600	0.3849	70.09
Apterygidae	*Apteryx australis*	Australasia	1	-0.7905	5.8334	1.99	0.6302	0.3492	38.80
Apterygidae	*Apteryx haastii*	Australasia	1	-0.0212	6.4161	2.33	0.6804	0.2473	40.38
Apterygidae	*Apteryx mantelli*	Australasia	1	-0.3309	3.3345	2.51	0.6961	0.6462	40.45
Aramidae	*Aramus guarauna*	Neotropics	1	0.3293	2.9808	3.00	0.7223	0.1613	66.08
Ardeidae	*Agamia agami*	Afrotropics	1	1.5817	6.3177	3.35	0.7722	0.6772	10.27
Ardeidae	*Ardea cinerea*	Australasia	0	-0.6866	8.1255	2.91	0.7120	0.1454	17.55
Ardeidae	*Ardea cocoi*	Nearctic	0	-0.6087	-	4.10	0.8091	0.3677	38.18
Ardeidae	*Ardea goliath*	Nearctic	0	-0.5894	-	4.24	0.8287	0.5948	9.03
Ardeidae	*Ardea herodias*	Palearctic	0	-0.4313	11.5986	3.86	0.8874	0.4771	20.96
Ardeidae	*Ardea humbloti*	Neotropics	0	-0.3073	-	3.68	0.8563	0.7670	73.26
Ardeidae	*Ardea insignis*	Afrotropics	0	-0.1634	8.2931	3.53	0.8614	0.7375	25.67
Ardeidae	*Ardea melanocephala*	Nearctic	0	-0.7930	-	4.17	0.8531	0.5770	8.05
Ardeidae	*Ardea pacifica*	Afrotropics	1	-0.1963	3.5329	3.09	0.7096	0.2876	70.46
Ardeidae	*Ardea picata*	IndoMalay	1	-0.1230	3.7851	3.64	0.7019	0.2112	4.17
Ardeidae	*Ardea purpurea*	Afrotropics	0	-0.0834	-	3.57	0.8630	0.7430	44.28
Ardeidae	*Ardea sumatrana*	Australasia	0	-0.5052	0.0000	3.38	0.8350	0.5602	8.07

Family	Species	Realm	Threat	Latitude	Elevation	Anomaly	Size	Shape	Orientation
Ardeidae	*Ardeola bacchus*	Australasia	0	-0.2115	-	3.18	0.7305	0.3585	44.67
Ardeidae	*Ardeola grayii*	Afrotropics	0	-0.3355	5.6778	3.55	0.8791	0.4931	13.12
Ardeidae	*Ardeola idae*	Australasia	0	-0.0964	-	2.87	0.8067	0.3303	25.11
Ardeidae	*Ardeola ralloides*	Palearctic	0	-0.8481	-	3.57	0.8299	0.4607	28.11
Ardeidae	*Ardeola rufiventris*	IndoMalay	0	-0.0866	-	3.48	0.8184	0.6136	10.34
Ardeidae	*Ardeola speciosa*	Afrotropics	1	-0.0638	6.1118	3.18	0.7604	0.4035	70.14
Ardeidae	*Botaurus lentiginosus*	Nearctic	0	-0.6720	-	4.16	0.8426	0.2310	12.33
Ardeidae	*Botaurus pinnatus*	Afrotropics	0	0.2769	15.0546	3.56	0.7648	0.6376	71.95
Ardeidae	*Botaurus poiciloptilus*	Afrotropics	0	-0.0860	-	3.51	0.8590	0.6404	36.70
Ardeidae	*Botaurus stellaris*	Afrotropics	0	-0.0143	12.2616	3.17	0.7806	0.1392	11.71
Ardeidae	*Bubulcus ibis*	IndoMalay	0	0.0037	11.3576	3.34	0.8101	0.7205	8.35
Ardeidae	*Butorides striata*	Nearctic	0	-0.8530	7.7692	4.16	0.8392	0.4004	37.94
Ardeidae	*Butorides virescens*	Neotropics	0	-0.4812	4.0863	1.99	0.7619	0.2991	84.11
Ardeidae	*Casmerodius albus*	Palearctic	0	-0.7983	8.0097	4.22	0.8004	0.1648	9.07
Ardeidae	*Cochlearius cochlearius*	Nearctic	0	-0.7369	10.8807	4.06	0.8255	0.4464	2.55
Ardeidae	*Egretta ardesiaca*	Australasia	0	-0.2187	-	2.48	0.6690	0.5741	71.12
Ardeidae	*Egretta caerulea*	Australasia	0	-0.1051	-	2.77	0.7702	0.3101	23.75
Ardeidae	*Egretta eulophotes*	Australasia	0	-0.3234	-	2.49	0.6824	0.2324	22.19
Ardeidae	*Egretta garzetta*	IndoMalay	0	-0.0039	3.7199	2.64	0.7327	0.2318	67.52
Ardeidae	*Egretta gularis*	Australasia	0	-0.1043	9.1893	2.71	0.7179	0.5352	59.02
Ardeidae	*Egretta novaehollandiae*	Australasia	0	0.2724	-	2.57	0.6716	0.2061	1.81
Ardeidae	*Egretta rufescens*	Australasia	0	-0.0665	3.6893	2.49	0.6824	0.2324	22.19
Ardeidae	*Egretta sacra*	Australasia	0	-0.0900	-	2.78	0.7709	0.3038	24.05
Ardeidae	*Egretta thula*	Australasia	0	-0.1945	1.7923	2.94	0.7680	0.7905	2.70
Ardeidae	*Egretta tricolor*	Australasia	0	-0.3270	3.0954	2.50	0.6716	0.2680	24.42
Ardeidae	*Egretta vinaceigula*	Australasia	0	-0.0938	-	2.78	0.7715	0.3090	23.60
Ardeidae	*Gorsachius goisagi*	Neotropics	0	-0.1989	6.0906	3.73	0.8569	0.4796	42.18
Ardeidae	*Gorsachius leuconotus*	IndoMalay	0	-0.1985	7.6893	3.29	0.8164	0.8334	31.38
Ardeidae	*Gorsachius magnificus*	Afrotropics	0	-0.2849	5.5365	3.69	0.8212	0.6908	31.45
Ardeidae	*Gorsachius melanolophus*	Afrotropics	0	0.1090	4.0924	3.23	0.7535	0.0924	12.08
Ardeidae	*Ixobrychus cinnamomeus*	Neotropics	1	-2.7773	4.7511	3.55	0.6302	0.3793	12.93
Ardeidae	*Ixobrychus eurhythmus*	Neotropics	0	-0.4468	6.9743	3.62	0.6876	0.5332	48.81
Ardeidae	*Ixobrychus exilis*	Neotropics	0	-0.0526	9.0153	3.62	0.6824	0.1576	64.52
Ardeidae	*Ixobrychus flavicollis*	Neotropics	0	0.0000	2.2319	3.50	0.6120	0.6124	0.00
Ardeidae	*Ixobrychus involucris*	IndoMalay	0	0.0539	1.4152	2.84	0.7868	0.4518	4.71
Ardeidae	*Ixobrychus minutus*	Afrotropics	0	-0.0449	-	3.32	0.8241	0.4153	17.25
Ardeidae	*Ixobrychus sinensis*	Afrotropics	0	-0.1363	-	3.53	0.8502	0.6899	39.11
Ardeidae	*Ixobrychus sturmii*	Afrotropics	0	-0.1051	-	3.28	0.8222	0.2462	13.61
Ardeidae	*Mesophoyx intermedia*	Australasia	0	-0.5233	-	3.23	0.7695	0.3559	80.39
Ardeidae	*Nyctanassa violacea*	IndoMalay	0	-0.0658	-	3.17	0.8015	0.4826	66.88
Ardeidae	*Nycticorax caledonicus*	Australasia	0	-0.4667	8.1249	3.30	0.8392	0.5169	14.68
Ardeidae	*Nycticorax nycticorax*	Australasia	0	0.0758	-	2.72	0.7136	0.6565	58.63
Ardeidae	*Pilherodius pileatus*	Palearctic	0	-0.8749	-	4.20	0.7820	0.4257	6.37
Ardeidae	*Tigriornis leucolopha*	Afrotropics	0	-0.6534	-	3.13	0.7425	0.2937	36.60
Ardeidae	*Tigrisoma fasciatum*	Afrotropics	0	0.1289	-	3.15	0.7705	0.1368	10.09
Ardeidae	*Tigrisoma lineatum*	Afrotropics	0	-0.0236	-	3.33	0.8305	0.2986	29.47
Ardeidae	*Tigrisoma mexicanum*	Neotropics	0	-0.5875	-	3.09	0.7648	0.2772	83.38
Ardeidae	*Zebrilus undulatus*	Afrotropics	0	0.0139	-	3.26	0.8152	0.3306	13.64
Ardeidae	*Zonerodius heliosylus*	Neotropics	0	-0.0142	8.5923	3.96	0.7789	0.2116	56.36
Artamidae	*Artamus cinereus*	Australasia	0	0.1298	-	2.79	0.7281	0.2156	21.48
Artamidae	*Artamus cyanopterus*	Australasia	0	-0.2898	-	3.01	0.6631	0.2435	70.72
Artamidae	*Artamus fuscus*	Australasia	0	-0.1119	-	2.79	0.7689	0.4600	27.82
Artamidae	*Artamus insignis*	Afrotropics	0	-0.0891	-	3.15	0.7552	0.3938	69.49
Artamidae	*Artamus leucorynchus*	Australasia	0	-0.4163	-	3.35	0.8374	0.6138	2.41
Artamidae	*Artamus maximus*	Australasia	0	-0.6395	-	3.18	0.8202	0.4762	4.93
Artamidae	*Artamus mentalis*	IndoMalay	0	-0.1501	-	3.22	0.8259	0.4137	1.60
Artamidae	*Artamus minor*	Australasia	0	-0.3240	-	2.50	0.6716	0.2680	24.42
Artamidae	*Artamus monachus*	Australasia	0	-0.2169	-	3.16	0.8386	0.4427	41.16
Artamidae	*Artamus personatus*	Australasia	0	-0.1024	-	2.79	0.7522	0.1825	24.48
Atrichornithidae	*Atrichornis clamosus*	Neotropics	0	-0.5275	7.2145	3.36	0.7008	0.1894	69.58
Atrichornithidae	*Atrichornis rufescens*	Neotropics	0	-0.3105	8.1539	3.83	0.7198	0.3748	29.45
Balaenicipitidae	*Balaeniceps rex*	Nearctic	0	-0.5054	8.1811	3.37	0.7389	0.2289	53.05
Bombycillidae	*Bombycilla cedrorum*	Neotropics	0	0.1394	8.2951	3.69	0.7385	0.2346	64.27
Bombycillidae	*Bombycilla garrulus*	IndoMalay	0	-0.0117	-	2.64	0.7256	0.2114	67.51
Bombycillidae	*Bombycilla japonica*	Neotropics	1	5.0506	4.9199	3.12	0.6662	0.1831	69.10
Bombycillidae	*Hypocolius ampelinus*	Neotropics	0	-0.0382	-	3.20	0.7493	-	-
Bombycillidae	*Hypocolius ampelinus*	Neotropics	0	-0.0382	-	3.20	0.7493	0.3970	43.43
Bombycillidae	*Phainopepla nitens*	Nearctic	0	-0.3119	7.1131	3.68	0.7827	0.2915	19.53
Bombycillidae	*Phainoptila melanoxantha*	Nearctic	0	-0.7291	10.0417	4.16	0.8524	0.4479	18.50
Bombycillidae	*Ptilogonys caudatus*	Neotropics	0	-0.2264	18.9073	3.93	0.7792	0.3746	65.33
Bombycillidae	*Ptilogonys cinereus*	Neotropics	0	3.3339	7.0988	3.68	0.7590	0.2461	84.59
Brachypteraciidae	*Atelornis crossleyi*	Australasia	0	-0.1978	8.8081	2.78	0.6428	0.2952	21.65
Brachypteraciidae	*Atelornis pittoides*	Australasia	0	0.0233	3.6400	2.85	0.6948	0.1702	13.46
Brachypteraciidae	*Brachypteracias leptosomus*	Afrotropics	0	0.1699	-	3.28	0.7531	0.5602	3.37

Family	Species	Realm	Threat	Latitude	Elevation	Anomaly	Size	Shape	Orientation
Brachypteraciidae	*Brachypteracias squamiger*	Neotropics	0	0.0983	-	4.13	0.7697	0.7892	73.42
Brachypteraciidae	*Uratelornis chimaera*	Neotropics	0	0.0257	-	3.31	0.7645	0.2165	39.93
Bucconidae	*Bucco capensis*	Nearctic	0	-0.5259	0.6637	5.97	0.8346	0.0572	0.40
Bucconidae	*Bucco macrodactylus*	Afrotropics	0	-0.0341	-	3.20	0.7946	0.2193	7.40
Bucconidae	*Bucco noanamae*	IndoMalay	0	-0.0005	-	2.83	0.7903	0.5667	3.32
Bucconidae	*Bucco tamatia*	Nearctic	0	-0.4663	10.1220	3.90	0.8723	0.3649	52.31
Bucconidae	*Chelidoptera tenebrosa*	Australasia	0	0.1021	5.5951	2.80	0.7323	0.1824	17.85
Bucconidae	*Hapaloptila castanea*	Neotropics	0	-0.4407	3.1651	1.95	0.7374	0.5028	59.30
Bucconidae	*Hypnelus ruficollis*	Neotropics	0	-0.3510	4.8892	3.84	0.7833	0.4427	10.15
Bucconidae	*Malacoptila fulvogularis*	Australasia	0	0.2024	-	2.48	0.6302	0.2423	87.23
Bucconidae	*Malacoptila fusca*	Palearctic	0	-1.0094	-	5.61	0.8408	0.1106	3.12
Bucconidae	*Malacoptila mystacalis*	Australasia	1	9.8099	1.8883	2.83	0.6876	0.0814	25.77
Bucconidae	*Malacoptila panamensis*	Australasia	0	-0.1220	-	2.80	0.7734	0.4897	43.89
Bucconidae	*Malacoptila rufa*	Australasia	0	-0.0504	-	2.81	0.7452	0.1552	23.83
Bucconidae	*Malacoptila rufa*	Australasia	0	-0.0504	-	2.81	0.7452	-	-
Bucconidae	*Malacoptila semicincta*	Neotropics	0	-0.1929	5.9301	2.99	0.6906	0.1896	56.36
Bucconidae	*Malacoptila striata*	Neotropics	0	-0.0502	-	4.19	0.8070	-	-
Bucconidae	*Malacoptila striata*	Neotropics	0	-0.0502	-	4.19	0.8070	0.5598	4.66
Bucconidae	*Micromonacha lanceolata*	Afrotropics	0	-0.0151	-	3.27	0.8153	0.2957	14.39
Bucconidae	*Monasa atra*	Neotropics	0	-0.4686	4.2279	3.12	0.7828	0.4449	29.63
Bucconidae	*Monasa flavirostris*	Neotropics	0	0.1082	13.1911	3.60	0.7659	0.3561	66.19
Bucconidae	*Monasa morphoeus*	IndoMalay	0	-0.0237	5.2184	3.18	0.7809	0.4910	57.06
Bucconidae	*Monasa nigrifrons*	Afrotropics	0	-0.1260	15.1103	3.73	0.8309	0.7433	75.19
Bucconidae	*Nonnula amaurocephala*	Afrotropics	0	-0.0859	8.4166	3.55	0.8386	0.5771	37.71
Bucconidae	*Nonnula brunnea*	Afrotropics	0	0.0294	-	3.34	0.7828	0.5501	46.30
Bucconidae	*Nonnula frontalis*	Afrotropics	1	-0.3872	0.0000	3.80	0.7842	0.3287	52.60
Bucconidae	*Nonnula rubecula*	Afrotropics	0	-0.1368	-	3.99	0.8096	0.1394	5.78
Bucconidae	*Nonnula ruficapilla*	Neotropics	0	-0.7681	-	2.10	0.7466	0.3433	89.38
Bucconidae	*Nonnula sclateri*	Neotropics	0	17.2658	4.8318	3.38	0.6598	0.3556	73.78
Bucconidae	*Notharchus hyperrhynchus*	Neotropics	0	0.0225	9.2080	3.33	0.7272	0.3101	74.54
Bucconidae	*Notharchus macrorhynchos*	Neotropics	0	-23.3005	3.1009	3.85	0.6804	0.3568	59.93
Bucconidae	*Notharchus ordii*	Australasia	1	-0.3200	2.1342	2.50	0.5788	0.0000	45.57
Bucconidae	*Notharchus pectoralis*	Afrotropics	0	-0.2798	1.8484	3.21	0.6948	0.4604	60.94
Bucconidae	*Notharchus swainsoni*	Afrotropics	0	-0.0595	9.3605	3.18	0.7586	0.4212	71.00
Bucconidae	*Notharchus tectus*	Afrotropics	0	-3.6114	-	3.44	0.7281	0.4758	83.33
Bucconidae	*Nystalus chacuru*	Australasia	0	-0.0716	-	2.78	0.7654	0.2192	22.76
Bucconidae	*Nystalus maculatus*	Australasia	0	-0.1450	-	2.50	0.6716	0.2680	24.42
Bucconidae	*Nystalus radiatus*	Palearctic	1	0.0000	4.0112	3.37	0.6120	0.8333	90.00
Bucconidae	*Nystalus striolatus*	IndoMalay	0	0.0141	8.3371	2.83	0.7621	0.5885	6.01
Bucerotidae	*Aceros cassidix*	Australasia	0	-0.0298	-	2.71	0.7185	0.5623	60.71
Bucerotidae	*Aceros comatus*	IndoMalay	0	0.0311	-	2.85	0.7870	0.5501	15.22
Bucerotidae	*Aceros corrugatus*	IndoMalay	0	0.0522	0.1862	2.83	0.7824	0.4462	3.49
Bucerotidae	*Aceros everetti*	Australasia	1	0.6186	3.6040	2.60	0.6120	0.4082	18.43
Bucerotidae	*Aceros leucocephalus*	IndoMalay	0	-0.1187	3.5278	2.63	0.6961	0.8096	10.51
Bucerotidae	*Aceros nipalensis*	IndoMalay	1	-0.1198	8.8463	3.34	0.7563	0.4146	38.58
Bucerotidae	*Aceros plicatus*	Australasia	0	-0.0588	3.2401	2.74	0.7736	0.2798	17.33
Bucerotidae	*Aceros subruficollis*	IndoMalay	1	0.0871	2.8291	3.01	0.7172	0.1448	74.78
Bucerotidae	*Aceros undulatus*	IndoMalay	0	-0.0882	-	2.97	0.8145	0.3880	58.22
Bucerotidae	*Aceros waldeni*	IndoMalay	1	-0.0121	4.5066	2.63	0.6478	0.3503	71.22
Bucerotidae	*Anorrhinus austeni*	IndoMalay	0	-0.1198	5.5511	3.10	0.7946	0.4273	39.67
Bucerotidae	*Anorrhinus galeritus*	IndoMalay	0	-0.0033	-	2.84	0.7885	0.4744	5.71
Bucerotidae	*Anorrhinus tickelli*	IndoMalay	0	0.1815	3.8257	3.06	0.7245	0.2827	83.28
Bucerotidae	*Anthracoceros albirostris*	IndoMalay	0	-0.0765	1.6655	3.06	0.8242	0.3718	47.59
Bucerotidae	*Anthracoceros coronatus*	IndoMalay	0	-0.0197	-	3.23	0.7739	0.4796	64.65
Bucerotidae	*Anthracoceros malayanus*	IndoMalay	0	0.0627	0.3975	2.82	0.7797	0.4472	3.65
Bucerotidae	*Anthracoceros marchei*	IndoMalay	1	0.0591	1.8317	2.52	0.5985	0.0024	45.41
Bucerotidae	*Buceros bicornis*	Afrotropics	0	-0.4223	-	3.60	0.8927	0.2931	4.20
Bucerotidae	*Buceros hydrocorax*	Palearctic	0	-0.5743	6.8169	4.13	0.8408	0.1811	6.58
Bucerotidae	*Buceros rhinoceros*	Afrotropics	0	0.0547	-	3.26	0.8174	0.2732	11.29
Bucerotidae	*Bycanistes albotibialis*	Neotropics	0	-0.0731	-	3.35	0.7378	0.1218	33.03
Bucerotidae	*Bycanistes brevis*	Neotropics	0	-0.0243	1.1984	3.65	0.8037	0.2127	29.46
Bucerotidae	*Bycanistes bucinator*	Neotropics	0	-0.3164	-	2.55	0.6740	0.1943	13.19
Bucerotidae	*Bycanistes cylindricus*	Neotropics	0	-0.2833	-	3.69	0.8476	0.7166	58.32
Bucerotidae	*Bycanistes fistulator*	Neotropics	0	-0.1983	-	3.78	0.8538	0.4578	43.35
Bucerotidae	*Bycanistes subcylindricus*	Neotropics	1	31.4556	2.8091	3.79	0.6598	0.2605	50.52
Bucerotidae	*Ceratogymna atrata*	Australasia	0	-2.0476	-	2.46	0.5788	0.0000	0.00
Bucerotidae	*Ceratogymna elata*	Afrotropics	0	0.1408	-	3.33	0.8164	0.3613	2.48
Bucerotidae	*Ocyceros birostris*	Neotropics	0	0.0723	-	3.24	0.7348	0.7509	57.60
Bucerotidae	*Ocyceros gingalensis*	Neotropics	0	-0.4192	-	3.31	0.7792	0.3554	19.30
Bucerotidae	*Ocyceros griseus*	Neotropics	0	-0.0326	-	3.96	0.8301	0.5624	20.14
Bucerotidae	*Penelopides affinis*	Afrotropics	0	-13.0849	0.0000	3.65	0.7382	0.6877	34.27
Bucerotidae	*Penelopides affinis*	Afrotropics	0	-13.0849	0.0000	3.65	0.7382	-	-
Bucerotidae	*Penelopides exarhatus*	Afrotropics	0	-0.2779	-	3.87	0.8204	0.7875	18.83
Bucerotidae	*Penelopides manillae*	Nearctic	0	-0.4348	-	3.80	0.8686	0.3410	54.74

Family	Species	Realm	Threat	Latitude	Elevation	Anomaly	Size	Shape	Orientation
Bucerotidae	*Penelopides mindorensis*	Afrotropics	0	0.0997	-	3.34	0.7744	0.5213	66.42
Bucerotidae	*Penelopides panini*	Afrotropics	0	-0.0892	2.0713	3.59	0.7300	0.3969	66.63
Bucerotidae	*Penelopides samarensis*	IndoMalay	0	-0.0629	-	3.03	0.7893	0.5707	48.28
Bucerotidae	*Rhinoplax vigil*	Neotropics	1	-0.3193	1.2990	3.07	0.6370	0.3326	44.28
Bucerotidae	*Tockus alboterminatus*	Neotropics	0	0.0655	-	4.10	0.7901	0.4765	25.83
Bucerotidae	*Tockus bradfieldi*	Neotropics	0	-0.2443	5.1441	3.13	0.7747	0.2791	29.51
Bucerotidae	*Tockus camurus*	Neotropics	1	-91.0055	3.6222	4.00	0.6370	0.4335	50.05
Bucerotidae	*Tockus deckeni*	Neotropics	1	0.1085	1.6669	3.20	0.6631	0.4387	59.29
Bucerotidae	*Tockus erythrorhynchus*	Neotropics	0	-0.0488	-	4.03	0.8046	0.4941	0.52
Bucerotidae	*Tockus fasciatus*	Neotropics	0	-0.3205	-	2.54	0.6562	0.2988	4.68
Bucerotidae	*Tockus flavirostris*	Neotropics	0	-0.3043	-	2.53	0.6804	0.1310	24.21
Bucerotidae	*Tockus hartlaubi*	Palearctic	0	-0.8477	2.7647	3.15	0.7469	0.4681	26.55
Bucerotidae	*Tockus hemprichii*	Afrotropics	0	-9.9330	3.1112	3.40	0.6986	0.1821	70.41
Bucerotidae	*Tockus jacksoni*	IndoMalay	0	-0.0267	3.0666	2.64	0.7314	0.2289	67.63
Bucerotidae	*Tockus leucomelas*	IndoMalay	0	0.2384	-	2.52	0.5985	0.0024	45.41
Bucerotidae	*Tockus monteiri*	Afrotropics	0	-0.0790	-	3.18	0.7614	0.3874	70.15
Bucerotidae	*Tockus nasutus*	IndoMalay	0	-0.4098	-	3.43	0.8488	0.7067	4.42
Bucerotidae	*Tockus pallidirostris*	Afrotropics	0	0.0045	-	3.32	0.8104	0.7267	1.36
Bucerotidae	*Tropicranus albocristatus*	Australasia	0	-0.1337	-	2.62	0.7344	0.1588	24.65
Bucorvidae	*Bucorvus abyssinicus*	Neotropics	0	0.0597	-	4.02	0.8299	0.6275	10.42
Burhinidae	*Burhinus bistriatus*	Palearctic	0	-0.6221	5.4701	4.85	0.8679	0.0620	0.05
Burhinidae	*Burhinus capensis*	Nearctic	0	-0.7504	10.9988	3.69	0.8080	0.2092	7.78
Burhinidae	*Burhinus grallarius*	IndoMalay	0	-0.0708	4.7257	3.09	0.8135	0.6716	45.06
Burhinidae	*Burhinus oedicnemus*	IndoMalay	0	-0.0212	5.2512	2.64	0.7256	0.2113	67.65
Burhinidae	*Burhinus senegalensis*	IndoMalay	0	-0.0078	2.7113	2.83	0.7903	0.5653	3.66
Burhinidae	*Burhinus superciliaris*	Afrotropics	0	0.0273	-	3.49	0.8267	0.1609	6.19
Burhinidae	*Burhinus vermiculatus*	Afrotropics	1	-0.1055	13.9311	3.62	0.8241	0.7983	41.04
Burhinidae	*Esacus giganteus*	Afrotropics	0	-0.9839	2.5089	3.77	0.8011	0.5376	10.72
Burhinidae	*Esacus recurvirostris*	Afrotropics	0	-0.4902	-	3.80	0.7778	0.5770	42.84
Callaeatidae	*Callaeas cinereus*	Nearctic	0	-0.4994	5.0142	6.04	0.8104	0.0251	0.45
Callaeatidae	*Philesturnus carunculatus*	Neotropics	0	-0.0325	-	2.46	0.6876	0.3855	3.42
Campephagidae	*Campephaga flava*	Australasia	0	-0.6068	-	3.09	0.7840	0.5695	68.89
Campephagidae	*Campephaga lobata*	Australasia	0	-0.5586	-	3.32	0.7724	0.5253	78.64
Campephagidae	*Campephaga oriolina*	Australasia	1	-0.9956	0.0000	2.84	0.6906	0.5463	56.32
Campephagidae	*Campephaga petiti*	Neotropics	1	-0.6184	3.0785	2.72	0.6221	0.2052	16.70
Campephagidae	*Campochaera sloetii*	Neotropics	0	-0.3319	-	3.39	0.8000	0.4522	37.60
Campephagidae	*Coracina abbotti*	Neotropics	0	-0.1393	15.7388	3.85	0.7840	0.2335	39.26
Campephagidae	*Coracina analis*	Nearctic	0	-0.5675	11.0378	3.97	0.8377	0.3756	46.94
Campephagidae	*Coracina atriceps*	Nearctic	0	-0.7570	5.3059	4.28	0.8230	0.6170	5.77
Campephagidae	*Coracina bicolor*	Afrotropics	0	-0.0757	-	3.18	0.7614	0.3874	70.15
Campephagidae	*Coracina boyeri*	IndoMalay	1	-0.2378	-	2.66	0.5985	0.2252	62.36
Campephagidae	*Coracina caeruleogrisea*	IndoMalay	0	-0.0718	-	2.65	0.7096	0.2520	74.77
Campephagidae	*Coracina caesia*	IndoMalay	0	-0.0818	1.5632	3.07	0.8249	0.4863	39.04
Campephagidae	*Coracina caledonica*	IndoMalay	0	0.2360	-	2.52	0.5985	-	-
Campephagidae	*Coracina caledonica*	IndoMalay	0	0.2360	-	2.52	0.5985	0.0024	45.41
Campephagidae	*Coracina ceramensis*	IndoMalay	0	-0.2290	-	3.21	0.8437	0.6423	14.81
Campephagidae	*Coracina cinerea*	Afrotropics	0	0.0521	3.9580	3.55	0.8384	0.2329	2.77
Campephagidae	*Coracina coerulescens*	IndoMalay	0	-0.1725	-	3.53	0.8369	0.3380	11.69
Campephagidae	*Coracina dispar*	Afrotropics	0	-0.1527	-	3.67	0.8430	0.4837	55.33
Campephagidae	*Coracina dohertyi*	Afrotropics	0	0.0176	-	3.44	0.8045	0.1278	9.23
Campephagidae	*Coracina fimbriata*	Palearctic	0	-0.7746	6.1507	4.33	0.8488	0.3543	1.29
Campephagidae	*Coracina fortis*	Afrotropics	0	-0.0754	5.6476	3.64	0.8469	0.7008	30.22
Campephagidae	*Coracina graueri*	Afrotropics	0	-0.2068	5.9253	3.62	0.8134	0.7085	6.22
Campephagidae	*Coracina holopolia*	Australasia	0	-0.2844	3.7176	2.71	0.7185	0.5623	60.71
Campephagidae	*Coracina incerta*	Australasia	0	0.8737	-	2.73	0.7008	0.5857	62.84
Campephagidae	*Coracina javensis*	Australasia	0	-0.7511	-	2.58	0.6221	0.1501	33.56
Campephagidae	*Coracina larvata*	Australasia	0	-0.7511	-	2.48	0.6598	0.2542	65.35
Campephagidae	*Coracina leucopygia*	Afrotropics	0	-0.0135	-	3.20	0.8002	0.2277	13.18
Campephagidae	*Coracina lineata*	Australasia	0	-0.0701	1.5907	2.70	0.7165	0.5531	61.27
Campephagidae	*Coracina longicauda*	Australasia	0	-0.0952	-	2.77	0.7692	0.3062	24.36
Campephagidae	*Coracina macei*	Australasia	0	-0.0634	-	2.79	0.7629	0.2201	22.84
Campephagidae	*Coracina maxima*	Afrotropics	0	-0.1704	-	3.33	0.7885	0.5027	79.87
Campephagidae	*Coracina mcgregori*	Australasia	0	-0.2419	-	2.53	0.6598	0.1976	58.92
Campephagidae	*Coracina melanoptera*	Australasia	0	0.0799	-	2.48	0.6523	0.3861	3.09
Campephagidae	*Coracina melas*	Afrotropics	0	-0.0869	-	3.15	0.7554	0.3915	69.76
Campephagidae	*Coracina melaschistos*	IndoMalay	0	-0.0553	2.4052	2.66	0.7030	0.3125	72.88
Campephagidae	*Coracina montana*	Australasia	0	-0.2675	6.8453	2.62	0.6631	0.3500	5.24
Campephagidae	*Coracina morio*	IndoMalay	0	-0.0507	-	2.83	0.7904	0.5985	10.91
Campephagidae	*Coracina novaehollandiae*	Australasia	0	-1.0688	5.8157	2.49	0.5788	0.0000	0.00
Campephagidae	*Coracina ostenta*	Afrotropics	0	-1.7614	4.4673	3.40	0.7008	0.2091	70.08
Campephagidae	*Coracina papuensis*	Australasia	0	-0.0652	3.0826	2.48	0.6120	0.0400	36.10
Campephagidae	*Coracina parvula*	Australasia	0	-0.0831	-	2.76	0.7600	-	-
Campephagidae	*Coracina parvula*	Australasia	0	-0.0831	-	2.76	0.7600	0.1990	23.26
Campephagidae	*Coracina pectoralis*	IndoMalay	0	-0.3984	-	2.71	0.7104	0.1572	11.52

Family	Species	Realm	Threat	Latitude	Elevation	Anomaly	Size	Shape	Orientation
Campephagidae	*Coracina personata*	IndoMalay	0	0.0029	-	2.83	0.7659	0.5720	6.73
Campephagidae	*Coracina polioptera*	Australasia	0	-0.6094	-	2.71	0.7185	0.5623	60.71
Campephagidae	*Coracina schistacea*	Australasia	0	-0.3091	-	2.90	0.7739	0.3718	64.53
Campephagidae	*Coracina schisticeps*	Australasia	0	-0.2726	12.9815	2.83	0.7352	0.1806	23.18
Campephagidae	*Coracina striata*	IndoMalay	0	-0.1704	-	3.25	0.8312	0.4183	0.16
Campephagidae	*Coracina temminckii*	Australasia	0	-0.5795	-	3.36	0.8339	0.5297	3.98
Campephagidae	*Coracina tenuirostris*	IndoMalay	0	-0.1154	5.2212	2.63	0.6804	0.7066	72.16
Campephagidae	*Hemipus hirundinaceus*	Neotropics	0	1.6837	11.7557	3.53	0.7245	0.3281	56.13
Campephagidae	*Hemipus picatus*	Neotropics	0	0.0301	1.9081	4.00	0.8082	0.7709	18.29
Campephagidae	*Lalage atrovirens*	Afrotropics	0	0.0550	9.2513	3.33	0.7419	-	-
Campephagidae	*Lalage atrovirens*	Afrotropics	0	0.0550	9.2513	3.33	0.7419	0.2100	19.27
Campephagidae	*Lalage aurea*	Afrotropics	0	-0.0191	-	3.48	0.8534	0.5003	33.34
Campephagidae	*Lalage leucomela*	IndoMalay	0	0.0313	1.9487	2.81	0.7348	0.4581	1.52
Campephagidae	*Lalage leucopyga*	Palearctic	1	-0.8143	-	4.30	0.8115	0.4166	34.08
Campephagidae	*Lalage leucopygialis*	IndoMalay	0	-0.6631	4.3671	3.30	0.8107	0.4434	8.44
Campephagidae	*Lalage maculosa*	IndoMalay	0	-0.1087	-	2.93	0.8096	0.4251	55.96
Campephagidae	*Lalage melanoleuca*	IndoMalay	0	-0.1099	-	3.30	0.8300	0.4233	10.95
Campephagidae	*Lalage moesta*	Neotropics	0	-0.0152	7.1520	3.65	0.7735	0.3767	68.15
Campephagidae	*Lalage nigra*	Neotropics	0	-0.3726	10.4668	3.23	0.7989	0.5725	88.19
Campephagidae	*Lalage sueurii*	Neotropics	0	-0.4742	7.1920	2.96	0.7946	0.2795	43.59
Campephagidae	*Lalage tricolor*	Neotropics	0	-16.4643	3.0408	3.80	0.6842	0.6196	82.41
Campephagidae	*Pericrocotus brevirostris*	Afrotropics	0	0.1508	20.8194	3.54	0.7761	0.8249	35.36
Campephagidae	*Pericrocotus cantonensis*	Nearctic	0	-0.8312	11.3642	4.35	0.8552	0.3827	10.88
Campephagidae	*Pericrocotus cinnamomeus*	Nearctic	0	-0.8553	-	4.22	0.8395	0.2928	7.77
Campephagidae	*Pericrocotus divaricatus*	Nearctic	0	-0.5261	-	4.13	0.8123	0.6891	3.37
Campephagidae	*Pericrocotus erythropygius*	Nearctic	0	-0.4716	7.9093	4.10	0.8302	0.6884	1.48
Campephagidae	*Pericrocotus ethologus*	Nearctic	0	-0.8997	4.6391	4.14	0.7937	0.4951	12.98
Campephagidae	*Pericrocotus flammeus*	Nearctic	0	-0.8406	-	4.29	0.8329	0.4893	8.49
Campephagidae	*Pericrocotus igneus*	Neotropics	0	-0.1757	-	3.58	0.7261	0.1933	21.13
Campephagidae	*Pericrocotus lansbergei*	Neotropics	0	-1.3646	3.2827	3.55	0.6302	0.2212	7.79
Campephagidae	*Pericrocotus miniatus*	Nearctic	0	-0.2026	-	3.93	0.7927	0.4366	45.27
Campephagidae	*Pericrocotus roseus*	Neotropics	0	-0.1647	5.2279	2.47	0.7455	0.1495	82.71
Campephagidae	*Pericrocotus solaris*	Neotropics	1	0.5156	8.1831	2.47	0.5985	0.0000	0.00
Campephagidae	*Pericrocotus tegimae*	Neotropics	0	-0.1867	2.4827	3.81	0.8540	0.5468	39.07
Campephagidae	*Tephrodornis gularis*	Neotropics	0	-0.1757	-	3.95	0.8170	0.4767	5.64
Campephagidae	*Tephrodornis pondicerianus*	Neotropics	1	-0.1806	1.9084	3.20	0.6631	0.4008	72.80
Caprimulgidae	*Caprimulgus aegyptius*	Neotropics	0	-0.1930	-	3.50	0.7696	0.2624	30.23
Caprimulgidae	*Caprimulgus affinis*	Afrotropics	0	-0.2049	6.1419	3.03	0.7165	0.2262	73.91
Caprimulgidae	*Caprimulgus affinis*	Afrotropics	0	-0.2049	6.1419	3.03	0.7165	-	-
Caprimulgidae	*Caprimulgus anthonyi*	Afrotropics	0	-0.0747	-	3.19	0.7969	0.2232	11.70
Caprimulgidae	*Caprimulgus arizonae*	Neotropics	0	0.1224	3.8451	4.02	0.8152	0.7379	65.48
Caprimulgidae	*Caprimulgus asiaticus*	Neotropics	0	0.1341	-	3.85	0.7864	0.5619	8.77
Caprimulgidae	*Caprimulgus atripennis*	Neotropics	0	-0.0235	-	4.29	0.7158	0.3779	27.37
Caprimulgidae	*Caprimulgus badius*	Neotropics	0	-0.0893	2.3806	4.23	0.7874	0.6342	23.88
Caprimulgidae	*Caprimulgus batesi*	Neotropics	1	11.7934	6.0767	3.31	0.6740	0.5384	81.26
Caprimulgidae	*Caprimulgus binotatus*	Neotropics	0	-0.3365	2.2825	3.07	0.7217	0.8209	51.39
Caprimulgidae	*Caprimulgus carolinensis*	Neotropics	0	0.0177	4.1054	4.01	0.8231	0.5972	32.11
Caprimulgidae	*Caprimulgus cayennensis*	Neotropics	1	-0.1141	1.2023	2.92	0.6891	0.2070	69.05
Caprimulgidae	*Caprimulgus celebensis*	Neotropics	0	0.7171	-	3.06	0.6961	0.3036	73.69
Caprimulgidae	*Caprimulgus clarus*	Palearctic	0	-0.7838	-	4.10	0.8142	0.1634	7.28
Caprimulgidae	*Caprimulgus climacurus*	IndoMalay	0	-0.0956	5.3656	3.21	0.8286	0.4610	28.28
Caprimulgidae	*Caprimulgus concretus*	Neotropics	0	-0.1449	2.2100	3.09	0.6948	0.2171	84.52
Caprimulgidae	*Caprimulgus cubanensis*	Nearctic	0	-0.2369	9.4272	3.95	0.7904	0.2785	44.11
Caprimulgidae	*Caprimulgus donaldsoni*	IndoMalay	0	-0.0875	4.1959	3.33	0.8217	0.4573	16.66
Caprimulgidae	*Caprimulgus ekmani*	IndoMalay	0	0.0177	5.7988	3.10	0.7634	0.4025	60.37
Caprimulgidae	*Caprimulgus enarratus*	Neotropics	0	-0.2250	-	2.83	0.7087	0.7142	48.54
Caprimulgidae	*Caprimulgus europaeus*	Afrotropics	0	-0.0157	-	3.30	0.7999	0.4629	3.43
Caprimulgidae	*Caprimulgus eximius*	Afrotropics	0	0.0365	-	3.19	0.7669	0.1013	12.74
Caprimulgidae	*Caprimulgus fossii*	Nearctic	0	-0.7836	6.9422	4.00	0.8003	0.5106	3.10
Caprimulgidae	*Caprimulgus fraenatus*	Neotropics	0	-0.0230	6.4545	3.89	0.7949	0.2855	9.28
Caprimulgidae	*Caprimulgus heterurus*	Australasia	0	0.1724	-	2.63	0.6784	0.6940	75.60
Caprimulgidae	*Caprimulgus hirundinaceus*	Palearctic	0	4.8721	-	4.22	0.7296	0.3650	2.02
Caprimulgidae	*Caprimulgus indicus*	Afrotropics	0	-0.0206	7.7622	3.27	0.7944	0.6920	66.16
Caprimulgidae	*Caprimulgus inornatus*	Afrotropics	0	0.1626	-	3.47	0.8414	0.4496	9.71
Caprimulgidae	*Caprimulgus longirostris*	IndoMalay	1	0.0718	1.0257	2.84	0.7829	0.4033	4.44
Caprimulgidae	*Caprimulgus macrurus*	Neotropics	0	-0.3017	3.2460	2.50	0.7185	0.1208	19.14
Caprimulgidae	*Caprimulgus maculicaudus*	Afrotropics	0	-0.0267	6.6319	3.28	0.7771	0.3979	48.72
Caprimulgidae	*Caprimulgus maculosus*	Neotropics	0	0.1926	3.9548	2.47	0.6716	0.4883	1.64
Caprimulgidae	*Caprimulgus madagascariensis*	Afrotropics	0	-0.1670	7.2789	3.08	0.7348	0.2043	75.10
Caprimulgidae	*Caprimulgus mahrattensis*	Palearctic	0	-0.7829	7.2883	4.36	0.8643	0.2673	0.08
Caprimulgidae	*Caprimulgus manillensis*	Afrotropics	0	0.1685	3.0366	3.78	0.8048	0.1111	3.21
Caprimulgidae	*Caprimulgus meesi*	Afrotropics	0	-0.0673	8.5491	3.59	0.8357	0.8793	34.78
Caprimulgidae	*Caprimulgus natalensis*	Afrotropics	0	0.0539	14.8054	3.33	0.7695	0.3690	72.57
Caprimulgidae	*Caprimulgus nigrescens*	Neotropics	0	0.0788	1.7389	4.01	0.7524	0.4112	6.30

Family	Species	Realm	Threat	Latitude	Elevation	Anomaly	Size	Shape	Orientation
Caprimulgidae	*Caprimulgus nigriscapularis*	Neotropics	0	-0.1710	-	3.59	0.7705	0.7863	60.61
Caprimulgidae	*Caprimulgus noctitherus*	IndoMalay	0	-0.6055	8.5812	3.52	0.8449	0.3099	23.27
Caprimulgidae	*Caprimulgus nubicus*	Afrotropics	0	0.0817	7.6226	3.65	0.8173	0.1558	7.32
Caprimulgidae	*Caprimulgus parvulus*	Neotropics	0	-0.3057	11.2567	3.14	0.8273	0.6044	88.47
Caprimulgidae	*Caprimulgus pectoralis*	IndoMalay	0	-0.1517	5.9121	3.02	0.8319	0.2478	34.25
Caprimulgidae	*Caprimulgus poliocephalus*	Neotropics	0	-0.1261	1.9444	3.89	0.8183	0.3849	42.23
Caprimulgidae	*Caprimulgus prigoginei*	Neotropics	0	0.2425	-	3.25	0.5788	0.0000	90.00
Caprimulgidae	*Caprimulgus pulchellus*	Afrotropics	0	-0.0693	-	3.18	0.7614	0.3874	70.15
Caprimulgidae	*Caprimulgus ridgwayi*	Palearctic	0	-0.3043	1.2991	4.11	0.7842	0.6948	12.78
Caprimulgidae	*Caprimulgus ruficollis*	IndoMalay	0	-0.0027	4.8632	2.64	0.7327	0.2318	67.52
Caprimulgidae	*Caprimulgus rufigena*	Australasia	0	-0.3156	3.1611	2.61	0.6428	0.4243	35.42
Caprimulgidae	*Caprimulgus rufus*	Afrotropics	0	-0.0032	9.7591	3.50	0.8111	0.6288	35.09
Caprimulgidae	*Caprimulgus ruwenzorii*	Neotropics	0	0.2157	3.8603	4.01	0.8237	0.6064	3.23
Caprimulgidae	*Caprimulgus salvini*	Afrotropics	0	-0.1255	8.0290	3.28	0.7867	0.2464	11.00
Caprimulgidae	*Caprimulgus saturatus*	Neotropics	1	0.2354	0.7080	2.41	0.5788	0.0000	0.00
Caprimulgidae	*Caprimulgus sericocaudatus*	Afrotropics	0	-0.2470	3.4805	3.38	0.7767	0.5397	88.43
Caprimulgidae	*Caprimulgus solala*	Neotropics	0	-0.1785	3.5804	3.62	0.8358	0.5873	39.58
Caprimulgidae	*Caprimulgus stellatus*	Afrotropics	0	-0.2697	7.2401	3.69	0.8274	0.7986	43.16
Caprimulgidae	*Caprimulgus tristigma*	Afrotropics	0	0.0667	12.9253	3.56	0.7802	-	-
Caprimulgidae	*Caprimulgus tristigma*	Afrotropics	0	0.0667	12.9253	3.56	0.7802	0.2739	69.67
Caprimulgidae	*Caprimulgus vociferus*	Afrotropics	1	0.0000	3.0607	3.41	0.5985	0.5774	45.00
Caprimulgidae	*Caprimulgus whitelyi*	IndoMalay	0	-0.1527	7.0623	2.78	0.7069	0.1483	29.91
Caprimulgidae	*Chordeiles acutipennis*	Neotropics	0	-0.1627	-	3.52	0.7480	0.4818	56.97
Caprimulgidae	*Chordeiles gundlachii*	Neotropics	0	-0.3432	11.7354	3.48	0.8312	0.4703	41.18
Caprimulgidae	*Chordeiles minor*	Neotropics	0	0.7898	3.2046	2.41	0.5788	0.0000	0.00
Caprimulgidae	*Chordeiles nacunda*	Neotropics	0	0.0867	8.6300	3.10	0.7296	0.2322	64.51
Caprimulgidae	*Chordeiles pusillus*	Neotropics	0	-0.0204	8.5801	4.06	0.8327	0.9234	29.34
Caprimulgidae	*Chordeiles rupestris*	Neotropics	0	0.3934	1.5315	3.77	0.6370	0.5773	44.43
Caprimulgidae	*Eleothreptus anomalus*	Neotropics	0	0.4801	4.8283	4.37	0.6763	0.2734	19.72
Caprimulgidae	*Eleothreptus candicans*	Neotropics	0	0.2697	7.4019	2.47	0.6763	0.1869	6.45
Caprimulgidae	*Eurostopodus archboldi*	Afrotropics	0	0.0371	-	3.60	0.7841	0.5334	38.51
Caprimulgidae	*Eurostopodus argus*	Afrotropics	0	-0.0084	-	3.44	0.8355	0.3982	24.46
Caprimulgidae	*Eurostopodus diabolicus*	Afrotropics	0	7.2003	8.8100	3.19	0.7165	0.7368	77.65
Caprimulgidae	*Eurostopodus macrotis*	Afrotropics	0	-0.0048	7.2907	3.45	0.8305	0.4829	23.05
Caprimulgidae	*Eurostopodus mystacalis*	Afrotropics	0	-0.0884	-	3.12	0.7323	0.3954	80.93
Caprimulgidae	*Eurostopodus papuensis*	Afrotropics	0	-0.2463	-	3.69	0.8195	0.5043	66.38
Caprimulgidae	*Eurostopodus temminckii*	Afrotropics	0	-0.1196	5.9876	3.68	0.7794	0.5174	83.17
Caprimulgidae	*Hydropsalis climacocerca*	Afrotropics	0	0.0272	10.4886	3.49	0.8297	0.2726	7.15
Caprimulgidae	*Hydropsalis torquata*	Afrotropics	0	-0.4681	-	3.66	0.7852	0.5433	28.58
Caprimulgidae	*Lurocalis rufiventris*	Neotropics	0	0.0757	7.1262	3.82	0.7505	0.2780	66.62
Caprimulgidae	*Lurocalis semitorquatus*	Neotropics	0	-0.0258	-	4.01	0.7873	0.3746	56.26
Caprimulgidae	*Macrodipteryx longipennis*	IndoMalay	0	-0.3847	4.8906	2.71	0.7040	0.1292	11.84
Caprimulgidae	*Macropsalis forcipata*	Neotropics	0	0.0131	-	2.41	0.5788	0.0000	0.00
Caprimulgidae	*Nyctidromus albicollis*	Australasia	1	-0.3300	2.7935	2.49	0.6662	0.3153	20.62
Caprimulgidae	*Nyctiphrynus mcleodii*	IndoMalay	0	-0.0003	-	2.64	0.7327	0.2318	67.52
Caprimulgidae	*Nyctiphrynus ocellatus*	Australasia	0	-0.1235	-	2.71	0.7185	0.5623	60.71
Caprimulgidae	*Nyctiphrynus rosenbergi*	Australasia	0	0.6946	3.7932	2.60	0.6120	0.4082	18.43
Caprimulgidae	*Nyctiphrynus yucatanicus*	Australasia	0	-0.1832	-	2.83	0.7782	0.8138	21.18
Caprimulgidae	*Nyctiprogne leucopyga*	IndoMalay	0	-0.4764	3.8729	3.38	0.8467	0.5513	35.66
Caprimulgidae	*Nyctiprogne vielliardi*	Australasia	0	-0.4764	4.8199	2.48	0.6716	0.4515	67.99
Caprimulgidae	*Phalaenoptilus nuttallii*	Australasia	0	-0.6319	-	3.36	0.8342	0.4572	5.93
Caprimulgidae	*Siphonorhis americana*	Neotropics	0	-14.6166	8.5072	3.84	0.7172	0.2103	63.11
Caprimulgidae	*Siphonorhis brewsteri*	Neotropics	0	-0.5613	-0.9353	2.97	0.7158	0.1278	81.17
Caprimulgidae	*Uropsalis lyra*	Neotropics	0	-0.5117	2.1408	3.21	0.6740	0.2651	62.85
Caprimulgidae	*Uropsalis segmentata*	Australasia	0	-0.0936	-	2.71	0.7185	0.5623	60.71
Cardinalidae	*Amaurospiza carrizalensis*	IndoMalay	0	-0.3051	-	3.21	0.8456	0.6471	14.54
Cardinalidae	*Amaurospiza carrizalensis*	IndoMalay	0	-0.3051	-	3.21	0.8456	-	-
Cardinalidae	*Amaurospiza moesta*	Neotropics	0	0.0182	9.0384	3.40	0.7474	0.2099	55.81
Cardinalidae	*Cardinalis cardinalis*	Afrotropics	0	8.7737	7.6340	3.45	0.7040	0.3402	82.09
Cardinalidae	*Cardinalis phoeniceus*	Nearctic	0	-0.1271	1.5318	3.61	0.7165	0.2995	69.01
Cardinalidae	*Cardinalis sinuatus*	Neotropics	0	-0.1467	9.0568	3.08	0.6562	0.3375	33.58
Cardinalidae	*Caryothraustes canadensis*	Palearctic	0	-0.9311	10.4096	4.88	0.8279	0.3674	5.96
Cardinalidae	*Caryothraustes poliogaster*	Palearctic	0	-0.7019	12.8995	3.78	0.7832	0.5201	21.41
Cardinalidae	*Chlorothraupis carmioli*	Neotropics	0	-0.3166	3.8392	2.87	0.6221	0.2738	27.05
Cardinalidae	*Chlorothraupis olivacea*	Neotropics	0	-0.4334	6.0204	3.76	0.7069	0.2608	2.85
Cardinalidae	*Chlorothraupis stolzmanni*	Neotropics	0	-0.0688	-	2.90	0.6876	0.2926	5.72
Cardinalidae	*Cyanocompsa brissonii*	IndoMalay	0	-0.1812	-	3.23	0.8279	0.6206	57.64
Cardinalidae	*Cyanocompsa cyanoides*	IndoMalay	0	0.0108	-	2.67	0.7485	0.2562	87.18
Cardinalidae	*Cyanocompsa parellina*	Neotropics	1	-0.3881	5.4510	3.76	0.7996	0.6602	26.25
Cardinalidae	*Cyanoloxia glaucocaerulea*	Neotropics	0	-0.0986	-	3.47	0.7705	0.3365	20.22
Cardinalidae	*Granatellus pelzelni*	Neotropics	0	-21.2002	3.3734	3.73	0.6859	0.2986	67.67
Cardinalidae	*Granatellus venustus*	Neotropics	0	-18.0332	1.6689	3.61	0.7310	0.6966	38.09
Cardinalidae	*Habia atrimaxillaris*	Neotropics	0	-0.3341	3.9709	3.44	0.8400	0.4711	50.89
Cardinalidae	*Habia cristata*	Afrotropics	0	0.0822	-	3.24	0.7800	0.3682	3.88

Family	Species	Realm	Threat	Latitude	Elevation	Anomaly	Size	Shape	Orientation
Cardinalidae	*Habia fuscicauda*	Afrotropics	0	-0.1163	-	3.36	0.8240	0.4983	27.75
Cardinalidae	*Habia gutturalis*	Neotropics	0	-4.9912	1.1492	3.68	0.7498	0.6035	66.48
Cardinalidae	*Habia rubica*	Afrotropics	0	0.1214	-	3.32	0.8162	0.5091	1.25
Cardinalidae	*Parkerthraustes humeralis*	Australasia	0	-0.0393	-	3.14	0.7689	0.3398	10.87
Cardinalidae	*Periporphyrus erythromelas*	Neotropics	0	0.1626	1.1305	3.56	0.7185	0.1684	8.01
Cardinalidae	*Pheucticus aureoventris*	Neotropics	0	-0.4847	2.9776	3.48	0.6428	0.3140	4.23
Cardinalidae	*Pheucticus chrysogaster*	Neotropics	0	-0.1954	3.8314	3.87	0.7796	0.2576	39.29
Cardinalidae	*Pheucticus chrysopeplus*	Neotropics	0	0.2435	7.2306	3.02	0.6428	0.3087	29.93
Cardinalidae	*Pheucticus ludovicianus*	Australasia	1	-0.6867	-	3.49	0.8113	0.4937	18.27
Cardinalidae	*Pheucticus melanocephalus*	Australasia	0	-0.5675	-	2.68	0.7469	0.3921	0.65
Cardinalidae	*Pheucticus tibialis*	Neotropics	1	-21.1468	4.7810	3.59	0.6370	0.2010	70.08
Cardinalidae	*Piranga bidentata*	Neotropics	0	-0.1341	2.4267	3.92	0.8426	0.7445	23.32
Cardinalidae	*Piranga erythrocephala*	Neotropics	0	-0.1077	-	3.98	0.8350	0.6016	26.72
Cardinalidae	*Piranga flava*	Neotropics	0	0.1823	-	4.04	0.7828	0.6840	17.04
Cardinalidae	*Piranga leucoptera*	Neotropics	0	0.2919	-	3.08	0.7198	0.2685	60.14
Cardinalidae	*Piranga ludoviciana*	Neotropics	0	0.0351	-	3.24	0.7144	0.3187	68.63
Cardinalidae	*Piranga olivacea*	Neotropics	0	-0.2737	-	3.98	0.8030	0.4979	13.84
Cardinalidae	*Piranga roseogularis*	Neotropics	0	0.0508	-	4.17	0.8141	0.5331	4.98
Cardinalidae	*Piranga rubra*	Neotropics	0	0.0612	-	3.98	0.7185	0.4258	61.58
Cardinalidae	*Piranga rubra*	Neotropics	0	0.0612	-	3.98	0.7185	-	-
Cardinalidae	*Piranga rubriceps*	Neotropics	0	-0.0315	0.4833	3.46	0.7205	0.6161	29.81
Cardinalidae	*Rhodothraupis celaeno*	Afrotropics	0	-0.0479	-	3.41	0.8207	0.3528	29.32
Cardinalidae	*Saltator atriceps*	Palearctic	0	-0.6635	4.7417	3.65	0.8156	0.4476	5.76
Cardinalidae	*Saltator atricollis*	Palearctic	0	-0.5444	8.1981	4.43	0.8563	0.2561	5.95
Cardinalidae	*Saltator atricollis*	Palearctic	0	-0.5444	8.1981	4.43	0.8563	-	-
Cardinalidae	*Saltator atripennis*	Nearctic	0	-0.7813	7.8899	4.07	0.8372	0.3918	8.75
Cardinalidae	*Saltator aurantiirostris*	IndoMalay	0	-0.0376	-	2.83	0.7883	0.5182	4.14
Cardinalidae	*Saltator cinctus*	Australasia	0	-0.2615	3.4173	2.50	0.6716	0.2680	24.42
Cardinalidae	*Saltator coerulescens*	Australasia	0	-0.2412	3.5571	2.48	0.6221	0.0384	35.15
Cardinalidae	*Saltator fuliginosus*	Australasia	0	-0.0635	8.7445	2.75	0.7657	0.1967	20.30
Cardinalidae	*Saltator grossus*	Palearctic	0	-1.2257	0.0000	4.33	0.7416	0.2571	27.30
Cardinalidae	*Saltator grossus*	Palearctic	0	-1.2257	0.0000	4.33	0.7416	-	-
Cardinalidae	*Saltator maxillosus*	Palearctic	0	-1.0048	8.0119	4.55	0.8018	0.2226	14.67
Cardinalidae	*Saltator maximus*	Palearctic	0	-1.1363	0.0000	4.33	0.7466	0.4069	21.50
Cardinalidae	*Saltator nigriceps*	Palearctic	0	-0.8394	2.1255	4.45	0.8379	0.2430	5.54
Cardinalidae	*Saltator orenocensis*	IndoMalay	0	-0.0982	-	2.67	0.6859	0.4381	87.80
Cardinalidae	*Saltator rufiventris*	IndoMalay	0	0.0095	-	2.62	0.7096	0.5946	66.57
Cardinalidae	*Saltator similis*	IndoMalay	0	-0.0286	-	2.64	0.7310	0.2224	67.87
Cardinalidae	*Saltator striatipectus*	Australasia	0	-0.0530	6.1224	2.81	0.7406	0.1490	23.78
Cardinalidae	*Spiza americana*	Australasia	0	-0.1890	0.0000	2.76	0.7185	0.1700	17.29
Cariamidae	*Cariama cristata*	Neotropics	0	-2.2658	9.8776	3.87	0.7458	0.2273	48.90
Cariamidae	*Chunga burmeisteri*	Australasia	0	-0.2178	4.8033	2.84	0.7371	0.1612	24.07
Casuariidae	*Casuarius bennetti*	Palearctic	0	-0.5321	12.5190	4.24	0.7938	0.3689	3.15
Casuariidae	*Casuarius casuarius*	Palearctic	0	1.1346	8.7191	3.74	0.7595	0.3668	43.93
Casuariidae	*Casuarius unappendiculatus*	Palearctic	0	-0.6606	7.9138	3.47	0.7919	0.6301	15.50
Cathartidae	*Cathartes aura*	Neotropics	0	-0.1118	-	3.85	0.8121	0.3932	32.62
Cathartidae	*Cathartes burrovianus*	Neotropics	0	-0.0334	-	3.44	0.7531	0.2868	32.34
Cathartidae	*Cathartes melambrotus*	Neotropics	0	0.0469	1.7944	3.73	0.8033	0.6885	15.21
Cathartidae	*Coragyps atratus*	IndoMalay	1	-0.0062	2.5691	2.64	0.7277	0.2226	67.15
Cathartidae	*Gymnogyps californianus*	Neotropics	0	-0.0172	2.6456	4.10	0.8224	0.7480	59.61
Cathartidae	*Sarcoramphus papa*	Neotropics	0	0.1438	-	4.20	0.6716	0.3814	58.40
Cathartidae	*Vultur gryphus*	Australasia	0	-0.4900	3.5457	3.37	0.8224	0.5001	40.83
Certhiidae	*Certhia americana*	Neotropics	0	-9.9174	-	4.22	0.7245	0.4988	46.21
Certhiidae	*Certhia brachydactyla*	Neotropics	0	-0.1810	-	4.04	0.7606	0.3329	15.48
Certhiidae	*Certhia discolor*	Neotropics	0	-0.0657	-	4.11	0.8221	0.5416	0.36
Certhiidae	*Certhia familiaris*	Neotropics	0	0.1660	-	3.55	0.7725	0.4664	25.29
Certhiidae	*Certhia himalayana*	Neotropics	0	-0.2319	4.7897	3.22	0.6921	0.2829	75.02
Certhiidae	*Certhia hodgsoni*	Neotropics	0	0.2044	-	3.92	0.7860	0.4476	61.25
Certhiidae	*Certhia manipurensis*	Neotropics	0	-0.0515	4.1955	3.84	0.8171	0.2998	20.61
Certhiidae	*Certhia nipalensis*	Afrotropics	0	40.3719	1.9833	3.70	0.7060	0.4242	9.35
Certhiidae	*Certhia tianquanensis*	Afrotropics	0	-0.1449	-	3.62	0.8404	0.6300	56.62
Certhiidae	*Salpornis spilonotus*	IndoMalay	0	-0.8692	-	2.61	0.6523	0.3873	66.90
Charadriidae	*Anarhynchus frontalis*	Australasia	1	-0.0263	2.5573	2.27	0.6598	0.3294	23.56
Charadriidae	*Charadrius alexandrinus*	Australasia	0	-0.3417	-	2.54	0.6906	0.5603	46.97
Charadriidae	*Charadrius alticola*	Australasia	0	-0.3043	-	2.48	0.6221	0.0384	35.15
Charadriidae	*Charadrius asiaticus*	Australasia	0	0.0035	0.6717	2.75	0.7158	0.1719	20.20
Charadriidae	*Charadrius australis*	Australasia	0	-0.0846	1.0155	2.86	0.7452	0.3399	18.31
Charadriidae	*Charadrius bicinctus*	Neotropics	0	0.4411	7.7072	3.20	0.7158	0.1461	66.17
Charadriidae	*Charadrius collaris*	Neotropics	0	-0.2284	5.5177	3.77	0.6859	0.3287	67.39
Charadriidae	*Charadrius dubius*	Neotropics	0	4.5246	6.2248	4.07	0.7205	0.1571	24.75
Charadriidae	*Charadrius falklandicus*	Neotropics	0	-3.7138	11.6813	3.94	0.7367	0.1609	42.56
Charadriidae	*Charadrius forbesi*	Neotropics	0	0.1171	7.2679	3.83	0.7363	0.2361	52.95
Charadriidae	*Charadrius hiaticula*	Neotropics	0	0.2385	4.8550	3.48	0.7673	0.5118	47.79
Charadriidae	*Charadrius javanicus*	Neotropics	0	-0.0519	1.8533	3.10	0.7399	0.3724	46.11

Family	Species	Realm	Threat	Latitude	Elevation	Anomaly	Size	Shape	Orientation
Charadriidae	*Charadrius leschenaultii*	Nearctic	0	-0.5902	-	3.26	0.7441	0.2222	57.80
Charadriidae	*Charadrius marginatus*	Neotropics	0	-0.1227	12.6767	3.35	0.7588	0.2708	81.47
Charadriidae	*Charadrius melodus*	Neotropics	0	-0.2695	8.7701	3.04	0.6804	0.2128	30.89
Charadriidae	*Charadrius modestus*	Neotropics	0	-0.1971	7.3646	3.52	0.8042	0.4460	50.08
Charadriidae	*Charadrius mongolus*	Neotropics	0	-0.6029	7.3344	2.84	0.7185	0.1847	30.62
Charadriidae	*Charadrius montanus*	Neotropics	0	0.0155	7.5664	3.71	0.7281	0.2061	58.55
Charadriidae	*Charadrius obscurus*	Neotropics	0	0.1798	-	4.05	0.8099	0.5952	14.63
Charadriidae	*Charadrius pallidus*	Neotropics	0	-0.4912	8.6586	2.77	0.7539	0.2052	36.69
Charadriidae	*Charadrius pecuarius*	Neotropics	0	-0.0205	9.0173	3.66	0.6631	0.0893	44.83
Charadriidae	*Charadrius peronii*	Palearctic	0	-0.5407	8.4478	4.05	0.8611	0.1752	3.61
Charadriidae	*Charadrius placidus*	Neotropics	0	-0.3035	9.2124	4.15	0.7697	0.3681	55.69
Charadriidae	*Charadrius ruficapillus*	Palearctic	0	-1.0551	-	4.44	0.8142	0.3122	3.40
Charadriidae	*Charadrius semipalmatus*	Australasia	0	-0.7180	-	3.11	0.7777	0.4047	0.99
Charadriidae	*Charadrius thoracicus*	Australasia	0	-0.2315	0.0000	2.93	0.5788	0.0000	90.00
Charadriidae	*Charadrius tricollaris*	Neotropics	0	-0.2033	2.4131	3.70	0.8570	0.6311	47.60
Charadriidae	*Charadrius veredus*	Palearctic	0	-0.5131	2.3608	4.23	0.8785	0.3824	4.98
Charadriidae	*Charadrius vociferus*	Neotropics	0	-0.5370	-	2.06	0.7774	0.4609	49.55
Charadriidae	*Charadrius wilsonia*	Afrotropics	0	0.0256	-	3.39	0.8364	0.4121	24.00
Charadriidae	*Elseyornis melanops*	Neotropics	0	-0.2139	10.9081	3.40	0.8018	0.3737	27.63
Charadriidae	*Erythrogonys cinctus*	Afrotropics	0	-0.1210	-	3.69	0.8411	0.6646	43.56
Charadriidae	*Eudromias morinellus*	Afrotropics	0	-0.0877	-	3.33	0.7802	0.7231	45.66
Charadriidae	*Oreopholus ruficollis*	IndoMalay	0	-0.1857	-	3.39	0.8034	0.7309	66.49
Charadriidae	*Phegornis mitchellii*	Neotropics	0	0.4854	5.7134	3.62	0.6974	0.4442	48.71
Charadriidae	*Pluvialis apricaria*	Australasia	0	-0.5803	-	2.98	0.7861	0.4567	7.20
Charadriidae	*Pluvialis dominica*	Australasia	0	-0.8542	-	2.69	0.7318	0.5325	7.15
Charadriidae	*Pluvialis fulva*	Australasia	0	0.0502	-	3.38	0.7739	0.4665	5.19
Charadriidae	*Pluvialis squatarola*	IndoMalay	0	-0.0570	2.8472	2.82	0.7913	0.5773	7.45
Charadriidae	*Thinornis rubricollis*	Neotropics	0	-0.2792	7.8410	3.02	0.6740	0.2182	28.75
Charadriidae	*Vanellus albiceps*	Afrotropics	0	-0.2854	-	4.25	0.7913	0.5253	16.44
Charadriidae	*Vanellus armatus*	IndoMalay	0	-0.2792	3.2105	3.81	0.8112	0.3445	22.28
Charadriidae	*Vanellus cayanus*	IndoMalay	0	-0.1281	-	3.56	0.7905	0.2537	15.93
Charadriidae	*Vanellus chilensis*	Palearctic	0	-0.3870	-	3.92	0.8236	0.4051	13.39
Charadriidae	*Vanellus cinereus*	IndoMalay	0	-0.2027	-	3.18	0.7446	0.3121	85.73
Charadriidae	*Vanellus coronatus*	Afrotropics	0	-0.2340	-	3.86	0.7542	0.4931	69.29
Charadriidae	*Vanellus crassirostris*	Afrotropics	0	3.5237	12.4380	3.88	0.7991	0.5619	26.21
Charadriidae	*Vanellus duvaucelii*	Afrotropics	1	19.8862	5.5708	3.21	0.6784	0.5989	83.03
Charadriidae	*Vanellus gregarius*	Afrotropics	0	0.2245	4.6469	3.18	0.7240	0.4304	86.22
Charadriidae	*Vanellus indicus*	Afrotropics	0	-0.1226	-	3.64	0.8326	0.9217	40.83
Charadriidae	*Vanellus leucurus*	Afrotropics	0	0.1331	7.1918	3.67	0.7482	0.7940	50.16
Charadriidae	*Vanellus lugubris*	Afrotropics	0	0.0539	7.0922	3.55	0.7760	0.8971	74.71
Charadriidae	*Vanellus macropterus*	IndoMalay	1	0.5987	1.8402	3.37	0.6716	0.1094	4.07
Charadriidae	*Vanellus malarbaricus*	IndoMalay	0	-0.1573	-	3.47	0.8010	0.6130	89.80
Charadriidae	*Vanellus melanocephalus*	Afrotropics	0	-3.8843	-	4.28	0.7403	0.3345	17.26
Charadriidae	*Vanellus melanopterus*	IndoMalay	0	0.3430	6.6873	3.92	0.6948	0.1530	16.63
Charadriidae	*Vanellus miles*	Afrotropics	0	0.0663	-	3.47	0.8296	0.2091	7.87
Charadriidae	*Vanellus resplendens*	Afrotropics	0	-0.0712	4.0833	3.44	0.8059	0.1738	8.54
Charadriidae	*Vanellus senegallus*	Afrotropics	0	0.0401	8.0865	3.32	0.7969	0.7211	76.13
Charadriidae	*Vanellus spinosus*	IndoMalay	0	0.5027	-	2.76	0.6302	0.6063	90.00
Charadriidae	*Vanellus superciliosus*	Afrotropics	0	0.0866	8.1563	3.36	0.7643	0.6177	68.43
Charadriidae	*Vanellus tectus*	Palearctic	0	-0.3951	11.6541	3.98	0.7899	0.5317	26.63
Charadriidae	*Vanellus tricolor*	Afrotropics	0	0.0747	1.6219	3.20	0.7240	0.4094	62.79
Charadriidae	*Vanellus vanellus*	IndoMalay	0	-0.1414	4.6335	3.48	0.8149	0.9221	27.19
Chionidae	*Chionis minor*	Australasia	0	-0.3115	2.9438	2.50	0.6716	0.2680	24.42
Chionidae	*Pluvianellus socialis*	Neotropics	0	-0.0598	3.0410	3.42	0.7618	0.4691	31.31
Chloropseidae	*Chloropsis aurifrons*	Neotropics	0	0.5286	7.7148	3.05	0.6986	0.0904	66.69
Chloropseidae	*Chloropsis cochinchinensis*	Afrotropics	0	0.0975	-	3.27	0.7831	0.3905	87.32
Chloropseidae	*Chloropsis cyanopogon*	Afrotropics	0	-0.0034	-	3.45	0.8349	0.4341	26.49
Chloropseidae	*Chloropsis flavipennis*	Afrotropics	0	-0.1549	-	3.62	0.8223	0.6818	21.12
Chloropseidae	*Chloropsis hardwickii*	Afrotropics	0	6.6259	-	3.36	0.7385	0.6913	63.69
Chloropseidae	*Chloropsis jerdoni*	Afrotropics	1	0.0000	3.0365	3.43	0.5788	0.0000	90.00
Chloropseidae	*Chloropsis kinabaluensis*	Afrotropics	0	-0.0190	-	3.28	0.8207	0.2881	17.37
Chloropseidae	*Chloropsis media*	Afrotropics	1	4.7378	6.6639	3.41	0.7327	0.5703	67.53
Chloropseidae	*Chloropsis palawanensis*	Afrotropics	0	-0.1147	-	3.50	0.8248	0.8038	65.68
Chloropseidae	*Chloropsis sonnerati*	Afrotropics	0	0.1176	11.2806	3.31	0.7560	0.7258	73.52
Chloropseidae	*Chloropsis venusta*	Neotropics	0	-0.1833	-	3.89	0.8415	0.5149	32.15
Ciconiidae	*Anastomus lamelligerus*	Afrotropics	0	-0.0683	-	3.56	0.8552	0.7264	58.35
Ciconiidae	*Anastomus oscitans*	IndoMalay	0	-0.1202	-	3.30	0.8061	0.6186	9.20
Ciconiidae	*Ciconia abdimii*	Palearctic	0	0.9089	9.7168	3.71	0.7477	0.4602	51.47
Ciconiidae	*Ciconia boyciana*	IndoMalay	0	-0.1219	-	3.34	0.8255	0.4143	6.48
Ciconiidae	*Ciconia ciconia*	Neotropics	0	0.0458	3.5994	3.86	0.8008	0.6110	15.85
Ciconiidae	*Ciconia episcopus*	Neotropics	0	-0.3140	1.6309	3.40	0.8303	0.4517	48.42
Ciconiidae	*Ciconia maguari*	Neotropics	0	-0.0257	6.1107	2.98	0.6921	0.1672	16.99
Ciconiidae	*Ciconia nigra*	Neotropics	0	0.4045	-	3.02	0.7120	0.2000	67.58
Ciconiidae	*Ciconia stormi*	Neotropics	0	0.0325	5.3148	3.89	0.7937	0.4586	88.65

Family	Species	Realm	Threat	Latitude	Elevation	Anomaly	Size	Shape	Orientation
Ciconiidae	*Ephippiorhynchus asiaticus*	IndoMalay	0	-0.1189	-	3.36	0.7833	0.4255	26.39
Ciconiidae	*Ephippiorhynchus senegalensis*	IndoMalay	0	-0.2344	-	3.18	0.8330	0.6147	85.06
Ciconiidae	*Jabiru mycteria*	Neotropics	0	0.2257	0.5168	3.99	0.7874	0.5378	23.53
Ciconiidae	*Leptoptilos crumeniferus*	Palearctic	1	0.3074	-	4.40	0.7952	0.2354	2.29
Ciconiidae	*Leptoptilos dubius*	Palearctic	0	-0.4597	-	4.36	0.7602	0.1294	1.18
Ciconiidae	*Leptoptilos javanicus*	Australasia	0	-0.4619	-	2.36	0.7266	0.2784	44.75
Ciconiidae	*Mycteria americana*	Palearctic	0	4.1959	7.3086	4.55	0.8012	0.4472	1.82
Ciconiidae	*Mycteria cinerea*	Palearctic	0	8.3341	6.6931	4.55	0.7965	0.2806	14.79
Ciconiidae	*Mycteria ibis*	Palearctic	0	0.1072	2.7063	4.35	0.7296	0.5563	10.93
Ciconiidae	*Mycteria leucocephala*	Neotropics	0	-0.0169	5.5060	3.58	0.7586	0.1400	23.30
Cinclidae	*Cinclus cinclus*	Neotropics	0	-16.7686	6.9195	2.82	0.6690	0.2890	66.73
Cinclidae	*Cinclus leucocephalus*	Neotropics	0	0.0792	12.0833	3.17	0.6935	0.1069	63.59
Cinclidae	*Cinclus mexicanus*	Neotropics	0	-0.6022	14.3860	2.72	0.7933	0.2524	86.42
Cinclidae	*Cinclus pallasii*	Neotropics	0	-2.6719	6.9254	2.79	0.6690	0.3406	66.31
Cinclidae	*Cinclus schulzi*	Neotropics	0	-0.6983	12.5428	2.56	0.7728	0.1517	85.11
Cinclosomatidae	*Cinclosoma ajax*	Neotropics	0	-0.0469	20.8097	3.40	0.7266	0.1766	57.36
Cinclosomatidae	*Cinclosoma castaneothorax*	Neotropics	0	-2.6808	26.6591	4.09	0.7743	0.2672	51.29
Cinclosomatidae	*Cinclosoma castanotum*	Neotropics	0	-0.0427	0.1642	1.60	0.6562	0.1096	16.18
Cinclosomatidae	*Cinclosoma cinnamomeum*	Neotropics	1	-15.6180	8.0857	4.11	0.7128	0.3830	29.53
Cinclosomatidae	*Cinclosoma punctatum*	Neotropics	0	-0.2485	13.5313	3.97	0.7806	0.3761	71.85
Cisticolidae	*Apalis argentea*	Afrotropics	1	23.2264	6.2504	3.48	0.6478	0.1308	83.60
Cisticolidae	*Apalis bamendae*	Afrotropics	0	15.3248	7.4182	3.26	0.6631	0.2797	39.08
Cisticolidae	*Apalis binotata*	Afrotropics	0	0.2037	-	3.26	0.7374	0.4526	2.68
Cisticolidae	*Apalis chapini*	Afrotropics	0	-3.9481	-	3.42	0.7136	0.3875	59.48
Cisticolidae	*Apalis chariessa*	Afrotropics	1	-0.0965	8.4292	3.36	0.6859	0.2514	77.34
Cisticolidae	*Apalis chirindensis*	Afrotropics	0	-0.3254	-	3.56	0.7069	0.3934	51.57
Cisticolidae	*Apalis cinerea*	Afrotropics	0	-0.0079	11.8043	3.35	0.7702	-	-
Cisticolidae	*Apalis cinerea*	Afrotropics	0	-0.0079	11.8043	3.35	0.7702	0.5714	5.66
Cisticolidae	*Apalis flavida*	Afrotropics	0	-0.1448	9.4950	3.47	0.8358	0.6539	29.51
Cisticolidae	*Apalis flavigularis*	Afrotropics	1	-0.8243	9.2540	3.59	0.5788	0.0000	46.12
Cisticolidae	*Apalis goslingi*	Afrotropics	0	0.1048	-	3.28	0.8026	0.4932	6.47
Cisticolidae	*Apalis jacksoni*	Afrotropics	0	0.0087	8.3128	3.34	0.7715	0.5353	2.13
Cisticolidae	*Apalis karamojae*	Afrotropics	1	10.9994	3.2494	3.26	0.6974	0.1648	88.03
Cisticolidae	*Apalis lynesi*	Afrotropics	0	-7.1440	5.2405	3.51	0.5788	0.0000	0.00
Cisticolidae	*Apalis melanocephala*	Afrotropics	0	-0.1056	-	3.25	0.7611	0.1934	75.56
Cisticolidae	*Apalis nigriceps*	Afrotropics	0	0.1325	-	3.21	0.7821	0.1088	10.93
Cisticolidae	*Apalis personata*	Afrotropics	0	-0.2294	8.9069	3.44	0.7363	0.3754	83.08
Cisticolidae	*Apalis porphyrolaema*	Afrotropics	0	7.6606	10.6229	3.30	0.7441	0.5891	13.58
Cisticolidae	*Apalis ruddi*	Afrotropics	0	-0.3245	-	3.27	0.7296	0.3658	73.10
Cisticolidae	*Apalis rufogularis*	Afrotropics	0	0.0249	10.2929	3.30	0.8139	0.5385	10.76
Cisticolidae	*Apalis sharpii*	Afrotropics	0	0.4094	-	3.14	0.7567	0.2901	10.97
Cisticolidae	*Apalis thoracica*	Afrotropics	0	-0.2810	-	3.50	0.7946	0.2215	65.54
Cisticolidae	*Camaroptera brachyura*	IndoMalay	0	0.1014	-	2.91	0.7352	-	-
Cisticolidae	*Camaroptera brachyura*	IndoMalay	0	0.1014	-	2.91	0.7352	0.3968	55.38
Cisticolidae	*Camaroptera chloronota*	IndoMalay	0	0.0088	3.1870	2.85	0.7903	0.5532	15.88
Cisticolidae	*Camaroptera chloronota*	IndoMalay	0	0.0088	3.1870	2.85	0.7903	-	-
Cisticolidae	*Camaroptera fasciolata*	IndoMalay	0	0.1575	4.6204	2.91	0.7291	0.4594	61.99
Cisticolidae	*Camaroptera simplex*	Neotropics	1	0.0686	6.7235	2.47	0.6716	0.2710	15.00
Cisticolidae	*Camaroptera superciliaris*	Australasia	0	-0.4474	-	3.37	0.8136	0.5778	0.95
Cisticolidae	*Camaroptera undosa*	Australasia	1	-0.1311	0.0000	2.29	0.6302	0.4542	60.96
Cisticolidae	*Cisticola aberdare*	Australasia	0	-0.3940	5.3994	3.33	0.8384	0.6292	13.20
Cisticolidae	*Cisticola aberrans*	Neotropics	0	-0.2692	3.5207	3.53	0.8337	0.6302	84.24
Cisticolidae	*Cisticola angusticauda*	Neotropics	0	-0.3049	10.2879	2.97	0.8197	0.4959	85.13
Cisticolidae	*Cisticola anonymus*	Palearctic	0	-0.8096	-	4.66	0.8757	0.0798	1.51
Cisticolidae	*Cisticola aridulus*	Afrotropics	1	-0.0715	6.6742	3.18	0.7614	0.3874	70.15
Cisticolidae	*Cisticola ayresii*	Palearctic	0	-0.7403	8.8169	4.82	0.8341	0.1860	0.24
Cisticolidae	*Cisticola bodessa*	Afrotropics	1	-0.3596	14.2552	3.65	0.7829	0.6568	14.87
Cisticolidae	*Cisticola brachypterus*	Palearctic	0	-0.7556	7.3973	4.36	0.8042	0.3845	4.51
Cisticolidae	*Cisticola brunnescens*	Palearctic	0	-0.7729	9.0425	4.52	0.8472	0.1942	3.92
Cisticolidae	*Cisticola bulliens*	Afrotropics	0	-0.1808	14.6326	3.62	0.8254	0.5030	73.02
Cisticolidae	*Cisticola cantans*	Palearctic	0	-0.8279	7.0656	4.53	0.8313	0.8093	39.70
Cisticolidae	*Cisticola cherina*	IndoMalay	0	-0.0609	-	3.17	0.8067	0.5352	58.35
Cisticolidae	*Cisticola chiniana*	IndoMalay	0	-0.2961	4.2652	3.11	0.7802	0.3271	88.39
Cisticolidae	*Cisticola chubbi*	IndoMalay	0	0.0525	8.7650	2.87	0.7323	0.1670	55.15
Cisticolidae	*Cisticola cinereolus*	Neotropics	0	-0.1522	3.7676	3.89	0.8307	0.5305	42.22
Cisticolidae	*Cisticola dambo*	Afrotropics	1	0.0000	8.6358	3.20	0.6221	0.5477	0.00
Cisticolidae	*Cisticola discolor*	Afrotropics	0	-0.1337	10.6840	3.53	0.8162	0.4724	46.16
Cisticolidae	*Cisticola erythrops*	Afrotropics	0	-0.0955	-	3.49	0.7829	0.4954	43.64
Cisticolidae	*Cisticola exilis*	Afrotropics	0	0.0928	-	3.28	0.8078	0.5664	10.00
Cisticolidae	*Cisticola eximius*	Afrotropics	0	-0.0795	-	3.81	0.8285	0.6296	74.16
Cisticolidae	*Cisticola fulvicapilla*	Afrotropics	0	-0.0653	-	3.54	0.7974	0.5921	88.91
Cisticolidae	*Cisticola fulvicapilla*	Afrotropics	0	-0.0653	-	3.54	0.7974	-	-
Cisticolidae	*Cisticola galactotes*	Afrotropics	0	0.0868	-	3.45	0.7640	0.5879	67.47
Cisticolidae	*Cisticola guinea*	Afrotropics	0	-0.0080	-	3.43	0.8410	0.4918	22.28

Family	Species	Realm	Threat	Latitude	Elevation	Anomaly	Size	Shape	Orientation
Cisticolidae	*Cisticola haesitatus*	Afrotropics	0	-0.0732	6.4866	3.55	0.8037	0.7545	77.71
Cisticolidae	*Cisticola hunteri*	Afrotropics	0	0.0370	-	3.42	0.7452	0.3455	85.66
Cisticolidae	*Cisticola juncidis*	Afrotropics	0	-0.0912	-	3.43	0.8338	0.3491	17.77
Cisticolidae	*Cisticola lais*	Afrotropics	0	-6.5101	-	3.35	0.7359	0.7148	19.12
Cisticolidae	*Cisticola lateralis*	Afrotropics	0	-0.0704	6.6742	3.18	0.7614	0.3874	70.15
Cisticolidae	*Cisticola melanurus*	Afrotropics	0	-0.1117	9.5683	3.64	0.8374	0.6868	54.76
Cisticolidae	*Cisticola nanus*	Afrotropics	0	1.4072	12.1397	3.37	0.7469	0.6203	45.66
Cisticolidae	*Cisticola natalensis*	Afrotropics	0	-0.0305	-	3.31	0.7752	0.5217	47.23
Cisticolidae	*Cisticola nigriloris*	Afrotropics	0	-9.2018	-	3.66	0.7653	0.5299	26.68
Cisticolidae	*Cisticola njombe*	Afrotropics	0	0.6576	12.0521	3.22	0.6974	0.4592	40.73
Cisticolidae	*Cisticola pipiens*	Afrotropics	0	-0.0276	9.1168	3.42	0.8385	0.4349	29.88
Cisticolidae	*Cisticola restrictus*	IndoMalay	0	-0.2567	-	3.11	0.8389	0.3267	43.81
Cisticolidae	*Cisticola robustus*	Afrotropics	0	0.0217	-	3.34	0.7784	0.2201	4.67
Cisticolidae	*Cisticola ruficeps*	Afrotropics	0	-0.2274	11.7055	3.75	0.8258	0.9934	43.44
Cisticolidae	*Cisticola rufilatus*	Afrotropics	0	-0.0118	-	3.43	0.8417	0.5627	26.93
Cisticolidae	*Cisticola rufus*	Afrotropics	0	0.0693	4.0432	3.53	0.7578	0.1316	4.18
Cisticolidae	*Cisticola textrix*	Afrotropics	0	0.3191	16.3933	3.20	0.7403	0.5206	87.48
Cisticolidae	*Cisticola tinniens*	Afrotropics	0	-0.2986	9.1645	3.44	0.8643	0.4576	8.14
Cisticolidae	*Cisticola troglodytes*	Afrotropics	0	-0.0835	13.3951	3.52	0.7858	0.4896	72.65
Cisticolidae	*Cisticola woosnami*	Afrotropics	0	0.0200	-	3.34	0.8185	0.3856	17.04
Cisticolidae	*Drymocichla incana*	Neotropics	0	1.2446	3.7635	3.57	0.7323	0.3242	79.50
Cisticolidae	*Eminia lepida*	Palearctic	0	-1.1385	-	4.95	0.8438	0.4583	0.16
Cisticolidae	*Euryptila subcinnamomea*	Afrotropics	0	-0.2667	-	3.38	0.7606	0.4389	22.66
Cisticolidae	*Heliolais erythropterus*	IndoMalay	0	-0.0272	-	2.83	0.7656	0.3491	2.76
Cisticolidae	*Hypergerus atriceps*	Neotropics	1	-0.0238	6.5044	3.16	0.6662	0.2097	82.57
Cisticolidae	*Incana incana*	Neotropics	0	-0.0949	1.8303	4.01	0.8184	0.5878	46.86
Cisticolidae	*Malcorus pectoralis*	Afrotropics	0	-0.0639	-	3.42	0.8529	0.3057	1.57
Cisticolidae	*Oreolais pulchra*	Neotropics	0	-3.8727	6.5191	3.98	0.7359	-	-
Cisticolidae	*Oreolais pulchra*	Neotropics	0	-3.8727	6.5191	3.98	0.7359	0.1641	39.15
Cisticolidae	*Oreolais ruwenzorii*	Neotropics	0	0.0098	9.2519	3.63	0.7663	0.3968	75.68
Cisticolidae	*Phragmacia substriata*	Nearctic	0	-0.5718	-	6.11	0.8248	0.0355	0.57
Cisticolidae	*Phyllolais pulchella*	Australasia	0	-0.1578	-	2.58	0.6302	0.1134	34.50
Cisticolidae	*Prinia atrogularis*	Afrotropics	0	0.0627	-	3.33	0.7959	0.4615	54.71
Cisticolidae	*Prinia bairdii*	Afrotropics	0	0.1930	4.1166	3.50	0.8115	0.2582	7.88
Cisticolidae	*Prinia buchanani*	Afrotropics	0	-0.1295	-	3.63	0.8564	0.7451	51.54
Cisticolidae	*Prinia burnesii*	IndoMalay	0	-0.0067	1.7468	3.05	0.7712	0.3405	31.43
Cisticolidae	*Prinia cinereocapilla*	Afrotropics	0	-0.0763	-	3.61	0.8158	0.2408	54.78
Cisticolidae	*Prinia crinigera*	Australasia	0	-0.6020	3.2990	2.98	0.7905	0.2705	6.15
Cisticolidae	*Prinia familiaris*	Australasia	1	-0.4598	2.8444	2.38	0.7245	0.2759	41.92
Cisticolidae	*Prinia flavicans*	Afrotropics	0	1.0176	-	3.18	0.6891	0.6106	46.11
Cisticolidae	*Prinia flaviventris*	Neotropics	0	-0.1041	-	3.44	0.7446	0.3535	18.66
Cisticolidae	*Prinia fluviatilis*	Nearctic	0	-0.7852	-	4.07	0.8358	0.5638	9.60
Cisticolidae	*Prinia gracilis*	Nearctic	0	-0.2620	-	3.07	0.7112	0.0994	54.78
Cisticolidae	*Prinia hypoxantha*	Neotropics	0	-0.3765	-	3.52	0.8200	0.6390	64.19
Cisticolidae	*Prinia inornata*	Neotropics	0	-0.0883	-	4.11	0.7946	0.8726	59.61
Cisticolidae	*Prinia leontica*	Neotropics	0	-0.4659	1.3963	3.01	0.7593	0.3237	20.56
Cisticolidae	*Prinia leucopogon*	Neotropics	0	-0.2448	-	2.52	0.6562	0.1636	17.95
Cisticolidae	*Prinia maculosa*	Nearctic	0	-0.5287	-	4.12	0.7864	0.3222	39.92
Cisticolidae	*Prinia polychroa*	Nearctic	0	-0.2420	-	3.80	0.7300	0.1558	58.93
Cisticolidae	*Prinia robertsi*	Neotropics	0	-0.1272	9.3480	3.78	0.8369	0.3659	20.55
Cisticolidae	*Prinia rufescens*	Neotropics	0	0.1066	-	3.11	0.7261	0.2965	64.77
Cisticolidae	*Prinia socialis*	Neotropics	0	0.0384	9.1429	3.98	0.7611	0.1689	42.24
Cisticolidae	*Prinia somalica*	Afrotropics	0	-0.0881	-	3.15	0.7558	0.3897	69.43
Cisticolidae	*Prinia subflava*	Afrotropics	0	-0.0989	-	3.53	0.8568	0.7179	45.55
Cisticolidae	*Prinia sylvatica*	Neotropics	0	-0.1271	11.5816	3.86	0.7327	0.1883	54.13
Cisticolidae	*Rhopophilus pekinensis*	Neotropics	0	0.0066	-	3.98	0.8319	0.6552	6.39
Cisticolidae	*Scotocerca inquieta*	Neotropics	0	-0.2588	-	3.78	0.7378	0.2464	58.04
Cisticolidae	*Spiloptila clamans*	Australasia	0	-0.2625	-	2.17	0.6804	0.6686	14.77
Cisticolidae	*Spiloptila rufifrons*	Australasia	0	-0.4135	5.4708	3.02	0.6690	0.2436	67.69
Cisticolidae	*Urolais epichlorus*	Afrotropics	0	0.1107	6.3766	3.23	0.8079	0.2538	14.99
Climacteridae	*Climacteris affinis*	Afrotropics	0	1.6474	-	3.45	0.7920	0.5519	6.10
Climacteridae	*Climacteris erythrops*	Afrotropics	0	1.8625	-	3.50	0.7935	0.5494	60.08
Climacteridae	*Climacteris melanurus*	Neotropics	1	-25.4725	8.3470	3.59	0.6842	0.3602	51.95
Climacteridae	*Climacteris picumnus*	Neotropics	0	-0.0447	6.7367	4.11	0.6478	0.3853	40.62
Climacteridae	*Climacteris rufus*	Nearctic	0	-0.8063	-	4.22	0.8310	0.4140	6.28
Climacteridae	*Cormobates leucophaea*	Australasia	0	-0.3432	-	3.22	0.8239	0.5508	66.86
Climacteridae	*Cormobates placens*	Australasia	0	-0.1203	-	2.48	0.6221	0.5774	71.57
Cnemophilidae	*Cnemophilus loriae*	Neotropics	0	-0.4747	3.5346	2.94	0.7580	0.8629	1.25
Cnemophilidae	*Cnemophilus macgregorii*	Australasia	0	-0.6077	-	3.32	0.8119	0.3418	4.87
Cnemophilidae	*Loboparadisea sericea*	Nearctic	0	-1.8928	8.3420	4.63	0.6986	0.6293	82.74
Coerebidae	*Coereba flaveola*	Neotropics	0	0.3968	2.5821	2.41	0.5788	0.0000	0.00
Coliidae	*Colius castanotus*	Neotropics	0	-0.1469	8.1681	3.71	0.7409	0.1977	47.04
Coliidae	*Colius colius*	Neotropics	0	-0.3188	-	3.46	0.8319	0.5077	51.79
Coliidae	*Colius leucocephalus*	Nearctic	0	-0.0997	3.2340	3.80	0.7515	0.5419	74.19

Family	Species	Realm	Threat	Latitude	Elevation	Anomaly	Size	Shape	Orientation
Coliidae	*Colius striatus*	Neotropics	1	-0.3352	-	2.53	0.6974	0.1632	17.43
Coliidae	*Urocolius indicus*	Neotropics	0	-0.1520	-	3.83	0.8365	0.5426	42.30
Coliidae	*Urocolius macrourus*	Neotropics	0	-0.4070	-	3.31	0.7961	0.5071	44.58
Colluricinclidae	*Colluricincla boweri*	Australasia	0	-0.0799	-	2.78	0.7642	0.2047	22.97
Colluricinclidae	*Colluricincla harmonica*	Australasia	0	0.0850	7.2673	2.68	0.7198	0.4112	7.97
Colluricinclidae	*Colluricincla megarhyncha*	IndoMalay	0	0.0152	1.3843	2.76	0.7482	0.4281	34.98
Colluricinclidae	*Colluricincla umbrina*	IndoMalay	0	0.0031	-	2.83	0.7820	0.6220	18.35
Colluricinclidae	*Colluricincla woodwardi*	IndoMalay	0	-0.0014	4.9603	2.64	0.7318	0.3128	67.97
Colluricinclidae	*Oreoica gutturalis*	Neotropics	0	-0.8727	9.8235	3.43	0.6740	0.1776	64.13
Colluricinclidae	*Pitohui cristatus*	Neotropics	0	0.0608	7.3631	2.99	0.6716	0.2765	64.11
Colluricinclidae	*Pitohui dichrous*	IndoMalay	0	-0.2297	8.3542	3.27	0.8251	0.6993	19.15
Colluricinclidae	*Pitohui ferrugineus*	Neotropics	0	0.3890	-	3.73	0.7750	0.4672	76.65
Colluricinclidae	*Pitohui incertus*	Neotropics	0	0.1080	3.3806	3.37	0.6523	0.3781	24.46
Colluricinclidae	*Pitohui kirhocephalus*	Neotropics	0	0.3087	-	3.50	0.6986	0.1581	16.02
Colluricinclidae	*Pitohui nigrescens*	Neotropics	0	-0.4261	-	2.66	0.7619	0.5442	62.10
Columbidae	*Alectroenas madagascariensis*	Palearctic	0	-0.5453	-	3.34	0.7854	0.7545	58.55
Columbidae	*Aplopelia larvata*	Afrotropics	0	-0.0752	14.0060	3.39	0.8110	0.8761	82.91
Columbidae	*Caloenas nicobarica*	Nearctic	0	-0.5279	-	4.26	0.7720	0.7919	63.39
Columbidae	*Chalcophaps indica*	Neotropics	1	-0.0289	10.2822	3.32	0.7409	0.3426	83.21
Columbidae	*Chalcophaps stephani*	Neotropics	0	0.0923	9.7839	3.54	0.7331	0.2699	54.83
Columbidae	*Claravis mondetoura*	Afrotropics	0	-0.0564	-	3.74	0.7793	0.3618	54.10
Columbidae	*Claravis pretiosa*	Afrotropics	0	-0.2840	-	3.59	0.7706	0.4905	70.40
Columbidae	*Columba albinucha*	IndoMalay	0	0.2746	-	2.52	0.5985	0.0024	45.41
Columbidae	*Columba albitorques*	Australasia	0	0.0250	6.6557	2.81	0.7240	0.2186	28.04
Columbidae	*Columba argentina*	IndoMalay	0	-0.0392	-	3.11	0.7542	0.7714	8.87
Columbidae	*Columba arquatrix*	IndoMalay	0	-0.0382	-	2.81	0.7576	0.5824	39.55
Columbidae	*Columba delegorguei*	Australasia	0	-0.2248	6.2179	2.86	0.7305	0.6423	1.42
Columbidae	*Columba elphinstonii*	IndoMalay	0	-0.0019	-	2.63	0.7340	0.3306	67.67
Columbidae	*Columba eversmanni*	IndoMalay	0	0.0784	-	3.05	0.7367	0.1967	55.02
Columbidae	*Columba guinea*	Australasia	0	-0.1302	-	2.75	0.7787	0.2047	10.84
Columbidae	*Columba hodgsonii*	IndoMalay	0	-0.2414	11.2671	2.74	0.6562	0.3722	20.74
Columbidae	*Columba iriditorques*	IndoMalay	0	-0.0316	-	2.64	0.7120	0.1691	68.47
Columbidae	*Columba leucomela*	Australasia	0	-0.3449	-	3.02	0.6824	0.2220	64.22
Columbidae	*Columba leuconota*	Australasia	0	-0.4211	-	3.29	0.8366	0.6847	1.94
Columbidae	*Columba livia*	Australasia	0	-0.3387	-	2.92	0.7896	0.5879	53.81
Columbidae	*Columba oenas*	Australasia	0	-0.3033	6.3398	2.86	0.7128	0.1442	12.23
Columbidae	*Columba oliviae*	Australasia	0	-0.1294	-	3.40	0.7659	0.3724	3.69
Columbidae	*Columba pallidiceps*	Neotropics	0	-0.1895	-	3.83	0.8372	0.5615	34.51
Columbidae	*Columba palumbus*	Neotropics	0	-0.7004	6.6422	2.01	0.7542	0.3069	86.77
Columbidae	*Columba pulchricollis*	Afrotropics	0	0.3082	9.1509	3.38	0.7172	0.2306	16.03
Columbidae	*Columba punicea*	Afrotropics	0	0.0795	15.4423	3.58	0.7392	0.4893	83.67
Columbidae	*Columba rupestris*	IndoMalay	1	0.0383	0.0217	2.69	0.5788	0.0000	34.99
Columbidae	*Columba sjostedti*	Afrotropics	0	-0.1409	14.6050	3.45	0.8052	0.3327	71.23
Columbidae	*Columba torringtoniae*	Afrotropics	0	-0.1810	11.7105	3.21	0.7505	0.1662	81.13
Columbidae	*Columba unicincta*	IndoMalay	1	-0.0403	10.2755	3.15	0.7251	0.1556	67.80
Columbidae	*Columba vitiensis*	Palearctic	1	-1.0841	-	4.30	0.8130	0.3475	1.54
Columbidae	*Columbina buckleyi*	Afrotropics	0	-0.1759	12.0574	3.61	0.8494	0.7737	42.18
Columbidae	*Columbina cruziana*	Palearctic	0	-0.4952	11.7147	3.79	0.8074	0.5672	1.69
Columbidae	*Columbina cyanopis*	Afrotropics	0	0.0228	6.3665	3.27	0.8128	0.2624	15.47
Columbidae	*Columbina inca*	Australasia	0	-0.5332	-	3.09	0.7472	0.3332	78.88
Columbidae	*Columbina minuta*	Palearctic	0	-0.6730	11.6965	4.38	0.8121	0.4761	8.88
Columbidae	*Columbina passerina*	Palearctic	0	-0.4615	11.7097	4.04	0.8835	0.2540	6.27
Columbidae	*Columbina picui*	Palearctic	0	-0.6952	-	4.33	0.8461	0.2930	1.76
Columbidae	*Columbina squammata*	Afrotropics	0	0.2687	3.2145	3.28	0.6921	0.4764	5.24
Columbidae	*Columbina talpacoti*	Australasia	1	-0.2856	4.2997	2.49	0.6824	0.2324	22.19
Columbidae	*Cryptophaps poecilorrhoa*	Palearctic	0	-0.7970	6.9697	4.71	0.8536	0.1754	2.46
Columbidae	*Drepanoptila holosericea*	Afrotropics	0	0.0534	4.3267	3.56	0.7524	0.6441	80.77
Columbidae	*Ducula aenea*	Neotropics	0	-0.2975	5.2019	3.14	0.7621	0.3645	34.44
Columbidae	*Ducula badia*	Neotropics	0	-0.5664	6.8562	2.96	0.6974	0.3732	37.08
Columbidae	*Ducula bakeri*	Neotropics	0	-0.5208	-	2.98	0.7744	0.3408	32.51
Columbidae	*Ducula basilica*	Neotropics	0	-0.4869	3.8576	2.94	0.7340	0.2565	33.20
Columbidae	*Ducula bicolor*	Neotropics	0	-0.3985	-	3.05	0.7510	0.4466	16.90
Columbidae	*Ducula brenchleyi*	Neotropics	0	-0.2891	3.2555	3.02	0.7464	0.2684	52.40
Columbidae	*Ducula brenchleyi*	Neotropics	0	-0.2891	3.2555	3.02	0.7464	-	-
Columbidae	*Ducula carola*	Neotropics	0	-0.5650	-	2.89	0.7877	0.4970	78.44
Columbidae	*Ducula chalconota*	Neotropics	0	-0.6158	7.3042	3.31	0.6921	0.3259	81.10
Columbidae	*Ducula cineracea*	Neotropics	1	-0.5855	3.4015	3.16	0.7654	0.7722	10.94
Columbidae	*Ducula concinna*	IndoMalay	0	-0.3957	-	2.97	0.8133	0.6449	38.21
Columbidae	*Ducula finschii*	Neotropics	0	-0.1727	-	3.80	0.8548	0.4198	36.21
Columbidae	*Ducula forsteni*	Palearctic	0	-0.7543	7.0742	4.75	0.8649	0.1822	1.03
Columbidae	*Ducula goliath*	Nearctic	0	-0.6689	6.5479	4.09	0.8343	0.4072	14.11
Columbidae	*Ducula lacernulata*	Neotropics	0	-0.5041	-	3.24	0.7664	0.5144	67.97
Columbidae	*Ducula latrans*	Afrotropics	0	-0.0744	6.0192	3.18	0.7614	0.3874	70.15
Columbidae	*Ducula luctuosa*	Afrotropics	0	0.0068	-	3.31	0.7656	0.5039	4.31

Family	Species	Realm	Threat	Latitude	Elevation	Anomaly	Size	Shape	Orientation
Columbidae	*Ducula melanochroa*	Afrotropics	0	-0.1580	-	3.65	0.8328	0.7899	47.01
Columbidae	*Ducula mindorensis*	Afrotropics	0	0.0532	-	3.44	0.8329	0.2640	5.52
Columbidae	*Ducula mullerii*	Afrotropics	0	-0.0548	-	3.23	0.7664	0.6262	60.63
Columbidae	*Ducula myristicivora*	Afrotropics	0	0.0312	-	3.23	0.7928	0.2544	17.62
Columbidae	*Ducula pacifica*	Afrotropics	0	0.1483	-	3.26	0.7961	0.4706	5.32
Columbidae	*Ducula perspicillata*	Afrotropics	0	-0.0785	-	3.25	0.8129	0.3211	8.39
Columbidae	*Ducula pinon*	Neotropics	0	-0.0153	6.0510	3.58	0.7582	0.2977	81.51
Columbidae	*Ducula pistrinaria*	IndoMalay	0	-0.1022	2.5281	3.00	0.8281	0.5332	34.67
Columbidae	*Ducula poliocephala*	IndoMalay	0	-0.0012	-	3.03	0.8153	0.5077	51.90
Columbidae	*Ducula rosacea*	Australasia	0	0.8758	3.2333	2.48	0.6302	0.2423	87.23
Columbidae	*Ducula rubricera*	IndoMalay	0	-0.0518	0.0000	2.73	0.7833	0.5321	15.79
Columbidae	*Ducula rufigaster*	Australasia	1	-0.8334	2.4808	2.46	0.5985	0.1035	28.88
Columbidae	*Ducula spilorrhoa*	IndoMalay	1	-0.0118	6.0002	2.65	0.7281	0.2077	67.81
Columbidae	*Ducula subflavescens*	Australasia	0	-0.0843	6.3408	2.81	0.7425	0.1455	23.88
Columbidae	*Ducula zoeae*	Australasia	1	0.0289	8.2971	2.56	0.6370	0.1401	27.99
Columbidae	*Gallicolumba beccarii*	Neotropics	0	-0.2144	1.4895	3.06	0.7211	0.4837	61.35
Columbidae	*Gallicolumba crinigera*	Neotropics	0	3.9930	13.2902	4.06	0.7595	0.4285	81.77
Columbidae	*Gallicolumba hoedtii*	Afrotropics	0	-0.2571	-	3.71	0.8306	0.5911	74.73
Columbidae	*Gallicolumba jobiensis*	Neotropics	0	-2.1597	18.1586	4.12	0.7648	0.2928	43.72
Columbidae	*Gallicolumba keayi*	Neotropics	0	-0.5601	10.4976	2.75	0.8145	0.4574	69.85
Columbidae	*Gallicolumba luzonica*	Neotropics	0	-0.6963	-	1.51	0.5428	-	-
Columbidae	*Gallicolumba platenae*	Neotropics	0	-0.4009	-	3.15	0.7697	0.5730	81.38
Columbidae	*Gallicolumba rufigula*	Neotropics	0	-0.1268	-	3.72	0.8026	0.7572	33.39
Columbidae	*Gallicolumba sanctaecrucis*	Neotropics	0	-0.0198	7.7237	3.91	0.8293	0.4777	13.66
Columbidae	*Gallicolumba stairi*	Neotropics	0	0.1251	0.8549	4.04	0.7623	0.2166	1.24
Columbidae	*Gallicolumba tristigmata*	Neotropics	0	-0.4108	-	3.40	0.8332	0.5433	46.45
Columbidae	*Geopelia cuneata*	IndoMalay	0	-0.0006	-	2.82	0.7344	0.2824	9.04
Columbidae	*Geopelia humeralis*	IndoMalay	0	-0.4604	-	3.29	0.8097	0.4940	16.65
Columbidae	*Geopelia maugeus*	IndoMalay	0	0.1359	4.5507	2.82	0.6859	0.6680	74.37
Columbidae	*Geopelia placida*	IndoMalay	0	-0.6083	-	3.30	0.7957	0.6805	41.01
Columbidae	*Geopelia striata*	IndoMalay	0	-0.8571	7.9165	2.61	0.6478	0.3444	72.46
Columbidae	*Geophaps plumifera*	IndoMalay	0	-1.0309	7.4909	2.61	0.6478	0.3444	72.46
Columbidae	*Geophaps scripta*	IndoMalay	0	0.1448	-	3.41	0.7546	0.5102	11.88
Columbidae	*Geophaps smithii*	IndoMalay	0	-0.3559	7.8885	2.75	0.6598	0.2960	18.25
Columbidae	*Geotrygon albifacies*	Australasia	0	-0.0966	-	2.74	0.7752	0.3686	4.69
Columbidae	*Geotrygon caniceps*	Australasia	0	-0.1621	5.6215	2.49	0.6824	0.2324	22.19
Columbidae	*Geotrygon carrikeri*	Australasia	0	-0.2434	7.1448	2.84	0.7493	0.1976	24.01
Columbidae	*Geotrygon chiriquensis*	Australasia	0	-0.4855	10.1903	2.72	0.6763	0.3369	68.79
Columbidae	*Geotrygon chrysia*	Australasia	0	-0.4832	-	3.48	0.8307	-	-
Columbidae	*Geotrygon chrysia*	Australasia	0	-0.4832	-	3.48	0.8307	0.5130	11.55
Columbidae	*Geotrygon costaricensis*	Australasia	0	-0.3421	-	3.36	0.8122	0.5143	29.96
Columbidae	*Geotrygon frenata*	Australasia	0	0.0512	4.8396	2.59	0.6891	0.1686	0.56
Columbidae	*Geotrygon goldmani*	Australasia	0	-0.4115	0.0000	3.37	0.8307	0.6196	19.19
Columbidae	*Geotrygon lawrencii*	IndoMalay	0	0.0013	1.9734	2.82	0.7932	0.8543	18.03
Columbidae	*Geotrygon linearis*	Australasia	0	-0.4317	-	3.60	0.8025	0.3886	0.72
Columbidae	*Geotrygon montana*	Australasia	0	-0.4014	-	3.42	0.7799	0.3113	55.85
Columbidae	*Geotrygon mystacea*	Australasia	0	-0.0138	-	3.17	0.6876	0.1266	20.50
Columbidae	*Geotrygon saphirina*	Neotropics	0	-0.7746	3.1774	1.84	0.7165	0.3385	63.71
Columbidae	*Geotrygon veraguensis*	Neotropics	0	-0.2269	15.0366	3.95	0.7069	0.1568	46.75
Columbidae	*Geotrygon versicolor*	Neotropics	0	-0.4476	10.7433	2.84	0.8146	0.5606	88.94
Columbidae	*Geotrygon violacea*	Neotropics	0	10.2968	11.6144	3.11	0.6906	0.3372	81.26
Columbidae	*Goura cristata*	IndoMalay	0	0.1492	3.2390	2.77	0.6804	0.5220	77.37
Columbidae	*Goura scheepmakeri*	IndoMalay	0	-0.2862	-	2.70	0.6859	0.1498	12.65
Columbidae	*Goura victoria*	Neotropics	0	-0.0858	1.8050	3.03	0.6598	0.3478	35.91
Columbidae	*Gymnophaps albertisii*	Australasia	1	-0.6800	-	3.14	0.7629	0.5887	58.71
Columbidae	*Gymnophaps mada*	Afrotropics	0	-0.4269	-	3.44	0.6986	0.4867	80.22
Columbidae	*Gymnophaps solomonensis*	Neotropics	0	-0.1837	-	3.96	0.7795	0.3357	4.89
Columbidae	*Hemiphaga novaeseelandiae*	Neotropics	0	-0.0976	7.1336	4.08	0.6631	0.3162	45.35
Columbidae	*Henicophaps albifrons*	Neotropics	1	0.3956	5.1721	3.74	0.6763	0.2078	59.49
Columbidae	*Henicophaps foersteri*	Neotropics	0	0.0285	6.0125	3.66	0.7613	0.4141	82.85
Columbidae	*Leptotila battyi*	Neotropics	0	0.0048	-	3.18	0.7647	0.6807	61.01
Columbidae	*Leptotila cassini*	Neotropics	0	-0.1421	-	3.92	0.8304	0.6186	44.41
Columbidae	*Leptotila conoveri*	Neotropics	0	-0.4655	8.2641	3.32	0.7713	0.3618	70.43
Columbidae	*Leptotila jamaicensis*	Neotropics	0	-0.5155	-	2.70	0.7789	0.4795	33.33
Columbidae	*Leptotila megalura*	Neotropics	1	-0.7898	5.1755	3.83	0.6716	0.3168	4.68
Columbidae	*Leptotila ochraceiventris*	Neotropics	0	-0.2328	-	3.76	0.8456	0.7932	18.76
Columbidae	*Leptotila pallida*	Neotropics	0	-0.1453	-	3.42	0.7643	0.5342	17.76
Columbidae	*Leptotila verreauxi*	Neotropics	1	-0.3520	4.2872	3.86	0.7990	0.4677	16.92
Columbidae	*Leucosarcia melanoleuca*	Neotropics	0	0.0619	3.3474	3.94	0.8166	0.8244	74.73
Columbidae	*Lopholaimus antarcticus*	IndoMalay	0	-0.1573	-	3.36	0.7827	0.5150	23.52
Columbidae	*Macropygia amboinensis*	Neotropics	0	-0.1842	-	2.46	0.6935	0.4554	10.15
Columbidae	*Macropygia mackinlayi*	Neotropics	0	0.1232	-	2.47	0.5985	0.0000	0.00
Columbidae	*Macropygia nigrirostris*	Australasia	0	-0.5834	1.1396	2.36	0.7482	0.0888	0.56
Columbidae	*Macropygia ruficeps*	Palearctic	0	-0.6724	6.9048	4.07	0.8398	0.4510	6.02

Family	Species	Realm	Threat	Latitude	Elevation	Anomaly	Size	Shape	Orientation
Columbidae	*Macropygia tenuirostris*	Neotropics	0	0.1008	7.8067	3.61	0.7558	0.5055	82.85
Columbidae	*Macropygia unchall*	Neotropics	0	-0.1751	4.7171	3.85	0.8423	0.5966	37.70
Columbidae	*Metriopelia aymara*	Australasia	0	0.0946	-	2.48	0.6221	0.5774	71.57
Columbidae	*Metriopelia ceciliae*	Neotropics	0	-0.3587	1.4177	2.53	0.6974	0.1632	17.43
Columbidae	*Metriopelia melanoptera*	Neotropics	0	-0.0321	-	2.46	0.6921	0.2089	5.23
Columbidae	*Metriopelia morenoi*	Afrotropics	0	-0.0672	-	3.43	0.8360	0.4157	25.73
Columbidae	*Nesoenas picturata*	Australasia	0	0.4530	-	2.59	0.6784	0.1433	4.01
Columbidae	*Ocyphaps lophotes*	Neotropics	0	0.0600	9.4876	3.44	0.7469	0.7189	55.91
Columbidae	*Oena capensis*	Nearctic	0	-0.7409	6.6641	4.06	0.8080	0.5479	46.82
Columbidae	*Otidiphaps nobilis*	Neotropics	0	0.2608	8.8954	4.01	0.6986	0.3594	68.85
Columbidae	*Patagioenas araucana*	Australasia	0	-0.3860	-	2.65	0.6763	0.2262	48.22
Columbidae	*Patagioenas caribaea*	Australasia	0	-0.3044	-	2.88	0.7158	0.2818	36.87
Columbidae	*Patagioenas cayennensis*	Australasia	0	-0.4167	-	2.52	0.6478	0.3065	45.21
Columbidae	*Patagioenas corensis*	Australasia	1	0.1198	3.5763	2.74	0.6562	0.2807	37.50
Columbidae	*Patagioenas fasciata*	Nearctic	0	-0.9194	-	4.18	0.8184	0.6632	25.41
Columbidae	*Patagioenas flavirostris*	Neotropics	0	0.0096	7.0286	3.05	0.6562	-	-
Columbidae	*Patagioenas flavirostris*	Neotropics	0	0.0096	7.0286	3.05	0.6562	0.3979	32.60
Columbidae	*Patagioenas goodsoni*	Neotropics	0	-0.1864	-	3.61	0.8435	0.4779	42.11
Columbidae	*Patagioenas inornata*	Neotropics	0	-0.0750	13.3022	3.73	0.7670	0.2327	28.79
Columbidae	*Patagioenas leucocephala*	Afrotropics	0	-0.1466	11.6016	3.39	0.7580	0.4728	12.34
Columbidae	*Patagioenas maculosa*	Afrotropics	0	5.7464	13.4131	3.27	0.7621	0.3920	22.60
Columbidae	*Patagioenas nigrirostris*	IndoMalay	0	0.2946	-	2.52	0.5985	0.0024	45.41
Columbidae	*Patagioenas oenops*	Palearctic	0	-0.6282	10.8633	4.48	0.8650	0.2055	0.55
Columbidae	*Patagioenas picazuro*	Nearctic	0	-0.6992	10.1209	4.22	0.8443	0.2705	11.90
Columbidae	*Patagioenas plumbea*	Palearctic	0	-0.6156	7.9057	4.11	0.8460	0.4469	8.76
Columbidae	*Patagioenas speciosa*	Nearctic	0	-0.8265	5.0812	4.05	0.8057	0.4352	10.93
Columbidae	*Patagioenas squamosa*	Palearctic	0	-0.7517	-	5.32	0.8499	0.0686	0.63
Columbidae	*Patagioenas subvinacea*	Afrotropics	0	-0.2939	9.1776	4.16	0.8012	0.6822	30.85
Columbidae	*Petrophassa albipennis*	Palearctic	1	-1.0102	-	4.74	0.8199	0.3142	6.15
Columbidae	*Petrophassa rufipennis*	Nearctic	0	-0.9484	-	4.30	0.7959	0.8119	3.09
Columbidae	*Phapitreron amethystinus*	Australasia	0	-0.5662	-	3.07	0.7721	0.4484	57.57
Columbidae	*Phapitreron brunneiceps*	Palearctic	0	-0.8007	0.0000	4.14	0.7980	0.4889	19.18
Columbidae	*Phapitreron leucotis*	Afrotropics	0	0.0083	7.7553	3.56	0.8255	0.1595	3.61
Columbidae	*Phaps chalcoptera*	Palearctic	0	-1.0316	-	4.15	0.8464	0.1281	4.01
Columbidae	*Phaps elegans*	Afrotropics	0	0.0590	-	3.45	0.8046	0.2609	12.46
Columbidae	*Phaps histrionica*	Afrotropics	0	-0.2683	-	3.70	0.8252	0.8500	58.67
Columbidae	*Ptilinopus arcanus*	Australasia	0	-0.7226	-	3.27	0.8170	0.3356	4.08
Columbidae	*Ptilinopus aurantiifrons*	Afrotropics	0	0.0400	-	3.29	0.7678	0.2908	42.05
Columbidae	*Ptilinopus bernsteinii*	Neotropics	0	0.0214	9.8033	3.82	0.6986	0.1529	50.36
Columbidae	*Ptilinopus cinctus*	Neotropics	0	-0.6796	4.0089	3.01	0.7165	0.1070	78.70
Columbidae	*Ptilinopus coronulatus*	Neotropics	0	-0.6099	2.6044	2.56	0.7653	0.6017	78.75
Columbidae	*Ptilinopus dohertyi*	Neotropics	0	8.8798	11.4480	3.61	0.7296	0.2217	68.70
Columbidae	*Ptilinopus eugeniae*	Neotropics	0	-1.0202	1.9438	3.19	0.6906	0.2681	75.22
Columbidae	*Ptilinopus fischeri*	Australasia	0	-0.0888	-	2.78	0.7713	0.3034	24.02
Columbidae	*Ptilinopus granulifrons*	Afrotropics	0	-0.0873	10.1119	3.58	0.8341	0.6002	39.81
Columbidae	*Ptilinopus greyii*	IndoMalay	1	0.0129	-	2.94	0.7198	0.1784	58.63
Columbidae	*Ptilinopus hyogastrus*	IndoMalay	0	-0.1587	-	3.39	0.8054	0.7950	68.93
Columbidae	*Ptilinopus insolitus*	Afrotropics	0	0.0567	11.3167	3.40	0.7838	0.8212	39.33
Columbidae	*Ptilinopus iozonus*	Afrotropics	0	-0.0922	5.9815	3.12	0.7359	0.2741	71.81
Columbidae	*Ptilinopus jambu*	Australasia	1	0.1513	0.0000	2.50	0.6523	0.2862	32.26
Columbidae	*Ptilinopus luteovirens*	Australasia	0	0.6243	7.1557	2.83	0.7327	0.0678	6.21
Columbidae	*Ptilinopus magnificus*	Afrotropics	1	17.2270	4.4175	3.42	0.6716	0.5254	70.86
Columbidae	*Ptilinopus marchei*	Afrotropics	0	0.0428	-	3.18	0.7382	0.3751	25.70
Columbidae	*Ptilinopus melanospilus*	Neotropics	0	0.0110	11.3819	3.63	0.7712	0.4409	83.75
Columbidae	*Ptilinopus merrilli*	Neotropics	0	-0.2655	11.4163	3.04	0.6662	0.2315	32.82
Columbidae	*Ptilinopus monacha*	Neotropics	0	-0.1691	8.0679	3.68	0.7730	0.2462	62.84
Columbidae	*Ptilinopus naina*	Neotropics	0	-0.6133	2.2907	2.95	0.7588	0.2760	67.67
Columbidae	*Ptilinopus occipitalis*	Neotropics	0	-0.7846	1.4606	2.91	0.7348	0.4522	79.75
Columbidae	*Ptilinopus ornatus*	Neotropics	0	-0.5974	1.1430	2.49	0.7830	0.6810	17.93
Columbidae	*Ptilinopus perlatus*	Neotropics	0	-0.5978	-	2.97	0.7696	0.2999	78.65
Columbidae	*Ptilinopus perousii*	Neotropics	0	-0.5295	-	3.30	0.8042	0.5198	57.19
Columbidae	*Ptilinopus porphyreus*	Neotropics	0	-0.5027	-	2.43	0.7784	0.6025	30.98
Columbidae	*Ptilinopus porphyreus*	Neotropics	0	-0.5027	-	2.43	0.7784	-	-
Columbidae	*Ptilinopus pulchellus*	Afrotropics	0	5.5107	-	3.29	0.7693	0.5062	78.52
Columbidae	*Ptilinopus regina*	Afrotropics	0	0.0827	-	3.28	0.7550	0.4812	59.73
Columbidae	*Ptilinopus rivoli*	Palearctic	0	3.0140	14.1390	4.50	0.8066	0.4520	1.87
Columbidae	*Ptilinopus solomonensis*	Neotropics	0	-0.2206	4.5419	3.58	0.8382	0.5335	51.60
Columbidae	*Ptilinopus subgularis*	Neotropics	0	-0.7477	-	2.47	0.5985	0.0000	0.00
Columbidae	*Ptilinopus superbus*	Neotropics	0	-0.1497	2.2567	3.62	0.7795	0.4834	76.18
Columbidae	*Ptilinopus tannensis*	Neotropics	0	-0.6600	9.5256	2.74	0.7752	0.3540	87.62
Columbidae	*Ptilinopus victor*	Neotropics	0	-0.5282	-	2.91	0.7911	0.6394	81.52
Columbidae	*Ptilinopus viridis*	Neotropics	0	-0.1623	1.1522	4.06	0.7593	0.3572	25.04
Columbidae	*Ptilinopus wallacii*	Neotropics	0	-0.0074	9.8448	3.27	0.7422	0.3738	84.64
Columbidae	*Reinwardtoena browni*	IndoMalay	0	0.5896	0.0000	2.94	0.6859	0.2219	37.99

Family	Species	Realm	Threat	Latitude	Elevation	Anomaly	Size	Shape	Orientation
Columbidae	*Reinwardtoena crassirostris*	IndoMalay	0	-0.2146	-	3.21	0.8290	0.4960	21.50
Columbidae	*Reinwardtoena reinwardtsi*	IndoMalay	0	-0.1157	9.4173	4.10	0.7466	0.1520	25.99
Columbidae	*Starnoenas cyanocephala*	IndoMalay	1	-0.0620	8.9158	3.37	0.7616	0.3972	25.73
Columbidae	*Stigmatopelia chinensis*	Palearctic	0	-1.0797	9.4029	3.93	0.7997	0.2006	16.67
Columbidae	*Stigmatopelia senegalensis*	Palearctic	1	-1.6053	6.8871	3.09	0.5788	0.0000	0.00
Columbidae	*Streptopelia bitorquata*	Neotropics	0	-0.5622	6.2315	1.92	0.7537	0.3476	83.61
Columbidae	*Streptopelia capicola*	Afrotropics	0	-0.1085	-	3.28	0.7901	0.4991	37.31
Columbidae	*Streptopelia hypopyrrha*	IndoMalay	1	0.0000	3.4875	3.91	0.6302	0.6981	0.00
Columbidae	*Streptopelia lugens*	IndoMalay	0	0.1491	9.2595	3.28	0.6428	0.7700	58.10
Columbidae	*Streptopelia orientalis*	IndoMalay	0	0.0735	9.2198	3.32	0.7809	0.3401	0.16
Columbidae	*Streptopelia reichenowi*	IndoMalay	1	-0.5003	6.2741	3.16	0.6562	0.4827	28.26
Columbidae	*Streptopelia roseogrisea*	IndoMalay	1	-0.2294	5.5419	3.22	0.6662	0.2181	8.27
Columbidae	*Streptopelia semitorquata*	IndoMalay	0	-0.4266	8.4837	3.07	0.6784	0.4298	86.71
Columbidae	*Streptopelia tranquebarica*	IndoMalay	0	-0.1394	9.4927	3.35	0.7419	0.2331	86.04
Columbidae	*Streptopelia turtur*	Palearctic	0	-0.7681	10.0577	3.59	0.7729	0.4034	19.57
Columbidae	*Streptopelia vinacea*	Afrotropics	0	0.0414	-	3.20	0.8034	0.2474	16.95
Columbidae	*Treron apicauda*	Neotropics	0	-0.3872	7.8657	2.92	0.8020	0.2123	84.90
Columbidae	*Treron australis*	Afrotropics	0	0.0147	-	3.26	0.8154	0.2579	16.40
Columbidae	*Treron bicinctus*	Neotropics	0	-0.2901	19.7864	3.42	0.7985	0.2582	88.95
Columbidae	*Treron calvus*	Neotropics	0	-0.3722	-	3.09	0.7823	0.2092	84.71
Columbidae	*Treron capellei*	Australasia	0	-0.7595	0.0000	2.69	0.7685	0.1634	12.85
Columbidae	*Treron curvirostra*	Neotropics	0	-0.4231	6.7531	3.89	0.7050	0.2856	12.92
Columbidae	*Treron floris*	Neotropics	0	-47.9196	7.9127	3.52	0.6598	0.4739	67.00
Columbidae	*Treron formosae*	Neotropics	0	0.1115	8.0243	3.57	0.7422	0.2709	74.01
Columbidae	*Treron fulvicollis*	Neotropics	0	-29.8037	8.2869	3.94	0.6562	0.2955	70.78
Columbidae	*Treron griseicauda*	Neotropics	0	4.0377	11.0211	3.96	0.7466	0.3227	53.26
Columbidae	*Treron olax*	Neotropics	0	-0.1577	-	3.80	0.8372	0.6612	10.44
Columbidae	*Treron oxyurus*	Neotropics	0	0.0161	-	3.43	0.7670	0.4382	25.26
Columbidae	*Treron phoenicopterus*	Neotropics	0	-0.2076	-	3.12	0.8110	0.3749	60.44
Columbidae	*Treron pompadora*	Neotropics	0	0.0376	8.4968	3.64	0.7734	0.4570	86.89
Columbidae	*Treron psittaceus*	Neotropics	0	-0.3454	3.5420	2.72	0.7406	0.1752	47.35
Columbidae	*Treron seimundi*	Neotropics	0	-0.0086	-	3.88	0.8406	0.5202	25.17
Columbidae	*Treron sieboldii*	Neotropics	0	0.1630	-	3.78	0.7382	0.3421	10.91
Columbidae	*Treron sphenurus*	Neotropics	0	-0.2671	-	3.07	0.7544	0.2568	47.40
Columbidae	*Treron vernans*	Neotropics	0	-0.2190	-	3.85	0.8513	0.5488	34.13
Columbidae	*Treron waalia*	Neotropics	0	-0.3358	-	3.52	0.8346	0.5190	43.15
Columbidae	*Trugon terrestris*	Neotropics	0	-0.6941	2.8223	2.16	0.7544	0.4688	89.94
Columbidae	*Turacoena manadensis*	Neotropics	0	-0.0332	3.7692	3.96	0.8395	0.6258	35.75
Columbidae	*Turacoena modesta*	Neotropics	1	-3.7698	2.9557	3.84	0.7030	0.1849	71.43
Columbidae	*Turtur abyssinicus*	IndoMalay	0	-0.0348	5.5825	3.13	0.7861	0.5953	58.54
Columbidae	*Turtur afer*	IndoMalay	0	0.0316	1.7908	2.84	0.7857	0.4261	1.27
Columbidae	*Turtur chalcospilos*	Australasia	0	0.0247	-	2.89	0.6935	0.2710	61.76
Columbidae	*Turtur tympanistria*	Australasia	0	-0.4500	-	3.32	0.7861	0.3934	62.28
Columbidae	*Uropelia campestris*	Nearctic	0	-0.7666	1.9466	6.20	0.7712	0.1013	2.72
Columbidae	*Zenaida asiatica*	Afrotropics	0	-0.1367	-	3.81	0.8307	0.6941	60.47
Columbidae	*Zenaida auriculata*	Neotropics	0	-0.1632	-	3.91	0.8496	0.6143	30.98
Columbidae	*Zenaida aurita*	Neotropics	0	-0.3180	7.3216	3.51	0.8535	0.6795	83.13
Columbidae	*Zenaida galapagoensis*	Palearctic	0	-0.8804	-	4.13	0.7667	0.8388	63.37
Columbidae	*Zenaida macroura*	Afrotropics	0	-0.1598	-	3.71	0.8370	0.3622	56.97
Columbidae	*Zenaida meloda*	Afrotropics	0	0.0408	-	3.54	0.8195	0.5251	83.99
Conopophagidae	*Conopophaga ardesiaca*	Neotropics	1	0.0000	7.4132	4.02	0.6598	0.6244	7.29
Conopophagidae	*Conopophaga aurita*	Neotropics	0	-0.1004	-	3.72	0.7933	0.5605	39.82
Conopophagidae	*Conopophaga castaneiceps*	Neotropics	0	-10.9506	-	4.21	0.7256	0.5998	45.51
Conopophagidae	*Conopophaga lineata*	Neotropics	0	0.0400	4.8155	3.63	0.7645	0.3995	76.41
Conopophagidae	*Conopophaga melanogaster*	Neotropics	0	-0.0888	-	3.66	0.7783	0.5510	24.64
Conopophagidae	*Conopophaga melanops*	Neotropics	0	-0.1957	-	3.77	0.7684	0.2739	55.44
Conopophagidae	*Conopophaga peruviana*	Neotropics	0	0.9541	3.7602	4.04	0.7378	0.1315	35.37
Conopophagidae	*Conopophaga roberti*	Neotropics	0	-0.0716	-	3.54	0.7474	0.5621	35.22
Coraciidae	*Coracias abyssinicus*	Neotropics	0	-0.1576	7.4108	3.63	0.8333	0.4209	39.21
Coraciidae	*Coracias benghalensis*	Nearctic	0	-0.8305	10.5207	4.15	0.8465	0.3239	10.56
Coraciidae	*Coracias caudatus*	Neotropics	0	-0.1599	10.6067	3.81	0.7920	0.5766	86.20
Coraciidae	*Coracias cyanogaster*	Neotropics	0	-0.0139	4.9050	2.46	0.6876	0.3855	3.42
Coraciidae	*Coracias garrulus*	Neotropics	0	-0.1268	9.4201	3.05	0.6662	0.3006	33.35
Coraciidae	*Coracias naevia*	Neotropics	0	-0.1268	11.0984	3.48	0.7078	0.2736	86.59
Coraciidae	*Coracias spatulatus*	Neotropics	0	0.1494	4.8552	3.00	0.6370	0.3956	30.13
Coraciidae	*Coracias temminckii*	Neotropics	0	-1.5530	7.4537	2.47	0.5985	0.0000	0.00
Coraciidae	*Eurystomus azureus*	Afrotropics	0	-0.3565	-	3.72	0.7455	0.2081	69.65
Coraciidae	*Eurystomus glaucurus*	Afrotropics	0	-0.1682	-	4.13	0.8030	0.5222	21.55
Coraciidae	*Eurystomus gularis*	Afrotropics	0	0.2131	-	3.63	0.7858	0.1078	3.36
Coraciidae	*Eurystomus orientalis*	Afrotropics	0	-0.0648	-	3.53	0.8311	0.6234	22.81
Corcoracidae	*Corcorax melanorhamphos*	IndoMalay	1	0.0305	2.7682	2.63	0.6478	0.3503	71.22
Corcoracidae	*Struthidea cinerea*	Nearctic	0	-0.5009	-	4.61	0.8288	0.1115	6.29
Corvidae	*Aphelocoma californica*	Nearctic	0	-0.6483	14.6850	4.09	0.8091	0.3961	45.58
Corvidae	*Aphelocoma coerulescens*	Nearctic	1	-0.7858	14.3892	3.26	0.6935	0.4804	70.10

Family	Species	Realm	Threat	Latitude	Elevation	Anomaly	Size	Shape	Orientation
Corvidae	*Aphelocoma ultramarina*	Nearctic	0	-0.4795	12.7898	4.05	0.7717	0.3051	50.38
Corvidae	*Aphelocoma unicolor*	Neotropics	0	-0.1690	11.1903	3.63	0.7382	0.2139	22.56
Corvidae	*Calocitta colliei*	Nearctic	0	-0.5357	-	3.63	0.7870	0.4683	66.97
Corvidae	*Calocitta formosa*	Nearctic	0	-0.1541	-	3.87	0.7228	0.1477	56.99
Corvidae	*Cissa chinensis*	Palearctic	0	-0.5320	5.8713	4.08	0.8548	0.3735	12.94
Corvidae	*Cissa hypoleuca*	Afrotropics	0	-0.0675	15.2782	3.64	0.8455	0.4844	58.35
Corvidae	*Cissa thalassina*	Palearctic	0	-0.7104	6.4231	4.49	0.8563	0.2779	1.35
Corvidae	*Corvus albicollis*	Australasia	0	0.0181	-	2.59	0.6876	0.1757	1.09
Corvidae	*Corvus albus*	IndoMalay	0	0.0065	-	3.01	0.7800	0.4348	28.99
Corvidae	*Corvus brachyrhynchos*	Australasia	0	-0.0397	-	2.81	0.7419	0.2245	26.99
Corvidae	*Corvus capensis*	IndoMalay	0	0.0009	-	2.80	0.7925	0.5118	21.03
Corvidae	*Corvus caurinus*	Australasia	0	-0.0587	-	2.72	0.7144	0.6005	55.67
Corvidae	*Corvus corax*	Australasia	0	-0.3354	-	3.01	0.8050	0.4912	59.61
Corvidae	*Corvus corone*	Afrotropics	0	-0.0728	-	3.18	0.7614	0.3874	70.15
Corvidae	*Corvus coronoides*	Afrotropics	0	-0.0971	-	3.14	0.7548	0.3972	69.59
Corvidae	*Corvus crassirostris*	Australasia	0	1.3606	-	2.73	0.6876	0.4043	69.61
Corvidae	*Corvus cryptoleucus*	Neotropics	0	-0.3910	6.6612	3.70	0.8653	0.4455	52.17
Corvidae	*Corvus dauuricus*	Neotropics	0	-0.0099	3.5619	3.13	0.7340	0.2629	44.49
Corvidae	*Corvus edithae*	Neotropics	0	0.0906	3.8993	4.07	0.7846	0.5219	22.17
Corvidae	*Corvus enca*	Neotropics	0	-0.2037	4.3317	3.75	0.7211	0.6186	32.57
Corvidae	*Corvus florensis*	Australasia	0	-0.5967	-	3.21	0.8003	0.6774	58.14
Corvidae	*Corvus frugilegus*	Australasia	0	-0.5398	4.3363	3.11	0.7806	0.5117	72.68
Corvidae	*Corvus fuscicapillus*	Australasia	0	-0.0707	9.6802	2.82	0.7399	0.1465	23.77
Corvidae	*Corvus imparatus*	Afrotropics	0	-0.0023	9.0898	3.51	0.8197	0.1804	6.79
Corvidae	*Corvus jamaicensis*	Afrotropics	0	-0.1785	11.1606	3.45	0.8047	0.2702	73.20
Corvidae	*Corvus leucognaphalus*	Afrotropics	0	-0.1034	0.0000	3.56	0.8638	0.6585	41.49
Corvidae	*Corvus levaillantii*	Australasia	0	-0.6486	-	3.39	0.8309	0.4901	1.63
Corvidae	*Corvus macrorhynchos*	Nearctic	0	-0.7351	-	4.23	0.8505	0.4798	7.34
Corvidae	*Corvus meeki*	Afrotropics	0	-0.1273	7.5780	3.78	0.8254	0.3392	63.33
Corvidae	*Corvus mellori*	Nearctic	0	-0.5049	-	3.05	0.7586	0.2235	23.45
Corvidae	*Corvus monedula*	Palearctic	0	-0.9256	-	4.60	0.8885	0.1395	0.09
Corvidae	*Corvus moneduloides*	Palearctic	0	-0.9051	-	4.62	0.8768	0.2239	3.67
Corvidae	*Corvus nasicus*	Australasia	0	-0.6052	-	3.26	0.8257	0.5476	4.40
Corvidae	*Corvus orru*	Afrotropics	0	0.2860	16.2224	3.55	0.7749	0.8077	78.92
Corvidae	*Corvus ossifragus*	Nearctic	0	-0.6930	-	4.17	0.7887	0.6405	70.51
Corvidae	*Corvus palmarum*	Palearctic	0	-1.0018	-	4.08	0.8328	0.4608	22.42
Corvidae	*Corvus rhipidurus*	Afrotropics	0	0.1518	0.0000	3.41	0.7875	0.7379	34.25
Corvidae	*Corvus ruficollis*	IndoMalay	0	-0.0285	-	2.79	0.7953	0.6204	19.04
Corvidae	*Corvus sinaloae*	Australasia	1	0.2139	3.9745	2.62	0.5985	0.0000	0.00
Corvidae	*Corvus splendens*	Palearctic	0	-0.7995	-	4.45	0.8643	0.1990	1.47
Corvidae	*Corvus tasmanicus*	Australasia	0	-0.0308	0.9796	2.55	0.6478	0.3519	36.25
Corvidae	*Corvus tasmanicus*	Australasia	0	-0.0308	0.9796	2.55	0.6478	-	-
Corvidae	*Corvus tristis*	Nearctic	0	-0.3321	-	3.53	0.7136	0.4046	86.87
Corvidae	*Corvus typicus*	Neotropics	0	-0.2874	-	2.47	0.5985	0.0000	0.00
Corvidae	*Corvus unicolor*	Neotropics	1	-0.1438	4.1933	2.46	0.6906	0.2797	2.64
Corvidae	*Corvus validus*	IndoMalay	0	-0.1334	-	3.49	0.8237	0.5288	20.25
Corvidae	*Corvus woodfordi*	Palearctic	0	-0.4847	-	3.61	0.8474	0.5975	43.39
Corvidae	*Crypsirina cucullata*	Neotropics	0	-2.4717	4.0515	4.08	0.7234	0.1976	31.21
Corvidae	*Crypsirina temia*	Neotropics	0	0.1098	7.3607	3.52	0.7340	0.2440	87.31
Corvidae	*Cyanocitta cristata*	IndoMalay	0	0.0232	-	2.85	0.7870	0.5871	15.02
Corvidae	*Cyanocitta stelleri*	IndoMalay	0	-0.1096	-	3.35	0.8141	0.7060	20.03
Corvidae	*Cyanocorax affinis*	Neotropics	0	-0.7095	-	2.23	0.7683	0.1918	89.02
Corvidae	*Cyanocorax beecheii*	Neotropics	1	-0.0313	1.8992	3.16	0.6562	0.0702	59.94
Corvidae	*Cyanocorax caeruleus*	IndoMalay	0	-0.0842	-	3.47	0.8077	0.7985	72.54
Corvidae	*Cyanocorax cayanus*	Palearctic	0	-0.5694	3.4893	3.98	0.8278	0.2876	13.16
Corvidae	*Cyanocorax chrysops*	Afrotropics	0	-0.3728	-	3.86	0.7912	0.4548	47.09
Corvidae	*Cyanocorax cristatellus*	Afrotropics	0	0.1238	3.5718	3.43	0.7748	0.4444	41.46
Corvidae	*Cyanocorax cyanomelas*	Afrotropics	0	-0.0600	8.2080	3.64	0.8501	0.5021	41.95
Corvidae	*Cyanocorax cyanopogon*	IndoMalay	0	-0.0423	0.0000	2.86	0.6784	0.2107	74.99
Corvidae	*Cyanocorax dickeyi*	IndoMalay	0	-0.0254	9.9131	3.45	0.7839	0.4252	30.37
Corvidae	*Cyanocorax heilprini*	Neotropics	0	0.0224	-	3.98	0.8363	0.7573	7.35
Corvidae	*Cyanocorax melanocyaneus*	Neotropics	0	-0.1157	-	3.91	0.8426	0.4481	33.53
Corvidae	*Cyanocorax mystacalis*	Neotropics	0	0.0116	-	3.29	0.7432	0.2543	37.92
Corvidae	*Cyanocorax sanblasianus*	Neotropics	0	-0.0025	-	4.06	0.8114	0.8418	24.25
Corvidae	*Cyanocorax violaceus*	Neotropics	0	-0.1336	-	3.99	0.7552	0.7586	8.82
Corvidae	*Cyanocorax yncas*	Afrotropics	1	-4.1716	7.2271	3.57	0.7512	0.8112	89.77
Corvidae	*Cyanocorax yucatanicus*	Nearctic	0	-0.7165	-	4.26	0.8365	0.5441	10.67
Corvidae	*Cyanolyca argentigula*	Neotropics	0	-0.0303	-	3.37	0.7586	0.5310	18.24
Corvidae	*Cyanolyca armillata*	Nearctic	0	-0.0871	-	3.82	0.7112	0.1732	55.72
Corvidae	*Cyanolyca cucullata*	Neotropics	0	-0.4757	3.4293	2.73	0.7632	0.5898	39.08
Corvidae	*Cyanolyca mirabilis*	Neotropics	0	-0.0868	-	4.09	0.7852	0.6649	34.18
Corvidae	*Cyanolyca nana*	Neotropics	0	-0.3521	-	3.50	0.8134	0.7260	62.24
Corvidae	*Cyanolyca pulchra*	Neotropics	0	-0.2690	4.5556	3.96	0.8128	0.9014	5.74
Corvidae	*Cyanolyca pumilo*	Neotropics	0	-0.3356	-	3.81	0.7971	0.6207	43.82

Family	Species	Realm	Threat	Latitude	Elevation	Anomaly	Size	Shape	Orientation
Corvidae	*Cyanolyca turcosa*	Neotropics	0	-0.1340	-	3.77	0.8136	0.7907	53.40
Corvidae	*Cyanolyca viridicyanus*	Nearctic	0	0.0824	4.9006	3.87	0.6859	0.4384	61.42
Corvidae	*Cyanopica cyanus*	Neotropics	0	0.9372	-	3.80	0.7245	0.5007	66.44
Corvidae	*Dendrocitta formosae*	IndoMalay	1	0.0048	3.7042	2.63	0.6478	0.3503	71.22
Corvidae	*Dendrocitta frontalis*	Australasia	1	-0.5461	3.4884	3.02	0.6921	0.1350	65.12
Corvidae	*Dendrocitta leucogastra*	Australasia	0	-0.5360	-	2.29	0.6690	0.2652	30.45
Corvidae	*Dendrocitta occipitalis*	Australasia	1	-0.7465	-	2.22	0.6120	0.1232	13.29
Corvidae	*Dendrocitta vagabunda*	Neotropics	0	0.0406	6.2490	4.00	0.8290	0.6738	8.97
Corvidae	*Garrulus glandarius*	IndoMalay	0	-0.3728	6.0660	3.33	0.7746	0.7391	0.23
Corvidae	*Garrulus lanceolatus*	Palearctic	0	-4.0355	11.1150	3.97	0.7730	0.7295	40.42
Corvidae	*Gymnorhinus cyanocephalus*	Palearctic	1	-0.8589	-	4.38	0.7765	0.4064	4.51
Corvidae	*Nucifraga caryocatactes*	Afrotropics	0	-0.0809	-	3.18	0.7613	0.3878	70.13
Corvidae	*Nucifraga columbiana*	Afrotropics	1	-0.1326	3.5927	3.08	0.7172	0.1361	73.88
Corvidae	*Perisoreus canadensis*	Nearctic	0	-0.3217	14.1964	3.87	0.8227	0.1951	44.70
Corvidae	*Perisoreus infaustus*	Neotropics	0	-0.0951	4.3235	3.49	0.7823	0.2742	29.28
Corvidae	*Perisoreus internigrans*	Neotropics	0	0.0931	2.4069	2.97	0.7078	0.1609	68.12
Corvidae	*Pica nuttalli*	Neotropics	0	0.0101	9.4217	3.56	0.7485	0.3462	84.12
Corvidae	*Pica pica*	Neotropics	0	-0.2989	8.0580	3.56	0.8183	0.4865	53.24
Corvidae	*Platylophus galericulatus*	Nearctic	0	-0.5774	10.8944	4.13	0.8388	0.5126	7.68
Corvidae	*Platysmurus leucopterus*	Neotropics	0	0.0034	-	3.94	0.8102	0.9682	63.28
Corvidae	*Podoces hendersoni*	Afrotropics	0	1.9863	0.0000	3.31	0.7677	0.6140	17.70
Corvidae	*Podoces panderi*	Afrotropics	1	6.2816	3.9606	3.23	0.6302	0.4924	53.13
Corvidae	*Podoces pleskei*	Afrotropics	0	-0.0245	13.8376	3.53	0.8212	0.8422	24.24
Corvidae	*Psilorhinus morio*	Afrotropics	0	-0.0914	-	3.59	0.8500	0.6517	34.00
Corvidae	*Ptilostomus afer*	IndoMalay	0	0.0740	-	2.83	0.7829	0.4361	1.29
Corvidae	*Pyrrhocorax graculus*	Neotropics	0	-0.4822	4.5158	2.76	0.6906	0.6941	68.19
Corvidae	*Pyrrhocorax pyrrhocorax*	Neotropics	0	-0.4470	9.3397	2.98	0.7128	0.1514	79.85
Corvidae	*Temnurus temnurus*	Neotropics	0	-0.0100	-	4.04	0.8255	0.7388	20.70
Corvidae	*Urocissa caerulea*	Neotropics	0	0.0305	5.3352	4.00	0.8358	0.7858	10.91
Corvidae	*Urocissa erythrorhyncha*	Neotropics	0	0.2802	2.3394	3.09	0.6961	0.3102	83.69
Corvidae	*Urocissa flavirostris*	Neotropics	0	-0.0584	14.1897	3.75	0.7650	0.2123	30.78
Corvidae	*Urocissa ornata*	Neotropics	0	0.0482	13.7566	3.79	0.7851	0.6011	87.43
Corvidae	*Urocissa whiteheadi*	Neotropics	0	0.3031	3.2845	4.03	0.8249	0.5658	7.66
Corvidae	*Zavattariornis stresemanni*	Neotropics	0	7.4025	3.6874	3.16	0.7019	0.1262	65.27
Cotingidae	*Ampelioides tschudii*	Neotropics	0	0.0141	10.2349	3.63	0.7560	0.3876	77.65
Cotingidae	*Ampelion rubrocristatus*	Neotropics	0	0.0408	8.2754	3.65	0.7715	0.4143	82.65
Cotingidae	*Ampelion rufaxilla*	Neotropics	0	-0.2655	5.9103	3.63	0.7340	0.3448	64.74
Cotingidae	*Calyptura cristata*	Neotropics	0	0.7168	7.0509	3.66	0.6974	0.3212	23.27
Cotingidae	*Carpodectes antoniae*	Nearctic	0	-0.6907	-	4.04	0.8280	0.2405	7.88
Cotingidae	*Carpodectes hopkei*	Palearctic	0	-1.0509	12.5099	4.39	0.8155	0.2600	20.37
Cotingidae	*Carpodectes nitidus*	Palearctic	0	11.9634	5.6915	4.22	0.7344	0.4218	7.38
Cotingidae	*Carpornis cucullata*	IndoMalay	0	0.0370	14.4029	4.11	0.7428	0.1397	16.63
Cotingidae	*Carpornis melanocephala*	Palearctic	0	-7.2946	9.3110	4.27	0.7281	0.1300	22.80
Cotingidae	*Cephalopterus glabricollis*	Afrotropics	0	-0.0303	-	3.47	0.8442	0.4975	32.32
Cotingidae	*Cephalopterus ornatus*	Afrotropics	0	0.1531	-	3.14	0.7721	0.0926	7.74
Cotingidae	*Cephalopterus penduliger*	IndoMalay	0	-0.0460	-	2.62	0.7019	0.6235	86.79
Cotingidae	*Conioptilon mcilhennyi*	Palearctic	0	-1.0505	18.1978	4.40	0.8509	0.4009	10.57
Cotingidae	*Cotinga amabilis*	Neotropics	0	-0.0601	3.7813	4.03	0.8351	0.7060	3.99
Cotingidae	*Cotinga cayana*	Neotropics	0	-0.5700	-	2.49	0.8001	0.4392	67.85
Cotingidae	*Cotinga cotinga*	Afrotropics	0	-0.0607	-	3.31	0.7893	0.4940	55.27
Cotingidae	*Cotinga maculata*	Afrotropics	0	-0.6602	-	3.31	0.7485	0.7859	89.16
Cotingidae	*Cotinga maynana*	Afrotropics	0	-0.1049	-	3.47	0.7933	0.1088	6.44
Cotingidae	*Cotinga nattererii*	Afrotropics	0	-0.1252	-	3.39	0.7323	0.4663	80.88
Cotingidae	*Cotinga ridgwayi*	Afrotropics	0	7.8999	15.6172	3.45	0.7112	0.4351	83.62
Cotingidae	*Doliornis remseni*	Afrotropics	0	0.0417	-	3.35	0.8077	0.6775	53.61
Cotingidae	*Doliornis sclateri*	IndoMalay	0	-0.1859	-	3.47	0.8151	0.7726	6.85
Cotingidae	*Gymnoderus foetidus*	Australasia	0	-0.4690	-	3.34	0.8352	0.5875	7.98
Cotingidae	*Haematoderus militaris*	Afrotropics	0	0.1471	-	3.15	0.7914	0.3037	20.69
Cotingidae	*Iodopleura fusca*	Neotropics	0	-0.3198	5.8037	3.52	0.8345	0.5163	43.36
Cotingidae	*Iodopleura isabellae*	Nearctic	0	-0.9182	5.3896	4.23	0.8271	0.6788	18.84
Cotingidae	*Iodopleura pipra*	Neotropics	0	-0.0365	9.5322	3.80	0.7760	0.2150	33.43
Cotingidae	*Laniisoma elegans*	Neotropics	0	0.0402	-	4.07	0.8175	0.4667	23.26
Cotingidae	*Laniocera hypopyrra*	Afrotropics	0	-0.1788	-	3.67	0.8418	0.6467	39.73
Cotingidae	*Laniocera rufescens*	Afrotropics	0	0.0720	-	3.48	0.8254	0.1896	4.82
Cotingidae	*Lipaugus fuscocinereus*	Neotropics	0	-0.2690	6.0533	3.00	0.7704	0.2801	40.02
Cotingidae	*Lipaugus lanioides*	Neotropics	0	0.0227	11.2276	3.12	0.7435	0.4944	75.68
Cotingidae	*Lipaugus streptophorus*	Neotropics	0	0.0726	1.4681	3.98	0.8212	0.7636	3.20
Cotingidae	*Lipaugus unirufus*	Neotropics	0	0.1208	2.3131	3.15	0.7446	0.4416	46.18
Cotingidae	*Lipaugus uropygialis*	Australasia	0	-0.5234	-	3.17	0.7606	0.3304	78.00
Cotingidae	*Lipaugus vociferans*	Palearctic	0	-1.1369	-	4.53	0.8139	0.1754	11.34
Cotingidae	*Lipaugus weberi*	Nearctic	0	2.6305	6.9179	4.40	0.7277	0.7799	46.97
Cotingidae	*Oxyruncus cristatus*	IndoMalay	0	-0.0729	-	3.17	0.7806	0.4402	35.66
Cotingidae	*Pachyramphus aglaiae*	Afrotropics	0	-0.1928	0.0000	3.65	0.8279	0.5961	50.23
Cotingidae	*Pachyramphus albogriseus*	Afrotropics	0	0.0063	-	3.65	0.7871	0.6913	63.41

Family	Species	Realm	Threat	Latitude	Elevation	Anomaly	Size	Shape	Orientation
Cotingidae	*Pachyramphus castaneus*	Afrotropics	0	-0.0119	8.9510	3.61	0.8153	0.2438	10.38
Cotingidae	*Pachyramphus cinnamomeus*	Neotropics	0	-0.1823	4.5086	3.86	0.8512	0.5999	36.71
Cotingidae	*Pachyramphus homochrous*	Neotropics	0	-3.3857	-	3.72	0.7413	0.2571	54.72
Cotingidae	*Pachyramphus major*	Neotropics	0	0.0484	-	3.80	0.8117	0.5353	10.13
Cotingidae	*Pachyramphus marginatus*	Neotropics	0	0.0015	2.4997	3.29	0.7653	0.2808	48.41
Cotingidae	*Pachyramphus minor*	Neotropics	0	-0.1632	4.5586	3.69	0.7957	0.6655	71.26
Cotingidae	*Pachyramphus niger*	Neotropics	0	-0.0053	-	3.31	0.6935	0.3729	71.93
Cotingidae	*Pachyramphus polychopterus*	Australasia	0	-0.0820	5.0674	2.78	0.7624	0.1936	22.77
Cotingidae	*Pachyramphus rufus*	Palearctic	1	-0.8277	-	4.53	0.8309	0.1409	1.76
Cotingidae	*Pachyramphus spodiurus*	Australasia	1	0.2833	2.4178	2.63	0.5788	0.0000	0.00
Cotingidae	*Pachyramphus surinamus*	IndoMalay	1	-0.4671	5.6698	2.72	0.6935	0.1056	11.26
Cotingidae	*Pachyramphus validus*	IndoMalay	0	-0.5266	-	3.40	0.8502	0.5765	21.50
Cotingidae	*Pachyramphus versicolor*	IndoMalay	0	0.0400	6.9508	2.80	0.7234	0.4515	4.44
Cotingidae	*Pachyramphus viridis*	Palearctic	0	-0.8411	5.1127	4.20	0.8043	0.4751	14.02
Cotingidae	*Perissocephalus tricolor*	Neotropics	0	-0.2325	3.5228	2.50	0.7217	0.1682	18.06
Cotingidae	*Phibalura flavirostris*	Neotropics	0	-0.2806	5.0949	3.19	0.7526	0.3862	58.36
Cotingidae	*Phoenicircus carnifex*	Neotropics	0	0.1582	-	4.20	0.7959	0.4759	4.72
Cotingidae	*Phoenicircus nigricollis*	Neotropics	0	-0.2505	8.9009	3.71	0.8199	0.4284	31.71
Cotingidae	*Phytotoma raimondii*	Afrotropics	0	0.0033	-	3.29	0.7931	0.9269	57.89
Cotingidae	*Phytotoma rara*	Afrotropics	0	-0.1898	-	3.57	0.8097	0.5930	60.09
Cotingidae	*Phytotoma rutila*	Afrotropics	0	0.2894	-	3.28	0.7837	0.2719	10.01
Cotingidae	*Pipreola arcuata*	Nearctic	0	-0.6565	-	3.59	0.7660	0.4070	85.85
Cotingidae	*Pipreola aureopectus*	Nearctic	0	-0.8764	9.6422	4.18	0.8384	0.1958	9.43
Cotingidae	*Pipreola chlorolepidota*	Nearctic	0	-0.4210	7.0277	4.02	0.7528	0.1800	55.23
Cotingidae	*Pipreola formosa*	Nearctic	1	-0.7844	0.9628	3.82	0.7828	0.4368	4.48
Cotingidae	*Pipreola frontalis*	Nearctic	0	-0.9192	11.7851	4.27	0.8416	0.2570	8.71
Cotingidae	*Pipreola intermedia*	Neotropics	0	-0.1578	11.6489	3.54	0.7959	0.3494	50.71
Cotingidae	*Pipreola jucunda*	Nearctic	0	-0.5241	5.2653	3.34	0.7310	0.2928	54.58
Cotingidae	*Pipreola lubomirskii*	Nearctic	0	-0.7209	8.3159	4.17	0.8533	0.3478	15.13
Cotingidae	*Pipreola pulchra*	Nearctic	0	-0.2573	-	3.94	0.8124	0.5100	39.45
Cotingidae	*Pipreola riefferii*	Neotropics	0	0.0000	16.1909	3.78	0.6598	0.2688	0.00
Cotingidae	*Pipreola whitelyi*	Palearctic	0	-0.9720	14.4565	4.98	0.8577	0.1883	0.01
Cotingidae	*Porphyrolaema porphyrolaema*	Australasia	0	-0.3919	0.0000	3.39	0.7762	0.3198	51.93
Cotingidae	*Procnias albus*	IndoMalay	0	0.1415	3.5873	2.77	0.6784	0.5917	79.40
Cotingidae	*Procnias averano*	IndoMalay	0	-0.0174	-	2.82	0.7846	0.5644	2.64
Cotingidae	*Procnias nudicollis*	IndoMalay	0	-0.7610	6.2354	2.61	0.6562	0.4622	64.29
Cotingidae	*Procnias tricarunculatus*	IndoMalay	0	-0.0209	-	3.19	0.7780	0.4919	39.86
Cotingidae	*Pyroderus scutatus*	Afrotropics	0	0.1126	-	3.30	0.8235	0.3102	16.68
Cotingidae	*Querula purpurata*	Australasia	0	-0.0246	-	2.71	0.7165	0.5271	59.13
Cotingidae	*Rupicola peruvianus*	Neotropics	0	-0.5657	-	2.69	0.7378	0.1841	38.83
Cotingidae	*Rupicola rupicola*	Neotropics	1	0.5087	14.4421	3.18	0.6120	0.6246	0.00
Cotingidae	*Schiffornis major*	Australasia	0	0.0435	-	2.48	0.6302	0.2130	7.47
Cotingidae	*Schiffornis turdina*	Australasia	0	0.7871	-	2.63	0.6478	0.0000	0.00
Cotingidae	*Schiffornis virescens*	Australasia	0	-0.0759	-	2.48	0.6120	0.0400	36.10
Cotingidae	*Snowornis cryptolophus*	Palearctic	0	9.7520	5.8272	3.49	0.6997	0.1929	31.04
Cotingidae	*Snowornis subalaris*	IndoMalay	0	0.0311	-	3.27	0.7876	0.3443	34.53
Cotingidae	*Tijuca atra*	Afrotropics	0	-0.0078	9.8957	3.29	0.7828	0.4834	1.36
Cotingidae	*Tijuca condita*	Afrotropics	0	9.6220	10.7472	3.38	0.7286	0.3793	24.67
Cotingidae	*Tityra cayana*	Australasia	0	0.0392	-	2.48	0.6302	0.2130	7.47
Cotingidae	*Tityra inquisitor*	Afrotropics	0	-0.0546	12.1732	3.59	0.8555	0.7182	30.56
Cotingidae	*Tityra semifasciata*	Neotropics	0	0.0101	3.3096	3.97	0.8394	0.6018	27.17
Cotingidae	*Xenopsaris albinucha*	Nearctic	0	-0.5294	15.2801	4.09	0.7970	0.4488	58.57
Cotingidae	*Xipholena atropurpurea*	Neotropics	0	-0.2769	9.3318	4.20	0.6906	0.2157	33.49
Cotingidae	*Xipholena lamellipennis*	Neotropics	0	-0.4040	11.6982	3.05	0.8023	0.1957	85.05
Cotingidae	*Xipholena punicea*	Neotropics	0	-0.8642	12.1697	4.07	0.7667	0.2228	42.73
Cotingidae	*Zaratornis stresemanni*	Neotropics	0	-3.9018	6.0376	3.38	0.7030	0.2577	78.55
Cracidae	*Aburria aburri*	Neotropics	0	0.1187	7.2558	3.69	0.7482	0.3257	85.17
Cracidae	*Chamaepetes goudotii*	Neotropics	0	-0.6228	5.9029	3.62	0.8281	0.8510	43.67
Cracidae	*Chamaepetes unicolor*	Nearctic	0	-0.8467	10.2484	4.30	0.8325	0.6434	5.80
Cracidae	*Crax alberti*	Neotropics	0	0.6701	15.2928	3.71	0.7158	0.2558	67.61
Cracidae	*Crax alector*	Neotropics	0	-2.3879	9.4089	3.75	0.7519	0.3765	67.94
Cracidae	*Crax blumenbachii*	Neotropics	0	0.4455	7.5737	4.44	0.7144	0.4895	5.76
Cracidae	*Crax daubentoni*	Neotropics	0	-0.0877	8.9341	3.05	0.7305	0.5671	66.78
Cracidae	*Crax fasciolata*	Neotropics	0	0.1597	-	4.07	0.8235	0.7430	17.95
Cracidae	*Crax globulosa*	Neotropics	0	-0.0162	8.5798	3.32	0.6120	0.9804	90.00
Cracidae	*Crax rubra*	Neotropics	1	0.0000	8.9945	4.05	0.6428	0.4087	32.48
Cracidae	*Mitu salvini*	IndoMalay	0	-0.8024	2.8826	3.25	0.8066	0.3891	13.59
Cracidae	*Mitu tomentosum*	Neotropics	0	0.0369	-	2.46	0.6631	0.6520	9.45
Cracidae	*Mitu tuberosum*	IndoMalay	0	-0.0424	6.1129	2.62	0.7008	0.6308	85.20
Cracidae	*Nothocrax urumutum*	Australasia	0	-0.0399	1.9546	2.48	0.6120	0.0400	36.10
Cracidae	*Oreophasis derbianus*	Neotropics	0	0.0631	1.4083	3.89	0.7572	0.6089	51.23
Cracidae	*Ortalis canicollis*	Neotropics	0	-0.0528	3.0305	3.37	0.7681	0.4668	38.34
Cracidae	*Ortalis cinereiceps*	Neotropics	0	-0.1748	2.0682	3.34	0.7371	0.7798	15.45
Cracidae	*Ortalis columbiana*	Afrotropics	0	0.0739	6.7671	3.60	0.7340	0.3868	85.60

Family	Species	Realm	Threat	Latitude	Elevation	Anomaly	Size	Shape	Orientation
Cracidae	*Ortalis erythroptera*	Afrotropics	0	0.1827	0.0000	3.52	0.7629	0.5979	11.95
Cracidae	*Ortalis guttata*	Afrotropics	0	0.0477	-	3.26	0.8103	0.2555	13.55
Cracidae	*Ortalis leucogastra*	Afrotropics	0	-0.1649	-	3.56	0.8313	0.6290	48.26
Cracidae	*Ortalis motmot*	Afrotropics	0	-0.2423	-	3.77	0.7839	0.3126	56.34
Cracidae	*Ortalis poliocephala*	Afrotropics	0	0.1708	-	3.41	0.7721	0.6014	40.69
Cracidae	*Ortalis ruficauda*	Afrotropics	0	0.0449	16.9179	3.44	0.7725	0.3874	72.08
Cracidae	*Ortalis superciliaris*	Palearctic	0	-0.3607	14.7107	3.90	0.7702	0.3144	43.83
Cracidae	*Ortalis vetula*	Afrotropics	0	-0.0825	11.0258	3.31	0.7672	0.4213	21.60
Cracidae	*Ortalis wagleri*	Neotropics	0	-0.1183	3.4410	3.88	0.8381	0.5051	34.71
Cracidae	*Pauxi pauxi*	Palearctic	0	-0.7773	6.4180	4.77	0.8458	0.1879	5.08
Cracidae	*Pauxi unicornis*	Palearctic	0	-1.5348	7.0528	3.59	0.7234	0.6040	21.96
Cracidae	*Penelope albipennis*	Palearctic	0	-0.3545	6.2609	4.10	0.8455	0.1127	1.52
Cracidae	*Penelope argyrotis*	Palearctic	0	-0.6188	10.1840	4.07	0.7858	0.4149	13.62
Cracidae	*Penelope barbata*	Nearctic	0	-0.5489	7.2485	3.28	0.7851	0.4171	36.57
Cracidae	*Penelope dabbenei*	Afrotropics	0	-0.2086	7.9504	3.70	0.8194	0.5548	3.39
Cracidae	*Penelope jacquacu*	Palearctic	0	-0.8852	14.8651	4.34	0.7796	0.6904	18.88
Cracidae	*Penelope jacucaca*	Nearctic	0	-0.3388	9.3502	3.88	0.7510	0.2323	49.16
Cracidae	*Penelope marail*	IndoMalay	0	-0.0035	3.0750	2.65	0.7019	0.2028	68.31
Cracidae	*Penelope montagnii*	IndoMalay	0	-0.0169	12.1619	3.26	0.7963	0.4584	0.97
Cracidae	*Penelope obscura*	Palearctic	0	4.5623	6.2238	4.00	0.7642	0.8273	28.56
Cracidae	*Penelope ochrogaster*	Afrotropics	0	0.0560	8.3636	3.33	0.7822	0.4014	58.81
Cracidae	*Penelope ortoni*	Palearctic	0	-0.8663	4.1037	3.36	0.7611	0.5021	22.84
Cracidae	*Penelope perspicax*	Palearctic	0	-0.6597	11.5538	3.49	0.7969	0.7056	52.37
Cracidae	*Penelope pileata*	IndoMalay	0	-0.1048	8.7306	3.47	0.7880	0.6862	66.88
Cracidae	*Penelope purpurascens*	Palearctic	0	-1.0022	-	4.28	0.7966	0.1908	1.54
Cracidae	*Penelope superciliaris*	Afrotropics	0	0.0286	-	3.36	0.7743	0.4166	26.42
Cracidae	*Penelopina nigra*	Afrotropics	0	0.0144	-	3.23	0.7857	0.6823	30.77
Cracidae	*Pipile cujubi*	Palearctic	0	-12.0209	3.6689	4.48	0.7344	0.4478	12.00
Cracidae	*Pipile cumanensis*	Palearctic	0	-0.7973	-	4.27	0.7826	0.3033	35.22
Cracidae	*Pipile jacutinga*	IndoMalay	0	-0.0186	-	2.75	0.7712	0.5489	30.55
Cracidae	*Pipile pipile*	Palearctic	0	-0.5168	9.6819	4.65	0.8539	0.2659	3.23
Cracticidae	*Cracticus cassicus*	Palearctic	1	-0.4485	-	4.38	0.7272	0.1470	15.89
Cracticidae	*Cracticus mentalis*	Neotropics	0	-0.5665	-	2.67	0.7943	0.5946	44.30
Cracticidae	*Cracticus nigrogularis*	Nearctic	0	-0.7140	2.7934	4.55	0.8209	0.2935	12.24
Cracticidae	*Cracticus quoyi*	Afrotropics	0	-0.2692	6.4985	3.25	0.8568	0.3197	3.36
Cracticidae	*Cracticus torquatus*	IndoMalay	0	-0.1494	-	3.39	0.8142	0.6438	9.67
Cracticidae	*Gymnorhina tibicen*	Palearctic	0	-0.8783	-	5.01	0.8586	0.1937	1.30
Cracticidae	*Peltops blainvillii*	Afrotropics	0	-0.3087	4.7106	3.81	0.8104	0.6624	0.32
Cracticidae	*Peltops montanus*	IndoMalay	1	-0.0820	2.2655	3.57	0.7165	0.3570	69.90
Cracticidae	*Strepera graculina*	Nearctic	0	-1.0775	-	5.20	0.7636	0.4686	22.52
Cracticidae	*Strepera versicolor*	Nearctic	0	-0.2459	-	6.26	0.8219	0.0456	0.29
Cuculidae	*Cacomantis castaneiventris*	Australasia	1	-0.0978	3.3772	2.68	0.7314	0.6126	89.45
Cuculidae	*Cacomantis flabelliformis*	Australasia	0	-0.5689	-	2.89	0.7687	0.7592	17.07
Cuculidae	*Cacomantis heinrichi*	Neotropics	0	-0.0126	-	3.98	0.8433	0.6798	28.03
Cuculidae	*Cacomantis merulinus*	Neotropics	0	0.0408	8.6062	3.65	0.7574	0.3723	73.89
Cuculidae	*Cacomantis passerinus*	Neotropics	0	-0.4768	4.8573	3.09	0.7992	0.9241	26.43
Cuculidae	*Cacomantis sepulcralis*	Neotropics	0	-0.1439	-	3.89	0.8409	0.6174	44.32
Cuculidae	*Cacomantis sonneratii*	Neotropics	1	0.0000	1.6092	4.14	0.5985	0.5771	46.13
Cuculidae	*Cacomantis variolosus*	Neotropics	0	-0.1084	-	3.65	0.7446	0.2154	37.17
Cuculidae	*Caliechthrus leucolophus*	Palearctic	0	-0.9491	0.7315	5.53	0.8086	0.0667	0.98
Cuculidae	*Carpococcyx radiatus*	Nearctic	0	-0.2328	-	3.95	0.8237	0.2455	40.54
Cuculidae	*Carpococcyx renauldi*	Neotropics	1	0.1842	1.7382	3.07	0.6662	0.3892	89.81
Cuculidae	*Carpococcyx viridis*	Palearctic	0	-0.7510	-	3.72	0.8335	0.3747	29.90
Cuculidae	*Centropus anselli*	Nearctic	0	-0.6241	-	4.08	0.8243	0.4397	52.61
Cuculidae	*Centropus ateralbus*	Nearctic	0	-0.6024	-	4.01	0.8116	0.4287	21.77
Cuculidae	*Centropus bengalensis*	Palearctic	1	-4.0310	11.2559	4.40	0.7087	0.1915	37.30
Cuculidae	*Centropus bernsteini*	IndoMalay	0	-0.1328	-	3.15	0.8340	0.6852	26.05
Cuculidae	*Centropus celebensis*	Neotropics	0	-0.0376	-	3.37	0.7570	0.4410	33.42
Cuculidae	*Centropus chlororhynchus*	Neotropics	0	-0.0278	3.0497	4.04	0.8348	0.7954	3.05
Cuculidae	*Centropus cupreicaudus*	Neotropics	0	-0.2447	-	3.57	0.8191	0.5165	62.88
Cuculidae	*Centropus goliath*	Neotropics	0	-0.1077	-	4.02	0.8340	0.7038	18.78
Cuculidae	*Centropus grillii*	Neotropics	0	0.0913	4.0776	4.09	0.8220	0.7676	12.73
Cuculidae	*Centropus leucogaster*	Neotropics	0	0.1049	3.0749	3.17	0.7507	0.8361	80.33
Cuculidae	*Centropus melanops*	Neotropics	0	-0.3050	-	3.85	0.7772	0.5239	54.62
Cuculidae	*Centropus menbeki*	Neotropics	1	-0.0210	0.0000	3.99	0.7764	0.3594	60.77
Cuculidae	*Centropus milo*	Neotropics	0	-4.5060	1.5585	3.99	0.7674	0.1915	50.35
Cuculidae	*Centropus monachus*	Neotropics	0	-0.0766	2.7227	4.06	0.8327	0.7629	4.82
Cuculidae	*Centropus nigrorufus*	Neotropics	0	-0.1137	-	3.90	0.7955	0.4365	32.57
Cuculidae	*Centropus phasianinus*	Nearctic	1	15.4395	1.9727	4.66	0.6740	0.5092	1.55
Cuculidae	*Centropus rectunguis*	Nearctic	0	-1.0869	-	4.30	0.7926	0.6412	20.61
Cuculidae	*Centropus senegalensis*	Afrotropics	0	-0.0204	-	3.24	0.7840	0.7018	1.21
Cuculidae	*Centropus sinensis*	Australasia	0	-0.1231	4.0444	2.50	0.6716	0.2680	24.42
Cuculidae	*Centropus steerii*	IndoMalay	0	-0.2340	-	3.05	0.8322	0.7192	45.70
Cuculidae	*Centropus superciliosus*	Australasia	0	-0.1060	0.9970	2.77	0.7631	0.4038	22.50

Family	Species	Realm	Threat	Latitude	Elevation	Anomaly	Size	Shape	Orientation
Cuculidae	*Centropus toulou*	Australasia	0	-0.0635	-	2.71	0.7185	0.5623	60.71
Cuculidae	*Centropus unirufus*	IndoMalay	1	0.2892	2.4759	2.76	0.6302	0.6063	90.00
Cuculidae	*Centropus violaceus*	Afrotropics	0	-1.6103	-	3.79	0.8009	0.5697	13.96
Cuculidae	*Centropus viridis*	Australasia	0	-1.6103	-	2.48	0.6302	0.2423	87.23
Cuculidae	*Cercococcyx mechowi*	Australasia	0	-0.3537	-	3.27	0.8115	0.6863	22.50
Cuculidae	*Cercococcyx montanus*	IndoMalay	1	0.0399	3.1400	2.84	0.7863	0.4293	0.00
Cuculidae	*Cercococcyx olivinus*	Afrotropics	0	-0.1416	-	3.50	0.8472	0.5560	25.54
Cuculidae	*Ceuthmochares aereus*	Australasia	0	-0.1249	-	3.32	0.7840	0.3106	2.45
Cuculidae	*Chrysococcyx basalis*	Neotropics	0	-0.1252	11.7428	3.69	0.7104	0.2048	59.92
Cuculidae	*Chrysococcyx caprius*	Neotropics	0	-0.2418	-	2.53	0.6986	0.2608	17.52
Cuculidae	*Chrysococcyx caprius*	Neotropics	0	-0.2418	-	2.53	0.6986	-	-
Cuculidae	*Chrysococcyx crassirostris*	Neotropics	0	0.0299	9.8562	3.37	0.6631	0.7524	1.64
Cuculidae	*Chrysococcyx cupreus*	Neotropics	0	0.0679	10.6713	4.05	0.6804	0.3906	34.57
Cuculidae	*Chrysococcyx flavigularis*	Neotropics	0	-0.0317	9.7317	2.46	0.6876	0.3855	3.42
Cuculidae	*Chrysococcyx klaas*	Neotropics	0	0.0323	4.1361	3.68	0.7522	0.1572	61.83
Cuculidae	*Chrysococcyx lucidus*	Neotropics	0	0.1347	3.3840	3.01	0.7144	0.2797	69.95
Cuculidae	*Chrysococcyx maculatus*	Neotropics	0	0.2777	7.7322	3.02	0.7040	0.1407	67.14
Cuculidae	*Chrysococcyx meyeri*	Nearctic	0	-0.8032	7.3132	4.28	0.8320	0.6088	13.83
Cuculidae	*Chrysococcyx minutillus*	Neotropics	0	-0.1260	6.5137	3.85	0.8471	0.4264	35.49
Cuculidae	*Chrysococcyx osculans*	Neotropics	1	-0.3091	0.6295	2.53	0.6974	0.1632	17.43
Cuculidae	*Chrysococcyx ruficollis*	Nearctic	0	-0.2278	3.9089	3.82	0.8064	0.2030	31.26
Cuculidae	*Chrysococcyx russatus*	Neotropics	0	-0.2561	-	2.50	0.7228	0.2094	19.58
Cuculidae	*Chrysococcyx xanthorhynchus*	Nearctic	0	-0.5534	7.7577	4.14	0.8543	0.5794	17.94
Cuculidae	*Clamator coromandus*	Afrotropics	0	-0.2601	0.1661	3.09	0.6120	0.4082	71.57
Cuculidae	*Clamator glandarius*	Afrotropics	0	0.0544	8.5703	3.56	0.7976	0.5138	47.39
Cuculidae	*Clamator jacobinus*	Afrotropics	0	2.6130	4.8749	3.51	0.7916	0.5707	6.93
Cuculidae	*Clamator levaillantii*	Afrotropics	0	-0.0927	-	4.02	0.8137	0.8570	53.78
Cuculidae	*Coccycua cinerea*	Neotropics	0	-0.3248	1.5184	2.90	0.7531	0.1851	47.46
Cuculidae	*Coccycua minuta*	Australasia	0	-0.0672	5.9523	2.81	0.7399	0.1467	24.11
Cuculidae	*Coccyzus americanus*	Australasia	0	-0.3294	-	2.57	0.6370	0.3880	82.91
Cuculidae	*Coccyzus euleri*	Afrotropics	0	0.0355	-	3.35	0.7941	0.8188	10.59
Cuculidae	*Coccyzus lansbergi*	Neotropics	0	0.0683	7.5437	3.75	0.7367	0.2284	55.75
Cuculidae	*Coccyzus longirostris*	Australasia	0	-0.2507	-	2.84	0.7363	0.1575	24.20
Cuculidae	*Coccyzus melacoryphus*	Australasia	0	-0.0217	5.5435	2.88	0.7151	0.1855	27.74
Cuculidae	*Coccyzus merlini*	Neotropics	0	-0.0189	5.3647	3.51	0.7406	0.2815	86.16
Cuculidae	*Coccyzus minor*	Neotropics	0	-0.1765	3.3419	3.86	0.8513	0.6471	40.75
Cuculidae	*Coccyzus pluvialis*	Neotropics	0	-0.0477	2.9620	3.87	0.8026	0.7139	31.11
Cuculidae	*Coccyzus rufigularis*	Neotropics	1	0.0686	0.9683	4.23	0.7428	0.4202	31.48
Cuculidae	*Coccyzus vetula*	Neotropics	0	-0.2003	12.5072	3.79	0.7542	0.1952	35.95
Cuculidae	*Coccyzus vieilloti*	Palearctic	0	-0.8148	-	4.37	0.8550	0.1548	0.80
Cuculidae	*Coua caerulea*	Afrotropics	0	0.0036	12.0635	3.46	0.7809	0.3698	83.90
Cuculidae	*Coua coquereli*	Afrotropics	0	0.1070	9.3628	3.34	0.7172	0.1112	21.88
Cuculidae	*Coua cristata*	Neotropics	0	0.0062	-	3.49	0.7505	0.2694	28.02
Cuculidae	*Coua cursor*	Neotropics	0	-0.0149	-	4.03	0.8342	0.6972	0.37
Cuculidae	*Coua gigas*	Neotropics	0	-0.0280	-	3.92	0.8009	0.4897	16.87
Cuculidae	*Coua reynaudii*	Neotropics	1	-0.1202	0.6137	3.04	0.7266	0.2589	65.53
Cuculidae	*Coua ruficeps*	Neotropics	0	0.2396	-	4.04	0.8090	0.6979	21.53
Cuculidae	*Coua serriana*	Neotropics	0	0.1542	-	3.22	0.7323	0.5660	54.93
Cuculidae	*Coua verreauxi*	Neotropics	1	-0.0326	6.1641	3.04	0.6598	0.2337	34.63
Cuculidae	*Crotophaga ani*	Neotropics	1	-0.0308	3.8897	3.32	0.7750	0.2681	44.36
Cuculidae	*Crotophaga major*	Afrotropics	0	-0.1269	-	3.70	0.8351	0.3690	60.94
Cuculidae	*Crotophaga sulcirostris*	Afrotropics	0	-0.0844	8.2768	3.50	0.8507	0.4977	40.26
Cuculidae	*Cuculus canorus*	Neotropics	0	-0.0092	1.7207	4.08	0.7966	0.6955	40.05
Cuculidae	*Cuculus clamosus*	Neotropics	0	0.7588	-	2.96	0.7030	0.1624	68.47
Cuculidae	*Cuculus crassirostris*	Neotropics	0	0.0098	4.1465	3.49	0.7512	0.2871	26.48
Cuculidae	*Cuculus fugax*	Neotropics	0	-0.0034	0.3784	4.10	0.7689	0.9096	6.84
Cuculidae	*Cuculus gularis*	Neotropics	0	0.5674	0.5316	3.68	0.6906	0.4683	45.86
Cuculidae	*Cuculus lepidus*	Neotropics	0	-0.0162	2.0856	4.01	0.8322	0.6897	8.28
Cuculidae	*Cuculus micropterus*	Neotropics	0	-0.1168	4.2012	3.50	0.7738	0.3623	24.82
Cuculidae	*Cuculus optatus*	Neotropics	0	0.4372	-	4.07	0.6997	0.3620	9.34
Cuculidae	*Cuculus pallidus*	Neotropics	0	0.0354	2.7861	3.39	0.7050	0.5578	80.14
Cuculidae	*Cuculus poliocephalus*	Neotropics	1	0.0074	3.6413	2.92	0.6562	0.2111	66.74
Cuculidae	*Cuculus rochii*	Neotropics	0	-0.3210	3.1908	3.35	0.7933	0.2970	61.32
Cuculidae	*Cuculus saturatus*	Neotropics	0	0.2360	8.8465	3.47	0.8012	0.3688	30.39
Cuculidae	*Cuculus solitarius*	Neotropics	0	-0.2404	4.4402	3.83	0.8352	0.7209	16.80
Cuculidae	*Cuculus sparverioides*	Neotropics	0	0.0000	2.7493	4.49	0.6302	0.7439	54.22
Cuculidae	*Cuculus vagans*	Neotropics	0	-0.1028	5.5422	3.90	0.8492	0.4421	30.22
Cuculidae	*Cuculus varius*	Neotropics	0	-0.0501	1.9500	4.10	0.8101	0.3690	7.61
Cuculidae	*Dromococcyx phasianellus*	Neotropics	0	-0.1275	1.7767	3.88	0.8045	0.3681	34.59
Cuculidae	*Eudynamys melanorhynchus*	Afrotropics	0	-0.0541	-	4.00	0.8009	0.4906	54.76
Cuculidae	*Eudynamys orientalis*	Afrotropics	0	-0.1560	11.2217	3.41	0.7978	0.3663	72.25
Cuculidae	*Eudynamys scolopaceus*	Afrotropics	0	-0.1506	-	3.33	0.8235	0.3180	18.19
Cuculidae	*Eudynamys scolopaceus*	Afrotropics	0	-0.1506	-	3.33	0.8235	-	-
Cuculidae	*Eudynamys taitensis*	Afrotropics	0	-33.8802	-	3.57	0.6824	0.4594	41.90

Family	Species	Realm	Threat	Latitude	Elevation	Anomaly	Size	Shape	Orientation
Cuculidae	*Geococcyx californianus*	IndoMalay	0	-0.4376	9.2504	3.22	0.7971	0.4960	16.38
Cuculidae	*Geococcyx velox*	IndoMalay	0	0.0596	-	2.84	0.7685	0.3678	2.98
Cuculidae	*Guira guira*	Neotropics	0	-0.2289	-	3.77	0.8158	0.4393	70.25
Cuculidae	*Microdynamis parva*	Neotropics	0	-0.1640	16.7674	3.31	0.7945	0.1928	84.58
Cuculidae	*Morococcyx erythropygus*	Afrotropics	0	4.1363	-	3.65	0.7656	0.0883	8.81
Cuculidae	*Neomorphus geoffroyi*	Afrotropics	0	0.0084	-	3.30	0.8165	0.3771	15.57
Cuculidae	*Neomorphus pucheranii*	Afrotropics	0	6.7827	-	3.34	0.7683	0.4149	79.58
Cuculidae	*Neomorphus radiolosus*	Afrotropics	0	-0.0626	-	3.46	0.7946	0.1837	70.83
Cuculidae	*Neomorphus rufipennis*	Afrotropics	0	-0.2063	0.0000	3.50	0.7505	0.4003	73.10
Cuculidae	*Neomorphus squamiger*	Afrotropics	0	0.0415	-	3.12	0.7552	0.3388	35.55
Cuculidae	*Pachycoccyx audeberti*	IndoMalay	0	-0.1853	-	3.31	0.8358	0.4786	10.73
Cuculidae	*Phaenicophaeus calyorhynchus*	Neotropics	1	0.4740	4.9720	3.03	0.6740	0.2647	69.07
Cuculidae	*Phaenicophaeus chlorophaeus*	Neotropics	0	-0.0144	-	3.90	0.7818	0.3475	7.33
Cuculidae	*Phaenicophaeus cumingi*	Neotropics	0	0.1417	-	3.46	0.7942	0.3011	25.37
Cuculidae	*Phaenicophaeus curvirostris*	Neotropics	0	-0.2827	-	3.72	0.8302	0.7562	66.15
Cuculidae	*Phaenicophaeus diardi*	IndoMalay	0	-0.0651	2.6647	2.63	0.6961	0.8096	10.51
Cuculidae	*Phaenicophaeus javanicus*	Australasia	0	-0.0633	-	2.71	0.7179	0.5677	60.66
Cuculidae	*Phaenicophaeus leschenaultii*	IndoMalay	0	-0.0604	2.1215	2.66	0.6997	0.4736	75.00
Cuculidae	*Phaenicophaeus pyrrhocephalus*	IndoMalay	1	0.1284	2.3950	2.66	0.5985	0.5771	46.13
Cuculidae	*Phaenicophaeus sumatranus*	IndoMalay	1	0.0121	3.4999	2.63	0.6523	0.5247	87.52
Cuculidae	*Phaenicophaeus superciliosus*	IndoMalay	0	-0.1458	2.0403	2.57	0.6221	0.2612	67.30
Cuculidae	*Phaenicophaeus tristis*	Neotropics	1	0.0040	13.0726	3.63	0.7251	0.1961	23.03
Cuculidae	*Phaenicophaeus viridirostris*	Australasia	0	-0.0546	6.3669	2.76	0.7240	0.1543	24.74
Cuculidae	*Piaya cayana*	Afrotropics	0	0.1250	-	3.43	0.8075	0.5816	16.73
Cuculidae	*Piaya melanogaster*	Neotropics	0	-0.4186	7.0715	3.20	0.7834	0.4041	44.67
Cuculidae	*Rhamphomantis megarhynchus*	IndoMalay	0	-0.0094	4.0650	3.11	0.7185	0.1565	65.59
Cuculidae	*Scythrops novaehollandiae*	Afrotropics	0	-0.3203	6.2796	3.48	0.8705	0.3478	16.09
Cuculidae	*Surniculus lugubris*	Neotropics	0	-0.2623	-	3.54	0.8098	0.3153	42.92
Cuculidae	*Tapera naevia*	Palearctic	0	-0.7726	11.0863	3.92	0.8216	0.1639	2.30
Dasyornithidae	*Dasyornis brachypterus*	Neotropics	1	0.1391	11.8939	3.57	0.7300	0.2810	13.05
Dasyornithidae	*Dasyornis broadbenti*	Australasia	0	-0.1050	-	2.78	0.7706	0.3107	23.58
Dasyornithidae	*Dasyornis longirostris*	Australasia	0	-0.1897	-	3.37	0.8058	0.4961	2.96
Dendrocolaptidae	*Campylorhamphus falcularius*	Neotropics	0	-0.1276	-	3.67	0.7932	0.2576	31.55
Dendrocolaptidae	*Campylorhamphus procurvoides*	Neotropics	0	-0.2100	-	3.75	0.8554	0.6972	46.81
Dendrocolaptidae	*Campylorhamphus pusillus*	Neotropics	0	-0.1519	2.3136	3.33	0.7399	0.4162	10.01
Dendrocolaptidae	*Campylorhamphus trochilirostris*	Neotropics	0	0.6349	6.9273	4.17	0.7128	0.4367	75.55
Dendrocolaptidae	*Certhiasomus stictolaemus*	IndoMalay	0	-0.2673	-	3.61	0.7833	0.7882	3.20
Dendrocolaptidae	*Deconychura longicauda*	Australasia	0	-0.5660	-	3.18	0.8105	0.6587	8.67
Dendrocolaptidae	*Dendrexetastes rufigula*	Neotropics	0	-0.2675	1.4603	3.00	0.6631	0.7553	16.68
Dendrocolaptidae	*Dendrocincla anabatina*	Neotropics	0	-0.0799	10.7477	3.40	0.7539	0.3990	8.94
Dendrocolaptidae	*Dendrocincla fuliginosa*	Neotropics	0	0.2581	3.8313	3.27	0.7466	0.3418	65.01
Dendrocolaptidae	*Dendrocincla homochroa*	Australasia	0	-0.4373	-	3.27	0.8293	0.7472	0.27
Dendrocolaptidae	*Dendrocincla merula*	Australasia	0	-0.3459	9.8528	2.85	0.7240	0.1707	21.14
Dendrocolaptidae	*Dendrocincla tyrannina*	Neotropics	0	0.0242	-	4.05	0.8358	0.8158	2.61
Dendrocolaptidae	*Dendrocolaptes certhia*	Palearctic	0	-0.7300	16.9210	3.99	0.8223	0.3678	11.01
Dendrocolaptidae	*Dendrocolaptes hoffmannsi*	IndoMalay	0	0.0044	14.6444	3.49	0.7725	0.2823	14.18
Dendrocolaptidae	*Dendrocolaptes picumnus*	Palearctic	0	-0.9798	10.8000	4.67	0.8716	0.1989	5.49
Dendrocolaptidae	*Dendrocolaptes platyrostris*	Neotropics	0	-0.2097	6.1270	4.02	0.7371	0.1356	36.12
Dendrocolaptidae	*Dendrocolaptes sanctithomae*	Neotropics	0	-0.2559	4.5916	3.51	0.7096	0.2366	32.87
Dendrocolaptidae	*Dendroplex kienerii*	Nearctic	0	-0.6039	-	4.33	0.8218	0.1681	14.42
Dendrocolaptidae	*Dendroplex picus*	Nearctic	0	-0.7202	3.6197	4.01	0.8011	0.4508	35.93
Dendrocolaptidae	*Drymornis bridgesii*	Neotropics	0	-0.5151	-	2.82	0.7976	0.6480	40.76
Dendrocolaptidae	*Drymotoxeres pucherani*	Neotropics	0	-0.3419	0.1224	2.73	0.6523	0.0687	10.88
Dendrocolaptidae	*Glyphorynchus spirurus*	Afrotropics	1	-0.0800	5.1019	3.18	0.7607	0.3956	69.98
Dendrocolaptidae	*Hylexetastes brigidai*	Afrotropics	1	-0.3556	11.3436	3.43	0.7363	0.1721	79.54
Dendrocolaptidae	*Hylexetastes perrotii*	IndoMalay	0	-0.1055	6.1587	3.34	0.8120	0.4434	5.86
Dendrocolaptidae	*Hylexetastes perrotii*	IndoMalay	0	-0.1055	6.1587	3.34	0.8120	-	-
Dendrocolaptidae	*Hylexetastes stresemanni*	Afrotropics	0	-0.4052	10.1325	3.87	0.8018	0.9165	2.25
Dendrocolaptidae	*Hylexetastes uniformis*	Palearctic	0	-0.4869	11.4368	3.77	0.8657	0.3200	11.19
Dendrocolaptidae	*Lepidocolaptes affinis*	Palearctic	0	-1.0776	11.9746	4.31	0.8433	0.2589	7.88
Dendrocolaptidae	*Lepidocolaptes albolineatus*	Nearctic	0	-0.7385	-	4.17	0.8437	0.6662	6.78
Dendrocolaptidae	*Lepidocolaptes angustirostris*	Afrotropics	0	0.0344	-	3.27	0.7909	0.4242	1.08
Dendrocolaptidae	*Lepidocolaptes falcinellus*	Afrotropics	0	-0.2686	8.5280	3.28	0.7245	0.4981	66.84
Dendrocolaptidae	*Lepidocolaptes lacrymiger*	Palearctic	0	-0.9432	-	4.50	0.8430	0.3126	3.87
Dendrocolaptidae	*Lepidocolaptes leucogaster*	Palearctic	0	-1.1691	8.5575	4.01	0.7604	0.1759	16.70
Dendrocolaptidae	*Lepidocolaptes souleyetii*	IndoMalay	0	-0.2434	-	3.44	0.8492	0.4989	19.15
Dendrocolaptidae	*Lepidocolaptes souleyetii*	IndoMalay	0	-0.2434	-	3.44	0.8492	-	-
Dendrocolaptidae	*Lepidocolaptes squamatus*	Palearctic	0	-0.7296	8.4326	3.78	0.8175	0.2454	2.94
Dendrocolaptidae	*Lepidocolaptes squamatus*	Palearctic	0	-0.7296	8.4326	3.78	0.8175	-	-
Dendrocolaptidae	*Nasica longirostris*	Neotropics	0	-0.2420	-	3.17	0.6997	0.2741	82.80

Family	Species	Realm	Threat	Latitude	Elevation	Anomaly	Size	Shape	Orientation
Dendrocolaptidae	*Sittasomus griseicapillus*	IndoMalay	0	-0.0846	-	3.36	0.7738	0.4775	10.81
Dendrocolaptidae	*Xiphocolaptes albicollis*	Australasia	0	-0.1384	3.9073	2.71	0.7179	0.5352	59.02
Dendrocolaptidae	*Xiphocolaptes falcirostris*	Australasia	0	0.0672	-	2.48	0.6523	0.6310	32.38
Dendrocolaptidae	*Xiphocolaptes major*	Afrotropics	1	-0.1424	8.2105	3.04	0.7211	0.1454	75.21
Dendrocolaptidae	*Xiphocolaptes promeropirhynchus*	Australasia	0	-0.3721	9.4223	2.89	0.7862	0.2524	68.69
Dendrocolaptidae	*Xiphorhynchus elegans*	Neotropics	0	-0.4362	7.4476	2.57	0.7179	0.4578	88.46
Dendrocolaptidae	*Xiphorhynchus erythropygius*	Neotropics	0	15.4108	14.6801	3.74	0.7441	0.3103	78.60
Dendrocolaptidae	*Xiphorhynchus flavigaster*	Palearctic	0	-0.4548	11.4178	3.93	0.8861	0.4841	19.12
Dendrocolaptidae	*Xiphorhynchus fuscus*	Afrotropics	0	-0.0788	5.0645	3.18	0.7614	0.3874	70.15
Dendrocolaptidae	*Xiphorhynchus guttatus*	Afrotropics	0	-0.2041	-	3.71	0.8199	0.7585	1.10
Dendrocolaptidae	*Xiphorhynchus lachrymosus*	Afrotropics	0	0.0220	-	3.49	0.8416	0.4010	15.27
Dendrocolaptidae	*Xiphorhynchus obsoletus*	Afrotropics	0	0.1177	-	3.25	0.7783	0.5205	56.27
Dendrocolaptidae	*Xiphorhynchus ocellatus*	Afrotropics	0	-0.2097	-	4.11	0.8080	0.6080	22.71
Dendrocolaptidae	*Xiphorhynchus pardalotus*	Afrotropics	0	0.0581	-	3.32	0.7975	0.4981	52.65
Dendrocolaptidae	*Xiphorhynchus spixii*	Palearctic	0	-0.9778	-	4.47	0.8315	0.6092	5.76
Dendrocolaptidae	*Xiphorhynchus susurrans*	Afrotropics	1	-0.3633	0.4936	3.26	0.6370	0.4264	82.46
Dendrocolaptidae	*Xiphorhynchus triangularis*	Neotropics	0	-0.0374	6.9189	3.12	0.7120	0.2313	72.86
Dicaeidae	*Dicaeum aeneum*	Neotropics	0	-0.2597	3.7095	2.47	0.5985	0.0000	0.00
Dicaeidae	*Dicaeum aeruginosum*	Nearctic	0	-0.7788	6.4174	4.07	0.8077	0.9286	27.21
Dicaeidae	*Dicaeum agile*	Neotropics	0	-0.2167	-	2.47	0.6302	0.0554	12.84
Dicaeidae	*Dicaeum annae*	Nearctic	0	-0.5535	-	4.39	0.8358	0.1327	8.93
Dicaeidae	*Dicaeum anthonyi*	Nearctic	0	-0.5299	-	4.39	0.8123	0.1590	14.21
Dicaeidae	*Dicaeum aureolimbatum*	Nearctic	0	-0.8010	-	3.44	0.7958	0.2361	30.63
Dicaeidae	*Dicaeum australe*	Nearctic	0	-0.6645	-	4.31	0.8194	0.2977	11.89
Dicaeidae	*Dicaeum bicolor*	Palearctic	0	-0.8467	-	3.69	0.8212	0.3842	37.30
Dicaeidae	*Dicaeum celebicum*	Afrotropics	0	0.0880	8.3212	3.55	0.7588	0.5959	83.25
Dicaeidae	*Dicaeum chrysorrheum*	Afrotropics	0	0.0046	7.2636	3.74	0.7978	0.1698	3.82
Dicaeidae	*Dicaeum concolor*	Afrotropics	0	-0.1447	-	3.57	0.8525	0.7760	35.26
Dicaeidae	*Dicaeum cruentatum*	Afrotropics	0	0.0820	5.8292	3.24	0.8083	0.2190	16.67
Dicaeidae	*Dicaeum erythrorhynchos*	Afrotropics	0	3.6578	7.6517	3.33	0.7785	0.2397	14.58
Dicaeidae	*Dicaeum erythrothorax*	Afrotropics	0	-0.2578	6.9071	3.47	0.7667	0.4690	84.21
Dicaeidae	*Dicaeum everetti*	Neotropics	0	-0.0107	0.2203	4.19	0.7611	0.5414	8.32
Dicaeidae	*Dicaeum eximium*	Neotropics	0	-0.0131	4.1073	3.96	0.8457	0.5954	21.01
Dicaeidae	*Dicaeum geelvinkianum*	Neotropics	1	-0.2247	7.1866	3.73	0.6523	0.2718	40.24
Dicaeidae	*Dicaeum haematostictum*	Neotropics	0	0.0643	11.6210	3.52	0.7266	0.4080	23.22
Dicaeidae	*Dicaeum hirundinaceum*	Neotropics	0	-0.0634	10.4195	3.67	0.7261	0.2337	19.04
Dicaeidae	*Dicaeum hypoleucum*	Neotropics	0	-0.0283	-	4.05	0.8231	0.6792	31.23
Dicaeidae	*Dicaeum igniferum*	Australasia	0	-0.2362	-	2.48	0.6120	0.0400	36.10
Dicaeidae	*Dicaeum ignipectus*	IndoMalay	0	-0.0017	6.9294	2.64	0.7300	0.3116	68.02
Dicaeidae	*Dicaeum maugei*	IndoMalay	0	-0.0621	-	3.19	0.8239	0.4571	30.23
Dicaeidae	*Dicaeum melanoxanthum*	Australasia	0	-0.3165	-	2.63	0.6478	0.0000	0.00
Dicaeidae	*Dicaeum monticolum*	IndoMalay	0	-0.0209	6.5923	2.65	0.7198	0.1625	66.92
Dicaeidae	*Dicaeum nehrkorni*	Australasia	0	-0.0870	-	2.71	0.7185	0.5623	60.71
Dicaeidae	*Dicaeum nigrilore*	IndoMalay	0	-0.0251	2.5750	2.64	0.7281	0.2129	67.63
Dicaeidae	*Dicaeum nigrilore*	IndoMalay	0	-0.0251	2.5750	2.64	0.7281	-	-
Dicaeidae	*Dicaeum pectorale*	IndoMalay	0	-0.0031	-	2.64	0.7318	0.2312	67.52
Dicaeidae	*Dicaeum proprium*	Australasia	0	-0.0350	-	2.71	0.7185	0.5623	60.71
Dicaeidae	*Dicaeum pygmaeum*	IndoMalay	0	-0.0646	-	3.02	0.8167	0.3824	59.08
Dicaeidae	*Dicaeum quadricolor*	IndoMalay	0	-0.1549	-	3.19	0.8281	-	-
Dicaeidae	*Dicaeum quadricolor*	IndoMalay	0	-0.1549	-	3.19	0.8281	0.8614	82.26
Dicaeidae	*Dicaeum retrocinctum*	IndoMalay	0	-0.1351	-	3.07	0.8247	0.6850	68.68
Dicaeidae	*Dicaeum sanguinolentum*	IndoMalay	0	-0.1177	-	3.30	0.8044	0.6609	38.02
Dicaeidae	*Dicaeum trigonostigma*	Australasia	0	-0.1177	-	2.48	0.6428	0.1580	73.00
Dicaeidae	*Dicaeum tristrami*	IndoMalay	0	0.1184	2.0186	2.86	0.7666	0.4440	3.42
Dicaeidae	*Dicaeum trochileum*	Australasia	0	-0.3323	-	2.50	0.6716	0.2680	24.42
Dicaeidae	*Dicaeum vincens*	Australasia	0	-0.0804	-	2.80	0.7654	0.4023	24.64
Dicaeidae	*Dicaeum vulneratum*	IndoMalay	1	-0.0004	3.1276	2.63	0.6478	0.3503	71.22
Dicaeidae	*Prionochilus maculatus*	IndoMalay	1	0.1240	3.0471	2.88	0.7488	0.5473	59.38
Dicaeidae	*Prionochilus olivaceus*	Palearctic	1	-1.0504	-	6.43	0.7963	0.0250	1.27
Dicaeidae	*Prionochilus percussus*	Neotropics	0	-0.2634	3.6051	3.49	0.8170	0.3746	82.39
Dicaeidae	*Prionochilus plateni*	Neotropics	0	-0.3766	6.2159	3.49	0.7531	0.2408	68.84
Dicaeidae	*Prionochilus thoracicus*	Australasia	0	-0.8545	-	3.56	0.7881	0.5825	4.53
Dicaeidae	*Prionochilus xanthopygius*	Australasia	0	-0.7585	-	2.97	0.7651	0.1629	12.74
Dicruridae	*Chaetorhynchus papuensis*	IndoMalay	0	-0.0875	-	2.65	0.6716	0.6458	83.32
Dicruridae	*Dicrurus adsimilis*	IndoMalay	0	-0.0187	-	2.64	0.7261	0.2111	67.49
Dicruridae	*Dicrurus aeneus*	Australasia	0	0.9850	-	2.63	0.6478	0.0000	0.00
Dicruridae	*Dicrurus annectans*	IndoMalay	0	-0.2068	-	3.26	0.8273	0.8542	18.03
Dicruridae	*Dicrurus atripennis*	Australasia	0	0.3530	-	2.58	0.6631	0.1360	2.68
Dicruridae	*Dicrurus balicassius*	IndoMalay	0	-0.0694	-	3.53	0.7928	0.7020	5.97
Dicruridae	*Dicrurus bracteatus*	IndoMalay	0	0.0929	-	2.88	0.7104	0.5070	71.68
Dicruridae	*Dicrurus caerulescens*	Australasia	0	-0.0413	-	2.72	0.7151	0.5029	56.34
Dicruridae	*Dicrurus densus*	IndoMalay	0	-0.0867	-	2.63	0.6842	0.7223	72.42
Dicruridae	*Dicrurus forficatus*	Australasia	0	-0.4822	-	2.57	0.6986	0.4095	43.94

Family	Species	Realm	Threat	Latitude	Elevation	Anomaly	Size	Shape	Orientation
Dicruridae	*Dicrurus hottentottus*	IndoMalay	0	-0.1158	-	2.63	0.6824	0.7072	73.13
Dicruridae	*Dicrurus leucophaeus*	IndoMalay	0	-0.0288	-	2.63	0.7327	0.3292	67.66
Dicruridae	*Dicrurus macrocercus*	IndoMalay	1	-0.0593	2.8820	2.66	0.5985	0.5771	46.13
Dicruridae	*Dicrurus megarhynchus*	IndoMalay	0	-0.1653	7.0371	2.67	0.7165	0.0743	7.52
Dicruridae	*Dicrurus montanus*	IndoMalay	0	-0.0943	-	2.89	0.8052	0.5964	44.93
Dicruridae	*Dicrurus remifer*	IndoMalay	0	-0.0544	0.9959	2.79	0.7464	0.5347	22.21
Dicruridae	*Dicrurus sumatranus*	IndoMalay	0	0.4252	6.1657	2.76	0.6302	0.6063	90.00
Donacobiidae	*Donacobius atricapilla*	IndoMalay	0	0.0121	2.1014	2.85	0.7835	0.5629	19.92
Dromadidae	*Dromas ardeola*	Neotropics	0	-0.1105	2.5146	3.90	0.8024	0.3586	27.71
Dromaiidae	*Dromaius novaehollandiae*	Neotropics	0	0.1702	1.7790	2.99	0.7318	0.4707	84.68
Emberizidae	*Acanthidops bairdi*	Neotropics	0	-0.0964	8.5128	3.08	0.6598	0.3844	32.22
Emberizidae	*Aimophila notosticta*	Australasia	0	-0.1268	-	2.76	0.7446	0.5118	51.16
Emberizidae	*Aimophila rufescens*	Neotropics	0	0.3753	1.8772	3.59	0.6842	0.5929	34.57
Emberizidae	*Aimophila ruficeps*	Neotropics	0	-0.0140	10.6164	3.74	0.7774	0.1991	31.00
Emberizidae	*Ammodramus aurifrons*	Neotropics	0	-0.5069	-	3.09	0.7911	0.4046	69.36
Emberizidae	*Ammodramus bairdii*	Neotropics	0	0.0661	-	3.98	0.8219	0.6911	1.20
Emberizidae	*Ammodramus caudacutus*	Nearctic	0	2.4322	4.6068	4.34	0.7771	0.4270	13.31
Emberizidae	*Ammodramus henslowii*	Nearctic	1	-0.9788	0.0000	3.74	0.7069	0.2187	43.54
Emberizidae	*Ammodramus humeralis*	Nearctic	0	-0.9614	-	4.46	0.7899	0.3723	6.50
Emberizidae	*Ammodramus leconteii*	Neotropics	0	-0.2678	-	3.53	0.8432	0.7552	86.71
Emberizidae	*Ammodramus maritimus*	Nearctic	0	-0.6292	-	4.42	0.8152	0.2519	15.46
Emberizidae	*Ammodramus nelsoni*	Nearctic	0	-0.6580	0.0000	4.41	0.7955	0.2219	10.81
Emberizidae	*Ammodramus savannarum*	Nearctic	0	-0.4591	-	4.22	0.8310	0.5504	15.52
Emberizidae	*Amphispiza belli*	Nearctic	0	-0.4257	-	4.18	0.7883	0.8344	3.81
Emberizidae	*Amphispiza bilineata*	Nearctic	0	-0.6566	-	4.10	0.8100	0.3937	42.71
Emberizidae	*Amphispiza quinquestriata*	Nearctic	0	-0.4571	7.2256	4.06	0.7310	0.1726	52.75
Emberizidae	*Arremon abeillei*	Nearctic	0	-0.9059	-	6.08	0.8194	0.0513	0.08
Emberizidae	*Arremon aurantiirostris*	Nearctic	0	-0.8377	0.9641	4.85	0.7205	0.4769	61.33
Emberizidae	*Arremon brunneinucha*	IndoMalay	0	-0.0087	2.7947	2.84	0.7882	0.4688	5.03
Emberizidae	*Arremon crassirostris*	Neotropics	0	-0.2041	2.0138	3.22	0.7078	0.2981	78.15
Emberizidae	*Arremon flavirostris*	Neotropics	0	0.0204	3.4149	3.38	0.7787	0.3296	56.38
Emberizidae	*Arremon franciscanus*	Neotropics	0	0.0097	13.4466	3.57	0.7862	0.4181	49.97
Emberizidae	*Arremon schlegeli*	Neotropics	0	0.1684	7.7779	3.33	0.7251	0.3066	77.29
Emberizidae	*Arremon semitorquatus*	Neotropics	0	-0.0108	7.1905	3.01	0.6740	0.1821	17.10
Emberizidae	*Arremon taciturnus*	Neotropics	0	-0.4165	5.4501	3.70	0.8053	0.6215	9.69
Emberizidae	*Arremon torquatus*	Neotropics	0	4.1992	-	3.65	0.6974	0.6076	52.21
Emberizidae	*Arremon virenticeps*	Neotropics	0	-0.0909	3.0755	3.61	0.7323	0.6133	15.22
Emberizidae	*Arremonops chloronotus*	Neotropics	0	-0.5785	4.0205	2.89	0.7234	0.2110	29.80
Emberizidae	*Arremonops conirostris*	Neotropics	0	-0.0651	3.4446	3.99	0.8370	0.6562	24.68
Emberizidae	*Arremonops rufivirgatus*	Neotropics	0	-0.1907	12.8574	3.63	0.7765	0.4181	73.92
Emberizidae	*Arremonops tocuyensis*	Neotropics	0	-0.5579	7.2670	3.82	0.7165	0.3180	38.16
Emberizidae	*Atlapetes albiceps*	Afrotropics	0	-0.0817	7.9247	3.19	0.7515	0.3279	69.49
Emberizidae	*Atlapetes albinucha*	IndoMalay	0	-0.0811	-	3.44	0.8288	0.3481	15.85
Emberizidae	*Atlapetes albofrenatus*	Neotropics	0	-0.4293	12.1441	3.71	0.8562	0.2593	52.92
Emberizidae	*Atlapetes blancae*	Palearctic	0	-0.4295	8.0811	4.16	0.8731	0.2766	1.59
Emberizidae	*Atlapetes canigenis*	Neotropics	0	-0.4743	-	3.22	0.6763	0.3444	71.06
Emberizidae	*Atlapetes citrinellus*	Neotropics	0	-0.1526	9.3810	3.41	0.7600	0.1283	35.86
Emberizidae	*Atlapetes flaviceps*	Neotropics	0	-0.3125	5.8495	3.88	0.6997	0.2726	52.63
Emberizidae	*Atlapetes fulviceps*	Neotropics	0	-68.3817	3.1412	4.15	0.6859	0.6570	39.29
Emberizidae	*Atlapetes fuscoolivaceus*	Neotropics	0	1.1198	8.8435	3.57	0.6631	0.2396	75.84
Emberizidae	*Atlapetes latinuchus*	Neotropics	1	5.4678	6.8420	3.14	0.6523	0.2540	71.57
Emberizidae	*Atlapetes leucopis*	Neotropics	0	-44.1609	6.1223	4.16	0.6598	0.9357	16.33
Emberizidae	*Atlapetes leucopterus*	Neotropics	0	7.3739	8.7578	3.91	0.7096	0.2426	83.66
Emberizidae	*Atlapetes melanocephalus*	Neotropics	0	0.0000	4.7708	3.30	0.6302	0.6124	90.00
Emberizidae	*Atlapetes melanolaemus*	Neotropics	0	0.0069	8.3985	3.37	0.7444	0.2222	71.76
Emberizidae	*Atlapetes melanopsis*	Neotropics	0	-8.6112	5.9296	3.25	0.6784	0.1587	66.06
Emberizidae	*Atlapetes nationi*	Neotropics	0	-0.0646	8.0732	3.20	0.7069	0.2191	75.48
Emberizidae	*Atlapetes pallidiceps*	Neotropics	0	0.1227	9.4590	3.25	0.6302	0.6246	0.00
Emberizidae	*Atlapetes pallidinucha*	Neotropics	0	-23.1057	8.6767	4.11	0.6961	0.3010	29.95
Emberizidae	*Atlapetes personatus*	Neotropics	1	0.0000	5.6432	4.05	0.5788	0.0000	90.00
Emberizidae	*Atlapetes pileatus*	Neotropics	0	-0.0793	11.1773	3.93	0.6997	0.1527	46.89
Emberizidae	*Atlapetes rufigenis*	Neotropics	1	0.0000	1.8606	3.29	0.5985	0.2277	61.85
Emberizidae	*Atlapetes rufinucha*	Neotropics	0	5.2061	4.8734	3.39	0.7272	0.1708	58.47
Emberizidae	*Atlapetes schistaceus*	Neotropics	0	4.5313	8.2708	4.38	0.7165	0.3902	1.77
Emberizidae	*Atlapetes seebohmi*	Neotropics	0	-0.1409	12.1115	3.81	0.7591	0.3434	41.33
Emberizidae	*Atlapetes semirufus*	Neotropics	0	0.4206	7.4227	3.56	0.6690	0.4284	56.91
Emberizidae	*Atlapetes terborghi*	Neotropics	0	3.2020	8.7841	4.00	0.6961	0.2317	33.75
Emberizidae	*Atlapetes tricolor*	Neotropics	0	0.2081	6.0542	3.55	0.7477	0.2813	77.88
Emberizidae	*Calamospiza melanocorys*	Australasia	0	-0.4015	8.2798	3.02	0.8103	0.8554	27.96
Emberizidae	*Calcarius lapponicus*	IndoMalay	1	-0.0561	3.0324	3.04	0.7857	0.2810	74.17
Emberizidae	*Calcarius ornatus*	Australasia	0	-0.8143	-	3.11	0.8026	0.3522	9.19
Emberizidae	*Calcarius pictus*	Australasia	0	-0.5780	-	2.53	0.7305	0.7928	0.58
Emberizidae	*Camarhynchus pallidus*	Nearctic	0	-0.8577	6.2273	3.54	0.7648	0.3277	47.17
Emberizidae	*Camarhynchus parvulus*	Nearctic	0	-0.1046	-	3.86	0.7669	0.4625	54.19

Family	Species	Realm	Threat	Latitude	Elevation	Anomaly	Size	Shape	Orientation
Emberizidae	*Camarhynchus psittacula*	Afrotropics	0	0.1185	-	3.17	0.7920	0.2121	11.15
Emberizidae	*Camarhynchus psittacula*	Afrotropics	0	0.1185	-	3.17	0.7920	-	-
Emberizidae	*Catamenia analis*	Neotropics	0	-0.0398	-	2.96	0.7112	0.2195	73.00
Emberizidae	*Catamenia homochroa*	Neotropics	0	0.1582	-	3.29	0.7144	0.4102	69.15
Emberizidae	*Catamenia inornata*	Neotropics	0	-0.5200	6.1952	2.66	0.7446	0.1856	43.87
Emberizidae	*Certhidea olivacea*	Afrotropics	0	-0.0075	-	3.48	0.7732	0.5412	14.10
Emberizidae	*Charitospiza eucosma*	Palearctic	0	-0.9532	11.9115	4.25	0.8339	0.1855	6.37
Emberizidae	*Chondestes grammacus*	Neotropics	0	-0.1533	10.2913	3.66	0.7389	0.2347	39.81
Emberizidae	*Coryphaspiza melanotis*	Australasia	0	-0.7074	-	2.98	0.7894	0.4863	1.11
Emberizidae	*Coryphospingus cucullatus*	Australasia	0	-0.1108	-	2.58	0.6302	0.1134	34.50
Emberizidae	*Coryphospingus pileatus*	Neotropics	0	-0.2309	-	2.54	0.6740	0.1459	14.25
Emberizidae	*Diuca diuca*	Neotropics	0	0.1880	6.0343	3.46	0.7300	0.1442	56.41
Emberizidae	*Diuca speculifera*	Neotropics	0	-2.9075	8.4924	4.39	0.6784	0.6193	2.61
Emberizidae	*Dolospingus fringilloides*	IndoMalay	0	-0.0846	-	2.98	0.8124	0.6281	53.68
Emberizidae	*Donacospiza albifrons*	IndoMalay	0	-0.1670	-	3.26	0.7648	0.7350	15.81
Emberizidae	*Emberiza affinis*	Neotropics	0	-0.0045	-	4.11	0.7660	0.3701	5.69
Emberizidae	*Emberiza aureola*	Neotropics	0	0.2004	-	3.99	0.7727	0.2776	11.34
Emberizidae	*Emberiza bruniceps*	Neotropics	0	-0.3141	3.6594	3.72	0.8133	0.3850	39.34
Emberizidae	*Emberiza buchanani*	Neotropics	0	-0.4996	7.5700	3.70	0.7409	0.1607	84.70
Emberizidae	*Emberiza cabanisi*	Neotropics	0	-0.2907	7.2495	3.82	0.8538	0.4929	41.02
Emberizidae	*Emberiza caesia*	Australasia	0	-0.5521	-	3.34	0.8379	0.5815	7.59
Emberizidae	*Emberiza capensis*	Afrotropics	0	-0.2354	10.5548	3.47	0.8717	0.4146	7.09
Emberizidae	*Emberiza chrysophrys*	Neotropics	0	-0.3430	-	3.44	0.8462	0.3678	47.35
Emberizidae	*Emberiza cia*	Australasia	0	-0.4289	3.5440	3.57	0.7747	0.4303	49.64
Emberizidae	*Emberiza cineracea*	Neotropics	1	-0.0924	4.5056	3.45	0.7136	0.3466	30.12
Emberizidae	*Emberiza cioides*	Neotropics	0	-0.0136	-	3.96	0.8151	0.5190	34.17
Emberizidae	*Emberiza cirlus*	Neotropics	0	-0.3400	4.4072	2.95	0.7639	0.2760	41.60
Emberizidae	*Emberiza citrinella*	Neotropics	1	-0.0209	0.9709	3.18	0.6562	0.2969	87.07
Emberizidae	*Emberiza elegans*	Neotropics	0	-0.6561	-	2.70	0.7803	0.5771	17.24
Emberizidae	*Emberiza flaviventris*	Neotropics	1	-0.2960	3.9202	3.86	0.7933	0.3522	20.17
Emberizidae	*Emberiza fucata*	Afrotropics	0	-0.0055	7.5471	3.66	0.8040	0.5759	14.19
Emberizidae	*Emberiza godlewskii*	Afrotropics	0	0.0665	8.7522	3.35	0.7416	0.1813	21.10
Emberizidae	*Emberiza hortulana*	Afrotropics	0	-0.0680	11.5216	3.43	0.7857	0.4579	87.23
Emberizidae	*Emberiza impetuani*	Afrotropics	0	0.0457	-	3.32	0.8236	0.3293	14.69
Emberizidae	*Emberiza jankowskii*	Afrotropics	0	0.1121	9.0443	3.25	0.8091	0.2084	10.32
Emberizidae	*Emberiza koslowi*	Australasia	0	-0.5441	0.0000	3.33	0.8371	0.4967	14.25
Emberizidae	*Emberiza leucocephalos*	Neotropics	0	-0.2026	6.7179	2.96	0.6562	0.2083	20.02
Emberizidae	*Emberiza melanocephala*	Neotropics	0	-0.3749	4.4373	3.18	0.6478	0.5152	27.35
Emberizidae	*Emberiza pallasi*	Afrotropics	0	0.0886	-	3.46	0.8011	-	-
Emberizidae	*Emberiza pallasi*	Afrotropics	0	0.0886	-	3.46	0.8011	0.1227	7.98
Emberizidae	*Emberiza poliopleura*	Palearctic	1	-0.7114	-	5.09	0.8585	0.1758	0.28
Emberizidae	*Emberiza pusilla*	Palearctic	0	-0.9363	-	4.41	0.8062	0.4759	6.60
Emberizidae	*Emberiza rustica*	Palearctic	0	-1.1129	0.0000	4.26	0.8036	0.2021	15.45
Emberizidae	*Emberiza rutila*	Afrotropics	0	-0.0470	-	3.50	0.8216	0.3786	34.84
Emberizidae	*Emberiza schoeniclus*	Palearctic	0	-0.6986	4.5041	3.79	0.7458	0.3146	23.29
Emberizidae	*Emberiza socotrana*	Afrotropics	0	-0.3004	-	3.74	0.7988	0.7947	37.19
Emberizidae	*Emberiza spodocephala*	Palearctic	0	-0.7668	-	5.03	0.8085	0.4782	11.16
Emberizidae	*Emberiza stewarti*	Palearctic	0	-0.8290	-	3.97	0.8272	0.1666	0.51
Emberizidae	*Emberiza striolata*	Palearctic	0	-1.1139	8.8134	4.16	0.7158	0.1679	8.41
Emberizidae	*Emberiza sulphurata*	Palearctic	0	-0.8506	-	4.09	0.8476	0.4832	1.92
Emberizidae	*Emberiza tahapisi*	Palearctic	0	-0.6626	-	3.62	0.8081	0.3362	3.18
Emberizidae	*Emberiza tristrami*	Palearctic	0	-0.4277	-	4.76	0.8541	0.1973	3.63
Emberizidae	*Emberiza variabilis*	Palearctic	0	-0.9666	-	3.87	0.8138	0.2610	39.18
Emberizidae	*Emberiza yessoensis*	Afrotropics	0	-0.1266	-	3.74	0.8405	0.6381	67.43
Emberizidae	*Emberizoides duidae*	Palearctic	0	-0.9803	-	4.09	0.8226	0.4138	27.32
Emberizidae	*Emberizoides herbicola*	Palearctic	0	-0.8389	-	4.17	0.8339	0.6387	14.79
Emberizidae	*Emberizoides ypiranganus*	Palearctic	0	-0.6070	5.5762	4.57	0.8444	0.3093	4.36
Emberizidae	*Embernagra longicauda*	Afrotropics	0	-0.2704	-	3.94	0.8011	0.7666	45.27
Emberizidae	*Embernagra platensis*	Palearctic	1	-0.6587	-	4.28	0.7385	0.3230	1.97
Emberizidae	*Euneornis campestris*	Neotropics	0	-0.1430	-	3.60	0.7815	0.4319	38.70
Emberizidae	*Geospiza difficilis*	IndoMalay	1	-0.4621	4.9215	2.84	0.6120	0.9804	90.00
Emberizidae	*Geospiza fortis*	Palearctic	0	-0.5757	-	4.27	0.8676	0.2578	4.87
Emberizidae	*Geospiza fuliginosa*	IndoMalay	0	-0.1416	8.4371	4.28	0.7546	0.2188	24.08
Emberizidae	*Geospiza fuliginosa*	IndoMalay	0	-0.1416	8.4371	4.28	0.7546	-	-
Emberizidae	*Geospiza magnirostris*	Palearctic	0	-0.4569	0.9782	6.24	0.8249	-	-
Emberizidae	*Geospiza magnirostris*	Palearctic	0	-0.4569	0.9782	6.24	0.8249	0.0238	0.53
Emberizidae	*Geospiza scandens*	Palearctic	0	-0.7569	1.3966	5.31	0.8626	0.1523	0.77
Emberizidae	*Geospiza scandens*	Palearctic	0	-0.7569	1.3966	5.31	0.8626	-	-
Emberizidae	*Gubernatrix cristata*	Neotropics	0	0.0308	8.0227	3.62	0.7580	0.3913	77.31
Emberizidae	*Haplospiza rustica*	Afrotropics	0	0.0278	6.7795	3.27	0.8158	0.3230	11.53
Emberizidae	*Haplospiza unicolor*	Afrotropics	0	-0.0503	9.4404	3.59	0.8543	0.6359	36.84
Emberizidae	*Idiopsar brachyurus*	Neotropics	0	-0.0755	3.9656	3.99	0.8422	0.5846	31.01
Emberizidae	*Incaspiza laeta*	Nearctic	0	-0.5195	-	3.69	0.7893	0.2109	29.27
Emberizidae	*Incaspiza ortizi*	Neotropics	0	-0.3547	3.1605	2.46	0.6876	0.3855	3.42

Family	Species	Realm	Threat	Latitude	Elevation	Anomaly	Size	Shape	Orientation
Emberizidae	*Incaspiza personata*	Nearctic	0	-0.7563	2.0484	4.24	0.8431	0.5242	1.10
Emberizidae	*Incaspiza pulchra*	Neotropics	0	-0.3045	-	3.18	0.7060	0.2549	81.79
Emberizidae	*Incaspiza watkinsi*	Neotropics	0	-0.3827	-	3.60	0.7503	0.8088	79.01
Emberizidae	*Junco hyemalis*	IndoMalay	0	-0.1524	-	3.08	0.7913	0.7594	62.11
Emberizidae	*Junco phaeonotus*	IndoMalay	0	-0.1780	-	3.16	0.7517	0.3851	57.38
Emberizidae	*Junco vulcani*	Palearctic	0	-0.8383	11.8960	4.11	0.7983	0.2136	2.18
Emberizidae	*Latoucheornis siemsseni*	IndoMalay	0	-0.0837	-	3.16	0.7965	0.6621	10.56
Emberizidae	*Lophospingus griseocristatus*	Palearctic	0	-0.8548	-	4.90	0.8490	0.2051	1.14
Emberizidae	*Lophospingus pusillus*	Palearctic	0	-0.6519	3.2709	4.19	0.8334	0.2379	0.67
Emberizidae	*Loxigilla portoricensis*	Palearctic	1	1.3948	7.1045	4.06	0.7323	0.6937	26.16
Emberizidae	*Loxigilla violacea*	Palearctic	1	-24.9411	6.9657	3.93	0.7040	0.4670	30.41
Emberizidae	*Loxipasser anoxanthus*	Neotropics	0	0.0778	4.4673	3.05	0.6523	0.2420	35.50
Emberizidae	*Melanodera melanodera*	Neotropics	0	-0.5415	4.9893	3.41	0.7752	0.3606	12.06
Emberizidae	*Melanodera xanthogramma*	Neotropics	0	-0.0298	4.2669	3.69	0.6921	0.3227	27.10
Emberizidae	*Melophus lathami*	Australasia	0	-0.0683	-	2.85	0.7392	0.1919	23.26
Emberizidae	*Melopyrrha nigra*	IndoMalay	0	-0.1164	-	3.16	0.7994	0.7559	53.36
Emberizidae	*Melospiza georgiana*	Palearctic	0	-0.9946	-	4.26	0.8170	0.2916	12.24
Emberizidae	*Melospiza lincolnii*	Palearctic	0	-0.8163	-	4.04	0.8342	0.2170	2.47
Emberizidae	*Melospiza melodia*	Palearctic	0	-0.7360	-	5.03	0.8143	0.2953	2.34
Emberizidae	*Melozone aberti*	Palearctic	0	5.1160	8.3618	4.25	0.7851	0.4384	11.43
Emberizidae	*Melozone albicollis*	Palearctic	0	4.9461	-	4.22	0.8178	0.5785	16.99
Emberizidae	*Melozone biarcuata*	Palearctic	0	-0.6841	-	5.10	0.8045	0.2345	0.23
Emberizidae	*Melozone crissalis*	Neotropics	0	-0.7694	-	2.00	0.7205	0.4209	83.11
Emberizidae	*Melozone fuscus*	Neotropics	0	-0.6176	10.9616	2.09	0.7548	0.2537	88.37
Emberizidae	*Melozone kieneri*	Australasia	0	-0.5575	-	3.36	0.8342	0.5566	9.53
Emberizidae	*Melozone leucotis*	Australasia	0	-0.2153	-	2.17	0.6804	0.6686	14.77
Emberizidae	*Miliaria calandra*	IndoMalay	0	-0.1778	8.5185	3.60	0.8508	0.1501	4.33
Emberizidae	*Oriturus superciliosus*	Afrotropics	0	-0.0808	-	3.78	0.8331	0.6247	57.40
Emberizidae	*Oryzoborus angolensis*	Australasia	0	-0.4198	4.8556	2.84	0.6859	0.2157	11.26
Emberizidae	*Oryzoborus atrirostris*	Nearctic	0	-0.5487	11.1426	3.43	0.7631	0.3901	70.09
Emberizidae	*Oryzoborus crassirostris*	Nearctic	0	-0.9029	-	4.24	0.8027	0.7446	2.57
Emberizidae	*Oryzoborus funereus*	Australasia	0	-0.1784	6.2619	3.18	0.7112	0.4136	64.88
Emberizidae	*Oryzoborus maximiliani*	Australasia	0	-0.2966	5.9386	2.84	0.7192	0.1618	22.71
Emberizidae	*Oryzoborus maximiliani*	Australasia	0	-0.2966	5.9386	2.84	0.7192	-	-
Emberizidae	*Oryzoborus nuttingi*	Neotropics	1	-0.0737	7.2490	3.08	0.7104	0.1193	69.68
Emberizidae	*Passerculus sandwichensis*	Afrotropics	0	0.1675	-	3.20	0.7759	0.7720	20.76
Emberizidae	*Passerella iliaca*	Neotropics	0	-13.1694	2.1066	4.29	0.7096	0.2855	68.74
Emberizidae	*Passerina amoena*	Neotropics	0	-0.5889	1.2333	3.21	0.7891	0.4463	72.00
Emberizidae	*Passerina caerulea*	Neotropics	0	-0.4671	1.4268	3.05	0.8072	0.4922	84.79
Emberizidae	*Passerina ciris*	Neotropics	0	-0.0499	4.8129	3.56	0.7860	0.6584	74.69
Emberizidae	*Passerina cyanea*	Neotropics	0	-0.0537	0.9833	4.05	0.8282	0.8669	14.55
Emberizidae	*Passerina leclancherii*	Neotropics	0	-0.2923	0.6738	4.15	0.7528	0.3404	6.81
Emberizidae	*Passerina rositae*	Afrotropics	0	0.8229	7.6036	3.57	0.7261	0.8214	72.72
Emberizidae	*Passerina versicolor*	Australasia	0	-0.3149	-	2.90	0.7050	0.1530	16.63
Emberizidae	*Peucaea aestivalis*	Palearctic	0	-0.4207	3.3548	3.78	0.7849	0.6130	48.83
Emberizidae	*Peucaea botterii*	IndoMalay	0	-0.1312	-	3.21	0.7963	0.5118	6.35
Emberizidae	*Peucaea carpalis*	Afrotropics	0	-0.1277	-	3.62	0.8560	0.8090	54.08
Emberizidae	*Peucaea cassinii*	IndoMalay	0	-0.1213	-	3.23	0.7677	0.3632	34.54
Emberizidae	*Peucaea humeralis*	IndoMalay	0	-0.0567	-	2.83	0.7916	0.5891	9.93
Emberizidae	*Peucaea mystacalis*	IndoMalay	0	0.2052	3.2940	2.76	0.6740	0.7282	83.10
Emberizidae	*Peucaea ruficauda*	IndoMalay	1	0.1309	1.9807	3.36	0.7040	0.3487	41.77
Emberizidae	*Peucaea sumichrasti*	IndoMalay	0	-0.0393	-	3.21	0.8097	0.5192	12.71
Emberizidae	*Pezopetes capitalis*	Australasia	0	-0.0488	-	2.79	0.7544	0.1900	24.92
Emberizidae	*Phrygilus alaudinus*	Nearctic	0	-0.6430	-	5.34	0.8465	0.0413	0.68
Emberizidae	*Phrygilus atriceps*	Neotropics	0	-0.5994	9.8639	1.88	0.7493	0.3593	82.35
Emberizidae	*Phrygilus carbonarius*	Neotropics	0	-0.0044	-	1.59	0.6478	0.0412	20.08
Emberizidae	*Phrygilus dorsalis*	Neotropics	0	-0.0044	6.0288	3.18	0.6986	0.1859	65.97
Emberizidae	*Phrygilus erythronotus*	Neotropics	0	-0.2583	17.1900	3.96	0.7857	0.3424	64.16
Emberizidae	*Phrygilus fruticeti*	IndoMalay	0	-0.0244	8.4306	2.64	0.7300	0.2214	67.86
Emberizidae	*Phrygilus gayi*	IndoMalay	1	-0.0645	4.3230	2.63	0.6961	0.8096	10.51
Emberizidae	*Phrygilus patagonicus*	IndoMalay	0	-0.0030	-	2.64	0.7327	0.2318	67.52
Emberizidae	*Phrygilus plebejus*	Australasia	0	-0.5735	-	3.32	0.8323	0.5559	6.35
Emberizidae	*Phrygilus punensis*	Australasia	0	-0.5630	-	2.64	0.7710	0.2651	4.10
Emberizidae	*Phrygilus unicolor*	Australasia	0	-0.4463	-	3.59	0.8195	0.4725	2.32
Emberizidae	*Pipilo chlorurus*	Palearctic	0	-0.9404	2.6716	5.08	0.8588	0.1247	4.64
Emberizidae	*Pipilo erythrophthalmus*	Palearctic	0	-32.2251	7.1353	4.52	0.7359	0.5288	19.42
Emberizidae	*Pipilo maculatus*	Afrotropics	0	0.0094	16.2615	3.48	0.7825	0.3406	64.97
Emberizidae	*Pipilo ocai*	IndoMalay	0	-0.1716	9.4411	3.86	0.7580	0.1941	15.57
Emberizidae	*Platyspiza crassirostris*	Neotropics	0	0.0279	-	3.26	0.7692	0.2824	48.81
Emberizidae	*Plectrophenax nivalis*	Neotropics	0	-9.6545	4.8096	4.03	0.7144	0.2410	47.13
Emberizidae	*Pooecetes gramineus*	Australasia	0	-0.1487	-	2.64	0.7192	0.1844	10.74
Emberizidae	*Poospiza alticola*	Australasia	0	-0.1289	-	3.34	0.7597	0.3674	4.43
Emberizidae	*Poospiza boliviana*	Australasia	0	-0.0851	-	2.79	0.7619	0.2843	21.50
Emberizidae	*Poospiza cabanisi*	Australasia	0	-0.0307	5.1805	2.85	0.7019	0.1316	20.53

Family	Species	Realm	Threat	Latitude	Elevation	Anomaly	Size	Shape	Orientation
Emberizidae	*Poospiza caesar*	Australasia	0	-0.1728	-	3.15	0.7416	0.5389	64.35
Emberizidae	*Poospiza cinerea*	Neotropics	0	0.1354	4.9935	4.15	0.6523	0.1973	18.77
Emberizidae	*Poospiza erythrophrys*	Neotropics	0	-20.2738	5.2137	3.54	0.7352	0.9377	5.61
Emberizidae	*Poospiza hispaniolensis*	Neotropics	0	0.1329	4.5193	3.78	0.7645	0.5116	28.69
Emberizidae	*Poospiza hypochondria*	Neotropics	0	-0.0103	1.3000	3.86	0.8032	0.6085	40.23
Emberizidae	*Poospiza lateralis*	Neotropics	0	-0.1025	3.7084	4.07	0.8263	0.4297	32.64
Emberizidae	*Poospiza melanoleuca*	Neotropics	1	0.0000	4.2339	3.61	0.5985	0.5774	45.00
Emberizidae	*Poospiza nigrorufa*	Neotropics	0	-0.4065	10.8249	3.20	0.7955	0.6335	4.88
Emberizidae	*Poospiza ornata*	Neotropics	0	0.0000	5.6624	4.14	0.6428	0.5978	20.56
Emberizidae	*Poospiza rubecula*	Neotropics	0	0.1318	6.3881	3.35	0.7444	0.1897	57.62
Emberizidae	*Poospiza thoracica*	Neotropics	0	0.0000	7.2697	4.43	0.6716	0.6424	24.97
Emberizidae	*Poospiza torquata*	Neotropics	0	-0.0921	0.2251	4.37	0.6221	0.5728	71.79
Emberizidae	*Porphyrospiza caerulescens*	Australasia	0	-0.3085	-	3.33	0.7826	0.2887	3.67
Emberizidae	*Pselliophorus luteoviridis*	Neotropics	0	0.0404	8.2158	3.65	0.7660	0.3322	79.72
Emberizidae	*Pselliophorus tibialis*	Neotropics	0	-0.1754	2.5842	4.00	0.7793	0.7275	19.88
Emberizidae	*Rhodospingus cruentus*	Afrotropics	0	-0.0054	-	3.31	0.7785	0.4591	84.95
Emberizidae	*Rhynchophanes mccownii*	IndoMalay	0	-0.0713	-	3.11	0.8045	0.3460	28.97
Emberizidae	*Rhynchospiza stolzmanni*	Australasia	0	-0.0291	3.8397	2.73	0.6523	0.2433	19.99
Emberizidae	*Rhynchospiza strigiceps*	Australasia	0	-0.1992	8.7551	2.86	0.7205	0.1598	20.14
Emberizidae	*Saltatricula multicolor*	Palearctic	0	-0.1869	-	3.88	0.7846	0.1522	4.08
Emberizidae	*Sicalis auriventris*	Afrotropics	0	-0.1186	9.6464	3.54	0.8639	0.7650	39.09
Emberizidae	*Sicalis citrina*	Palearctic	0	-0.6921	11.6374	4.04	0.8295	0.2325	3.84
Emberizidae	*Sicalis columbiana*	Afrotropics	0	0.1432	-	3.30	0.8164	0.6891	15.22
Emberizidae	*Sicalis flaveola*	Afrotropics	0	-0.0035	6.7618	3.42	0.8289	0.4886	33.71
Emberizidae	*Sicalis lebruni*	Afrotropics	1	0.3720	0.0000	3.08	0.7500	0.2937	6.05
Emberizidae	*Sicalis lutea*	Neotropics	0	0.7206	5.0663	3.97	0.6598	0.7658	38.79
Emberizidae	*Sicalis luteocephala*	Neotropics	0	-29.4801	9.4722	3.53	0.6716	0.2139	59.62
Emberizidae	*Sicalis luteola*	Neotropics	0	-16.4886	4.8732	3.74	0.6804	0.2056	63.81
Emberizidae	*Sicalis olivascens*	Neotropics	0	-0.2392	11.0786	3.04	0.6784	0.1941	29.99
Emberizidae	*Sicalis raimondii*	Neotropics	0	0.3502	7.3511	3.54	0.7495	0.2582	77.45
Emberizidae	*Sicalis taczanowskii*	Neotropics	0	4.0733	10.2457	4.04	0.7151	0.1644	35.81
Emberizidae	*Sicalis uropygialis*	Neotropics	1	0.0000	3.9928	3.17	0.5985	0.5771	46.13
Emberizidae	*Spizella arborea*	Afrotropics	0	-0.3886	-	3.78	0.7867	0.3876	51.03
Emberizidae	*Spizella atrogularis*	Afrotropics	1	0.0127	9.4717	3.62	0.7136	0.4488	88.73
Emberizidae	*Spizella breweri*	Afrotropics	0	-0.1117	-	3.77	0.8264	0.3834	60.59
Emberizidae	*Spizella pallida*	Afrotropics	0	9.2256	-	3.16	0.7277	0.6619	72.93
Emberizidae	*Spizella passerina*	Afrotropics	0	0.0555	8.8045	3.32	0.7657	0.3891	14.49
Emberizidae	*Spizella pusilla*	Afrotropics	0	-0.4403	5.9086	3.45	0.7716	0.4005	38.98
Emberizidae	*Spizella wortheni*	Afrotropics	0	-0.1438	-	3.46	0.7781	0.4376	20.07
Emberizidae	*Sporophila albogularis*	Afrotropics	0	-0.0160	6.2321	3.31	0.7643	0.7955	24.12
Emberizidae	*Sporophila americana*	Afrotropics	0	-0.2605	8.0203	3.54	0.8182	0.3419	52.17
Emberizidae	*Sporophila ardesiaca*	Afrotropics	0	-0.0612	6.0672	3.38	0.7482	0.2794	84.00
Emberizidae	*Sporophila bouvreuil*	Afrotropics	0	-2.1650	-	3.32	0.7158	0.4058	46.25
Emberizidae	*Sporophila bouvronides*	Afrotropics	0	-0.8601	-	2.89	0.7060	0.2720	3.47
Emberizidae	*Sporophila caerulescens*	Afrotropics	0	0.0793	-	3.60	0.8177	0.1671	6.00
Emberizidae	*Sporophila castaneiventris*	Afrotropics	0	-2.4766	8.6815	3.39	0.7185	0.4401	68.07
Emberizidae	*Sporophila cinnamomea*	Afrotropics	0	0.1775	7.5830	3.71	0.7144	0.2115	58.73
Emberizidae	*Sporophila collaris*	Afrotropics	0	-0.1945	-	3.68	0.7977	0.8808	26.14
Emberizidae	*Sporophila corvina*	Afrotropics	0	-0.1138	-	3.54	0.8479	0.5236	38.87
Emberizidae	*Sporophila falcirostris*	Afrotropics	0	0.0547	13.6398	3.60	0.7300	0.3762	86.05
Emberizidae	*Sporophila frontalis*	Palearctic	0	-1.0903	14.2976	4.25	0.8109	0.2866	0.79
Emberizidae	*Sporophila hypochroma*	Afrotropics	0	3.9084	-	3.61	0.7861	0.3957	66.10
Emberizidae	*Sporophila hypoxantha*	Afrotropics	0	0.0063	10.2609	3.80	0.7245	0.2004	63.86
Emberizidae	*Sporophila intermedia*	Afrotropics	0	-0.5403	-	3.15	0.7416	0.3619	42.25
Emberizidae	*Sporophila leucoptera*	Palearctic	0	-0.6621	-	3.77	0.8265	0.4461	16.55
Emberizidae	*Sporophila lineola*	Afrotropics	0	-0.0321	17.0223	3.43	0.7829	0.3911	67.89
Emberizidae	*Sporophila luctuosa*	Afrotropics	0	-0.1300	-	3.53	0.8102	0.5580	78.63
Emberizidae	*Sporophila melanogaster*	Afrotropics	0	19.0987	3.6623	3.47	0.6824	0.5514	51.07
Emberizidae	*Sporophila melanops*	Palearctic	1	-0.5047	5.0175	3.63	0.6428	0.1847	83.25
Emberizidae	*Sporophila minuta*	Palearctic	0	-0.6189	7.1033	4.23	0.7836	0.4798	12.22
Emberizidae	*Sporophila murallae*	Afrotropics	0	-0.7905	-	2.86	0.7060	0.3391	8.63
Emberizidae	*Sporophila nigricollis*	Afrotropics	0	0.0323	12.7218	3.57	0.7584	0.9399	42.72
Emberizidae	*Sporophila nigrorufa*	Afrotropics	0	1.0865	-	3.45	0.7078	0.4525	58.49
Emberizidae	*Sporophila nigrorufa*	Afrotropics	0	1.0865	-	3.45	0.7078	-	-
Emberizidae	*Sporophila palustris*	Afrotropics	1	-9.4475	3.0394	3.54	0.6370	-	-
Emberizidae	*Sporophila palustris*	Afrotropics	1	-9.4475	3.0394	3.54	0.6370	0.1502	54.71
Emberizidae	*Sporophila peruviana*	Afrotropics	0	0.0601	-	3.59	0.7314	0.3482	88.46
Emberizidae	*Sporophila plumbea*	Neotropics	0	0.0387	14.9983	3.58	0.7776	0.4155	77.83
Emberizidae	*Sporophila ruficollis*	Neotropics	0	0.4654	1.3919	4.07	0.7808	0.6183	4.34
Emberizidae	*Sporophila schistacea*	Neotropics	0	3.8582	-	3.42	0.7663	0.1596	78.24
Emberizidae	*Sporophila simplex*	Neotropics	0	-0.5308	3.0534	3.07	0.8182	0.4103	41.09
Emberizidae	*Sporophila telasco*	Neotropics	0	-0.4221	6.0507	3.23	0.8279	0.5403	41.80
Emberizidae	*Sporophila torqueola*	Nearctic	0	-0.6134	-	4.16	0.8387	0.3267	14.29
Emberizidae	*Tiaris bicolor*	Afrotropics	1	0.0000	3.2607	3.44	0.6523	0.6101	63.71

Family	Species	Realm	Threat	Latitude	Elevation	Anomaly	Size	Shape	Orientation
Emberizidae	*Tiaris canorus*	Afrotropics	0	-0.0956	10.7320	3.69	0.8026	0.5370	13.27
Emberizidae	*Tiaris fuliginosus*	Afrotropics	0	0.5066	11.7716	3.38	0.8042	0.6658	32.80
Emberizidae	*Tiaris obscurus*	Afrotropics	0	-0.0991	-	3.60	0.8426	0.6254	40.20
Emberizidae	*Tiaris olivaceus*	Afrotropics	0	-0.1008	-	3.30	0.7892	0.5447	37.42
Emberizidae	*Torreornis inexpectata*	Neotropics	0	0.0917	-	4.11	0.7883	0.7257	63.09
Emberizidae	*Urothraupis stolzmanni*	Palearctic	0	-0.5182	0.0000	4.27	0.7363	0.2529	31.34
Emberizidae	*Volatinia jacarina*	Australasia	1	0.1402	1.4799	3.08	0.6428	0.2943	69.18
Emberizidae	*Xenospingus concolor*	Australasia	1	-0.6229	5.8660	2.49	0.6662	0.3153	20.62
Emberizidae	*Xenospiza baileyi*	Afrotropics	0	-0.1006	15.5595	3.54	0.8127	0.6633	68.92
Emberizidae	*Zonotrichia albicollis*	Afrotropics	0	-0.0768	-	3.59	0.8505	0.5785	35.03
Emberizidae	*Zonotrichia atricapilla*	Afrotropics	0	-0.2715	-	3.55	0.8428	0.4267	3.60
Emberizidae	*Zonotrichia capensis*	Afrotropics	0	0.1363	-	3.29	0.7750	0.1279	9.51
Emberizidae	*Zonotrichia leucophrys*	Afrotropics	0	-0.0274	-	3.61	0.8309	0.2009	9.77
Emberizidae	*Zonotrichia querula*	Australasia	0	-0.6231	-	3.30	0.8307	0.4866	2.59
Estrildidae	*Amadina erythrocephala*	Afrotropics	0	-0.3027	16.2224	3.61	0.8611	0.7834	55.72
Estrildidae	*Amadina fasciata*	Afrotropics	0	-0.2032	-	4.06	0.8036	0.5592	45.12
Estrildidae	*Amandava amandava*	Australasia	0	-0.6799	5.5718	2.80	0.6784	0.0694	26.24
Estrildidae	*Amandava formosa*	IndoMalay	0	-0.4260	-	3.31	0.8245	0.3929	8.42
Estrildidae	*Amandava subflava*	IndoMalay	1	0.0222	-	3.50	0.7606	0.7274	87.29
Estrildidae	*Clytospiza monteiri*	Neotropics	0	0.0056	10.9487	3.52	0.7356	0.1703	32.60
Estrildidae	*Cryptospiza jacksoni*	Afrotropics	0	0.0640	5.3387	3.45	0.8198	0.2899	7.86
Estrildidae	*Cryptospiza reichenovii*	Afrotropics	0	0.1462	-	3.45	0.7914	0.8086	29.70
Estrildidae	*Cryptospiza salvadorii*	Afrotropics	0	0.0128	-	3.12	0.7681	0.1857	4.05
Estrildidae	*Cryptospiza shelleyi*	Afrotropics	0	-0.0620	-	3.27	0.8170	0.2993	14.08
Estrildidae	*Emblema pictum*	Palearctic	0	-4.9915	6.0625	4.56	0.7120	0.6130	36.29
Estrildidae	*Erythrura coloria*	Neotropics	0	0.0876	11.7299	3.66	0.7286	0.2168	64.38
Estrildidae	*Erythrura coloria*	Neotropics	0	0.0876	11.7299	3.66	0.7286	-	-
Estrildidae	*Erythrura hyperythra*	Neotropics	0	2.1413	12.6842	3.19	0.7019	0.1769	63.89
Estrildidae	*Erythrura kleinschmidti*	Neotropics	1	2.1413	15.4552	3.11	0.6120	0.6185	89.76
Estrildidae	*Erythrura papuana*	Neotropics	0	-0.0226	4.2953	3.45	0.7281	0.1514	59.03
Estrildidae	*Erythrura pealii*	Palearctic	0	-0.9064	-	3.52	0.7505	0.2964	57.85
Estrildidae	*Erythrura prasina*	Palearctic	0	-0.6607	4.6681	4.40	0.8477	0.3491	10.32
Estrildidae	*Erythrura psittacea*	IndoMalay	0	-0.1178	11.6737	3.13	0.8216	0.6094	51.33
Estrildidae	*Erythrura regia*	Afrotropics	0	0.1985	-	3.09	0.7344	0.1990	61.25
Estrildidae	*Erythrura trichroa*	Afrotropics	0	-0.1575	-	3.45	0.7858	0.5651	60.56
Estrildidae	*Erythrura tricolor*	Afrotropics	0	0.0858	-	3.23	0.8069	0.2138	13.41
Estrildidae	*Erythrura viridifacies*	Australasia	0	-0.4414	0.0000	3.35	0.8302	0.5698	10.19
Estrildidae	*Estrilda astrild*	Palearctic	0	-0.5030	3.4712	3.97	0.8373	0.3664	6.11
Estrildidae	*Estrilda atricapilla*	Afrotropics	0	0.0131	10.4259	3.28	0.7727	0.2432	10.42
Estrildidae	*Estrilda caerulescens*	Afrotropics	0	-0.1549	6.4104	3.55	0.8425	0.7037	53.68
Estrildidae	*Estrilda charmosyna*	Afrotropics	0	0.0438	6.4539	3.26	0.7845	0.3075	12.46
Estrildidae	*Estrilda erythronotos*	Afrotropics	0	-0.2345	7.0122	4.20	0.8005	0.6864	23.13
Estrildidae	*Estrilda melanotis*	Afrotropics	0	-0.0837	4.9910	3.55	0.7991	0.4505	59.86
Estrildidae	*Estrilda melpoda*	Afrotropics	0	-0.5317	-	3.24	0.7382	0.3150	62.21
Estrildidae	*Estrilda nigriloris*	Australasia	0	-0.1246	5.7423	2.81	0.7498	0.2165	25.56
Estrildidae	*Estrilda nonnula*	Australasia	1	-0.4076	2.9910	3.26	0.7850	0.3733	21.69
Estrildidae	*Estrilda paludicola*	IndoMalay	0	-0.1010	2.7383	2.64	0.6716	0.8413	51.59
Estrildidae	*Estrilda perreini*	Australasia	1	-0.0997	-	3.36	0.7234	0.2745	0.49
Estrildidae	*Estrilda poliopareia*	IndoMalay	0	-0.0499	11.0721	2.74	0.7560	0.6219	68.60
Estrildidae	*Estrilda rufibarba*	Australasia	0	-0.0545	-	2.77	0.7382	0.1560	24.12
Estrildidae	*Estrilda troglodytes*	IndoMalay	0	-0.0123	-	2.86	0.7959	0.6326	51.28
Estrildidae	*Euschistospiza cinereovinacea*	Afrotropics	0	-0.1803	-	4.11	0.7984	0.4515	19.09
Estrildidae	*Euschistospiza dybowskii*	Afrotropics	0	-0.0397	6.4982	3.32	0.8042	0.4961	54.80
Estrildidae	*Heteromunia pectoralis*	Neotropics	0	-0.0209	2.4032	3.86	0.8034	0.2751	9.67
Estrildidae	*Hypargos margaritatus*	Nearctic	0	-0.8770	2.0808	4.23	0.8187	0.5791	19.80
Estrildidae	*Hypargos niveoguttatus*	Australasia	0	0.5197	12.4301	2.71	0.7060	0.4844	57.70
Estrildidae	*Lagonosticta landanae*	IndoMalay	1	-0.0991	-	2.66	0.5985	0.2252	62.36
Estrildidae	*Lagonosticta larvata*	Neotropics	0	-0.2000	-	3.90	0.8471	0.5782	58.33
Estrildidae	*Lagonosticta nitidula*	IndoMalay	0	-0.1786	8.3263	2.84	0.7019	0.1810	66.84
Estrildidae	*Lagonosticta rara*	Neotropics	1	-0.4128	4.3514	3.31	0.7570	0.3063	16.60
Estrildidae	*Lagonosticta rhodopareia*	Neotropics	0	0.0540	2.7589	3.98	0.8316	0.6409	1.82
Estrildidae	*Lagonosticta rubricata*	Neotropics	0	-0.2445	2.6579	3.75	0.8546	0.6942	52.25
Estrildidae	*Lagonosticta rufopicta*	Neotropics	0	-0.1761	-	3.32	0.7852	0.3831	8.11
Estrildidae	*Lagonosticta sanguinodorsalis*	Afrotropics	0	0.0782	-	3.25	0.8059	0.2550	12.06
Estrildidae	*Lagonosticta senegala*	Nearctic	0	-0.8470	2.2987	4.23	0.8484	0.3171	9.87
Estrildidae	*Lagonosticta virata*	Nearctic	0	-0.1824	17.7167	3.91	0.7705	0.2736	42.63
Estrildidae	*Lonchura atricapilla*	Australasia	0	-0.3398	-	3.22	0.7531	0.4295	53.78
Estrildidae	*Lonchura bicolor*	Australasia	0	-0.4085	6.0817	3.09	0.6921	0.2882	61.53
Estrildidae	*Lonchura caniceps*	Australasia	0	-0.5681	-	3.15	0.7806	0.4887	77.60
Estrildidae	*Lonchura cantans*	Australasia	0	-0.5611	-	3.10	0.6302	0.5670	90.00
Estrildidae	*Lonchura castaneothorax*	Australasia	0	-0.4858	-	3.62	0.8131	0.3921	1.76
Estrildidae	*Lonchura cucullata*	Australasia	0	-0.5952	-	3.07	0.8028	0.3317	3.39
Estrildidae	*Lonchura ferruginosa*	Australasia	0	-0.6107	-	3.14	0.7739	0.4457	62.15
Estrildidae	*Lonchura flaviprymna*	Australasia	0	-0.0666	-	2.78	0.7482	0.1742	26.40

Family	Species	Realm	Threat	Latitude	Elevation	Anomaly	Size	Shape	Orientation
Estrildidae	*Lonchura forbesi*	Australasia	0	-0.9184	-	2.87	0.7877	0.1576	5.61
Estrildidae	*Lonchura fringilloides*	Australasia	0	-0.6818	-	3.44	0.8272	0.4176	14.89
Estrildidae	*Lonchura fuscans*	Australasia	0	-0.5534	-	3.41	0.8302	0.5704	3.57
Estrildidae	*Lonchura grandis*	Australasia	0	-0.1043	12.8158	2.82	0.7392	0.1582	23.62
Estrildidae	*Lonchura griseicapilla*	Australasia	0	-0.3116	-	3.32	0.7870	0.3116	3.89
Estrildidae	*Lonchura kelaarti*	Australasia	0	-0.1452	-	2.79	0.7378	0.4886	59.05
Estrildidae	*Lonchura leucogastra*	Australasia	0	-0.5455	-	3.36	0.8368	0.5496	7.97
Estrildidae	*Lonchura leucogastroides*	Australasia	0	-0.0376	-	2.69	0.7144	0.1537	33.76
Estrildidae	*Lonchura leucosticta*	Australasia	0	-0.0376	-	2.48	0.6523	0.1989	64.35
Estrildidae	*Lonchura maja*	Australasia	0	-0.3065	6.9719	2.49	0.5788	0.0000	0.00
Estrildidae	*Lonchura malabarica*	Australasia	0	-0.1329	-	2.56	0.6523	0.1875	30.24
Estrildidae	*Lonchura malacca*	Australasia	0	-0.2987	-	2.58	0.6302	0.1134	34.50
Estrildidae	*Lonchura melaena*	Australasia	0	-0.3761	-	3.44	0.8285	0.5061	1.49
Estrildidae	*Lonchura molucca*	Australasia	0	0.1796	-	2.60	0.6906	0.1485	3.36
Estrildidae	*Lonchura montana*	Australasia	0	0.2470	-	2.63	0.6523	0.0000	0.00
Estrildidae	*Lonchura monticola*	Australasia	0	0.3803	-	2.48	0.6120	0.0000	0.00
Estrildidae	*Lonchura nevermanni*	Palearctic	0	-1.5561	-	5.80	0.7901	0.0539	2.53
Estrildidae	*Lonchura nigriceps*	Neotropics	0	-0.6391	1.5841	2.24	0.7286	0.2722	26.27
Estrildidae	*Lonchura pallida*	Nearctic	0	-0.4705	-	4.40	0.8098	0.1416	15.09
Estrildidae	*Lonchura punctulata*	Palearctic	0	-1.1779	-	6.02	0.7864	0.0156	0.18
Estrildidae	*Lonchura quinticolor*	Palearctic	0	-0.5839	-	4.81	0.8131	0.1377	8.73
Estrildidae	*Lonchura spectabilis*	Neotropics	0	-0.6805	0.1123	2.27	0.7374	0.5462	16.78
Estrildidae	*Lonchura striata*	Nearctic	0	-0.7469	1.9810	3.90	0.7866	0.3871	9.59
Estrildidae	*Lonchura stygia*	Nearctic	0	-0.4949	-	4.39	0.7879	0.3064	7.09
Estrildidae	*Lonchura teerinki*	Nearctic	0	0.5758	-	4.32	0.7539	0.0910	18.26
Estrildidae	*Lonchura tristissima*	Palearctic	0	-0.9291	0.7047	6.23	0.8032	0.0288	0.36
Estrildidae	*Lonchura vana*	Palearctic	0	-0.6950	-	4.83	0.8491	0.1364	1.58
Estrildidae	*Mandingoa nitidula*	Australasia	0	-0.1300	2.8689	2.48	0.6302	0.0421	37.95
Estrildidae	*Neochmia modesta*	Afrotropics	0	0.0026	10.9402	3.44	0.7318	0.2799	80.82
Estrildidae	*Neochmia phaeton*	Afrotropics	0	-0.1328	-	3.59	0.8244	0.7616	68.89
Estrildidae	*Neochmia ruficauda*	IndoMalay	0	-0.0856	-	3.43	0.8316	0.3729	12.70
Estrildidae	*Neochmia temporalis*	Australasia	0	-0.0643	3.2467	2.74	0.7796	0.2061	11.55
Estrildidae	*Nesocharis ansorgei*	Afrotropics	0	0.0305	-	3.37	0.8160	0.7579	4.43
Estrildidae	*Nesocharis capistrata*	Afrotropics	1	0.0000	6.7873	3.27	0.6302	0.4947	37.39
Estrildidae	*Nesocharis shelleyi*	Afrotropics	0	0.0400	-	3.25	0.8064	0.2376	12.41
Estrildidae	*Nigrita bicolor*	Australasia	0	-0.3268	-	3.37	0.7845	0.3695	1.71
Estrildidae	*Nigrita canicapillus*	Australasia	0	-0.5072	-	3.09	0.7840	0.4875	89.20
Estrildidae	*Nigrita fusconotus*	Afrotropics	0	0.3799	-	3.64	0.7507	0.2553	25.62
Estrildidae	*Nigrita luteifrons*	Afrotropics	0	0.0233	6.2437	3.25	0.8098	0.2662	11.49
Estrildidae	*Oreostruthus fuliginosus*	Neotropics	0	-0.6626	6.5433	3.77	0.6562	0.3333	9.07
Estrildidae	*Ortygospiza atricollis*	Neotropics	0	-0.3931	6.1334	3.43	0.8028	0.2470	83.57
Estrildidae	*Ortygospiza locustella*	Neotropics	0	-34.4113	7.7478	4.01	0.7030	0.5977	90.00
Estrildidae	*Padda fuscata*	Nearctic	0	-0.5786	8.8051	4.02	0.7981	0.3018	53.39
Estrildidae	*Padda oryzivora*	IndoMalay	0	0.1129	-	2.52	0.5985	0.0024	45.41
Estrildidae	*Parmoptila jamesoni*	Australasia	0	-0.1065	-	2.75	0.7050	0.2801	19.18
Estrildidae	*Parmoptila rubrifrons*	Australasia	0	0.7184	-	2.63	0.6478	0.0000	0.00
Estrildidae	*Parmoptila woodhousei*	Australasia	0	-0.5820	-	2.74	0.7464	0.4639	49.44
Estrildidae	*Poephila acuticauda*	Afrotropics	0	-0.1597	-	3.45	0.7399	0.4346	82.75
Estrildidae	*Poephila cincta*	Afrotropics	0	-0.1185	-	3.38	0.8039	0.6779	43.48
Estrildidae	*Poephila personata*	Afrotropics	0	-0.0614	-	3.15	0.7452	0.3731	45.31
Estrildidae	*Pyrenestes minor*	Australasia	1	-1.2233	0.0000	2.53	0.6662	0.0342	9.33
Estrildidae	*Pyrenestes sanguineus*	Australasia	1	-0.9799	1.7237	2.73	0.7060	0.1063	8.74
Estrildidae	*Pytilia afra*	Australasia	0	-0.0983	-	2.78	0.7709	0.3095	23.61
Estrildidae	*Pytilia melba*	IndoMalay	0	-0.1511	4.7483	2.78	0.7136	0.1498	26.92
Estrildidae	*Pytilia phoenicoptera*	Australasia	0	-0.0704	-	2.77	0.7613	0.1944	23.11
Estrildidae	*Spermophaga haematina*	Neotropics	0	0.3908	-	4.06	0.7787	0.9531	37.20
Estrildidae	*Spermophaga poliogenys*	Neotropics	0	-0.0953	2.4978	4.03	0.7835	0.7498	35.12
Estrildidae	*Spermophaga ruficapilla*	Neotropics	0	0.1091	-	4.00	0.7994	0.7522	24.89
Estrildidae	*Stagonopleura bella*	Palearctic	0	0.9180	8.9128	4.44	0.7477	0.4780	13.07
Estrildidae	*Stagonopleura guttata*	IndoMalay	0	-0.0901	-	3.31	0.8062	0.4261	10.66
Estrildidae	*Stagonopleura oculata*	Palearctic	0	-0.8746	-	4.55	0.8692	0.2168	1.94
Estrildidae	*Taeniopygia bichenovii*	Neotropics	0	-0.0200	11.2261	3.69	0.7659	0.3749	73.51
Estrildidae	*Taeniopygia guttata*	Palearctic	0	-0.5049	5.4340	4.01	0.8574	0.2089	9.74
Estrildidae	*Uraeginthus angolensis*	Neotropics	0	-0.2216	3.6487	3.57	0.7261	0.2276	31.15
Estrildidae	*Uraeginthus bengalus*	Neotropics	0	-0.2338	4.9193	3.05	0.6784	0.1760	30.09
Estrildidae	*Uraeginthus cyanocephalus*	Neotropics	0	-0.1053	-	3.92	0.8375	0.4630	34.66
Estrildidae	*Uraeginthus granatinus*	Neotropics	0	0.5097	4.1175	2.97	0.7078	0.1831	67.84
Estrildidae	*Uraeginthus ianthinogaster*	Neotropics	0	-0.1136	-	3.95	0.8385	0.5334	5.54
Eupetidae	*Androphobus viridis*	Australasia	0	-0.2044	7.8864	2.82	0.6876	0.2831	5.12
Eupetidae	*Eupetes macrocerus*	Neotropics	0	-0.5564	2.3835	3.29	0.7446	0.4660	60.80
Eupetidae	*Psophodes occidentalis*	Australasia	0	-0.4779	-	2.45	0.7128	0.3928	46.81
Eupetidae	*Psophodes olivaceus*	Nearctic	0	-0.8352	-	4.15	0.8077	0.6606	12.64
Eupetidae	*Ptilorrhoa caerulescens*	IndoMalay	0	-0.1015	3.3036	3.14	0.7969	0.4082	42.05
Eupetidae	*Ptilorrhoa castanonota*	IndoMalay	0	-0.2365	7.0122	4.11	0.7435	0.1483	22.79

Family	Species	Realm	Threat	Latitude	Elevation	Anomaly	Size	Shape	Orientation
Eupetidae	*Ptilorrhoa leucosticta*	Afrotropics	0	-0.5989	-	3.52	0.8440	0.2863	4.57
Eurylaimidae	*Calyptomena hosii*	Neotropics	0	-0.0540	5.1166	3.66	0.7305	0.2590	80.01
Eurylaimidae	*Calyptomena viridis*	Neotropics	0	-0.2183	-	3.85	0.7300	0.1633	57.50
Eurylaimidae	*Calyptomena whiteheadi*	Neotropics	0	0.2487	-	3.53	0.7517	0.1251	21.44
Eurylaimidae	*Corydon sumatranus*	Australasia	0	-0.2795	4.7935	2.50	0.5788	0.0000	45.57
Eurylaimidae	*Cymbirhynchus macrorhynchos*	Neotropics	0	-1.9867	5.9591	3.99	0.7455	0.1600	39.43
Eurylaimidae	*Eurylaimus javanicus*	Afrotropics	0	-0.6193	-	3.08	0.7286	0.4435	17.86
Eurylaimidae	*Eurylaimus ochromalus*	Afrotropics	0	-0.4643	-	4.11	0.8012	0.7305	48.66
Eurylaimidae	*Eurylaimus samarensis*	Afrotropics	0	-0.4411	9.0210	3.68	0.7446	0.3942	47.37
Eurylaimidae	*Eurylaimus steerii*	Afrotropics	0	0.0549	6.9102	3.31	0.7993	0.4752	51.56
Eurylaimidae	*Psarisomus dalhousiae*	IndoMalay	0	-0.4559	-	3.28	0.8372	0.6577	34.74
Eurylaimidae	*Pseudocalyptomena graueri*	Afrotropics	1	-16.0027	5.2751	3.26	0.6631	0.2489	23.31
Eurylaimidae	*Serilophus lunatus*	Afrotropics	0	-0.0400	-	3.57	0.8011	0.7306	41.39
Eurylaimidae	*Smithornis capensis*	IndoMalay	0	-0.2135	-	3.30	0.8150	0.9276	8.58
Eurylaimidae	*Smithornis rufolateralis*	IndoMalay	0	-0.0758	-	2.73	0.7217	0.1692	39.93
Eurylaimidae	*Smithornis sharpei*	IndoMalay	0	0.0773	7.7953	2.82	0.7637	0.4537	9.01
Eurypygidae	*Eurypyga helias*	Afrotropics	0	-0.0918	-	3.54	0.8498	0.5535	32.92
Falconidae	*Caracara cheriway*	Palearctic	0	-0.6701	6.3472	3.70	0.7796	0.5965	2.72
Falconidae	*Caracara plancus*	Afrotropics	0	-0.2374	9.0227	4.10	0.8142	0.8065	69.36
Falconidae	*Daptrius ater*	Neotropics	0	-0.1940	-	3.91	0.8161	0.7036	23.32
Falconidae	*Falco alopex*	IndoMalay	0	-0.0605	3.0369	3.03	0.8034	0.4759	24.50
Falconidae	*Falco amurensis*	Australasia	0	-0.4082	6.3792	3.29	0.7816	0.4250	84.18
Falconidae	*Falco ardosiaceus*	Australasia	0	-0.0982	0.8707	2.77	0.7670	0.3151	24.32
Falconidae	*Falco berigora*	IndoMalay	0	0.0484	2.0727	2.84	0.7874	0.4345	0.52
Falconidae	*Falco biarmicus*	Afrotropics	1	-0.1733	4.2847	2.95	0.6842	0.2310	75.95
Falconidae	*Falco cenchroides*	IndoMalay	0	-0.0320	-	2.90	0.8055	0.5585	60.26
Falconidae	*Falco cherrug*	IndoMalay	0	0.0285	-	2.85	0.7894	0.5664	19.59
Falconidae	*Falco chicquera*	IndoMalay	1	-0.1427	2.8399	2.57	0.6221	0.2612	67.30
Falconidae	*Falco columbarius*	IndoMalay	1	-0.0735	2.9532	2.63	0.6961	0.8096	10.51
Falconidae	*Falco concolor*	Afrotropics	0	-0.3362	-	3.59	0.7490	0.4808	45.09
Falconidae	*Falco cuvierii*	Neotropics	0	-0.0259	-	3.99	0.8421	0.6391	30.87
Falconidae	*Falco deiroleucus*	Australasia	0	-0.0471	1.9187	2.48	0.6221	0.5774	71.57
Falconidae	*Falco dickinsoni*	Afrotropics	0	0.0084	-	3.45	0.8552	0.5353	29.51
Falconidae	*Falco eleonorae*	Afrotropics	0	-0.0267	-	3.25	0.8136	0.2496	14.67
Falconidae	*Falco fasciinucha*	IndoMalay	0	-0.5067	-	3.35	0.8549	0.5845	58.08
Falconidae	*Falco femoralis*	Neotropics	0	-0.1689	7.6288	3.68	0.8337	0.7850	31.74
Falconidae	*Falco hypoleucos*	Neotropics	0	-0.1127	4.4288	3.92	0.8026	0.6839	15.51
Falconidae	*Falco jugger*	Afrotropics	0	-0.0006	-	3.54	0.7323	0.1880	32.18
Falconidae	*Falco longipennis*	Afrotropics	0	-0.2004	-	3.33	0.7799	0.1010	7.98
Falconidae	*Falco mexicanus*	Neotropics	0	-0.0445	-	3.69	0.7626	0.1990	32.71
Falconidae	*Falco moluccensis*	Neotropics	0	-0.0377	6.6667	3.23	0.7637	0.5306	89.59
Falconidae	*Falco naumanni*	Neotropics	0	-3.8467	11.1806	3.85	0.7590	0.2962	62.24
Falconidae	*Falco newtoni*	Afrotropics	1	-0.4029	6.3686	2.98	0.6906	0.2506	78.87
Falconidae	*Falco novaeseelandiae*	Afrotropics	0	0.1305	-	3.54	0.8304	0.1902	4.54
Falconidae	*Falco pelegrinoides*	Palearctic	0	-0.7960	-	4.32	0.8113	0.7705	14.70
Falconidae	*Falco peregrinus*	Afrotropics	0	0.0389	-	3.48	0.8409	0.4418	17.69
Falconidae	*Falco rufigularis*	Australasia	0	-0.3814	-	3.30	0.8401	0.7780	2.85
Falconidae	*Falco rupicoloides*	Afrotropics	0	-0.2901	-	3.68	0.8632	0.6734	56.95
Falconidae	*Falco rusticolus*	Australasia	0	-0.4557	13.3919	3.33	0.8383	0.6287	6.50
Falconidae	*Falco severus*	Palearctic	1	-1.0481	12.7499	4.54	0.8525	0.2968	0.86
Falconidae	*Falco sparverius*	Afrotropics	0	-0.1451	3.9410	3.64	0.8591	0.5163	18.41
Falconidae	*Falco subbuteo*	Palearctic	0	-0.7548	-	4.82	0.8718	0.0659	1.14
Falconidae	*Falco subniger*	Palearctic	0	-0.5832	5.3783	3.84	0.7651	0.3616	1.93
Falconidae	*Falco tinnunculus*	Afrotropics	0	-0.0694	-	3.46	0.8403	0.5215	24.59
Falconidae	*Falco vespertinus*	Neotropics	0	-0.1179	-	3.71	0.8195	0.6531	53.99
Falconidae	*Falco zoniventris*	Afrotropics	0	-0.1629	-	3.72	0.8211	0.6397	15.39
Falconidae	*Herpetotheres cachinnans*	Neotropics	0	-0.0991	-	4.00	0.8317	0.6946	24.71
Falconidae	*Ibycter americanus*	Neotropics	0	0.1129	-	3.20	0.7574	0.4853	52.47
Falconidae	*Micrastur buckleyi*	IndoMalay	0	-0.1668	-	3.38	0.8219	0.2412	3.59
Falconidae	*Micrastur gilvicollis*	Australasia	0	-0.4299	-	3.42	0.8319	0.2961	12.71
Falconidae	*Micrastur mintoni*	Neotropics	0	-0.3395	-	2.53	0.6974	0.1632	17.43
Falconidae	*Micrastur mirandollei*	Nearctic	0	-0.7019	-	4.42	0.8342	0.2807	14.75
Falconidae	*Micrastur plumbeus*	Nearctic	0	-0.9187	-	4.21	0.8408	0.2488	7.10
Falconidae	*Micrastur ruficollis*	Nearctic	0	-0.7650	-	4.17	0.8481	0.4097	10.08
Falconidae	*Micrastur semitorquatus*	Nearctic	0	-0.5360	5.3537	4.24	0.7331	0.5803	33.00
Falconidae	*Microhierax caerulescens*	Australasia	0	-0.2125	6.4483	2.73	0.7112	0.5724	50.70
Falconidae	*Microhierax erythrogenys*	Afrotropics	0	0.0582	6.8084	3.79	0.7993	0.1046	0.92
Falconidae	*Microhierax fringillarius*	Palearctic	0	-0.8756	6.7797	4.18	0.8513	0.6329	11.04
Falconidae	*Microhierax melanoleucos*	Afrotropics	0	-0.0461	-	3.49	0.7775	0.5997	21.97
Falconidae	*Milvago chimachima*	Australasia	0	-0.4123	-	3.36	0.8342	0.5863	8.32
Falconidae	*Milvago chimango*	Palearctic	0	-0.6578	6.4069	4.08	0.8276	0.2850	4.80
Falconidae	*Phalcoboenus albogularis*	Australasia	0	-0.4685	-	2.34	0.7327	0.2586	44.57
Falconidae	*Phalcoboenus australis*	Australasia	0	-0.4642	-	2.77	0.7696	0.3700	3.67
Falconidae	*Phalcoboenus carunculatus*	Australasia	0	-0.5981	5.6397	2.86	0.7673	0.5461	38.46

Family	Species	Realm	Threat	Latitude	Elevation	Anomaly	Size	Shape	Orientation
Falconidae	*Phalcoboenus megalopterus*	Australasia	0	-0.5858	-	2.65	0.7399	0.9132	21.86
Falconidae	*Polihierax insignis*	Afrotropics	0	-0.1698	-	3.05	0.7318	0.1635	76.89
Falconidae	*Polihierax semitorquatus*	Afrotropics	1	0.2017	5.4040	3.16	0.6562	0.1712	54.23
Falconidae	*Spiziapteryx circumcincta*	Afrotropics	0	0.0568	10.0580	3.50	0.7710	0.4401	77.24
Falcunculidae	*Eulacestoma nigropectus*	Afrotropics	0	-0.0289	-	3.42	0.7981	0.4789	74.98
Falcunculidae	*Falcunculus frontatus*	Afrotropics	0	-0.1183	16.7960	3.42	0.7908	0.2720	76.02
Formicariidae	*Chamaeza campanisona*	Neotropics	0	-0.1294	1.9107	3.89	0.8154	0.4034	18.90
Formicariidae	*Chamaeza meruloides*	Nearctic	0	-0.3888	6.9509	3.48	0.8071	0.1861	45.43
Formicariidae	*Chamaeza mollissima*	Neotropics	0	-0.0436	-	4.04	0.8025	0.4626	37.85
Formicariidae	*Chamaeza nobilis*	Palearctic	0	-0.6798	-	3.86	0.8236	0.3670	1.65
Formicariidae	*Chamaeza ruficauda*	IndoMalay	0	-0.2484	-	3.01	0.8321	0.3546	36.02
Formicariidae	*Chamaeza turdina*	Australasia	0	-0.0968	-	2.75	0.7783	0.1966	11.23
Formicariidae	*Formicarius analis*	Palearctic	0	-0.6089	0.0000	4.68	0.8422	0.2632	6.08
Formicariidae	*Formicarius colma*	IndoMalay	0	0.0259	-	3.25	0.7874	0.3437	32.47
Formicariidae	*Formicarius nigricapillus*	Palearctic	0	-0.7456	-	4.55	0.8314	0.3568	8.57
Formicariidae	*Formicarius rufifrons*	Palearctic	0	-0.8965	-	3.68	0.7710	0.3623	6.75
Formicariidae	*Formicarius rufipectus*	IndoMalay	0	-0.1236	-	3.00	0.6842	0.2539	62.46
Formicariidae	*Grallaria albigula*	Neotropics	0	-0.0283	2.1699	4.15	0.8174	0.5370	24.53
Formicariidae	*Grallaria alleni*	Neotropics	0	0.0720	8.1412	3.52	0.7522	0.3066	79.35
Formicariidae	*Grallaria andicolus*	Neotropics	0	-0.2409	4.5620	3.13	0.7661	0.3219	28.26
Formicariidae	*Grallaria blakei*	Neotropics	0	-0.5418	6.8638	2.19	0.7687	0.2469	89.86
Formicariidae	*Grallaria capitalis*	Neotropics	1	0.5535	3.5705	2.99	0.6824	0.1454	62.85
Formicariidae	*Grallaria carrikeri*	Neotropics	0	-0.1864	6.0011	3.73	0.7449	0.1823	47.50
Formicariidae	*Grallaria chthonia*	Neotropics	0	-3.0107	3.1848	3.61	0.6921	0.1665	72.80
Formicariidae	*Grallaria dignissima*	Palearctic	0	-0.2640	7.7925	4.90	0.8513	0.1276	1.68
Formicariidae	*Grallaria eludens*	Afrotropics	0	-0.0812	-	3.61	0.8520	0.7211	40.90
Formicariidae	*Grallaria eludens*	Afrotropics	0	-0.0812	-	3.61	0.8520	-	-
Formicariidae	*Grallaria erythroleuca*	Neotropics	0	-0.2319	-	3.58	0.7498	0.1642	60.47
Formicariidae	*Grallaria erythrotis*	IndoMalay	0	-0.1343	-	3.32	0.8039	0.6285	55.69
Formicariidae	*Grallaria excelsa*	Neotropics	0	-0.5952	6.7640	3.66	0.6716	0.2012	90.00
Formicariidae	*Grallaria fenwickorum*	Neotropics	0	-0.3425	1.4828	2.53	0.6974	0.1632	17.43
Formicariidae	*Grallaria flavotincta*	Afrotropics	0	0.1777	-	3.26	0.7914	0.4108	23.95
Formicariidae	*Grallaria gigantea*	Afrotropics	0	0.0327	-	3.25	0.8053	0.1859	10.45
Formicariidae	*Grallaria gigantea*	Afrotropics	0	0.0327	-	3.25	0.8053	-	-
Formicariidae	*Grallaria griseonucha*	Neotropics	0	0.1384	1.8400	3.09	0.7272	0.2758	55.70
Formicariidae	*Grallaria guatimalensis*	Neotropics	1	-0.1723	1.5767	3.03	0.7144	0.2176	74.57
Formicariidae	*Grallaria haplonota*	Neotropics	0	-0.1251	3.0439	3.97	0.8401	0.6675	35.44
Formicariidae	*Grallaria hypoleuca*	Australasia	0	-0.5674	-	2.86	0.7700	0.5787	30.01
Formicariidae	*Grallaria kaestneri*	Australasia	0	-0.5548	-	2.75	0.7802	0.1713	7.63
Formicariidae	*Grallaria milleri*	Australasia	0	-0.5631	-	3.06	0.7764	0.4710	76.88
Formicariidae	*Grallaria nuchalis*	Australasia	0	-0.0922	-	2.77	0.7709	0.3873	27.19
Formicariidae	*Grallaria przewalskii*	Neotropics	0	-0.0977	-	3.94	0.8386	0.5728	26.79
Formicariidae	*Grallaria quitensis*	Neotropics	0	-0.3158	-	3.53	0.8320	0.5734	40.84
Formicariidae	*Grallaria ridgelyi*	Neotropics	0	-0.1023	4.6185	2.90	0.6120	0.3853	32.40
Formicariidae	*Grallaria ruficapilla*	Neotropics	0	-0.0750	3.3197	2.93	0.6120	0.1543	33.23
Formicariidae	*Grallaria rufocinerea*	Palearctic	1	-0.9612	7.2746	3.28	0.6716	-	-
Formicariidae	*Grallaria rufocinerea*	Palearctic	1	-0.9612	7.2746	3.28	0.6716	0.5737	41.90
Formicariidae	*Grallaria rufula*	Afrotropics	0	-0.0864	0.0000	3.47	0.8412	0.4292	36.43
Formicariidae	*Grallaria squamigera*	IndoMalay	1	-0.7013	-	3.25	0.7794	0.4628	21.61
Formicariidae	*Grallaria varia*	IndoMalay	0	-0.1645	5.5271	2.99	0.7934	0.4663	7.74
Formicariidae	*Grallaria watkinsi*	Australasia	1	-0.1419	0.9007	2.57	0.7104	0.5042	8.44
Formicariidae	*Grallaricula cucullata*	Australasia	1	-0.1494	0.7422	2.87	0.7446	0.2990	17.37
Formicariidae	*Grallaricula ferrugineipectus*	Australasia	1	-0.0794	1.9728	2.72	0.7185	0.1920	20.84
Formicariidae	*Grallaricula flavirostris*	IndoMalay	0	0.2596	-	2.76	0.6478	0.7122	78.50
Formicariidae	*Grallaricula lineifrons*	IndoMalay	0	-0.1006	7.7568	2.99	0.8216	0.5052	44.93
Formicariidae	*Grallaricula loricata*	Afrotropics	0	3.5257	-	3.34	0.7409	0.3171	8.86
Formicariidae	*Grallaricula nana*	Neotropics	0	2.4479	12.5291	3.97	0.7078	0.2545	52.60
Formicariidae	*Grallaricula ochraceifrons*	Neotropics	1	3.5058	4.1960	3.15	0.7060	0.2510	61.25
Formicariidae	*Grallaricula peruviana*	Neotropics	0	-7.6091	7.8727	3.92	0.7363	0.2688	41.60
Formicariidae	*Hylopezus auricularis*	Afrotropics	0	-0.2786	9.6568	3.54	0.8583	0.3947	18.43
Formicariidae	*Hylopezus berlepschi*	Afrotropics	0	2.8331	-	3.95	0.7732	0.9190	39.35
Formicariidae	*Hylopezus dives*	IndoMalay	0	-0.1699	4.5449	2.79	0.8151	0.3586	17.00
Formicariidae	*Hylopezus fulviventris*	Palearctic	0	-0.7730	-	4.33	0.8420	0.0508	0.06
Formicariidae	*Hylopezus macularius*	Afrotropics	0	-1.0742	5.3274	3.21	0.7151	0.7422	33.59
Formicariidae	*Hylopezus nattereri*	Palearctic	0	-0.7442	-	3.72	0.8038	0.5772	8.37
Formicariidae	*Hylopezus ochroleucus*	IndoMalay	1	-0.1170	-	3.35	0.7544	0.3128	28.16
Formicariidae	*Hylopezus perspicillatus*	IndoMalay	0	-0.0736	5.6138	2.77	0.7469	0.2974	46.29
Formicariidae	*Myrmothera campanisona*	Neotropics	0	-0.0820	-	4.01	0.8424	0.7124	40.01
Formicariidae	*Myrmothera simplex*	Neotropics	1	-3.6214	3.4916	3.60	0.6740	0.1558	72.53
Formicariidae	*Pittasoma michleri*	Neotropics	0	0.1199	-	4.13	0.8185	0.5074	11.39
Formicariidae	*Pittasoma rufopileatum*	Neotropics	0	-0.0086	2.1827	3.93	0.8131	0.4133	11.54
Fringillidae	*Bucanetes githagineus*	IndoMalay	1	-0.0196	1.6601	2.64	0.7261	0.2111	67.49
Fringillidae	*Callacanthis burtoni*	Nearctic	0	-1.0258	-	5.21	0.7482	0.8367	33.54
Fringillidae	*Carduelis ambigua*	Neotropics	0	-0.2157	-	3.60	0.7869	0.2551	27.31

Family	Species	Realm	Threat	Latitude	Elevation	Anomaly	Size	Shape	Orientation
Fringillidae	*Carduelis atriceps*	Afrotropics	0	-11.8055	8.9371	3.47	0.7327	0.5151	49.65
Fringillidae	*Carduelis barbata*	Afrotropics	0	-0.1032	7.6852	3.58	0.8294	0.5476	38.66
Fringillidae	*Carduelis cannabina*	Nearctic	0	-0.7834	11.8374	4.34	0.8191	0.5969	12.17
Fringillidae	*Carduelis carduelis*	Neotropics	0	0.3483	3.0224	4.40	0.6891	0.4407	32.21
Fringillidae	*Carduelis chloris*	Neotropics	0	-0.1620	-	3.74	0.8320	0.3948	44.34
Fringillidae	*Carduelis citrinella*	Neotropics	0	-0.2222	4.8383	3.72	0.8224	0.2516	26.77
Fringillidae	*Carduelis corsicana*	Neotropics	0	-0.3877	8.2483	3.31	0.8428	0.3947	51.81
Fringillidae	*Carduelis crassirostris*	Nearctic	0	0.6785	9.6845	4.18	0.7574	0.2418	57.60
Fringillidae	*Carduelis cucullata*	Nearctic	0	-0.6685	-	4.11	0.8326	0.5584	24.79
Fringillidae	*Carduelis dominicensis*	Neotropics	0	0.6403	-	3.45	0.7008	0.1419	6.65
Fringillidae	*Carduelis flammea*	Nearctic	0	-0.5662	-	4.01	0.7951	0.6855	31.13
Fringillidae	*Carduelis flavirostris*	Palearctic	0	-0.0539	10.0154	3.41	0.7832	0.6833	72.01
Fringillidae	*Carduelis hornemanni*	Neotropics	0	-0.3644	6.1965	3.98	0.7803	0.3564	74.53
Fringillidae	*Carduelis johannis*	Neotropics	0	0.0096	4.9313	3.65	0.6986	0.5231	49.71
Fringillidae	*Carduelis lawrencei*	Neotropics	0	-0.7014	-	2.37	0.7862	0.4039	88.12
Fringillidae	*Carduelis magellanica*	Palearctic	0	-0.6028	-	4.28	0.8526	0.2777	2.82
Fringillidae	*Carduelis monguilloti*	Palearctic	0	-0.6826	-	4.27	0.8610	0.4363	20.48
Fringillidae	*Carduelis notata*	Palearctic	0	-0.6064	-	4.12	0.8466	0.4348	6.25
Fringillidae	*Carduelis olivacea*	Palearctic	0	-0.7725	5.9256	3.58	0.7422	0.1930	17.65
Fringillidae	*Carduelis pinus*	Palearctic	0	0.3320	6.9113	3.04	0.5985	0.2140	82.49
Fringillidae	*Carduelis psaltria*	Neotropics	0	3.1630	6.1552	3.85	0.7539	0.3831	79.77
Fringillidae	*Carduelis siemiradzkii*	Neotropics	1	-0.2569	4.9294	3.85	0.7266	0.5595	19.23
Fringillidae	*Carduelis sinica*	Neotropics	0	0.2141	11.5758	2.47	0.6428	0.7499	39.27
Fringillidae	*Carduelis spinescens*	Palearctic	0	-0.9175	-	5.09	0.8640	0.1251	0.96
Fringillidae	*Carduelis spinoides*	Palearctic	0	-0.6181	-	4.36	0.8408	0.3042	10.72
Fringillidae	*Carduelis spinus*	Nearctic	0	-0.5335	-	5.53	0.8108	0.1660	2.75
Fringillidae	*Carduelis tristis*	Afrotropics	1	0.5155	4.5143	3.36	0.6221	0.2897	11.15
Fringillidae	*Carduelis uropygialis*	Nearctic	0	-0.4459	-	3.44	0.7318	0.2333	51.41
Fringillidae	*Carduelis xanthogastra*	Neotropics	0	-0.2065	-	3.46	0.8339	0.9196	64.92
Fringillidae	*Carduelis yarrellii*	IndoMalay	0	0.1914	5.0030	2.90	0.6631	0.6488	18.54
Fringillidae	*Carduelis yemenensis*	Neotropics	0	-0.0162	9.6212	3.72	0.7727	0.1985	29.31
Fringillidae	*Carpodacus cassinii*	Neotropics	0	-0.1522	10.7477	3.47	0.7363	0.2960	54.72
Fringillidae	*Carpodacus edwardsii*	IndoMalay	0	-0.1023	14.3720	3.85	0.7707	0.2453	21.58
Fringillidae	*Carpodacus eos*	Palearctic	0	-0.5361	-	4.57	0.8464	0.1279	0.12
Fringillidae	*Carpodacus erythrinus*	Nearctic	0	-0.6675	-	4.23	0.8405	0.3528	4.90
Fringillidae	*Carpodacus mexicanus*	Neotropics	0	-0.9315	8.4901	3.35	0.7198	0.0964	78.94
Fringillidae	*Carpodacus nipalensis*	Neotropics	0	-0.0511	12.5232	3.58	0.7488	0.4110	69.39
Fringillidae	*Carpodacus pulcherrimus*	Neotropics	1	0.0454	2.3855	3.61	0.7607	0.8433	24.92
Fringillidae	*Carpodacus puniceus*	Afrotropics	0	0.2600	7.4925	3.75	0.7211	0.2363	58.65
Fringillidae	*Carpodacus purpureus*	Neotropics	0	-0.3426	-	3.49	0.8321	0.4495	43.35
Fringillidae	*Carpodacus rhodochlamys*	Australasia	0	0.6980	5.7567	2.63	0.6523	0.0000	0.00
Fringillidae	*Carpodacus roborowskii*	IndoMalay	0	0.0874	6.6601	2.85	0.7639	0.6441	49.50
Fringillidae	*Carpodacus rodochroa*	IndoMalay	0	-0.0404	2.4140	2.97	0.7639	0.6695	52.28
Fringillidae	*Carpodacus rodopeplus*	IndoMalay	1	-0.1507	5.2146	2.86	0.6935	0.1564	48.90
Fringillidae	*Carpodacus roseus*	Nearctic	0	-0.7268	4.3284	4.11	0.7978	0.6748	40.89
Fringillidae	*Carpodacus rubescens*	Palearctic	0	-0.4655	6.9906	3.85	0.7702	0.4410	21.11
Fringillidae	*Carpodacus rubicilla*	Palearctic	0	-2.6093	5.6747	4.29	0.7802	0.6451	1.96
Fringillidae	*Carpodacus rubicilloides*	Palearctic	0	-0.7607	3.9426	4.76	0.8696	0.3046	2.30
Fringillidae	*Carpodacus synoicus*	Nearctic	0	-0.6711	-	4.10	0.8397	0.5240	2.36
Fringillidae	*Carpodacus thura*	Palearctic	0	-0.5187	-	3.78	0.7814	0.3619	2.12
Fringillidae	*Carpodacus trifasciatus*	Palearctic	0	-0.9429	6.2809	4.07	0.8084	0.3114	23.10
Fringillidae	*Carpodacus vinaceus*	Palearctic	0	-0.7544	10.8703	4.42	0.8197	0.4189	12.84
Fringillidae	*Coccothraustes abeillei*	Neotropics	0	-0.0035	3.0951	3.92	0.7696	0.3674	65.82
Fringillidae	*Coccothraustes coccothraustes*	Australasia	0	-0.0704	6.7698	2.80	0.7560	0.1599	22.60
Fringillidae	*Coccothraustes vespertinus*	Neotropics	1	-0.2858	6.8593	3.57	0.7228	0.5820	61.91
Fringillidae	*Eophona migratoria*	Nearctic	0	-0.6305	5.3687	4.26	0.8334	0.2368	12.11
Fringillidae	*Eophona personata*	Nearctic	0	-0.6908	12.1817	3.95	0.8124	0.5028	42.96
Fringillidae	*Eremopsaltria mongolicus*	Neotropics	0	0.1522	4.3689	3.14	0.7503	0.4712	57.95
Fringillidae	*Fringilla coelebs*	Afrotropics	0	0.0960	-	3.36	0.8044	0.6075	61.43
Fringillidae	*Fringilla montifringilla*	Afrotropics	0	-0.1466	7.2036	3.50	0.7851	0.4496	79.05
Fringillidae	*Haematospiza sipahi*	Australasia	0	0.0591	6.4144	2.48	0.6478	0.1611	2.45
Fringillidae	*Hemignathus munroi*	Neotropics	0	-0.0057	10.2695	3.86	0.7524	0.3724	82.52
Fringillidae	*Hemignathus virens*	Neotropics	0	-4.2353	10.5496	3.22	0.7179	0.2782	63.69
Fringillidae	*Himatione sanguinea*	Neotropics	0	-0.0949	-	3.88	0.7609	0.4068	29.69
Fringillidae	*Leucosticte arctoa*	Neotropics	0	0.0232	0.6167	3.85	0.7634	0.6560	47.60
Fringillidae	*Leucosticte atrata*	Neotropics	0	4.3725	6.0007	3.53	0.7281	0.2903	75.67
Fringillidae	*Leucosticte australis*	Neotropics	0	-0.0650	1.7037	4.29	0.7785	0.7048	86.60
Fringillidae	*Leucosticte brandti*	Neotropics	0	-0.0270	1.6484	3.90	0.7650	0.6532	39.19
Fringillidae	*Leucosticte nemoricola*	Neotropics	0	0.0718	7.3382	4.23	0.7495	0.5573	21.02
Fringillidae	*Leucosticte sillemi*	Neotropics	1	-0.0713	0.6357	4.13	0.7482	0.6410	54.54
Fringillidae	*Leucosticte tephrocotis*	Neotropics	0	-0.4391	-	2.98	0.8012	0.2405	86.66
Fringillidae	*Linurgus olivaceus*	IndoMalay	1	0.4539	0.4586	2.66	0.5788	0.0000	26.57
Fringillidae	*Loxia curvirostra*	Nearctic	0	-0.5011	4.6188	4.17	0.8317	0.3558	11.22
Fringillidae	*Loxia leucoptera*	Australasia	0	-0.5616	3.4105	3.27	0.8005	0.5097	89.87

Family	Species	Realm	Threat	Latitude	Elevation	Anomaly	Size	Shape	Orientation
Fringillidae	*Loxia megaplaga*	Australasia	0	-0.5368	-	3.12	0.7488	0.3156	77.19
Fringillidae	*Loxia pytyopsittacus*	Neotropics	0	-0.3265	15.6973	3.24	0.8021	-	-
Fringillidae	*Loxia pytyopsittacus*	Neotropics	0	-0.3265	15.6973	3.24	0.8021	0.2118	86.52
Fringillidae	*Loxia scotica*	Palearctic	0	-0.1966	13.2232	4.34	0.7680	0.1841	20.02
Fringillidae	*Loxioides bailleui*	Australasia	0	-0.0737	-	2.80	0.7435	-	-
Fringillidae	*Loxioides bailleui*	Australasia	0	-0.0737	-	2.80	0.7435	0.1494	23.97
Fringillidae	*Loxops coccineus*	Neotropics	1	-0.8777	5.1400	3.45	0.6221	0.5954	19.86
Fringillidae	*Mycerobas affinis*	Palearctic	0	1.5334	10.1284	4.40	0.7828	0.2562	0.51
Fringillidae	*Mycerobas carnipes*	Palearctic	0	-4.6401	4.8374	4.52	0.7348	0.1840	4.53
Fringillidae	*Mycerobas icterioides*	Palearctic	0	4.3044	10.5787	4.23	0.8062	0.5625	7.11
Fringillidae	*Mycerobas melanozanthos*	Palearctic	0	-0.9700	-	4.33	0.8344	0.2479	2.76
Fringillidae	*Oreomystis mana*	Neotropics	0	0.0335	10.4702	3.64	0.7718	0.4164	84.11
Fringillidae	*Pinicola enucleator*	Australasia	0	-0.1569	-	2.72	0.7631	0.2146	16.40
Fringillidae	*Pinicola subhimachala*	Australasia	0	0.2096	-	2.57	0.6662	0.2660	3.32
Fringillidae	*Psittirostra psittacea*	Neotropics	1	-0.1178	5.8632	3.65	0.7296	0.1205	54.09
Fringillidae	*Pyrrhoplectes epauletta*	IndoMalay	0	-0.1267	-	3.28	0.8217	0.7445	47.92
Fringillidae	*Pyrrhula aurantiaca*	IndoMalay	0	0.0241	-	3.35	0.7852	0.5356	39.83
Fringillidae	*Pyrrhula erythaca*	IndoMalay	0	-0.0623	6.3779	3.59	0.7548	0.3705	12.93
Fringillidae	*Pyrrhula erythrocephala*	Palearctic	0	-0.4677	-	3.71	0.7853	0.2914	1.28
Fringillidae	*Pyrrhula nipalensis*	Australasia	0	-0.1069	0.6212	2.76	0.7650	0.3129	26.20
Fringillidae	*Pyrrhula pyrrhula*	Australasia	0	-0.1069	6.5550	2.48	0.6302	0.2423	87.23
Fringillidae	*Rhodopechys obsoletus*	Afrotropics	0	-0.0652	-	3.67	0.8204	0.8963	56.83
Fringillidae	*Rhodopechys sanguineus*	Afrotropics	0	0.1111	-	3.31	0.7825	0.1528	2.95
Fringillidae	*Rhynchostruthus louisae*	Australasia	0	-0.1242	-	2.79	0.7739	0.4870	38.04
Fringillidae	*Rhynchostruthus percivali*	Neotropics	1	-0.5410	-	2.02	0.7700	0.6072	72.55
Fringillidae	*Rhynchostruthus socotranus*	Palearctic	0	-0.7041	5.2754	4.31	0.8611	0.1912	0.95
Fringillidae	*Serinus alario*	Afrotropics	0	-0.1231	13.9976	3.47	0.8378	0.6825	38.86
Fringillidae	*Serinus albogularis*	Afrotropics	0	-0.1088	9.3231	3.13	0.7428	0.2149	72.96
Fringillidae	*Serinus ankoberensis*	Afrotropics	0	-1.6376	-	3.49	0.7482	0.4256	43.27
Fringillidae	*Serinus atrogularis*	Afrotropics	0	0.0638	6.8576	3.32	0.8260	0.3959	18.22
Fringillidae	*Serinus buchanani*	Afrotropics	0	-0.2023	12.7126	3.55	0.8266	0.7198	72.75
Fringillidae	*Serinus burtoni*	Afrotropics	1	-0.1171	4.8749	3.18	0.6631	0.1961	68.87
Fringillidae	*Serinus canicollis*	IndoMalay	0	0.0101	-	2.84	0.7871	0.5961	30.93
Fringillidae	*Serinus capistratus*	Afrotropics	0	0.0436	-	3.33	0.8092	0.6327	4.67
Fringillidae	*Serinus citrinelloides*	IndoMalay	0	-0.0934	10.2808	3.19	0.7966	0.4790	35.12
Fringillidae	*Serinus citrinipectus*	Neotropics	0	-0.1736	-	3.54	0.8351	0.7812	72.37
Fringillidae	*Serinus donaldsoni*	IndoMalay	0	-0.3579	6.6249	3.56	0.8459	-	-
Fringillidae	*Serinus donaldsoni*	IndoMalay	0	-0.3579	6.6249	3.56	0.8459	0.2771	27.12
Fringillidae	*Serinus dorsostriatus*	IndoMalay	0	-0.3151	7.4098	3.30	0.8179	0.4933	4.55
Fringillidae	*Serinus estherae*	Australasia	0	-0.1790	4.5518	2.56	0.6523	0.1875	30.24
Fringillidae	*Serinus flavigula*	Palearctic	1	2.4241	5.8974	4.35	0.7422	0.2564	10.48
Fringillidae	*Serinus flaviventris*	IndoMalay	0	-0.1921	2.1472	3.26	0.7637	0.3153	28.49
Fringillidae	*Serinus flavivertex*	IndoMalay	0	-0.1257	-	3.41	0.7846	0.2665	18.68
Fringillidae	*Serinus frontalis*	IndoMalay	1	-0.1828	-	3.90	0.7689	0.7932	10.21
Fringillidae	*Serinus gularis*	Palearctic	0	-0.6510	6.3754	4.63	0.8474	0.2647	4.00
Fringillidae	*Serinus hypostictus*	Palearctic	0	-0.4984	9.3535	4.34	0.8783	0.4548	23.78
Fringillidae	*Serinus koliensis*	IndoMalay	0	-0.0941	4.9004	3.51	0.8152	0.7702	55.26
Fringillidae	*Serinus leucopterus*	Neotropics	0	-0.2578	11.7510	3.76	0.8189	0.1966	45.52
Fringillidae	*Serinus leucopygius*	Nearctic	0	-0.7372	9.0877	4.32	0.8355	0.4074	27.44
Fringillidae	*Serinus melanochrous*	Nearctic	0	-0.9001	10.9718	4.17	0.8388	0.3478	35.33
Fringillidae	*Serinus mennelli*	Neotropics	0	-0.7078	6.0128	3.28	0.7128	0.1031	81.14
Fringillidae	*Serinus mozambicus*	Neotropics	0	-0.7966	5.5717	2.37	0.7444	0.2545	82.33
Fringillidae	*Serinus nigriceps*	Australasia	0	-0.4290	2.2570	3.01	0.6562	0.1808	64.24
Fringillidae	*Serinus pusillus*	Afrotropics	0	-0.0635	-	3.16	0.7554	0.4258	70.32
Fringillidae	*Serinus reichardi*	Neotropics	0	0.2837	-	4.11	0.8110	0.8023	3.66
Fringillidae	*Serinus rothschildi*	Neotropics	0	-0.0434	4.3276	3.93	0.8414	0.5325	30.41
Fringillidae	*Serinus scotops*	Neotropics	0	-0.2734	-	3.37	0.7971	0.5002	38.99
Fringillidae	*Serinus serinus*	Neotropics	0	0.1082	8.4161	3.60	0.7537	0.3859	81.57
Fringillidae	*Serinus striolatus*	Neotropics	0	-0.1122	-	3.89	0.8351	0.6015	42.72
Fringillidae	*Serinus sulphuratus*	Neotropics	0	-0.2693	-	3.71	0.8009	0.8345	57.44
Fringillidae	*Serinus symonsi*	Neotropics	0	6.9887	8.4447	4.11	0.7030	0.1713	20.31
Fringillidae	*Serinus syriacus*	Neotropics	0	0.3984	5.9269	4.28	0.7409	0.7202	72.81
Fringillidae	*Serinus thibetanus*	Neotropics	0	0.0961	0.6791	4.23	0.7586	0.6768	27.00
Fringillidae	*Serinus totta*	Neotropics	0	-0.0330	4.0820	4.02	0.7925	0.3399	23.61
Fringillidae	*Serinus tristriatus*	Neotropics	0	-0.0529	1.2092	4.22	0.7628	0.3122	28.17
Fringillidae	*Serinus whytii*	Neotropics	0	-0.5087	7.6076	4.30	0.6948	0.4318	4.75
Fringillidae	*Serinus xantholaemus*	Neotropics	0	0.0742	1.3237	3.96	0.7659	0.7557	12.71
Fringillidae	*Serinus xanthopygius*	Afrotropics	0	-0.0843	-	3.50	0.8174	0.7581	35.73
Fringillidae	*Uragus sibiricus*	Neotropics	0	-0.0676	-	3.75	0.7778	0.2203	35.84
Fringillidae	*Vestiaria coccinea*	Palearctic	0	-0.8728	-	5.31	0.8518	0.1380	2.84
Furnariidae	*Acrobatornis fonsecai*	Neotropics	1	-0.1236	1.8146	3.13	0.6302	0.5888	90.00
Furnariidae	*Anabacerthia amaurotis*	Neotropics	0	-0.4602	6.1774	2.91	0.7632	0.3182	26.37
Furnariidae	*Anabacerthia striaticollis*	Neotropics	0	0.0127	8.8928	3.67	0.7687	0.3836	86.16
Furnariidae	*Anabacerthia variegaticeps*	Neotropics	0	0.0633	9.7407	3.36	0.7554	0.2565	45.85

Family	Species	Realm	Threat	Latitude	Elevation	Anomaly	Size	Shape	Orientation
Furnariidae	*Anabazenops dorsalis*	Neotropics	0	-6.5547	4.8220	3.90	0.7716	0.2951	63.37
Furnariidae	*Anabazenops fuscus*	Neotropics	0	-0.3119	4.8126	3.14	0.7533	0.2894	50.12
Furnariidae	*Ancistrops strigilatus*	Neotropics	0	0.0993	-	4.08	0.8092	0.5519	8.98
Furnariidae	*Anumbius annumbi*	Neotropics	0	-0.5082	-	2.94	0.8130	0.4114	42.34
Furnariidae	*Aphrastura spinicauda*	Neotropics	0	-0.5289	6.2408	2.10	0.7602	0.2316	88.43
Furnariidae	*Asthenes anthoides*	Afrotropics	0	-0.0811	5.3802	3.18	0.7614	0.3874	70.15
Furnariidae	*Asthenes baeri*	Palearctic	0	-0.8062	7.6847	4.41	0.8745	0.0867	0.92
Furnariidae	*Asthenes berlepschi*	Neotropics	0	-0.1941	-	3.58	0.8186	0.2342	41.98
Furnariidae	*Asthenes coryi*	Neotropics	0	0.0061	9.6037	3.61	0.7228	0.2943	18.97
Furnariidae	*Asthenes dorbignyi*	Neotropics	0	-0.5170	4.6196	2.03	0.7389	0.4805	88.35
Furnariidae	*Asthenes flammulata*	Neotropics	0	-0.5634	3.1049	2.80	0.7977	0.6015	85.59
Furnariidae	*Asthenes fuliginosa*	Neotropics	0	0.0000	8.6312	4.06	0.5788	0.0000	90.00
Furnariidae	*Asthenes griseomurina*	Neotropics	0	0.7218	7.8590	4.10	0.6631	0.3162	45.35
Furnariidae	*Asthenes harterti*	Neotropics	0	4.1040	17.0760	4.03	0.7542	0.3146	47.64
Furnariidae	*Asthenes helleri*	Neotropics	0	0.3416	10.0500	3.48	0.7382	0.3021	78.91
Furnariidae	*Asthenes heterura*	Neotropics	0	2.7815	9.8735	3.41	0.7151	0.2624	64.64
Furnariidae	*Asthenes hudsoni*	Neotropics	0	0.1009	10.7286	3.34	0.6716	0.3261	84.75
Furnariidae	*Asthenes humilis*	Neotropics	0	14.8187	3.2016	4.01	0.6876	0.2010	31.34
Furnariidae	*Asthenes luizae*	Neotropics	0	-40.0932	4.9831	4.12	0.7008	0.2616	28.44
Furnariidae	*Asthenes maculicauda*	Neotropics	0	3.9781	10.2086	3.96	0.7185	-	-
Furnariidae	*Asthenes maculicauda*	Neotropics	0	3.9781	10.2086	3.96	0.7185	0.3684	81.18
Furnariidae	*Asthenes modesta*	Neotropics	0	-0.5059	1.6628	2.33	0.7519	0.7805	65.87
Furnariidae	*Asthenes moreirae*	Neotropics	0	-3.3688	12.1979	3.95	0.7348	0.2008	36.24
Furnariidae	*Asthenes ottonis*	Neotropics	0	-0.3288	5.6736	3.59	0.6690	0.3318	87.81
Furnariidae	*Asthenes palpebralis*	Neotropics	0	36.8885	12.1891	4.04	0.6824	0.1596	32.62
Furnariidae	*Asthenes perijana*	Neotropics	0	-0.2952	-	3.26	0.8041	0.2340	87.56
Furnariidae	*Asthenes pudibunda*	Neotropics	0	-0.5843	5.7138	3.02	0.6859	0.3296	36.53
Furnariidae	*Asthenes pyrrholeuca*	Neotropics	0	-18.7121	7.5705	4.13	0.6876	0.3152	13.05
Furnariidae	*Asthenes sclateri*	Neotropics	0	0.0000	4.2083	4.01	0.6120	0.9804	90.00
Furnariidae	*Asthenes vilcabambae*	Neotropics	0	-0.8254	7.6550	3.98	0.7251	0.1916	51.35
Furnariidae	*Asthenes virgata*	Neotropics	0	-0.6842	9.5894	2.47	0.7846	0.4545	74.96
Furnariidae	*Asthenes wyatti*	Neotropics	0	7.8341	12.8648	3.97	0.7488	0.2640	82.44
Furnariidae	*Automolus infuscatus*	Neotropics	1	0.0000	2.1671	3.70	0.5985	0.5771	46.13
Furnariidae	*Automolus leucophthalmus*	Neotropics	0	-0.0437	11.7575	3.71	0.7939	0.2660	53.14
Furnariidae	*Automolus melanopezus*	Neotropics	0	-0.0969	9.2724	3.82	0.7151	0.2968	3.71
Furnariidae	*Automolus ochrolaemus*	Nearctic	0	-0.5164	-	4.01	0.7995	0.5210	25.58
Furnariidae	*Automolus rubiginosus*	Neotropics	0	0.0681	2.3030	4.04	0.8325	0.6466	0.78
Furnariidae	*Automolus rufipectus*	Neotropics	0	-0.2251	-	3.31	0.8002	0.4103	43.08
Furnariidae	*Automolus rufipileatus*	Neotropics	0	0.0552	1.7030	4.04	0.7722	0.4066	38.68
Furnariidae	*Berlepschia rikeri*	IndoMalay	0	0.2993	-	2.91	0.6961	0.2435	89.56
Furnariidae	*Certhiaxis cinnamomeus*	Afrotropics	0	-0.4110	9.1138	3.95	0.8115	0.3364	2.94
Furnariidae	*Certhiaxis mustelinus*	Afrotropics	0	-0.3400	-	3.75	0.7857	0.3417	54.36
Furnariidae	*Cichlocolaptes leucophrus*	IndoMalay	0	-0.0786	-	3.08	0.8153	0.5230	22.12
Furnariidae	*Cinclodes albidiventris*	Afrotropics	0	0.0732	-	3.26	0.7945	0.9005	24.49
Furnariidae	*Cinclodes albiventris*	Afrotropics	0	-0.0376	-	3.43	0.7735	0.3031	79.54
Furnariidae	*Cinclodes antarcticus*	Neotropics	0	-0.2349	-	2.92	0.7482	0.2532	42.45
Furnariidae	*Cinclodes aricomae*	Neotropics	0	-0.0862	4.7650	3.55	0.7385	0.2740	37.89
Furnariidae	*Cinclodes atacamensis*	Australasia	0	-0.1297	-	2.75	0.7352	0.1885	22.55
Furnariidae	*Cinclodes comechingonus*	Australasia	0	-0.0872	-	2.77	0.7656	0.2555	23.34
Furnariidae	*Cinclodes fuscus*	Afrotropics	0	0.0283	-	3.65	0.8354	0.2085	1.31
Furnariidae	*Cinclodes nigrofumosus*	Palearctic	1	-0.9118	-	4.37	0.7628	0.3708	8.66
Furnariidae	*Cinclodes olrogi*	Palearctic	0	-0.5488	8.7780	4.04	0.8379	0.9386	36.24
Furnariidae	*Cinclodes oustaleti*	Afrotropics	0	-0.1471	5.4611	3.45	0.8631	0.3937	9.98
Furnariidae	*Cinclodes pabsti*	Neotropics	0	-0.2767	-	3.47	0.8296	0.3608	86.44
Furnariidae	*Cinclodes palliatus*	Palearctic	0	-0.8001	-	4.49	0.8637	0.4454	11.52
Furnariidae	*Cinclodes patagonicus*	IndoMalay	1	0.0575	-	2.84	0.7845	0.4231	1.71
Furnariidae	*Cinclodes taczanowskii*	IndoMalay	0	-0.0181	-	2.75	0.7356	0.1672	40.87
Furnariidae	*Clibanornis dendrocolaptoides*	Afrotropics	0	-0.1565	14.4953	3.68	0.8020	0.5558	87.92
Furnariidae	*Coryphistera alaudina*	Palearctic	0	-0.6454	-	4.32	0.8587	0.2523	1.77
Furnariidae	*Cranioleuca albicapilla*	Palearctic	0	-0.4680	-	4.34	0.8673	0.6518	18.39
Furnariidae	*Cranioleuca albiceps*	Afrotropics	0	-0.0708	8.2957	3.60	0.8461	0.7814	76.06
Furnariidae	*Cranioleuca antisiensis*	Palearctic	0	-1.0145	-	4.34	0.8175	0.1634	2.86
Furnariidae	*Cranioleuca curtata*	Australasia	0	-0.5905	-	3.34	0.8333	0.4518	4.48
Furnariidae	*Cranioleuca demissa*	Australasia	0	-0.3952	-	3.29	0.8324	0.7178	28.58
Furnariidae	*Cranioleuca erythrops*	Afrotropics	0	-0.0882	7.0481	3.17	0.7505	0.3267	71.56
Furnariidae	*Cranioleuca gutturata*	Afrotropics	0	-0.0416	2.4606	3.17	0.7291	0.2413	64.53
Furnariidae	*Cranioleuca hellmayri*	Afrotropics	0	-0.0770	2.9766	3.16	0.7560	0.3861	70.19
Furnariidae	*Cranioleuca henricae*	Afrotropics	0	-0.3546	0.8022	3.19	0.6876	0.4450	55.15
Furnariidae	*Cranioleuca marcapatae*	Afrotropics	0	-0.3127	2.5573	3.24	0.7228	0.2616	84.20
Furnariidae	*Cranioleuca muelleri*	Afrotropics	0	-0.0980	10.0267	3.17	0.7490	0.3231	71.02
Furnariidae	*Cranioleuca obsoleta*	Afrotropics	0	-0.2348	2.7213	3.22	0.7318	0.2772	79.22
Furnariidae	*Cranioleuca pallida*	Afrotropics	0	-0.3175	4.2644	3.00	0.7040	0.2983	79.45
Furnariidae	*Cranioleuca pyrrhophia*	Afrotropics	0	0.0115	0.4247	3.11	0.6740	0.4451	17.36
Furnariidae	*Cranioleuca semicinerea*	Australasia	0	-0.1033	-	2.77	0.7669	0.3168	23.51

Family	Species	Realm	Threat	Latitude	Elevation	Anomaly	Size	Shape	Orientation
Furnariidae	*Cranioleuca subcristata*	Australasia	0	-0.0229	-	2.88	0.7348	0.6451	88.43
Furnariidae	*Cranioleuca sulphurifera*	Australasia	0	-0.5235	-	3.38	0.8338	0.5448	4.32
Furnariidae	*Cranioleuca vulpecula*	Australasia	0	-0.2271	-	2.92	0.7875	0.7479	52.84
Furnariidae	*Cranioleuca vulpina*	Australasia	0	-0.5723	-	3.30	0.8336	0.5330	6.15
Furnariidae	*Furnarius cristatus*	Afrotropics	0	-0.0975	-	3.56	0.8209	0.3749	61.94
Furnariidae	*Furnarius figulus*	Afrotropics	0	-0.1124	12.9320	3.59	0.7959	0.4028	80.70
Furnariidae	*Furnarius leucopus*	Afrotropics	0	0.0949	11.4761	3.32	0.8141	0.4891	0.48
Furnariidae	*Furnarius minor*	Afrotropics	0	12.4061	6.7534	3.36	0.7435	0.5097	11.52
Furnariidae	*Furnarius rufus*	Afrotropics	0	-0.2224	-	4.01	0.7971	0.8560	18.22
Furnariidae	*Furnarius torridus*	Afrotropics	1	0.7324	3.1637	3.64	0.6784	0.2880	63.61
Furnariidae	*Geocerthia serrana*	IndoMalay	0	-0.0293	4.7983	2.83	0.6842	0.3249	74.52
Furnariidae	*Geositta antarctica*	IndoMalay	0	-0.1203	-	3.80	0.7576	0.1272	17.36
Furnariidae	*Geositta crassirostris*	IndoMalay	0	-0.5957	-	3.29	0.8053	0.4566	16.91
Furnariidae	*Geositta cunicularia*	IndoMalay	0	0.0187	-	3.42	0.7779	0.3680	18.17
Furnariidae	*Geositta isabellina*	IndoMalay	0	-0.1185	6.7733	3.15	0.7432	0.7373	86.59
Furnariidae	*Geositta maritima*	IndoMalay	0	0.1622	11.5856	3.97	0.7560	0.1526	8.15
Furnariidae	*Geositta peruviana*	IndoMalay	0	0.0781	-	3.64	0.7604	0.2364	17.22
Furnariidae	*Geositta poeciloptera*	Palearctic	1	-18.4340	8.3505	3.75	0.6824	0.6654	25.11
Furnariidae	*Geositta punensis*	IndoMalay	0	-0.8768	3.1195	2.61	0.6562	0.4622	64.29
Furnariidae	*Geositta rufipennis*	IndoMalay	0	-0.2062	13.0009	4.31	0.7531	0.1612	29.22
Furnariidae	*Geositta saxicolina*	IndoMalay	0	-0.1155	-	2.85	0.7008	0.3433	73.37
Furnariidae	*Geositta tenuirostris*	IndoMalay	0	-0.3242	7.8969	3.14	0.7185	0.3779	70.38
Furnariidae	*Gyalophylax hellmayri*	Neotropics	0	0.1563	13.3697	3.85	0.7474	0.3238	84.69
Furnariidae	*Heliobletus contaminatus*	Neotropics	0	0.0000	7.6979	3.04	0.6370	0.3678	56.34
Furnariidae	*Hellmayrea gularis*	Australasia	0	-10.4668	-	2.74	0.6876	0.4986	76.15
Furnariidae	*Hylocryptus erythrocephalus*	Palearctic	0	-0.6612	15.6487	3.99	0.8467	0.1869	1.10
Furnariidae	*Hylocryptus rectirostris*	Palearctic	0	-0.5155	8.1923	4.32	0.8880	0.1280	0.65
Furnariidae	*Hyloctistes subulatus*	Afrotropics	0	-0.1351	6.2796	3.53	0.8446	0.4435	42.76
Furnariidae	*Leptasthenura aegithaloides*	Palearctic	0	-0.7386	0.0000	4.54	0.8016	0.1487	0.97
Furnariidae	*Leptasthenura andicola*	Nearctic	0	-0.9208	10.6907	4.21	0.8046	0.8499	34.39
Furnariidae	*Leptasthenura fuliginiceps*	Palearctic	0	-0.7822	3.7171	4.91	0.8629	0.0611	0.32
Furnariidae	*Leptasthenura pileata*	Afrotropics	0	-0.2854	-	3.46	0.8381	0.4111	3.93
Furnariidae	*Leptasthenura platensis*	Nearctic	0	-0.8491	0.4329	4.22	0.8315	0.2736	2.87
Furnariidae	*Leptasthenura setaria*	Neotropics	0	-0.3516	-	2.60	0.8108	0.1163	0.39
Furnariidae	*Leptasthenura striata*	Palearctic	0	-0.5406	0.0000	4.10	0.7753	0.3057	11.17
Furnariidae	*Leptasthenura striolata*	Palearctic	0	-0.5955	-	4.37	0.8021	0.3362	10.60
Furnariidae	*Leptasthenura xenothorax*	Nearctic	0	-0.8679	-	3.63	0.7765	0.0380	0.05
Furnariidae	*Leptasthenura yanacensis*	Afrotropics	0	0.1583	-	2.85	0.7060	0.1042	64.19
Furnariidae	*Limnoctites rectirostris*	Palearctic	0	-0.9597	11.6293	4.39	0.7820	0.2580	3.99
Furnariidae	*Limnornis curvirostris*	Neotropics	0	-0.6760	-	2.34	0.7892	0.4409	82.55
Furnariidae	*Lochmias nematura*	Palearctic	0	2.8027	12.3438	4.44	0.8225	0.7900	0.03
Furnariidae	*Margarornis bellulus*	Australasia	1	-0.9514	4.2613	2.49	0.5788	0.0000	0.00
Furnariidae	*Margarornis rubiginosus*	IndoMalay	0	-0.0446	-	2.94	0.8050	0.4343	50.26
Furnariidae	*Margarornis squamiger*	IndoMalay	0	0.3086	-	2.52	0.5985	0.0024	45.41
Furnariidae	*Margarornis stellatus*	IndoMalay	0	0.0389	1.9527	2.84	0.7876	0.4558	3.58
Furnariidae	*Megaxenops parnaguae*	IndoMalay	0	-0.0757	-	3.01	0.8140	0.3874	59.88
Furnariidae	*Metopothrix aurantiaca*	Australasia	0	-0.2237	-	2.17	0.6804	0.6686	14.77
Furnariidae	*Ochetorhynchus andaecola*	IndoMalay	1	-0.4698	5.2796	2.71	0.6935	0.1207	11.58
Furnariidae	*Ochetorhynchus melanura*	IndoMalay	0	-0.0851	4.2863	3.11	0.8247	0.3999	31.35
Furnariidae	*Ochetorhynchus phoenicurus*	Australasia	1	-0.5392	5.4378	2.63	0.6523	0.0000	0.00
Furnariidae	*Ochetorhynchus ruficaudus*	Australasia	0	-0.0204	7.9373	2.71	0.7158	0.4968	56.93
Furnariidae	*Phacellodomus dorsalis*	Neotropics	1	-0.2656	9.6688	3.38	0.6876	0.3548	85.12
Furnariidae	*Phacellodomus erythrophthalmus*	Neotropics	0	13.6347	6.0143	3.88	0.6891	0.1737	83.17
Furnariidae	*Phacellodomus ferrugineigula*	Neotropics	0	0.0322	5.9617	4.07	0.8278	0.8612	70.41
Furnariidae	*Phacellodomus maculipectus*	Neotropics	1	-0.0457	4.7777	3.63	0.7684	0.5808	51.87
Furnariidae	*Phacellodomus ruber*	Neotropics	0	0.0262	2.5672	4.06	0.7868	0.6185	34.29
Furnariidae	*Phacellodomus rufifrons*	Neotropics	0	0.0069	9.4658	3.75	0.7663	0.3482	81.43
Furnariidae	*Phacellodomus sibilatrix*	Neotropics	0	-0.5424	-	2.87	0.7851	0.5656	16.00
Furnariidae	*Phacellodomus striaticeps*	Neotropics	1	-0.0271	-	4.08	0.7367	0.5923	40.07
Furnariidae	*Phacellodomus striaticollis*	Neotropics	1	-0.0210	8.9745	3.01	0.7172	0.1850	69.95
Furnariidae	*Philydor atricapillus*	Neotropics	0	-0.1607	6.8578	3.91	0.8492	0.6880	32.51
Furnariidae	*Philydor erythrocercum*	Neotropics	0	0.0704	2.8180	3.83	0.8100	0.4211	51.07
Furnariidae	*Philydor erythropterum*	Neotropics	0	-0.2007	3.0072	3.84	0.8227	0.5118	66.27
Furnariidae	*Philydor fuscipenne*	Neotropics	0	-0.1018	0.0000	4.16	0.7331	0.2788	66.31
Furnariidae	*Philydor lichtensteini*	Neotropics	0	-0.0661	3.6911	3.65	0.7618	0.5482	26.24
Furnariidae	*Philydor novaesi*	Neotropics	0	0.0364	4.3303	3.76	0.7777	0.4498	79.14
Furnariidae	*Philydor pyrrhodes*	Neotropics	0	-0.1988	-	4.11	0.7684	0.4633	13.03
Furnariidae	*Philydor ruficaudatum*	Neotropics	0	0.0646	-	3.95	0.8105	0.3475	11.71
Furnariidae	*Philydor rufum*	Neotropics	0	-0.3133	9.1556	3.07	0.7804	0.3511	29.35
Furnariidae	*Phleocryptes melanops*	Neotropics	0	-9.4874	4.3463	4.03	0.7286	0.2272	52.02
Furnariidae	*Premnoplex brunnescens*	Afrotropics	0	-0.1414	3.9993	3.35	0.7866	0.2487	70.00
Furnariidae	*Premnoplex tatei*	Afrotropics	0	-3.7112	15.8549	3.54	0.7389	0.7327	86.03
Furnariidae	*Pseudasthenes cactorum*	IndoMalay	0	-0.1757	0.0000	4.05	0.7500	0.4316	41.06

Family	Species	Realm	Threat	Latitude	Elevation	Anomaly	Size	Shape	Orientation
Furnariidae	*Pseudasthenes humicola*	IndoMalay	1	-0.1977	3.6651	3.70	0.6974	0.1377	12.30
Furnariidae	*Pseudasthenes patagonica*	Palearctic	0	-0.2471	11.1018	3.51	0.8148	0.2107	3.64
Furnariidae	*Pseudasthenes steinbachi*	IndoMalay	0	0.0189	-	2.82	0.7607	0.1865	41.04
Furnariidae	*Pseudocolaptes boissonneautii*	Afrotropics	0	-0.2646	-	3.44	0.7651	0.4778	4.07
Furnariidae	*Pseudocolaptes lawrencii*	IndoMalay	0	-0.1303	-	3.05	0.7783	0.2596	68.26
Furnariidae	*Pseudocolaptes lawrencii*	IndoMalay	0	-0.1303	-	3.05	0.7783	-	-
Furnariidae	*Pseudoseisura cristata*	Australasia	0	-0.2107	-	2.71	0.7185	0.5623	60.71
Furnariidae	*Pseudoseisura gutturalis*	IndoMalay	0	0.0191	-	2.84	0.7895	0.5189	10.46
Furnariidae	*Pseudoseisura lophotes*	IndoMalay	0	-0.0237	-	2.64	0.7261	0.2111	67.49
Furnariidae	*Pseudoseisura unirufa*	IndoMalay	0	-0.0168	-	2.82	0.7913	0.5760	7.08
Furnariidae	*Pygarrhichas albogularis*	Neotropics	1	-0.7696	2.2305	2.51	0.6562	0.0848	71.87
Furnariidae	*Roraimia adusta*	Neotropics	0	-0.1263	-	3.34	0.7602	0.8100	32.99
Furnariidae	*Schoeniophylax phryganophilus*	Australasia	0	-0.2961	-	3.19	0.7552	0.2901	18.38
Furnariidae	*Sclerurus albigularis*	IndoMalay	0	-0.0391	-	2.62	0.7019	0.6235	86.79
Furnariidae	*Sclerurus caudacutus*	Australasia	0	-0.5465	4.4699	2.49	0.5788	0.0000	0.00
Furnariidae	*Sclerurus mexicanus*	Australasia	0	-0.1244	-	2.72	0.7096	0.5293	48.75
Furnariidae	*Sclerurus rufigularis*	Australasia	0	-0.0887	-	2.78	0.7683	0.2599	22.86
Furnariidae	*Sclerurus scansor*	Australasia	0	-0.6160	-	2.52	0.6523	0.2952	15.25
Furnariidae	*Simoxenops striatus*	Neotropics	0	0.2073	5.6128	2.94	0.6842	0.2269	68.70
Furnariidae	*Simoxenops ucayalae*	Neotropics	0	-4.4089	4.5321	3.66	0.6740	0.4599	70.39
Furnariidae	*Siptornis striaticollis*	Neotropics	1	0.0000	1.2283	2.52	0.5788	0.0000	90.00
Furnariidae	*Siptornopsis hypochondriaca*	Neotropics	0	0.0639	9.6191	3.32	0.6120	0.9804	90.00
Furnariidae	*Spartonoica maluroides*	Palearctic	0	-0.2161	4.5414	3.32	0.7272	0.3288	10.75
Furnariidae	*Sylviorthorhynchus desmursii*	IndoMalay	0	0.0217	-	2.63	0.6662	0.2692	43.93
Furnariidae	*Synallaxis albescens*	IndoMalay	0	-0.1164	4.1091	2.63	0.6935	0.8844	54.39
Furnariidae	*Synallaxis albigularis*	IndoMalay	0	0.0294	-	2.83	0.7725	0.4557	4.89
Furnariidae	*Synallaxis albilora*	IndoMalay	0	-0.0401	4.2505	2.54	0.6120	0.5391	75.64
Furnariidae	*Synallaxis azarae*	IndoMalay	0	0.0466	10.9232	4.03	0.7371	0.1381	24.81
Furnariidae	*Synallaxis beverlyae*	Palearctic	0	-0.4493	-	3.34	0.8072	0.5380	8.94
Furnariidae	*Synallaxis brachyura*	IndoMalay	0	-0.0390	8.3850	2.94	0.8007	0.4503	52.63
Furnariidae	*Synallaxis cabanisi*	IndoMalay	0	-0.0724	3.4998	2.69	0.6631	0.2160	80.96
Furnariidae	*Synallaxis candei*	IndoMalay	0	-0.1243	-	3.02	0.7737	0.3889	83.69
Furnariidae	*Synallaxis castanea*	IndoMalay	0	-0.4818	-	2.71	0.7019	0.1163	11.45
Furnariidae	*Synallaxis cherriei*	IndoMalay	0	-0.0176	9.6438	2.66	0.6961	0.5140	78.75
Furnariidae	*Synallaxis cinerascens*	Afrotropics	0	-6.8728	9.1696	3.73	0.7944	0.5910	13.28
Furnariidae	*Synallaxis cinnamomea*	Afrotropics	0	-0.1961	-	3.29	0.7811	0.2183	73.88
Furnariidae	*Synallaxis courseni*	Afrotropics	0	-0.1414	-	3.25	0.7314	0.2353	73.80
Furnariidae	*Synallaxis erythrothorax*	Afrotropics	0	-0.3843	10.0492	3.59	0.7702	0.7138	79.56
Furnariidae	*Synallaxis frontalis*	Australasia	0	-0.5533	-	2.62	0.7416	0.7331	10.48
Furnariidae	*Synallaxis fuscorufa*	Australasia	0	-0.6377	-	3.15	0.7922	0.6428	41.95
Furnariidae	*Synallaxis gujanensis*	Australasia	0	-0.8582	-	2.41	0.7040	0.3629	3.55
Furnariidae	*Synallaxis hypospodia*	Neotropics	1	-0.3662	-	2.53	0.6974	0.1632	17.43
Furnariidae	*Synallaxis infuscata*	Neotropics	0	0.0567	10.6232	3.79	0.7927	0.7030	68.01
Furnariidae	*Synallaxis kollari*	Nearctic	0	-0.8649	9.7274	4.15	0.8222	0.4029	7.14
Furnariidae	*Synallaxis macconnelli*	Neotropics	0	-0.2358	9.3006	3.77	0.8545	0.6272	43.13
Furnariidae	*Synallaxis maranonica*	Nearctic	0	-0.5225	7.8662	4.09	0.8469	0.6135	15.16
Furnariidae	*Synallaxis moesta*	Nearctic	0	-0.7016	16.0232	3.85	0.7918	0.7469	70.41
Furnariidae	*Synallaxis propinqua*	Afrotropics	0	-0.4816	-	3.77	0.7853	0.6014	27.33
Furnariidae	*Synallaxis ruficapilla*	Afrotropics	0	-0.0817	-	3.37	0.8362	0.5645	30.28
Furnariidae	*Synallaxis rutilans*	Neotropics	0	-0.5163	-	2.72	0.7850	0.3303	38.25
Furnariidae	*Synallaxis scutata*	Neotropics	0	-0.5422	3.5256	3.10	0.7855	0.4935	31.26
Furnariidae	*Synallaxis spixi*	Nearctic	0	-0.7542	-	5.78	0.8376	0.0454	0.03
Furnariidae	*Synallaxis stictothorax*	Palearctic	0	-0.6579	1.4128	5.72	0.8381	0.0480	0.10
Furnariidae	*Synallaxis subpudica*	Palearctic	0	-0.6972	0.4954	6.50	0.8174	0.0289	0.46
Furnariidae	*Synallaxis tithys*	IndoMalay	0	-0.1334	2.2201	3.30	0.8202	0.4131	12.51
Furnariidae	*Synallaxis unirufa*	Palearctic	0	-0.4772	4.7559	4.15	0.8544	0.3916	4.61
Furnariidae	*Synallaxis whitneyi*	Australasia	1	-0.5060	2.3415	2.19	0.7040	0.2392	42.02
Furnariidae	*Synallaxis zimmeri*	Palearctic	0	-0.2912	-	4.11	0.7535	0.0253	0.84
Furnariidae	*Syndactyla dimidiata*	IndoMalay	0	-0.0496	1.8711	3.42	0.8282	0.3084	16.70
Furnariidae	*Syndactyla guttulata*	Afrotropics	0	-0.3208	-	3.13	0.7416	0.2323	71.05
Furnariidae	*Syndactyla roraimae*	Australasia	0	-0.2788	-	2.87	0.8107	0.4259	6.47
Furnariidae	*Syndactyla ruficollis*	Palearctic	0	-0.4612	-	3.66	0.8406	0.3277	17.55
Furnariidae	*Syndactyla rufosuperciliata*	Nearctic	0	-0.8301	8.0999	4.30	0.8030	0.6031	6.09
Furnariidae	*Syndactyla subalaris*	Palearctic	0	-1.0148	9.8317	4.56	0.8758	0.1280	0.16
Furnariidae	*Tarphonomus certhioides*	Palearctic	0	-0.3502	-	3.63	0.8048	0.3004	4.99
Furnariidae	*Tarphonomus harterti*	Palearctic	0	-0.2104	-	3.26	0.5985	0.0000	0.00
Furnariidae	*Thripadectes flammulatus*	Neotropics	0	-1.0102	6.7426	3.80	0.6428	0.1991	9.06
Furnariidae	*Thripadectes holostictus*	Neotropics	0	0.5883	8.2090	2.97	0.6824	0.1306	64.46
Furnariidae	*Thripadectes ignobilis*	Neotropics	0	0.2127	-	4.02	0.8121	0.8437	20.07
Furnariidae	*Thripadectes melanorhynchus*	Neotropics	0	-0.2310	-	2.98	0.7711	0.2968	31.81
Furnariidae	*Thripadectes rufobrunneus*	Neotropics	0	0.1314	-	4.27	0.7884	0.4278	49.70
Furnariidae	*Thripadectes scrutator*	Neotropics	0	0.0533	8.1769	3.64	0.7647	0.4124	79.24
Furnariidae	*Thripadectes virgaticeps*	Neotropics	0	-0.0620	1.4259	4.00	0.8288	0.6585	10.83
Furnariidae	*Thripophaga berlepschi*	Neotropics	0	0.2037	-	3.98	0.8121	0.6829	71.39

Family	Species	Realm	Threat	Latitude	Elevation	Anomaly	Size	Shape	Orientation
Furnariidae	*Thripophaga cherriei*	Australasia	1	-1.0070	4.8768	2.49	0.5788	0.0000	0.00
Furnariidae	*Thripophaga fusciceps*	IndoMalay	0	-0.0277	2.5312	2.63	0.7340	0.3306	67.67
Furnariidae	*Thripophaga macroura*	Australasia	0	-0.0277	-	2.53	0.6935	0.3767	45.63
Furnariidae	*Upucerthia albigula*	Neotropics	0	0.2149	9.8906	4.27	0.7069	0.5178	23.05
Furnariidae	*Upucerthia dumetaria*	Neotropics	0	-0.1509	10.2127	3.65	0.7779	0.3306	71.82
Furnariidae	*Upucerthia jelskii*	Palearctic	0	-0.6691	-	4.07	0.8685	0.1040	2.11
Furnariidae	*Upucerthia saturatior*	Neotropics	0	-0.2827	7.2248	3.03	0.6824	0.2105	28.39
Furnariidae	*Upucerthia validirostris*	Neotropics	0	-0.2968	-	3.03	0.6302	0.3362	32.48
Furnariidae	*Xenerpestes minlosi*	Afrotropics	0	-0.1926	6.4352	3.13	0.7399	0.3296	68.66
Furnariidae	*Xenerpestes singularis*	Nearctic	1	-0.6736	-	4.37	0.8185	0.6148	21.86
Furnariidae	*Xenops minutus*	Neotropics	0	-0.2458	-	3.52	0.8417	0.5452	66.16
Furnariidae	*Xenops rutilans*	Nearctic	0	-0.7297	-	4.21	0.8446	0.4246	17.89
Furnariidae	*Xenops tenuirostris*	Nearctic	0	-0.6928	-	4.21	0.8283	0.7730	29.33
Galbulidae	*Brachygalba albogularis*	Neotropics	0	-0.2850	3.8971	3.17	0.8071	0.3099	64.38
Galbulidae	*Brachygalba goeringi*	Australasia	1	-0.5680	2.5522	2.92	0.7928	0.3259	7.56
Galbulidae	*Brachygalba lugubris*	Palearctic	0	-0.6862	3.9552	4.57	0.8633	0.3506	6.50
Galbulidae	*Brachygalba salmoni*	Australasia	0	-0.4742	3.5788	2.32	0.7281	0.2445	45.91
Galbulidae	*Galbalcyrhynchus leucotis*	Afrotropics	0	0.0095	-	3.27	0.8182	0.2888	17.34
Galbulidae	*Galbalcyrhynchus purusianus*	Afrotropics	0	0.1139	7.0703	3.28	0.8168	0.3120	15.45
Galbulidae	*Galbula albirostris*	Palearctic	0	-0.4802	-	4.45	0.7762	0.2259	4.13
Galbulidae	*Galbula chalcothorax*	Palearctic	0	-0.7176	-	4.18	0.7507	0.0450	0.31
Galbulidae	*Galbula cyanescens*	Palearctic	0	-0.4962	-	4.84	0.7574	0.0293	0.58
Galbulidae	*Galbula cyanicollis*	Neotropics	0	0.1321	-	3.96	0.7990	0.9003	4.97
Galbulidae	*Galbula dea*	Neotropics	0	0.0026	-	4.10	0.7883	0.5576	27.30
Galbulidae	*Galbula galbula*	IndoMalay	1	0.0007	-	2.73	0.7576	0.7635	4.27
Galbulidae	*Galbula leucogastra*	Australasia	0	-0.2106	-	2.77	0.8074	0.2744	1.40
Galbulidae	*Galbula pastazae*	Neotropics	0	-0.1713	-	2.86	0.7151	0.3019	40.30
Galbulidae	*Galbula tombacea*	Palearctic	0	-0.6009	-	4.50	0.8545	0.3075	9.26
Galbulidae	*Jacamaralcyon tridactyla*	Neotropics	0	0.0539	-	3.90	0.7570	0.3425	14.09
Galbulidae	*Jacamerops aureus*	Neotropics	0	0.3563	-	3.99	0.8251	0.4704	7.45
Glareolidae	*Cursorius coromandelicus*	Afrotropics	0	-0.0959	8.2596	3.52	0.8552	0.6989	39.81
Glareolidae	*Cursorius cursor*	Australasia	0	-0.0004	4.7142	2.73	0.7112	0.5666	51.47
Glareolidae	*Cursorius rufus*	IndoMalay	0	-0.5530	10.0844	3.40	0.8384	0.4299	3.15
Glareolidae	*Cursorius somalensis*	Afrotropics	0	-0.0657	-	3.62	0.8483	0.4946	45.18
Glareolidae	*Cursorius temminckii*	IndoMalay	0	-0.0742	10.0741	2.81	0.7747	0.4796	15.94
Glareolidae	*Glareola cinerea*	Neotropics	1	0.0000	6.5974	3.91	0.6428	0.2705	11.81
Glareolidae	*Glareola lactea*	Nearctic	0	-0.6981	-	4.15	0.8536	0.5234	10.26
Glareolidae	*Glareola maldivarum*	Neotropics	0	-0.2154	9.1288	3.60	0.7469	0.2341	22.98
Glareolidae	*Glareola nordmanni*	Neotropics	1	-0.3697	2.6374	2.52	0.7078	0.1084	19.70
Glareolidae	*Glareola ocularis*	Neotropics	0	-0.3954	12.0404	3.04	0.6804	0.2128	30.89
Glareolidae	*Glareola pratincola*	Neotropics	0	-0.2608	-	2.50	0.7205	0.1442	18.97
Glareolidae	*Pluvianus aegyptius*	Neotropics	0	0.0273	-	4.00	0.8291	0.7513	7.45
Glareolidae	*Rhinoptilus africanus*	Neotropics	0	0.1314	1.5102	4.07	0.8178	0.8503	52.48
Glareolidae	*Rhinoptilus bitorquatus*	Neotropics	0	0.0337	2.9591	4.08	0.7884	0.6174	29.04
Glareolidae	*Rhinoptilus chalcopterus*	Neotropics	0	-0.0768	2.6895	3.38	0.7602	0.2171	33.39
Glareolidae	*Rhinoptilus cinctus*	Neotropics	0	0.2204	2.7995	3.00	0.7158	0.1453	69.52
Glareolidae	*Stiltia isabella*	Australasia	0	-0.5169	-	3.33	0.8367	0.5793	5.81
Gruidae	*Balearica pavonina*	Nearctic	0	-0.4924	13.9511	4.40	0.7853	0.5284	23.70
Gruidae	*Balearica regulorum*	Nearctic	0	-0.4000	12.3736	3.96	0.7696	0.2279	50.67
Gruidae	*Grus antigone*	Neotropics	0	0.6368	6.0575	3.01	0.6891	0.1479	68.47
Gruidae	*Grus canadensis*	Neotropics	1	0.3416	4.8015	3.17	0.6997	0.3167	57.61
Gruidae	*Grus grus*	Neotropics	0	-0.0321	11.1323	3.74	0.7984	0.4301	38.99
Gruidae	*Grus japonensis*	Neotropics	0	-0.0031	6.8059	3.39	0.7096	0.1717	44.99
Gruidae	*Grus monacha*	Neotropics	1	0.0000	3.0499	3.62	0.5985	0.5771	46.13
Gruidae	*Grus nigricollis*	Neotropics	1	-0.6781	7.7178	3.05	0.6120	0.9804	90.00
Gruidae	*Grus rubicunda*	Neotropics	0	0.0000	6.2532	3.63	0.6221	0.5774	18.43
Gruidae	*Grus vipio*	Neotropics	0	-0.0255	13.1727	3.37	0.7335	0.2473	63.90
Haematopodidae	*Haematopus ater*	Afrotropics	0	-0.6614	-	3.35	0.7909	0.8816	19.71
Haematopodidae	*Haematopus bachmani*	Neotropics	0	-0.0131	-	3.29	0.7570	0.3077	28.73
Haematopodidae	*Haematopus finschi*	Australasia	0	-0.0587	3.3464	2.77	0.7721	0.2939	22.65
Haematopodidae	*Haematopus fuliginosus*	Australasia	1	0.0245	6.7320	2.71	0.7179	0.5352	59.02
Haematopodidae	*Haematopus leucopodus*	Neotropics	0	0.1230	-	4.06	0.8301	0.6726	3.46
Haematopodidae	*Haematopus longirostris*	Neotropics	0	-0.3169	-	2.53	0.6974	0.1632	17.43
Haematopodidae	*Haematopus moquini*	Nearctic	1	-0.6461	7.5921	3.38	0.7548	0.4366	54.47
Haematopodidae	*Haematopus ostralegus*	Neotropics	0	0.0143	-	3.90	0.8006	0.5937	6.33
Haematopodidae	*Haematopus palliatus*	Australasia	1	-0.0522	3.6321	2.58	0.6221	0.1501	33.56
Heliornithidae	*Heliopais personatus*	Neotropics	0	-0.1530	2.5309	3.88	0.8401	0.5215	40.29
Heliornithidae	*Heliornis fulica*	Neotropics	1	-0.3958	3.9972	3.44	0.8253	0.6252	44.86
Heliornithidae	*Podica senegalensis*	Afrotropics	0	0.0641	1.3734	3.30	0.7234	0.2809	84.57
Hemiprocnidae	*Hemiprocne comata*	Neotropics	0	15.6131	6.5169	3.41	0.6690	0.2062	89.68
Hemiprocnidae	*Hemiprocne coronata*	Neotropics	1	0.0000	8.5206	3.59	0.6370	0.3528	46.36
Hemiprocnidae	*Hemiprocne longipennis*	Neotropics	0	0.3852	8.7104	3.13	0.6662	0.1376	69.71
Hemiprocnidae	*Hemiprocne mystacea*	Neotropics	0	0.0106	5.0905	3.40	0.7008	0.3269	76.02
Hirundinidae	*Alopochelidon fucata*	IndoMalay	0	0.0505	-	2.84	0.7883	0.4657	3.68

Family	Species	Realm	Threat	Latitude	Elevation	Anomaly	Size	Shape	Orientation
Hirundinidae	*Atticora fasciata*	Neotropics	0	-0.2832	22.7824	3.65	0.7871	0.2788	87.94
Hirundinidae	*Atticora melanoleuca*	Neotropics	0	-0.4428	7.2538	2.07	0.7389	0.2267	89.84
Hirundinidae	*Cheramoeca leucosterna*	Australasia	0	-0.0737	11.1275	2.81	0.7399	0.1506	24.11
Hirundinidae	*Delichon dasypus*	Australasia	0	-0.0489	-	2.74	0.6804	0.1479	17.39
Hirundinidae	*Delichon nipalense*	Neotropics	0	0.6220	-	3.78	0.7841	0.4895	43.48
Hirundinidae	*Delichon urbicum*	Neotropics	1	-0.6866	2.8135	2.99	0.6598	0.4970	56.01
Hirundinidae	*Eurochelidon sirintarae*	Afrotropics	0	0.0310	-	3.34	0.7597	0.6177	19.41
Hirundinidae	*Haplochelidon andecola*	Palearctic	0	-0.6148	-	4.67	0.8486	0.2075	3.55
Hirundinidae	*Hirundo abyssinica*	IndoMalay	0	-0.7042	-	2.61	0.6478	0.3444	72.46
Hirundinidae	*Hirundo aethiopica*	IndoMalay	0	-0.0871	11.4418	4.08	0.7522	0.1617	17.54
Hirundinidae	*Hirundo albigularis*	Palearctic	0	-0.3504	10.3988	3.24	0.7768	0.7155	69.54
Hirundinidae	*Hirundo angolensis*	IndoMalay	0	0.0581	9.4963	3.50	0.7519	0.7001	3.12
Hirundinidae	*Hirundo ariel*	IndoMalay	0	-0.2337	8.9348	3.20	0.7392	0.8269	60.64
Hirundinidae	*Hirundo atrocaerulea*	IndoMalay	0	-0.0590	-	3.14	0.7988	0.4094	72.13
Hirundinidae	*Hirundo concolor*	IndoMalay	0	-0.0188	8.5164	3.64	0.7435	0.6176	14.55
Hirundinidae	*Hirundo cucullata*	Palearctic	0	-0.5765	-	4.90	0.8120	0.1946	2.91
Hirundinidae	*Hirundo daurica*	Nearctic	0	-1.1861	-	4.10	0.7904	0.2951	4.50
Hirundinidae	*Hirundo dimidiata*	Neotropics	0	-0.1891	-	2.92	0.6763	0.2172	7.75
Hirundinidae	*Hirundo domicola*	Neotropics	0	-0.1260	-	3.14	0.7300	0.3326	60.84
Hirundinidae	*Hirundo fluvicola*	Afrotropics	0	-0.0989	12.2563	3.46	0.8308	0.7547	37.64
Hirundinidae	*Hirundo fuliginosa*	Australasia	0	-0.5128	5.4576	3.37	0.8368	0.5665	7.10
Hirundinidae	*Hirundo fuligula*	Palearctic	0	-0.3941	8.8614	4.18	0.8414	0.3915	5.80
Hirundinidae	*Hirundo leucosoma*	Afrotropics	0	-0.0991	12.5019	3.60	0.8525	0.7504	33.48
Hirundinidae	*Hirundo lucida*	Australasia	0	-0.0881	4.3726	2.78	0.7702	0.3011	24.24
Hirundinidae	*Hirundo megaensis*	Afrotropics	0	-0.4167	12.6504	3.77	0.8763	0.6589	25.97
Hirundinidae	*Hirundo neoxena*	Australasia	0	-0.3891	-	3.29	0.8388	0.4810	22.71
Hirundinidae	*Hirundo nigricans*	Neotropics	0	-0.3198	4.5560	3.46	0.8471	0.3155	46.98
Hirundinidae	*Hirundo nigrita*	Australasia	1	-0.3560	-	2.40	0.7185	0.2914	48.83
Hirundinidae	*Hirundo nigrorufa*	Afrotropics	0	-0.0484	-	3.23	0.8084	0.2279	12.68
Hirundinidae	*Hirundo preussi*	Palearctic	0	-0.8648	0.0000	4.81	0.8429	0.3879	3.15
Hirundinidae	*Hirundo rufigula*	Palearctic	0	-0.5421	-	4.58	0.8357	0.2674	2.09
Hirundinidae	*Hirundo rupestris*	Palearctic	0	-1.0462	8.8127	4.12	0.8171	0.4045	0.45
Hirundinidae	*Hirundo rustica*	Palearctic	0	-0.7876	-	3.86	0.7548	0.2860	17.97
Hirundinidae	*Hirundo semirufa*	Palearctic	0	-0.2948	8.0096	3.67	0.7747	0.4315	18.31
Hirundinidae	*Hirundo senegalensis*	Palearctic	0	-0.6131	7.6606	3.96	0.8335	0.3767	5.98
Hirundinidae	*Hirundo smithii*	Palearctic	0	-0.6572	-	3.56	0.8000	0.6425	30.29
Hirundinidae	*Hirundo spilodera*	Palearctic	0	-0.9231	-	4.22	0.8184	0.7554	20.68
Hirundinidae	*Hirundo tahitica*	Palearctic	0	-0.9499	12.6696	4.35	0.8344	0.4571	14.59
Hirundinidae	*Neochelidon tibialis*	Afrotropics	0	0.0640	-	3.18	0.7791	0.2070	2.36
Hirundinidae	*Notiochelidon flavipes*	Australasia	0	-0.3274	-	3.18	0.8026	0.6682	59.78
Hirundinidae	*Notiochelidon murina*	Afrotropics	0	-0.1255	-	3.10	0.7392	0.1930	74.27
Hirundinidae	*Notiochelidon pileata*	Afrotropics	0	-0.3810	-	3.25	0.6997	0.4879	60.73
Hirundinidae	*Petrochelidon fulva*	Neotropics	1	-32.0181	8.3972	3.53	0.6763	0.3418	63.57
Hirundinidae	*Petrochelidon pyrrhonota*	Neotropics	0	-0.3781	3.7912	3.44	0.8317	0.4789	46.78
Hirundinidae	*Petrochelidon rufocollaris*	Neotropics	0	-0.1842	5.3499	3.84	0.8364	0.5746	34.67
Hirundinidae	*Phedina borbonica*	Nearctic	0	-0.6925	7.9617	4.22	0.8027	0.8176	70.19
Hirundinidae	*Phedina brazzae*	Neotropics	0	-0.3736	6.8872	3.63	0.7128	0.2513	21.34
Hirundinidae	*Progne chalybea*	Australasia	0	-0.5729	-	3.63	0.7624	0.5554	49.36
Hirundinidae	*Progne cryptoleuca*	Australasia	0	-0.1074	-	2.78	0.7688	0.3032	24.42
Hirundinidae	*Progne dominicensis*	Australasia	0	-0.7121	-	3.28	0.7858	0.8854	41.32
Hirundinidae	*Progne elegans*	Australasia	0	-0.7843	-	3.28	0.8250	0.3368	8.15
Hirundinidae	*Progne modesta*	Australasia	0	-0.3829	-	3.45	0.8204	0.5057	12.78
Hirundinidae	*Progne murphyi*	Nearctic	0	-0.7787	-	4.26	0.8367	0.3603	4.08
Hirundinidae	*Progne sinaloae*	Neotropics	1	-0.1533	4.9344	3.59	0.6891	0.3299	68.24
Hirundinidae	*Progne subis*	Neotropics	0	-10.3606	8.3293	3.98	0.7050	0.3118	83.24
Hirundinidae	*Progne tapera*	Neotropics	0	-0.7174	3.9103	2.55	0.7663	0.5523	43.63
Hirundinidae	*Psalidoprocne albiceps*	Neotropics	0	0.3210	-	4.07	0.8050	0.5718	4.87
Hirundinidae	*Psalidoprocne fuliginosa*	Neotropics	0	-0.1279	2.9193	3.92	0.7958	0.7115	20.34
Hirundinidae	*Psalidoprocne nitens*	Neotropics	0	-0.1933	-	3.78	0.8339	0.6113	52.48
Hirundinidae	*Psalidoprocne obscura*	Nearctic	0	-0.8963	-	4.28	0.8465	0.4150	6.70
Hirundinidae	*Psalidoprocne pristoptera*	Australasia	0	-0.0163	-	2.90	0.8191	0.3839	23.41
Hirundinidae	*Pseudhirundo griseopyga*	Afrotropics	0	0.0565	-	3.63	0.7136	0.0903	5.70
Hirundinidae	*Pseudochelidon eurystomina*	Afrotropics	0	-0.0177	-	3.33	0.8110	0.6288	9.64
Hirundinidae	*Pygochelidon cyanoleuca*	Neotropics	1	0.0583	3.0323	3.12	0.7240	0.3956	45.24
Hirundinidae	*Riparia cincta*	Neotropics	0	-0.1827	-	3.31	0.7732	0.2617	29.86
Hirundinidae	*Riparia congica*	Neotropics	0	0.0705	-	3.23	0.7637	0.6558	57.58
Hirundinidae	*Riparia paludicola*	Neotropics	0	-0.2010	-	3.75	0.8249	0.7829	79.15
Hirundinidae	*Riparia riparia*	Neotropics	0	-0.0496	4.0602	4.03	0.8379	0.7646	6.03
Hirundinidae	*Stelgidopteryx ruficollis*	Palearctic	0	-0.7148	9.8744	3.81	0.7507	0.4355	19.76
Hirundinidae	*Stelgidopteryx serripennis*	Palearctic	1	0.1125	9.6559	3.53	0.5788	0.0000	0.00
Hirundinidae	*Tachycineta albilinea*	Australasia	1	-0.7096	-	2.72	0.6716	0.4513	17.01
Hirundinidae	*Tachycineta albiventer*	Australasia	0	-0.5757	-	3.68	0.7739	0.2608	1.25
Hirundinidae	*Tachycineta bicolor*	Afrotropics	0	0.0227	6.4510	3.33	0.8202	0.4739	14.65
Hirundinidae	*Tachycineta cyaneoviridis*	Australasia	0	-0.5373	-	3.27	0.7960	0.4214	82.99

Family	Species	Realm	Threat	Latitude	Elevation	Anomaly	Size	Shape	Orientation
Hirundinidae	*Tachycineta euchrysea*	Australasia	0	-0.8583	-	2.98	0.8043	0.2469	12.82
Hirundinidae	*Tachycineta leucorrhoa*	Australasia	0	-0.0958	-	2.71	0.7185	0.5623	60.71
Hirundinidae	*Tachycineta meyeni*	IndoMalay	0	-0.1346	-	2.65	0.7533	0.4170	64.51
Hirundinidae	*Tachycineta stolzmanni*	Afrotropics	0	-0.1390	-	3.64	0.8453	0.4771	55.43
Hirundinidae	*Tachycineta thalassina*	Palearctic	0	-0.3308	-	3.91	0.8632	0.2564	6.73
Ibidorhynchidae	*Ibidorhyncha struthersii*	Neotropics	0	-0.1243	-	3.96	0.8126	0.8435	0.55
Icteridae	*Agelaioides badius*	Afrotropics	0	0.0185	9.5748	3.55	0.7593	0.5053	82.87
Icteridae	*Agelaius assimilis*	Neotropics	0	-0.3761	7.2535	3.26	0.8240	0.4141	46.88
Icteridae	*Agelaius humeralis*	Neotropics	0	-0.3591	-	2.54	0.6302	0.3028	5.97
Icteridae	*Agelaius phoeniceus*	Neotropics	0	-0.4466	-	2.53	0.7008	0.1290	18.24
Icteridae	*Agelaius tricolor*	Nearctic	0	-0.7528	9.1971	4.13	0.8553	0.5739	22.46
Icteridae	*Agelaius xanthomus*	Nearctic	1	-0.5704	3.8201	3.36	0.7314	0.3660	73.25
Icteridae	*Agelasticus cyanopus*	Afrotropics	0	0.0934	-	3.22	0.7783	0.3631	4.41
Icteridae	*Agelasticus thilius*	Neotropics	0	-0.2892	-	3.59	0.8124	0.6361	66.64
Icteridae	*Agelasticus xanthophthalmus*	Neotropics	0	-0.6445	7.7642	2.79	0.8083	0.7148	81.48
Icteridae	*Amblycercus holosericeus*	Neotropics	0	-0.2414	-	3.68	0.8428	0.8255	85.64
Icteridae	*Amblyramphus holosericeus*	Afrotropics	0	-0.1338	-	3.41	0.8244	0.7291	31.89
Icteridae	*Cacicus cela*	Australasia	0	-0.3946	-	3.16	0.8215	0.6033	66.45
Icteridae	*Cacicus chrysonotus*	Neotropics	0	-0.3946	-	2.50	0.6120	0.0218	6.74
Icteridae	*Cacicus chrysopterus*	IndoMalay	1	-0.0032	0.2878	2.62	0.7223	0.5229	58.36
Icteridae	*Cacicus haemorrhous*	Australasia	0	-0.6880	-	3.32	0.8143	0.4872	14.58
Icteridae	*Cacicus koepckeae*	Australasia	1	0.2491	2.8780	2.48	0.6370	0.1926	10.82
Icteridae	*Cacicus melanicterus*	Australasia	1	-0.3344	3.4173	2.50	0.6716	0.2680	24.42
Icteridae	*Cacicus sclateri*	Australasia	0	-0.7653	-	2.99	0.7205	0.5416	78.49
Icteridae	*Cacicus solitarius*	Australasia	0	-0.5595	-	3.34	0.8379	0.5807	7.72
Icteridae	*Cacicus uropygialis*	Australasia	0	-0.4522	-	3.37	0.8294	0.5592	15.91
Icteridae	*Chrysomus icterocephalus*	Afrotropics	0	-0.1545	-	3.49	0.8555	0.6017	35.73
Icteridae	*Chrysomus ruficapillus*	Australasia	0	-0.4764	6.2656	3.03	0.8115	0.5148	10.57
Icteridae	*Clypicterus oseryi*	Neotropics	0	-0.4709	12.7583	3.50	0.8396	0.2652	67.98
Icteridae	*Curaeus curaeus*	Neotropics	0	-0.1018	4.5336	4.00	0.8299	0.6485	2.67
Icteridae	*Curaeus forbesi*	Palearctic	0	-0.8678	-	4.45	0.8801	0.2960	0.99
Icteridae	*Dives atroviolaceus*	Neotropics	0	-0.4032	8.9100	3.99	0.7363	0.1695	39.13
Icteridae	*Dives dives*	Neotropics	0	0.0016	11.4038	3.04	0.6784	0.1941	29.99
Icteridae	*Dives warszewiczi*	Neotropics	0	-0.2095	5.8819	3.70	0.7794	0.3843	74.64
Icteridae	*Dolichonyx oryzivorus*	Neotropics	1	-0.6252	8.4991	3.77	0.5985	0.0000	0.00
Icteridae	*Euphagus carolinus*	Australasia	0	-0.0525	4.7726	2.71	0.7185	0.5623	60.71
Icteridae	*Euphagus cyanocephalus*	Australasia	0	-0.3298	-	3.25	0.7980	0.4538	32.66
Icteridae	*Gnorimopsar chopi*	Palearctic	0	-0.3859	6.2802	3.92	0.8365	0.4738	21.95
Icteridae	*Gymnomystax mexicanus*	Neotropics	0	-0.2637	0.0000	3.36	0.7461	0.4967	13.82
Icteridae	*Hypopyrrhus pyrohypogaster*	Neotropics	0	0.0200	-	3.33	0.7821	0.3203	42.93
Icteridae	*Icterus abeillei*	Neotropics	0	0.0852	3.6188	3.73	0.5985	0.5765	47.24
Icteridae	*Icterus auratus*	Australasia	1	-0.4318	-	2.33	0.7291	0.1817	40.61
Icteridae	*Icterus auricapillus*	Neotropics	0	-0.5452	7.7806	2.68	0.8103	0.5637	48.37
Icteridae	*Icterus bullockii*	Afrotropics	0	-0.2939	-	3.29	0.7286	0.5896	65.63
Icteridae	*Icterus cayanensis*	Afrotropics	0	-0.0946	-	3.56	0.8037	0.7875	43.18
Icteridae	*Icterus chrysater*	Afrotropics	0	0.1001	-	3.42	0.8079	0.1679	9.54
Icteridae	*Icterus croconotus*	Neotropics	0	-0.0663	-	3.92	0.7771	0.4569	1.10
Icteridae	*Icterus cucullatus*	Neotropics	0	-0.0059	3.4930	4.01	0.7804	0.7848	41.82
Icteridae	*Icterus dominicensis*	Neotropics	0	0.3034	3.8499	4.07	0.7639	0.4858	25.68
Icteridae	*Icterus galbula*	Neotropics	0	-0.1465	-	3.86	0.7908	0.4252	1.77
Icteridae	*Icterus graceannae*	Neotropics	0	-0.0168	5.6115	4.21	0.7595	0.4806	40.88
Icteridae	*Icterus graduacauda*	Neotropics	0	0.0760	3.2432	4.02	0.8015	0.6859	10.99
Icteridae	*Icterus gularis*	Neotropics	0	0.0974	-	4.15	0.7868	0.6754	11.35
Icteridae	*Icterus icterus*	Neotropics	0	0.1078	5.3036	4.12	0.7570	0.3689	30.71
Icteridae	*Icterus jamacaii*	Neotropics	0	0.0040	-	4.10	0.8171	0.4749	6.30
Icteridae	*Icterus jamacaii*	Neotropics	0	0.0040	-	4.10	0.8171	-	-
Icteridae	*Icterus leucopteryx*	Neotropics	0	-0.0898	-	3.99	0.8181	0.5869	2.55
Icteridae	*Icterus maculialatus*	Palearctic	0	-0.5846	3.4298	4.26	0.7718	0.2566	10.25
Icteridae	*Icterus melanopsis*	IndoMalay	0	-0.1250	-	2.63	0.6784	0.6648	80.50
Icteridae	*Icterus mesomelas*	Neotropics	0	-0.2028	-	3.35	0.7880	0.4482	27.07
Icteridae	*Icterus nigrogularis*	IndoMalay	0	-0.0710	-	2.92	0.8042	0.4985	59.42
Icteridae	*Icterus northropi*	Neotropics	1	3.2797	8.4074	3.17	0.6986	0.2064	87.52
Icteridae	*Icterus parisorum*	Afrotropics	0	-0.1539	-	3.05	0.7300	0.1625	75.56
Icteridae	*Icterus portoricensis*	IndoMalay	0	-0.0841	-	3.09	0.8363	0.5567	27.93
Icteridae	*Icterus prosthemelas*	IndoMalay	1	-0.0114	1.9557	2.64	0.7281	0.2159	67.95
Icteridae	*Icterus pustulatus*	IndoMalay	0	0.0172	2.5681	2.64	0.7120	0.1798	61.67
Icteridae	*Icterus pyrrhopterus*	IndoMalay	0	-0.0833	-	3.41	0.8286	0.5011	1.53
Icteridae	*Icterus spurius*	Afrotropics	0	-0.0726	-	3.18	0.7614	0.3874	70.15
Icteridae	*Icterus wagleri*	IndoMalay	0	-0.2374	-	3.29	0.8123	0.5476	1.44
Icteridae	*Lampropsar tanagrinus*	Afrotropics	0	1.9936	3.8657	3.41	0.7078	0.2243	71.94
Icteridae	*Macroagelaius subalaris*	IndoMalay	0	0.0167	-	2.83	0.7889	0.4809	5.17
Icteridae	*Molothrus aeneus*	Australasia	0	-0.0497	-	2.71	0.7480	0.2872	21.11
Icteridae	*Molothrus ater*	Neotropics	0	-0.2595	-	3.95	0.8136	0.5667	28.83
Icteridae	*Molothrus bonariensis*	Neotropics	0	-0.0508	-	3.95	0.8243	0.5073	29.82

Family	Species	Realm	Threat	Latitude	Elevation	Anomaly	Size	Shape	Orientation
Icteridae	*Molothrus oryzivorus*	Neotropics	0	-4.8437	6.0497	3.78	0.7234	0.1605	83.99
Icteridae	*Molothrus rufoaxillaris*	Palearctic	0	-0.5962	1.8397	3.89	0.8074	0.2104	0.10
Icteridae	*Nesopsar nigerrimus*	Afrotropics	0	-0.0704	7.6654	3.18	0.7614	0.3874	70.15
Icteridae	*Ocyalus latirostris*	Neotropics	0	0.3430	0.8715	4.00	0.7477	0.2221	79.76
Icteridae	*Oreopsar bolivianus*	IndoMalay	0	0.1484	2.1677	2.77	0.6804	0.5220	77.37
Icteridae	*Psarocolius angustifrons*	Palearctic	0	-0.6141	-	4.66	0.8378	0.2708	1.88
Icteridae	*Psarocolius atrovirens*	Palearctic	0	-0.8728	-	4.14	0.7953	0.4376	41.78
Icteridae	*Psarocolius bifasciatus*	Palearctic	0	-0.8422	-	4.78	0.8504	0.2434	2.67
Icteridae	*Psarocolius cassini*	Palearctic	0	-0.4501	7.7082	4.12	0.8630	0.5233	34.35
Icteridae	*Psarocolius guatimozinus*	Neotropics	1	-0.4892	-	2.36	0.7515	0.7275	62.35
Icteridae	*Psarocolius montezuma*	Australasia	0	-0.2941	7.6667	2.97	0.8114	0.6611	42.28
Icteridae	*Psarocolius viridis*	Neotropics	0	0.0489	11.4890	3.59	0.7758	0.4151	76.11
Icteridae	*Psarocolius wagleri*	Neotropics	1	-0.1850	8.8297	3.68	0.6221	0.5865	19.14
Icteridae	*Pseudoleistes guirahuro*	IndoMalay	0	-0.0830	-	3.38	0.8037	0.6513	88.94
Icteridae	*Pseudoleistes virescens*	IndoMalay	0	-0.0293	4.3223	2.64	0.7314	0.2285	67.50
Icteridae	*Quiscalus lugubris*	Australasia	0	-0.0004	3.6552	2.75	0.7815	0.3229	16.39
Icteridae	*Quiscalus nicaraguensis*	Australasia	0	-0.0911	3.3828	2.58	0.7019	0.1316	9.38
Icteridae	*Quiscalus niger*	Australasia	0	0.1227	0.0575	2.49	0.5985	0.1250	23.08
Icteridae	*Quiscalus quiscula*	IndoMalay	1	0.2949	1.4806	2.52	0.5788	0.0000	45.38
Icteridae	*Sturnella bellicosa*	Nearctic	0	-0.7381	10.0775	4.01	0.8167	0.4779	37.71
Icteridae	*Sturnella defilippii*	Nearctic	0	-0.6641	-	4.30	0.8183	0.2838	15.95
Icteridae	*Sturnella loyca*	Nearctic	0	-0.7303	-	4.14	0.8543	0.5333	20.53
Icteridae	*Sturnella magna*	Nearctic	0	-0.6555	-	4.28	0.8226	0.5321	4.18
Icteridae	*Sturnella militaris*	Nearctic	1	-0.4022	7.4519	3.82	0.6961	0.4019	60.54
Icteridae	*Sturnella neglecta*	Neotropics	0	-0.5480	2.6382	2.97	0.7923	0.3984	73.89
Icteridae	*Sturnella superciliaris*	IndoMalay	0	-0.2390	14.3784	3.24	0.7875	0.7213	9.62
Icteridae	*Xanthocephalus xanthocephalus*	IndoMalay	0	-0.0635	2.9363	2.66	0.6997	0.4736	75.00
Icteridae	*Xanthopsar flavus*	Afrotropics	0	-0.0042	-	3.44	0.8471	0.4725	22.17
Indicatoridae	*Indicator archipelagicus*	Neotropics	0	-0.2048	-	3.44	0.7681	0.4267	36.15
Indicatoridae	*Indicator exilis*	Neotropics	0	-0.2395	1.0318	3.97	0.7560	0.6168	7.51
Indicatoridae	*Indicator indicator*	Neotropics	0	-0.1082	2.1033	3.67	0.8017	0.8884	67.83
Indicatoridae	*Indicator maculatus*	Neotropics	0	-1.0098	-	2.47	0.5985	0.0000	0.00
Indicatoridae	*Indicator meliphilus*	Neotropics	0	0.1585	6.5606	3.61	0.7128	0.2538	29.91
Indicatoridae	*Indicator minor*	Neotropics	0	-0.3596	1.1814	2.53	0.6974	0.1632	17.43
Indicatoridae	*Indicator pumilio*	Neotropics	0	0.3118	-	3.33	0.7864	0.4406	47.39
Indicatoridae	*Indicator variegatus*	Neotropics	0	0.0388	-	3.90	0.7921	0.3004	13.44
Indicatoridae	*Indicator xanthonotus*	Nearctic	0	-0.4757	-	4.09	0.8014	0.4272	47.27
Indicatoridae	*Melichneutes robustus*	Neotropics	0	-0.2933	4.1459	3.23	0.7112	0.4108	78.09
Indicatoridae	*Melignomon eisentrauti*	Nearctic	0	-0.4111	7.7371	3.79	0.7932	0.4281	49.98
Indicatoridae	*Melignomon zenkeri*	Neotropics	0	-0.4802	6.3958	3.22	0.7803	0.4276	84.82
Indicatoridae	*Prodotiscus insignis*	IndoMalay	0	-0.7294	8.8881	3.35	0.8077	0.3975	6.60
Indicatoridae	*Prodotiscus regulus*	IndoMalay	0	-0.0386	-	3.23	0.7961	0.3262	36.12
Indicatoridae	*Prodotiscus zambesiae*	IndoMalay	0	-0.7875	0.0000	3.32	0.7706	0.4856	19.27
Irenidae	*Irena cyanogastra*	Afrotropics	0	1.3110	-	3.33	0.7904	0.5441	18.86
Irenidae	*Irena puella*	Afrotropics	0	0.0337	-	3.13	0.7833	0.2673	15.54
Jacanidae	*Actophilornis africanus*	Afrotropics	0	-0.1062	-	3.54	0.8600	0.6878	41.69
Jacanidae	*Actophilornis albinucha*	Afrotropics	0	-0.0644	2.1673	3.09	0.7172	0.2955	52.77
Jacanidae	*Hydrophasianus chirurgus*	Afrotropics	0	-0.1113	8.1187	3.50	0.8549	0.6157	33.84
Jacanidae	*Irediparra gallinacea*	Neotropics	0	-0.2603	5.5809	3.25	0.7760	0.3836	30.76
Jacanidae	*Jacana jacana*	Neotropics	0	-0.1602	3.1209	3.04	0.7452	0.2278	52.73
Jacanidae	*Jacana spinosa*	IndoMalay	0	-0.0069	-	3.06	0.7399	0.4106	58.09
Jacanidae	*Metopidius indicus*	Australasia	0	-0.5647	-	3.04	0.7933	0.5048	10.63
Jacanidae	*Microparra capensis*	Afrotropics	0	-0.1351	-	3.69	0.8435	0.5536	43.99
Laniidae	*Corvinella corvina*	Afrotropics	0	-0.1244	-	3.57	0.8352	0.5408	37.17
Laniidae	*Eurocephalus anguitimens*	Afrotropics	0	0.0699	-	3.13	0.7428	0.3018	40.07
Laniidae	*Eurocephalus rueppelli*	Afrotropics	0	0.0474	-	3.53	0.8233	0.1840	4.06
Laniidae	*Lanius bucephalus*	Afrotropics	0	0.2147	-	3.07	0.7480	0.2503	11.92
Laniidae	*Lanius cabanisi*	Afrotropics	0	-0.1512	-	3.65	0.7908	0.5672	47.95
Laniidae	*Lanius collaris*	Afrotropics	0	0.3035	-	3.20	0.7567	0.6813	74.15
Laniidae	*Lanius collurio*	Afrotropics	0	-0.1582	-	3.97	0.7833	0.4736	3.96
Laniidae	*Lanius collurioides*	Afrotropics	0	-0.2234	-	3.96	0.8138	0.5613	48.14
Laniidae	*Lanius cristatus*	Afrotropics	0	-0.0017	-	3.78	0.8210	0.1496	0.99
Laniidae	*Lanius dorsalis*	Afrotropics	0	0.1061	-	3.26	0.8018	0.4549	5.05
Laniidae	*Lanius excubitor*	Afrotropics	0	-0.0202	-	3.47	0.8188	0.1773	8.51
Laniidae	*Lanius excubitoroides*	Afrotropics	0	0.2446	7.4383	3.39	0.8007	0.7468	83.10
Laniidae	*Lanius gubernator*	Afrotropics	0	-0.0470	-	3.45	0.7716	0.3394	24.05
Laniidae	*Lanius isabellinus*	Afrotropics	0	-0.0298	-	3.35	0.8230	0.4638	19.59
Laniidae	*Lanius ludovicianus*	Afrotropics	0	-0.0682	-	3.33	0.8047	0.5117	50.69
Laniidae	*Lanius mackinnoni*	Afrotropics	0	0.0192	12.5850	3.48	0.8394	0.7264	23.14
Laniidae	*Lanius marwitzi*	Afrotropics	1	2.5010	1.2373	3.30	0.5788	0.0000	90.00
Laniidae	*Lanius minor*	Afrotropics	0	-0.2278	-	4.20	0.8041	0.6809	29.08
Laniidae	*Lanius nubicus*	Afrotropics	0	0.5473	8.3754	3.23	0.7019	0.4687	34.26
Laniidae	*Lanius schach*	Afrotropics	0	0.2363	-	3.56	0.8132	0.2759	5.52

Family	Species	Realm	Threat	Latitude	Elevation	Anomaly	Size	Shape	Orientation
Laniidae	*Lanius senator*	Afrotropics	0	0.0543	-	3.89	0.7660	0.3846	46.56
Laniidae	*Lanius somalicus*	Afrotropics	1	-0.6276	-1.5923	3.17	0.6120	0.9804	90.00
Laniidae	*Lanius souzae*	Afrotropics	0	3.4393	-	3.47	0.8062	0.6742	10.17
Laniidae	*Lanius sphenocercus*	Afrotropics	0	-0.5143	-	3.40	0.7743	0.3599	46.95
Laniidae	*Lanius tephronotus*	Afrotropics	0	-0.0498	9.5385	3.33	0.7223	0.2200	55.02
Laniidae	*Lanius tigrinus*	Afrotropics	0	-0.0961	-	3.32	0.7937	0.4595	56.65
Laniidae	*Lanius validirostris*	Afrotropics	0	0.1183	-	3.26	0.8096	0.2519	11.95
Laniidae	*Lanius vittatus*	Afrotropics	0	0.2712	-	3.25	0.7949	0.3411	0.05
Laniidae	*Urolestes melanoleucus*	Australasia	0	-0.0967	1.4003	2.78	0.7707	0.3041	24.05
Laridae	*Chlidonias hybrida*	Australasia	0	-0.7218	-	3.39	0.8285	0.3752	7.07
Laridae	*Chlidonias leucopterus*	Afrotropics	0	-0.1894	-	3.94	0.8075	0.6710	65.30
Laridae	*Larus bulleri*	Afrotropics	0	-2.1440	3.0431	3.33	0.7256	0.5600	43.70
Laridae	*Larus relictus*	Afrotropics	0	0.0436	13.9138	3.35	0.7378	0.2048	22.22
Laridae	*Larus serranus*	Afrotropics	0	-0.0995	-	3.32	0.7740	0.5228	56.90
Laridae	*Phaetusa simplex*	IndoMalay	0	-0.4763	5.3933	3.66	0.8512	0.6962	40.89
Laridae	*Rynchops albicollis*	Neotropics	0	-3.8328	-	4.10	0.7580	0.3826	51.40
Laridae	*Rynchops flavirostris*	Neotropics	0	-0.0974	4.6113	3.93	0.8095	0.4366	47.54
Laridae	*Rynchops niger*	Neotropics	0	-0.0645	-	4.05	0.8356	0.7271	4.95
Laridae	*Sterna acuticauda*	Nearctic	0	-0.8158	-	3.74	0.7797	0.4070	8.56
Laridae	*Sterna aurantia*	Nearctic	0	-0.6056	9.8386	4.08	0.8071	0.4288	53.71
Laridae	*Sterna superciliaris*	IndoMalay	0	-0.1643	9.5240	2.81	0.6921	0.4448	76.76
Leptosomidae	*Leptosomus discolor*	Nearctic	0	-0.4057	-	6.25	0.7860	0.2808	1.91
Machaerirhynchidae	*Machaerirhynchus flaviventer*	IndoMalay	0	0.0125	2.0136	2.85	0.7872	0.5331	14.43
Machaerirhynchidae	*Machaerirhynchus nigripectus*	IndoMalay	1	-0.9112	8.3416	2.85	0.7078	0.1340	50.98
Malaconotidae	*Dryoscopus angolensis*	Australasia	0	-0.5507	5.4535	3.28	0.8227	0.4673	1.11
Malaconotidae	*Dryoscopus cubla*	Neotropics	0	-0.1430	-	3.98	0.8164	0.5612	55.36
Malaconotidae	*Dryoscopus gambensis*	Neotropics	0	-0.1681	4.7806	3.85	0.8517	0.4885	36.21
Malaconotidae	*Dryoscopus pringlii*	Afrotropics	0	5.3095	-	3.37	0.7864	0.2753	6.57
Malaconotidae	*Dryoscopus sabini*	Australasia	0	-0.6746	-	2.87	0.7810	0.1532	6.71
Malaconotidae	*Dryoscopus senegalensis*	Australasia	0	-0.1210	-	2.82	0.7637	0.5648	25.81
Malaconotidae	*Laniarius aethiopicus*	Australasia	0	-0.2576	-	2.56	0.6428	0.2897	74.47
Malaconotidae	*Laniarius amboimensis*	Australasia	0	-0.1692	-	2.70	0.7165	0.5498	61.21
Malaconotidae	*Laniarius atroflavus*	IndoMalay	0	-0.0174	-	2.64	0.7272	0.2228	67.18
Malaconotidae	*Laniarius bicolor*	IndoMalay	0	-0.0020	-	2.79	0.7936	0.5366	24.12
Malaconotidae	*Laniarius brauni*	Australasia	0	0.1372	5.0436	2.69	0.7327	0.8162	29.45
Malaconotidae	*Laniarius erythrogaster*	Australasia	0	-0.4762	-	3.34	0.8379	0.5846	5.31
Malaconotidae	*Laniarius ferrugineus*	Neotropics	0	-0.2344	13.1754	3.67	0.7477	0.2171	22.97
Malaconotidae	*Laniarius fuelleborni*	Neotropics	0	0.0243	7.3207	3.15	0.6716	0.1913	35.38
Malaconotidae	*Laniarius funebris*	Neotropics	0	-0.1635	11.8530	3.01	0.6523	0.5119	34.03
Malaconotidae	*Laniarius leucorhynchus*	Nearctic	0	-0.4092	11.8666	3.96	0.7711	0.3029	50.67
Malaconotidae	*Laniarius luehderi*	Neotropics	0	-0.0584	5.0805	3.02	0.6631	0.1815	32.51
Malaconotidae	*Laniarius mufumbiri*	Neotropics	0	0.2134	7.9158	3.49	0.6935	0.7400	70.30
Malaconotidae	*Laniarius poensis*	Neotropics	0	0.0509	11.6429	3.61	0.7192	0.3478	17.19
Malaconotidae	*Laniarius ruficeps*	Neotropics	0	-0.1224	9.9587	3.61	0.7356	0.2193	19.45
Malaconotidae	*Laniarius turatii*	Neotropics	0	-0.0070	-	4.08	0.8090	0.8049	78.81
Malaconotidae	*Malaconotus alius*	Afrotropics	0	0.0576	-	3.31	0.8141	0.3138	6.99
Malaconotidae	*Malaconotus blanchoti*	Afrotropics	1	8.9262	3.1165	3.88	0.7050	0.8080	54.54
Malaconotidae	*Malaconotus cruentus*	Afrotropics	0	0.0088	-	3.49	0.8074	0.1908	8.66
Malaconotidae	*Malaconotus gladiator*	Afrotropics	0	0.1618	7.2599	3.47	0.7931	0.7000	50.07
Malaconotidae	*Malaconotus lagdeni*	Afrotropics	0	0.0557	8.8876	3.37	0.8062	0.4046	24.46
Malaconotidae	*Malaconotus monteiri*	Afrotropics	0	-0.0767	-	3.29	0.7769	0.3369	75.90
Malaconotidae	*Nilaus afer*	Afrotropics	0	0.0331	-	3.21	0.7882	0.2620	9.07
Malaconotidae	*Prionops alberti*	Neotropics	0	-0.1714	2.2162	3.86	0.8278	0.6814	49.74
Malaconotidae	*Prionops caniceps*	Neotropics	0	-0.4217	5.2755	4.40	0.7019	0.4779	13.27
Malaconotidae	*Prionops gabela*	Neotropics	0	-0.1166	0.9795	3.98	0.8024	0.6706	8.85
Malaconotidae	*Prionops plumatus*	IndoMalay	0	-0.7673	8.8052	2.61	0.6562	0.4622	64.29
Malaconotidae	*Prionops poliolophus*	IndoMalay	0	-0.4095	-	3.23	0.7172	0.3641	75.07
Malaconotidae	*Prionops retzii*	IndoMalay	0	0.0846	-	3.32	0.7779	0.4407	42.09
Malaconotidae	*Prionops scopifrons*	Palearctic	0	-0.6943	14.5092	3.52	0.8024	0.5364	52.50
Malaconotidae	*Rhodophoneus cruentus*	Afrotropics	0	-0.0922	-	3.65	0.8400	0.7725	56.30
Malaconotidae	*Tchagra australis*	Australasia	0	-0.0822	5.7493	2.83	0.7432	0.1811	24.15
Malaconotidae	*Tchagra jamesi*	Australasia	0	-0.0842	-	2.77	0.7709	0.4354	29.88
Malaconotidae	*Tchagra minutus*	Neotropics	0	-0.2362	-	3.68	0.8467	0.7380	73.49
Malaconotidae	*Tchagra senegalus*	Neotropics	0	0.6058	5.1716	3.93	0.7884	0.8455	13.38
Malaconotidae	*Tchagra tchagra*	Neotropics	0	-0.1701	-	4.02	0.7374	0.5890	65.31
Malaconotidae	*Telophorus bocagei*	Neotropics	0	-2.9729	0.8816	4.07	0.7413	0.1543	34.45
Malaconotidae	*Telophorus dohertyi*	Neotropics	0	0.0707	2.2177	3.44	0.7223	0.3974	13.54
Malaconotidae	*Telophorus kupeensis*	Neotropics	0	0.8236	5.3508	3.69	0.5985	0.0000	0.00
Malaconotidae	*Telophorus multicolor*	Neotropics	0	-0.0352	6.1774	4.18	0.7651	0.3326	42.20
Malaconotidae	*Telophorus nigrifrons*	Neotropics	0	-0.5490	-	3.00	0.7872	0.4201	39.46
Malaconotidae	*Telophorus olivaceus*	Neotropics	0	-0.4271	7.3885	3.80	0.7223	0.3359	28.22
Malaconotidae	*Telophorus quadricolor*	Neotropics	1	0.0000	6.4289	4.18	0.5788	0.0000	0.00
Malaconotidae	*Telophorus sulfureopectus*	Neotropics	0	-0.1847	-	3.37	0.7515	0.7525	8.74
Malaconotidae	*Telophorus viridis*	Neotropics	0	-0.3360	-	3.55	0.8316	0.3901	46.27

Family	Species	Realm	Threat	Latitude	Elevation	Anomaly	Size	Shape	Orientation
Malaconotidae	*Telophorus zeylonus*	Neotropics	1	0.3881	6.0086	3.25	0.6221	0.5865	19.14
Maluridae	*Amytornis ballarae*	Australasia	0	-0.3446	-	3.64	0.6961	0.5293	37.75
Maluridae	*Amytornis barbatus*	Australasia	0	-0.4862	-	3.50	0.7078	0.5436	43.77
Maluridae	*Amytornis dorotheae*	Australasia	1	-0.2240	0.0000	3.48	0.7314	0.3400	46.48
Maluridae	*Amytornis goyderi*	Australasia	0	-0.4810	-	3.48	0.7403	0.6168	52.85
Maluridae	*Amytornis housei*	Australasia	0	0.0909	-	3.18	0.6690	0.8019	85.17
Maluridae	*Amytornis merrotsyi*	Australasia	0	-1.0177	-	2.90	0.6935	0.3408	29.99
Maluridae	*Amytornis purnelli*	Australasia	0	-6.8098	-	3.57	0.7695	0.8054	45.17
Maluridae	*Amytornis striatus*	Australasia	0	-0.8038	-	3.49	0.7868	0.3814	18.53
Maluridae	*Amytornis textilis*	Australasia	0	-0.7261	-	3.02	0.7526	0.2537	13.27
Maluridae	*Amytornis woodwardi*	Australasia	1	0.0675	-	3.28	0.6302	0.4866	54.42
Maluridae	*Clytomyias insignis*	Afrotropics	0	-0.1825	11.1647	3.59	0.8595	0.4727	22.35
Maluridae	*Malurus alboscapulatus*	IndoMalay	0	-0.4095	-	2.72	0.6997	0.1258	12.28
Maluridae	*Malurus amabilis*	IndoMalay	0	-0.0513	3.8780	3.10	0.8154	0.6180	53.72
Maluridae	*Malurus coronatus*	IndoMalay	0	-0.1031	4.1518	2.89	0.7363	0.3323	60.72
Maluridae	*Malurus cyaneus*	IndoMalay	0	0.0105	1.2801	2.83	0.7712	0.5298	4.33
Maluridae	*Malurus cyanocephalus*	IndoMalay	0	-0.0303	-	2.62	0.7019	0.6235	86.79
Maluridae	*Malurus elegans*	Afrotropics	0	0.0146	12.5132	3.81	0.8035	0.5847	35.32
Maluridae	*Malurus grayi*	Afrotropics	0	-0.1176	-	3.14	0.7550	0.3127	35.26
Maluridae	*Malurus lamberti*	Afrotropics	0	-0.3168	10.9792	3.58	0.7774	0.4514	55.72
Maluridae	*Malurus leucopterus*	Afrotropics	0	-0.0900	8.3345	3.37	0.8363	0.3784	28.48
Maluridae	*Malurus melanocephalus*	Afrotropics	0	-4.9010	10.2080	3.54	0.7558	0.6082	85.72
Maluridae	*Malurus pulcherrimus*	Afrotropics	0	-0.0728	10.1253	3.75	0.8046	0.5337	9.85
Maluridae	*Malurus splendens*	Afrotropics	0	-10.2638	4.1661	3.80	0.7713	0.8793	11.18
Maluridae	*Sipodotus wallacii*	Neotropics	0	-1.2139	7.7043	3.68	0.6478	0.3318	24.70
Maluridae	*Stipiturus malachurus*	Afrotropics	0	0.0043	-	3.24	0.8101	0.2492	10.87
Maluridae	*Stipiturus mallee*	Afrotropics	0	0.2255	8.5043	3.28	0.7654	0.2781	13.86
Maluridae	*Stipiturus ruficeps*	Neotropics	0	-0.2192	8.5021	3.56	0.7256	-	-
Maluridae	*Stipiturus ruficeps*	Neotropics	0	-0.2192	8.5021	3.56	0.7256	0.2323	81.30
Megapodiidae	*Aepypodius arfakianus*	Australasia	0	-0.0472	10.4615	2.82	0.7444	0.1453	24.13
Megapodiidae	*Alectura lathami*	Neotropics	1	-0.3548	6.1315	3.67	0.7858	0.5046	10.02
Megapodiidae	*Eulipoa wallacei*	Afrotropics	0	-0.1714	-	3.55	0.8109	0.1611	5.81
Megapodiidae	*Leipoa ocellata*	Afrotropics	0	0.0387	5.2703	3.52	0.8131	0.4803	23.07
Megapodiidae	*Macrocephalon maleo*	IndoMalay	0	-0.0004	3.1500	2.64	0.7327	0.2318	67.52
Megapodiidae	*Megapodius affinis*	Australasia	0	-0.5777	-	3.23	0.8024	0.5404	87.47
Megapodiidae	*Megapodius bernsteinii*	Australasia	0	-0.5674	-	3.08	0.7472	0.2418	61.14
Megapodiidae	*Megapodius cumingii*	Australasia	1	-0.6985	-	2.78	0.6120	0.8420	0.28
Megapodiidae	*Megapodius eremita*	Australasia	0	-0.1042	2.4786	2.75	0.7378	0.3109	25.64
Megapodiidae	*Megapodius freycinet*	Australasia	0	-0.1566	4.7492	2.72	0.7240	0.1582	23.34
Megapodiidae	*Megapodius layardi*	Australasia	0	0.1701	-	2.79	0.6935	0.2160	9.38
Megapodiidae	*Megapodius reinwardt*	Australasia	0	-0.0936	-	2.78	0.7729	0.4080	28.55
Megapodiidae	*Megapodius tenimberensis*	Afrotropics	0	-0.0624	9.1921	3.18	0.7599	0.4109	70.91
Megapodiidae	*Talegalla cuvieri*	Neotropics	0	-0.6144	-	3.16	0.7750	0.3475	71.33
Megapodiidae	*Talegalla fuscirostris*	Neotropics	0	-0.0697	10.5619	3.58	0.7261	0.3019	12.47
Megapodiidae	*Talegalla jobiensis*	Neotropics	0	-0.1716	2.5500	3.93	0.8385	0.7355	28.09
Melanocharitidae	*Melanocharis arfakiana*	IndoMalay	0	-0.1564	-	3.22	0.8097	0.3962	26.45
Melanocharitidae	*Melanocharis crassirostris*	Australasia	0	-0.2282	-	3.13	0.8092	0.4459	52.55
Melanocharitidae	*Melanocharis longicauda*	Australasia	0	0.1190	5.6262	2.77	0.7340	0.1881	19.54
Melanocharitidae	*Melanocharis striativentris*	IndoMalay	0	0.0066	7.8323	2.68	0.7539	0.4029	85.72
Melanocharitidae	*Melanocharis versteri*	Australasia	0	-0.0706	4.8094	2.49	0.6824	0.2324	22.19
Melanocharitidae	*Oedistoma pygmaeum*	Palearctic	0	-0.8702	-	4.70	0.8632	0.2881	0.87
Melanocharitidae	*Oreocharis arfaki*	Neotropics	0	4.5783	14.5622	4.00	0.7727	0.2843	51.91
Melanocharitidae	*Paramythia montium*	IndoMalay	0	0.0462	-	2.63	0.7078	0.5114	56.92
Melanocharitidae	*Toxorhamphus iliolophus*	Neotropics	0	-4.9936	-	4.09	0.6478	0.6154	36.41
Melanocharitidae	*Toxorhamphus novaeguineae*	Neotropics	0	-0.1415	5.7172	3.85	0.8488	0.5002	37.65
Melanocharitidae	*Toxorhamphus poliopterus*	Neotropics	0	0.4282	5.9791	4.34	0.6961	0.2826	18.04
Meliphagidae	*Acanthagenys rufogularis*	Australasia	0	-0.6201	-	3.37	0.8319	0.4353	4.85
Meliphagidae	*Acanthorhynchus superciliosus*	Australasia	0	-0.8654	-	2.61	0.7245	0.5523	30.15
Meliphagidae	*Acanthorhynchus tenuirostris*	Australasia	0	-0.5485	-	2.96	0.7748	0.5322	73.34
Meliphagidae	*Anthochaera carunculata*	Australasia	0	-0.8295	-	2.98	0.8040	0.2093	5.87
Meliphagidae	*Anthochaera chrysoptera*	Australasia	0	-0.5691	3.0141	2.85	0.7704	0.5601	40.68
Meliphagidae	*Anthochaera lunulata*	Australasia	0	-0.9408	-	2.69	0.7340	0.5450	24.29
Meliphagidae	*Anthochaera paradoxa*	Australasia	0	-0.5529	-	2.18	0.6784	0.6178	18.26
Meliphagidae	*Anthornis melanura*	Australasia	0	-0.4736	-	2.33	0.7318	0.2222	43.08
Meliphagidae	*Ashbyia lovensis*	Australasia	0	-0.5601	-	3.36	0.8365	0.5679	10.98
Meliphagidae	*Certhionyx niger*	IndoMalay	0	0.1481	6.4765	3.94	0.7413	0.2034	6.21
Meliphagidae	*Certhionyx pectoralis*	Palearctic	0	-0.4205	7.4275	4.56	0.8664	0.2342	2.58
Meliphagidae	*Certhionyx variegatus*	Palearctic	0	-3.7283	-	3.97	0.7871	0.2639	15.70
Meliphagidae	*Conopophila albogularis*	Neotropics	0	0.0157	-	4.15	0.7172	0.1239	1.33
Meliphagidae	*Conopophila rufogularis*	Neotropics	0	-0.3238	4.0210	3.64	0.6948	0.3734	89.79
Meliphagidae	*Conopophila whitei*	Neotropics	0	0.0048	6.0755	3.66	0.7636	0.4258	79.75
Meliphagidae	*Entomyzon cyanotis*	Nearctic	0	-0.9078	10.5500	3.84	0.8136	0.2115	34.80
Meliphagidae	*Epthianura albifrons*	Palearctic	0	-0.9989	-	3.95	0.7808	0.8118	44.38
Meliphagidae	*Epthianura aurifrons*	Australasia	0	-0.5600	-	3.22	0.7865	0.4738	85.38

Family	Species	Realm	Threat	Latitude	Elevation	Anomaly	Size	Shape	Orientation
Meliphagidae	*Epthianura crocea*	Australasia	0	-0.2245	-	2.58	0.6302	0.1134	34.50
Meliphagidae	*Epthianura tricolor*	Australasia	0	-0.6343	-	2.43	0.6859	0.2947	69.41
Meliphagidae	*Foulehaio carunculatus*	Neotropics	0	-0.0513	3.1684	3.48	0.8003	0.6709	17.92
Meliphagidae	*Glycichaera fallax*	Afrotropics	0	0.0727	-	3.49	0.8356	0.5411	33.19
Meliphagidae	*Grantiella picta*	Neotropics	0	-58.0479	5.2162	4.14	0.6891	0.3616	31.41
Meliphagidae	*Guadalcanaria inexpectata*	Neotropics	0	-0.0881	12.6763	3.48	0.7542	0.2112	59.67
Meliphagidae	*Gymnomyza aubryana*	Neotropics	0	-0.1745	4.5954	3.61	0.7363	0.2278	29.09
Meliphagidae	*Gymnomyza viridis*	Palearctic	0	-0.8123	9.5781	4.32	0.7989	0.3444	6.07
Meliphagidae	*Lichenostomus chrysops*	Neotropics	0	0.1177	13.0672	3.79	0.7179	0.1743	62.11
Meliphagidae	*Lichenostomus cratitius*	Neotropics	0	-0.5109	-	2.76	0.8014	0.6526	69.66
Meliphagidae	*Lichenostomus fasciogularis*	Neotropics	0	-0.5847	6.5982	2.83	0.7556	0.4816	36.40
Meliphagidae	*Lichenostomus flavescens*	Neotropics	0	-0.0281	11.4447	4.01	0.7396	0.1546	46.40
Meliphagidae	*Lichenostomus flavicollis*	Neotropics	0	-0.4508	3.7932	2.73	0.7348	0.7728	79.05
Meliphagidae	*Lichenostomus flavus*	Neotropics	1	0.0000	5.3349	4.17	0.6221	0.6249	35.84
Meliphagidae	*Lichenostomus frenatus*	Neotropics	0	2.6796	10.3493	4.00	0.7165	0.1839	45.24
Meliphagidae	*Lichenostomus fuscus*	Neotropics	0	-0.1504	6.1056	3.85	0.8513	0.4674	39.90
Meliphagidae	*Lichenostomus hindwoodi*	Neotropics	1	-0.2328	1.9910	3.16	0.6478	0.3288	65.63
Meliphagidae	*Lichenostomus keartlandi*	Palearctic	0	4.0159	9.9635	4.26	0.8021	0.3595	13.86
Meliphagidae	*Lichenostomus leucotis*	Palearctic	0	3.7379	15.7953	4.26	0.7987	0.3335	15.46
Meliphagidae	*Lichenostomus melanops*	Neotropics	0	-0.1592	-	3.81	0.8418	0.4784	40.45
Meliphagidae	*Lichenostomus obscurus*	Neotropics	0	-1.1683	6.8770	3.50	0.7291	0.1894	60.84
Meliphagidae	*Lichenostomus ornatus*	Neotropics	0	-0.0064	8.8502	3.60	0.7828	0.4624	75.82
Meliphagidae	*Lichenostomus penicillatus*	Neotropics	0	-5.9471	8.4634	3.96	0.7217	0.1856	51.01
Meliphagidae	*Lichenostomus plumulus*	Afrotropics	0	-0.0705	-	3.18	0.7614	0.3874	70.15
Meliphagidae	*Lichenostomus subfrenatus*	Afrotropics	0	-0.0668	-	3.57	0.8538	0.7747	42.23
Meliphagidae	*Lichenostomus unicolor*	IndoMalay	1	-0.1856	-	3.21	0.7595	0.3818	55.92
Meliphagidae	*Lichenostomus versicolor*	IndoMalay	1	-0.0790	-	2.94	0.7946	0.4123	48.14
Meliphagidae	*Lichenostomus virescens*	Neotropics	1	-5.6297	6.0362	3.65	0.7277	0.2094	79.48
Meliphagidae	*Lichmera alboauricularis*	Afrotropics	0	-0.0754	6.6742	3.18	0.7614	0.3874	70.15
Meliphagidae	*Lichmera argentauris*	Neotropics	1	-0.2065	-	2.86	0.5788	0.0000	90.00
Meliphagidae	*Lichmera deningeri*	Neotropics	0	-0.1019	3.4563	3.32	0.7537	0.1821	33.65
Meliphagidae	*Lichmera flavicans*	Neotropics	1	4.2582	3.7741	3.18	0.6598	0.3474	70.32
Meliphagidae	*Lichmera incana*	Neotropics	0	-0.2931	-	2.88	0.7060	0.2828	2.89
Meliphagidae	*Lichmera indistincta*	Neotropics	0	5.3491	10.0673	3.78	0.7340	0.2830	83.12
Meliphagidae	*Lichmera limbata*	Neotropics	1	-0.0968	6.4143	3.18	0.7008	0.3236	84.15
Meliphagidae	*Lichmera lombokia*	Neotropics	0	0.3205	1.9807	3.00	0.7205	0.1650	64.92
Meliphagidae	*Lichmera monticola*	Neotropics	0	-0.0531	5.5579	3.47	0.7576	0.2328	34.98
Meliphagidae	*Lichmera squamata*	Neotropics	0	-0.2223	6.4180	3.85	0.8501	0.6468	37.76
Meliphagidae	*Macgregoria pulchra*	IndoMalay	1	0.1377	7.3508	2.65	0.6921	0.2403	44.20
Meliphagidae	*Manorina flavigula*	Australasia	0	0.0706	-	2.55	0.6562	0.1420	18.65
Meliphagidae	*Manorina melanocephala*	Australasia	0	-0.0491	6.8536	2.76	0.7660	0.2343	18.37
Meliphagidae	*Manorina melanophrys*	IndoMalay	0	-0.0925	10.2174	2.92	0.7997	0.4298	58.41
Meliphagidae	*Manorina melanotis*	IndoMalay	0	-0.0026	-	2.63	0.7335	0.3308	67.69
Meliphagidae	*Melidectes belfordi*	Neotropics	0	-0.4832	3.4075	2.65	0.7539	0.6736	26.31
Meliphagidae	*Melidectes foersteri*	Neotropics	0	-0.8184	5.6345	3.64	0.7087	0.2802	16.25
Meliphagidae	*Melidectes fuscus*	Neotropics	0	-0.0527	12.3178	3.86	0.7780	0.2258	38.05
Meliphagidae	*Melidectes leucostephes*	Neotropics	0	-0.0154	2.4373	4.07	0.8342	0.8553	4.75
Meliphagidae	*Melidectes nouhuysi*	Neotropics	0	0.0602	-	4.01	0.8030	0.7035	21.58
Meliphagidae	*Melidectes ochromelas*	Australasia	0	-0.1008	2.9236	2.78	0.7689	0.2984	24.32
Meliphagidae	*Melidectes princeps*	Neotropics	0	-0.0039	4.6003	3.75	0.7730	0.3634	57.01
Meliphagidae	*Melidectes rufocrissalis*	IndoMalay	0	-0.1062	-	3.05	0.7767	0.4159	34.54
Meliphagidae	*Melidectes sclateri*	IndoMalay	0	0.0214	-	2.84	0.7909	0.5777	12.46
Meliphagidae	*Melidectes torquatus*	IndoMalay	0	-0.0247	-	2.84	0.7855	0.5159	13.18
Meliphagidae	*Melidectes whitemanensis*	Afrotropics	1	0.1635	2.4388	3.10	0.7228	0.3951	17.65
Meliphagidae	*Melilestes megarhynchus*	Neotropics	0	-0.2462	-	3.58	0.8278	0.6952	57.68
Meliphagidae	*Meliphaga albilineata*	Nearctic	0	-0.8052	2.9488	4.14	0.8134	0.6820	8.29
Meliphagidae	*Meliphaga albonotata*	Neotropics	0	-0.0349	4.8269	3.01	0.6370	0.2651	32.26
Meliphagidae	*Meliphaga analoga*	Neotropics	0	-0.2871	6.0114	3.63	0.7314	0.2484	34.60
Meliphagidae	*Meliphaga aruensis*	Neotropics	0	0.0341	4.1961	4.04	0.8376	0.7826	8.57
Meliphagidae	*Meliphaga flavirictus*	Nearctic	0	-0.7050	-	4.30	0.8306	0.5962	14.72
Meliphagidae	*Meliphaga gracilis*	Neotropics	0	-0.1842	7.6976	3.33	0.7948	0.4906	38.54
Meliphagidae	*Meliphaga lewinii*	Nearctic	0	-0.3929	12.7417	3.72	0.8016	0.1452	37.65
Meliphagidae	*Meliphaga mimikae*	Neotropics	0	0.0412	5.0166	3.38	0.6935	0.3124	55.18
Meliphagidae	*Meliphaga montana*	Neotropics	0	0.7356	8.5100	3.64	0.7069	0.4283	22.63
Meliphagidae	*Meliphaga notata*	Nearctic	0	-0.6852	14.1485	4.07	0.8061	0.5686	41.75
Meliphagidae	*Meliphaga notata*	Nearctic	0	-0.6852	14.1485	4.07	0.8061	-	-
Meliphagidae	*Meliphaga orientalis*	Neotropics	0	0.7331	-	2.41	0.5788	0.0000	0.00
Meliphagidae	*Meliphaga reticulata*	Neotropics	0	-0.1530	-	3.29	0.7669	0.3264	46.45
Meliphagidae	*Melipotes ater*	Neotropics	0	-0.4262	5.5149	3.63	0.6891	0.8188	61.94
Meliphagidae	*Melipotes fumigatus*	Neotropics	0	-0.0467	-	2.87	0.7128	0.7338	55.28
Meliphagidae	*Melipotes gymnops*	Neotropics	0	-0.6763	6.7172	2.47	0.5985	0.0000	0.00
Meliphagidae	*Melithreptus affinis*	Neotropics	0	-0.2796	-	3.78	0.7780	0.3298	1.24
Meliphagidae	*Melithreptus albogularis*	Neotropics	0	-0.1005	-	2.46	0.6876	0.3855	3.42
Meliphagidae	*Melithreptus brevirostris*	Nearctic	0	-0.4412	-	3.86	0.7659	0.3418	53.03

Family	Species	Realm	Threat	Latitude	Elevation	Anomaly	Size	Shape	Orientation
Meliphagidae	*Melithreptus gularis*	Palearctic	0	-0.7731	-	5.00	0.8614	0.0573	0.20
Meliphagidae	*Melithreptus lunatus*	Palearctic	0	-0.5846	-	5.54	0.8442	0.0489	1.06
Meliphagidae	*Melithreptus validirostris*	Nearctic	0	-0.6213	-	4.36	0.8273	0.1178	7.29
Meliphagidae	*Melitograis gilolensis*	Australasia	0	0.0836	2.7243	2.77	0.6716	0.3539	29.65
Meliphagidae	*Myza celebensis*	IndoMalay	0	-0.7387	8.8229	2.61	0.6523	0.3873	66.90
Meliphagidae	*Myza sarasinorum*	IndoMalay	0	0.0331	-	2.76	0.7198	0.1025	54.23
Meliphagidae	*Myzomela adolphinae*	IndoMalay	0	0.1330	5.6685	2.84	0.6690	0.3320	77.01
Meliphagidae	*Myzomela blasii*	Neotropics	0	0.0396	11.0731	3.42	0.7422	0.2784	73.12
Meliphagidae	*Myzomela boiei*	Neotropics	0	0.0397	3.5263	4.06	0.8358	0.8133	7.54
Meliphagidae	*Myzomela caledonica*	Neotropics	0	0.0905	1.5170	2.92	0.6974	0.2262	66.89
Meliphagidae	*Myzomela cardinalis*	Neotropics	0	26.1843	4.9932	3.78	0.7060	0.5433	8.96
Meliphagidae	*Myzomela chloroptera*	Neotropics	0	0.2107	-	3.78	0.6859	0.3691	59.83
Meliphagidae	*Myzomela cruentata*	Neotropics	0	0.3878	-	3.17	0.7572	0.5194	54.05
Meliphagidae	*Myzomela dammermani*	Neotropics	0	-0.0512	-	4.01	0.7832	0.8310	84.24
Meliphagidae	*Myzomela eques*	Neotropics	0	0.1518	-	3.98	0.8016	0.7997	30.32
Meliphagidae	*Myzomela erythrocephala*	Neotropics	0	-0.0250	-	4.21	0.7505	0.9188	21.16
Meliphagidae	*Myzomela erythromelas*	Neotropics	1	0.0071	11.3131	3.28	0.6740	0.4805	85.73
Meliphagidae	*Myzomela jugularis*	Neotropics	0	0.1560	4.4257	4.12	0.8109	0.5120	8.29
Meliphagidae	*Myzomela lafargei*	Neotropics	0	0.2212	-	4.07	0.7971	0.8221	44.86
Meliphagidae	*Myzomela melanocephala*	Neotropics	0	0.1139	-	3.23	0.7517	0.7377	55.38
Meliphagidae	*Myzomela nigrita*	Neotropics	0	-0.5704	-	3.37	0.7340	0.2639	11.86
Meliphagidae	*Myzomela obscura*	Neotropics	0	-0.0738	-	3.94	0.7945	0.3556	20.82
Meliphagidae	*Myzomela pulchella*	Neotropics	0	-0.1489	3.4260	3.12	0.7323	0.2376	71.10
Meliphagidae	*Myzomela rosenbergii*	Neotropics	0	0.1804	-	3.85	0.7903	0.8603	78.71
Meliphagidae	*Myzomela sanguinolenta*	Neotropics	0	0.1635	5.5030	3.01	0.7069	0.1627	68.66
Meliphagidae	*Myzomela sclateri*	Neotropics	0	0.0601	-	3.73	0.6935	0.8874	41.25
Meliphagidae	*Myzomela tristrami*	Neotropics	1	-0.0294	1.9374	3.10	0.7205	0.1658	65.44
Meliphagidae	*Myzomela vulnerata*	Neotropics	0	-0.5614	4.3944	2.67	0.7389	-	-
Meliphagidae	*Myzomela vulnerata*	Neotropics	0	-0.5614	4.3944	2.67	0.7389	0.4196	44.02
Meliphagidae	*Myzomela wakoloensis*	Afrotropics	0	-0.0076	9.0405	3.68	0.8078	0.2088	9.15
Meliphagidae	*Oreornis chrysogenys*	Australasia	0	-0.5672	-	3.36	0.8371	0.5441	7.16
Meliphagidae	*Philemon argenticeps*	Neotropics	0	5.1386	8.2827	3.72	0.7261	0.1158	78.48
Meliphagidae	*Philemon brassi*	Neotropics	0	-0.3171	5.6271	3.84	0.8126	0.6097	27.67
Meliphagidae	*Philemon buceroides*	Neotropics	0	-0.1438	-	3.77	0.8153	0.7044	27.88
Meliphagidae	*Philemon citreogularis*	Neotropics	0	-0.7148	-	3.05	0.7815	0.5556	89.48
Meliphagidae	*Philemon cockerelli*	Neotropics	0	3.9533	13.4178	4.01	0.7522	0.3501	66.58
Meliphagidae	*Philemon corniculatus*	Neotropics	0	-0.7044	1.2244	2.50	0.7710	0.5092	12.62
Meliphagidae	*Philemon diemenensis*	Australasia	0	0.0069	4.1783	2.71	0.7185	0.5623	60.71
Meliphagidae	*Philemon eichhorni*	IndoMalay	0	0.0139	-	2.84	0.7897	0.5262	11.37
Meliphagidae	*Philemon fuscicapillus*	IndoMalay	0	-0.0398	-	2.66	0.6859	0.4375	72.70
Meliphagidae	*Philemon inornatus*	IndoMalay	0	-0.0286	-	2.82	0.7900	0.6330	17.37
Meliphagidae	*Philemon meyeri*	IndoMalay	0	-0.0087	-	2.84	0.7884	0.4875	7.46
Meliphagidae	*Philemon moluccensis*	IndoMalay	0	-0.0343	-	2.83	0.7922	0.6112	15.58
Meliphagidae	*Philemon novaeguineae*	IndoMalay	0	-0.1011	-	3.40	0.8097	0.8672	86.23
Meliphagidae	*Philemon subcorniculatus*	IndoMalay	1	0.2619	3.3666	2.76	0.6690	0.8248	84.45
Meliphagidae	*Phylidonyris albifrons*	Neotropics	0	-0.0048	9.7974	3.68	0.7721	0.4230	75.32
Meliphagidae	*Phylidonyris melanops*	Neotropics	0	-0.0164	10.3080	3.65	0.7756	0.4032	80.99
Meliphagidae	*Phylidonyris niger*	Neotropics	0	1.1080	5.8880	3.68	0.6824	0.0803	2.77
Meliphagidae	*Phylidonyris notabilis*	Neotropics	0	0.0356	9.5633	3.48	0.7389	0.2943	32.76
Meliphagidae	*Phylidonyris novaehollandiae*	Neotropics	0	0.3027	-	4.08	0.8199	0.7215	4.91
Meliphagidae	*Phylidonyris pyrrhopterus*	Palearctic	0	-0.5365	-	3.98	0.8623	0.1433	2.98
Meliphagidae	*Phylidonyris undulatus*	Afrotropics	0	-0.0786	7.9979	3.18	0.7614	0.3874	70.15
Meliphagidae	*Plectorhyncha lanceolata*	Neotropics	0	-4.8480	6.2284	3.32	0.6948	0.1441	68.76
Meliphagidae	*Prosthemadera novaeseelandiae*	Neotropics	0	4.5439	9.0208	3.77	0.7485	0.2162	83.20
Meliphagidae	*Ptiloprora erythropleura*	Australasia	0	-0.0595	12.6805	2.81	0.7385	0.1419	24.66
Meliphagidae	*Ptiloprora guisei*	Australasia	0	-0.1025	7.6385	2.85	0.7422	0.1812	23.60
Meliphagidae	*Ptiloprora mayri*	Australasia	0	-0.0745	6.4366	2.84	0.7234	0.1551	21.79
Meliphagidae	*Ptiloprora meekiana*	Australasia	0	-0.1754	10.2083	2.85	0.7331	0.1615	20.87
Meliphagidae	*Ptiloprora perstriata*	IndoMalay	0	-0.0854	-	3.17	0.8111	0.4547	48.88
Meliphagidae	*Ptiloprora plumbea*	IndoMalay	0	0.3328	4.6479	2.75	0.6562	0.7113	15.53
Meliphagidae	*Pycnopygius cinereus*	Australasia	0	-0.6327	0.9981	2.49	0.7409	0.0926	13.66
Meliphagidae	*Pycnopygius ixoides*	Australasia	0	-0.5309	-	2.13	0.7680	0.0448	2.65
Meliphagidae	*Pycnopygius stictocephalus*	Australasia	1	-0.3174	0.0000	2.63	0.7192	0.3280	4.09
Meliphagidae	*Ramsayornis fasciatus*	IndoMalay	0	-0.1514	-	3.07	0.8197	0.5762	85.69
Meliphagidae	*Ramsayornis modestus*	Afrotropics	0	-0.2319	11.6884	3.56	0.8637	0.6510	34.09
Meliphagidae	*Stresemannia bougainvillei*	IndoMalay	0	0.3276	5.6366	3.85	0.7104	0.2093	11.48
Meliphagidae	*Timeliopsis fulvigula*	Afrotropics	0	-0.1004	11.8124	3.55	0.7765	0.7009	41.95
Meliphagidae	*Timeliopsis griseigula*	Afrotropics	0	-0.4639	0.0000	3.31	0.7574	0.2797	59.67
Meliphagidae	*Trichodere cockerelli*	Neotropics	0	0.0493	13.5974	3.36	0.7234	0.1737	62.40
Meliphagidae	*Xanthomyza phrygia*	Afrotropics	0	0.1240	8.1105	3.57	0.8223	0.1673	4.79
Meliphagidae	*Xanthotis flaviventer*	Afrotropics	0	0.0114	-	3.24	0.8124	0.2441	12.30
Meliphagidae	*Xanthotis macleayanus*	Afrotropics	0	-0.1688	-	3.57	0.8330	0.4703	58.09
Meliphagidae	*Xanthotis polygrammus*	Afrotropics	0	-0.1038	12.7816	3.36	0.8419	0.5428	29.81

Family	Species	Realm	Threat	Latitude	Elevation	Anomaly	Size	Shape	Orientation
Menuridae	*Menura alberti*	Neotropics	0	-0.2733	5.5330	3.21	0.7087	0.1859	82.47
Menuridae	*Menura novaehollandiae*	Neotropics	0	9.4033	3.0376	3.48	0.6523	0.7303	90.00
Meropidae	*Meropogon forsteni*	Neotropics	0	-0.1720	-	3.80	0.7676	0.3011	39.65
Meropidae	*Merops albicollis*	Neotropics	0	-0.0398	11.0743	3.62	0.7205	0.3000	21.32
Meropidae	*Merops apiaster*	Nearctic	0	-0.6370	9.1087	4.18	0.8394	0.2326	14.88
Meropidae	*Merops boehmi*	Neotropics	0	-0.1962	0.5279	3.07	0.7240	0.5321	58.36
Meropidae	*Merops breweri*	Afrotropics	0	0.0028	-	3.29	0.8118	0.3401	11.79
Meropidae	*Merops bullockoides*	Australasia	0	-0.0452	13.4623	2.84	0.7348	0.1587	24.37
Meropidae	*Merops bulocki*	Australasia	0	-0.1396	11.9958	2.78	0.6428	0.2952	21.65
Meropidae	*Merops gularis*	Australasia	0	-0.2564	8.9949	2.85	0.7245	0.1578	22.35
Meropidae	*Merops hirundineus*	Australasia	0	-0.4149	4.6565	2.51	0.6478	0.6630	83.13
Meropidae	*Merops leschenaulti*	Australasia	0	0.0000	8.9630	2.88	0.6690	0.2147	15.90
Meropidae	*Merops malimbicus*	Australasia	0	-0.0846	-	2.69	0.7185	0.1251	24.46
Meropidae	*Merops mentalis*	Australasia	1	0.0000	4.9258	2.94	0.6478	0.5344	13.51
Meropidae	*Merops muelleri*	Australasia	0	-0.1993	-	2.94	0.7112	0.2565	22.06
Meropidae	*Merops nubicus*	Australasia	0	-0.0635	-	2.81	0.7432	0.1584	23.67
Meropidae	*Merops oreobates*	Australasia	0	0.4950	3.6664	2.49	0.6631	0.3432	20.37
Meropidae	*Merops orientalis*	Australasia	0	-0.0810	-	2.78	0.7702	0.2980	24.16
Meropidae	*Merops ornatus*	Afrotropics	0	-0.2654	10.6375	4.01	0.8051	0.7861	49.74
Meropidae	*Merops persicus*	Afrotropics	0	-0.1157	8.3076	3.63	0.8558	0.7731	46.12
Meropidae	*Merops philippinus*	Afrotropics	0	-0.2086	11.8424	3.64	0.8527	0.6613	37.17
Meropidae	*Merops pusillus*	Afrotropics	0	0.0178	7.6959	3.31	0.8025	0.4257	51.37
Meropidae	*Merops revoilii*	Afrotropics	0	0.2044	3.0208	3.19	0.6784	0.0564	8.09
Meropidae	*Merops superciliosus*	Afrotropics	0	0.2126	-	3.27	0.7911	0.3200	5.03
Meropidae	*Merops variegatus*	Australasia	0	-0.0796	-	2.79	0.7670	0.2383	24.04
Meropidae	*Merops viridis*	Australasia	0	0.1182	0.0000	3.20	0.7128	0.1592	16.35
Meropidae	*Nyctyornis amictus*	Australasia	0	-0.5886	5.3227	3.10	0.7673	0.4077	63.40
Meropidae	*Nyctyornis athertoni*	Australasia	0	-0.0517	2.0887	2.60	0.5985	0.2277	28.15
Mesitornithidae	*Mesitornis unicolor*	Australasia	0	-0.0631	-	2.80	0.7631	0.2509	24.38
Mesitornithidae	*Mesitornis variegatus*	Australasia	0	-0.1639	-	2.81	0.7493	0.6103	51.98
Mesitornithidae	*Monias benschi*	Afrotropics	0	-0.1682	-	4.17	0.8052	0.7970	19.87
Mimidae	*Dumetella carolinensis*	Australasia	0	0.1338	4.8505	2.50	0.6716	0.2680	24.42
Mimidae	*Margarops fuscatus*	Afrotropics	1	-0.2397	1.4153	3.35	0.6842	0.1896	81.45
Mimidae	*Melanoptila glabrirostris*	Nearctic	0	-0.6057	10.1241	3.94	0.8201	0.2822	47.19
Mimidae	*Melanotis caerulescens*	Neotropics	0	1.1220	11.7744	3.86	0.6120	0.1672	58.57
Mimidae	*Melanotis hypoleucus*	Neotropics	0	-1.4401	2.2209	4.06	0.6986	0.1038	32.66
Mimidae	*Mimus dorsalis*	Afrotropics	0	-0.0353	6.3999	3.41	0.8258	0.8777	59.95
Mimidae	*Mimus gilvus*	Afrotropics	0	0.0657	17.0885	3.43	0.8251	0.6737	30.67
Mimidae	*Mimus gundlachii*	IndoMalay	0	-0.2901	-	2.96	0.8137	0.5758	80.13
Mimidae	*Mimus longicaudatus*	Neotropics	0	-0.3272	5.6604	2.92	0.7340	0.1896	43.27
Mimidae	*Mimus parvulus*	Neotropics	1	1.1118	2.0038	3.21	0.5985	0.2326	29.21
Mimidae	*Mimus patagonicus*	Neotropics	0	-0.1894	-	3.92	0.8479	0.5326	42.56
Mimidae	*Mimus polyglottos*	Afrotropics	1	-0.1312	5.0440	3.03	0.7104	0.1068	72.68
Mimidae	*Mimus saturninus*	Afrotropics	1	0.0330	1.3139	3.19	0.7040	0.2714	59.25
Mimidae	*Mimus thenca*	Afrotropics	0	-0.3609	4.7798	3.43	0.8723	0.3818	2.65
Mimidae	*Mimus triurus*	Afrotropics	0	0.0451	10.1459	3.32	0.7823	0.6413	4.29
Mimidae	*Oreoscoptes montanus*	Neotropics	0	-3.5049	6.0009	4.08	0.7228	0.1695	32.04
Mimidae	*Toxostoma bendirei*	Neotropics	0	-0.1326	9.4720	3.33	0.7286	0.3573	59.73
Mimidae	*Toxostoma cinereum*	Neotropics	0	0.0542	-	4.04	0.8228	0.5652	29.05
Mimidae	*Toxostoma crissale*	Neotropics	0	-0.1668	-	3.38	0.7359	0.9650	77.12
Mimidae	*Toxostoma curvirostre*	Neotropics	0	0.0756	-	4.03	0.8038	0.5501	28.76
Mimidae	*Toxostoma lecontei*	Neotropics	0	-0.1855	6.3284	3.92	0.8109	0.4979	8.56
Mimidae	*Toxostoma longirostre*	Neotropics	0	-0.1996	3.8163	3.79	0.8095	0.4306	42.98
Mimidae	*Toxostoma redivivum*	Neotropics	0	0.0000	0.3197	3.58	0.6478	0.7196	11.98
Mimidae	*Toxostoma rufum*	Neotropics	0	-0.0358	3.6338	4.07	0.7964	0.5406	12.12
Momotidae	*Aspatha gularis*	Palearctic	0	-0.5419	-	4.52	0.8831	0.3032	7.11
Momotidae	*Baryphthengus martii*	Neotropics	0	-0.7347	6.4797	2.98	0.6784	0.1674	57.16
Momotidae	*Baryphthengus ruficapillus*	Neotropics	1	0.2089	7.1319	3.08	0.6716	0.2616	77.49
Momotidae	*Electron carinatum*	Afrotropics	1	0.0332	-	4.24	0.7179	0.1974	0.66
Momotidae	*Electron platyrhynchum*	Neotropics	0	-0.3865	11.0026	3.16	0.8086	0.2248	82.55
Momotidae	*Eumomota superciliosa*	Neotropics	0	0.0801	9.0921	3.27	0.7560	0.7294	69.73
Momotidae	*Hylomanes momotula*	Afrotropics	0	0.0008	11.2390	3.50	0.8536	0.5601	29.91
Momotidae	*Momotus mexicanus*	Neotropics	0	-0.2070	-	3.78	0.8539	0.6654	46.52
Momotidae	*Momotus momota*	Neotropics	0	-0.6048	-	2.66	0.8174	0.4588	58.17
Monarchidae	*Arses insularis*	Neotropics	0	-0.2804	-	3.35	0.7367	0.7589	51.41
Monarchidae	*Arses kaupi*	Neotropics	0	0.0035	-	3.70	0.7899	0.5473	3.40
Monarchidae	*Arses telescophthalmus*	Neotropics	0	-0.1722	-	3.43	0.7656	0.4668	25.33
Monarchidae	*Chasiempis sandwichensis*	Nearctic	0	-0.6230	7.4851	4.12	0.8561	0.6252	30.34
Monarchidae	*Clytorhynchus nigrogularis*	Afrotropics	0	-0.1229	8.2106	3.60	0.8466	0.5560	35.58
Monarchidae	*Clytorhynchus pachycephaloides*	Nearctic	0	-0.6818	-	5.49	0.8476	0.0676	1.16
Monarchidae	*Clytorhynchus vitiensis*	Neotropics	1	-0.3346	8.0298	2.96	0.7472	0.2489	29.14
Monarchidae	*Elminia albicauda*	Neotropics	0	-0.1760	-	3.74	0.8478	0.4807	38.11
Monarchidae	*Elminia albiventris*	Neotropics	0	-0.1254	13.7875	3.48	0.7606	0.4649	20.89

Family	Species	Realm	Threat	Latitude	Elevation	Anomaly	Size	Shape	Orientation
Monarchidae	*Elminia albonotata*	Neotropics	0	-3.7217	4.8115	3.84	0.7674	0.3544	63.44
Monarchidae	*Elminia longicauda*	Neotropics	0	-0.0562	-	2.59	0.6478	0.0892	3.12
Monarchidae	*Elminia nigromitrata*	Neotropics	0	-0.3898	8.7837	3.36	0.8018	0.4115	41.15
Monarchidae	*Erythrocercus holochlorus*	Afrotropics	0	-0.3896	-	3.87	0.7683	0.4506	44.56
Monarchidae	*Erythrocercus livingstonei*	IndoMalay	0	-0.1019	3.0680	3.45	0.8134	0.8810	51.64
Monarchidae	*Erythrocercus mccallii*	Afrotropics	0	0.1311	-	3.31	0.7824	0.5896	80.66
Monarchidae	*Grallina bruijni*	Neotropics	1	-0.1200	4.8066	3.25	0.6221	0.5865	19.14
Monarchidae	*Grallina cyanoleuca*	Neotropics	0	-14.1691	6.1327	3.80	0.6631	0.1753	66.16
Monarchidae	*Hypothymis azurea*	Neotropics	0	-0.0100	-	4.13	0.8080	0.5182	77.47
Monarchidae	*Hypothymis coelestis*	Neotropics	0	0.0066	-	3.93	0.8336	0.5748	24.90
Monarchidae	*Hypothymis helenae*	Neotropics	0	-16.1749	5.3208	3.66	0.7211	0.2227	74.11
Monarchidae	*Monarcha axillaris*	Palearctic	0	-0.6310	2.3787	3.66	0.8077	0.3925	10.97
Monarchidae	*Monarcha barbatus*	IndoMalay	1	-0.0628	1.9091	2.62	0.6997	0.6660	83.57
Monarchidae	*Monarcha castaneiventris*	Neotropics	0	-3.4656	7.3691	4.03	0.7256	0.2284	82.22
Monarchidae	*Monarcha castus*	Neotropics	0	-0.1152	-	3.66	0.8069	0.2418	27.78
Monarchidae	*Monarcha cinerascens*	Neotropics	0	-0.0973	-	3.45	0.7382	0.1467	63.85
Monarchidae	*Monarcha frater*	Neotropics	0	-0.6681	6.4655	2.64	0.7920	0.3015	76.38
Monarchidae	*Monarcha guttula*	Nearctic	0	-0.6666	-	4.12	0.8466	0.5710	1.03
Monarchidae	*Monarcha leucotis*	Neotropics	0	-0.3329	-	3.43	0.8373	0.4788	51.05
Monarchidae	*Monarcha loricatus*	Neotropics	0	-0.6161	-	2.79	0.7179	0.1018	79.01
Monarchidae	*Monarcha manadensis*	Neotropics	0	-0.5323	-	2.74	0.7790	0.5995	65.20
Monarchidae	*Monarcha melanopsis*	IndoMalay	0	-0.1029	-	3.32	0.8138	0.8634	33.84
Monarchidae	*Monarcha mundus*	IndoMalay	0	-0.2079	11.9061	3.40	0.7926	0.5335	4.46
Monarchidae	*Monarcha pileatus*	IndoMalay	0	-0.0046	-	3.51	0.7853	0.5321	26.73
Monarchidae	*Monarcha rubiensis*	Australasia	0	-0.1031	-	2.77	0.7614	0.2943	23.05
Monarchidae	*Monarcha sacerdotum*	Australasia	0	-0.0888	-	2.76	0.7730	0.3111	16.92
Monarchidae	*Monarcha trivirgatus*	Neotropics	0	0.0156	-	4.06	0.8135	0.7326	34.91
Monarchidae	*Monarcha verticalis*	Neotropics	0	-0.1217	-	3.91	0.8441	0.4994	32.22
Monarchidae	*Monarcha viduus*	Neotropics	0	0.0362	11.0213	3.56	0.7834	0.5065	89.86
Monarchidae	*Myiagra alecto*	Neotropics	0	-0.1829	-	3.92	0.8447	0.6099	29.85
Monarchidae	*Myiagra azureocapilla*	Afrotropics	0	-0.1259	12.2605	3.48	0.8512	0.7015	36.65
Monarchidae	*Myiagra caledonica*	Palearctic	0	-0.7649	10.4295	4.62	0.8810	0.2655	1.06
Monarchidae	*Myiagra cervinicauda*	Afrotropics	0	-0.1383	-	3.82	0.8222	0.6097	77.39
Monarchidae	*Myiagra cyanoleuca*	Palearctic	0	-0.8977	-	4.48	0.8645	0.2070	3.79
Monarchidae	*Myiagra ferrocyanea*	Palearctic	0	-0.7277	9.8450	4.92	0.8610	0.4793	7.11
Monarchidae	*Myiagra galeata*	Afrotropics	0	-0.0519	3.0708	3.49	0.8182	0.9260	39.18
Monarchidae	*Myiagra hebetior*	Palearctic	0	-0.8719	10.5535	4.76	0.8744	0.1823	2.42
Monarchidae	*Myiagra inquieta*	Afrotropics	0	-0.0675	8.6640	3.18	0.7614	0.3874	70.15
Monarchidae	*Myiagra rubecula*	Palearctic	0	-0.8883	4.0352	3.28	0.7507	0.3626	28.19
Monarchidae	*Myiagra ruficollis*	IndoMalay	0	-0.1549	-	3.45	0.8117	0.9281	57.24
Monarchidae	*Myiagra vanikorensis*	IndoMalay	0	0.0841	-	3.04	0.6804	0.6055	79.48
Monarchidae	*Neolalage banksiana*	Afrotropics	0	-0.1258	-	3.06	0.7008	0.1368	44.50
Monarchidae	*Terpsiphone atrocaudata*	Neotropics	1	0.4093	2.4173	3.11	0.6631	0.3196	84.03
Monarchidae	*Terpsiphone bedfordi*	Neotropics	0	0.0585	13.1523	3.52	0.7563	0.2901	83.35
Monarchidae	*Terpsiphone cinnamomea*	Neotropics	1	9.5057	2.7183	3.43	0.6370	0.2746	86.08
Monarchidae	*Terpsiphone cyanescens*	Neotropics	1	0.7760	6.3566	3.33	0.5788	0.0000	90.00
Monarchidae	*Terpsiphone mutata*	Neotropics	0	-0.3947	5.4070	3.90	0.7923	0.8768	46.86
Monarchidae	*Terpsiphone paradisi*	Neotropics	0	-0.4171	6.7346	3.76	0.6598	0.1152	0.51
Monarchidae	*Terpsiphone rufocinerea*	Neotropics	0	0.7085	7.8739	4.33	0.7172	0.3229	5.07
Monarchidae	*Terpsiphone viridis*	Neotropics	1	-0.2978	7.3802	3.25	0.6876	0.4266	84.49
Monarchidae	*Trochocercus cyanomelas*	Nearctic	0	-0.5982	6.1939	3.99	0.8156	0.5986	34.83
Monarchidae	*Trochocercus nitens*	Neotropics	0	-0.0299	2.6590	3.41	0.7678	0.3547	32.36
Motacillidae	*Anthus bogotensis*	Neotropics	0	-0.1478	13.5016	3.70	0.7629	0.3745	76.14
Motacillidae	*Anthus brachyurus*	Afrotropics	0	-0.1646	-	3.56	0.7968	0.5639	50.75
Motacillidae	*Anthus caffer*	Afrotropics	0	0.1135	-	3.70	0.7803	0.6885	79.47
Motacillidae	*Anthus campestris*	Palearctic	0	-0.7966	-	4.31	0.8520	0.2121	3.62
Motacillidae	*Anthus cervinus*	Palearctic	0	-0.7644	-	6.13	0.8228	0.0578	0.49
Motacillidae	*Anthus chacoensis*	Neotropics	0	-0.5999	0.0000	2.72	0.7700	0.4098	69.02
Motacillidae	*Anthus chloris*	Afrotropics	1	-2.5194	5.8990	3.35	0.6824	0.2372	60.87
Motacillidae	*Anthus correndera*	Neotropics	0	-0.4087	10.2310	2.90	0.8195	0.5446	87.44
Motacillidae	*Anthus crenatus*	Afrotropics	0	4.7202	11.1903	3.67	0.7480	0.4782	23.12
Motacillidae	*Anthus furcatus*	Neotropics	0	-0.3110	-	2.97	0.8031	0.4883	65.43
Motacillidae	*Anthus godlewskii*	Palearctic	0	-0.6735	13.4992	4.40	0.8078	0.3177	7.82
Motacillidae	*Anthus gustavi*	Palearctic	0	-0.6586	-	5.68	0.8213	0.0956	0.96
Motacillidae	*Anthus gutturalis*	Australasia	0	-0.3073	8.0696	2.85	0.7228	0.1643	21.83
Motacillidae	*Anthus hellmayri*	Neotropics	0	-0.5697	10.5355	2.86	0.7908	0.5209	38.99
Motacillidae	*Anthus hodgsoni*	Palearctic	0	-0.9503	-	4.64	0.8560	0.5382	7.10
Motacillidae	*Anthus hoeschi*	Afrotropics	0	12.6846	4.2503	3.51	0.6804	0.5640	47.30
Motacillidae	*Anthus leucophrys*	Afrotropics	0	-0.1547	-	3.57	0.8457	0.6826	36.92
Motacillidae	*Anthus lineiventris*	Afrotropics	0	-0.1621	-	3.64	0.8009	0.8408	61.78
Motacillidae	*Anthus longicaudatus*	Afrotropics	0	3.9516	-	4.05	0.6824	0.6126	79.85
Motacillidae	*Anthus lutescens*	Neotropics	0	-0.2433	-	3.53	0.8385	0.7688	83.47
Motacillidae	*Anthus melindae*	Afrotropics	0	0.0700	-	3.07	0.7144	0.1315	45.12
Motacillidae	*Anthus nattereri*	Neotropics	1	-0.5115	3.0976	2.94	0.7695	0.3751	34.96

Family	Species	Realm	Threat	Latitude	Elevation	Anomaly	Size	Shape	Orientation
Motacillidae	*Anthus nilghiriensis*	IndoMalay	1	0.0040	7.1212	3.01	0.6824	0.3805	66.51
Motacillidae	*Anthus novaeseelandiae*	Australasia	0	-0.4963	-	3.28	0.8356	0.4778	12.65
Motacillidae	*Anthus pallidiventris*	Afrotropics	0	-0.0797	-	3.24	0.7765	0.5367	63.59
Motacillidae	*Anthus petrosus*	Palearctic	0	-0.5022	1.0667	3.94	0.7663	0.2247	23.07
Motacillidae	*Anthus pratensis*	Palearctic	0	-0.6415	7.5781	4.67	0.8354	0.2904	8.32
Motacillidae	*Anthus richardi*	Palearctic	0	-0.5340	6.9615	4.06	0.8680	0.2183	34.49
Motacillidae	*Anthus roseatus*	Palearctic	0	-0.8883	0.0000	4.15	0.8283	0.3712	1.83
Motacillidae	*Anthus rubescens*	Nearctic	0	-0.6843	7.4669	4.72	0.8535	0.0640	0.64
Motacillidae	*Anthus rufulus*	IndoMalay	0	-0.0706	5.8836	3.31	0.8389	0.3879	29.36
Motacillidae	*Anthus similis*	Afrotropics	0	-0.1832	10.5078	3.70	0.8516	0.5170	40.38
Motacillidae	*Anthus sokokensis*	Afrotropics	1	0.0070	-	2.95	0.6370	0.1415	78.69
Motacillidae	*Anthus spinoletta*	Palearctic	0	-1.2491	12.7724	4.15	0.8268	0.1339	1.96
Motacillidae	*Anthus spragueii*	Nearctic	1	2.1744	11.4327	4.32	0.7892	0.4683	13.10
Motacillidae	*Anthus sylvanus*	IndoMalay	0	0.0775	9.6294	3.48	0.7712	0.1414	7.43
Motacillidae	*Anthus sylvanus*	IndoMalay	0	0.0775	9.6294	3.48	0.7712	-	-
Motacillidae	*Anthus trivialis*	Palearctic	0	-0.6182	8.8819	4.78	0.8584	0.2084	3.73
Motacillidae	*Anthus vaalensis*	Afrotropics	0	-0.2651	-	3.89	0.8201	0.7642	88.16
Motacillidae	*Dendronanthus indicus*	Nearctic	1	23.6921	-2.8344	4.10	0.6740	0.5014	56.82
Motacillidae	*Macronyx ameliae*	Australasia	1	-0.0064	3.2645	2.48	0.6302	0.2423	87.23
Motacillidae	*Macronyx aurantiigula*	Australasia	0	-0.1663	4.7165	2.52	0.6906	0.3582	59.75
Motacillidae	*Macronyx capensis*	Australasia	0	-0.0788	-	2.78	0.7707	0.3039	24.02
Motacillidae	*Macronyx croceus*	Palearctic	0	-0.7934	15.2080	4.47	0.8721	0.1145	1.21
Motacillidae	*Macronyx flavicollis*	Nearctic	0	-0.5860	-	4.79	0.8599	0.0568	0.75
Motacillidae	*Macronyx fuelleborni*	Neotropics	1	-0.0099	7.2632	2.47	0.6478	0.4894	25.81
Motacillidae	*Macronyx grimwoodi*	Palearctic	0	-0.3059	-	5.08	0.8167	0.2678	3.40
Motacillidae	*Macronyx sharpei*	Palearctic	0	-0.0659	0.0000	2.01	0.6523	0.5202	13.88
Motacillidae	*Motacilla aguimp*	Afrotropics	0	-0.1542	-	4.01	0.8096	0.6518	27.42
Motacillidae	*Motacilla alba*	Afrotropics	0	-0.0221	-	3.28	0.7281	0.5635	72.47
Motacillidae	*Motacilla capensis*	Afrotropics	0	15.3841	3.7563	3.29	0.6804	0.4383	87.51
Motacillidae	*Motacilla cinerea*	Neotropics	0	-4.1932	5.5804	3.96	0.7277	0.1754	41.79
Motacillidae	*Motacilla cinerea*	Neotropics	0	-4.1932	5.5804	3.96	0.7277	-	-
Motacillidae	*Motacilla citreola*	Neotropics	0	-0.0117	14.8973	3.60	0.7791	0.1483	33.76
Motacillidae	*Motacilla clara*	Neotropics	0	0.1770	-	3.06	0.7367	0.7049	77.36
Motacillidae	*Motacilla flava*	Neotropics	0	-8.9845	5.1219	4.39	0.6824	0.5412	11.00
Motacillidae	*Motacilla grandis*	Neotropics	0	0.1173	2.4151	3.67	0.7614	0.7788	36.74
Motacillidae	*Motacilla madaraspatensis*	Neotropics	0	0.1484	1.6894	4.03	0.7897	0.5532	2.87
Motacillidae	*Motacilla samveasnae*	Neotropics	0	0.0817	2.7888	4.11	0.8213	0.4819	7.58
Motacillidae	*Tmetothylacus tenellus*	Neotropics	0	0.0946	8.8436	3.14	0.7310	0.4321	75.05
Muscicapidae	*Bradornis infuscatus*	Afrotropics	1	-0.1080	4.2102	3.02	0.7050	0.0995	72.00
Muscicapidae	*Bradornis mariquensis*	IndoMalay	0	0.2482	11.4888	3.60	0.6784	0.1676	4.39
Muscicapidae	*Bradornis microrhynchus*	IndoMalay	0	-0.1383	7.3586	3.18	0.8122	0.6902	73.10
Muscicapidae	*Bradornis pallidus*	IndoMalay	0	-0.2222	-	3.27	0.8199	0.6988	68.40
Muscicapidae	*Cercomela dubia*	Afrotropics	0	-0.0488	-	3.26	0.8076	0.3044	12.04
Muscicapidae	*Cercomela familiaris*	Afrotropics	0	-0.1066	3.2031	3.14	0.7643	0.1663	8.51
Muscicapidae	*Cercomela fusca*	Neotropics	0	-0.0556	1.4014	4.15	0.7569	0.5381	10.44
Muscicapidae	*Cercomela melanura*	Afrotropics	0	-0.0139	-	3.28	0.7957	0.2222	13.22
Muscicapidae	*Cercomela schlegelii*	Afrotropics	0	-0.1193	-	3.33	0.7778	0.4539	84.48
Muscicapidae	*Cercomela scotocerca*	Afrotropics	0	-0.0106	-	3.25	0.8016	0.2479	14.31
Muscicapidae	*Cercomela sinuata*	Neotropics	0	-0.0120	2.0218	3.09	0.7240	0.2060	67.11
Muscicapidae	*Cercomela sordida*	Neotropics	0	0.1269	0.6188	4.38	0.6562	0.4298	64.74
Muscicapidae	*Cercomela tractrac*	Neotropics	0	-0.0374	-	4.04	0.8355	0.7445	0.51
Muscicapidae	*Cercotrichas podobe*	Neotropics	1	0.1346	-	4.07	0.7060	0.2223	69.64
Muscicapidae	*Chaimarrornis leucocephalus*	Neotropics	1	0.0851	0.6317	2.96	0.6120	0.1357	57.87
Muscicapidae	*Cichladusa arquata*	Australasia	0	-0.1784	0.0000	2.92	0.8067	0.2845	32.64
Muscicapidae	*Cichladusa guttata*	IndoMalay	0	-0.1023	-	2.94	0.8135	0.5598	53.59
Muscicapidae	*Cichladusa ruficauda*	IndoMalay	0	-0.0952	-	3.35	0.7990	0.6799	78.08
Muscicapidae	*Cinclidium diana*	Australasia	0	-0.6191	-	3.26	0.7879	0.5157	73.92
Muscicapidae	*Cinclidium frontale*	Neotropics	0	-0.5180	-	3.27	0.7737	0.3993	71.17
Muscicapidae	*Cinclidium leucurum*	Afrotropics	0	-0.0961	-	3.53	0.8091	0.7722	79.79
Muscicapidae	*Copsychus albospecularis*	Australasia	0	-0.9077	-	3.64	0.7934	0.3451	15.12
Muscicapidae	*Copsychus cebuensis*	Palearctic	0	-0.6102	9.1628	3.83	0.7806	0.3785	8.34
Muscicapidae	*Copsychus luzoniensis*	Neotropics	1	-20.0053	1.2909	4.14	0.6120	0.0495	40.98
Muscicapidae	*Copsychus malabaricus*	Neotropics	0	-0.6912	5.1001	3.30	0.6974	0.2929	71.75
Muscicapidae	*Copsychus niger*	Neotropics	0	0.0110	1.8241	3.55	0.7185	0.3996	44.10
Muscicapidae	*Copsychus saularis*	Neotropics	0	-0.3316	1.4507	2.53	0.6986	0.2608	17.52
Muscicapidae	*Cossypha albicapilla*	Australasia	0	-0.0948	-	2.78	0.7705	0.2897	23.45
Muscicapidae	*Cossypha anomala*	Australasia	0	-0.1500	-	2.74	0.7096	0.8066	80.56
Muscicapidae	*Cossypha caffra*	Australasia	0	-3.0110	-	2.48	0.6302	0.2423	87.23
Muscicapidae	*Cossypha cyanocampter*	Australasia	0	1.3482	-	2.46	0.5788	0.0000	0.00
Muscicapidae	*Cossypha dichroa*	IndoMalay	0	-0.0019	-	2.91	0.8048	0.5674	57.70
Muscicapidae	*Cossypha heinrichi*	Neotropics	1	-0.3731	4.7375	3.77	0.8033	0.6617	18.75
Muscicapidae	*Cossypha heuglini*	Neotropics	0	-0.5539	-	3.00	0.7935	0.4753	75.12
Muscicapidae	*Cossypha humeralis*	Neotropics	0	-0.3503	-	3.35	0.8224	0.6773	74.50
Muscicapidae	*Cossypha isabellae*	Neotropics	0	-0.1191	-	3.72	0.8118	0.4178	38.61

Family	Species	Realm	Threat	Latitude	Elevation	Anomaly	Size	Shape	Orientation
Muscicapidae	*Cossypha natalensis*	Afrotropics	0	0.0917	11.3261	3.28	0.8216	0.3580	13.08
Muscicapidae	*Cossypha niveicapilla*	Afrotropics	0	-0.1623	7.0844	3.81	0.8206	0.6257	12.96
Muscicapidae	*Cossypha polioptera*	Afrotropics	0	0.0505	7.0547	3.35	0.8073	0.5554	56.92
Muscicapidae	*Cossypha semirufa*	Afrotropics	0	0.0241	8.4878	3.41	0.7770	0.4464	67.07
Muscicapidae	*Cossyphicula roberti*	Neotropics	0	-0.4067	-	3.70	0.8081	0.7626	23.99
Muscicapidae	*Culicicapa ceylonensis*	Neotropics	0	-0.2969	4.9868	3.52	0.8270	0.5579	30.02
Muscicapidae	*Culicicapa helianthea*	Neotropics	0	-0.2390	-	3.06	0.6859	0.2270	84.63
Muscicapidae	*Cyanoptila cyanomelana*	Neotropics	0	-0.3287	-	3.17	0.7030	0.2699	86.24
Muscicapidae	*Cyornis banyumas*	Palearctic	0	-0.9509	-	3.94	0.7935	0.6084	3.97
Muscicapidae	*Cyornis caerulatus*	Australasia	0	-0.4514	3.5531	2.32	0.7087	0.2285	42.33
Muscicapidae	*Cyornis concretus*	Australasia	1	-0.6684	5.0092	2.22	0.6906	0.2040	35.34
Muscicapidae	*Cyornis hainanus*	Australasia	1	-0.4531	0.0000	2.33	0.7331	0.2569	44.63
Muscicapidae	*Cyornis herioti*	Australasia	1	-0.0693	0.0000	2.58	0.6302	0.1134	34.50
Muscicapidae	*Cyornis hoevelli*	Neotropics	0	-0.2068	-	3.73	0.8545	0.6087	46.57
Muscicapidae	*Cyornis hyacinthinus*	Neotropics	0	-0.1691	7.8663	3.24	0.7291	0.3058	57.46
Muscicapidae	*Cyornis lemprieri*	Australasia	0	-0.2707	-	2.79	0.7639	0.4098	50.43
Muscicapidae	*Cyornis magnirostris*	Australasia	0	-0.0897	-	2.79	0.7656	0.3501	23.62
Muscicapidae	*Cyornis omissus*	Australasia	0	-0.5047	0.0000	3.40	0.8292	0.4692	15.98
Muscicapidae	*Cyornis pallipes*	Nearctic	0	-0.7204	-	4.01	0.8007	0.1535	18.89
Muscicapidae	*Cyornis poliogenys*	Palearctic	0	-0.6363	3.2858	6.01	0.8328	0.0304	0.53
Muscicapidae	*Cyornis rubeculoides*	Palearctic	0	-0.7934	-	5.10	0.8609	0.1996	0.11
Muscicapidae	*Cyornis ruckii*	Neotropics	0	-0.5082	2.7566	2.15	0.7862	0.3965	51.79
Muscicapidae	*Cyornis rufigastra*	Palearctic	0	-0.7541	-	4.19	0.8347	0.1166	0.97
Muscicapidae	*Cyornis sanfordi*	Neotropics	0	-0.0282	-	4.01	0.8359	0.6905	22.00
Muscicapidae	*Cyornis superbus*	Neotropics	0	0.1034	-	4.24	0.7618	0.4847	8.25
Muscicapidae	*Cyornis tickelliae*	IndoMalay	0	0.0045	1.7696	2.88	0.7969	0.5767	40.99
Muscicapidae	*Cyornis turcosus*	Nearctic	0	-0.1574	9.2467	3.89	0.7765	0.2775	46.81
Muscicapidae	*Cyornis unicolor*	Neotropics	0	0.8437	7.4806	3.61	0.7136	0.2718	19.49
Muscicapidae	*Dioptrornis brunneus*	Neotropics	0	3.8401	9.1772	4.04	0.6997	0.4220	18.33
Muscicapidae	*Dioptrornis chocolatinus*	Neotropics	0	0.0339	8.7217	3.65	0.7705	0.4343	82.69
Muscicapidae	*Dioptrornis fischeri*	Neotropics	0	0.9925	5.3330	3.96	0.7128	-	-
Muscicapidae	*Dioptrornis fischeri*	Neotropics	0	0.9925	5.3330	3.96	0.7128	0.4982	88.09
Muscicapidae	*Empidornis semipartitus*	Neotropics	0	-0.2010	-	3.67	0.8291	0.5919	59.18
Muscicapidae	*Enicurus immaculatus*	Neotropics	0	-0.6096	-	2.94	0.8123	0.6946	38.07
Muscicapidae	*Enicurus leschenaulti*	Australasia	0	-0.5703	-	3.62	0.7955	0.3511	3.00
Muscicapidae	*Enicurus maculatus*	Afrotropics	0	0.0909	-	3.31	0.7713	0.8244	32.30
Muscicapidae	*Enicurus ruficapillus*	Neotropics	0	-0.0393	11.3449	3.85	0.7684	0.2872	36.85
Muscicapidae	*Enicurus schistaceus*	Neotropics	0	0.0022	11.3545	3.77	0.7681	0.1730	30.77
Muscicapidae	*Enicurus scouleri*	Nearctic	0	-0.6256	4.4827	4.25	0.8387	0.1642	12.82
Muscicapidae	*Enicurus velatus*	Neotropics	0	0.1852	7.1718	3.02	0.6428	0.3087	29.93
Muscicapidae	*Erithacus akahige*	Palearctic	0	-0.6009	13.0756	4.62	0.8785	0.1323	0.70
Muscicapidae	*Erithacus rubecula*	Palearctic	0	-0.1725	3.6540	3.79	0.7977	0.1449	2.88
Muscicapidae	*Erythropygia barbata*	Afrotropics	0	-0.1122	4.7729	3.87	0.8421	0.2016	4.47
Muscicapidae	*Erythropygia coryphaeus*	Afrotropics	0	-0.0200	5.9320	3.32	0.7839	0.5644	41.98
Muscicapidae	*Erythropygia galactotes*	Afrotropics	0	-0.2776	-	3.99	0.8138	0.8126	69.24
Muscicapidae	*Erythropygia hartlaubi*	Neotropics	0	-0.1571	6.8065	3.74	0.7482	0.2777	37.80
Muscicapidae	*Erythropygia leucophrys*	Neotropics	1	-0.0550	10.0324	3.64	0.6784	0.5393	31.92
Muscicapidae	*Erythropygia leucosticta*	Neotropics	0	-2.6284	3.0707	3.46	0.7245	0.2715	78.67
Muscicapidae	*Erythropygia paena*	Neotropics	0	-0.0493	6.2684	3.84	0.7008	0.3228	55.38
Muscicapidae	*Erythropygia quadrivirgata*	Neotropics	0	10.8885	6.5833	3.11	0.6876	0.1686	59.02
Muscicapidae	*Erythropygia signata*	Neotropics	0	6.8912	8.2152	3.86	0.7050	0.2190	88.35
Muscicapidae	*Eumyias albicaudatus*	Neotropics	0	0.1155	-	3.97	0.8015	0.7217	52.16
Muscicapidae	*Eumyias indigo*	Neotropics	0	-14.3653	3.4450	4.20	0.7318	0.4330	56.11
Muscicapidae	*Eumyias panayensis*	Neotropics	0	-1.1230	9.2066	3.99	0.7522	0.1522	39.61
Muscicapidae	*Eumyias sordidus*	Neotropics	0	-0.1161	-	3.90	0.8339	0.4573	40.54
Muscicapidae	*Eumyias thalassinus*	Neotropics	0	-0.3316	0.3148	3.42	0.7940	0.4860	32.89
Muscicapidae	*Ficedula albicilla*	Australasia	0	-0.5517	1.9036	3.48	0.8293	0.4862	14.82
Muscicapidae	*Ficedula albicollis*	IndoMalay	0	-0.2468	-	3.62	0.8093	0.6051	19.10
Muscicapidae	*Ficedula basilanica*	Australasia	0	-0.5026	7.2485	3.33	0.8382	0.5902	9.30
Muscicapidae	*Ficedula bonthaina*	Nearctic	0	-0.7782	9.1048	4.13	0.8216	0.6619	61.01
Muscicapidae	*Ficedula buruensis*	Australasia	0	-0.0160	-	2.66	0.7510	0.4101	15.70
Muscicapidae	*Ficedula crypta*	Palearctic	0	-0.9152	7.7333	4.35	0.8495	0.1898	3.42
Muscicapidae	*Ficedula disposita*	Afrotropics	0	-0.0809	-	3.18	0.7614	0.3874	70.15
Muscicapidae	*Ficedula dumetoria*	Australasia	0	-0.4615	5.5162	2.33	0.7272	0.1650	39.02
Muscicapidae	*Ficedula harterti*	Palearctic	0	-0.5818	3.8281	4.12	0.8192	0.2678	6.89
Muscicapidae	*Ficedula hodgsonii*	Palearctic	0	-0.5384	8.5115	4.31	0.8864	0.3410	0.71
Muscicapidae	*Ficedula hyperythra*	Neotropics	0	-0.1318	-	3.83	0.8541	0.4251	34.80
Muscicapidae	*Ficedula hypoleuca*	Afrotropics	0	-0.0127	-	3.85	0.8177	0.2800	61.04
Muscicapidae	*Ficedula monileger*	Palearctic	0	-0.7378	2.9882	5.57	0.8452	0.0427	0.18
Muscicapidae	*Ficedula mugimaki*	IndoMalay	0	-0.0911	6.5832	3.04	0.8200	0.2544	34.03
Muscicapidae	*Ficedula narcissina*	Nearctic	0	-0.4601	12.3308	3.85	0.8705	0.3140	52.14
Muscicapidae	*Ficedula nigrorufa*	Palearctic	0	-0.6815	-	4.47	0.8747	0.2592	0.34
Muscicapidae	*Ficedula parva*	Australasia	0	-0.5074	3.5439	3.39	0.8316	0.5808	23.64
Muscicapidae	*Ficedula platenae*	Palearctic	0	-0.4418	10.2342	4.14	0.8883	0.4812	21.11

Family	Species	Realm	Threat	Latitude	Elevation	Anomaly	Size	Shape	Orientation
Muscicapidae	*Ficedula rufigula*	Palearctic	0	-0.8061	4.0733	5.04	0.8456	0.1907	4.36
Muscicapidae	*Ficedula sapphira*	Afrotropics	0	-0.0525	-	3.18	0.7569	0.3638	68.46
Muscicapidae	*Ficedula semitorquata*	Afrotropics	0	-0.1226	-	3.20	0.7446	0.2882	71.92
Muscicapidae	*Ficedula solitaris*	Australasia	0	-0.5881	-	3.03	0.7868	0.5564	5.17
Muscicapidae	*Ficedula strophiata*	Neotropics	1	-0.2401	-	2.54	0.5788	0.0000	0.00
Muscicapidae	*Ficedula subrubra*	Palearctic	0	-0.7657	0.0000	5.08	0.8483	0.1959	1.71
Muscicapidae	*Ficedula superciliaris*	Palearctic	0	-0.7771	-	4.23	0.8099	0.2505	11.11
Muscicapidae	*Ficedula timorensis*	IndoMalay	1	-0.0482	2.5437	2.62	0.7008	0.6308	85.20
Muscicapidae	*Ficedula tricolor*	Australasia	1	1.3401	4.3590	2.60	0.5788	0.0000	90.00
Muscicapidae	*Ficedula westermanni*	Australasia	0	0.3216	-	2.48	0.6478	0.1611	2.45
Muscicapidae	*Ficedula zanthopygia*	IndoMalay	0	-0.1036	4.4674	2.63	0.6859	0.7028	70.40
Muscicapidae	*Fraseria cinerascens*	Afrotropics	0	0.0429	3.1137	3.40	0.7851	0.5597	80.72
Muscicapidae	*Fraseria ocreata*	Afrotropics	0	2.3803	4.9764	3.37	0.7933	0.4455	11.43
Muscicapidae	*Fraseria ocreata*	Afrotropics	0	2.3803	4.9764	3.37	0.7933	-	-
Muscicapidae	*Grandala coelicolor*	Neotropics	0	0.1514	2.0361	4.10	0.7563	0.5841	83.63
Muscicapidae	*Hodgsonius phaenicuroides*	IndoMalay	0	0.1304	-	3.34	0.7522	0.1479	9.99
Muscicapidae	*Irania gutturalis*	Australasia	0	-0.2751	12.3147	2.84	0.7323	0.1873	23.32
Muscicapidae	*Luscinia brunnea*	Neotropics	0	-0.0162	4.9570	3.51	0.7474	0.2821	26.09
Muscicapidae	*Luscinia calliope*	Neotropics	0	-0.3690	-	3.61	0.8084	0.7055	39.83
Muscicapidae	*Luscinia cyane*	Neotropics	0	-0.0225	3.4416	4.05	0.7770	0.3211	17.76
Muscicapidae	*Luscinia luscinia*	Neotropics	0	0.2894	7.3608	4.38	0.7335	0.4875	13.17
Muscicapidae	*Luscinia megarhynchos*	Neotropics	0	-0.7941	4.2525	3.52	0.6986	0.1025	53.25
Muscicapidae	*Luscinia obscura*	Neotropics	0	-1.1644	8.5026	3.96	0.7128	0.3961	78.31
Muscicapidae	*Luscinia pectardens*	Neotropics	0	-0.3908	-	3.32	0.7654	0.3421	71.50
Muscicapidae	*Luscinia pectoralis*	Neotropics	0	0.0127	5.0211	3.95	0.8293	0.5220	25.92
Muscicapidae	*Luscinia ruficeps*	Afrotropics	0	-0.0741	6.6549	3.18	0.7613	0.3878	70.13
Muscicapidae	*Luscinia sibilans*	Neotropics	0	0.9741	-	4.22	0.7482	0.6729	17.22
Muscicapidae	*Luscinia svecica*	Neotropics	0	-0.0764	-	3.96	0.8078	0.4967	24.07
Muscicapidae	*Melaenornis annamarulae*	IndoMalay	0	-0.6251	6.1376	3.20	0.7060	0.3933	80.31
Muscicapidae	*Melaenornis ardesiacus*	IndoMalay	0	-0.2186	-	3.01	0.7776	0.6988	73.06
Muscicapidae	*Melaenornis edolioides*	IndoMalay	0	0.2931	-	2.76	0.6662	0.9678	90.00
Muscicapidae	*Melaenornis pammelaina*	IndoMalay	0	-0.0331	9.2531	3.26	0.7898	0.5247	46.51
Muscicapidae	*Modulatrix orostruthus*	Neotropics	0	0.0855	7.5237	3.86	0.7982	0.5998	48.71
Muscicapidae	*Modulatrix stictigula*	Afrotropics	0	-0.0845	-	3.67	0.8201	0.5518	52.99
Muscicapidae	*Monticola angolensis*	Afrotropics	0	0.1462	-	3.34	0.7698	0.4890	45.60
Muscicapidae	*Monticola angolensis*	Afrotropics	0	0.1462	-	3.34	0.7698	-	-
Muscicapidae	*Monticola brevipes*	Afrotropics	0	0.0797	6.7753	3.34	0.7801	0.3457	57.94
Muscicapidae	*Monticola cinclorhynchus*	Afrotropics	0	-0.1736	-	3.78	0.7765	0.3313	15.53
Muscicapidae	*Monticola erythronotus*	Afrotropics	0	-0.3535	-	4.06	0.8009	0.6720	78.38
Muscicapidae	*Monticola explorator*	Afrotropics	1	-0.1734	-	3.03	0.5985	0.2277	28.15
Muscicapidae	*Monticola gularis*	IndoMalay	0	-0.1227	-	3.28	0.8168	0.4598	13.74
Muscicapidae	*Monticola imerinus*	Afrotropics	0	-0.1742	5.3593	3.63	0.8251	0.2103	3.46
Muscicapidae	*Monticola rufiventris*	Afrotropics	0	2.6063	-	3.80	0.7461	0.5672	67.81
Muscicapidae	*Monticola rufocinereus*	Afrotropics	0	-0.1350	-	3.25	0.7510	0.3192	37.09
Muscicapidae	*Monticola rupestris*	Afrotropics	0	0.1013	-	3.99	0.7989	0.1293	10.46
Muscicapidae	*Monticola saxatilis*	IndoMalay	0	0.0391	2.3979	3.03	0.7790	-	-
Muscicapidae	*Monticola saxatilis*	IndoMalay	0	0.0391	2.3979	3.03	0.7790	0.5028	35.30
Muscicapidae	*Monticola sharpei*	IndoMalay	0	-0.1358	-	3.48	0.7948	0.7770	2.07
Muscicapidae	*Monticola solitarius*	Afrotropics	0	-0.0188	6.5676	3.41	0.7643	0.5723	25.59
Muscicapidae	*Muscicapa aquatica*	Australasia	0	-0.2844	-	2.52	0.6921	0.7329	50.04
Muscicapidae	*Muscicapa boehmi*	Australasia	0	-0.4944	-	2.20	0.7069	0.2780	38.25
Muscicapidae	*Muscicapa caerulescens*	Australasia	1	-0.5648	4.4847	2.09	0.6859	0.3191	34.07
Muscicapidae	*Muscicapa cassini*	Neotropics	0	-0.1926	7.1634	3.73	0.8066	0.2762	35.42
Muscicapidae	*Muscicapa comitata*	Nearctic	0	-0.7427	-	4.15	0.8495	0.5558	8.20
Muscicapidae	*Muscicapa dauurica*	Neotropics	0	-0.2492	-	3.58	0.8543	0.7366	68.63
Muscicapidae	*Muscicapa epulata*	Neotropics	0	-0.1594	5.3333	3.89	0.8485	0.4897	42.41
Muscicapidae	*Muscicapa ferruginea*	Neotropics	0	-0.6178	-	3.03	0.8148	0.6165	51.64
Muscicapidae	*Muscicapa gambagae*	Neotropics	0	-0.1210	-	3.69	0.7522	0.2244	34.48
Muscicapidae	*Muscicapa griseisticta*	Neotropics	0	-0.0825	5.8916	3.93	0.8465	0.4889	38.34
Muscicapidae	*Muscicapa infuscata*	Australasia	0	-0.0608	-	2.77	0.7632	0.2333	19.89
Muscicapidae	*Muscicapa lendu*	Australasia	0	-0.0678	-	2.80	0.7352	0.1812	24.89
Muscicapidae	*Muscicapa muttui*	Australasia	0	0.0862	3.2389	2.48	0.6120	0.0400	36.10
Muscicapidae	*Muscicapa olivascens*	Australasia	0	-0.4480	-	2.46	0.5985	0.1035	28.88
Muscicapidae	*Muscicapa ruficauda*	Australasia	0	-0.0849	-	2.78	0.7707	0.3150	22.66
Muscicapidae	*Muscicapa segregata*	Australasia	0	-0.0716	-	2.63	0.7310	0.9825	49.58
Muscicapidae	*Muscicapa sethsmithi*	Australasia	0	-0.4961	3.6657	2.50	0.5788	0.0000	45.57
Muscicapidae	*Muscicapa sibirica*	Australasia	0	-0.1444	-	2.82	0.7515	0.4652	35.67
Muscicapidae	*Muscicapa striata*	Australasia	0	-0.0955	2.5447	2.78	0.7711	0.3093	23.60
Muscicapidae	*Muscicapa tessmanni*	Australasia	0	-0.3803	-	3.16	0.7488	0.1215	55.87
Muscicapidae	*Muscicapa ussheri*	Australasia	0	-0.5015	4.7634	2.49	0.5788	0.0000	0.00
Muscicapidae	*Muscicapella hodgsoni*	Australasia	0	-0.0669	-	2.77	0.7640	0.2067	22.87
Muscicapidae	*Myiomela albiventris*	Neotropics	0	-0.2867	13.3540	3.88	0.7869	0.3824	73.81
Muscicapidae	*Myiomela major*	Afrotropics	0	0.0102	9.0552	3.53	0.8169	0.8850	65.76
Muscicapidae	*Myioparus griseigularis*	Neotropics	0	-0.0275	10.6023	3.66	0.7744	0.4089	78.47

Family	Species	Realm	Threat	Latitude	Elevation	Anomaly	Size	Shape	Orientation
Muscicapidae	*Myioparus plumbeus*	Nearctic	0	-0.8529	15.8338	3.90	0.8214	0.2861	42.06
Muscicapidae	*Myrmecocichla aethiops*	Neotropics	0	0.4041	6.0085	3.32	0.6120	0.9804	90.00
Muscicapidae	*Myrmecocichla albifrons*	Neotropics	0	0.0246	7.6301	3.74	0.7485	0.2239	50.46
Muscicapidae	*Myrmecocichla arnoti*	Neotropics	0	-0.0187	9.4812	3.72	0.8023	0.3354	39.47
Muscicapidae	*Myrmecocichla melaena*	Nearctic	0	-0.2400	6.8752	3.97	0.7918	0.2549	45.62
Muscicapidae	*Myrmecocichla nigra*	Neotropics	0	0.2561	11.6907	3.02	0.6740	0.2182	28.75
Muscicapidae	*Myrmecocichla tholloni*	Neotropics	0	-0.0721	10.8061	3.63	0.7780	0.4428	80.38
Muscicapidae	*Namibornis herero*	Afrotropics	0	-0.1886	9.1460	3.75	0.8167	0.7720	6.25
Muscicapidae	*Niltava davidi*	Neotropics	0	0.0331	4.3966	2.93	0.6921	0.0695	59.10
Muscicapidae	*Niltava grandis*	Neotropics	0	-0.1127	-	3.72	0.7961	0.6017	68.16
Muscicapidae	*Niltava macgrigoriae*	Neotropics	0	0.4837	-	3.84	0.7889	0.7012	0.32
Muscicapidae	*Niltava sumatrana*	Afrotropics	0	-0.1516	6.9428	3.06	0.7291	0.1578	75.24
Muscicapidae	*Niltava sundara*	Afrotropics	1	-0.1050	7.5667	3.11	0.6906	0.1114	73.62
Muscicapidae	*Oenanthe albonigra*	Nearctic	0	-0.8284	-	4.18	0.8064	0.6852	18.73
Muscicapidae	*Oenanthe bifasciata*	Palearctic	0	-0.3956	-	4.82	0.8530	0.1510	0.85
Muscicapidae	*Oenanthe bottae*	Nearctic	1	-0.8361	1.3312	4.91	0.7623	0.2978	5.28
Muscicapidae	*Oenanthe chrysopygia*	Palearctic	1	-0.7374	0.0000	4.50	0.8040	0.4657	9.56
Muscicapidae	*Oenanthe cypriaca*	Palearctic	0	-0.2334	-	5.51	0.7851	0.1510	1.40
Muscicapidae	*Oenanthe deserti*	Palearctic	0	-0.6628	-	5.20	0.8314	0.0468	0.20
Muscicapidae	*Oenanthe finschii*	Nearctic	1	-0.5739	0.5244	5.06	0.7008	0.5033	38.37
Muscicapidae	*Oenanthe hispanica*	Palearctic	1	-0.3603	-	5.62	0.6428	-	-
Muscicapidae	*Oenanthe hispanica*	Palearctic	1	-0.3603	-	5.62	0.6428	0.8229	0.00
Muscicapidae	*Oenanthe isabellina*	Afrotropics	0	-0.2273	12.2919	3.55	0.8559	0.4360	14.23
Muscicapidae	*Oenanthe leucopyga*	Neotropics	0	-0.3194	-	3.68	0.8268	0.3741	42.59
Muscicapidae	*Oenanthe leucura*	Neotropics	0	-0.1479	-	3.95	0.8282	0.5540	39.76
Muscicapidae	*Oenanthe lugens*	Neotropics	0	-0.4616	-	3.72	0.7128	0.1283	54.51
Muscicapidae	*Oenanthe lugentoides*	Neotropics	0	-0.1220	-	3.94	0.8422	0.5643	39.54
Muscicapidae	*Oenanthe moesta*	Neotropics	0	-0.2120	-	3.77	0.8545	0.6485	43.26
Muscicapidae	*Oenanthe monacha*	Neotropics	0	-0.0727	-	3.37	0.7781	0.3143	8.91
Muscicapidae	*Oenanthe monticola*	Neotropics	0	-0.1791	-	4.30	0.7593	0.3895	68.21
Muscicapidae	*Oenanthe oenanthe*	Neotropics	0	0.0000	5.8473	3.26	0.6221	0.5774	71.57
Muscicapidae	*Oenanthe phillipsi*	Australasia	0	-0.2712	-	3.20	0.8391	0.6805	45.33
Muscicapidae	*Oenanthe picata*	Neotropics	0	-0.4232	-	3.77	0.8668	0.3250	56.60
Muscicapidae	*Oenanthe pileata*	Neotropics	0	-0.1758	6.2402	3.80	0.8546	0.4212	36.72
Muscicapidae	*Oenanthe pleschanka*	Neotropics	0	-0.1375	7.4370	3.77	0.7422	0.2046	50.62
Muscicapidae	*Oenanthe xanthoprymna*	Neotropics	0	-0.0995	4.6445	3.91	0.8189	0.4411	26.02
Muscicapidae	*Philentoma pyrhoptera*	IndoMalay	0	-0.0130	-	2.84	0.7887	0.4901	7.43
Muscicapidae	*Philentoma velata*	IndoMalay	0	-0.0095	2.8263	2.67	0.6690	0.4542	81.93
Muscicapidae	*Phoenicurus alaschanicus*	Neotropics	0	-0.2735	12.6228	3.04	0.6784	0.1941	29.99
Muscicapidae	*Phoenicurus auroreus*	Afrotropics	0	-0.2285	-	3.52	0.8612	-	-
Muscicapidae	*Phoenicurus auroreus*	Afrotropics	0	-0.2285	-	3.52	0.8612	0.7153	41.35
Muscicapidae	*Phoenicurus caeruleocephala*	Nearctic	0	-0.6807	6.7846	4.21	0.8213	0.3709	11.12
Muscicapidae	*Phoenicurus erythrogastrus*	Neotropics	0	-0.2751	-	3.59	0.8615	0.6149	55.49
Muscicapidae	*Phoenicurus erythrogastrus*	Neotropics	0	-0.2751	-	3.59	0.8615	-	-
Muscicapidae	*Phoenicurus erythronotus*	Palearctic	0	-0.4617	-	3.70	0.8731	0.6009	12.16
Muscicapidae	*Phoenicurus frontalis*	IndoMalay	0	-0.0976	0.0000	3.27	0.7850	0.5248	15.31
Muscicapidae	*Phoenicurus hodgsoni*	Australasia	0	-0.5625	0.0000	2.95	0.8029	0.4038	7.98
Muscicapidae	*Phoenicurus moussieri*	IndoMalay	0	-0.0939	-	3.27	0.8221	0.4723	20.60
Muscicapidae	*Phoenicurus ochruros*	Palearctic	0	-0.9229	-	4.11	0.7851	0.2646	0.23
Muscicapidae	*Phoenicurus phoenicurus*	Australasia	0	-0.3634	-	2.87	0.8093	0.6198	45.88
Muscicapidae	*Phoenicurus schisticeps*	Australasia	0	-0.4881	-	3.22	0.8246	-	-
Muscicapidae	*Phoenicurus schisticeps*	Australasia	0	-0.4881	-	3.22	0.8246	0.5771	21.59
Muscicapidae	*Pinarornis plumosus*	IndoMalay	0	-0.0163	-	2.63	0.7078	0.6547	74.73
Muscicapidae	*Pogonocichla stellata*	Afrotropics	0	0.0740	-	3.40	0.8110	0.2151	8.27
Muscicapidae	*Pogonocichla stellata*	Afrotropics	0	0.0740	-	3.40	0.8110	-	-
Muscicapidae	*Rhinomyias additus*	Australasia	0	-0.6740	-	2.99	0.7378	0.3140	46.02
Muscicapidae	*Rhinomyias albigularis*	Australasia	0	-0.0704	-	2.81	0.7435	0.1602	23.76
Muscicapidae	*Rhinomyias brunneatus*	Australasia	0	-0.0754	-	2.78	0.7636	0.2044	23.07
Muscicapidae	*Rhinomyias colonus*	Australasia	0	-0.1066	-	2.78	0.7706	0.3097	23.63
Muscicapidae	*Rhinomyias goodfellowi*	Neotropics	0	-0.4089	4.0247	2.12	0.7602	0.2536	89.45
Muscicapidae	*Rhinomyias gularis*	Neotropics	0	0.1045	-	4.02	0.8267	0.6139	3.54
Muscicapidae	*Rhinomyias insignis*	Neotropics	0	-0.3152	9.1907	3.47	0.8533	0.7218	83.19
Muscicapidae	*Rhinomyias olivaceus*	Neotropics	0	0.1685	-	1.51	0.6221	0.0002	1.05
Muscicapidae	*Rhinomyias oscillans*	Afrotropics	0	-0.2041	-	3.37	0.7619	0.2984	73.97
Muscicapidae	*Rhinomyias ruficauda*	Afrotropics	0	0.0429	-	3.36	0.8274	0.4988	28.68
Muscicapidae	*Rhinomyias umbratilis*	Afrotropics	0	0.0990	-	3.21	0.7595	0.3464	32.25
Muscicapidae	*Rhinomyias umbratilis*	Afrotropics	0	0.0990	-	3.21	0.7595	-	-
Muscicapidae	*Rhyacornis bicolor*	Nearctic	0	-0.8667	-	3.35	0.7385	0.5127	27.49
Muscicapidae	*Rhyacornis fuliginosa*	Nearctic	0	-0.2001	-	4.01	0.8281	0.4538	44.37
Muscicapidae	*Saxicola caprata*	IndoMalay	0	0.0277	2.2871	2.84	0.7861	0.4266	0.86
Muscicapidae	*Saxicola ferreus*	IndoMalay	0	0.0026	2.1973	2.83	0.7887	0.5968	8.30
Muscicapidae	*Saxicola gutturalis*	Afrotropics	0	0.0757	8.1924	3.60	0.8410	0.5880	12.92
Muscicapidae	*Saxicola insignis*	Afrotropics	0	-0.0758	9.5984	3.73	0.8345	0.5334	51.70
Muscicapidae	*Saxicola jerdoni*	Afrotropics	0	0.0078	7.9677	3.32	0.8038	0.4850	52.17

Family	Species	Realm	Threat	Latitude	Elevation	Anomaly	Size	Shape	Orientation
Muscicapidae	*Saxicola leucurus*	Afrotropics	0	-0.1736	-	3.75	0.8157	0.2512	55.51
Muscicapidae	*Saxicola macrorhynchus*	IndoMalay	1	0.0324	-	3.25	0.6598	0.5815	18.08
Muscicapidae	*Saxicola rubetra*	Afrotropics	0	-0.0891	-	3.62	0.8485	0.5791	42.31
Muscicapidae	*Saxicola torquatus*	Afrotropics	0	-0.0479	-	3.55	0.8064	0.2808	57.24
Muscicapidae	*Saxicoloides fulicatus*	IndoMalay	0	-0.0856	-	3.30	0.8316	0.6729	9.93
Muscicapidae	*Sheppardia aequatorialis*	Neotropics	0	-0.1338	-	3.99	0.8302	0.6205	0.04
Muscicapidae	*Sheppardia aurantiithorax*	Neotropics	0	-0.0519	-	3.32	0.7629	0.3236	40.77
Muscicapidae	*Sheppardia bocagei*	Neotropics	0	-0.1131	-	3.90	0.8356	0.4897	24.41
Muscicapidae	*Sheppardia cyornithopsis*	Neotropics	0	-0.0898	-	4.02	0.8271	0.6532	4.16
Muscicapidae	*Sheppardia gabela*	Neotropics	0	-0.2909	-	3.50	0.8111	0.3987	53.23
Muscicapidae	*Sheppardia gunningi*	IndoMalay	0	-0.0050	-	2.64	0.6740	0.1516	67.28
Muscicapidae	*Sheppardia lowei*	Australasia	0	-0.0384	3.6990	2.71	0.6921	0.4054	50.77
Muscicapidae	*Sheppardia montana*	Nearctic	0	-0.9102	-	4.27	0.8239	0.6269	15.19
Muscicapidae	*Sigelus silens*	Neotropics	0	-0.2023	5.5507	3.70	0.6120	0.0000	0.00
Muscicapidae	*Stenostira scita*	IndoMalay	1	-0.0480	5.4851	3.26	0.7584	0.5315	49.55
Muscicapidae	*Stiphrornis erythrothorax*	Afrotropics	0	-0.0381	-	3.42	0.8178	0.4601	27.16
Muscicapidae	*Swynnertonia swynnertoni*	Neotropics	0	-0.0381	7.3354	3.70	0.7749	0.4254	78.92
Muscicapidae	*Tarsiger chrysaeus*	Palearctic	0	-1.0392	9.8096	4.18	0.8179	0.2140	2.07
Muscicapidae	*Tarsiger chrysaeus*	Palearctic	0	-1.0392	9.8096	4.18	0.8179	-	-
Muscicapidae	*Tarsiger cyanurus*	Palearctic	0	-1.0108	3.6581	4.06	0.7998	0.3128	9.20
Muscicapidae	*Tarsiger hyperythrus*	Palearctic	0	-0.8199	7.6945	4.21	0.8370	0.0987	8.88
Muscicapidae	*Tarsiger indicus*	Palearctic	0	-0.7183	-	4.57	0.8484	0.2721	2.27
Muscicapidae	*Tarsiger johnstoniae*	Palearctic	0	-0.8294	3.6499	3.83	0.7446	0.3176	7.07
Muscicapidae	*Trichixos pyrropygus*	Australasia	0	-0.3872	-	2.95	0.7616	0.5334	80.20
Muscicapidae	*Xenocopsychus ansorgei*	Nearctic	0	-0.7798	-	4.30	0.8351	0.2573	15.42
Musophagidae	*Corythaeola cristata*	Australasia	0	-0.2853	-	3.40	0.8304	0.7123	13.07
Musophagidae	*Corythaixoides concolor*	Nearctic	0	-0.8944	-	3.86	0.7861	0.4788	20.46
Musophagidae	*Corythaixoides leucogaster*	Neotropics	0	-0.3126	3.2549	2.51	0.7120	0.1190	19.09
Musophagidae	*Corythaixoides personatus*	Afrotropics	0	-0.2211	16.9656	3.73	0.8165	0.8465	9.83
Musophagidae	*Crinifer piscator*	Neotropics	0	-0.0113	2.2905	3.66	0.7825	0.4711	46.04
Musophagidae	*Crinifer zonurus*	Neotropics	0	-0.2906	7.3125	3.90	0.7352	0.4258	21.76
Musophagidae	*Musophaga rossae*	IndoMalay	0	-0.1031	6.0105	4.21	0.7659	0.1609	26.11
Musophagidae	*Ruwenzorornis johnstoni*	Neotropics	0	0.0258	9.6176	3.57	0.7505	0.3257	86.53
Musophagidae	*Tauraco bannermani*	Palearctic	0	-0.3462	6.5921	3.00	0.6523	0.3484	12.24
Musophagidae	*Tauraco corythaix*	Afrotropics	0	-0.3187	-	3.98	0.8128	0.7564	46.92
Musophagidae	*Tauraco erythrolophus*	Palearctic	0	-0.6482	-	3.45	0.7833	0.6550	4.82
Musophagidae	*Tauraco fischeri*	Afrotropics	0	-0.0184	-	3.47	0.8403	0.2228	6.74
Musophagidae	*Tauraco hartlaubi*	Afrotropics	0	0.0556	-	3.21	0.7925	0.2321	15.99
Musophagidae	*Tauraco leucolophus*	Afrotropics	0	-0.1112	-	3.33	0.7819	0.4936	51.22
Musophagidae	*Tauraco leucotis*	Afrotropics	0	6.8755	7.0182	3.34	0.7409	0.5620	25.03
Musophagidae	*Tauraco livingstonii*	Afrotropics	0	-15.2237	4.2185	3.34	0.7403	0.3922	35.32
Musophagidae	*Tauraco macrorhynchus*	Afrotropics	0	-0.2675	-	3.84	0.8245	0.7592	62.98
Musophagidae	*Tauraco persa*	Afrotropics	0	-0.0831	-	3.64	0.8106	0.6133	24.19
Musophagidae	*Tauraco porphyreolophus*	Afrotropics	0	-0.0337	-	3.28	0.8217	0.3409	14.62
Musophagidae	*Tauraco ruspolii*	Afrotropics	0	-0.0814	-	3.43	0.8047	0.3299	81.63
Musophagidae	*Tauraco schalowi*	Neotropics	0	-0.5766	-	2.20	0.7528	0.1668	81.86
Musophagidae	*Tauraco schuetti*	Palearctic	0	-0.1541	-	3.47	0.7869	0.3172	7.41
Musophagidae	*Tauraco schuetti*	Palearctic	0	-0.1541	-	3.47	0.7869	-	-
Nectariniidae	*Aethopyga bella*	Palearctic	0	-0.3109	1.4262	4.95	0.7060	0.0313	1.01
Nectariniidae	*Aethopyga boltoni*	IndoMalay	0	0.0007	4.8824	2.64	0.7323	0.2320	67.55
Nectariniidae	*Aethopyga christinae*	IndoMalay	0	-0.0112	-	2.63	0.6631	0.6679	76.33
Nectariniidae	*Aethopyga eximia*	IndoMalay	0	-0.3319	-	3.24	0.7984	0.9078	68.59
Nectariniidae	*Aethopyga flagrans*	IndoMalay	0	-0.5821	10.1199	2.72	0.6859	0.1000	10.20
Nectariniidae	*Aethopyga gouldiae*	IndoMalay	0	-0.0561	-	2.65	0.7087	0.2460	75.53
Nectariniidae	*Aethopyga ignicauda*	Palearctic	0	-0.3604	-	3.57	0.8193	0.5592	8.26
Nectariniidae	*Aethopyga linaraborae*	IndoMalay	0	-0.0283	-	3.77	0.7805	0.4298	17.97
Nectariniidae	*Aethopyga mystacalis*	IndoMalay	0	-0.1865	5.6663	2.65	0.5985	0.0000	90.00
Nectariniidae	*Aethopyga nipalensis*	IndoMalay	0	-0.0112	-	2.82	0.7767	0.5962	8.97
Nectariniidae	*Aethopyga primigenia*	IndoMalay	0	-0.0402	-	3.38	0.7919	0.5002	29.14
Nectariniidae	*Aethopyga pulcherrima*	IndoMalay	0	-0.0587	-	2.63	0.6763	0.5091	78.07
Nectariniidae	*Aethopyga saturata*	IndoMalay	0	-0.0214	-	2.64	0.7234	0.2096	67.39
Nectariniidae	*Aethopyga shelleyi*	IndoMalay	0	-0.0832	-	3.20	0.8052	0.6781	38.40
Nectariniidae	*Aethopyga siparaja*	IndoMalay	0	0.1892	3.8413	2.52	0.5985	0.0024	45.41
Nectariniidae	*Anthreptes anchietae*	Afrotropics	0	-0.2567	-	3.69	0.7702	0.3403	0.74
Nectariniidae	*Anthreptes aurantium*	Afrotropics	0	0.0678	-	3.27	0.7986	0.5319	7.61
Nectariniidae	*Anthreptes collaris*	Afrotropics	0	-0.0860	-	3.41	0.8452	0.5763	29.04
Nectariniidae	*Anthreptes fraseri*	Afrotropics	0	0.1052	6.4058	3.26	0.8164	0.2537	14.76
Nectariniidae	*Anthreptes gabonicus*	Afrotropics	0	-0.0002	-	3.16	0.7919	0.3172	22.12
Nectariniidae	*Anthreptes longuemarei*	Afrotropics	0	-0.1637	-	3.50	0.8324	0.4331	33.20
Nectariniidae	*Anthreptes malacensis*	IndoMalay	0	-0.0414	-	2.85	0.8063	0.6282	40.75
Nectariniidae	*Anthreptes metallicus*	Afrotropics	0	-0.4331	6.9847	3.77	0.7931	0.5668	2.38
Nectariniidae	*Anthreptes neglectus*	Afrotropics	0	-0.0854	-	3.14	0.7419	0.4657	87.60
Nectariniidae	*Anthreptes orientalis*	Afrotropics	0	0.0094	-	3.32	0.8031	0.6021	51.38
Nectariniidae	*Anthreptes pallidigaster*	Afrotropics	1	0.1582	3.2721	3.12	0.6120	0.0572	50.21

Family	Species	Realm	Threat	Latitude	Elevation	Anomaly	Size	Shape	Orientation
Nectariniidae	*Anthreptes platurus*	Afrotropics	0	-0.0617	-	3.62	0.8235	0.1962	7.25
Nectariniidae	*Anthreptes rectirostris*	Afrotropics	0	0.0845	-	3.26	0.8152	0.3019	12.35
Nectariniidae	*Anthreptes reichenowi*	Afrotropics	0	-0.1349	2.6346	3.31	0.7600	0.1875	69.84
Nectariniidae	*Anthreptes rhodolaemus*	IndoMalay	0	0.0124	1.8056	2.80	0.7642	0.5110	12.48
Nectariniidae	*Anthreptes rubritorques*	Afrotropics	1	0.0686	4.2534	3.13	0.6562	0.1928	57.13
Nectariniidae	*Anthreptes simplex*	IndoMalay	0	0.0058	-	2.83	0.7870	0.5220	11.47
Nectariniidae	*Anthreptes singalensis*	IndoMalay	0	-0.0925	-	2.98	0.8152	0.3832	58.00
Nectariniidae	*Arachnothera affinis*	Neotropics	1	-14.8772	10.8626	3.96	0.6690	0.9066	53.23
Nectariniidae	*Arachnothera chrysogenys*	Neotropics	0	0.0221	2.3938	3.98	0.8325	0.7298	24.09
Nectariniidae	*Arachnothera crassirostris*	IndoMalay	0	-0.0295	-	2.83	0.7919	0.6133	15.29
Nectariniidae	*Arachnothera everetti*	IndoMalay	0	0.0169	2.6218	2.83	0.7887	0.5288	6.87
Nectariniidae	*Arachnothera flavigaster*	IndoMalay	0	-0.0176	3.4490	2.64	0.7256	0.2114	67.51
Nectariniidae	*Arachnothera juliae*	IndoMalay	0	0.0515	-	2.82	0.7811	0.4099	8.07
Nectariniidae	*Arachnothera longirostra*	IndoMalay	0	0.1815	-	2.85	0.7008	0.4401	54.57
Nectariniidae	*Arachnothera magna*	IndoMalay	0	-0.0021	-	2.84	0.7874	0.4818	7.24
Nectariniidae	*Arachnothera robusta*	IndoMalay	0	0.1714	-	2.91	0.7217	0.4065	65.42
Nectariniidae	*Hypogramma hypogrammicum*	Neotropics	0	0.7988	-	3.96	0.7640	0.6538	4.46
Nectariniidae	*Nectarinia adelberti*	Neotropics	0	0.1096	-	4.07	0.8272	0.6161	13.69
Nectariniidae	*Nectarinia afra*	Neotropics	0	0.0607	-	4.08	0.7613	0.6561	6.09
Nectariniidae	*Nectarinia alinae*	Neotropics	0	-0.2458	-	3.62	0.7993	0.2432	30.88
Nectariniidae	*Nectarinia alinae*	Neotropics	0	-0.2458	-	3.62	0.7993	-	-
Nectariniidae	*Nectarinia amethystina*	Neotropics	0	-0.1932	3.2598	3.99	0.8151	0.5738	19.89
Nectariniidae	*Nectarinia amethystina*	Neotropics	0	-0.1932	3.2598	3.99	0.8151	-	-
Nectariniidae	*Nectarinia asiatica*	Neotropics	0	0.0679	-	4.02	0.8252	0.5940	18.42
Nectariniidae	*Nectarinia aspasia*	Neotropics	0	-0.6029	9.0926	4.31	0.7179	0.2680	3.39
Nectariniidae	*Nectarinia balfouri*	Neotropics	0	0.4304	-	3.82	0.7266	0.4875	26.17
Nectariniidae	*Nectarinia bannermani*	Neotropics	0	-0.1545	-	4.17	0.7721	0.7843	4.84
Nectariniidae	*Nectarinia batesi*	Neotropics	0	-0.1298	-	3.95	0.8408	0.6047	26.81
Nectariniidae	*Nectarinia bifasciata*	Neotropics	0	0.3327	5.3017	4.10	0.7493	0.3353	6.16
Nectariniidae	*Nectarinia bocagii*	Neotropics	0	-0.0300	3.0639	4.05	0.8334	0.7674	7.70
Nectariniidae	*Nectarinia bouvieri*	Neotropics	0	0.4211	-	3.92	0.7672	0.4907	17.78
Nectariniidae	*Nectarinia buettikoferi*	Neotropics	1	-0.2049	0.9058	2.64	0.5788	0.0000	0.00
Nectariniidae	*Nectarinia calcostetha*	Neotropics	0	-2.2556	5.1546	4.03	0.7096	0.1622	37.60
Nectariniidae	*Nectarinia chalcomelas*	Neotropics	0	-0.4911	5.9563	2.99	0.7624	0.3608	26.05
Nectariniidae	*Nectarinia chalybea*	Neotropics	0	-0.0181	-	4.07	0.7964	0.5704	11.85
Nectariniidae	*Nectarinia chloropygia*	Neotropics	0	-0.0265	-	4.03	0.7835	0.8322	25.58
Nectariniidae	*Nectarinia coccinigaster*	Neotropics	0	0.0745	-	4.04	0.8236	0.4633	6.23
Nectariniidae	*Nectarinia congensis*	Neotropics	0	0.0904	3.0565	3.70	0.7939	0.8255	65.20
Nectariniidae	*Nectarinia cuprea*	Neotropics	0	0.2626	-	4.26	0.7836	0.3766	17.83
Nectariniidae	*Nectarinia cyanolaema*	Neotropics	0	-0.0683	0.0740	4.26	0.7060	0.2722	9.74
Nectariniidae	*Nectarinia erythrocerca*	Neotropics	0	0.4851	7.5155	3.85	0.7327	0.2211	52.09
Nectariniidae	*Nectarinia famosa*	Neotropics	0	-0.0537	-	4.02	0.8283	0.6068	10.54
Nectariniidae	*Nectarinia fuelleborni*	Neotropics	0	0.0250	-	4.02	0.8340	0.6879	7.80
Nectariniidae	*Nectarinia fuliginosa*	Neotropics	1	-0.4180	2.0143	2.81	0.7008	-	-
Nectariniidae	*Nectarinia fuliginosa*	Neotropics	1	-0.4180	2.0143	2.81	0.7008	0.2101	42.36
Nectariniidae	*Nectarinia fusca*	Neotropics	0	0.0897	1.7430	4.05	0.8254	0.4707	8.24
Nectariniidae	*Nectarinia habessinica*	Neotropics	0	0.2409	1.9819	3.19	0.7422	0.5748	64.90
Nectariniidae	*Nectarinia hunteri*	Neotropics	0	0.0378	7.8981	3.48	0.7782	0.5458	65.53
Nectariniidae	*Nectarinia johannae*	Neotropics	0	-0.1063	-	4.22	0.8014	0.5907	5.00
Nectariniidae	*Nectarinia johnstoni*	Neotropics	1	-0.5821	0.9223	3.22	0.5788	0.0000	90.00
Nectariniidae	*Nectarinia jugularis*	Neotropics	0	-3.9044	-	3.60	0.7192	0.3031	80.38
Nectariniidae	*Nectarinia kilimensis*	Neotropics	0	-0.1503	1.2924	4.04	0.7872	0.4438	14.95
Nectariniidae	*Nectarinia lotenia*	Neotropics	0	-0.4228	1.3708	2.60	0.7179	0.1598	41.05
Nectariniidae	*Nectarinia loveridgei*	Neotropics	1	-0.1148	0.2861	3.01	0.7151	0.1675	73.38
Nectariniidae	*Nectarinia manoensis*	Neotropics	0	-0.1944	-	3.66	0.7474	0.1940	65.56
Nectariniidae	*Nectarinia mariquensis*	Afrotropics	0	-0.1426	-	3.04	0.7291	0.1531	75.96
Nectariniidae	*Nectarinia mediocris*	Australasia	0	-0.2526	-	2.71	0.7078	0.4637	55.06
Nectariniidae	*Nectarinia minima*	Australasia	0	1.1935	6.6603	2.73	0.6891	0.4931	70.63
Nectariniidae	*Nectarinia minulla*	Australasia	0	-0.0895	-	2.80	0.7296	0.1871	28.06
Nectariniidae	*Nectarinia moreaui*	Australasia	0	0.3797	-	2.48	0.6370	0.1926	10.82
Nectariniidae	*Nectarinia neergardi*	Australasia	0	-0.0015	-	2.58	0.6302	0.1134	34.50
Nectariniidae	*Nectarinia notata*	Australasia	0	-0.1422	-	2.47	0.5788	0.0000	42.74
Nectariniidae	*Nectarinia olivacea*	Australasia	0	0.0489	-	2.71	0.7158	0.4968	56.93
Nectariniidae	*Nectarinia oritis*	Australasia	0	-0.0594	-	2.76	0.7586	0.2380	18.45
Nectariniidae	*Nectarinia osea*	Australasia	0	0.5421	-	2.60	0.6120	0.4082	18.43
Nectariniidae	*Nectarinia oustaleti*	Australasia	0	-0.0851	-	2.76	0.7683	0.2818	19.42
Nectariniidae	*Nectarinia preussi*	Australasia	0	-0.0210	-	3.02	0.7645	0.4085	23.73
Nectariniidae	*Nectarinia pulchella*	Australasia	0	0.0351	-	2.49	0.6662	0.3153	20.62
Nectariniidae	*Nectarinia regia*	Australasia	0	-0.7210	-	2.50	0.5788	0.0000	45.57
Nectariniidae	*Nectarinia reichenbachii*	Australasia	0	0.2257	-	2.46	0.5788	-	-
Nectariniidae	*Nectarinia reichenbachii*	Australasia	0	0.2257	-	2.46	0.5788	0.0000	0.00
Nectariniidae	*Nectarinia reichenowi*	Australasia	0	-0.0982	-	2.78	0.7567	0.2636	24.12
Nectariniidae	*Nectarinia rockefelleri*	Australasia	0	-0.2287	2.6768	3.00	0.7783	0.5278	41.03
Nectariniidae	*Nectarinia rubescens*	Australasia	0	-0.4843	-	2.52	0.5788	0.0000	90.00

Family	Species	Realm	Threat	Latitude	Elevation	Anomaly	Size	Shape	Orientation
Nectariniidae	*Nectarinia rufipennis*	Australasia	0	-0.0575	-	2.80	0.7461	0.1760	24.46
Nectariniidae	*Nectarinia seimundi*	Australasia	0	-0.5604	-	3.24	0.7792	0.3660	79.85
Nectariniidae	*Nectarinia solaris*	Australasia	0	0.1124	4.5518	2.56	0.6523	0.1875	30.24
Nectariniidae	*Nectarinia sovimanga*	Australasia	0	0.3595	-	2.48	0.6428	0.1451	5.06
Nectariniidae	*Nectarinia sperata*	IndoMalay	0	0.1104	10.6556	3.85	0.7367	0.1911	6.28
Nectariniidae	*Nectarinia superba*	Afrotropics	0	-0.6077	3.5082	3.72	0.7314	0.2386	71.25
Nectariniidae	*Nectarinia tacazze*	Neotropics	0	-0.4803	-	3.53	0.7438	0.2789	81.41
Nectariniidae	*Nectarinia talatala*	Neotropics	0	-9.3732	0.0000	4.18	0.7223	0.5114	48.31
Nectariniidae	*Nectarinia ursulae*	Neotropics	0	-0.1296	7.7441	4.23	0.7087	0.7712	43.70
Nectariniidae	*Nectarinia venusta*	IndoMalay	0	-0.0859	-	3.09	0.7933	0.6640	61.05
Nectariniidae	*Nectarinia veroxii*	IndoMalay	0	0.2456	-	2.88	0.6974	0.4142	45.25
Nectariniidae	*Nectarinia verticalis*	IndoMalay	0	-0.0894	-	3.03	0.7999	0.4513	63.56
Nectariniidae	*Nectarinia violacea*	Neotropics	0	0.2778	-	4.01	0.8297	0.5336	2.79
Nectariniidae	*Nectarinia zeylonica*	Afrotropics	0	-0.1234	-	3.48	0.8019	0.7963	21.32
Neosittidae	*Daphoenositta chrysoptera*	IndoMalay	0	-0.0711	-	3.11	0.8352	0.4574	28.42
Neosittidae	*Daphoenositta miranda*	Afrotropics	0	-0.0247	-	3.50	0.8591	0.6294	33.84
Numididae	*Acryllium vulturinum*	Afrotropics	0	-0.0521	-	3.33	0.7940	0.5469	52.48
Numididae	*Agelastes meleagrides*	Neotropics	1	0.5459	-	2.41	0.5788	0.0000	0.00
Numididae	*Agelastes niger*	Afrotropics	1	0.1732	-	3.06	0.7078	0.2673	8.40
Numididae	*Guttera plumifera*	Neotropics	0	0.5395	5.9266	3.11	0.6562	0.2175	75.59
Numididae	*Guttera pucherani*	Neotropics	1	-2.4335	2.0702	3.23	0.6598	0.3702	68.30
Numididae	*Numida meleagris*	IndoMalay	0	-0.3985	5.9643	3.31	0.7982	0.5008	1.72
Nyctibiidae	*Nyctibius aethereus*	IndoMalay	0	-0.1060	-	3.31	0.7985	0.3784	20.91
Nyctibiidae	*Nyctibius bracteatus*	IndoMalay	0	-0.0418	11.2173	2.77	0.7198	0.2877	57.01
Nyctibiidae	*Nyctibius grandis*	Palearctic	0	-0.4167	-	3.64	0.8107	0.3980	2.63
Nyctibiidae	*Nyctibius griseus*	IndoMalay	0	-0.3153	-	3.41	0.7771	0.3340	7.71
Nyctibiidae	*Nyctibius jamaicensis*	Australasia	0	-0.3121	-	3.24	0.8126	0.7510	32.15
Nyctibiidae	*Nyctibius leucopterus*	Australasia	1	-0.1197	1.5524	2.68	0.6891	0.3818	37.75
Nyctibiidae	*Nyctibius maculosus*	Australasia	0	-0.3619	4.7039	2.48	0.6221	0.0384	35.15
Odontophoridae	*Callipepla californica*	Palearctic	0	-1.0367	-	6.81	0.8015	0.0818	2.15
Odontophoridae	*Callipepla douglasii*	Nearctic	0	-0.3825	-	4.59	0.8369	0.1656	2.56
Odontophoridae	*Callipepla gambelii*	Palearctic	0	-1.1694	0.6333	5.97	0.7409	0.0109	0.00
Odontophoridae	*Callipepla squamata*	Nearctic	0	-0.6419	-	5.53	0.8129	0.1482	4.48
Odontophoridae	*Colinus cristatus*	Neotropics	0	-0.1972	-	3.76	0.8490	0.5003	38.81
Odontophoridae	*Colinus nigrogularis*	Neotropics	0	-0.1937	12.0401	3.72	0.7300	0.1877	56.68
Odontophoridae	*Colinus virginianus*	Nearctic	0	-0.7448	10.4153	4.18	0.8575	0.4563	17.21
Odontophoridae	*Cyrtonyx montezumae*	IndoMalay	0	-0.0063	1.9717	2.72	0.7715	0.4794	31.29
Odontophoridae	*Cyrtonyx ocellatus*	Australasia	1	-0.0929	5.9332	2.56	0.6370	0.2051	8.15
Odontophoridae	*Dactylortyx thoracicus*	Neotropics	0	-0.2947	-	3.86	0.8031	0.7240	35.82
Odontophoridae	*Dendrortyx barbatus*	Nearctic	0	-0.6865	8.5583	3.29	0.7510	0.3253	70.58
Odontophoridae	*Dendrortyx leucophrys*	Nearctic	0	-0.5560	-	4.41	0.8191	0.1567	11.58
Odontophoridae	*Dendrortyx macroura*	Nearctic	0	-0.7656	-	4.42	0.8111	0.3170	11.89
Odontophoridae	*Odontophorus atrifrons*	Neotropics	0	0.0674	8.8299	3.38	0.7296	0.1593	57.69
Odontophoridae	*Odontophorus balliviani*	Neotropics	0	-1.4244	5.9620	4.00	0.7464	0.1716	39.72
Odontophoridae	*Odontophorus capueira*	Neotropics	0	0.2474	-	3.99	0.8028	0.4380	16.85
Odontophoridae	*Odontophorus columbianus*	Neotropics	0	-0.5712	5.5289	3.05	0.7882	0.3288	74.24
Odontophoridae	*Odontophorus dialeucos*	Neotropics	0	-0.0258	5.4273	3.31	0.6948	0.1735	83.19
Odontophoridae	*Odontophorus erythrops*	Neotropics	0	4.3685	13.7583	4.07	0.7755	0.3445	57.82
Odontophoridae	*Odontophorus gujanensis*	Neotropics	0	-0.2568	13.6108	3.73	0.7778	0.3407	67.08
Odontophoridae	*Odontophorus guttatus*	Neotropics	0	-0.5736	7.2954	2.70	0.7165	0.1042	76.84
Odontophoridae	*Odontophorus hyperythrus*	Neotropics	1	-40.8182	7.9053	4.12	0.7019	0.3048	28.78
Odontophoridae	*Odontophorus leucolaemus*	Neotropics	0	-0.0798	2.2709	3.66	0.7928	0.3665	22.33
Odontophoridae	*Odontophorus melanonotus*	Neotropics	0	-0.5541	2.0874	3.52	0.7165	0.9254	52.94
Odontophoridae	*Odontophorus melanotis*	Neotropics	0	-0.2166	16.5174	3.15	0.7862	0.2114	85.33
Odontophoridae	*Odontophorus speciosus*	Neotropics	0	-0.4601	6.7636	3.11	0.8204	0.3572	45.52
Odontophoridae	*Odontophorus stellatus*	Neotropics	1	-0.3497	3.8602	3.72	0.7982	0.5788	22.09
Odontophoridae	*Odontophorus strophium*	Neotropics	0	0.0069	7.7015	3.74	0.7472	0.4282	74.13
Odontophoridae	*Oreortyx pictus*	Neotropics	1	-0.3058	10.3998	3.66	0.6562	0.2208	80.18
Odontophoridae	*Philortyx fasciatus*	Neotropics	0	-0.1205	-	3.33	0.7464	0.2815	28.30
Odontophoridae	*Rhynchortyx cinctus*	Australasia	0	-0.2590	3.0776	2.52	0.6478	0.3065	45.21
Opisthocomidae	*Opisthocomus hoazin*	Neotropics	0	-0.3905	15.8946	3.57	0.7861	0.1824	85.19
Oriolidae	*Oriolus albiloris*	Neotropics	0	-5.6444	3.8598	3.89	0.7435	0.1990	46.78
Oriolidae	*Oriolus auratus*	Neotropics	0	0.0881	-	4.15	0.7992	0.5962	9.46
Oriolidae	*Oriolus bouroensis*	Neotropics	1	8.6372	1.8775	3.57	0.6763	0.3583	57.50
Oriolidae	*Oriolus brachyrhynchus*	Neotropics	0	0.0135	6.3334	3.58	0.7413	0.2550	73.96
Oriolidae	*Oriolus chinensis*	Neotropics	0	0.0108	-	4.24	0.7836	0.6354	51.14
Oriolidae	*Oriolus chlorocephalus*	Australasia	0	-0.0906	-	2.78	0.7692	0.3088	24.28
Oriolidae	*Oriolus cruentus*	Afrotropics	0	-0.2112	7.1086	3.72	0.8475	0.9114	52.70
Oriolidae	*Oriolus flavocinctus*	Palearctic	0	-0.3484	12.5155	4.17	0.7912	0.4497	13.28
Oriolidae	*Oriolus forsteni*	Afrotropics	0	-0.4474	1.2456	3.39	0.7422	0.4086	72.02
Oriolidae	*Oriolus hosii*	Afrotropics	0	0.0057	14.8409	3.61	0.8298	0.2175	5.80
Oriolidae	*Oriolus isabellae*	Palearctic	0	-1.0823	14.8567	4.15	0.7670	0.1802	2.70
Oriolidae	*Oriolus larvatus*	Palearctic	0	0.2317	0.0000	3.26	0.5985	0.0000	0.00
Oriolidae	*Oriolus melanotis*	Palearctic	0	-0.8011	10.4592	4.27	0.8431	0.1338	7.48

Family	Species	Realm	Threat	Latitude	Elevation	Anomaly	Size	Shape	Orientation
Oriolidae	*Oriolus mellianus*	Palearctic	0	-0.7962	2.5976	4.10	0.8062	0.2328	2.98
Oriolidae	*Oriolus monacha*	Palearctic	0	-0.9068	0.0000	3.89	0.8214	0.2079	0.33
Oriolidae	*Oriolus nigripennis*	Palearctic	0	-1.0851	9.4357	4.32	0.8508	0.2428	3.37
Oriolidae	*Oriolus oriolus*	Palearctic	0	-0.4065	12.9201	4.09	0.8337	0.2873	1.49
Oriolidae	*Oriolus percivali*	Palearctic	0	-0.3382	-	3.73	0.7936	0.5432	22.97
Oriolidae	*Oriolus phaeochromus*	Palearctic	0	-0.2548	16.4514	3.93	0.8095	0.4459	7.92
Oriolidae	*Oriolus sagittatus*	Afrotropics	0	0.2707	11.2011	3.74	0.7554	0.4926	7.37
Oriolidae	*Oriolus steerii*	Palearctic	0	-0.2171	-	3.74	0.7869	0.1534	1.07
Oriolidae	*Oriolus szalayi*	Palearctic	0	-0.2149	5.7397	4.03	0.7857	0.3499	0.21
Oriolidae	*Oriolus tenuirostris*	Afrotropics	0	-0.3093	-	3.79	0.7929	0.5367	41.46
Oriolidae	*Oriolus traillii*	Palearctic	0	-0.8730	6.9288	4.75	0.8765	0.1483	0.90
Oriolidae	*Oriolus xanthonotus*	Afrotropics	0	-0.1480	-	3.40	0.7632	0.6273	26.98
Oriolidae	*Oriolus xanthornus*	Palearctic	0	-0.7991	-	4.23	0.8050	0.6954	9.94
Oriolidae	*Sphecotheres vieilloti*	Neotropics	0	-0.0763	3.8839	3.14	0.7296	0.2636	39.96
Oriolidae	*Sphecotheres viridis*	Australasia	0	-0.0986	0.2991	2.77	0.7560	0.3001	24.39
Orthonychidae	*Ifrita kowaldi*	IndoMalay	0	-0.0561	3.6360	3.03	0.8153	0.4251	46.55
Orthonychidae	*Melampitta gigantea*	IndoMalay	0	-0.0576	-	3.22	0.8321	0.4661	27.03
Orthonychidae	*Melampitta lugubris*	IndoMalay	0	0.0260	2.7071	2.84	0.7830	0.4460	5.05
Orthonychidae	*Orthonyx novaeguineae*	Neotropics	1	0.3882	3.1499	3.08	0.6921	0.3625	84.00
Orthonychidae	*Orthonyx spaldingii*	Neotropics	1	-0.2991	2.7808	2.91	0.7367	0.2110	35.54
Orthonychidae	*Orthonyx temminckii*	IndoMalay	1	0.0000	4.4758	3.88	0.6120	0.8772	90.00
Otididae	*Ardeotis arabs*	Afrotropics	0	-0.3342	7.3437	3.59	0.8657	0.8318	75.28
Otididae	*Ardeotis australis*	Afrotropics	0	-0.0419	-	3.65	0.8311	0.8815	25.34
Otididae	*Ardeotis kori*	IndoMalay	0	0.0087	-	2.80	0.7747	0.6953	40.88
Otididae	*Ardeotis nigriceps*	Afrotropics	0	0.0273	1.0121	3.72	0.8159	0.1728	2.80
Otididae	*Chlamydotis undulata*	Australasia	0	-0.5814	0.0000	3.37	0.8321	0.5009	7.27
Otididae	*Eupodotis afra*	Neotropics	0	-0.3752	-	3.32	0.7914	0.6741	42.22
Otididae	*Eupodotis afraoides*	Neotropics	0	0.0237	2.5872	4.06	0.7773	0.5847	5.33
Otididae	*Eupodotis caerulescens*	Neotropics	0	0.1540	-	4.08	0.8241	0.8144	27.16
Otididae	*Eupodotis gindiana*	Neotropics	0	0.0186	-	3.06	0.7151	0.2375	64.75
Otididae	*Eupodotis hartlaubii*	Neotropics	0	-0.4928	-	3.98	0.7660	0.3140	8.57
Otididae	*Eupodotis humilis*	Neotropics	0	-0.1976	-	3.76	0.8279	0.4672	61.30
Otididae	*Eupodotis melanogaster*	Neotropics	0	-0.0922	-	3.90	0.8220	0.5497	32.60
Otididae	*Eupodotis rueppellii*	Afrotropics	0	-0.1849	-	3.61	0.8273	0.5210	41.99
Otididae	*Eupodotis ruficrista*	Afrotropics	0	-0.1045	-	3.58	0.8171	0.5450	74.66
Otididae	*Eupodotis savilei*	Afrotropics	0	-0.0644	-	3.50	0.8280	0.8136	52.53
Otididae	*Eupodotis senegalensis*	Afrotropics	0	-0.0207	1.0627	3.27	0.6859	0.3272	88.40
Otididae	*Eupodotis vigorsii*	Afrotropics	0	-0.0802	-	3.51	0.8182	0.8016	85.38
Otididae	*Houbaropsis bengalensis*	IndoMalay	0	-0.0926	-	2.94	0.8102	0.5260	44.20
Otididae	*Neotis denhami*	Afrotropics	0	-0.0732	-	3.18	0.7614	0.3874	70.15
Otididae	*Neotis heuglinii*	Afrotropics	0	-0.0577	-	3.34	0.8335	0.4340	24.25
Otididae	*Neotis ludwigii*	Afrotropics	0	0.7976	-	3.22	0.7030	0.5085	40.62
Otididae	*Neotis nuba*	Afrotropics	0	-0.3055	13.6636	3.71	0.7705	0.8172	59.49
Otididae	*Otis tarda*	Neotropics	0	0.0945	10.4154	3.18	0.6859	0.3031	75.15
Otididae	*Sypheotides indicus*	Neotropics	1	-0.2507	-	3.55	0.5788	0.0000	57.83
Otididae	*Tetrax tetrax*	Afrotropics	1	-0.0698	6.6742	3.18	0.7614	0.3874	70.15
Pachycephalidae	*Aleadryas rufinucha*	Palearctic	0	-0.3165	12.9141	3.81	0.7773	0.3087	3.87
Pachycephalidae	*Coracornis raveni*	IndoMalay	0	-0.6169	-	3.37	0.8268	0.5696	13.19
Pachycephalidae	*Hylocitrea bonensis*	Afrotropics	0	-0.0663	-	3.62	0.7904	0.5498	12.64
Pachycephalidae	*Pachycare flavogriseum*	Neotropics	0	-0.1414	3.3650	4.01	0.8357	0.6641	7.16
Pachycephalidae	*Pachycephala albiventris*	Neotropics	0	-0.0466	3.5513	3.49	0.7472	0.2774	28.81
Pachycephalidae	*Pachycephala arctitorquis*	Neotropics	0	-0.4366	-	3.46	0.7867	0.4236	63.31
Pachycephalidae	*Pachycephala aurea*	Neotropics	0	-0.1290	-	3.15	0.7356	0.2894	38.97
Pachycephalidae	*Pachycephala caledonica*	Neotropics	0	-0.0555	10.3095	3.31	0.7211	0.3800	68.56
Pachycephalidae	*Pachycephala griseonota*	Neotropics	1	0.3177	4.3255	3.10	0.7040	0.3461	82.45
Pachycephalidae	*Pachycephala grisola*	Neotropics	0	-0.4157	-	3.36	0.7104	0.5746	69.44
Pachycephalidae	*Pachycephala homeyeri*	Neotropics	0	-0.2161	4.6361	3.87	0.8209	0.4942	24.68
Pachycephalidae	*Pachycephala hyperythra*	Neotropics	0	0.1806	-	3.53	0.6974	0.1228	21.92
Pachycephalidae	*Pachycephala hypoxantha*	Neotropics	0	-0.0554	4.1706	4.07	0.8040	0.4823	35.66
Pachycephalidae	*Pachycephala implicata*	Neotropics	0	-0.0005	-	3.63	0.7331	0.2052	20.38
Pachycephalidae	*Pachycephala inornata*	Neotropics	0	0.1127	-	3.94	0.7591	0.3758	0.14
Pachycephalidae	*Pachycephala lanioides*	Neotropics	0	0.0181	1.9679	3.47	0.7576	0.7014	27.86
Pachycephalidae	*Pachycephala leucogastra*	Neotropics	0	-0.2043	3.9903	3.46	0.7691	0.3687	37.59
Pachycephalidae	*Pachycephala lorentzi*	Nearctic	0	-0.1813	-	3.85	0.7211	0.1760	58.52
Pachycephalidae	*Pachycephala melanura*	Neotropics	0	-0.5919	6.1028	2.78	0.7217	0.1936	35.91
Pachycephalidae	*Pachycephala meyeri*	Australasia	0	-0.0075	12.1726	2.78	0.7151	0.0784	26.63
Pachycephalidae	*Pachycephala modesta*	Australasia	0	-0.2599	-	2.99	0.6740	0.1766	72.33
Pachycephalidae	*Pachycephala monacha*	Australasia	0	-0.5660	-	3.15	0.7261	0.2418	76.81
Pachycephalidae	*Pachycephala nudigula*	Neotropics	0	-0.0175	3.7094	4.04	0.8375	0.6514	19.13
Pachycephalidae	*Pachycephala orpheus*	IndoMalay	0	-0.0765	-	2.96	0.8115	0.4632	59.36
Pachycephalidae	*Pachycephala pectoralis*	IndoMalay	0	-0.0867	-	2.66	0.7060	0.2230	75.20
Pachycephalidae	*Pachycephala phaionota*	IndoMalay	0	0.0415	-	2.63	0.6370	-	-
Pachycephalidae	*Pachycephala phaionota*	IndoMalay	0	0.0415	-	2.63	0.6370	0.4660	1.62
Pachycephalidae	*Pachycephala philippinensis*	IndoMalay	0	-0.0880	-	3.04	0.8008	0.4700	56.75

Family	Species	Realm	Threat	Latitude	Elevation	Anomaly	Size	Shape	Orientation
Pachycephalidae	*Pachycephala rufiventris*	IndoMalay	0	0.0142	-	2.65	0.6876	0.5282	67.51
Pachycephalidae	*Pachycephala rufogularis*	IndoMalay	0	-0.0434	-	2.62	0.7019	0.6235	86.79
Pachycephalidae	*Pachycephala schlegelii*	IndoMalay	0	-0.0129	-	2.65	0.6523	0.5526	23.87
Pachycephalidae	*Pachycephala simplex*	IndoMalay	0	0.0524	2.2563	2.63	0.6478	0.2778	79.83
Pachycephalidae	*Pachycephala soror*	IndoMalay	0	-0.0298	-	2.82	0.7823	0.6001	22.27
Pachycephalidae	*Pachycephala sulfuriventer*	IndoMalay	0	-0.1731	-	2.57	0.6221	0.2612	67.30
Pachycephalidae	*Rhagologus leucostigma*	IndoMalay	0	0.3845	6.1005	2.76	0.6221	0.9901	90.00
Paradisaeidae	*Astrapia mayeri*	Neotropics	0	-0.3588	9.3273	4.02	0.7217	0.1074	37.56
Paradisaeidae	*Astrapia nigra*	Neotropics	0	-65.9111	4.9363	4.13	0.6662	0.5529	58.98
Paradisaeidae	*Astrapia rothschildi*	Neotropics	0	1.8963	7.6984	4.06	0.6876	0.2466	21.15
Paradisaeidae	*Astrapia splendidissima*	Neotropics	0	0.0463	15.1135	3.87	0.7428	0.3624	82.55
Paradisaeidae	*Astrapia stephaniae*	Australasia	0	-0.7382	6.1631	2.97	0.6690	0.3577	10.57
Paradisaeidae	*Cicinnurus magnificus*	Palearctic	0	-0.4398	8.3985	3.44	0.7552	0.0928	14.00
Paradisaeidae	*Cicinnurus regius*	Palearctic	0	-0.3661	-	3.45	0.7823	0.1218	12.14
Paradisaeidae	*Cicinnurus regius*	Palearctic	0	-0.3661	-	3.45	0.7823	-	-
Paradisaeidae	*Cicinnurus respublica*	IndoMalay	1	-0.1140	-	3.70	0.7096	0.1266	2.17
Paradisaeidae	*Epimachus albertisi*	IndoMalay	0	-0.1380	-	3.46	0.8014	0.2317	11.63
Paradisaeidae	*Epimachus bruijnii*	IndoMalay	0	-0.0072	-	2.84	0.7875	0.4845	8.87
Paradisaeidae	*Epimachus fastuosus*	IndoMalay	0	-0.2218	-	3.25	0.8148	0.6323	1.45
Paradisaeidae	*Epimachus meyeri*	Palearctic	0	-0.4622	-	3.68	0.8206	0.2247	12.20
Paradisaeidae	*Lophorina superba*	Neotropics	0	-0.3161	4.0221	2.99	0.7464	0.3124	35.30
Paradisaeidae	*Lycocorax pyrrhopterus*	IndoMalay	1	0.0462	0.6185	2.84	0.7864	0.4319	0.59
Paradisaeidae	*Manucodia ater*	IndoMalay	0	-0.1767	11.4408	3.20	0.8148	0.7812	81.82
Paradisaeidae	*Manucodia chalybatus*	Afrotropics	0	0.0898	-	3.27	0.8156	0.2428	16.40
Paradisaeidae	*Manucodia jobiensis*	Afrotropics	0	0.0233	-	3.29	0.8022	0.5143	2.98
Paradisaeidae	*Manucodia keraudrenii*	Afrotropics	0	-0.0383	-	3.07	0.7597	0.1435	4.01
Paradisaeidae	*Paradigalla brevicauda*	IndoMalay	0	-0.0109	-	2.64	0.7291	0.2179	67.85
Paradisaeidae	*Paradigalla carunculata*	IndoMalay	0	0.5985	-	2.65	0.5788	0.0000	0.00
Paradisaeidae	*Paradisaea apoda*	IndoMalay	0	0.0206	7.6247	2.62	0.6221	0.2782	73.89
Paradisaeidae	*Paradisaea guilielmi*	IndoMalay	0	0.0462	-	2.84	0.7861	0.4873	0.77
Paradisaeidae	*Paradisaea minor*	Afrotropics	0	-0.0777	0.0000	3.18	0.7614	0.3874	70.15
Paradisaeidae	*Paradisaea raggiana*	IndoMalay	1	0.1023	1.6633	2.89	0.7291	0.1448	72.47
Paradisaeidae	*Paradisaea rubra*	Palearctic	0	-0.6932	7.8026	4.32	0.8458	0.2294	4.81
Paradisaeidae	*Paradisaea rudolphi*	Afrotropics	0	-0.1291	7.9898	3.61	0.8564	0.7801	27.39
Paradisaeidae	*Parotia carolae*	Australasia	0	-0.1532	3.1379	2.83	0.7781	0.7834	29.27
Paradisaeidae	*Parotia helenae*	Australasia	0	-0.0735	-	2.81	0.7480	0.1596	24.39
Paradisaeidae	*Parotia lawesii*	Australasia	0	0.0703	-	2.72	0.7096	0.4755	56.23
Paradisaeidae	*Parotia sefilata*	Australasia	0	-0.0643	-	2.81	0.7261	0.1781	22.65
Paradisaeidae	*Parotia wahnesi*	Australasia	0	-0.2526	-	2.83	0.7477	0.2186	24.49
Paradisaeidae	*Pteridophora alberti*	Palearctic	0	1.8891	-	4.44	0.7865	0.2781	27.57
Paradisaeidae	*Ptiloris intercedens*	IndoMalay	0	-0.0248	-	3.12	0.7234	0.2678	63.66
Paradisaeidae	*Ptiloris magnificus*	IndoMalay	0	-0.0963	4.3090	3.42	0.8103	0.7448	88.22
Paradisaeidae	*Ptiloris paradiseus*	Palearctic	0	-20.5878	19.1193	3.96	0.7531	0.5423	33.63
Paradisaeidae	*Ptiloris victoriae*	IndoMalay	0	-0.0743	-	3.40	0.8179	0.4097	19.60
Paradisaeidae	*Seleucidis melanoleucus*	Neotropics	0	-0.2744	5.2326	3.02	0.7746	-	-
Paradisaeidae	*Seleucidis melanoleucus*	Neotropics	0	-0.2744	5.2326	3.02	0.7746	0.3392	38.08
Pardalotidae	*Pardalotus punctatus*	Australasia	0	-0.0749	-	2.80	0.7416	0.1868	22.28
Pardalotidae	*Pardalotus quadragintus*	IndoMalay	0	0.1541	-	2.90	0.7296	0.4742	54.07
Pardalotidae	*Pardalotus rubricatus*	Australasia	0	-0.1648	4.7117	2.48	0.6120	0.0400	36.10
Pardalotidae	*Pardalotus striatus*	Australasia	0	-0.8444	-	2.97	0.7979	0.1965	7.82
Paridae	*Baeolophus atricristatus*	Palearctic	0	-0.7920	-	4.30	0.8399	0.2634	2.09
Paridae	*Baeolophus bicolor*	Nearctic	0	-0.8077	-	4.24	0.8240	0.2865	22.31
Paridae	*Baeolophus inornatus*	Palearctic	0	-2.3554	5.1866	4.48	0.7040	0.5553	67.93
Paridae	*Baeolophus ridgwayi*	Palearctic	0	-0.4761	-	3.40	0.8052	0.5579	1.19
Paridae	*Baeolophus wollweberi*	Palearctic	0	1.0780	11.1712	4.87	0.7179	0.2562	6.72
Paridae	*Melanochlora sultanea*	Australasia	0	-0.0706	1.6140	2.48	0.6428	0.4850	33.00
Paridae	*Parus afer*	Australasia	0	-0.6316	0.7722	2.45	0.7493	0.0788	3.36
Paridae	*Parus albiventris*	Australasia	0	-0.5605	-	2.48	0.7539	0.0745	3.32
Paridae	*Parus amabilis*	Australasia	0	-0.5178	0.9143	2.49	0.7266	0.0475	3.29
Paridae	*Parus ater*	Neotropics	0	-0.0622	-	3.60	0.7916	0.2560	32.28
Paridae	*Parus atricapillus*	Neotropics	0	0.1436	11.4121	3.52	0.7651	0.5251	74.97
Paridae	*Parus caeruleus*	Neotropics	0	-0.1581	-	3.83	0.8328	0.4806	33.32
Paridae	*Parus carolinensis*	Neotropics	0	-0.0612	5.1531	3.38	0.7786	0.4101	33.91
Paridae	*Parus cinctus*	Neotropics	0	0.1125	-	3.27	0.7537	0.2569	62.89
Paridae	*Parus cinerascens*	Neotropics	0	-0.0997	8.9474	3.57	0.7752	0.2703	24.40
Paridae	*Parus cristatus*	Neotropics	0	-0.1758	-	3.99	0.8343	0.7157	18.63
Paridae	*Parus cyanus*	Neotropics	0	-0.0418	-	4.04	0.8363	0.7573	0.69
Paridae	*Parus davidi*	Neotropics	0	-0.0393	-	2.47	0.5985	0.0000	0.00
Paridae	*Parus dichrous*	Neotropics	0	-0.2201	5.3136	3.76	0.8548	0.6455	47.52
Paridae	*Parus elegans*	Neotropics	0	-0.0332	-	4.00	0.8215	0.6687	22.63
Paridae	*Parus fasciiventer*	Neotropics	1	-0.2804	3.6598	3.18	0.6370	0.2805	85.47
Paridae	*Parus fringillinus*	Neotropics	0	-0.1476	-	3.98	0.7696	0.3975	34.63
Paridae	*Parus funereus*	Neotropics	0	-0.2452	-	3.67	0.8332	0.6189	38.58
Paridae	*Parus gambeli*	Neotropics	0	0.0481	10.5256	3.57	0.7654	0.4143	79.19

Family	Species	Realm	Threat	Latitude	Elevation	Anomaly	Size	Shape	Orientation
Paridae	*Parus griseiventris*	Neotropics	0	-0.1945	-	3.58	0.8292	0.7568	40.32
Paridae	*Parus holsti*	Australasia	0	-0.0094	-	2.56	0.6523	0.1875	30.24
Paridae	*Parus hudsonicus*	IndoMalay	1	-0.4123	1.2916	2.71	0.7112	0.1623	10.99
Paridae	*Parus hypermelaenus*	Palearctic	0	-0.6288	3.0239	4.41	0.8770	0.1995	4.11
Paridae	*Parus leucomelas*	Neotropics	0	-0.0431	9.1513	3.08	0.6690	0.2697	32.14
Paridae	*Parus leuconotus*	Palearctic	0	-0.8962	9.2020	4.31	0.8516	0.1691	1.78
Paridae	*Parus lugubris*	Neotropics	0	-0.1645	-	3.92	0.8428	0.5238	30.15
Paridae	*Parus major*	Neotropics	0	-0.0125	6.6045	3.64	0.7560	0.1196	20.32
Paridae	*Parus montanus*	Neotropics	0	-0.2571	5.1109	3.58	0.8459	0.3514	41.82
Paridae	*Parus monticolus*	Australasia	0	-0.3933	-	2.89	0.7251	0.1405	16.14
Paridae	*Parus niger*	Australasia	0	-28.8482	4.1258	2.68	0.6370	0.0943	33.46
Paridae	*Parus nuchalis*	Australasia	0	0.6010	-	2.88	0.7192	0.4279	23.82
Paridae	*Parus palustris*	Australasia	0	-0.1975	5.3727	2.79	0.6523	0.3096	21.97
Paridae	*Parus rubidiventris*	Australasia	0	-0.0327	-	2.71	0.7441	0.1664	14.04
Paridae	*Parus rufescens*	Australasia	0	-0.2351	-	2.85	0.7378	0.4469	19.59
Paridae	*Parus rufonuchalis*	Australasia	1	-0.0790	2.4429	2.88	0.7120	0.3283	35.88
Paridae	*Parus sclateri*	Palearctic	0	-0.4890	-	3.28	0.7374	0.3720	81.32
Paridae	*Parus semilarvatus*	IndoMalay	0	-0.0609	8.4911	3.29	0.7498	0.4400	31.83
Paridae	*Parus spilonotus*	Palearctic	0	-0.4601	7.1523	3.36	0.7396	0.3388	44.33
Paridae	*Parus superciliosus*	Palearctic	0	-0.3789	11.1857	3.67	0.7515	0.4939	27.78
Paridae	*Parus thruppi*	IndoMalay	0	-0.7393	6.9111	3.15	0.7406	0.2350	22.79
Paridae	*Parus varius*	IndoMalay	1	-0.4357	5.5105	3.37	0.6891	0.3999	30.63
Paridae	*Parus venustulus*	Palearctic	0	-0.6300	7.0380	3.75	0.7550	0.2717	11.08
Paridae	*Parus xanthogenys*	IndoMalay	0	-0.6410	7.7435	3.28	0.8057	0.4033	9.69
Paridae	*Pseudopodoces humilis*	Australasia	0	-0.2042	6.7482	2.49	0.5788	0.0000	0.00
Paridae	*Sylviparus modestus*	IndoMalay	1	-0.4373	3.8357	2.66	0.5788	0.0000	90.00
Parulidae	*Basileuterus basilicus*	Neotropics	0	0.6219	2.7340	2.94	0.6876	0.1880	60.67
Parulidae	*Basileuterus belli*	Australasia	0	-0.6572	-	3.36	0.8297	0.4527	6.29
Parulidae	*Basileuterus bivittatus*	Nearctic	0	-0.7009	-	4.33	0.8222	0.1912	20.04
Parulidae	*Basileuterus chrysogaster*	Neotropics	0	-0.0206	-	3.96	0.8172	0.4956	45.05
Parulidae	*Basileuterus cinereicollis*	Neotropics	0	-0.4003	-	3.36	0.7947	0.4426	33.74
Parulidae	*Basileuterus conspicillatus*	Neotropics	1	0.3214	5.4500	3.18	0.6120	0.6246	0.00
Parulidae	*Basileuterus coronatus*	Neotropics	0	-0.0581	10.2158	3.70	0.7619	0.2281	26.36
Parulidae	*Basileuterus culicivorus*	Neotropics	0	-0.2085	5.9948	4.13	0.7498	0.2203	83.76
Parulidae	*Basileuterus flaveolus*	Neotropics	0	-0.0727	-	3.63	0.7165	0.2014	65.40
Parulidae	*Basileuterus fraseri*	Neotropics	0	-0.0922	7.0115	3.89	0.7030	0.4302	81.75
Parulidae	*Basileuterus griseiceps*	Neotropics	0	0.0448	8.5511	3.29	0.6428	0.6755	18.16
Parulidae	*Basileuterus hypoleucus*	Neotropics	0	0.0114	7.4734	3.64	0.7697	0.4128	77.21
Parulidae	*Basileuterus ignotus*	Neotropics	0	-0.1716	-	3.65	0.8428	0.5072	42.64
Parulidae	*Basileuterus leucoblepharus*	Neotropics	0	-0.1945	-	3.85	0.8219	0.7677	2.27
Parulidae	*Basileuterus leucophrys*	Neotropics	0	0.2047	-	3.14	0.7008	0.2542	89.32
Parulidae	*Basileuterus luteoviridis*	Neotropics	1	-1.0298	8.0241	3.77	0.5985	0.0000	0.00
Parulidae	*Basileuterus melanogenys*	Neotropics	0	-0.3188	-	3.89	0.7882	0.5573	14.56
Parulidae	*Basileuterus nigrocristatus*	Neotropics	1	0.0031	2.7977	2.96	0.5788	0.0000	0.00
Parulidae	*Basileuterus rufifrons*	Neotropics	0	-0.5379	-	2.95	0.7910	0.5408	43.65
Parulidae	*Basileuterus signatus*	Neotropics	0	-0.3613	-	3.92	0.7771	0.6868	19.54
Parulidae	*Basileuterus trifasciatus*	Neotropics	0	0.0321	6.6119	3.68	0.7599	0.3512	75.33
Parulidae	*Basileuterus trifasciatus*	Neotropics	0	0.0321	6.6119	3.68	0.7599	-	-
Parulidae	*Basileuterus tristriatus*	Neotropics	0	-0.1902	6.1094	3.01	0.6690	0.2442	28.98
Parulidae	*Cardellina rubrifrons*	Neotropics	0	-0.1290	3.7156	3.67	0.8306	0.6487	54.41
Parulidae	*Dendroica adelaidae*	Palearctic	0	-0.5565	4.8566	4.58	0.8511	0.1484	1.98
Parulidae	*Dendroica caerulescens*	IndoMalay	0	-0.0610	-	3.21	0.8077	0.3664	46.35
Parulidae	*Dendroica castanea*	IndoMalay	0	-0.0233	6.0717	2.64	0.7323	0.2310	67.52
Parulidae	*Dendroica cerulea*	IndoMalay	0	-0.1048	-	3.44	0.8130	0.6347	21.84
Parulidae	*Dendroica chrysoparia*	Palearctic	0	-0.6173	-	4.48	0.8700	0.2439	1.45
Parulidae	*Dendroica coronata*	Palearctic	0	-0.8022	-	3.98	0.8240	0.4061	8.41
Parulidae	*Dendroica discolor*	Palearctic	0	-0.4907	-	4.66	0.8638	0.1673	0.90
Parulidae	*Dendroica dominica*	IndoMalay	0	0.0127	-	2.79	0.7758	0.6110	8.67
Parulidae	*Dendroica fusca*	IndoMalay	0	-0.0730	-	3.39	0.8066	0.6679	76.50
Parulidae	*Dendroica graciae*	Afrotropics	0	0.0948	11.7499	3.47	0.8335	0.2216	6.09
Parulidae	*Dendroica kirtlandii*	Palearctic	0	-0.9133	-	4.09	0.8148	0.3296	24.90
Parulidae	*Dendroica magnolia*	Australasia	0	-0.0278	-	2.71	0.7185	0.5623	60.71
Parulidae	*Dendroica nigrescens*	Neotropics	1	-0.2513	-	2.51	0.7104	0.1781	16.91
Parulidae	*Dendroica occidentalis*	Australasia	0	-0.2128	-	3.18	0.8282	0.4959	31.99
Parulidae	*Dendroica palmarum*	Neotropics	0	-0.1732	-	3.83	0.8458	0.3816	37.81
Parulidae	*Dendroica pensylvanica*	Afrotropics	0	-0.2508	3.2631	3.46	0.8637	0.3498	4.96
Parulidae	*Dendroica petechia*	Australasia	0	-0.5080	0.0000	3.46	0.8266	0.4938	17.83
Parulidae	*Dendroica pharetra*	Australasia	0	0.0156	-	2.73	0.7799	0.3361	24.55
Parulidae	*Dendroica pinus*	IndoMalay	0	-0.2155	-	3.22	0.8374	0.6051	8.98
Parulidae	*Dendroica pityophila*	Afrotropics	0	-0.1488	3.4942	3.63	0.8765	0.3207	3.92
Parulidae	*Dendroica striata*	Neotropics	0	-0.0116	2.2755	2.41	0.5788	0.0000	0.00
Parulidae	*Dendroica tigrina*	Nearctic	0	-0.9185	-	4.26	0.7913	0.4973	3.85
Parulidae	*Dendroica townsendi*	Nearctic	0	-0.5138	-	4.48	0.8130	0.1334	14.92
Parulidae	*Dendroica virens*	Nearctic	1	-0.8867	7.7555	4.33	0.7950	0.5006	8.03

Family	Species	Realm	Threat	Latitude	Elevation	Anomaly	Size	Shape	Orientation
Parulidae	*Ergaticus ruber*	Australasia	0	-0.7620	-	3.37	0.8007	-	-
Parulidae	*Ergaticus ruber*	Australasia	0	-0.7620	-	3.37	0.8007	0.3976	5.34
Parulidae	*Ergaticus versicolor*	Palearctic	0	-0.4205	4.8580	4.10	0.8126	-	-
Parulidae	*Ergaticus versicolor*	Palearctic	0	-0.4205	4.8580	4.10	0.8126	0.2023	5.18
Parulidae	*Euthlypis lachrymosa*	IndoMalay	1	-0.0190	-	3.16	0.7788	0.6562	78.27
Parulidae	*Geothlypis aequinoctialis*	Nearctic	0	-0.5714	1.8380	4.32	0.8453	0.2095	5.13
Parulidae	*Geothlypis aequinoctialis*	Nearctic	0	-0.5714	1.8380	4.32	0.8453	-	-
Parulidae	*Geothlypis beldingi*	Nearctic	0	-0.5861	1.3770	5.09	0.8307	-	-
Parulidae	*Geothlypis beldingi*	Nearctic	0	-0.5861	1.3770	5.09	0.8307	0.0455	0.68
Parulidae	*Geothlypis flavovelata*	Palearctic	0	-0.7416	1.3957	5.25	0.8683	0.0710	0.96
Parulidae	*Geothlypis nelsoni*	IndoMalay	0	-0.1855	-	3.11	0.7812	0.5332	25.34
Parulidae	*Geothlypis poliocephala*	IndoMalay	0	0.0089	4.4104	3.03	0.7452	0.2599	79.62
Parulidae	*Geothlypis rostrata*	Neotropics	0	-13.1252	9.1858	3.78	0.7205	0.1804	59.66
Parulidae	*Geothlypis semiflava*	Nearctic	0	-0.5971	-	4.06	0.8142	0.6375	13.91
Parulidae	*Geothlypis speciosa*	Neotropics	0	-0.0797	9.8067	3.57	0.7684	-	-
Parulidae	*Geothlypis speciosa*	Neotropics	0	-0.0797	9.8067	3.57	0.7684	0.2553	23.75
Parulidae	*Geothlypis trichas*	Afrotropics	0	-0.5182	9.9290	3.47	0.7736	0.4642	29.41
Parulidae	*Helmitheros vermivorum*	Australasia	0	0.1391	-	2.63	0.6478	0.0000	0.00
Parulidae	*Icteria virens*	Neotropics	1	-1.7481	2.9776	3.72	0.6221	0.3002	11.66
Parulidae	*Limnothlypis swainsonii*	Neotropics	0	-0.6796	2.9324	3.14	0.6740	0.4567	84.74
Parulidae	*Microligea palustris*	Afrotropics	0	0.1883	-	3.28	0.7983	0.6098	4.13
Parulidae	*Mniotilta varia*	IndoMalay	0	-0.0866	5.7890	2.64	0.6784	0.9780	56.45
Parulidae	*Myioborus albifacies*	Afrotropics	0	-1.0542	-	3.15	0.7425	0.0960	8.05
Parulidae	*Myioborus albifrons*	Afrotropics	0	0.2006	-	3.15	0.7650	0.2194	6.16
Parulidae	*Myioborus brunniceps*	IndoMalay	0	0.0129	-	3.21	0.7868	0.4047	56.55
Parulidae	*Myioborus brunniceps*	IndoMalay	0	0.0129	-	3.21	0.7868	-	-
Parulidae	*Myioborus cardonai*	Neotropics	0	-0.1566	-	3.53	0.7340	0.1638	58.91
Parulidae	*Myioborus castaneocapilla*	Neotropics	0	-0.4415	-	2.97	0.7766	0.3335	32.98
Parulidae	*Myioborus flavivertex*	Neotropics	0	-2.8391	9.9380	4.07	0.7495	0.2417	35.40
Parulidae	*Myioborus melanocephalus*	Neotropics	0	-0.9360	5.6735	2.88	0.7558	0.1115	81.29
Parulidae	*Myioborus miniatus*	Neotropics	0	-0.0511	8.5342	3.34	0.7069	0.1328	53.14
Parulidae	*Myioborus ornatus*	Neotropics	0	-0.8830	11.3403	1.95	0.7403	0.7809	74.09
Parulidae	*Myioborus pariae*	Neotropics	0	-0.5240	14.8290	3.89	0.7716	0.2615	82.50
Parulidae	*Myioborus pictus*	Neotropics	0	-0.2227	15.9680	3.08	0.7779	0.1113	84.80
Parulidae	*Myioborus torquatus*	Neotropics	0	-0.1334	-	4.12	0.7919	0.6776	27.95
Parulidae	*Oporornis agilis*	Neotropics	0	0.0218	8.0461	3.48	0.7438	0.2257	57.50
Parulidae	*Oporornis formosus*	Neotropics	0	0.0296	13.1601	3.69	0.7552	0.3606	70.32
Parulidae	*Oporornis philadelphia*	Neotropics	0	0.0345	11.4527	3.61	0.7623	0.4049	80.16
Parulidae	*Oporornis tolmiei*	Neotropics	0	0.4509	11.4024	3.56	0.7144	0.2166	62.07
Parulidae	*Parkesia motacilla*	Australasia	0	0.0319	2.8830	2.50	0.6428	0.5161	33.02
Parulidae	*Parkesia noveboracensis*	Australasia	0	-0.2027	13.4342	2.85	0.7223	0.2792	32.97
Parulidae	*Parula americana*	Afrotropics	0	-0.0303	-	3.53	0.8268	0.4723	33.39
Parulidae	*Parula gutturalis*	Neotropics	0	-0.4700	0.7443	2.31	0.7766	0.0780	1.74
Parulidae	*Parula pitiayumi*	Australasia	0	-0.7153	0.0000	2.33	0.7087	0.1846	6.50
Parulidae	*Parula superciliosa*	Neotropics	0	-0.4434	0.7318	2.30	0.7790	0.0853	1.04
Parulidae	*Phaeothlypis fulvicauda*	Neotropics	0	-0.0641	-	3.89	0.7866	0.3635	5.10
Parulidae	*Phaeothlypis rivularis*	IndoMalay	0	-0.0827	-	3.50	0.7875	0.4438	84.98
Parulidae	*Protonotaria citrea*	Neotropics	0	-0.6584	9.4034	3.02	0.7008	0.2938	28.43
Parulidae	*Seiurus aurocapilla*	Neotropics	0	0.0409	-	3.99	0.8021	0.6554	9.16
Parulidae	*Setophaga ruticilla*	Neotropics	0	0.0232	-	4.02	0.8332	0.7136	6.33
Parulidae	*Teretistris fernandinae*	Neotropics	0	-0.1563	-	3.26	0.7069	0.3752	70.99
Parulidae	*Teretistris fornsi*	Neotropics	0	-18.4940	6.5662	3.69	0.6631	0.3165	45.13
Parulidae	*Vermivora bachmanii*	Neotropics	0	0.1352	7.8505	3.56	0.7266	0.1393	61.45
Parulidae	*Vermivora celata*	Neotropics	0	-0.0945	-	3.94	0.8231	0.4500	40.42
Parulidae	*Vermivora chrysoptera*	Neotropics	0	0.0442	14.7061	3.64	0.7715	0.4123	81.48
Parulidae	*Vermivora crissalis*	Neotropics	0	-0.2090	5.2399	3.39	0.7843	0.2238	31.03
Parulidae	*Vermivora cyanoptera*	Neotropics	0	-1.7105	0.6543	4.06	0.7331	0.5360	2.00
Parulidae	*Vermivora luciae*	Neotropics	0	0.0617	-	4.05	0.8099	0.5481	45.26
Parulidae	*Vermivora peregrina*	Afrotropics	1	0.0000	3.1681	3.07	0.5788	0.0000	45.00
Parulidae	*Vermivora ruficapilla*	Palearctic	0	-0.7911	-	4.49	0.7939	0.8948	81.92
Parulidae	*Vermivora virginiae*	Neotropics	0	0.0759	5.9869	4.04	0.8194	0.9374	16.50
Parulidae	*Wilsonia canadensis*	IndoMalay	0	-0.6935	5.7691	3.48	0.8442	0.3633	22.10
Parulidae	*Wilsonia citrina*	Australasia	0	-0.5905	5.1926	3.11	0.8054	0.5368	10.29
Parulidae	*Wilsonia pusilla*	Australasia	0	-0.5879	3.7646	3.37	0.8359	0.5161	7.04
Parulidae	*Xenoligea montana*	Neotropics	0	-0.0577	-	4.03	0.8346	0.6775	15.17
Parulidae	*Zeledonia coronata*	Afrotropics	0	0.0234	-	3.42	0.8396	0.3889	32.41
Passeridae	*Histurgops ruficaudus*	IndoMalay	0	-0.0248	7.8632	2.63	0.7352	0.2773	78.30
Passeridae	*Montifringilla adamsi*	Afrotropics	0	-0.0843	8.3309	3.18	0.7614	-	-
Passeridae	*Montifringilla adamsi*	Afrotropics	0	-0.0843	8.3309	3.18	0.7614	0.3874	70.15
Passeridae	*Montifringilla blanfordi*	Afrotropics	0	0.0304	-	3.26	0.7659	0.7510	86.54
Passeridae	*Montifringilla davidiana*	Australasia	0	-0.2803	-	3.26	0.8357	0.3171	49.46
Passeridae	*Montifringilla nivalis*	IndoMalay	0	-0.4337	-	3.19	0.7078	0.4285	77.60
Passeridae	*Montifringilla nivalis*	IndoMalay	0	-0.4337	-	3.19	0.7078	-	-
Passeridae	*Montifringilla ruficollis*	Afrotropics	0	-0.2952	-	4.16	0.8014	0.5605	15.02

Family	Species	Realm	Threat	Latitude	Elevation	Anomaly	Size	Shape	Orientation
Passeridae	*Montifringilla taczanowskii*	Afrotropics	0	0.0029	-	3.22	0.7727	0.8135	9.03
Passeridae	*Montifringilla theresae*	Afrotropics	0	-7.3958	-	3.23	0.6997	0.1954	81.90
Passeridae	*Passer ammodendri*	IndoMalay	0	-0.6014	-	3.27	0.7977	0.4720	11.81
Passeridae	*Passer castanopterus*	Palearctic	0	-0.4535	2.0897	4.17	0.7693	0.5391	59.55
Passeridae	*Passer cordofanicus*	IndoMalay	0	0.1862	3.7293	2.97	0.6428	0.4562	0.00
Passeridae	*Passer diffusus*	IndoMalay	0	0.0014	10.7060	3.48	0.7689	0.3425	33.05
Passeridae	*Passer domesticus*	Palearctic	0	0.5282	10.9130	3.56	0.7367	0.4945	20.05
Passeridae	*Passer eminibey*	Palearctic	1	-16.8908	3.6194	3.74	0.6859	0.6868	12.60
Passeridae	*Passer euchlorus*	IndoMalay	0	0.0354	7.6125	3.35	0.7613	0.3823	28.34
Passeridae	*Passer flaveolus*	Palearctic	0	-0.5204	9.6247	3.73	0.7409	0.2352	4.16
Passeridae	*Passer gongonensis*	Palearctic	0	-0.6164	10.6140	3.28	0.7684	0.7068	23.12
Passeridae	*Passer griseus*	Palearctic	0	-0.7878	-	3.61	0.8204	0.4912	36.27
Passeridae	*Passer hispaniolensis*	Palearctic	1	-0.5680	6.4108	3.28	0.6974	0.4729	43.61
Passeridae	*Passer insularis*	Australasia	0	-0.1115	11.5099	2.82	0.7296	0.1543	24.53
Passeridae	*Passer luteus*	Australasia	0	-0.5707	6.6960	3.01	0.8050	0.4207	5.33
Passeridae	*Passer melanurus*	Australasia	1	-0.3058	3.1396	2.50	0.6221	0.3193	76.17
Passeridae	*Passer moabiticus*	Australasia	0	-0.4137	-	3.50	0.8286	0.4820	0.42
Passeridae	*Passer montanus*	Australasia	0	-0.6154	6.7307	3.14	0.8159	0.6584	13.97
Passeridae	*Passer motitensis*	Neotropics	0	-0.2392	-	3.00	0.8072	0.3701	51.69
Passeridae	*Passer pyrrhonotus*	Neotropics	0	-0.1603	7.8924	3.59	0.8171	0.4815	20.99
Passeridae	*Passer rufocinctus*	Neotropics	0	-0.4497	-	2.90	0.8260	0.6956	81.15
Passeridae	*Passer rutilans*	Neotropics	0	0.1171	3.6439	4.11	0.8015	0.4086	4.30
Passeridae	*Passer shelleyi*	Nearctic	0	-0.8501	0.0000	4.20	0.8092	0.5413	14.54
Passeridae	*Passer simplex*	Nearctic	0	-0.6246	0.0000	4.28	0.8410	0.1640	10.51
Passeridae	*Passer suahelicus*	Afrotropics	0	-4.6855	4.1682	3.33	0.7582	0.4388	1.12
Passeridae	*Passer swainsonii*	Afrotropics	0	0.2625	-	3.20	0.7631	0.4844	9.84
Passeridae	*Petronia brachydactyla*	IndoMalay	0	-0.0606	-	2.92	0.7296	0.2624	43.55
Passeridae	*Petronia dentata*	IndoMalay	0	-0.0528	-	3.03	0.8219	0.3794	26.82
Passeridae	*Petronia petronia*	Australasia	0	-0.1880	3.1801	2.71	0.7185	0.5623	60.71
Passeridae	*Petronia xanthocollis*	Australasia	0	-0.5208	-	3.32	0.8304	0.5921	13.52
Passeridae	*Philetairus socius*	Neotropics	0	-0.5262	-	2.46	0.6221	0.0000	0.00
Passeridae	*Plocepasser donaldsoni*	Neotropics	1	-0.5203	7.9444	2.79	0.7444	0.4398	26.17
Passeridae	*Plocepasser mahali*	Neotropics	0	-0.0434	5.5815	3.70	0.7695	0.2024	31.80
Passeridae	*Plocepasser rufoscapulatus*	Neotropics	0	-0.1504	9.2878	3.77	0.7505	0.2102	44.14
Passeridae	*Plocepasser superciliosus*	Neotropics	0	-0.2613	7.2193	3.66	0.8426	0.3088	43.43
Passeridae	*Pseudonigrita arnaudi*	Australasia	0	0.3164	3.8313	2.65	0.6784	0.5032	25.70
Passeridae	*Pseudonigrita cabanisi*	IndoMalay	1	-0.0056	3.4891	2.66	0.6997	0.4736	75.00
Passeridae	*Sporopipes frontalis*	IndoMalay	1	0.1102	1.8390	2.86	0.7760	0.4171	9.33
Passeridae	*Sporopipes squamifrons*	Afrotropics	0	4.2143	6.5186	3.35	0.7323	0.6092	41.67
Pedionomidae	*Pedionomus torquatus*	Afrotropics	0	-25.4775	9.5575	3.45	0.7050	0.3589	82.68
Pelecanidae	*Pelecanus conspicillatus*	Afrotropics	0	-0.1783	6.2111	3.74	0.7984	0.7039	4.31
Pelecanidae	*Pelecanus crispus*	IndoMalay	0	-0.9050	8.7642	2.61	0.6478	0.3444	72.46
Pelecanidae	*Pelecanus erythrorhynchos*	Nearctic	0	-0.6166	6.3129	4.32	0.8387	0.1417	9.72
Pelecanidae	*Pelecanus philippensis*	Palearctic	0	-0.0658	16.3690	3.72	0.7697	0.5287	14.53
Pelecanidae	*Pelecanus rufescens*	Palearctic	0	-0.6147	10.1757	4.11	0.8460	0.4469	8.76
Petroicidae	*Amalocichla incerta*	Afrotropics	0	-0.1417	-	3.65	0.8272	0.6025	33.28
Petroicidae	*Amalocichla sclateriana*	Australasia	0	-0.2616	-	2.84	0.7403	0.1953	23.76
Petroicidae	*Drymodes brunneopygia*	Neotropics	0	-0.7103	7.0489	2.81	0.7950	0.4164	83.23
Petroicidae	*Drymodes superciliaris*	Neotropics	0	-4.8516	9.3052	4.03	0.7472	0.2677	34.66
Petroicidae	*Eopsaltria australis*	Nearctic	0	-0.5637	13.8897	4.12	0.8109	0.2424	60.80
Petroicidae	*Eopsaltria australis*	Nearctic	0	-0.5637	13.8897	4.12	0.8109	-	-
Petroicidae	*Eopsaltria flaviventris*	Nearctic	0	-0.6402	8.8304	4.18	0.8383	0.3682	6.79
Petroicidae	*Eopsaltria georgiana*	Nearctic	0	-0.8217	3.9011	4.15	0.8135	0.5591	15.46
Petroicidae	*Eopsaltria griseogularis*	Nearctic	0	-0.7730	14.0057	4.30	0.7883	0.4503	28.19
Petroicidae	*Eopsaltria pulverulenta*	Neotropics	0	-0.3386	3.6304	3.54	0.8301	0.3674	62.02
Petroicidae	*Eugerygone rubra*	Afrotropics	0	0.0658	-	3.49	0.8092	0.5889	51.74
Petroicidae	*Heteromyias albispecularis*	IndoMalay	0	-0.6606	-	3.20	0.7803	0.3796	14.62
Petroicidae	*Melanodryas cucullata*	Neotropics	0	-0.2166	7.6495	3.75	0.8533	0.6888	50.77
Petroicidae	*Melanodryas vittata*	Neotropics	0	-0.0371	12.3860	3.03	0.6842	0.1573	18.96
Petroicidae	*Microeca fascinans*	Palearctic	0	-0.9324	-	5.33	0.8519	0.1552	0.02
Petroicidae	*Microeca flavovirescens*	Palearctic	0	-1.0129	0.0000	4.76	0.8681	0.0985	0.62
Petroicidae	*Microeca griseoceps*	Neotropics	1	-0.4459	5.3485	3.49	0.7895	0.5463	59.58
Petroicidae	*Microeca hemixantha*	Palearctic	0	-0.6729	-	5.01	0.8690	0.0681	0.21
Petroicidae	*Microeca papuana*	Palearctic	1	-0.7895	2.6643	4.33	0.7792	0.4261	14.84
Petroicidae	*Monachella muelleriana*	Palearctic	0	-0.4867	12.9511	3.93	0.8931	0.6058	5.41
Petroicidae	*Pachycephalopsis hattamensis*	IndoMalay	0	-0.4227	-	2.71	0.7128	0.1436	11.08
Petroicidae	*Pachycephalopsis poliosoma*	IndoMalay	0	-0.0041	-	2.83	0.7865	0.5192	8.94
Petroicidae	*Peneothello bimaculata*	Afrotropics	0	0.0702	8.2359	3.50	0.8495	0.4835	21.69
Petroicidae	*Peneothello cryptoleuca*	Palearctic	0	-0.8477	-	4.05	0.8308	0.1189	4.97
Petroicidae	*Peneothello sigillatus*	Afrotropics	0	0.0524	-	3.82	0.8148	0.1254	1.19
Petroicidae	*Petroica archboldi*	Neotropics	0	-0.1325	3.8749	3.89	0.8460	0.4988	39.27
Petroicidae	*Petroica australis*	Neotropics	0	-0.2328	4.1319	2.50	0.7198	0.1174	18.42
Petroicidae	*Petroica bivittata*	Neotropics	0	0.0760	4.2725	3.97	0.8372	0.7962	7.13
Petroicidae	*Petroica goodenovii*	Neotropics	0	-0.1364	11.5081	3.78	0.7920	0.3415	73.18

Family	Species	Realm	Threat	Latitude	Elevation	Anomaly	Size	Shape	Orientation
Petroicidae	*Petroica macrocephala*	Neotropics	1	-0.3772	8.1029	3.88	0.7344	0.4441	28.31
Petroicidae	*Petroica multicolor*	Neotropics	1	-9.2113	5.4983	4.05	0.7151	0.1220	37.95
Petroicidae	*Petroica phoenicea*	IndoMalay	0	-0.3372	-	3.31	0.8202	0.3134	25.25
Petroicidae	*Petroica rodinogaster*	IndoMalay	1	-0.1113	5.5100	3.05	0.7997	0.3649	67.07
Petroicidae	*Petroica rosea*	Australasia	1	-0.6729	-	3.30	0.6974	0.2798	70.32
Petroicidae	*Poecilodryas albonotata*	Afrotropics	0	0.2593	-	3.25	0.7884	0.2973	9.72
Petroicidae	*Poecilodryas brachyura*	Australasia	0	-0.6106	-	3.32	0.8020	0.6881	79.63
Petroicidae	*Poecilodryas cerviniventris*	Nearctic	0	-0.7616	-	5.56	0.8396	0.0494	0.59
Petroicidae	*Poecilodryas hypoleuca*	Afrotropics	0	-0.1200	-	3.55	0.8582	0.5993	44.19
Petroicidae	*Poecilodryas placens*	Neotropics	0	-0.4637	-	3.38	0.8312	0.1864	52.44
Petroicidae	*Poecilodryas superciliosa*	Afrotropics	0	-0.3334	-	3.59	0.8649	0.4664	11.63
Petroicidae	*Tregellasia capito*	Neotropics	0	-0.4795	-	3.21	0.7991	0.5100	70.67
Petroicidae	*Tregellasia leucops*	Neotropics	0	-0.2174	6.3226	3.67	0.8302	0.6358	66.60
Peucedramidae	*Peucedramus taeniatus*	Australasia	0	-0.0728	-	2.78	0.7659	0.2355	23.48
Phalacrocoracidae	*Phalacrocorax africanus*	Neotropics	0	-0.2541	4.6274	3.24	0.7172	0.1914	73.16
Phalacrocoracidae	*Phalacrocorax niger*	Australasia	0	0.0000	1.9062	2.85	0.6120	0.1999	8.30
Phalacrocoracidae	*Phalacrocorax pygmeus*	Australasia	0	-0.4159	-	2.36	0.7151	0.2443	44.06
Phalacrocoracidae	*Phalacrocorax sulcirostris*	Australasia	0	-0.4564	6.9521	2.85	0.6876	0.1126	24.06
Phasianidae	*Afropavo congensis*	IndoMalay	0	-0.0849	-	3.11	0.8272	0.4277	42.92
Phasianidae	*Alectoris barbara*	Australasia	0	-0.0797	12.8974	2.82	0.7409	0.1474	23.70
Phasianidae	*Alectoris chukar*	Palearctic	0	-0.6322	-	3.75	0.7931	0.3697	0.24
Phasianidae	*Alectoris graeca*	Palearctic	0	-0.6068	17.6454	4.16	0.8514	0.1321	0.67
Phasianidae	*Alectoris magna*	Palearctic	0	-0.5331	8.5970	3.82	0.7684	0.3552	17.42
Phasianidae	*Alectoris melanocephala*	Palearctic	0	4.7672	14.1509	4.01	0.7680	0.3747	12.13
Phasianidae	*Alectoris philbyi*	Afrotropics	0	0.0537	13.1314	3.75	0.7584	0.5685	2.02
Phasianidae	*Alectoris rufa*	Afrotropics	0	0.1089	13.2771	3.76	0.7151	0.1659	60.16
Phasianidae	*Ammoperdix griseogularis*	Palearctic	0	-0.8459	-	4.15	0.8142	0.4691	8.72
Phasianidae	*Ammoperdix heyi*	Palearctic	0	-0.3758	8.9465	4.06	0.7990	0.4918	27.00
Phasianidae	*Anurophasis monorthonyx*	Australasia	0	0.0000	7.3813	2.86	0.6598	0.3014	10.21
Phasianidae	*Arborophila ardens*	Neotropics	1	-0.0366	3.5500	4.16	0.7845	0.8446	27.21
Phasianidae	*Arborophila atrogularis*	Neotropics	0	-0.0422	9.8820	3.57	0.7642	0.3403	82.88
Phasianidae	*Arborophila brunneopectus*	Neotropics	0	0.1251	-	4.07	0.8064	0.4887	35.78
Phasianidae	*Arborophila cambodiana*	IndoMalay	1	-0.3501	5.5391	2.53	0.6428	0.4740	0.00
Phasianidae	*Arborophila campbelli*	IndoMalay	0	-0.1360	4.3730	3.25	0.7548	0.7898	47.75
Phasianidae	*Arborophila charltonii*	IndoMalay	0	-0.1547	-	3.07	0.7650	0.7031	25.20
Phasianidae	*Arborophila chloropus*	IndoMalay	0	0.1478	5.3458	3.01	0.6690	0.4183	48.81
Phasianidae	*Arborophila crudigularis*	IndoMalay	0	0.1690	8.4583	2.81	0.6784	0.6474	89.05
Phasianidae	*Arborophila davidi*	IndoMalay	0	-0.0636	0.5856	2.79	0.7331	0.9255	86.64
Phasianidae	*Arborophila gingica*	IndoMalay	0	-0.1206	4.1188	3.02	0.7805	0.7842	56.77
Phasianidae	*Arborophila hyperythra*	IndoMalay	0	-0.9816	7.8853	2.61	0.6478	0.3444	72.46
Phasianidae	*Arborophila javanica*	IndoMalay	0	0.2912	1.4194	3.08	0.6523	0.7160	90.00
Phasianidae	*Arborophila mandellii*	IndoMalay	1	-0.7951	6.0305	3.25	0.7567	0.3360	22.08
Phasianidae	*Arborophila orientalis*	IndoMalay	0	0.1602	6.5383	2.94	0.7185	0.4446	59.55
Phasianidae	*Arborophila rolli*	IndoMalay	0	-0.3980	12.1288	2.73	0.6906	0.1900	11.99
Phasianidae	*Arborophila rubrirostris*	IndoMalay	1	0.1539	10.0531	3.86	0.7112	0.2380	14.05
Phasianidae	*Arborophila rufipectus*	IndoMalay	1	-0.2690	8.0881	2.68	0.6370	0.2803	9.84
Phasianidae	*Arborophila rufogularis*	IndoMalay	0	0.0947	8.5976	2.66	0.6948	0.2307	43.55
Phasianidae	*Arborophila sumatrana*	IndoMalay	0	0.0509	8.8135	2.77	0.7198	0.0963	53.84
Phasianidae	*Arborophila torqueola*	Palearctic	1	38.3645	6.2125	3.30	0.6478	0.6493	64.06
Phasianidae	*Argusianus argus*	IndoMalay	1	-0.1259	0.0000	3.53	0.7884	0.6037	79.72
Phasianidae	*Bambusicola fytchii*	Afrotropics	0	0.0290	-	3.23	0.7797	0.3724	6.97
Phasianidae	*Bambusicola thoracicus*	Afrotropics	0	0.0551	-	3.31	0.8237	0.3925	17.26
Phasianidae	*Bonasa bonasia*	Neotropics	0	0.0372	12.3236	3.47	0.7618	0.3591	48.47
Phasianidae	*Bonasa sewerzowi*	Neotropics	0	0.1291	6.1407	3.97	0.7464	0.1632	37.07
Phasianidae	*Bonasa umbellus*	Nearctic	0	-0.7501	7.1519	4.20	0.8401	0.3129	6.93
Phasianidae	*Caloperdix oculeus*	Nearctic	0	-0.5768	7.3393	4.19	0.7937	0.5772	67.96
Phasianidae	*Catreus wallichi*	Neotropics	0	-0.4057	10.7973	3.79	0.8726	0.3448	52.48
Phasianidae	*Centrocercus minimus*	Neotropics	0	-0.1378	11.8177	3.84	0.7643	0.3471	44.41
Phasianidae	*Centrocercus urophasianus*	Nearctic	0	-0.8025	8.2915	4.16	0.8420	0.2545	9.95
Phasianidae	*Chrysolophus amherstiae*	Australasia	0	-0.5518	0.0000	3.34	0.8379	0.5827	7.73
Phasianidae	*Chrysolophus pictus*	Afrotropics	0	-0.1456	9.7200	3.57	0.8611	0.6964	46.03
Phasianidae	*Coturnix chinensis*	Afrotropics	1	0.0000	0.0000	3.39	0.6120	0.9804	90.00
Phasianidae	*Coturnix coromandelica*	Afrotropics	0	-0.0593	-	3.54	0.8361	0.7982	82.60
Phasianidae	*Coturnix coturnix*	Afrotropics	0	-0.2562	-	3.70	0.7691	0.8258	77.50
Phasianidae	*Coturnix delegorguei*	Afrotropics	0	0.3389	-	3.23	0.6997	0.5118	37.59
Phasianidae	*Coturnix japonica*	Afrotropics	0	-0.1193	-	3.47	0.8229	0.9174	69.62
Phasianidae	*Coturnix pectoralis*	Afrotropics	0	0.0723	-	3.41	0.8306	0.2807	13.48
Phasianidae	*Coturnix ypsilophora*	Afrotropics	0	-0.0415	-	3.38	0.7772	0.3193	16.91
Phasianidae	*Crossoptilon auritum*	Neotropics	1	-0.1594	1.6888	3.05	0.7228	0.3064	63.54
Phasianidae	*Crossoptilon crossoptilon*	Neotropics	0	-0.2514	4.7576	4.02	0.7554	0.4246	3.79
Phasianidae	*Crossoptilon harmani*	Neotropics	0	-0.2209	-	3.88	0.8199	0.6234	58.89
Phasianidae	*Crossoptilon mantchuricum*	Neotropics	1	0.0105	1.1059	4.05	0.7291	0.6842	50.93
Phasianidae	*Dendragapus canadensis*	Neotropics	1	0.0000	9.9488	3.43	0.5985	0.5771	46.13
Phasianidae	*Dendragapus falcipennis*	Neotropics	0	0.0960	-	4.02	0.8311	0.8068	8.06

Family	Species	Realm	Threat	Latitude	Elevation	Anomaly	Size	Shape	Orientation
Phasianidae	*Dendragapus fuliginosus*	Neotropics	0	-0.5468	2.6033	2.77	0.7120	0.2244	33.17
Phasianidae	*Dendragapus obscurus*	Neotropics	0	-0.0175	-	3.00	0.7382	0.6125	53.98
Phasianidae	*Francolinus adspersus*	Neotropics	0	-0.0208	-	3.90	0.7980	0.3694	6.92
Phasianidae	*Francolinus afer*	Neotropics	0	0.0040	-	3.91	0.8342	0.5462	29.10
Phasianidae	*Francolinus africanus*	Neotropics	0	-0.1905	-	3.95	0.8370	0.6473	20.25
Phasianidae	*Francolinus ahantensis*	Neotropics	0	-0.0140	-	2.99	0.7286	0.4961	69.40
Phasianidae	*Francolinus albogularis*	Neotropics	0	-5.5305	0.3280	4.20	0.6804	0.6224	2.87
Phasianidae	*Francolinus bicalcaratus*	Neotropics	0	0.0338	11.3130	3.44	0.7550	0.3129	76.88
Phasianidae	*Francolinus capensis*	Neotropics	0	0.0000	1.5395	3.65	0.6120	0.3768	59.29
Phasianidae	*Francolinus castaneicollis*	Neotropics	0	-0.1108	-	3.92	0.8334	0.4363	35.92
Phasianidae	*Francolinus clappertoni*	Neotropics	0	-0.0404	4.0704	3.49	0.6891	0.3712	68.35
Phasianidae	*Francolinus coqui*	Neotropics	0	-0.2229	-	3.77	0.8105	0.3730	30.74
Phasianidae	*Francolinus erckelii*	Neotropics	0	-0.1305	-	3.86	0.8226	0.5961	4.24
Phasianidae	*Francolinus finschi*	Neotropics	0	-0.5017	-	3.29	0.7318	0.8819	72.29
Phasianidae	*Francolinus francolinus*	Neotropics	0	-0.1709	-	3.22	0.7234	0.2801	84.95
Phasianidae	*Francolinus griseostriatus*	Neotropics	0	-0.0171	5.2517	3.53	0.7569	0.8615	3.85
Phasianidae	*Francolinus gularis*	Neotropics	0	-0.1969	4.4678	3.79	0.7223	0.1379	59.73
Phasianidae	*Francolinus hartlaubi*	Neotropics	0	-0.0021	3.0149	3.94	0.8139	0.6985	8.08
Phasianidae	*Francolinus harwoodi*	Neotropics	0	-0.0018	-	3.87	0.7859	0.2780	29.71
Phasianidae	*Francolinus hildebrandti*	Neotropics	1	0.0000	6.8286	3.58	0.6221	0.5728	71.79
Phasianidae	*Francolinus icterorhynchus*	Neotropics	0	-0.1514	3.9726	3.87	0.8425	0.5872	13.82
Phasianidae	*Francolinus jacksoni*	Afrotropics	0	-0.0726	-	3.18	0.7614	0.3874	70.15
Phasianidae	*Francolinus lathami*	Afrotropics	0	-0.1089	8.0094	3.16	0.7474	0.3170	71.22
Phasianidae	*Francolinus levaillantii*	Afrotropics	0	-0.1333	-	4.23	0.7874	0.6248	22.26
Phasianidae	*Francolinus levaillantoides*	Afrotropics	0	-0.2358	-	3.52	0.8263	0.8183	28.62
Phasianidae	*Francolinus nahani*	Afrotropics	0	-0.4806	5.5689	3.37	0.7606	0.3642	24.64
Phasianidae	*Francolinus natalensis*	Afrotropics	0	0.0000	-	3.15	0.7716	0.2569	6.76
Phasianidae	*Francolinus nobilis*	Afrotropics	0	-0.1396	-	3.49	0.7897	0.2751	28.11
Phasianidae	*Francolinus ochropectus*	Afrotropics	0	-0.2738	-	3.52	0.8188	0.4461	11.32
Phasianidae	*Francolinus pintadeanus*	Afrotropics	0	0.0099	-	3.15	0.7378	0.4146	44.28
Phasianidae	*Francolinus pondicerianus*	Afrotropics	0	0.1829	15.5674	3.52	0.7552	0.6272	33.95
Phasianidae	*Francolinus psilolaemus*	Afrotropics	0	0.0376	-	3.58	0.8099	0.3124	8.06
Phasianidae	*Francolinus rufopictus*	Afrotropics	0	-0.0670	-	3.67	0.8263	0.7293	51.22
Phasianidae	*Francolinus schlegelii*	Afrotropics	0	0.0487	9.1510	3.59	0.7382	0.4031	85.46
Phasianidae	*Francolinus sephaena*	Afrotropics	0	0.4077	-	3.52	0.7505	0.5746	83.73
Phasianidae	*Francolinus shelleyi*	IndoMalay	0	-0.5371	-	3.85	0.8168	0.1845	5.81
Phasianidae	*Francolinus squamatus*	Afrotropics	0	-0.2783	4.5787	3.27	0.6906	0.4737	88.11
Phasianidae	*Francolinus streptophorus*	IndoMalay	1	-0.0652	0.4989	3.30	0.7507	0.4245	9.24
Phasianidae	*Francolinus swainsonii*	Afrotropics	0	-0.2695	-	3.91	0.7544	0.3722	74.64
Phasianidae	*Francolinus swierstrai*	Afrotropics	1	-21.2215	7.5668	3.58	0.6997	0.3662	28.96
Phasianidae	*Galloperdix bicalcarata*	Australasia	0	-0.0837	6.2794	2.71	0.7179	0.5352	59.02
Phasianidae	*Galloperdix lunulata*	IndoMalay	0	-0.4749	-	3.35	0.8459	0.5295	17.29
Phasianidae	*Galloperdix spadicea*	Neotropics	0	0.3052	8.0221	4.05	0.7777	0.2348	52.20
Phasianidae	*Gallus gallus*	Nearctic	0	-0.9076	10.5675	4.32	0.8512	0.2652	10.80
Phasianidae	*Gallus lafayetii*	Palearctic	0	-0.9553	4.0588	3.49	0.7363	0.4084	58.93
Phasianidae	*Gallus sonneratii*	Neotropics	0	0.0826	5.7901	3.71	0.7416	0.2128	65.73
Phasianidae	*Gallus varius*	Neotropics	0	0.0253	12.4693	3.71	0.7599	0.4030	81.73
Phasianidae	*Haematortyx sanguiniceps*	Australasia	0	-0.0556	9.2539	2.75	0.7626	0.2269	18.59
Phasianidae	*Ithaginis cruentus*	Neotropics	0	-0.2795	8.4651	3.53	0.6631	0.5876	89.98
Phasianidae	*Lagopus lagopus*	Neotropics	0	-0.2532	5.5387	3.04	0.6221	0.3473	45.31
Phasianidae	*Lagopus leucura*	Afrotropics	0	-0.0949	12.0694	3.42	0.8028	0.5791	80.70
Phasianidae	*Lagopus muta*	Palearctic	0	-0.5725	-	4.58	0.8623	0.1826	0.09
Phasianidae	*Lerwa lerwa*	Australasia	1	-0.1694	-	2.27	0.6523	0.4886	24.05
Phasianidae	*Lophophorus impejanus*	Afrotropics	0	-0.4689	5.9586	3.24	0.7323	0.3226	57.72
Phasianidae	*Lophophorus lhuysii*	Neotropics	0	0.3497	-	4.07	0.8064	0.5354	6.16
Phasianidae	*Lophophorus sclateri*	Neotropics	0	-9.5578	5.9212	3.36	0.7087	0.1320	61.02
Phasianidae	*Lophura bulweri*	Australasia	0	-0.1227	-	2.52	0.5788	0.0000	90.00
Phasianidae	*Lophura diardi*	Afrotropics	0	-0.0891	-	3.38	0.8089	0.3735	30.24
Phasianidae	*Lophura edwardsi*	IndoMalay	0	0.0918	-	2.84	0.7687	0.6575	46.07
Phasianidae	*Lophura erythrophthalma*	Australasia	0	-0.0862	-	2.76	0.7416	0.1890	32.84
Phasianidae	*Lophura hatinhensis*	Afrotropics	0	-0.1417	-	3.26	0.7672	0.4670	87.43
Phasianidae	*Lophura hoogerwerfi*	IndoMalay	0	0.0441	-	3.00	0.7256	0.4105	63.49
Phasianidae	*Lophura ignita*	IndoMalay	0	-0.0039	-	2.80	0.7936	0.5364	20.87
Phasianidae	*Lophura inornata*	IndoMalay	0	0.0201	-	2.77	0.7281	0.2443	23.85
Phasianidae	*Lophura leucomelanos*	Australasia	0	-0.0266	-	2.77	0.7096	0.3830	35.57
Phasianidae	*Lophura nycthemera*	IndoMalay	0	-0.0356	-	2.81	0.7718	0.4682	58.22
Phasianidae	*Lophura swinhoii*	IndoMalay	0	-0.0980	3.7778	3.57	0.8176	0.6084	12.24
Phasianidae	*Margaroperdix madagascariensis*	Afrotropics	0	-0.1746	5.5366	3.13	0.7261	0.3543	75.99
Phasianidae	*Melanoperdix niger*	Neotropics	0	0.0241	6.6281	3.68	0.7524	0.3318	67.07
Phasianidae	*Meleagris gallopavo*	Neotropics	0	-0.1981	2.8909	2.41	0.5788	0.0000	0.00
Phasianidae	*Meleagris ocellata*	Neotropics	0	6.9663	1.8516	3.41	0.6598	0.2321	74.66
Phasianidae	*Ophrysia superciliosa*	Neotropics	0	-0.6827	4.3526	2.42	0.7611	0.4579	79.74
Phasianidae	*Pavo cristatus*	Palearctic	0	-0.6248	12.1846	3.87	0.7865	0.4285	7.41

Family	Species	Realm	Threat	Latitude	Elevation	Anomaly	Size	Shape	Orientation
Phasianidae	*Pavo muticus*	IndoMalay	0	-0.0014	6.0138	2.64	0.7323	0.2320	67.55
Phasianidae	*Perdicula argoondah*	Afrotropics	0	-0.2497	0.0000	4.16	0.7973	0.6134	16.91
Phasianidae	*Perdicula asiatica*	Palearctic	0	0.3553	-	3.97	0.7314	0.2864	3.25
Phasianidae	*Perdicula erythrorhyncha*	Afrotropics	0	-0.3070	11.2219	3.19	0.7120	0.4435	72.48
Phasianidae	*Perdicula manipurensis*	Palearctic	0	-0.8139	-	3.57	0.8269	0.4009	8.06
Phasianidae	*Perdix dauurica*	Afrotropics	0	-0.2435	0.0000	3.42	0.7522	0.1656	11.85
Phasianidae	*Perdix hodgsoniae*	Palearctic	0	-0.3455	0.0000	4.08	0.7880	0.4826	5.01
Phasianidae	*Perdix perdix*	Afrotropics	0	1.3440	-	3.27	0.7526	0.5164	86.48
Phasianidae	*Phasianus colchicus*	Nearctic	0	-0.2369	4.3485	3.95	0.7291	0.2698	58.92
Phasianidae	*Polyplectron bicalcaratum*	Afrotropics	0	0.0257	-	3.19	0.7914	0.2024	11.81
Phasianidae	*Polyplectron chalcurum*	Afrotropics	0	-0.3105	-	3.89	0.8196	0.8636	35.13
Phasianidae	*Polyplectron germaini*	Afrotropics	0	0.0626	7.0932	3.50	0.8275	0.2347	13.28
Phasianidae	*Polyplectron inopinatum*	Afrotropics	0	-1.3521	-	3.32	0.7040	0.5386	13.37
Phasianidae	*Polyplectron katsumatae*	Afrotropics	0	-0.1232	6.5487	3.64	0.8156	0.8591	64.68
Phasianidae	*Polyplectron malacense*	Afrotropics	0	-0.1736	-	3.75	0.7629	0.7617	18.65
Phasianidae	*Polyplectron napoleonis*	Palearctic	0	-0.9797	-	5.50	0.8190	0.1131	5.59
Phasianidae	*Polyplectron schleiermacheri*	Nearctic	0	-1.0747	2.9459	5.19	0.8209	0.1355	1.86
Phasianidae	*Ptilopachus petrosus*	Neotropics	0	-0.1224	4.9995	3.40	0.7723	0.2759	38.53
Phasianidae	*Pucrasia macrolopha*	Australasia	0	-0.1023	-	2.79	0.7554	0.2115	24.80
Phasianidae	*Rheinardia ocellata*	IndoMalay	0	-0.6383	-	3.32	0.8035	0.5064	17.96
Phasianidae	*Rhizothera longirostris*	Neotropics	0	0.3636	-	4.18	0.7643	0.5941	18.37
Phasianidae	*Rollulus rouloul*	Neotropics	0	0.2429	5.4815	3.02	0.6562	0.1674	28.61
Phasianidae	*Syrmaticus ellioti*	Palearctic	0	-0.4416	0.2317	3.90	0.8442	0.4308	5.71
Phasianidae	*Syrmaticus humiae*	Nearctic	0	-0.7266	0.2018	5.30	0.8492	0.0628	0.77
Phasianidae	*Syrmaticus mikado*	Neotropics	0	-0.2365	2.4027	3.84	0.8494	0.6504	53.18
Phasianidae	*Syrmaticus reevesii*	Neotropics	0	-0.5513	-	2.37	0.7674	0.2693	28.60
Phasianidae	*Tetrao mlokosiewiczi*	IndoMalay	0	-0.8643	8.3763	2.61	0.6478	0.3444	72.46
Phasianidae	*Tetrao parvirostris*	Palearctic	1	-0.6124	9.1498	3.44	0.7664	0.3602	17.46
Phasianidae	*Tetrao tetrix*	Palearctic	0	-0.7812	5.1021	3.14	0.7323	0.2098	29.68
Phasianidae	*Tetrao urogallus*	Palearctic	0	-0.9938	7.9535	4.48	0.8391	0.1634	1.60
Phasianidae	*Tetraogallus altaicus*	Palearctic	0	10.7724	14.3582	4.63	0.7912	0.3716	13.27
Phasianidae	*Tetraogallus caspius*	Neotropics	0	-0.1021	-	3.88	0.6859	0.7005	89.48
Phasianidae	*Tetraogallus caucasicus*	Neotropics	0	-0.2357	-	2.50	0.7205	0.1761	18.86
Phasianidae	*Tetraogallus himalayensis*	Neotropics	0	-0.0956	3.2845	4.01	0.8448	0.6332	23.30
Phasianidae	*Tetraogallus tibetanus*	Neotropics	0	-0.5844	8.4990	2.86	0.8070	0.5202	77.07
Phasianidae	*Tetraophasis obscurus*	Neotropics	0	-0.2297	7.2145	3.60	0.8450	0.4049	41.61
Phasianidae	*Tetraophasis szechenyii*	Australasia	0	-0.4199	3.0469	2.93	0.8128	0.8573	48.84
Phasianidae	*Tragopan blythii*	Neotropics	0	0.1846	6.2248	3.35	0.7449	0.2926	78.79
Phasianidae	*Tragopan caboti*	Neotropics	0	0.0009	9.5938	3.26	0.6784	0.5948	79.30
Phasianidae	*Tragopan melanocephalus*	Afrotropics	0	-0.4098	-	3.32	0.6986	0.4577	89.99
Phasianidae	*Tragopan satyra*	Neotropics	0	-0.2202	8.7863	3.71	0.7327	0.1377	53.94
Phasianidae	*Tragopan temminckii*	IndoMalay	1	0.4583	-	3.02	0.6662	0.5944	7.58
Phasianidae	*Tympanuchus cupido*	Australasia	0	-0.0969	8.5256	2.71	0.7144	0.4924	56.18
Phasianidae	*Tympanuchus pallidicinctus*	Australasia	0	-0.3684	2.3069	3.10	0.8168	0.6952	58.41
Phasianidae	*Tympanuchus phasianellus*	IndoMalay	0	-0.1312	8.5069	2.64	0.6690	0.5573	68.41
Phasianidae	*Xenoperdix udzungwensis*	Neotropics	0	0.1006	-	3.96	0.8350	0.7569	4.34
Philepittidae	*Neodrepanis coruscans*	Australasia	0	0.3841	-	2.60	0.6120	0.4082	18.43
Philepittidae	*Neodrepanis hypoxantha*	IndoMalay	0	-0.0181	-	2.79	0.7755	0.7473	44.03
Philepittidae	*Philepitta castanea*	IndoMalay	0	-0.0831	-	3.16	0.8240	0.7344	36.21
Philepittidae	*Philepitta schlegeli*	IndoMalay	0	-0.0131	-	3.18	0.7737	0.7471	83.90
Phoenicopteridae	*Phoeniconaias minor*	Neotropics	0	-0.1953	-	3.94	0.8452	0.5607	21.71
Phoenicopteridae	*Phoenicoparrus andinus*	Neotropics	0	0.1528	1.0652	4.30	0.7702	0.3852	42.74
Phoenicopteridae	*Phoenicoparrus jamesi*	Neotropics	0	-0.5708	9.4955	3.00	0.7416	0.2717	39.91
Phoenicopteridae	*Phoenicopterus chilensis*	Neotropics	0	0.0858	4.1386	3.40	0.7797	0.4192	33.13
Phoenicopteridae	*Phoenicopterus roseus*	Neotropics	0	-1.1301	3.0873	4.07	0.7367	0.1336	31.34
Phoenicopteridae	*Phoenicopterus ruber*	Neotropics	0	-0.0237	3.4517	3.98	0.7464	0.3288	13.00
Phoeniculidae	*Phoeniculus bollei*	Neotropics	0	0.0175	3.3571	4.02	0.8090	0.4579	35.71
Phoeniculidae	*Phoeniculus castaneiceps*	Neotropics	0	-0.0937	7.5947	3.25	0.7464	0.2533	71.77
Phoeniculidae	*Phoeniculus damarensis*	Neotropics	0	0.2155	-	3.01	0.7223	0.1488	71.52
Phoeniculidae	*Phoeniculus purpureus*	Neotropics	0	-0.2229	-	3.77	0.8534	0.7332	54.03
Phoeniculidae	*Phoeniculus somaliensis*	Nearctic	0	-0.6279	10.0696	3.94	0.7912	0.2614	38.25
Phoeniculidae	*Rhinopomastus aterrimus*	Neotropics	0	0.0609	5.2641	3.79	0.8000	0.5837	8.66
Phoeniculidae	*Rhinopomastus cyanomelas*	Neotropics	0	-0.3049	-	3.21	0.7874	0.3773	30.59
Phoeniculidae	*Rhinopomastus minor*	Neotropics	0	-0.0674	-	4.27	0.7524	0.5240	37.51
Picathartidae	*Picathartes gymnocephalus*	Neotropics	0	0.2096	-	3.82	0.7777	0.4984	43.38
Picathartidae	*Picathartes oreas*	Neotropics	0	-0.5185	6.2289	2.93	0.7314	0.2242	36.32
Picidae	*Blythipicus pyrrhotis*	Australasia	0	-0.5943	0.0000	2.93	0.7860	0.2853	0.34
Picidae	*Blythipicus rubiginosus*	Afrotropics	0	0.0957	3.4630	3.24	0.7841	0.2342	10.32
Picidae	*Campephilus guatemalensis*	Afrotropics	0	-0.0691	8.8664	3.52	0.8583	0.6295	32.63
Picidae	*Campephilus haematogaster*	Afrotropics	0	-0.0237	-	3.27	0.8154	0.1994	14.97
Picidae	*Campephilus imperialis*	Afrotropics	0	-0.0848	-	4.24	0.7928	0.4928	21.63
Picidae	*Campephilus leucopogon*	Afrotropics	0	0.0014	6.0275	3.34	0.7964	0.5741	48.53
Picidae	*Campephilus magellanicus*	Afrotropics	0	0.0347	-	3.26	0.8162	0.2954	13.66
Picidae	*Campephilus melanoleucos*	Afrotropics	0	-0.2043	8.2707	3.67	0.8171	0.8828	3.37

Family	Species	Realm	Threat	Latitude	Elevation	Anomaly	Size	Shape	Orientation
Picidae	*Campephilus pollens*	Afrotropics	0	-0.1724	-	3.59	0.8299	0.6627	68.60
Picidae	*Campephilus principalis*	Afrotropics	1	0.3935	2.3099	3.07	0.7488	0.3051	4.22
Picidae	*Campephilus robustus*	Afrotropics	0	0.3741	5.9984	3.26	0.7618	0.2565	2.92
Picidae	*Campephilus rubricollis*	Afrotropics	0	0.0538	-	3.25	0.7648	-	-
Picidae	*Campephilus rubricollis*	Afrotropics	0	0.0538	-	3.25	0.7648	0.6132	4.59
Picidae	*Campethera abingoni*	Afrotropics	0	-0.0233	-	3.43	0.8275	0.1714	4.04
Picidae	*Campethera bennettii*	Afrotropics	0	0.0165	5.8500	3.34	0.7947	0.3666	18.27
Picidae	*Campethera cailliautii*	Neotropics	0	-0.1251	2.1915	3.10	0.7192	0.2283	80.66
Picidae	*Campethera caroli*	Neotropics	0	-0.0082	-	3.48	0.7826	0.2530	27.60
Picidae	*Campethera maculosa*	Neotropics	0	0.0344	-	3.45	0.7569	0.2620	79.22
Picidae	*Campethera mombassica*	Nearctic	1	-0.3391	7.0670	3.98	0.7642	0.3359	46.02
Picidae	*Campethera nivosa*	Neotropics	0	-0.3463	-	3.38	0.7863	0.5532	86.58
Picidae	*Campethera notata*	Neotropics	0	-0.4924	6.3290	1.99	0.7548	0.2611	86.53
Picidae	*Campethera nubica*	Neotropics	0	-0.1325	-	3.94	0.8488	0.6030	31.98
Picidae	*Campethera punctuligera*	Neotropics	0	-0.1951	14.3358	3.41	0.7490	0.3107	74.49
Picidae	*Celeus brachyurus*	Neotropics	0	-0.2069	-	3.68	0.8417	0.4696	46.29
Picidae	*Celeus castaneus*	Neotropics	0	-0.0669	-	4.03	0.8353	0.6983	2.29
Picidae	*Celeus elegans*	Neotropics	0	-0.0683	11.8077	3.68	0.7821	0.1819	25.84
Picidae	*Celeus flavescens*	Nearctic	1	-0.9498	7.2316	4.24	0.7205	0.3552	29.03
Picidae	*Celeus flavus*	Neotropics	0	-0.0839	11.3864	3.72	0.7769	0.2765	56.66
Picidae	*Celeus grammicus*	Neotropics	0	-0.0502	12.1932	3.60	0.7574	0.1618	24.23
Picidae	*Celeus loricatus*	Neotropics	0	0.0302	12.6757	3.68	0.7634	0.4000	72.99
Picidae	*Celeus lugubris*	Nearctic	0	-0.5608	-	4.23	0.8233	0.2173	4.65
Picidae	*Celeus obrieni*	Neotropics	0	0.1234	9.8714	2.99	0.6523	0.2357	33.36
Picidae	*Celeus spectabilis*	Nearctic	0	-0.8515	-	4.17	0.8436	0.3151	8.00
Picidae	*Celeus torquatus*	Neotropics	0	-0.0582	7.0878	3.56	0.7396	0.1667	33.33
Picidae	*Celeus undatus*	Nearctic	0	-0.5439	8.1559	4.72	0.8298	0.0513	0.37
Picidae	*Chrysocolaptes festivus*	Neotropics	0	-0.2283	2.9358	3.79	0.8513	0.6609	51.77
Picidae	*Chrysocolaptes lucidus*	Neotropics	0	-0.0967	3.6407	3.96	0.8216	0.5155	39.04
Picidae	*Colaptes atricollis*	Neotropics	0	-0.1359	2.4287	3.88	0.8507	0.4071	38.28
Picidae	*Colaptes auratus*	IndoMalay	1	-0.2329	11.5500	2.75	0.6598	0.2841	13.18
Picidae	*Colaptes auricularis*	IndoMalay	1	0.1135	6.8341	2.77	0.7205	0.1065	55.01
Picidae	*Colaptes campestris*	IndoMalay	0	-0.2213	10.4586	3.35	0.7738	0.3780	15.44
Picidae	*Colaptes chrysoides*	IndoMalay	0	-0.1298	5.8424	3.18	0.7948	0.5370	30.02
Picidae	*Colaptes fernandinae*	Neotropics	0	-0.3379	10.0637	3.85	0.7030	0.4062	64.48
Picidae	*Colaptes melanochloros*	Neotropics	0	0.0720	6.4196	3.69	0.7713	0.3987	84.85
Picidae	*Colaptes pitius*	Neotropics	0	-0.2430	8.2238	3.78	0.6961	0.2981	83.81
Picidae	*Colaptes punctigula*	Neotropics	0	0.2419	9.0102	3.45	0.7104	0.3600	69.81
Picidae	*Colaptes rivolii*	Neotropics	0	0.1164	5.9982	3.19	0.7165	0.1441	66.19
Picidae	*Colaptes rubiginosus*	Neotropics	1	0.0000	2.2140	3.18	0.5788	0.0000	0.00
Picidae	*Colaptes rupicola*	Neotropics	0	0.1527	12.4247	3.32	0.6120	0.9804	90.00
Picidae	*Dendrocopos assimilis*	Nearctic	0	-0.6421	-	4.31	0.8407	0.1622	9.53
Picidae	*Dendrocopos atratus*	Palearctic	0	-0.7873	-	4.48	0.7808	0.8627	86.51
Picidae	*Dendrocopos auriceps*	Nearctic	0	-0.5644	12.7266	3.03	0.7590	0.2841	50.11
Picidae	*Dendrocopos canicapillus*	Nearctic	0	-0.7656	16.8925	3.81	0.8032	0.3197	45.44
Picidae	*Dendrocopos cathpharius*	Neotropics	0	0.0000	-	4.06	0.8170	0.6480	18.13
Picidae	*Dendrocopos darjellensis*	Neotropics	0	-0.0284	-	3.38	0.7565	0.4354	32.74
Picidae	*Dendrocopos dorae*	Neotropics	0	-0.1671	5.4308	3.88	0.8458	0.5534	42.14
Picidae	*Dendrocopos himalayensis*	Neotropics	0	-0.1295	3.8037	3.36	0.7678	0.2961	27.85
Picidae	*Dendrocopos hyperythrus*	Neotropics	0	0.0184	-	4.04	0.8299	0.7127	10.68
Picidae	*Dendrocopos kizuki*	Neotropics	0	0.0604	10.3879	3.44	0.7533	0.3219	84.21
Picidae	*Dendrocopos leucopterus*	IndoMalay	0	-0.6117	-	3.31	0.8182	0.3821	4.78
Picidae	*Dendrocopos leucotos*	IndoMalay	0	0.1663	-	3.50	0.7422	0.3243	22.18
Picidae	*Dendrocopos macei*	IndoMalay	0	0.0110	6.4104	3.09	0.7165	0.1511	64.82
Picidae	*Dendrocopos maculatus*	IndoMalay	0	0.0848	-	2.84	0.7546	0.3475	7.48
Picidae	*Dendrocopos mahrattensis*	IndoMalay	0	-0.1078	-	3.37	0.8248	0.4374	13.44
Picidae	*Dendrocopos major*	Neotropics	0	-0.0401	3.7975	4.04	0.8355	0.7422	0.13
Picidae	*Dendrocopos medius*	Neotropics	0	0.0161	-	4.27	0.7791	0.6373	67.85
Picidae	*Dendrocopos minor*	Neotropics	0	-0.1305	-	3.95	0.8332	0.7753	55.34
Picidae	*Dendrocopos moluccensis*	Neotropics	0	-0.3168	-	3.56	0.8195	0.4456	58.23
Picidae	*Dendrocopos nanus*	Neotropics	0	-0.0444	3.8018	3.35	0.7806	0.3123	33.55
Picidae	*Dendrocopos obsoletus*	Palearctic	0	-0.1672	-	4.10	0.7718	0.3522	11.67
Picidae	*Dendrocopos syriacus*	IndoMalay	0	-0.0753	7.6154	3.11	0.7631	0.3670	27.86
Picidae	*Dendrocopos temminckii*	IndoMalay	0	0.0156	-	4.08	0.7305	0.1660	21.96
Picidae	*Dendropicos abyssinicus*	Nearctic	0	-0.9422	6.6695	4.23	0.8477	0.3279	9.27
Picidae	*Dendropicos elachus*	Nearctic	0	-0.8683	3.3404	3.99	0.7997	0.5178	21.33
Picidae	*Dendropicos fuscescens*	Nearctic	0	-0.8085	-	4.04	0.8016	0.5973	12.75
Picidae	*Dendropicos gabonensis*	Nearctic	0	-0.8341	-	4.40	0.8067	0.3378	12.92
Picidae	*Dendropicos poecilolaemus*	Nearctic	0	-0.2711	9.4941	4.08	0.7911	0.2948	47.20
Picidae	*Dendropicos stierlingi*	Nearctic	0	23.2054	-	4.31	0.6662	0.6186	0.00
Picidae	*Dinopium benghalense*	IndoMalay	0	0.0716	1.1815	2.85	0.7522	0.2221	48.81
Picidae	*Dinopium javanense*	Neotropics	0	-0.0090	9.5836	3.52	0.7570	0.2620	67.32
Picidae	*Dinopium rafflesii*	Neotropics	0	0.2008	12.0156	3.66	0.7517	0.1567	20.42
Picidae	*Dinopium shorii*	Neotropics	0	-0.1352	8.8729	3.93	0.7574	0.4024	66.40

Family	Species	Realm	Threat	Latitude	Elevation	Anomaly	Size	Shape	Orientation
Picidae	*Dryocopus galeatus*	Neotropics	0	-0.1322	10.7705	3.62	0.7104	0.2741	26.53
Picidae	*Dryocopus javensis*	Neotropics	0	0.2170	5.9066	3.97	0.7654	0.3496	36.62
Picidae	*Dryocopus lineatus*	Neotropics	0	0.0257	8.1052	3.59	0.7707	0.3696	75.54
Picidae	*Dryocopus pileatus*	Australasia	0	-0.2195	-	2.58	0.6302	0.1134	34.50
Picidae	*Dryocopus schulzi*	Afrotropics	0	-0.1094	10.2319	3.04	0.7192	0.1438	74.47
Picidae	*Gecinulus grantia*	IndoMalay	0	-0.1422	10.4201	3.15	0.7648	0.9003	26.72
Picidae	*Gecinulus viridis*	IndoMalay	0	-0.3208	6.3408	3.25	0.7666	0.6238	12.81
Picidae	*Geocolaptes olivaceus*	IndoMalay	0	-0.2275	-	3.19	0.8111	0.6127	0.09
Picidae	*Hemicircus canente*	Australasia	0	-0.2201	4.9036	2.56	0.6523	0.1875	30.24
Picidae	*Hemicircus concretus*	Neotropics	0	-0.2153	4.7242	3.92	0.8091	0.6122	17.36
Picidae	*Jynx ruficollis*	Australasia	0	-0.1708	-	3.09	0.8056	0.4626	36.72
Picidae	*Jynx torquilla*	IndoMalay	0	-0.0217	3.7762	2.64	0.7261	0.2111	67.49
Picidae	*Meiglyptes jugularis*	IndoMalay	0	0.0055	5.8519	2.83	0.7824	0.4624	12.50
Picidae	*Meiglyptes tristis*	IndoMalay	0	0.0848	6.5628	2.76	0.6478	0.4543	18.43
Picidae	*Meiglyptes tukki*	IndoMalay	0	0.1280	5.1694	2.88	0.7438	0.4234	46.48
Picidae	*Melanerpes aurifrons*	IndoMalay	0	-0.2056	5.0503	3.00	0.7740	0.6026	65.33
Picidae	*Melanerpes cactorum*	IndoMalay	0	-0.3571	3.8982	2.71	0.7060	0.1368	11.62
Picidae	*Melanerpes candidus*	IndoMalay	0	-0.1638	-	2.92	0.7498	0.3063	73.14
Picidae	*Melanerpes carolinus*	IndoMalay	0	-0.1120	-	3.16	0.8053	0.3807	46.48
Picidae	*Melanerpes chrysauchen*	IndoMalay	0	0.1635	3.4182	2.89	0.7245	0.4383	57.23
Picidae	*Melanerpes chrysogenys*	IndoMalay	0	0.0668	2.0357	2.84	0.7887	0.5564	14.90
Picidae	*Melanerpes cruentatus*	IndoMalay	0	-0.8490	10.3318	2.61	0.6478	0.3444	72.46
Picidae	*Melanerpes erythrocephalus*	IndoMalay	0	0.0149	7.3037	2.78	0.7331	0.4147	62.05
Picidae	*Melanerpes flavifrons*	IndoMalay	0	-0.0549	11.6674	2.79	0.6631	0.3462	47.38
Picidae	*Melanerpes formicivorus*	IndoMalay	0	0.0084	1.1605	2.85	0.7813	0.4723	10.98
Picidae	*Melanerpes hoffmannii*	IndoMalay	0	0.0715	3.7887	2.99	0.7261	0.2036	50.71
Picidae	*Melanerpes hypopolius*	IndoMalay	0	-0.3405	8.4715	3.36	0.8167	0.4000	3.16
Picidae	*Melanerpes lewis*	IndoMalay	0	-0.0978	6.6650	3.18	0.7422	0.2042	64.87
Picidae	*Melanerpes portoricensis*	IndoMalay	0	-0.0999	-	3.37	0.8045	0.6326	80.24
Picidae	*Melanerpes pucherani*	Australasia	1	-0.1522	0.0000	2.50	0.6598	0.3252	28.84
Picidae	*Melanerpes pulcher*	Australasia	0	-0.6907	0.0000	2.50	0.5788	0.0000	45.57
Picidae	*Melanerpes pygmaeus*	Australasia	0	0.0626	3.4599	2.58	0.6302	0.1134	34.50
Picidae	*Melanerpes radiolatus*	Australasia	0	-0.1839	4.5944	2.49	0.6662	0.3153	20.62
Picidae	*Melanerpes rubricapillus*	Australasia	0	-0.0261	5.9615	2.49	0.6562	0.1342	28.92
Picidae	*Melanerpes striatus*	Australasia	1	-0.0774	-	2.79	0.6221	0.4726	87.23
Picidae	*Melanerpes uropygialis*	Australasia	0	-0.6265	-	3.24	0.8154	0.5850	1.89
Picidae	*Mesopicos elliotii*	Australasia	0	-0.2296	-	2.86	0.7251	0.1361	24.90
Picidae	*Mesopicos goertae*	Australasia	0	-0.0755	-	2.73	0.7413	0.1774	23.63
Picidae	*Mesopicos griseocephalus*	Australasia	0	-0.1983	-	2.91	0.7128	0.1874	57.82
Picidae	*Mulleripicus fulvus*	Nearctic	0	-0.6654	-	4.29	0.8323	0.4049	19.12
Picidae	*Mulleripicus funebris*	Afrotropics	1	0.0541	4.1957	3.29	0.6478	0.2592	88.69
Picidae	*Mulleripicus pulverulentus*	Afrotropics	0	-0.0246	8.8008	3.28	0.7158	0.2467	51.52
Picidae	*Nesoctites micromegas*	Afrotropics	0	-0.1228	-	3.69	0.7657	0.4964	31.52
Picidae	*Picoides albolarvatus*	Neotropics	0	0.0151	10.5242	3.56	0.7554	0.3098	80.41
Picidae	*Picoides arcticus*	Neotropics	0	0.0446	5.7270	3.58	0.7272	0.3212	84.32
Picidae	*Picoides arcticus*	Neotropics	0	0.0446	5.7270	3.58	0.7272	-	-
Picidae	*Picoides arizonae*	Neotropics	0	-0.2181	1.8186	4.00	0.7716	0.2111	48.00
Picidae	*Picoides borealis*	Neotropics	0	2.4009	9.8778	3.93	0.7256	0.3064	59.54
Picidae	*Picoides dorsalis*	Neotropics	1	0.1095	1.2300	3.72	0.6120	0.6246	0.00
Picidae	*Picoides fumigatus*	Neotropics	0	-0.1238	3.1254	3.77	0.7567	0.3555	64.89
Picidae	*Picoides nuttallii*	Neotropics	0	-0.4822	6.7370	3.05	0.7831	0.4731	30.41
Picidae	*Picoides pubescens*	Neotropics	1	-6.9893	3.0909	4.05	0.6662	0.3401	44.56
Picidae	*Picoides scalaris*	Neotropics	1	0.0382	2.3837	3.43	0.6562	0.2771	68.11
Picidae	*Picoides stricklandi*	Neotropics	1	0.0673	0.9184	3.18	0.5788	0.0000	0.00
Picidae	*Picoides tridactylus*	Neotropics	0	0.5749	5.5642	4.38	0.7305	0.4861	3.33
Picidae	*Picoides villosus*	Neotropics	0	-0.5146	6.8640	2.65	0.7296	0.2026	42.58
Picidae	*Piculus aurulentus*	Neotropics	0	-0.5043	-	3.20	0.7814	0.4046	29.99
Picidae	*Piculus callopterus*	Neotropics	0	-0.2697	4.5589	3.80	0.6370	0.3555	3.48
Picidae	*Piculus chrysochloros*	Neotropics	0	-0.0075	5.4831	2.90	0.6598	0.3745	36.50
Picidae	*Piculus flavigula*	Neotropics	0	-4.7893	6.8349	3.34	0.6763	0.1325	69.98
Picidae	*Piculus leucolaemus*	Neotropics	1	-0.5882	-	2.36	0.6631	0.1482	68.22
Picidae	*Piculus litae*	Neotropics	1	9.9352	2.5392	3.29	0.6631	0.4607	65.12
Picidae	*Piculus simplex*	Neotropics	0	0.5938	5.4683	4.40	0.7300	0.5296	6.43
Picidae	*Picumnus albosquamatus*	Neotropics	0	-0.0510	8.9232	3.57	0.7537	0.4648	72.63
Picidae	*Picumnus aurifrons*	Neotropics	0	3.0528	4.8640	3.88	0.7385	0.2335	48.65
Picidae	*Picumnus castelnau*	Neotropics	0	-0.4436	2.2275	2.84	0.7112	0.1779	35.50
Picidae	*Picumnus cinnamomeus*	Neotropics	0	2.6521	2.9192	4.07	0.6740	0.7885	47.43
Picidae	*Picumnus cirratus*	Neotropics	0	-0.4605	2.1829	3.04	0.7631	0.2944	18.32
Picidae	*Picumnus dorbignyanus*	Neotropics	0	-0.0079	5.2986	3.67	0.7567	0.3363	86.59
Picidae	*Picumnus exilis*	Neotropics	1	-7.6889	2.8271	3.71	0.7291	0.8951	26.92
Picidae	*Picumnus fulvescens*	Neotropics	0	0.2035	7.1886	3.20	0.6906	0.1803	0.37
Picidae	*Picumnus fuscus*	Neotropics	0	-0.4652	-	3.00	0.7593	0.2170	23.31
Picidae	*Picumnus granadensis*	Neotropics	0	-0.5077	3.2680	3.82	0.6523	0.3043	2.33
Picidae	*Picumnus innominatus*	Neotropics	0	-0.2746	6.9641	3.16	0.7987	0.5339	29.50

Family	Species	Realm	Threat	Latitude	Elevation	Anomaly	Size	Shape	Orientation
Picidae	*Picumnus lafresnayi*	Neotropics	0	-0.0523	-	3.78	0.7546	0.5037	25.37
Picidae	*Picumnus limae*	Palearctic	0	-0.4452	10.6352	4.10	0.8052	0.2698	2.58
Picidae	*Picumnus minutissimus*	Palearctic	0	-0.9993	11.3421	3.85	0.8110	0.3478	32.52
Picidae	*Picumnus nebulosus*	Palearctic	0	-0.6916	7.2877	3.67	0.8084	0.3402	0.28
Picidae	*Picumnus olivaceus*	Palearctic	0	-0.6540	3.7591	5.19	0.8600	0.1078	1.34
Picidae	*Picumnus pumilus*	Palearctic	0	-0.8327	-	3.60	0.7455	0.2831	62.30
Picidae	*Picumnus pygmaeus*	Afrotropics	0	0.2968	-	3.29	0.7472	0.2087	2.59
Picidae	*Picumnus rufiventris*	IndoMalay	0	-0.0723	7.9583	3.44	0.7659	0.2558	30.64
Picidae	*Picumnus sclateri*	IndoMalay	0	-0.0695	-	2.66	0.7069	0.2545	74.20
Picidae	*Picumnus spilogaster*	IndoMalay	0	-0.1101	12.0134	4.22	0.7480	0.1728	17.34
Picidae	*Picumnus squamulatus*	Palearctic	0	-0.9604	10.8597	3.67	0.7982	0.3844	41.95
Picidae	*Picumnus steindachneri*	Palearctic	0	-0.9331	4.8593	4.86	0.8614	0.1585	6.70
Picidae	*Picumnus subtilis*	Palearctic	0	-0.9116	-	4.01	0.8045	0.4905	30.23
Picidae	*Picumnus temminckii*	IndoMalay	0	-0.4320	8.5208	3.23	0.7842	0.5510	20.89
Picidae	*Picumnus varzeae*	Palearctic	0	0.2646	5.3269	3.46	0.6631	0.1575	21.74
Picidae	*Picus awokera*	Palearctic	0	-4.1999	8.7372	3.90	0.7725	0.5588	25.68
Picidae	*Picus canus*	Palearctic	0	0.1614	6.5677	4.33	0.7446	0.2425	0.06
Picidae	*Picus chlorolophus*	Palearctic	0	-0.9051	-	4.53	0.8519	0.3837	16.04
Picidae	*Picus erythropygius*	IndoMalay	0	-0.6686	5.8470	3.26	0.7674	0.4554	21.43
Picidae	*Picus flavinucha*	Palearctic	0	-1.0200	11.4963	4.38	0.8241	0.4294	15.51
Picidae	*Picus mentalis*	IndoMalay	1	-0.4673	4.8756	2.53	0.6428	0.4740	0.00
Picidae	*Picus mineaceus*	Afrotropics	0	0.3562	-	3.19	0.6974	0.4654	72.84
Picidae	*Picus puniceus*	Palearctic	0	-0.8640	11.1742	4.30	0.7929	0.6075	56.72
Picidae	*Picus rabieri*	Palearctic	0	-0.7463	14.5844	3.55	0.7677	0.4195	20.94
Picidae	*Picus squamatus*	Palearctic	0	-0.7245	7.0664	5.17	0.8495	0.2545	1.54
Picidae	*Picus vaillantii*	Palearctic	0	-1.2912	8.3620	3.86	0.7331	0.3795	34.72
Picidae	*Picus viridanus*	Afrotropics	0	2.1591	10.8651	3.44	0.7205	0.3437	79.43
Picidae	*Picus viridis*	Afrotropics	0	-1.8269	-	3.63	0.7570	0.3725	5.28
Picidae	*Picus vittatus*	Palearctic	0	-0.1886	9.3528	3.64	0.7779	0.2196	13.36
Picidae	*Picus xanthopygaeus*	Palearctic	0	-0.5963	10.4945	3.96	0.7777	0.2750	4.28
Picidae	*Reinwardtipicus validus*	IndoMalay	0	-0.0462	-	2.83	0.8028	0.8729	10.09
Picidae	*Sasia abnormis*	IndoMalay	1	-0.0933	-	2.65	0.6716	0.6458	83.32
Picidae	*Sasia africana*	IndoMalay	0	-0.0373	1.6095	2.81	0.7673	0.6206	44.97
Picidae	*Sasia ochracea*	Australasia	0	-0.0942	5.8750	2.62	0.6631	0.3500	5.24
Picidae	*Sphyrapicus nuchalis*	Australasia	0	-0.0659	-	2.80	0.7495	0.1730	24.91
Picidae	*Sphyrapicus ruber*	Australasia	0	-0.1550	-	2.83	0.7542	0.6212	55.88
Picidae	*Sphyrapicus thyroideus*	Australasia	0	-0.5275	-	3.09	0.7300	0.2706	70.49
Picidae	*Sphyrapicus varius*	Australasia	0	-0.5670	-	2.93	0.7977	0.3906	3.45
Picidae	*Thripias namaquus*	Neotropics	0	0.1204	9.0266	3.77	0.7300	0.1637	45.99
Picidae	*Thripias pyrrhogaster*	Neotropics	0	-0.1245	10.2731	3.26	0.7327	0.3509	55.41
Picidae	*Thripias xantholophus*	Neotropics	0	-0.0241	7.4267	3.65	0.7701	0.3881	76.09
Picidae	*Veniliornis affinis*	Afrotropics	0	3.9409	3.4540	3.38	0.7544	0.5461	12.64
Picidae	*Veniliornis callonotus*	Neotropics	0	-0.1932	7.1141	3.84	0.8404	0.6229	49.48
Picidae	*Veniliornis cassini*	Palearctic	0	0.0106	10.5356	4.12	0.7613	0.1908	4.76
Picidae	*Veniliornis chocoensis*	Neotropics	0	-0.4405	-	3.27	0.8199	0.6998	37.65
Picidae	*Veniliornis dignus*	Neotropics	0	-0.0055	9.8860	3.51	0.7807	0.1709	33.77
Picidae	*Veniliornis frontalis*	Neotropics	0	-0.3183	6.7172	2.47	0.5985	0.0000	0.00
Picidae	*Veniliornis kirkii*	IndoMalay	0	-0.1095	-	3.50	0.7854	0.1962	17.67
Picidae	*Veniliornis lignarius*	Palearctic	0	-0.8456	5.0109	3.33	0.7707	0.1421	16.88
Picidae	*Veniliornis maculifrons*	Neotropics	0	-0.2338	13.6481	3.56	0.7954	0.2640	68.46
Picidae	*Veniliornis mixtus*	Palearctic	0	-0.8892	5.8520	3.71	0.7399	0.2125	72.68
Picidae	*Veniliornis nigriceps*	IndoMalay	0	0.0702	7.1125	3.23	0.7781	0.5926	5.28
Picidae	*Veniliornis passerinus*	Neotropics	0	-0.6801	-	2.35	0.7833	0.4241	88.26
Picidae	*Veniliornis sanguineus*	Palearctic	1	-0.7706	4.9007	3.82	0.7378	0.4263	34.55
Picidae	*Veniliornis spilogaster*	Neotropics	0	-0.0574	6.3941	3.39	0.7839	0.3014	56.91
Pipridae	*Antilophia galeata*	Neotropics	0	-0.2764	2.8495	3.94	0.8021	0.7274	45.14
Pipridae	*Chiroxiphia boliviana*	Australasia	0	-0.0149	-	2.72	0.7416	0.1558	15.17
Pipridae	*Chiroxiphia caudata*	Australasia	1	0.1329	0.9479	2.49	0.5788	0.0000	0.00
Pipridae	*Chiroxiphia lanceolata*	Australasia	0	-0.0711	6.7283	2.81	0.7503	0.1640	23.82
Pipridae	*Chiroxiphia pareola*	Neotropics	0	-0.1411	1.6636	3.45	0.7151	0.6393	16.21
Pipridae	*Corapipo altera*	Australasia	0	-0.0528	-	2.81	0.7482	0.1835	18.31
Pipridae	*Corapipo gutturalis*	Australasia	0	0.1674	-	2.71	0.7185	0.5623	60.71
Pipridae	*Corapipo leucorrhoa*	Australasia	0	-0.5093	-	3.33	0.8380	0.5912	7.17
Pipridae	*Heterocercus aurantiivertex*	Neotropics	1	-0.3200	1.8301	3.34	0.7050	-	-
Pipridae	*Heterocercus aurantiivertex*	Neotropics	1	-0.3200	1.8301	3.34	0.7050	0.2516	43.50
Pipridae	*Heterocercus flavivertex*	Neotropics	0	-0.2510	4.0422	3.24	0.7647	0.3777	50.94
Pipridae	*Heterocercus linteatus*	Neotropics	0	-0.5266	9.5242	2.73	0.7452	0.4103	35.57
Pipridae	*Ilicura militaris*	IndoMalay	0	-0.0632	0.7789	3.06	0.8277	0.4132	28.40
Pipridae	*Lepidothrix coeruleocapilla*	Palearctic	0	-0.5371	12.8319	3.98	0.7975	0.5975	5.09
Pipridae	*Lepidothrix coronata*	Palearctic	0	-0.8108	-	3.66	0.8120	0.4581	23.85
Pipridae	*Lepidothrix iris*	IndoMalay	0	-0.0009	6.8918	2.65	0.6921	0.1544	69.70
Pipridae	*Lepidothrix isidorei*	IndoMalay	0	-0.3660	-	3.72	0.8226	0.5243	35.58
Pipridae	*Lepidothrix nattereri*	Palearctic	0	-0.7371	4.8732	4.92	0.8681	0.0958	0.21
Pipridae	*Lepidothrix serena*	Palearctic	0	-0.4830	-	3.52	0.6891	0.2044	3.83

Family	Species	Realm	Threat	Latitude	Elevation	Anomaly	Size	Shape	Orientation
Pipridae	*Lepidothrix suavissima*	IndoMalay	0	-0.0830	-	3.11	0.8032	0.4952	17.80
Pipridae	*Lepidothrix vilasboasi*	Australasia	1	-0.4732	0.0000	2.32	0.7069	0.1858	42.92
Pipridae	*Machaeropterus pyrocephalus*	IndoMalay	0	-0.3858	-	3.09	0.7864	0.5759	28.86
Pipridae	*Machaeropterus regulus*	IndoMalay	0	-0.8764	7.3317	2.61	0.6523	0.3873	66.90
Pipridae	*Manacus aurantiacus*	Afrotropics	1	-15.5782	5.7830	3.19	0.6478	0.4630	33.86
Pipridae	*Manacus candei*	Neotropics	0	-0.5472	7.6347	2.87	0.7654	0.2410	39.94
Pipridae	*Manacus manacus*	Australasia	0	-0.3420	-	2.92	0.7958	0.3989	51.76
Pipridae	*Manacus vitellinus*	IndoMalay	0	0.0192	4.1500	2.83	0.7725	0.6556	20.11
Pipridae	*Masius chrysopterus*	IndoMalay	0	-0.0151	3.7590	2.81	0.7846	0.5856	4.22
Pipridae	*Neopelma aurifrons*	Afrotropics	0	-0.3879	-	3.91	0.7885	0.3466	57.55
Pipridae	*Neopelma chrysocephalum*	Afrotropics	0	-0.0376	9.0762	3.52	0.7973	0.7040	57.96
Pipridae	*Neopelma chrysolophum*	Afrotropics	0	-0.1122	-	3.30	0.7922	0.4942	43.56
Pipridae	*Neopelma pallescens*	Afrotropics	0	0.1519	-	3.22	0.8001	0.2309	12.23
Pipridae	*Neopelma sulphureiventer*	Afrotropics	0	8.6585	14.5273	3.37	0.6961	0.8544	84.90
Pipridae	*Pipra aureola*	Palearctic	0	-0.8664	9.1022	3.75	0.7590	0.2614	28.86
Pipridae	*Pipra chloromeros*	Neotropics	1	-0.2440	1.4941	3.32	0.6921	0.1284	62.10
Pipridae	*Pipra cornuta*	Neotropics	0	-0.4295	9.9768	2.39	0.7606	0.1481	87.22
Pipridae	*Pipra erythrocephala*	Neotropics	0	-0.3933	6.2798	2.90	0.7987	0.4940	81.38
Pipridae	*Pipra fasciicauda*	Neotropics	0	-0.1760	6.6312	3.78	0.8556	0.4492	37.87
Pipridae	*Pipra filicauda*	Neotropics	0	-0.0210	-	4.07	0.8267	0.7725	21.37
Pipridae	*Pipra mentalis*	Nearctic	0	-0.1741	-	3.36	0.7172	0.3833	59.95
Pipridae	*Pipra pipra*	Palearctic	0	-0.6346	-	4.39	0.8766	0.2865	2.31
Pipridae	*Pipra rubrocapilla*	Afrotropics	1	0.4769	2.8644	3.09	0.7539	0.2888	11.31
Pipridae	*Piprites chloris*	Nearctic	0	-0.7293	12.7597	4.14	0.8552	0.4433	18.54
Pipridae	*Piprites griseiceps*	Neotropics	0	-0.5322	7.1826	2.93	0.7744	0.3465	28.80
Pipridae	*Piprites pileata*	Neotropics	0	0.0842	3.0737	2.90	0.6478	0.4061	17.05
Pipridae	*Tyranneutes stolzmanni*	Australasia	0	-0.0827	3.2425	2.71	0.7185	0.5623	60.71
Pipridae	*Tyranneutes virescens*	Afrotropics	0	0.0693	8.0563	3.32	0.7740	0.3808	81.92
Pipridae	*Xenopipo atronitens*	Neotropics	0	-0.0756	3.6716	4.09	0.8234	0.7012	70.43
Pipridae	*Xenopipo flavicapilla*	Neotropics	0	-0.2497	-	2.50	0.7223	0.1893	18.63
Pipridae	*Xenopipo holochlora*	Neotropics	0	-0.3279	-	3.45	0.7618	0.4049	38.13
Pipridae	*Xenopipo unicolor*	Neotropics	0	-0.2170	8.8139	3.85	0.7629	0.1898	50.13
Pipridae	*Xenopipo uniformis*	Neotropics	1	-0.3589	0.5150	2.53	0.6974	0.1632	17.43
Pittidae	*Pitta anerythra*	Neotropics	0	-0.0203	-	3.33	0.7681	0.6197	50.22
Pittidae	*Pitta angolensis*	Neotropics	0	0.3923	-	3.82	0.7392	0.4280	25.40
Pittidae	*Pitta arcuata*	Neotropics	0	-0.1891	-	3.61	0.7767	0.7061	48.38
Pittidae	*Pitta baudii*	Neotropics	0	0.0498	-	4.00	0.7881	0.3580	54.71
Pittidae	*Pitta brachyura*	Neotropics	0	0.0787	-	3.16	0.6921	0.2637	82.09
Pittidae	*Pitta caerulea*	Neotropics	0	-0.1429	-	3.86	0.7480	0.4272	19.56
Pittidae	*Pitta cyanea*	Neotropics	0	-0.2270	-	3.96	0.7597	0.3849	32.55
Pittidae	*Pitta dohertyi*	Neotropics	1	0.0000	6.1609	3.67	0.6428	0.7896	46.14
Pittidae	*Pitta elegans*	Neotropics	0	-30.7066	-	4.14	0.6948	0.3058	26.64
Pittidae	*Pitta elliotii*	Neotropics	0	-0.3636	-	3.01	0.7772	0.2841	33.89
Pittidae	*Pitta erythrogaster*	Neotropics	0	-0.1075	-	4.27	0.6824	0.2261	21.62
Pittidae	*Pitta granatina*	Palearctic	0	-0.7859	4.7583	3.16	0.7359	0.2344	32.11
Pittidae	*Pitta guajana*	Palearctic	0	-0.4832	6.2893	4.14	0.8588	0.3474	11.97
Pittidae	*Pitta gurneyi*	IndoMalay	0	-0.0706	-	3.22	0.8205	0.6364	11.60
Pittidae	*Pitta irena*	IndoMalay	0	-0.0785	-	3.08	0.7666	0.3279	43.62
Pittidae	*Pitta iris*	IndoMalay	0	-0.0881	-	3.19	0.8152	0.7362	52.49
Pittidae	*Pitta kochi*	IndoMalay	0	0.0016	-	2.84	0.7828	0.4804	10.28
Pittidae	*Pitta maxima*	IndoMalay	0	0.0154	-	2.85	0.7884	0.5530	11.53
Pittidae	*Pitta megarhyncha*	IndoMalay	0	-0.0217	-	2.83	0.7910	0.5867	14.98
Pittidae	*Pitta moluccensis*	IndoMalay	0	0.0083	2.3166	2.99	0.7498	0.5244	75.68
Pittidae	*Pitta nipalensis*	Palearctic	0	-0.5187	14.3297	4.26	0.7800	0.3462	7.21
Pittidae	*Pitta nympha*	Palearctic	0	-0.7116	6.8148	3.79	0.7544	0.1873	14.52
Pittidae	*Pitta oatesi*	IndoMalay	0	-0.0516	-	3.00	0.7522	0.1802	65.46
Pittidae	*Pitta phayrei*	Palearctic	0	-0.5176	-	3.98	0.8362	0.4329	1.15
Pittidae	*Pitta reichenowi*	IndoMalay	0	-0.0503	3.7365	2.99	0.7920	0.3238	75.83
Pittidae	*Pitta schneideri*	IndoMalay	0	-0.1341	-	3.30	0.8109	0.5323	5.71
Pittidae	*Pitta schwaneri*	Neotropics	0	-0.5670	-	3.18	0.6763	0.3181	68.82
Pittidae	*Pitta sordida*	Neotropics	0	-0.1541	-	3.97	0.8470	0.6424	37.56
Pittidae	*Pitta soror*	Afrotropics	0	0.1510	-	3.46	0.8183	0.1957	7.55
Pittidae	*Pitta steerii*	Afrotropics	0	0.0352	-	3.71	0.8022	0.5793	4.75
Pittidae	*Pitta ussheri*	Afrotropics	0	-0.2741	-	3.77	0.7505	0.4707	63.11
Pittidae	*Pitta venusta*	Palearctic	0	-0.8139	-	4.80	0.8645	0.0649	0.68
Pittidae	*Pitta versicolor*	Palearctic	0	-0.5628	12.0771	3.97	0.7841	0.3316	6.16
Pityriaseidae	*Pityriasis gymnocephala*	Neotropics	0	-0.3064	-	2.88	0.7741	0.3705	34.01
Platysteiridae	*Batis capensis*	IndoMalay	0	-0.1588	-	2.65	0.6120	0.3586	79.16
Platysteiridae	*Batis crypta*	Neotropics	0	-0.7635	-	2.95	0.7695	0.4780	1.77
Platysteiridae	*Batis diops*	Afrotropics	0	0.1868	-	3.06	0.7409	0.2731	12.24
Platysteiridae	*Batis fratrum*	Afrotropics	0	0.3569	-	3.26	0.7758	0.2428	4.88
Platysteiridae	*Batis ituriensis*	Afrotropics	1	1.1002	6.7514	3.23	0.6662	0.5111	65.22
Platysteiridae	*Batis margaritae*	Afrotropics	0	-0.4056	10.0871	3.39	0.7705	0.2993	57.74
Platysteiridae	*Batis minima*	Afrotropics	0	0.4297	8.6338	3.38	0.7087	0.3764	51.45

Family	Species	Realm	Threat	Latitude	Elevation	Anomaly	Size	Shape	Orientation
Platysteiridae	*Batis minor*	Afrotropics	0	7.8253	11.3004	3.43	0.7144	0.3413	82.90
Platysteiridae	*Batis minulla*	Afrotropics	0	-0.3088	2.2954	3.33	0.7550	0.3193	59.88
Platysteiridae	*Batis mixta*	Afrotropics	0	-1.4220	-	3.34	0.7458	0.7871	39.10
Platysteiridae	*Batis molitor*	Afrotropics	0	11.4147	-	3.74	0.7158	0.2118	1.91
Platysteiridae	*Batis molitor*	Afrotropics	0	11.4147	-	3.74	0.7158	-	-
Platysteiridae	*Batis occulta*	Afrotropics	0	-0.1904	3.8696	3.16	0.6631	0.9097	70.45
Platysteiridae	*Batis orientalis*	Afrotropics	0	0.0906	0.0000	3.42	0.8278	0.6348	23.77
Platysteiridae	*Batis perkeo*	Afrotropics	0	-0.1115	-	3.34	0.7632	0.5941	74.64
Platysteiridae	*Batis poensis*	Afrotropics	0	-0.0404	8.5270	3.09	0.7060	0.5600	88.32
Platysteiridae	*Batis pririt*	Afrotropics	0	-0.1099	-	3.66	0.8304	0.7481	65.01
Platysteiridae	*Batis senegalensis*	Afrotropics	0	0.1434	9.4258	3.14	0.7659	0.1345	10.78
Platysteiridae	*Batis soror*	Afrotropics	0	-0.0700	-	3.54	0.8142	0.4084	4.00
Platysteiridae	*Bias musicus*	Afrotropics	1	-0.5137	3.9577	3.41	0.6370	0.6422	17.66
Platysteiridae	*Lanioturdus torquatus*	Afrotropics	0	-0.1340	-	3.16	0.7720	0.1679	69.59
Platysteiridae	*Megabyas flammulatus*	IndoMalay	0	0.0036	-	2.83	0.7626	0.5662	1.58
Platysteiridae	*Platysteira albifrons*	Neotropics	0	-0.1188	4.4714	3.95	0.8288	0.5985	9.86
Platysteiridae	*Platysteira blissetti*	Neotropics	0	-0.1389	-	4.04	0.8179	0.4539	5.24
Platysteiridae	*Platysteira castanea*	Neotropics	0	-0.2127	-	3.39	0.8095	0.4198	46.36
Platysteiridae	*Platysteira chalybea*	Neotropics	0	0.0633	11.6443	3.69	0.7609	0.3996	80.25
Platysteiridae	*Platysteira concreta*	Neotropics	0	-0.0596	11.5436	3.74	0.7151	0.4087	34.48
Platysteiridae	*Platysteira cyanea*	Neotropics	0	-0.0013	4.8334	3.58	0.7211	0.2353	82.07
Platysteiridae	*Platysteira jamesoni*	Neotropics	0	-0.0908	7.7544	3.76	0.6716	0.1661	1.42
Platysteiridae	*Platysteira laticincta*	Neotropics	0	-7.0132	7.1468	3.86	0.7356	0.1949	49.67
Platysteiridae	*Platysteira peltata*	Neotropics	0	-1.4977	10.9893	4.05	0.7335	0.1294	35.18
Platysteiridae	*Platysteira tonsa*	Neotropics	0	0.0032	8.3902	3.04	0.6804	0.1583	64.02
Ploceidae	*Amblyospiza albifrons*	Australasia	0	-0.5753	-	2.61	0.6690	0.2544	36.90
Ploceidae	*Anaplectes rubriceps*	Afrotropics	0	-0.0809	-	3.62	0.8351	0.8239	69.62
Ploceidae	*Brachycope anomala*	Nearctic	0	-0.8253	6.5379	4.30	0.8438	0.4074	5.06
Ploceidae	*Bubalornis albirostris*	Neotropics	0	-0.5127	-	3.86	0.8214	0.6203	33.23
Ploceidae	*Bubalornis niger*	Neotropics	0	-0.0951	2.9063	4.04	0.8145	0.8337	41.10
Ploceidae	*Dinemellia dinemelli*	IndoMalay	0	-0.0526	-	3.16	0.8097	0.5406	62.34
Ploceidae	*Euplectes afer*	Neotropics	0	-0.5026	3.7688	2.98	0.7732	0.5626	28.74
Ploceidae	*Euplectes albonotatus*	Neotropics	0	-0.2022	-	3.73	0.8448	0.8520	52.45
Ploceidae	*Euplectes ardens*	Neotropics	0	0.2036	-	4.05	0.8304	0.7641	12.37
Ploceidae	*Euplectes aureus*	Neotropics	0	13.9062	-	3.28	0.6804	0.2353	69.13
Ploceidae	*Euplectes axillaris*	Neotropics	0	-0.3281	-	3.47	0.8134	0.4505	44.02
Ploceidae	*Euplectes capensis*	Neotropics	0	-0.0133	8.3277	3.69	0.7713	0.1835	30.00
Ploceidae	*Euplectes diadematus*	Neotropics	0	-0.2498	-	4.01	0.7469	0.5191	1.73
Ploceidae	*Euplectes franciscanus*	Neotropics	0	0.2293	-	3.18	0.7367	0.5469	62.24
Ploceidae	*Euplectes gierowii*	Neotropics	0	-0.0386	-	3.47	0.7522	0.2772	29.32
Ploceidae	*Euplectes hartlaubi*	Neotropics	0	-0.0556	-	3.41	0.7695	0.3571	32.66
Ploceidae	*Euplectes hordeaceus*	Neotropics	0	-0.2544	-	3.01	0.6370	0.2651	32.26
Ploceidae	*Euplectes jacksoni*	Neotropics	0	-0.2956	-	2.47	0.5985	0.0000	0.00
Ploceidae	*Euplectes macroura*	Neotropics	0	0.0061	-	3.97	0.8241	0.5797	56.58
Ploceidae	*Euplectes nigroventris*	Neotropics	0	-0.2496	-	3.09	0.7144	0.3741	32.44
Ploceidae	*Euplectes orix*	Neotropics	0	-2.6047	6.3686	3.80	0.7512	0.3498	60.76
Ploceidae	*Euplectes progne*	Neotropics	0	0.0338	-	3.97	0.8280	0.7851	34.53
Ploceidae	*Euplectes psammocromius*	Neotropics	0	0.0376	-	2.46	0.6906	0.2797	2.64
Ploceidae	*Foudia madagascariensis*	Neotropics	0	-0.0253	2.5282	3.96	0.8411	-	-
Ploceidae	*Foudia madagascariensis*	Neotropics	0	-0.0253	2.5282	3.96	0.8411	0.6193	31.05
Ploceidae	*Foudia omissa*	Neotropics	0	-0.2890	3.2642	3.71	0.8310	0.4867	53.16
Ploceidae	*Malimbus ballmanni*	Neotropics	0	-0.5452	-	2.93	0.7753	0.3437	32.51
Ploceidae	*Malimbus cassini*	Neotropics	0	-0.2763	5.9702	3.18	0.7809	0.3624	28.95
Ploceidae	*Malimbus coronatus*	Neotropics	0	-0.6796	7.4783	4.30	0.6997	0.1846	1.22
Ploceidae	*Malimbus erythrogaster*	Neotropics	1	0.0130	3.0894	3.69	0.7060	0.4043	64.53
Ploceidae	*Malimbus ibadanensis*	Australasia	1	-0.1187	3.4535	2.71	0.7185	0.5623	60.71
Ploceidae	*Malimbus malimbicus*	Afrotropics	0	0.0166	5.6655	3.39	0.8208	0.1658	8.09
Ploceidae	*Malimbus nitens*	Afrotropics	0	-0.1644	14.1753	3.70	0.8178	0.7278	12.50
Ploceidae	*Malimbus racheliae*	Neotropics	0	-0.3968	0.7493	2.24	0.7760	0.0675	1.96
Ploceidae	*Malimbus rubricollis*	Neotropics	0	-0.5557	0.0000	2.27	0.7764	0.0699	1.88
Ploceidae	*Malimbus scutatus*	IndoMalay	0	0.0696	2.4725	2.83	0.7510	0.4744	54.01
Ploceidae	*Ploceus albinucha*	Neotropics	0	-0.0132	9.1917	3.69	0.7846	0.4279	51.79
Ploceidae	*Ploceus alienus*	Nearctic	0	-0.8718	8.3269	3.96	0.8220	0.5765	56.46
Ploceidae	*Ploceus angolensis*	Nearctic	0	-0.7318	0.0000	4.33	0.8121	0.5179	4.54
Ploceidae	*Ploceus aurantius*	Neotropics	0	-0.2471	0.0000	3.08	0.7128	0.5580	44.89
Ploceidae	*Ploceus aureonucha*	Nearctic	0	-1.1796	4.4497	4.08	0.8173	0.3428	11.02
Ploceidae	*Ploceus badius*	Neotropics	0	-0.0841	7.4685	3.35	0.7344	0.2339	75.21
Ploceidae	*Ploceus baglafecht*	Neotropics	0	-0.1139	1.6121	3.98	0.8442	0.6097	31.38
Ploceidae	*Ploceus bannermani*	Neotropics	0	-0.2342	-	3.68	0.8588	0.5428	46.83
Ploceidae	*Ploceus batesi*	IndoMalay	1	-0.0122	7.0683	2.64	0.7151	0.2337	68.90
Ploceidae	*Ploceus benghalensis*	Neotropics	0	0.0593	-	3.95	0.8153	0.4590	17.15
Ploceidae	*Ploceus bertrandi*	Neotropics	0	0.0000	0.3192	3.73	0.6302	0.4947	37.39
Ploceidae	*Ploceus bicolor*	Australasia	0	-0.0927	-	2.78	0.7528	0.1928	23.61
Ploceidae	*Ploceus bojeri*	Australasia	0	-0.0791	-	2.78	0.7567	0.1890	23.83

Family	Species	Realm	Threat	Latitude	Elevation	Anomaly	Size	Shape	Orientation
Ploceidae	*Ploceus burnieri*	Australasia	0	-0.0962	-	2.77	0.7700	0.3111	23.72
Ploceidae	*Ploceus capensis*	Australasia	0	-0.0551	0.1213	2.90	0.7185	0.2488	24.32
Ploceidae	*Ploceus capensis*	Australasia	0	-0.0551	0.1213	2.90	0.7185	-	-
Ploceidae	*Ploceus castaneiceps*	Australasia	0	-0.0955	-	2.78	0.7666	0.3067	23.95
Ploceidae	*Ploceus castanops*	Australasia	0	-0.0901	-	2.82	0.7399	0.1530	23.70
Ploceidae	*Ploceus cucullatus*	Australasia	1	-1.2567	1.8292	2.50	0.5788	0.0000	45.57
Ploceidae	*Ploceus dichrocephalus*	Afrotropics	0	-0.0503	3.1606	3.30	0.7903	0.1601	31.85
Ploceidae	*Ploceus dorsomaculatus*	IndoMalay	0	0.1214	5.8500	2.86	0.7567	0.4735	43.49
Ploceidae	*Ploceus flavipes*	IndoMalay	1	0.0784	2.6547	2.84	0.7676	0.6488	45.81
Ploceidae	*Ploceus galbula*	IndoMalay	0	-0.1410	-	3.56	0.7797	0.8165	33.74
Ploceidae	*Ploceus golandi*	IndoMalay	0	0.0136	2.5574	2.86	0.7838	0.5805	10.90
Ploceidae	*Ploceus heuglini*	IndoMalay	0	-0.1168	7.3508	3.11	0.7864	0.4755	46.97
Ploceidae	*Ploceus insignis*	Australasia	0	-0.0003	-	2.60	0.6891	0.1573	2.63
Ploceidae	*Ploceus intermedius*	IndoMalay	0	-0.1751	2.1371	3.00	0.7691	0.4425	76.22
Ploceidae	*Ploceus jacksoni*	Australasia	0	-0.0289	-	2.72	0.7875	0.3651	33.72
Ploceidae	*Ploceus katangae*	IndoMalay	0	0.0518	0.5730	2.85	0.7797	0.4129	5.74
Ploceidae	*Ploceus luteolus*	IndoMalay	0	-0.3768	3.1809	2.71	0.7104	0.1572	11.52
Ploceidae	*Ploceus manyar*	IndoMalay	1	-0.0983	0.2282	2.89	0.6370	0.2279	88.39
Ploceidae	*Ploceus megarhynchus*	IndoMalay	0	-0.0104	2.3186	2.83	0.7642	0.3729	65.05
Ploceidae	*Ploceus melanocephalus*	Australasia	0	0.1232	1.2558	3.20	0.7389	0.2359	18.90
Ploceidae	*Ploceus melanogaster*	IndoMalay	1	-0.0652	8.7131	2.66	0.6974	0.4841	74.85
Ploceidae	*Ploceus nelicourvi*	Australasia	0	-0.0652	-	2.48	0.6302	0.2423	87.23
Ploceidae	*Ploceus nicolli*	IndoMalay	0	-0.0273	-	2.88	0.7419	0.1966	59.27
Ploceidae	*Ploceus nigerrimus*	IndoMalay	0	-0.0474	-	2.92	0.8085	0.5178	62.48
Ploceidae	*Ploceus nigricollis*	IndoMalay	0	-0.0023	4.7825	3.30	0.7744	0.3219	22.59
Ploceidae	*Ploceus nigrimentus*	Palearctic	1	-0.6784	3.4735	3.28	0.7875	0.2719	17.25
Ploceidae	*Ploceus ocularis*	IndoMalay	0	-0.1182	11.0433	3.08	0.7729	0.6954	77.71
Ploceidae	*Ploceus olivaceiceps*	IndoMalay	0	-0.1844	-	3.07	0.7822	0.7138	39.93
Ploceidae	*Ploceus pelzelni*	Afrotropics	0	0.3306	1.8492	3.29	0.7707	0.2743	2.17
Ploceidae	*Ploceus philippinus*	IndoMalay	1	0.0843	8.2805	2.77	0.7211	0.1083	54.63
Ploceidae	*Ploceus preussi*	IndoMalay	0	0.0888	5.6541	2.84	0.7685	0.6418	46.68
Ploceidae	*Ploceus reichardi*	IndoMalay	0	-0.0498	-	3.03	0.8216	0.3097	32.37
Ploceidae	*Ploceus rubiginosus*	IndoMalay	0	-0.3007	5.8384	3.03	0.7685	0.4341	82.08
Ploceidae	*Ploceus ruweti*	IndoMalay	1	-0.0413	2.1021	2.62	0.7019	0.6235	86.79
Ploceidae	*Ploceus sakalava*	IndoMalay	0	-0.0586	0.9777	2.69	0.6784	0.6287	28.04
Ploceidae	*Ploceus spekei*	IndoMalay	1	0.0147	5.1208	2.77	0.7245	0.0977	53.64
Ploceidae	*Ploceus spekeoides*	Australasia	0	-0.4370	4.9638	3.12	0.7595	0.2246	66.61
Ploceidae	*Ploceus subaureus*	Neotropics	0	-0.2410	-	2.97	0.6997	0.1839	18.88
Ploceidae	*Ploceus subpersonatus*	Neotropics	0	0.2552	2.4848	2.96	0.7040	0.1784	69.70
Ploceidae	*Ploceus superciliosus*	IndoMalay	0	0.1185	2.6656	2.84	0.7685	0.6418	46.68
Ploceidae	*Ploceus taeniopterus*	Neotropics	0	-0.2819	2.3045	3.56	0.8415	0.4625	61.21
Ploceidae	*Ploceus temporalis*	Afrotropics	0	-0.1085	-	3.53	0.8557	0.6659	37.90
Ploceidae	*Ploceus tricolor*	Australasia	0	-0.5287	0.0000	3.36	0.8274	0.5401	7.51
Ploceidae	*Ploceus velatus*	Palearctic	0	-0.6120	-	4.16	0.8380	0.3646	6.61
Ploceidae	*Ploceus vitellinus*	Australasia	0	-0.3873	-	3.25	0.8331	0.5280	27.68
Ploceidae	*Ploceus weynsi*	Australasia	0	-0.3404	-	3.42	0.7923	0.3913	62.91
Ploceidae	*Ploceus xanthops*	Australasia	0	-0.2857	-	2.17	0.6804	0.6686	14.77
Ploceidae	*Ploceus xanthopterus*	Australasia	0	-0.5458	6.2435	2.99	0.7771	0.7253	58.72
Ploceidae	*Quelea cardinalis*	Australasia	0	-0.3738	-	3.06	0.7654	0.3231	29.98
Ploceidae	*Quelea erythrops*	Australasia	0	0.0438	12.0234	2.77	0.7548	0.2208	15.80
Ploceidae	*Quelea quelea*	Australasia	0	-0.2272	-	2.49	0.6824	0.2324	22.19
Podargidae	*Batrachostomus affinis*	Afrotropics	0	-0.0668	-	3.26	0.7826	0.6322	33.36
Podargidae	*Batrachostomus cornutus*	Afrotropics	0	-0.2445	-	4.06	0.7967	0.5234	52.92
Podargidae	*Batrachostomus harterti*	Afrotropics	0	0.2479	-	3.53	0.8154	0.3392	0.61
Podargidae	*Batrachostomus hodgsoni*	Afrotropics	0	-0.1368	-	3.31	0.7781	0.2732	73.82
Podargidae	*Batrachostomus javensis*	IndoMalay	0	0.0089	-	2.90	0.7899	0.4012	46.46
Podargidae	*Batrachostomus mixtus*	IndoMalay	0	0.0900	-	2.85	0.7767	0.4448	2.53
Podargidae	*Batrachostomus moniliger*	IndoMalay	0	0.0757	-	2.84	0.7776	0.4123	8.14
Podargidae	*Batrachostomus poliolophus*	IndoMalay	0	0.1117	3.5700	2.88	0.7474	0.5910	48.01
Podargidae	*Batrachostomus septimus*	IndoMalay	0	-0.1124	7.5475	3.22	0.7438	0.2535	32.11
Podargidae	*Batrachostomus stellatus*	IndoMalay	0	-0.0081	-	2.84	0.7643	0.5286	59.49
Podargidae	*Podargus ocellatus*	Neotropics	0	-0.0048	-	3.98	0.8051	0.4441	16.06
Podargidae	*Podargus papuensis*	IndoMalay	0	0.0118	1.7692	2.84	0.7895	0.5271	11.64
Podicipedidae	*Aechmophorus clarkii*	Nearctic	0	-0.7267	9.3673	4.13	0.8193	0.6854	60.79
Podicipedidae	*Aechmophorus occidentalis*	Nearctic	0	-0.6956	9.1334	4.13	0.8277	0.7839	52.29
Podicipedidae	*Podiceps gallardoi*	Afrotropics	0	0.2300	0.0000	3.10	0.7616	0.1831	5.06
Podicipedidae	*Podiceps major*	Afrotropics	0	0.0329	-	3.31	0.8226	0.3812	14.93
Podicipedidae	*Podiceps occipitalis*	Afrotropics	0	0.2021	0.0000	3.21	0.7650	0.5695	89.80
Podicipedidae	*Podiceps taczanowskii*	Afrotropics	0	0.0094	-	3.23	0.7903	0.3461	12.08
Podicipedidae	*Podilymbus podiceps*	Afrotropics	0	0.1196	-	3.33	0.8324	0.3085	9.00
Podicipedidae	*Poliocephalus poliocephalus*	Afrotropics	0	0.0594	-	3.28	0.8135	0.3143	16.11
Podicipedidae	*Poliocephalus rufopectus*	Afrotropics	0	0.0072	-	3.28	0.8227	0.2802	13.07
Podicipedidae	*Rollandia microptera*	Neotropics	0	-0.1912	-	3.06	0.7560	0.2249	54.70
Podicipedidae	*Rollandia rolland*	Neotropics	0	-0.1272	-	3.97	0.8473	0.6181	35.03

Family	Species	Realm	Threat	Latitude	Elevation	Anomaly	Size	Shape	Orientation
Podicipedidae	*Tachybaptus dominicus*	Neotropics	0	0.0463	0.6770	3.94	0.7792	0.1788	12.86
Podicipedidae	*Tachybaptus novaehollandiae*	Australasia	0	-0.5388	6.2262	3.48	0.8164	0.4413	33.33
Podicipedidae	*Tachybaptus pelzelnii*	Afrotropics	0	-0.0994	5.3647	3.24	0.8132	0.2163	11.60
Podicipedidae	*Tachybaptus ruficollis*	Australasia	0	-0.6103	-	2.76	0.7724	0.2606	4.36
Polioptilidae	*Microbates cinereiventris*	Neotropics	0	0.0851	11.3998	3.56	0.7192	0.3073	34.20
Polioptilidae	*Microbates collaris*	Nearctic	0	-0.4481	8.3998	3.27	0.7505	0.1280	53.87
Polioptilidae	*Polioptila albiloris*	Afrotropics	0	-0.1348	-	3.50	0.8225	0.7148	73.18
Polioptilidae	*Polioptila caerulea*	Afrotropics	0	0.0698	8.1020	3.55	0.7419	0.4498	85.30
Polioptilidae	*Polioptila californica*	Afrotropics	0	-0.0670	-	3.27	0.7780	0.3677	6.44
Polioptilidae	*Polioptila clementsi*	IndoMalay	0	-0.0637	-	3.30	0.8335	0.4600	30.63
Polioptilidae	*Polioptila dumicola*	Afrotropics	0	-0.0829	-	3.23	0.7731	0.1359	11.46
Polioptilidae	*Polioptila guianensis*	Afrotropics	0	-23.4965	-	3.51	0.6891	0.7531	31.97
Polioptilidae	*Polioptila lactea*	Afrotropics	0	-0.0474	-	3.51	0.7946	0.2326	45.42
Polioptilidae	*Polioptila lembeyei*	Afrotropics	0	0.0000	-	3.62	0.6120	0.9804	90.00
Polioptilidae	*Polioptila melanura*	Afrotropics	0	-0.1391	-	3.21	0.7422	0.2883	72.92
Polioptilidae	*Polioptila nigriceps*	Afrotropics	0	0.0719	-	3.37	0.7659	0.3877	48.67
Polioptilidae	*Polioptila plumbea*	Afrotropics	0	6.5767	-	3.29	0.6986	0.5932	17.23
Polioptilidae	*Polioptila schistaceigula*	Afrotropics	0	-0.1195	4.1789	3.21	0.7692	0.1718	74.18
Polioptilidae	*Ramphocaenus melanurus*	Nearctic	1	-0.4489	0.5554	2.91	0.7701	0.1676	14.32
Pomatostomidae	*Pomatostomus halli*	Neotropics	0	-0.4567	8.7713	3.78	0.8698	0.3374	54.96
Pomatostomidae	*Pomatostomus isidorei*	Palearctic	0	-0.7736	5.9605	4.33	0.7951	0.3018	3.85
Pomatostomidae	*Pomatostomus ruficeps*	Palearctic	0	-0.9916	-	4.06	0.7477	0.2939	14.47
Pomatostomidae	*Pomatostomus superciliosus*	Palearctic	0	0.7983	0.0000	4.18	0.7488	0.4854	58.33
Pomatostomidae	*Pomatostomus temporalis*	Australasia	0	-0.0712	8.8519	2.81	0.7389	0.1497	24.08
Promeropidae	*Promerops cafer*	Neotropics	0	-78.8269	6.1699	4.14	0.6784	0.6030	13.89
Promeropidae	*Promerops gurneyi*	Neotropics	1	-0.3689	4.6073	3.91	0.7914	0.6464	7.54
Prunellidae	*Prunella atrogularis*	Neotropics	0	-0.6326	-	3.23	0.7937	0.5569	83.70
Prunellidae	*Prunella collaris*	Neotropics	0	-0.5321	4.5735	2.76	0.7952	0.9152	62.12
Prunellidae	*Prunella fagani*	Neotropics	0	-0.7277	-	2.95	0.7582	0.5052	84.13
Prunellidae	*Prunella fulvescens*	Neotropics	1	-0.0401	7.9483	3.64	0.6935	0.3038	64.71
Prunellidae	*Prunella himalayana*	Neotropics	0	-0.5467	7.2667	2.69	0.7165	0.2968	39.81
Prunellidae	*Prunella immaculata*	Neotropics	0	-0.3899	-	3.09	0.7822	0.2734	88.22
Prunellidae	*Prunella koslowi*	Afrotropics	0	-0.0536	7.5742	3.53	0.8591	0.5727	32.33
Prunellidae	*Prunella modularis*	Neotropics	0	-0.1313	-	3.98	0.8305	0.6328	53.50
Prunellidae	*Prunella montanella*	Australasia	1	0.0359	4.7256	1.95	0.6221	0.7062	38.71
Prunellidae	*Prunella rubida*	Neotropics	0	-0.2995	3.2980	3.72	0.8592	0.4549	44.07
Prunellidae	*Prunella strophiata*	Afrotropics	0	-0.3727	7.2750	3.37	0.8636	0.4269	8.10
Psittacidae	*Agapornis canus*	Neotropics	0	-0.0356	9.7159	4.01	0.8403	0.5698	36.92
Psittacidae	*Agapornis fischeri*	Afrotropics	0	-0.0926	-	3.15	0.7550	0.3939	69.52
Psittacidae	*Agapornis lilianae*	Afrotropics	0	4.4993	6.3529	3.25	0.7399	0.6909	14.46
Psittacidae	*Agapornis nigrigenis*	Afrotropics	0	7.9542	-	3.60	0.7413	0.4359	51.67
Psittacidae	*Agapornis personatus*	Afrotropics	1	21.0174	-	4.07	0.6598	0.6091	47.85
Psittacidae	*Agapornis pullarius*	Afrotropics	0	3.6067	4.1856	3.29	0.7348	0.5333	63.04
Psittacidae	*Agapornis roseicollis*	Afrotropics	0	-0.0134	-	3.33	0.8144	0.3925	10.08
Psittacidae	*Agapornis swindernianus*	Afrotropics	0	-0.1712	-	4.06	0.7642	0.2395	62.22
Psittacidae	*Agapornis taranta*	Afrotropics	0	0.0959	2.9260	3.27	0.7952	0.2498	9.50
Psittacidae	*Alipiopsitta xanthops*	Afrotropics	0	6.0346	9.7475	3.43	0.7096	0.2728	79.01
Psittacidae	*Alisterus amboinensis*	Neotropics	0	-0.2494	3.5095	3.99	0.8030	0.6025	37.62
Psittacidae	*Alisterus chloropterus*	Australasia	0	-0.1744	5.4555	2.55	0.7136	0.4561	13.68
Psittacidae	*Alisterus scapularis*	Australasia	0	-0.0479	6.0465	2.81	0.7556	0.2454	25.95
Psittacidae	*Amazona aestiva*	Neotropics	0	-0.4048	-	3.40	0.7544	0.4719	28.30
Psittacidae	*Amazona agilis*	Neotropics	0	-0.3162	-	3.70	0.8235	0.5557	23.51
Psittacidae	*Amazona albifrons*	Neotropics	1	-0.0061	5.6662	2.47	0.5985	0.0000	0.00
Psittacidae	*Amazona amazonica*	Neotropics	0	-0.0279	-	3.44	0.7685	0.2707	26.45
Psittacidae	*Amazona auropalliata*	Neotropics	0	-0.1060	-	3.93	0.8389	0.5692	37.70
Psittacidae	*Amazona autumnalis*	Neotropics	0	0.1581	-	3.41	0.7165	0.3963	19.33
Psittacidae	*Amazona barbadensis*	Neotropics	0	-0.0118	2.1871	3.44	0.7817	0.2497	35.87
Psittacidae	*Amazona brasiliensis*	Neotropics	1	-0.4303	1.1512	3.64	0.6961	0.1876	1.03
Psittacidae	*Amazona collaria*	Neotropics	1	-0.5189	1.8411	2.58	0.6302	0.2340	44.74
Psittacidae	*Amazona dufresniana*	Neotropics	1	-0.6466	4.5531	2.47	0.5985	0.0000	0.00
Psittacidae	*Amazona farinosa*	Neotropics	0	-0.1609	6.0467	3.91	0.7505	0.3995	19.82
Psittacidae	*Amazona festiva*	Neotropics	0	-0.1488	-	3.92	0.8382	0.5510	32.53
Psittacidae	*Amazona finschi*	Neotropics	0	-0.0598	-	3.99	0.7881	0.5643	9.19
Psittacidae	*Amazona kawalli*	Neotropics	1	-0.2549	8.4990	3.73	0.7291	0.1736	51.28
Psittacidae	*Amazona leucocephala*	Neotropics	0	-0.0641	-	4.27	0.7729	0.3808	7.61
Psittacidae	*Amazona ochrocephala*	Neotropics	0	0.0379	8.4910	3.64	0.7711	0.3920	79.70
Psittacidae	*Amazona oratrix*	Neotropics	0	-0.0258	-	4.01	0.8354	0.8499	27.73
Psittacidae	*Amazona pretrei*	Neotropics	1	-0.3714	1.0737	3.54	0.7413	0.4976	18.29
Psittacidae	*Amazona rhodocorytha*	Neotropics	1	-0.5276	3.4097	2.71	0.7151	0.6049	52.95
Psittacidae	*Amazona tucumana*	Neotropics	1	-0.1347	2.9951	3.06	0.6891	0.2353	64.49
Psittacidae	*Amazona ventralis*	Neotropics	1	12.3337	6.0287	3.72	0.7112	0.1613	85.39
Psittacidae	*Amazona vinacea*	Neotropics	1	-0.0297	4.1553	2.46	0.6859	0.2733	5.34
Psittacidae	*Amazona viridigenalis*	Neotropics	1	-0.3898	7.9533	2.94	0.7684	0.3730	29.07
Psittacidae	*Amazona vittata*	Neotropics	1	-0.0794	2.7864	3.61	0.7019	0.2639	67.01

Family	Species	Realm	Threat	Latitude	Elevation	Anomaly	Size	Shape	Orientation
Psittacidae	*Amazona xantholora*	Neotropics	1	0.0791	2.0233	2.41	0.5788	0.0000	0.00
Psittacidae	*Anodorhynchus hyacinthinus*	Neotropics	1	-0.0708	2.9556	4.02	0.8125	0.7057	82.31
Psittacidae	*Anodorhynchus leari*	Neotropics	1	0.0000	2.4487	3.56	0.6120	1.0000	0.00
Psittacidae	*Aprosmictus erythropterus*	Australasia	0	-0.2796	-	3.40	0.8094	0.4464	34.61
Psittacidae	*Aprosmictus jonquillaceus*	Australasia	0	-0.1643	7.9595	2.56	0.6523	0.1875	30.24
Psittacidae	*Aprosmictus jonquillaceus*	Australasia	0	-0.1643	7.9595	2.56	0.6523	-	-
Psittacidae	*Ara ambiguus*	Neotropics	1	0.0213	3.1920	3.11	0.7340	0.4357	53.72
Psittacidae	*Ara ararauna*	Neotropics	0	-0.0831	-	4.03	0.8402	0.6271	33.10
Psittacidae	*Ara chloropterus*	Neotropics	0	-0.1049	-	4.00	0.8411	0.7322	40.05
Psittacidae	*Ara macao*	Neotropics	1	2.9991	0.6545	4.16	0.6598	0.4910	42.97
Psittacidae	*Ara rubrogenys*	Neotropics	0	-0.0206	-	3.99	0.8377	0.5799	30.49
Psittacidae	*Ara severus*	Neotropics	1	-0.0669	11.1808	3.72	0.7830	0.2284	44.35
Psittacidae	*Aratinga acuticaudata*	Neotropics	0	-0.4008	-	3.53	0.8191	0.4155	56.13
Psittacidae	*Aratinga aurea*	Australasia	1	0.0001	3.2425	2.71	0.7185	0.5623	60.71
Psittacidae	*Aratinga auricapillus*	Neotropics	0	-0.2918	-	3.66	0.8521	0.5595	48.53
Psittacidae	*Aratinga cactorum*	Neotropics	0	-0.2547	-	3.54	0.8179	0.6894	86.41
Psittacidae	*Aratinga canicularis*	Neotropics	0	-0.1915	-	3.80	0.8314	0.7400	33.23
Psittacidae	*Aratinga chloroptera*	Neotropics	0	-0.2223	6.5452	3.46	0.7894	0.4469	41.19
Psittacidae	*Aratinga erythrogenys*	Neotropics	0	-0.0681	-	3.64	0.7866	0.5148	77.89
Psittacidae	*Aratinga euops*	Neotropics	0	-0.0297	-	3.55	0.7498	0.1292	26.49
Psittacidae	*Aratinga finschi*	Neotropics	1	-0.0452	7.7742	2.46	0.6876	0.3855	3.42
Psittacidae	*Aratinga holochlora*	Neotropics	0	-0.1484	2.3011	3.15	0.7144	0.2432	88.17
Psittacidae	*Aratinga jandaya*	Neotropics	1	-0.3204	0.9520	2.53	0.6974	0.1632	17.43
Psittacidae	*Aratinga leucophthalma*	Neotropics	0	-0.0847	-	3.22	0.7158	0.3481	54.90
Psittacidae	*Aratinga mitrata*	Neotropics	0	-0.0802	6.1291	3.61	0.7464	0.2152	39.81
Psittacidae	*Aratinga nana*	Neotropics	0	0.0984	-	3.69	0.7933	0.6694	3.46
Psittacidae	*Aratinga pertinax*	Neotropics	0	-0.2288	3.0066	3.78	0.8428	0.6966	46.25
Psittacidae	*Aratinga rubritorquis*	Neotropics	0	10.5958	10.6429	3.94	0.7493	0.3286	49.03
Psittacidae	*Aratinga solstitialis*	Neotropics	0	-0.0612	-	3.36	0.7639	0.3702	30.14
Psittacidae	*Aratinga wagleri*	Neotropics	0	-0.0400	-	3.95	0.7972	0.4480	10.40
Psittacidae	*Aratinga weddellii*	Neotropics	0	0.1841	9.8539	3.52	0.7205	0.4907	14.80
Psittacidae	*Barnardius zonarius*	Neotropics	0	-0.1114	5.6348	3.02	0.6740	0.2651	22.73
Psittacidae	*Bolbopsittacus lunulatus*	IndoMalay	0	-0.1896	9.6824	3.23	0.8070	0.5204	4.16
Psittacidae	*Bolborhynchus ferrugineifrons*	IndoMalay	0	0.0037	-	2.84	0.7887	0.5025	8.43
Psittacidae	*Bolborhynchus lineola*	Neotropics	0	-0.1270	8.7088	3.43	0.7403	0.2788	52.84
Psittacidae	*Bolborhynchus orbygnesius*	Neotropics	0	0.5457	9.0488	2.99	0.6935	0.1670	64.28
Psittacidae	*Brotogeris chiriri*	Australasia	0	-0.3211	-	2.56	0.6120	0.2302	28.68
Psittacidae	*Brotogeris chrysoptera*	Afrotropics	0	-0.7914	-	2.88	0.7060	0.2305	0.73
Psittacidae	*Brotogeris cyanoptera*	Nearctic	0	-1.1739	0.0000	5.75	0.7980	0.1738	6.20
Psittacidae	*Brotogeris jugularis*	Nearctic	0	-0.8317	6.2340	4.34	0.8524	0.3384	9.75
Psittacidae	*Brotogeris pyrrhoptera*	Nearctic	0	-0.8311	0.0000	4.37	0.8513	0.5340	15.10
Psittacidae	*Brotogeris sanctithomae*	Palearctic	0	-0.9741	-	6.13	0.7340	0.1853	3.69
Psittacidae	*Brotogeris tirica*	Palearctic	1	-0.7499	-	6.97	0.7833	0.1811	10.82
Psittacidae	*Cacatua alba*	Neotropics	0	1.9121	4.2872	3.32	0.7144	0.1495	60.73
Psittacidae	*Cacatua ducorpsii*	Neotropics	0	0.0646	9.5605	3.60	0.7637	0.3514	75.87
Psittacidae	*Cacatua galerita*	Neotropics	1	5.6397	3.9939	3.25	0.6935	0.1084	68.41
Psittacidae	*Cacatua goffiniana*	Afrotropics	0	-0.4527	5.7895	3.52	0.8854	0.2233	8.74
Psittacidae	*Cacatua haematuropygia*	Nearctic	0	-0.5252	-	3.98	0.8389	0.7080	5.47
Psittacidae	*Cacatua leadbeateri*	Afrotropics	0	0.0623	18.8172	3.28	0.8043	0.4756	10.29
Psittacidae	*Cacatua moluccensis*	Afrotropics	0	0.0204	11.0424	3.38	0.7822	0.1557	83.91
Psittacidae	*Cacatua ophthalmica*	Afrotropics	0	-0.1816	9.9861	3.53	0.8223	0.9036	81.65
Psittacidae	*Cacatua pastinator*	Afrotropics	0	0.4505	7.4531	3.14	0.7584	0.3651	3.31
Psittacidae	*Cacatua roseicapilla*	Afrotropics	0	-0.0545	2.6762	3.25	0.8136	0.3177	15.00
Psittacidae	*Cacatua sanguinea*	Afrotropics	0	0.0151	10.6820	3.33	0.7865	0.3300	12.08
Psittacidae	*Cacatua sulphurea*	Australasia	1	0.0151	2.8084	2.48	0.6302	0.2423	87.23
Psittacidae	*Cacatua tenuirostris*	Australasia	0	-0.0385	5.2956	2.48	0.6120	0.0400	36.10
Psittacidae	*Callocephalon fimbriatum*	Australasia	0	-0.0742	-	2.78	0.7656	0.2174	23.14
Psittacidae	*Calyptorhynchus banksii*	Palearctic	0	-0.3848	0.0000	3.22	0.7958	0.6561	2.27
Psittacidae	*Calyptorhynchus baudinii*	Australasia	0	-0.4058	-	2.90	0.7991	0.6401	74.48
Psittacidae	*Calyptorhynchus funereus*	IndoMalay	0	0.0123	2.2688	2.86	0.7637	0.7570	34.98
Psittacidae	*Calyptorhynchus lathami*	IndoMalay	0	-0.0047	-	2.84	0.7828	0.4720	7.44
Psittacidae	*Calyptorhynchus latirostris*	Nearctic	0	-0.4576	7.7024	4.08	0.7578	0.2679	50.11
Psittacidae	*Chalcopsitta atra*	Neotropics	0	-0.3859	10.7290	3.88	0.7087	0.2802	13.03
Psittacidae	*Chalcopsitta cardinalis*	Neotropics	0	0.0819	7.4611	3.62	0.7580	0.3727	70.27
Psittacidae	*Chalcopsitta duivenbodei*	Afrotropics	0	22.1469	-	3.54	0.7172	0.4354	29.48
Psittacidae	*Chalcopsitta sintillata*	Afrotropics	0	-0.8448	-	2.94	0.7008	0.4606	6.43
Psittacidae	*Charmosyna amabilis*	Afrotropics	0	-0.1052	-	3.39	0.8311	0.5065	38.11
Psittacidae	*Charmosyna diadema*	Nearctic	0	-0.7365	-	4.40	0.7947	0.3458	14.92
Psittacidae	*Charmosyna josefinae*	Neotropics	0	-0.5149	-	1.75	0.7419	0.6228	44.21
Psittacidae	*Charmosyna margarethae*	Palearctic	0	-0.4679	-	4.54	0.8249	0.2405	5.29
Psittacidae	*Charmosyna meeki*	Nearctic	0	0.4533	13.3836	4.40	0.7779	0.4027	68.85
Psittacidae	*Charmosyna multistriata*	Australasia	1	-0.4953	2.3921	2.48	0.6763	0.5757	56.95
Psittacidae	*Charmosyna palmarum*	Afrotropics	0	-0.1712	-	3.80	0.7539	0.4340	60.90
Psittacidae	*Charmosyna papou*	Afrotropics	0	-0.2434	-	3.60	0.8596	-	-

Family	Species	Realm	Threat	Latitude	Elevation	Anomaly	Size	Shape	Orientation
Psittacidae	*Charmosyna papou*	Afrotropics	0	-0.2434	-	3.60	0.8596	0.7767	52.69
Psittacidae	*Charmosyna placentis*	Palearctic	0	-1.0201	-	3.92	0.8068	0.4089	19.47
Psittacidae	*Charmosyna pulchella*	Australasia	0	-0.5000	0.0000	3.27	0.8283	0.5715	9.91
Psittacidae	*Charmosyna rubrigularis*	Nearctic	0	-0.7366	-	4.82	0.8274	0.1715	2.45
Psittacidae	*Charmosyna rubronotata*	Afrotropics	1	-0.2961	0.1689	3.10	0.6891	0.2750	83.58
Psittacidae	*Charmosyna toxopei*	Afrotropics	0	-0.0995	-	3.63	0.8449	0.6037	62.38
Psittacidae	*Charmosyna wilhelminae*	Palearctic	0	-0.7788	-	4.33	0.8070	0.2388	3.78
Psittacidae	*Coracopsis nigra*	IndoMalay	0	-0.0974	-	3.42	0.7854	0.6808	61.44
Psittacidae	*Coracopsis vasa*	Australasia	0	-0.0873	-	2.77	0.7691	0.3118	23.97
Psittacidae	*Cyanoliseus patagonus*	Neotropics	0	-0.0029	-	3.94	0.8395	0.5962	28.87
Psittacidae	*Cyanopsitta spixii*	Neotropics	0	0.1898	9.3297	3.55	0.7211	0.4052	10.64
Psittacidae	*Cyanoramphus auriceps*	Neotropics	0	-0.5753	-	3.56	0.7019	0.2004	33.16
Psittacidae	*Cyanoramphus malherbi*	Neotropics	0	0.0831	-	4.01	0.8171	0.6319	66.73
Psittacidae	*Cyanoramphus novaezelandiae*	Neotropics	0	-0.0756	-	3.58	0.7945	0.3561	47.04
Psittacidae	*Cyanoramphus saisseti*	Neotropics	0	-0.2362	-	3.11	0.7256	0.5329	32.56
Psittacidae	*Cyclopsitta diophthalma*	Neotropics	0	-0.6988	-	2.82	0.7822	0.4520	88.39
Psittacidae	*Cyclopsitta gulielmitertii*	Neotropics	0	-0.4955	1.9958	2.57	0.7704	0.4398	32.08
Psittacidae	*Deroptyus accipitrinus*	Nearctic	0	-0.7319	8.9405	4.26	0.8574	0.4178	16.24
Psittacidae	*Diopsittaca nobilis*	Neotropics	0	0.0118	6.4420	3.69	0.7628	0.4061	88.15
Psittacidae	*Eclectus roratus*	Australasia	0	-0.2187	-	2.56	0.6370	0.3056	34.87
Psittacidae	*Enicognathus ferrugineus*	Neotropics	0	-0.5883	-	2.85	0.7660	0.6229	17.59
Psittacidae	*Enicognathus leptorhynchus*	Neotropics	0	-2.7048	3.4897	3.54	0.7266	0.2400	69.28
Psittacidae	*Eos bornea*	Neotropics	0	-0.2584	-	3.75	0.8465	0.7570	85.80
Psittacidae	*Eos reticulata*	Afrotropics	0	0.2074	-	3.39	0.7748	0.3745	57.30
Psittacidae	*Eos semilarvata*	Neotropics	0	-0.7162	-	2.25	0.7651	0.2702	88.18
Psittacidae	*Eos squamata*	Neotropics	0	-0.6491	-	2.39	0.7179	0.1863	80.46
Psittacidae	*Eunymphicus cornutus*	Neotropics	0	-0.6586	7.8620	2.70	0.7942	0.3316	75.89
Psittacidae	*Forpus coelestis*	IndoMalay	1	0.0000	5.3105	4.53	0.6370	0.5446	11.26
Psittacidae	*Forpus conspicillatus*	Palearctic	0	-0.2557	-	4.12	0.7881	0.1504	9.24
Psittacidae	*Forpus cyanopygius*	Australasia	0	-0.1325	4.5501	2.56	0.6370	0.1401	27.99
Psittacidae	*Forpus modestus*	Palearctic	0	-0.2670	-	3.67	0.8018	0.4135	7.21
Psittacidae	*Forpus passerinus*	IndoMalay	0	-0.0834	-	3.09	0.8190	0.4852	50.24
Psittacidae	*Forpus xanthops*	Palearctic	0	-0.8150	-	4.11	0.8107	0.6505	49.18
Psittacidae	*Forpus xanthopterygius*	Neotropics	0	-0.3411	4.4191	2.90	0.7744	0.2297	43.65
Psittacidae	*Geoffroyus geoffroyi*	IndoMalay	0	-0.6996	9.8866	2.61	0.6478	0.3444	72.46
Psittacidae	*Geoffroyus heteroclitus*	IndoMalay	1	-0.1156	1.2530	2.73	0.5985	0.5771	46.13
Psittacidae	*Geoffroyus simplex*	IndoMalay	0	0.4476	3.3594	3.33	0.7069	0.5991	2.81
Psittacidae	*Glossopsitta concinna*	IndoMalay	0	-0.0786	4.8144	3.39	0.8215	0.4777	12.64
Psittacidae	*Glossopsitta porphyrocephala*	Palearctic	0	-0.6007	-	3.71	0.8335	0.5068	45.95
Psittacidae	*Glossopsitta pusilla*	Palearctic	0	-0.9491	-	4.91	0.8199	0.2114	4.86
Psittacidae	*Graydidascalus brachyurus*	Neotropics	1	-0.0107	3.6662	3.95	0.6891	0.5241	11.68
Psittacidae	*Guaruba guarouba*	Neotropics	1	-0.0881	5.6927	3.15	0.7050	0.2119	64.10
Psittacidae	*Hapalopsittaca amazonina*	Neotropics	0	-0.1826	2.7733	3.80	0.8333	0.3071	40.29
Psittacidae	*Hapalopsittaca fuertesi*	Australasia	1	0.1179	2.2259	2.48	0.6221	0.5774	71.57
Psittacidae	*Hapalopsittaca melanotis*	Neotropics	0	-0.0307	-	3.93	0.7818	0.6965	13.73
Psittacidae	*Hapalopsittaca pyrrhops*	Australasia	0	-0.5967	0.0000	2.17	0.6974	0.2695	41.87
Psittacidae	*Lathamus discolor*	Afrotropics	0	0.0061	5.3628	3.17	0.7674	0.4182	50.27
Psittacidae	*Leptosittaca branickii*	Neotropics	0	-0.3346	8.0462	3.84	0.7872	0.3230	74.50
Psittacidae	*Loriculus amabilis*	IndoMalay	0	-0.0736	3.6059	3.00	0.8275	0.2400	5.38
Psittacidae	*Loriculus aurantiifrons*	Australasia	0	-0.0097	-	2.49	0.6428	0.2516	25.26
Psittacidae	*Loriculus beryllinus*	Australasia	0	-0.1066	-	2.64	0.7378	0.8456	46.26
Psittacidae	*Loriculus exilis*	Australasia	0	-0.5538	6.7584	2.89	0.7087	0.1350	15.64
Psittacidae	*Loriculus flosculus*	Australasia	0	-0.4730	7.4364	2.63	0.6478	0.3846	50.26
Psittacidae	*Loriculus galgulus*	Afrotropics	0	-0.0727	6.6742	3.18	0.7614	0.3874	70.15
Psittacidae	*Loriculus philippensis*	Australasia	0	-0.0074	0.6740	2.81	0.7019	0.3630	10.89
Psittacidae	*Loriculus pusillus*	Afrotropics	0	-0.1531	-	3.46	0.7994	0.5106	73.75
Psittacidae	*Loriculus sclateri*	Australasia	0	-0.0997	-	2.69	0.7261	0.6554	75.84
Psittacidae	*Loriculus stigmatus*	IndoMalay	0	-0.2370	-	3.19	0.8399	0.4380	18.37
Psittacidae	*Loriculus tener*	Australasia	0	0.0445	-	2.59	0.6891	0.1686	0.56
Psittacidae	*Loriculus vernalis*	Australasia	0	0.0396	-	2.80	0.7382	0.2974	14.53
Psittacidae	*Lorius albidinucha*	IndoMalay	0	-0.2804	-	3.15	0.8307	0.6894	15.49
Psittacidae	*Lorius chlorocercus*	Australasia	0	-0.1153	-	2.85	0.6631	0.7842	5.34
Psittacidae	*Lorius domicella*	Australasia	0	-0.9130	-	2.85	0.6763	0.3425	15.40
Psittacidae	*Lorius garrulus*	Australasia	0	-0.0872	-	2.79	0.7711	0.2969	24.12
Psittacidae	*Lorius hypoinochrous*	Australasia	1	0.2734	0.6362	2.50	0.6370	0.3679	33.40
Psittacidae	*Lorius lory*	Afrotropics	0	-0.0986	-	3.47	0.8556	0.5916	32.97
Psittacidae	*Melopsittacus undulatus*	Australasia	0	-0.0755	13.1819	2.81	0.7413	0.1491	24.21
Psittacidae	*Micropsitta bruijnii*	IndoMalay	0	-0.0913	-	3.09	0.8128	0.6644	31.64
Psittacidae	*Micropsitta finschii*	Afrotropics	0	0.1540	-	3.26	0.7879	0.3938	36.99
Psittacidae	*Micropsitta keiensis*	Afrotropics	0	0.1803	3.8866	3.16	0.7546	0.1850	6.86
Psittacidae	*Micropsitta pusio*	Afrotropics	0	0.0752	6.0819	3.28	0.7980	0.4634	2.42
Psittacidae	*Myiopsitta monachus*	IndoMalay	1	0.0617	0.4823	2.95	0.7261	0.2648	88.18
Psittacidae	*Nandayus nenday*	Afrotropics	0	-0.1286	10.8729	3.51	0.8211	0.7029	68.55
Psittacidae	*Nannopsittaca dachilleae*	Afrotropics	0	-0.4808	-	4.04	0.8032	0.7399	47.79

Family	Species	Realm	Threat	Latitude	Elevation	Anomaly	Size	Shape	Orientation
Psittacidae	*Nannopsittaca panychlora*	Afrotropics	0	0.0619	5.5017	3.58	0.7517	0.6253	85.90
Psittacidae	*Neophema chrysogaster*	IndoMalay	0	-0.1526	-	2.92	0.8283	0.3703	34.42
Psittacidae	*Neophema chrysostoma*	Afrotropics	0	-0.0710	7.6848	3.42	0.7758	0.6915	38.01
Psittacidae	*Neophema elegans*	IndoMalay	0	-0.0312	-	3.13	0.7510	-	-
Psittacidae	*Neophema elegans*	IndoMalay	0	-0.0312	-	3.13	0.7510	0.3451	59.41
Psittacidae	*Neophema pulchella*	Afrotropics	0	-0.1697	-	3.70	0.7956	0.7119	7.06
Psittacidae	*Neophema splendida*	Afrotropics	0	-0.0525	-	3.86	0.8175	0.3758	58.31
Psittacidae	*Neopsephotus bourkii*	Afrotropics	0	0.0822	-	3.21	0.7933	0.2066	14.01
Psittacidae	*Neopsittacus musschenbroekii*	Afrotropics	0	4.4713	7.1815	3.23	0.6876	0.3070	60.54
Psittacidae	*Neopsittacus pullicauda*	Afrotropics	0	-0.1127	-	3.24	0.7654	0.4809	57.61
Psittacidae	*Nestor meridionalis*	Afrotropics	0	0.0617	-	3.30	0.8156	0.3720	14.39
Psittacidae	*Nestor notabilis*	Afrotropics	0	0.1539	14.2371	3.48	0.7631	0.3403	77.06
Psittacidae	*Northiella haematogaster*	Neotropics	0	-4.3519	7.7988	3.97	0.7281	0.1924	42.02
Psittacidae	*Nymphicus hollandicus*	Afrotropics	0	-0.0812	2.6749	3.16	0.7569	0.3878	70.07
Psittacidae	*Ognorhynchus icterotis*	Neotropics	0	0.1671	1.9000	2.94	0.7019	0.1752	73.57
Psittacidae	*Oreopsittacus arfaki*	IndoMalay	0	-0.0427	0.0000	3.21	0.7385	0.1645	69.23
Psittacidae	*Orthopsittaca manilata*	Neotropics	0	-0.0312	-	4.02	0.8389	0.7415	9.88
Psittacidae	*Pezoporus occidentalis*	Neotropics	1	-0.4000	5.0726	3.20	0.6662	0.3401	70.04
Psittacidae	*Pezoporus wallicus*	Neotropics	0	-0.1343	7.9913	3.81	0.7240	0.5264	22.94
Psittacidae	*Phigys solitarius*	Neotropics	0	-0.6280	3.1715	2.56	0.7526	0.2732	42.57
Psittacidae	*Pionites leucogaster*	Palearctic	0	-0.8167	1.1363	4.58	0.8296	0.3126	0.37
Psittacidae	*Pionites melanocephalus*	Palearctic	0	-0.6220	11.9769	3.82	0.7876	0.4476	1.19
Psittacidae	*Pionopsitta pileata*	IndoMalay	0	-0.0047	5.9667	3.41	0.8039	0.5289	23.87
Psittacidae	*Pionus chalcopterus*	Palearctic	0	-0.4239	3.5470	3.34	0.7987	0.8979	3.58
Psittacidae	*Pionus fuscus*	Afrotropics	0	-0.2787	10.0072	3.34	0.7696	0.2134	68.98
Psittacidae	*Pionus maximiliani*	Australasia	0	-0.0992	-	2.68	0.7211	0.5222	74.32
Psittacidae	*Pionus menstruus*	Palearctic	0	-0.5675	-	4.52	0.8142	0.1764	5.68
Psittacidae	*Pionus senilis*	Palearctic	0	-0.5385	-	4.47	0.8412	0.2514	2.07
Psittacidae	*Pionus sordidus*	Palearctic	0	-1.0132	1.2451	4.18	0.7863	0.1483	1.96
Psittacidae	*Pionus tumultuosus*	Palearctic	0	-0.3011	9.4757	3.53	0.8088	0.5630	15.49
Psittacidae	*Platycercus adscitus*	Neotropics	0	0.0660	5.9311	3.61	0.7597	0.4386	80.38
Psittacidae	*Platycercus caledonicus*	Neotropics	0	0.0351	-	4.15	0.8099	0.7779	11.74
Psittacidae	*Platycercus caledonicus*	Neotropics	0	0.0351	-	4.15	0.8099	-	-
Psittacidae	*Platycercus elegans*	Neotropics	0	-0.0914	-	3.94	0.8214	0.8418	87.74
Psittacidae	*Platycercus eximius*	Neotropics	1	-0.2710	3.5726	3.14	0.7852	0.3638	34.24
Psittacidae	*Platycercus venustus*	Nearctic	0	-0.6072	-	4.25	0.8001	0.6658	14.23
Psittacidae	*Poicephalus crassus*	IndoMalay	0	0.0371	-	3.00	0.7801	0.4562	81.74
Psittacidae	*Poicephalus cryptoxanthus*	Afrotropics	0	0.0350	-	3.31	0.7569	0.3845	10.47
Psittacidae	*Poicephalus flavifrons*	Afrotropics	0	-0.1197	-	3.62	0.8247	0.3864	57.87
Psittacidae	*Poicephalus gulielmi*	Afrotropics	0	0.1163	-	3.29	0.7718	0.5703	74.93
Psittacidae	*Poicephalus meyeri*	Afrotropics	0	-16.2406	-	3.60	0.7112	0.4535	44.24
Psittacidae	*Poicephalus robustus*	Afrotropics	0	0.0935	-	3.52	0.8197	0.2032	7.76
Psittacidae	*Poicephalus rueppellii*	IndoMalay	0	-0.1312	-	3.37	0.8215	0.4481	23.29
Psittacidae	*Poicephalus rufiventris*	IndoMalay	1	-0.0746	-	3.51	0.7136	0.1831	13.82
Psittacidae	*Poicephalus senegalus*	Afrotropics	0	-0.1021	-	3.47	0.8040	0.3022	19.13
Psittacidae	*Polytelis alexandrae*	Neotropics	0	-0.7486	3.8162	1.81	0.7205	0.4341	59.11
Psittacidae	*Polytelis anthopeplus*	Afrotropics	0	0.0836	-	3.46	0.8339	0.3070	4.81
Psittacidae	*Polytelis swainsonii*	IndoMalay	0	-0.2023	15.0176	3.69	0.7759	0.2941	5.83
Psittacidae	*Primolius auricollis*	Afrotropics	0	-0.0566	8.9538	3.68	0.8337	0.4955	81.00
Psittacidae	*Primolius couloni*	Afrotropics	0	-0.1345	16.7924	3.60	0.8167	0.2932	33.59
Psittacidae	*Primolius maracana*	Afrotropics	0	-0.1686	-	3.98	0.7674	0.3916	67.35
Psittacidae	*Prioniturus discurus*	IndoMalay	0	-0.1402	5.9346	3.15	0.7922	0.5384	34.30
Psittacidae	*Prioniturus flavicans*	IndoMalay	0	0.0837	5.0355	2.77	0.7228	0.1061	53.73
Psittacidae	*Prioniturus luconensis*	IndoMalay	0	0.0940	4.0689	2.90	0.6824	0.5140	32.62
Psittacidae	*Prioniturus mada*	IndoMalay	1	0.2670	5.6370	2.84	0.6740	0.3637	78.76
Psittacidae	*Prioniturus montanus*	IndoMalay	1	-0.4680	0.0000	2.53	0.6302	0.5219	0.00
Psittacidae	*Prioniturus platenae*	IndoMalay	1	0.1694	0.5472	2.79	0.7120	0.4130	60.35
Psittacidae	*Prioniturus platurus*	IndoMalay	1	0.2091	1.6355	2.52	0.5985	0.0024	45.41
Psittacidae	*Probosciger aterrimus*	IndoMalay	0	0.1784	5.7257	3.37	0.7510	0.5894	81.22
Psittacidae	*Prosopeia personata*	Neotropics	0	2.9638	8.9880	3.77	0.7185	0.1478	84.04
Psittacidae	*Prosopeia tabuensis*	Neotropics	0	-0.2956	-	3.38	0.7211	0.1233	65.57
Psittacidae	*Psephotus chrysopterygius*	Neotropics	1	-7.6125	3.6530	4.16	0.7537	0.3981	32.81
Psittacidae	*Psephotus dissimilis*	Neotropics	0	-0.2634	4.3561	3.74	0.8207	0.8644	40.33
Psittacidae	*Psephotus haematonotus*	IndoMalay	0	-0.1218	-	3.18	0.8072	0.8259	83.32
Psittacidae	*Psephotus haematonotus*	IndoMalay	0	-0.1218	-	3.18	0.8072	-	-
Psittacidae	*Psephotus varius*	Afrotropics	0	0.0293	8.6412	3.24	0.7896	0.4040	0.47
Psittacidae	*Pseudeos fuscata*	IndoMalay	0	-0.1018	-	3.14	0.8279	0.6208	39.72
Psittacidae	*Psilopsiagon aurifrons*	Afrotropics	1	12.6360	6.3306	3.42	0.6961	0.2801	87.60
Psittacidae	*Psilopsiagon aymara*	Afrotropics	0	0.0636	6.0786	3.25	0.8128	0.2245	14.31
Psittacidae	*Psittacella brehmii*	Afrotropics	0	40.1708	5.8112	3.17	0.7008	0.4933	36.12
Psittacidae	*Psittacella madaraszi*	Afrotropics	0	-0.1116	-	3.71	0.8252	0.7141	24.32
Psittacidae	*Psittacella modesta*	Afrotropics	0	-0.0840	-	3.26	0.7786	0.2274	77.70
Psittacidae	*Psittacella picta*	Neotropics	0	0.1647	11.7501	2.47	0.6562	0.3610	16.86
Psittacidae	*Psittacula alexandri*	Neotropics	0	-0.3367	-	2.53	0.6974	0.1632	17.43

Family	Species	Realm	Threat	Latitude	Elevation	Anomaly	Size	Shape	Orientation
Psittacidae	*Psittacula calthropae*	Australasia	0	-0.0828	2.6793	2.78	0.7732	0.4454	30.62
Psittacidae	*Psittacula columboides*	Neotropics	1	-0.3565	0.4772	2.34	0.7792	0.1051	0.52
Psittacidae	*Psittacula cyanocephala*	Neotropics	0	-0.3653	0.9024	2.27	0.7687	0.1134	0.11
Psittacidae	*Psittacula derbiana*	Neotropics	1	-0.5384	0.7015	2.37	0.7050	0.1007	0.36
Psittacidae	*Psittacula eupatria*	Australasia	1	-0.2694	1.6911	2.52	0.6891	0.0366	8.14
Psittacidae	*Psittacula finschii*	Neotropics	1	-0.5382	-	2.14	0.7455	0.0400	1.31
Psittacidae	*Psittacula himalayana*	Neotropics	0	-0.1841	5.4930	4.06	0.7648	0.4025	39.52
Psittacidae	*Psittacula krameri*	Neotropics	0	-0.0465	-	3.71	0.7822	0.1682	28.13
Psittacidae	*Psittacula longicauda*	Neotropics	1	-0.2872	4.6617	3.19	0.7937	0.2939	44.32
Psittacidae	*Psittacula roseata*	Neotropics	1	0.0766	9.9929	3.18	0.7008	0.2583	57.38
Psittacidae	*Psittaculirostris desmarestii*	Afrotropics	0	-0.0044	8.6010	3.27	0.8154	0.3258	13.24
Psittacidae	*Psittaculirostris edwardsii*	Afrotropics	0	-0.1086	9.9313	3.60	0.8199	0.6683	79.85
Psittacidae	*Psittaculirostris salvadorii*	Afrotropics	0	-0.0965	10.5638	3.67	0.8229	0.6062	45.30
Psittacidae	*Psittacus erithacus*	Neotropics	0	-0.2087	5.3727	3.72	0.8570	0.5290	43.00
Psittacidae	*Psitteuteles goldiei*	Neotropics	0	-0.3061	-	2.53	0.6974	0.1632	17.43
Psittacidae	*Psitteuteles iris*	Neotropics	0	0.1573	-	2.46	0.6948	0.1958	1.91
Psittacidae	*Psitteuteles versicolor*	Neotropics	0	-0.5989	8.1329	2.93	0.7897	0.2640	84.04
Psittacidae	*Psittrichas fulgidus*	Nearctic	0	-0.4363	9.6928	3.95	0.7396	0.1521	56.62
Psittacidae	*Purpureicephalus spurius*	Australasia	0	-0.2898	8.3136	2.84	0.7505	0.2084	23.13
Psittacidae	*Purpureicephalus spurius*	Australasia	0	-0.2898	8.3136	2.84	0.7505	-	-
Psittacidae	*Pyrilia aurantiocephala*	Neotropics	0	0.1141	-	4.17	0.8009	0.5705	8.24
Psittacidae	*Pyrilia barrabandi*	Neotropics	0	-0.0668	1.7832	4.08	0.8032	0.4783	31.98
Psittacidae	*Pyrilia caica*	Neotropics	0	-0.1797	-	4.02	0.8288	0.3929	38.52
Psittacidae	*Pyrilia haematotis*	Neotropics	0	0.0278	-	3.01	0.6662	0.1454	29.30
Psittacidae	*Pyrilia pulchra*	Neotropics	0	0.0847	-	4.04	0.8273	0.4244	3.24
Psittacidae	*Pyrilia pyrilia*	Neotropics	0	0.0934	-	3.82	0.7904	0.4916	27.57
Psittacidae	*Pyrilia vulturina*	Neotropics	0	-0.2107	3.2398	3.43	0.7821	0.2349	25.23
Psittacidae	*Pyrrhura albipectus*	Australasia	0	0.3255	3.9330	2.87	0.7078	0.1068	14.91
Psittacidae	*Pyrrhura calliptera*	Australasia	0	-0.1050	-	2.78	0.7711	0.3093	23.60
Psittacidae	*Pyrrhura cruentata*	Australasia	1	0.9838	1.8282	2.60	0.6120	0.4082	18.43
Psittacidae	*Pyrrhura egregia*	Australasia	0	-0.0230	11.0760	2.72	0.7069	0.4752	54.39
Psittacidae	*Pyrrhura griseipectus*	Australasia	0	-0.2111	-	2.57	0.6370	0.3880	82.91
Psittacidae	*Pyrrhura hoematotis*	Australasia	0	-0.0777	3.1109	2.48	0.6221	0.5774	71.57
Psittacidae	*Pyrrhura hoffmanni*	Australasia	0	-0.3736	4.0444	2.50	0.6716	0.2680	24.42
Psittacidae	*Pyrrhura lepida*	Australasia	0	-0.0998	-	2.78	0.7711	0.3093	23.60
Psittacidae	*Pyrrhura leucotis*	IndoMalay	0	0.0010	-	2.83	0.7895	0.5066	5.44
Psittacidae	*Pyrrhura melanura*	IndoMalay	0	-0.2588	-	2.63	0.7359	0.2870	72.97
Psittacidae	*Pyrrhura orcesi*	Australasia	0	-0.2471	-	2.90	0.7844	0.2480	61.10
Psittacidae	*Pyrrhura perlata*	IndoMalay	1	-0.0627	5.4465	2.66	0.6997	0.4736	75.00
Psittacidae	*Pyrrhura pfrimeri*	IndoMalay	0	-0.0318	4.4762	2.67	0.7544	0.4683	41.77
Psittacidae	*Pyrrhura picta*	IndoMalay	0	0.0000	2.5579	2.67	0.6891	0.5490	73.78
Psittacidae	*Pyrrhura rhodocephala*	Australasia	0	0.0000	2.4031	2.48	0.6302	0.2423	87.23
Psittacidae	*Pyrrhura rupicola*	Australasia	0	-0.1263	-	2.79	0.7464	0.0998	24.42
Psittacidae	*Pyrrhura viridicata*	IndoMalay	0	-0.0044	-	2.64	0.7323	0.2310	67.52
Psittacidae	*Rhynchopsitta pachyrhyncha*	IndoMalay	0	-0.0843	-	2.87	0.8044	0.5778	44.68
Psittacidae	*Rhynchopsitta terrisi*	Australasia	0	-0.3224	10.3076	2.84	0.7367	0.1916	23.45
Psittacidae	*Strigops habroptila*	IndoMalay	0	-0.1969	8.2021	3.43	0.7374	0.3636	41.04
Psittacidae	*Tanygnathus gramineus*	Palearctic	0	-1.0295	9.3042	4.31	0.8004	0.4904	23.23
Psittacidae	*Tanygnathus lucionensis*	Palearctic	0	-0.6834	5.0996	4.36	0.8475	0.2885	8.18
Psittacidae	*Tanygnathus megalorynchos*	Afrotropics	0	-0.0830	-	3.33	0.7780	0.2445	59.44
Psittacidae	*Tanygnathus sumatranus*	Palearctic	0	-0.5729	5.5361	4.54	0.8470	0.2684	4.26
Psittacidae	*Touit batavicus*	Neotropics	0	0.1395	-	4.08	0.8269	0.6917	4.88
Psittacidae	*Touit costaricensis*	Neotropics	0	-0.0364	-	4.03	0.8335	0.6420	7.19
Psittacidae	*Touit dilectissimus*	Neotropics	0	-0.0723	2.1780	3.07	0.7348	0.2094	63.10
Psittacidae	*Touit huetii*	Neotropics	0	-10.1130	6.1956	4.03	0.7060	0.2590	40.42
Psittacidae	*Touit melanonotus*	Neotropics	0	0.1726	3.3501	3.29	0.7698	0.6379	44.25
Psittacidae	*Touit purpuratus*	Neotropics	0	-0.0842	3.0429	3.00	0.6740	0.1113	30.84
Psittacidae	*Touit stictopterus*	Neotropics	0	-0.3228	8.4997	3.36	0.8226	0.6712	25.84
Psittacidae	*Touit surdus*	Neotropics	0	0.1426	-	3.96	0.7488	0.4594	7.25
Psittacidae	*Trichoglossus chlorolepidotus*	Neotropics	0	0.0332	10.7578	3.69	0.7563	0.3792	69.09
Psittacidae	*Trichoglossus euteles*	Neotropics	0	0.1526	9.5731	3.04	0.7078	0.1026	69.94
Psittacidae	*Trichoglossus flavoviridis*	Neotropics	0	-1.5088	4.7816	3.72	0.7399	0.3665	80.21
Psittacidae	*Trichoglossus haematodus*	Neotropics	0	-0.1674	11.4227	3.04	0.6784	0.1941	29.99
Psittacidae	*Trichoglossus johnstoniae*	Neotropics	0	-3.1600	4.6495	3.98	0.7335	0.1790	41.78
Psittacidae	*Trichoglossus ornatus*	Neotropics	0	-0.7341	6.5014	3.29	0.7128	-	-
Psittacidae	*Trichoglossus ornatus*	Neotropics	0	-0.7341	6.5014	3.29	0.7128	0.1559	46.48
Psittacidae	*Triclaria malachitacea*	Neotropics	0	-0.0046	8.3979	3.04	0.6221	0.3473	45.31
Psophiidae	*Psophia crepitans*	Nearctic	0	-0.7158	-	4.20	0.8341	0.6368	2.80
Psophiidae	*Psophia leucoptera*	Neotropics	0	-0.2375	4.9321	3.53	0.8416	0.6482	66.24
Psophiidae	*Psophia viridis*	Afrotropics	0	-0.8631	-	2.94	0.7172	0.2871	3.08
Pteroclididae	*Pterocles alchata*	Palearctic	0	-1.2248	16.1743	4.30	0.8506	0.2240	4.47
Pteroclididae	*Pterocles bicinctus*	Afrotropics	0	-0.1985	7.2022	3.49	0.6784	0.3755	71.22
Pteroclididae	*Pterocles burchelli*	Palearctic	0	-1.0326	15.0825	4.24	0.8207	0.4388	8.13
Pteroclididae	*Pterocles coronatus*	Palearctic	0	1.8174	8.8195	4.41	0.7861	0.3527	11.64

Family	Species	Realm	Threat	Latitude	Elevation	Anomaly	Size	Shape	Orientation
Pteroclididae	*Pterocles decoratus*	Palearctic	0	0.0112	12.1116	4.05	0.7749	0.5095	18.17
Pteroclididae	*Pterocles exustus*	Palearctic	0	-8.7093	-	4.29	0.7738	0.4839	26.77
Pteroclididae	*Pterocles gutturalis*	Palearctic	0	-0.5440	-	4.27	0.8391	0.3829	25.23
Pteroclididae	*Pterocles lichtensteinii*	Palearctic	0	-1.1338	13.5351	4.03	0.7539	0.1761	8.99
Pteroclididae	*Pterocles namaqua*	Palearctic	0	-0.6934	14.4928	4.33	0.7955	0.3925	5.08
Pteroclididae	*Pterocles orientalis*	Palearctic	0	-0.9546	10.1160	3.53	0.7422	0.2802	58.63
Pteroclididae	*Pterocles personatus*	Palearctic	0	-0.6234	-	4.01	0.7994	0.3681	0.93
Pteroclididae	*Pterocles quadricinctus*	Afrotropics	0	3.9808	-	3.42	0.7904	0.6777	61.74
Pteroclididae	*Pterocles senegallus*	Afrotropics	0	0.9896	11.2621	3.23	0.6763	0.5383	49.86
Pteroclididae	*Syrrhaptes paradoxus*	Neotropics	0	-0.6096	0.1598	2.25	0.7256	0.1387	4.40
Pteroclididae	*Syrrhaptes tibetanus*	Neotropics	0	-0.2770	6.8802	3.88	0.6948	0.3114	33.67
Ptilonorhynchidae	*Ailuroedus buccoides*	Neotropics	0	-0.6632	8.5575	2.53	0.7580	0.7313	53.61
Ptilonorhynchidae	*Ailuroedus crassirostris*	Australasia	0	-0.0664	-	2.78	0.7611	0.2272	23.44
Ptilonorhynchidae	*Ailuroedus melanotis*	Australasia	0	-0.5746	-	3.17	0.7331	0.2679	82.92
Ptilonorhynchidae	*Amblyornis flavifrons*	Neotropics	0	-0.0180	-	3.46	0.7946	0.3006	54.07
Ptilonorhynchidae	*Amblyornis inornata*	Australasia	0	0.5990	6.2367	2.60	0.5788	0.0000	0.00
Ptilonorhynchidae	*Amblyornis macgregoriae*	Australasia	0	-0.4296	6.1496	2.54	0.6562	0.5019	53.72
Ptilonorhynchidae	*Amblyornis subalaris*	Australasia	0	-0.3029	-	2.83	0.7392	0.1834	22.88
Ptilonorhynchidae	*Archboldia papuensis*	IndoMalay	0	-0.0439	9.9248	3.40	0.7937	0.3402	31.35
Ptilonorhynchidae	*Chlamydera cerviniventris*	Neotropics	0	-0.4224	-	3.29	0.8200	0.6020	78.91
Ptilonorhynchidae	*Chlamydera guttata*	Afrotropics	0	0.0638	-	3.79	0.8298	0.1869	1.59
Ptilonorhynchidae	*Chlamydera lauterbachi*	Neotropics	0	-0.1068	-	3.93	0.8494	0.6050	29.91
Ptilonorhynchidae	*Chlamydera maculata*	Nearctic	0	-0.5453	4.4013	5.88	0.7813	0.3448	2.89
Ptilonorhynchidae	*Chlamydera nuchalis*	Palearctic	0	-1.1032	-	6.46	0.7378	0.0102	0.13
Ptilonorhynchidae	*Prionodura newtoniana*	Australasia	1	-0.9247	-	3.07	0.7096	0.5546	18.03
Ptilonorhynchidae	*Ptilonorhynchus violaceus*	Neotropics	0	-0.4260	3.1209	2.97	0.7464	0.1735	27.58
Ptilonorhynchidae	*Scenopoeetes dentirostris*	IndoMalay	0	-0.0510	-	2.65	0.7096	0.2520	74.77
Ptilonorhynchidae	*Sericulus aureus*	IndoMalay	0	-0.0733	-	3.97	0.7245	0.2465	30.35
Ptilonorhynchidae	*Sericulus bakeri*	Afrotropics	0	-0.1988	15.2987	3.27	0.7692	0.1964	72.49
Ptilonorhynchidae	*Sericulus chrysocephalus*	Afrotropics	1	0.2653	2.5221	3.68	0.7234	0.0634	72.69
Pycnonotidae	*Alophoixus affinis*	Australasia	0	-0.5456	6.2338	3.23	0.7759	0.4025	89.93
Pycnonotidae	*Alophoixus bres*	Australasia	0	-0.7168	-	2.49	0.6716	0.7398	45.94
Pycnonotidae	*Alophoixus finschii*	IndoMalay	0	-0.0489	-	2.83	0.7850	0.6333	9.36
Pycnonotidae	*Alophoixus flaveolus*	IndoMalay	0	0.0516	-	2.85	0.7836	0.4158	0.98
Pycnonotidae	*Alophoixus ochraceus*	IndoMalay	0	-0.0591	-	3.31	0.7727	0.5528	56.48
Pycnonotidae	*Alophoixus pallidus*	IndoMalay	0	-0.0041	-	2.86	0.7730	0.5370	21.80
Pycnonotidae	*Alophoixus phaeocephalus*	IndoMalay	0	-0.2364	-	3.12	0.7939	0.9349	80.91
Pycnonotidae	*Andropadus ansorgei*	Afrotropics	0	0.0470	-	3.23	0.8021	0.2907	13.84
Pycnonotidae	*Andropadus curvirostris*	Afrotropics	0	-0.0595	-	3.27	0.8187	0.3180	12.39
Pycnonotidae	*Andropadus gracilirostris*	Afrotropics	0	-0.0378	-	3.29	0.8210	0.2885	14.60
Pycnonotidae	*Andropadus gracilis*	Afrotropics	0	0.0421	-	3.28	0.8182	0.3143	15.37
Pycnonotidae	*Andropadus importunus*	Afrotropics	0	-0.1087	-	3.30	0.7928	0.2155	66.28
Pycnonotidae	*Andropadus latirostris*	Afrotropics	0	0.1483	-	3.26	0.8203	0.2856	11.98
Pycnonotidae	*Andropadus masukuensis*	Afrotropics	0	-0.1996	7.7266	3.33	0.7444	0.5559	46.44
Pycnonotidae	*Andropadus milanjensis*	Afrotropics	0	-0.2122	9.3441	3.41	0.7510	0.2050	76.60
Pycnonotidae	*Andropadus montanus*	Afrotropics	0	0.4910	10.2717	3.24	0.7008	0.4157	42.74
Pycnonotidae	*Andropadus nigriceps*	Afrotropics	0	-0.0390	13.8862	3.30	0.7510	0.7089	84.46
Pycnonotidae	*Andropadus tephrolaemus*	Afrotropics	0	0.9258	10.5616	3.21	0.6997	0.4398	43.29
Pycnonotidae	*Andropadus virens*	Afrotropics	0	-0.0700	9.0031	3.36	0.8324	0.3206	23.02
Pycnonotidae	*Baeopogon clamans*	Nearctic	0	-0.3680	8.0399	3.99	0.7704	0.4628	77.95
Pycnonotidae	*Baeopogon indicator*	Nearctic	0	-0.8231	-	4.14	0.8131	0.5925	16.35
Pycnonotidae	*Bleda canicapillus*	Afrotropics	1	-0.1540	3.6133	2.98	0.6824	0.2237	73.68
Pycnonotidae	*Bleda eximius*	Afrotropics	0	-0.1324	-	3.06	0.7340	0.1778	74.73
Pycnonotidae	*Bleda notatus*	Afrotropics	0	-0.0731	-	3.29	0.8217	0.3608	23.92
Pycnonotidae	*Bleda syndactylus*	Neotropics	1	-0.5031	4.8429	2.80	0.7348	0.3242	29.01
Pycnonotidae	*Calyptocichla serina*	Neotropics	0	-0.4699	1.7496	3.22	0.7909	0.5107	71.38
Pycnonotidae	*Chlorocichla falkensteini*	Palearctic	0	-0.8391	-	4.72	0.8353	0.1550	2.11
Pycnonotidae	*Chlorocichla flavicollis*	Palearctic	0	-0.6907	-	4.52	0.8558	0.0694	0.90
Pycnonotidae	*Chlorocichla flaviventris*	Neotropics	0	-0.3205	8.5418	3.85	0.7888	0.3599	76.32
Pycnonotidae	*Chlorocichla laetissima*	Neotropics	0	-0.5823	-	2.10	0.7725	0.4265	83.30
Pycnonotidae	*Chlorocichla prigoginei*	Neotropics	0	-0.5895	-	1.95	0.7546	0.3190	85.25
Pycnonotidae	*Chlorocichla simplex*	Neotropics	0	-0.5451	-	1.71	0.6921	0.2884	5.52
Pycnonotidae	*Criniger barbatus*	Neotropics	0	-0.4973	-	2.36	0.7701	0.4112	39.23
Pycnonotidae	*Criniger calurus*	Neotropics	0	0.0722	0.9060	4.07	0.7697	0.4112	8.56
Pycnonotidae	*Criniger chloronotus*	Neotropics	0	-0.1020	1.3858	4.00	0.8325	0.6291	38.78
Pycnonotidae	*Criniger ndussumensis*	Australasia	0	-0.0589	-	2.79	0.7565	0.1909	23.28
Pycnonotidae	*Criniger olivaceus*	Australasia	0	-0.0860	7.3185	2.82	0.7416	0.1493	24.21
Pycnonotidae	*Hemixos castanonotus*	Neotropics	1	0.2247	3.7317	4.12	0.6523	0.3358	36.62
Pycnonotidae	*Hemixos flavala*	Neotropics	0	-0.0054	4.8055	3.73	0.7548	0.4124	71.07
Pycnonotidae	*Hypsipetes leucocephalus*	Neotropics	0	-0.0359	-	3.95	0.8069	0.7722	84.22
Pycnonotidae	*Hypsipetes madagascariensis*	Neotropics	0	-0.5329	-	3.06	0.7744	0.5773	15.36
Pycnonotidae	*Hypsipetes mcclellandii*	Neotropics	0	0.3544	7.0905	4.26	0.7461	0.5555	18.16
Pycnonotidae	*Hypsipetes thompsoni*	Neotropics	0	-0.0470	7.9928	3.47	0.6974	0.1467	63.68
Pycnonotidae	*Hypsipetes virescens*	Neotropics	0	0.0929	-	4.02	0.8160	0.5599	2.43

Family	Species	Realm	Threat	Latitude	Elevation	Anomaly	Size	Shape	Orientation
Pycnonotidae	*Iole indica*	IndoMalay	0	-0.0573	10.4809	3.05	0.8158	0.4312	34.66
Pycnonotidae	*Iole olivacea*	Nearctic	0	-0.6878	-	4.13	0.7846	0.3005	4.62
Pycnonotidae	*Iole propinqua*	Neotropics	0	-0.1607	-	3.81	0.8536	0.5065	41.98
Pycnonotidae	*Iole virescens*	Neotropics	0	2.6397	7.5469	4.05	0.7060	0.2285	62.08
Pycnonotidae	*Ixonotus guttatus*	Afrotropics	0	-0.0060	9.7720	3.56	0.7963	0.6853	43.45
Pycnonotidae	*Ixos amaurotis*	Afrotropics	0	-0.0989	12.0295	3.53	0.8545	0.6736	40.85
Pycnonotidae	*Ixos everetti*	Afrotropics	0	8.1583	5.3801	3.44	0.7078	0.4358	82.87
Pycnonotidae	*Ixos malaccensis*	Afrotropics	0	-0.1833	-	3.47	0.8208	0.6666	64.20
Pycnonotidae	*Ixos palawanensis*	Afrotropics	0	0.1656	-	3.28	0.8171	0.2598	12.23
Pycnonotidae	*Ixos philippinus*	IndoMalay	0	-0.0863	13.9258	4.05	0.7537	0.1765	16.54
Pycnonotidae	*Ixos rufigularis*	Neotropics	0	0.1515	0.7568	3.86	0.7695	0.2211	15.43
Pycnonotidae	*Ixos siquijorensis*	Neotropics	0	-0.2309	2.8193	3.84	0.7866	0.6461	53.46
Pycnonotidae	*Neolestes torquatus*	Afrotropics	0	-0.5520	-	3.38	0.7654	0.4925	23.85
Pycnonotidae	*Nicator chloris*	Neotropics	0	-0.0989	-	4.04	0.8401	0.7943	58.37
Pycnonotidae	*Nicator gularis*	Australasia	0	-0.4212	-	3.49	0.7949	0.5228	57.78
Pycnonotidae	*Nicator vireo*	Australasia	0	-0.2030	-	3.30	0.7889	0.5328	1.83
Pycnonotidae	*Phyllastrephus albigularis*	Neotropics	0	-0.2807	12.9080	3.93	0.7779	0.4004	79.91
Pycnonotidae	*Phyllastrephus baumanni*	Neotropics	0	-0.6909	1.0170	4.17	0.7438	0.5327	20.50
Pycnonotidae	*Phyllastrephus cabanisi*	Neotropics	0	-0.2362	-	3.70	0.7821	0.2399	69.40
Pycnonotidae	*Phyllastrephus cerviniventris*	Neotropics	0	0.0089	8.4483	3.65	0.7626	0.4948	88.85
Pycnonotidae	*Phyllastrephus debilis*	Neotropics	0	-0.1435	-	3.72	0.7517	0.2160	36.11
Pycnonotidae	*Phyllastrephus fischeri*	Nearctic	0	-0.5824	-	4.27	0.8227	0.2566	17.76
Pycnonotidae	*Phyllastrephus flavostriatus*	Nearctic	0	-0.5812	12.3608	4.10	0.8256	0.5422	54.96
Pycnonotidae	*Phyllastrephus fulviventris*	Neotropics	0	-0.1562	9.5615	3.04	0.6824	0.2322	31.68
Pycnonotidae	*Phyllastrephus hypochloris*	Neotropics	0	-0.2920	8.2644	3.11	0.7853	0.4185	40.15
Pycnonotidae	*Phyllastrephus leucolepis*	Australasia	0	-0.0356	-	3.29	0.7797	0.4203	1.54
Pycnonotidae	*Phyllastrephus poensis*	Australasia	0	-0.4757	1.0490	2.69	0.6428	0.6065	24.41
Pycnonotidae	*Phyllastrephus poliocephalus*	Australasia	0	0.0108	1.8887	2.83	0.7584	0.4216	16.30
Pycnonotidae	*Phyllastrephus strepitans*	Australasia	0	-0.3772	3.1012	3.34	0.8152	0.4930	43.03
Pycnonotidae	*Phyllastrephus terrestris*	Australasia	0	-0.1666	-	2.49	0.6662	0.3153	20.62
Pycnonotidae	*Phyllastrephus xavieri*	Australasia	0	-0.3715	-	3.29	0.7973	0.3619	79.25
Pycnonotidae	*Pycnonotus atriceps*	Australasia	0	-0.1922	5.5635	2.56	0.6523	0.1875	30.24
Pycnonotidae	*Pycnonotus aurigaster*	Australasia	0	-0.2319	-	3.42	0.7879	0.3731	11.57
Pycnonotidae	*Pycnonotus barbatus*	IndoMalay	0	-0.0152	1.3947	2.84	0.7886	0.4712	5.29
Pycnonotidae	*Pycnonotus blanfordi*	Australasia	1	-0.0710	6.0428	2.79	0.7606	0.2041	23.07
Pycnonotidae	*Pycnonotus brunneus*	Neotropics	0	-0.0494	-	3.96	0.8113	-	-
Pycnonotidae	*Pycnonotus brunneus*	Neotropics	0	-0.0494	-	3.96	0.8113	0.4182	11.23
Pycnonotidae	*Pycnonotus cafer*	Neotropics	0	0.0938	-	4.14	0.7956	0.8878	13.63
Pycnonotidae	*Pycnonotus capensis*	Neotropics	0	-0.1288	-	4.14	0.7940	0.4374	30.28
Pycnonotidae	*Pycnonotus capensis*	Neotropics	0	-0.1288	-	4.14	0.7940	-	-
Pycnonotidae	*Pycnonotus cyaniventris*	Afrotropics	0	-0.0915	21.7365	3.88	0.8220	0.4790	62.94
Pycnonotidae	*Pycnonotus erythropthalmos*	Australasia	0	-0.7175	-	3.26	0.7866	0.8505	10.34
Pycnonotidae	*Pycnonotus eutilotus*	Australasia	0	-0.8068	-	2.67	0.7331	0.1363	5.71
Pycnonotidae	*Pycnonotus finlaysoni*	Australasia	0	-0.6241	-	3.54	0.8075	0.4298	4.08
Pycnonotidae	*Pycnonotus flavescens*	Australasia	0	-0.5545	-	3.09	0.7576	0.3912	89.77
Pycnonotidae	*Pycnonotus goiavier*	Australasia	0	0.2080	4.2493	2.89	0.7266	0.1408	16.72
Pycnonotidae	*Pycnonotus hualon*	Palearctic	0	-1.0086	-	4.07	0.8193	0.1522	5.69
Pycnonotidae	*Pycnonotus jocosus*	Afrotropics	0	-0.1996	-	4.00	0.8014	0.6139	1.37
Pycnonotidae	*Pycnonotus leucogenys*	Afrotropics	0	-0.4997	-	4.34	0.7889	0.7490	19.91
Pycnonotidae	*Pycnonotus leucogrammicus*	Palearctic	0	-0.4079	5.1788	4.09	0.8306	0.1872	3.86
Pycnonotidae	*Pycnonotus leucotis*	Afrotropics	0	0.0203	-	3.27	0.7915	0.3823	56.61
Pycnonotidae	*Pycnonotus luteolus*	Afrotropics	0	-0.1323	-	3.64	0.8462	0.2489	5.91
Pycnonotidae	*Pycnonotus melanicterus*	Afrotropics	0	-2.8646	6.9183	3.75	0.7855	0.2387	61.36
Pycnonotidae	*Pycnonotus melanoleucos*	IndoMalay	0	-0.1504	-	3.48	0.8019	0.6879	76.86
Pycnonotidae	*Pycnonotus nieuwenhuisii*	Palearctic	0	-0.1503	8.0147	3.89	0.8250	0.3423	7.38
Pycnonotidae	*Pycnonotus nigricans*	Afrotropics	0	-0.2777	-	4.04	0.8030	0.6769	56.69
Pycnonotidae	*Pycnonotus penicillatus*	Palearctic	0	-1.0169	-	4.14	0.8332	0.1502	5.63
Pycnonotidae	*Pycnonotus plumosus*	Afrotropics	0	-0.2768	-	3.24	0.7416	0.3024	76.37
Pycnonotidae	*Pycnonotus priocephalus*	Afrotropics	0	0.0909	-	3.58	0.8233	0.1635	5.01
Pycnonotidae	*Pycnonotus simplex*	Palearctic	0	-0.3676	-	4.07	0.8467	0.2703	1.31
Pycnonotidae	*Pycnonotus sinensis*	Australasia	1	-0.6062	0.0000	2.48	0.6302	0.1525	48.23
Pycnonotidae	*Pycnonotus squamatus*	Australasia	1	-0.6048	1.1797	3.04	0.6562	0.1032	79.81
Pycnonotidae	*Pycnonotus striatus*	Australasia	1	-0.2259	-	2.51	0.6690	0.5037	33.72
Pycnonotidae	*Pycnonotus striatus*	Australasia	1	-0.2259	-	2.51	0.6690	-	-
Pycnonotidae	*Pycnonotus tympanistrigus*	Australasia	1	1.3090	0.3045	2.73	0.6763	0.0453	7.86
Pycnonotidae	*Pycnonotus urostictus*	Australasia	1	-0.4162	2.0276	2.39	0.7128	0.0514	10.06
Pycnonotidae	*Pycnonotus xantholaemus*	Neotropics	1	-0.7137	2.2218	2.21	0.7179	0.2232	38.60
Pycnonotidae	*Pycnonotus xanthopygos*	Nearctic	0	-0.7354	1.3757	3.47	0.6370	0.1028	38.27
Pycnonotidae	*Pycnonotus xanthorrhous*	Neotropics	1	-0.3637	2.2192	2.80	0.7228	0.3471	45.74
Pycnonotidae	*Pycnonotus zeylanicus*	Australasia	0	-0.2518	3.1952	2.52	0.6631	0.4604	29.52
Pycnonotidae	*Pyrrhurus scandens*	Australasia	0	-0.0466	4.8702	2.78	0.7609	0.2029	23.58
Pycnonotidae	*Setornis criniger*	Neotropics	0	0.0325	8.1372	3.83	0.7674	0.4462	74.58
Pycnonotidae	*Spizixos canifrons*	Afrotropics	0	-0.3258	2.2975	3.44	0.7648	0.3788	70.35
Pycnonotidae	*Spizixos semitorques*	Afrotropics	0	0.0698	5.5167	3.36	0.7735	0.4663	27.13

Family	Species	Realm	Threat	Latitude	Elevation	Anomaly	Size	Shape	Orientation
Pycnonotidae	*Thescelocichla leucopleura*	Neotropics	0	-1.3478	5.5814	3.77	0.7344	0.2875	55.21
Pycnonotidae	*Tricholestes criniger*	Neotropics	1	-0.1067	3.0754	3.06	0.7240	0.1722	73.72
Rallidae	*Aenigmatolimnas marginalis*	Afrotropics	0	-0.0081	-	3.55	0.8253	0.6306	48.16
Rallidae	*Amaurolimnas concolor*	Afrotropics	0	-0.0901	8.5009	3.51	0.8273	0.5740	47.13
Rallidae	*Amaurornis akool*	Neotropics	0	-0.1143	2.5379	3.71	0.8009	0.3712	29.64
Rallidae	*Amaurornis bicolor*	IndoMalay	0	-0.4851	-	3.35	0.8209	0.2450	6.43
Rallidae	*Amaurornis flavirostra*	IndoMalay	0	-0.1201	12.7689	3.38	0.7946	0.7062	5.50
Rallidae	*Amaurornis isabellina*	Afrotropics	0	-0.0837	11.9520	3.54	0.8596	0.6435	42.72
Rallidae	*Amaurornis moluccana*	Australasia	0	-0.0101	2.6133	2.71	0.7179	0.5352	59.02
Rallidae	*Amaurornis olivacea*	Australasia	0	-0.3058	3.6108	2.90	0.7901	0.5067	56.77
Rallidae	*Amaurornis olivieri*	IndoMalay	0	0.0009	-	2.64	0.7327	0.2318	67.52
Rallidae	*Amaurornis phoenicurus*	Afrotropics	1	-0.2203	0.2192	3.16	0.6891	0.2392	75.89
Rallidae	*Anurolimnas castaneiceps*	Neotropics	0	0.0925	5.9751	3.88	0.7684	0.3164	59.94
Rallidae	*Anurolimnas fasciatus*	Neotropics	0	0.0624	1.5587	3.98	0.7902	0.5957	16.32
Rallidae	*Anurolimnas viridis*	Neotropics	0	-0.0964	4.0939	4.01	0.8340	0.7569	31.44
Rallidae	*Aramides axillaris*	IndoMalay	0	-0.0785	-	2.99	0.8196	0.5233	48.05
Rallidae	*Aramides calopterus*	IndoMalay	0	-0.0340	-	2.78	0.7697	0.6549	19.18
Rallidae	*Aramides mangle*	Neotropics	0	0.0836	-	3.33	0.7604	0.2743	18.91
Rallidae	*Aramides saracura*	Neotropics	0	-0.2078	-	3.77	0.8544	0.5416	42.55
Rallidae	*Aramides wolfi*	Neotropics	0	0.1950	-	3.99	0.7705	0.5015	25.96
Rallidae	*Aramides ypecaha*	Neotropics	0	-0.1648	-	3.35	0.7747	0.5099	85.22
Rallidae	*Aramidopsis plateni*	Neotropics	0	-0.4236	-	2.94	0.7794	0.3181	31.55
Rallidae	*Canirallus kioloides*	Neotropics	0	-0.0274	-	2.94	0.7112	0.3861	85.34
Rallidae	*Canirallus oculeus*	Nearctic	0	-0.4762	-	4.01	0.8012	0.4830	31.47
Rallidae	*Coturnicops exquisitus*	Afrotropics	0	-0.1245	-	3.51	0.8019	0.2505	67.77
Rallidae	*Coturnicops notatus*	Afrotropics	0	0.1926	-	3.26	0.8043	0.1543	11.05
Rallidae	*Coturnicops noveboracensis*	Afrotropics	0	-0.5562	-	3.25	0.7425	0.3743	58.92
Rallidae	*Crecopsis egregia*	Neotropics	0	-0.0834	0.1779	3.90	0.7165	0.2190	23.54
Rallidae	*Crex crex*	Neotropics	0	-0.5880	-	3.02	0.8033	0.6535	86.14
Rallidae	*Cyanolimnas cerverai*	Neotropics	0	-0.2616	5.4575	3.47	0.8319	0.7328	44.70
Rallidae	*Eulabeornis castaneoventris*	Afrotropics	1	-0.1173	-	3.00	0.6804	0.7810	11.58
Rallidae	*Fulica alai*	Afrotropics	0	-0.1792	-	4.26	0.7846	0.4152	40.36
Rallidae	*Fulica americana*	Afrotropics	1	-0.4319	4.9205	3.32	0.7192	0.2790	1.08
Rallidae	*Fulica ardesiaca*	Afrotropics	0	-0.3676	-	3.76	0.7875	0.4699	83.08
Rallidae	*Fulica armillata*	Afrotropics	0	-6.6564	12.6354	3.44	0.6859	0.3048	76.77
Rallidae	*Fulica atra*	Afrotropics	1	0.6360	4.7305	3.50	0.6120	0.6246	0.00
Rallidae	*Fulica caribaea*	IndoMalay	0	-0.0384	-	3.36	0.7948	0.7400	86.59
Rallidae	*Fulica cornuta*	IndoMalay	0	-0.5414	-	3.12	0.8033	0.4383	24.21
Rallidae	*Fulica cristata*	IndoMalay	0	-0.0845	-	3.61	0.8184	-	-
Rallidae	*Fulica cristata*	IndoMalay	0	-0.0845	-	3.61	0.8184	0.5448	16.77
Rallidae	*Fulica gigantea*	Afrotropics	0	-1.0578	12.8945	3.42	0.7050	0.2203	69.72
Rallidae	*Fulica leucoptera*	Afrotropics	0	-2.5463	-	3.23	0.7050	0.7531	51.11
Rallidae	*Fulica rufifrons*	Afrotropics	0	12.1691	-	3.46	0.7660	0.2411	7.90
Rallidae	*Gallicrex cinerea*	Neotropics	0	0.1945	0.8781	3.91	0.7300	0.5053	20.28
Rallidae	*Gallinula angulata*	Afrotropics	0	0.0227	-	3.48	0.8167	0.1412	8.03
Rallidae	*Gallinula angulata*	Afrotropics	0	0.0227	-	3.48	0.8167	-	-
Rallidae	*Gallinula chloropus*	Palearctic	0	-0.3652	12.3756	3.62	0.8066	0.2074	29.92
Rallidae	*Gallinula melanops*	Australasia	0	-0.0910	8.2898	2.76	0.7482	0.2197	15.52
Rallidae	*Gallinula mortierii*	IndoMalay	1	-0.0455	2.1021	2.62	0.7019	0.6235	86.79
Rallidae	*Gallinula silvestris*	Australasia	1	-0.1488	3.7353	2.56	0.6523	0.1875	30.24
Rallidae	*Gallinula tenebrosa*	Australasia	0	-0.0595	-	2.79	0.7669	0.3047	16.36
Rallidae	*Gallinula ventralis*	IndoMalay	1	-0.0175	3.5791	2.63	0.6478	0.3503	71.22
Rallidae	*Gallirallus australis*	IndoMalay	0	-0.0102	3.2631	2.66	0.6997	0.4736	75.00
Rallidae	*Gallirallus insignis*	IndoMalay	1	2.7802	1.6075	2.65	0.5788	0.0000	0.00
Rallidae	*Gallirallus lafresnayanus*	Australasia	0	-0.0947	2.1427	2.78	0.7716	0.3088	23.58
Rallidae	*Gymnocrex plumbeiventris*	Neotropics	0	15.8518	2.4280	3.40	0.6523	0.2390	76.88
Rallidae	*Gymnocrex rosenbergii*	Australasia	0	-0.0518	-	2.81	0.7519	0.1845	24.84
Rallidae	*Habroptila wallacii*	Afrotropics	0	0.0865	-	3.20	0.8005	0.4047	30.44
Rallidae	*Himantornis haematopus*	Neotropics	0	-0.0713	4.5517	3.67	0.7821	0.9170	35.83
Rallidae	*Laterallus albigularis*	Afrotropics	0	-0.0171	-	3.24	0.7165	0.3407	48.06
Rallidae	*Laterallus exilis*	Neotropics	0	-0.3639	7.8205	3.20	0.7621	0.3635	21.19
Rallidae	*Laterallus jamaicensis*	Neotropics	0	-0.2099	-	3.56	0.7406	0.3942	9.30
Rallidae	*Laterallus leucopyrrhus*	Neotropics	0	-0.0234	-	3.98	0.8070	0.3812	12.50
Rallidae	*Laterallus levraudi*	Neotropics	0	0.0573	-	3.22	0.7198	0.3370	64.99
Rallidae	*Laterallus melanophaius*	Neotropics	0	0.1312	-	4.16	0.8129	0.4655	6.66
Rallidae	*Laterallus ruber*	Neotropics	0	-0.1394	-	4.02	0.8354	0.7107	6.88
Rallidae	*Laterallus spilonotus*	Neotropics	0	0.0821	-	3.32	0.7614	0.2578	36.58
Rallidae	*Laterallus tuerosi*	Afrotropics	0	-0.2533	-	3.93	0.7515	0.2632	77.36
Rallidae	*Laterallus xenopterus*	Palearctic	0	-1.1580	3.7342	3.81	0.7972	0.3215	14.85
Rallidae	*Lewinia mirifica*	Neotropics	0	-0.0008	8.9812	3.75	0.7586	0.2964	85.61
Rallidae	*Lewinia pectoralis*	Neotropics	0	6.1511	16.3042	3.75	0.7574	0.2269	83.12
Rallidae	*Megacrex inepta*	Neotropics	0	-0.0007	8.4844	3.37	0.7291	0.3712	41.26
Rallidae	*Micropygia schomburgkii*	Afrotropics	0	-0.3460	-	3.91	0.7868	0.3061	6.86
Rallidae	*Neocrex colombiana*	Afrotropics	0	-0.1196	-	3.51	0.8117	0.8592	40.48

Family	Species	Realm	Threat	Latitude	Elevation	Anomaly	Size	Shape	Orientation
Rallidae	*Neocrex erythrops*	Afrotropics	0	-16.2974	-	3.73	0.7234	0.4120	50.60
Rallidae	*Nesoclopeus woodfordi*	Afrotropics	0	-0.1321	-	3.57	0.8469	0.6905	37.74
Rallidae	*Pardirallus maculatus*	Australasia	0	-0.0957	-	3.24	0.7469	0.2603	15.93
Rallidae	*Pardirallus nigricans*	Australasia	0	0.0419	-	2.71	0.6428	0.0719	45.34
Rallidae	*Pardirallus nigricans*	Australasia	0	0.0419	-	2.71	0.6428	-	-
Rallidae	*Pardirallus sanguinolentus*	Australasia	0	-0.7005	11.5874	2.86	0.6961	0.1971	15.18
Rallidae	*Porphyrio alleni*	Neotropics	0	-0.0004	2.3865	3.68	0.8042	0.1380	24.15
Rallidae	*Porphyrio flavirostris*	Afrotropics	0	-0.1108	-	3.24	0.7399	0.4024	70.56
Rallidae	*Porphyrio hochstetteri*	Afrotropics	0	0.0318	-	3.24	0.7811	0.2628	12.56
Rallidae	*Porphyrio porphyrio*	Australasia	0	-0.0612	-	3.39	0.7770	0.4242	1.81
Rallidae	*Porzana albicollis*	Afrotropics	0	-0.0377	-	3.25	0.8156	0.2662	14.15
Rallidae	*Porzana carolina*	Afrotropics	0	-0.0553	11.5701	3.34	0.8382	0.3802	26.72
Rallidae	*Porzana cinerea*	Afrotropics	0	-0.0841	12.1252	3.60	0.8477	0.5644	40.04
Rallidae	*Porzana flaviventer*	Afrotropics	0	-0.0427	11.8054	3.44	0.7519	0.9354	0.43
Rallidae	*Porzana fluminea*	Afrotropics	0	-0.0114	10.3825	3.29	0.7681	0.2577	80.88
Rallidae	*Porzana fusca*	Afrotropics	0	0.0000	9.6318	3.82	0.6120	0.9615	90.00
Rallidae	*Porzana parva*	Afrotropics	0	-0.1080	8.7992	3.32	0.8094	0.2686	73.67
Rallidae	*Porzana paykullii*	Afrotropics	0	0.0263	-	3.28	0.8216	0.3382	14.71
Rallidae	*Porzana porzana*	Afrotropics	0	-0.1203	5.3772	3.29	0.7648	0.2686	75.73
Rallidae	*Porzana pusilla*	Afrotropics	0	0.0504	8.8851	3.26	0.8181	0.2816	13.61
Rallidae	*Porzana spiloptera*	Afrotropics	0	-0.1316	15.7220	3.34	0.7997	0.2700	79.01
Rallidae	*Porzana tabuensis*	Afrotropics	0	6.4999	-	3.38	0.7522	0.2303	10.23
Rallidae	*Rallina eurizonoides*	IndoMalay	1	0.0821	2.0585	2.84	0.7676	0.6488	45.81
Rallidae	*Rallina fasciata*	IndoMalay	0	-0.0469	3.8731	2.62	0.6986	0.4794	89.83
Rallidae	*Rallina forbesi*	Neotropics	0	-0.0505	7.0286	3.05	0.6562	0.3979	32.60
Rallidae	*Rallina leucospila*	Neotropics	0	-0.0647	13.8673	3.79	0.7716	0.2751	34.00
Rallidae	*Rallina mayri*	Australasia	0	-0.5508	-	3.10	0.7629	0.4107	88.41
Rallidae	*Rallina rubra*	Afrotropics	0	0.0813	5.0670	3.49	0.8272	0.2014	4.60
Rallidae	*Rallina tricolor*	Australasia	0	0.1881	8.3546	2.76	0.7096	0.2199	24.25
Rallidae	*Rallus antarcticus*	Australasia	0	-0.2676	9.8996	2.83	0.7205	0.2965	37.49
Rallidae	*Rallus aquaticus*	Australasia	0	-0.8058	-	2.62	0.6120	0.1467	8.55
Rallidae	*Rallus caerulescens*	Australasia	0	-0.2697	7.3707	2.84	0.7392	0.1734	23.03
Rallidae	*Rallus elegans*	Australasia	0	-0.2258	13.2574	2.85	0.7296	0.1604	22.45
Rallidae	*Rallus limicola*	Australasia	0	-0.2246	-	2.84	0.7378	0.1604	24.04
Rallidae	*Rallus longirostris*	Australasia	0	-0.1819	-	2.74	0.7158	0.3045	49.27
Rallidae	*Rallus madagascariensis*	Australasia	0	-0.0839	-	2.78	0.7702	0.3803	28.16
Rallidae	*Rallus semiplumbeus*	Australasia	0	-0.4913	-	3.19	0.7185	0.2906	82.72
Rallidae	*Rallus wetmorei*	Australasia	0	-0.1962	-	3.02	0.6763	0.1987	72.37
Rallidae	*Rougetius rougetii*	Neotropics	0	-0.0395	-	3.42	0.7458	0.2739	32.56
Rallidae	*Rougetius rougetii*	Neotropics	0	-0.0395	-	3.42	0.7458	-	-
Rallidae	*Sarothrura affinis*	Neotropics	0	0.1215	-	4.02	0.8019	0.7504	22.96
Rallidae	*Sarothrura ayresi*	IndoMalay	0	-0.1371	5.4292	2.82	0.7158	0.4421	85.39
Rallidae	*Sarothrura boehmi*	Neotropics	0	-0.5339	5.6805	3.03	0.7832	0.2749	72.47
Rallidae	*Sarothrura elegans*	Australasia	0	-0.0713	5.2676	2.49	0.5788	0.0000	0.00
Rallidae	*Sarothrura insularis*	IndoMalay	1	-0.0372	2.4244	2.63	0.6478	0.3503	71.22
Rallidae	*Sarothrura lugens*	IndoMalay	1	-0.7306	3.5971	3.28	0.7712	0.5400	23.07
Rallidae	*Sarothrura pulchra*	Australasia	0	0.5127	1.2199	2.77	0.6370	0.3389	24.54
Rallidae	*Sarothrura rufa*	IndoMalay	0	-0.2063	-	2.64	0.6740	-	-
Rallidae	*Sarothrura rufa*	IndoMalay	0	-0.2063	-	2.64	0.6740	0.7803	73.94
Rallidae	*Sarothrura watersi*	IndoMalay	0	0.0712	-	2.90	0.7019	0.2778	63.37
Ramphastidae	*Andigena cucullata*	Neotropics	0	1.3089	5.6085	4.03	0.7040	0.2136	36.42
Ramphastidae	*Andigena hypoglauca*	Neotropics	0	0.1286	7.8879	3.58	0.7371	0.3000	79.25
Ramphastidae	*Andigena laminirostris*	Neotropics	0	-0.6078	7.2038	3.08	0.6716	0.2439	69.37
Ramphastidae	*Andigena nigrirostris*	Neotropics	0	0.0451	6.4035	3.43	0.7363	0.2267	57.90
Ramphastidae	*Aulacorhynchus coeruleicinctis*	Neotropics	0	-0.0925	-	3.89	0.8391	0.4267	30.77
Ramphastidae	*Aulacorhynchus derbianus*	Neotropics	1	0.1965	2.7865	3.09	0.7096	0.3360	77.84
Ramphastidae	*Aulacorhynchus haematopygus*	Neotropics	0	-2.8323	4.0012	3.68	0.6935	0.6195	73.78
Ramphastidae	*Aulacorhynchus huallagae*	Neotropics	0	-3.1094	6.2025	3.56	0.7030	0.3350	80.22
Ramphastidae	*Aulacorhynchus prasinus*	Neotropics	0	0.9205	9.1250	4.05	0.7286	0.1135	32.82
Ramphastidae	*Aulacorhynchus sulcatus*	Neotropics	0	0.1788	9.3539	4.05	0.7661	0.5329	60.15
Ramphastidae	*Buccanodon duchaillui*	Afrotropics	0	0.0491	6.8833	3.27	0.8201	0.3730	12.70
Ramphastidae	*Calorhamphus fuliginosus*	Neotropics	0	-0.2198	-	3.81	0.8406	0.6878	42.38
Ramphastidae	*Capito auratus*	Neotropics	0	-0.1545	-	3.54	0.6370	0.2466	46.13
Ramphastidae	*Capito aurovirens*	Neotropics	0	-0.1101	-	3.32	0.7266	0.2513	69.29
Ramphastidae	*Capito brunneipectus*	Neotropics	0	-0.0744	-	3.91	0.7809	0.4951	9.42
Ramphastidae	*Capito dayi*	Neotropics	0	-0.4925	10.5611	3.85	0.7458	0.3535	42.20
Ramphastidae	*Capito hypoleucus*	Neotropics	0	0.8743	9.1552	3.67	0.7040	0.4075	18.87
Ramphastidae	*Capito maculicoronatus*	Neotropics	0	0.5568	6.4660	3.82	0.7120	0.2370	18.74
Ramphastidae	*Capito niger*	Neotropics	0	-0.2932	-	3.97	0.7623	0.2901	2.32
Ramphastidae	*Capito quinticolor*	Neotropics	0	0.4138	-	3.51	0.7446	0.1268	21.43
Ramphastidae	*Capito squamatus*	Neotropics	0	-0.1242	-	3.99	0.8256	0.5739	21.24
Ramphastidae	*Capito wallacei*	Neotropics	0	-1.1184	-	2.57	0.6428	0.2068	19.80
Ramphastidae	*Eubucco bourcierii*	Australasia	0	-0.1024	-	2.58	0.6302	0.1134	34.50
Ramphastidae	*Eubucco tucinkae*	Australasia	0	-0.0908	10.6363	2.74	0.7759	0.2594	12.86

Family	Species	Realm	Threat	Latitude	Elevation	Anomaly	Size	Shape	Orientation
Ramphastidae	*Eubucco versicolor*	Australasia	0	0.0306	-	2.55	0.6562	0.1420	18.65
Ramphastidae	*Gymnobucco bonapartei*	Neotropics	0	0.0329	10.4049	3.60	0.7584	0.2248	62.89
Ramphastidae	*Gymnobucco calvus*	Neotropics	0	3.1335	3.0804	3.27	0.6598	0.1232	71.55
Ramphastidae	*Gymnobucco peli*	Neotropics	0	-0.5223	4.2265	3.82	0.6562	0.2537	4.18
Ramphastidae	*Gymnobucco sladeni*	Neotropics	0	-0.0095	11.8963	3.63	0.7385	0.4232	30.71
Ramphastidae	*Lybius bidentatus*	Neotropics	0	0.1167	8.6148	3.56	0.7749	0.4747	85.68
Ramphastidae	*Lybius chaplini*	Neotropics	0	-0.0179	-	3.86	0.8055	0.3331	16.41
Ramphastidae	*Lybius dubius*	IndoMalay	0	-0.0755	3.4772	2.99	0.8173	0.5635	41.45
Ramphastidae	*Lybius guifsobalito*	Australasia	0	-0.1545	5.3970	2.63	0.6478	0.0000	0.00
Ramphastidae	*Lybius leucocephalus*	IndoMalay	0	-0.0685	-	2.64	0.6716	0.7479	80.97
Ramphastidae	*Lybius melanopterus*	IndoMalay	0	-0.2834	-	2.72	0.6921	0.1009	10.98
Ramphastidae	*Lybius minor*	Australasia	0	0.7491	-	2.47	0.5788	0.0000	0.00
Ramphastidae	*Lybius rolleti*	Australasia	0	0.4575	-	2.72	0.7060	0.5118	62.13
Ramphastidae	*Lybius rubrifacies*	Australasia	0	0.0302	-	2.63	0.6428	0.0000	0.00
Ramphastidae	*Lybius torquatus*	IndoMalay	1	0.1252	5.8920	2.88	0.7512	0.5472	59.34
Ramphastidae	*Lybius undatus*	IndoMalay	0	-0.1605	3.5776	3.02	0.7680	0.5263	46.58
Ramphastidae	*Lybius vieilloti*	IndoMalay	1	-0.1060	0.7289	2.72	0.6302	0.2799	41.30
Ramphastidae	*Megalaima armillaris*	Neotropics	0	0.0289	-	3.25	0.7672	0.3708	49.09
Ramphastidae	*Megalaima asiatica*	Neotropics	0	-0.0838	-	4.13	0.8104	0.4649	1.66
Ramphastidae	*Megalaima australis*	Neotropics	0	0.0756	3.5092	4.20	0.7691	0.7048	45.46
Ramphastidae	*Megalaima chrysopogon*	Neotropics	0	-0.2481	-	3.25	0.7747	0.5850	55.58
Ramphastidae	*Megalaima corvina*	Australasia	0	-0.7052	0.0000	3.12	0.7945	0.3601	3.36
Ramphastidae	*Megalaima eximia*	Afrotropics	0	-0.4846	-	3.94	0.7895	0.6455	47.49
Ramphastidae	*Megalaima faber*	Australasia	0	0.5035	-	2.72	0.7060	0.4939	55.68
Ramphastidae	*Megalaima faiostricta*	Afrotropics	1	-0.0606	1.7821	3.12	0.6906	0.4423	45.69
Ramphastidae	*Megalaima flavifrons*	Afrotropics	0	-0.0329	-	3.25	0.7845	0.3599	4.42
Ramphastidae	*Megalaima franklinii*	Afrotropics	0	0.3568	-	3.28	0.7659	0.3075	5.93
Ramphastidae	*Megalaima haemacephala*	Afrotropics	0	0.1524	-	3.27	0.7946	0.3519	6.22
Ramphastidae	*Megalaima henricii*	Afrotropics	1	0.5354	-	3.11	0.6690	0.3947	0.51
Ramphastidae	*Megalaima incognita*	Afrotropics	0	-0.0636	-	3.24	0.8106	0.2558	11.75
Ramphastidae	*Megalaima javensis*	Afrotropics	0	0.1479	-	3.25	0.8122	0.2362	13.35
Ramphastidae	*Megalaima lagrandieri*	Afrotropics	0	0.4891	-	3.14	0.7344	0.4457	65.89
Ramphastidae	*Megalaima lineata*	Afrotropics	0	0.0348	-	3.26	0.8122	0.2728	12.50
Ramphastidae	*Megalaima monticola*	Afrotropics	0	0.0566	-	3.14	0.7762	0.1432	3.07
Ramphastidae	*Megalaima mystacophanos*	Australasia	0	-0.0828	-	2.77	0.7597	0.3406	25.88
Ramphastidae	*Megalaima nuchalis*	Australasia	0	-0.1872	-	2.93	0.7165	0.2623	59.02
Ramphastidae	*Megalaima oorti*	Australasia	0	-0.3039	-	3.46	0.7537	0.2484	2.54
Ramphastidae	*Megalaima pulcherrima*	Australasia	0	-0.6027	-	3.07	0.7873	0.5705	56.85
Ramphastidae	*Megalaima rafflesii*	Australasia	0	-0.0970	-	2.77	0.7623	0.3223	26.31
Ramphastidae	*Megalaima rubricapillus*	Australasia	0	-0.8603	-	2.43	0.6948	0.5612	63.66
Ramphastidae	*Megalaima virens*	Australasia	0	-0.1159	3.0469	2.69	0.7331	0.2509	16.20
Ramphastidae	*Megalaima viridis*	Australasia	0	-0.5567	-	3.44	0.8324	0.4796	11.23
Ramphastidae	*Megalaima zeylanica*	Australasia	0	-0.6820	-	3.40	0.8302	0.4378	6.82
Ramphastidae	*Pogoniulus atroflavus*	Afrotropics	1	0.0000	0.0000	3.26	0.5985	0.5774	45.00
Ramphastidae	*Pogoniulus bilineatus*	Afrotropics	0	-0.5478	-	3.43	0.7724	0.4748	33.25
Ramphastidae	*Pogoniulus chrysoconus*	Afrotropics	0	10.3325	-	3.16	0.6906	0.8677	51.70
Ramphastidae	*Pogoniulus coryphaeus*	Afrotropics	0	5.5602	11.8895	3.34	0.7403	0.8396	60.38
Ramphastidae	*Pogoniulus leucomystax*	Afrotropics	0	-0.1794	-	3.48	0.8557	0.5229	27.16
Ramphastidae	*Pogoniulus makawai*	Afrotropics	0	0.1105	4.9426	3.34	0.7464	0.5389	53.17
Ramphastidae	*Pogoniulus pusillus*	Afrotropics	0	0.1927	-	3.28	0.7550	0.2083	10.07
Ramphastidae	*Pogoniulus scolopaceus*	Afrotropics	1	0.0000	-	3.40	0.6370	0.9258	0.00
Ramphastidae	*Pogoniulus simplex*	Afrotropics	0	-0.0102	8.4842	3.60	0.7877	0.9217	1.88
Ramphastidae	*Pogoniulus subsulphureus*	Afrotropics	1	-0.2275	-	3.04	0.5788	0.0000	90.00
Ramphastidae	*Psilopogon pyrolophus*	Australasia	0	-0.3797	-	3.03	0.6562	0.2427	65.64
Ramphastidae	*Pteroglossus aracari*	Afrotropics	0	0.0636	-	3.25	0.8129	0.2327	13.42
Ramphastidae	*Pteroglossus azara*	Afrotropics	0	0.1565	-	3.30	0.7937	0.2634	7.45
Ramphastidae	*Pteroglossus bailloni*	Afrotropics	0	-0.1031	9.7007	3.40	0.8390	0.9054	60.38
Ramphastidae	*Pteroglossus beauharnaesii*	IndoMalay	0	-0.3027	-	2.74	0.6740	0.2180	12.79
Ramphastidae	*Pteroglossus bitorquatus*	Nearctic	0	-0.6472	13.6345	4.01	0.8147	0.3206	45.52
Ramphastidae	*Pteroglossus castanotis*	IndoMalay	0	-0.0502	9.9648	3.18	0.7986	0.4688	52.96
Ramphastidae	*Pteroglossus frantzii*	Neotropics	0	0.0374	-	3.87	0.8086	0.5376	68.05
Ramphastidae	*Pteroglossus inscriptus*	Neotropics	0	0.5615	9.3658	4.05	0.7344	0.1235	32.42
Ramphastidae	*Pteroglossus pluricinctus*	Neotropics	0	0.1980	2.7179	4.06	0.8260	0.5862	17.43
Ramphastidae	*Pteroglossus torquatus*	Neotropics	1	-0.0053	1.2815	2.96	0.6562	0.2670	85.15
Ramphastidae	*Pteroglossus viridis*	Neotropics	0	-0.1654	-	3.89	0.8480	0.5782	47.40
Ramphastidae	*Ramphastos ambiguus*	Australasia	0	-0.1118	-	2.78	0.7637	0.2626	23.22
Ramphastidae	*Ramphastos brevis*	Australasia	0	-0.0612	-	2.78	0.7572	0.1975	23.57
Ramphastidae	*Ramphastos dicolorus*	Australasia	0	-0.0882	8.2921	2.81	0.7485	0.1693	23.75
Ramphastidae	*Ramphastos sulfuratus*	Afrotropics	0	-0.0772	-	3.47	0.8215	0.1728	6.86
Ramphastidae	*Ramphastos swainsonii*	Afrotropics	0	-13.9625	-	3.48	0.7480	0.5883	68.03
Ramphastidae	*Ramphastos toco*	Palearctic	0	-0.7291	10.6345	3.63	0.7984	0.3400	0.63
Ramphastidae	*Ramphastos tucanus*	Australasia	0	-0.5492	-	2.53	0.7674	0.2652	8.12
Ramphastidae	*Ramphastos vitellinus*	Neotropics	1	-0.7355	2.5932	3.19	0.6740	0.0736	31.61
Ramphastidae	*Selenidera gouldii*	Neotropics	0	-0.0610	-	3.40	0.7752	0.3417	29.83

Family	Species	Realm	Threat	Latitude	Elevation	Anomaly	Size	Shape	Orientation
Ramphastidae	*Selenidera maculirostris*	Neotropics	0	-0.2520	4.0181	3.84	0.8118	0.5937	39.88
Ramphastidae	*Selenidera nattereri*	Neotropics	0	0.2536	8.4223	3.16	0.7318	0.1913	58.21
Ramphastidae	*Selenidera piperivora*	Neotropics	0	-0.3312	6.5216	3.21	0.8099	0.5214	63.38
Ramphastidae	*Selenidera reinwardtii*	Neotropics	0	-0.1971	7.9603	3.38	0.7305	0.2448	86.54
Ramphastidae	*Selenidera spectabilis*	Neotropics	0	-0.1960	-	3.86	0.8477	0.5496	42.35
Ramphastidae	*Semnornis frantzii*	Neotropics	0	-0.0152	3.2790	3.97	0.8342	0.6720	6.15
Ramphastidae	*Semnornis ramphastinus*	Neotropics	0	-0.5127	7.5275	2.83	0.7611	0.3332	31.92
Ramphastidae	*Stactolaema anchietae*	Neotropics	0	-0.2073	-	3.86	0.8377	0.6195	41.27
Ramphastidae	*Stactolaema leucotis*	IndoMalay	0	-0.0649	-	2.82	0.7528	0.2711	44.85
Ramphastidae	*Stactolaema olivacea*	Nearctic	0	-0.8540	-	4.08	0.8397	0.3320	6.16
Ramphastidae	*Stactolaema whytii*	Nearctic	0	-0.6786	12.8365	4.16	0.8436	0.5239	3.56
Ramphastidae	*Trachyphonus darnaudii*	Neotropics	0	-0.2935	8.2804	3.04	0.7961	0.6714	21.70
Ramphastidae	*Trachyphonus erythrocephalus*	Neotropics	0	0.0775	-	4.06	0.8219	0.6209	13.31
Ramphastidae	*Trachyphonus margaritatus*	Neotropics	0	-0.0891	2.4052	4.10	0.7809	0.3642	45.94
Ramphastidae	*Trachyphonus purpuratus*	Neotropics	0	0.2597	4.5956	3.91	0.7461	0.7133	11.89
Ramphastidae	*Trachyphonus usambiro*	Neotropics	0	-0.5385	9.0322	3.50	0.7281	0.2508	68.60
Ramphastidae	*Trachyphonus vaillantii*	Neotropics	0	-0.2227	-	3.86	0.8196	0.6995	22.64
Ramphastidae	*Tricholaema diademata*	Afrotropics	0	-0.0827	14.0309	3.66	0.8418	0.5383	74.44
Ramphastidae	*Tricholaema frontata*	Afrotropics	0	0.2947	-	3.16	0.7749	0.2174	6.54
Ramphastidae	*Tricholaema hirsuta*	Afrotropics	0	0.0392	9.7809	3.29	0.8117	0.5197	1.42
Ramphastidae	*Tricholaema lacrymosa*	Neotropics	1	-40.8005	8.8481	3.64	0.6221	0.1292	71.16
Ramphastidae	*Tricholaema leucomelas*	Neotropics	1	-2.6452	0.0000	3.97	0.5788	0.0000	90.00
Ramphastidae	*Tricholaema melanocephala*	Neotropics	0	0.0047	2.0947	4.05	0.7642	0.8580	14.23
Recurvirostridae	*Cladorhynchus leucocephalus*	Afrotropics	0	0.4809	-	3.77	0.7893	0.6811	23.06
Recurvirostridae	*Himantopus himantopus*	Neotropics	1	-0.2242	2.8315	3.35	0.7425	0.5440	44.26
Recurvirostridae	*Himantopus leucocephalus*	Neotropics	1	-0.1595	0.0569	3.00	0.5788	0.0000	90.00
Recurvirostridae	*Himantopus mexicanus*	Neotropics	0	0.2783	6.8417	4.30	0.7444	0.6750	45.45
Recurvirostridae	*Himantopus novaezelandiae*	Neotropics	0	-0.1617	-	3.71	0.8191	0.5630	38.64
Recurvirostridae	*Recurvirostra americana*	IndoMalay	0	-0.0882	-	3.04	0.7879	0.5305	41.81
Recurvirostridae	*Recurvirostra andina*	IndoMalay	0	-0.0116	-	2.83	0.7871	0.4770	4.72
Recurvirostridae	*Recurvirostra avosetta*	IndoMalay	0	-0.2979	-	3.46	0.8229	0.5929	9.71
Recurvirostridae	*Recurvirostra novaehollandiae*	Afrotropics	0	-0.0683	-	2.99	0.7251	0.4194	21.34
Reguliidae	*Regulus calendula*	IndoMalay	0	0.0094	-	2.85	0.7876	0.5328	13.98
Reguliidae	*Regulus goodfellowi*	IndoMalay	0	-0.0158	-	2.83	0.7884	0.4736	5.39
Reguliidae	*Regulus ignicapilla*	IndoMalay	0	0.0192	-	2.85	0.7876	0.5328	13.98
Reguliidae	*Regulus regulus*	IndoMalay	0	-0.0479	-	3.04	0.7919	0.6209	67.13
Reguliidae	*Regulus satrapa*	IndoMalay	0	-0.0589	-	3.10	0.7881	0.2441	45.91
Remizidae	*Anthoscopus caroli*	Afrotropics	0	-0.0929	10.3163	3.61	0.8280	0.8953	56.50
Remizidae	*Anthoscopus flavifrons*	Afrotropics	0	0.0104	2.8473	3.20	0.7901	0.2681	8.10
Remizidae	*Anthoscopus minutus*	Afrotropics	0	-0.3100	-	4.04	0.8065	0.8016	56.15
Remizidae	*Anthoscopus musculus*	Afrotropics	0	0.0748	-	3.35	0.7897	0.6320	36.98
Remizidae	*Anthoscopus parvulus*	Afrotropics	0	-0.0304	-	3.53	0.8039	0.1243	10.92
Remizidae	*Anthoscopus punctifrons*	Afrotropics	0	0.0014	-	3.74	0.8100	0.1377	3.17
Remizidae	*Auriparus flaviceps*	Neotropics	0	0.0782	8.7107	3.29	0.7461	0.2000	57.36
Remizidae	*Cephalopyrus flammiceps*	Australasia	0	-0.1134	1.7386	2.77	0.7715	0.3006	21.69
Remizidae	*Pholidornis rushiae*	Nearctic	0	-0.7382	2.8748	4.18	0.8242	0.7387	47.14
Remizidae	*Remiz consobrinus*	IndoMalay	0	0.0402	-	2.76	0.7217	0.1029	54.04
Remizidae	*Remiz coronatus*	Palearctic	0	-0.2357	0.0000	4.04	0.8005	0.2970	6.86
Remizidae	*Remiz macronyx*	IndoMalay	0	-0.0367	-	3.26	0.7898	0.7456	61.98
Remizidae	*Remiz pendulinus*	IndoMalay	0	-0.0849	-	3.09	0.8186	0.6047	47.38
Rhabdornithidae	*Rhabdornis grandis*	IndoMalay	0	0.0640	3.4303	2.84	0.7868	0.4434	3.13
Rhabdornithidae	*Rhabdornis inornatus*	IndoMalay	0	0.6963	2.8940	2.76	0.6948	0.1046	1.61
Rhabdornithidae	*Rhabdornis mystacalis*	Afrotropics	0	-0.2715	-	4.11	0.8045	0.5870	47.41
Rheidae	*Rhea americana*	IndoMalay	0	-0.0538	-	2.83	0.7677	0.6117	9.46
Rheidae	*Rhea pennata*	IndoMalay	0	-0.1560	-	3.30	0.7903	0.5544	26.74
Rhinocryptidae	*Acropternis orthonyx*	Neotropics	0	0.0675	11.3003	3.38	0.7331	0.1716	57.30
Rhinocryptidae	*Eleoscytalopus indigoticus*	Neotropics	0	-0.1595	-	3.84	0.8382	0.5456	47.09
Rhinocryptidae	*Eleoscytalopus psychopompus*	Neotropics	0	0.0063	-	3.93	0.8287	0.5456	42.73
Rhinocryptidae	*Eugralla paradoxa*	Afrotropics	0	-0.2312	-	3.53	0.7932	0.5060	29.52
Rhinocryptidae	*Liosceles thoracicus*	Neotropics	0	-0.1288	1.9364	3.04	0.7318	-	-
Rhinocryptidae	*Liosceles thoracicus*	Neotropics	0	-0.1288	1.9364	3.04	0.7318	0.2888	74.46
Rhinocryptidae	*Melanopareia elegans*	Neotropics	0	0.5373	5.5164	3.01	0.6906	0.1615	65.90
Rhinocryptidae	*Melanopareia maranonica*	Neotropics	0	0.0479	2.5003	3.45	0.7136	0.1362	27.72
Rhinocryptidae	*Melanopareia maximiliani*	Neotropics	0	0.0382	5.0120	3.61	0.7940	0.3522	37.46
Rhinocryptidae	*Melanopareia torquata*	Neotropics	0	13.5993	9.9302	3.78	0.7050	0.1242	89.00
Rhinocryptidae	*Merulaxis ater*	Australasia	0	-0.0736	-	2.80	0.7517	0.1364	23.47
Rhinocryptidae	*Merulaxis stresemanni*	Australasia	0	-0.1116	-	2.78	0.7705	0.3099	23.72
Rhinocryptidae	*Myornis senilis*	Neotropics	0	-0.0352	6.8913	3.72	0.7723	0.1775	32.94
Rhinocryptidae	*Psilorhamphus guttatus*	Afrotropics	1	-0.2390	3.6753	3.32	0.6935	0.2818	80.52
Rhinocryptidae	*Pteroptochos castaneus*	Neotropics	0	-0.1560	-	4.00	0.8270	0.5966	1.72
Rhinocryptidae	*Pteroptochos megapodius*	Neotropics	0	-0.0508	-	3.32	0.7666	0.2630	36.89
Rhinocryptidae	*Pteroptochos tarnii*	Neotropics	1	-0.8879	3.5671	2.89	0.6120	0.0000	0.00
Rhinocryptidae	*Rhinocrypta lanceolata*	IndoMalay	1	0.0484	3.8169	2.83	0.7684	0.4224	1.35
Rhinocryptidae	*Scelorchilus albicollis*	Australasia	0	-0.0705	14.6820	2.82	0.7378	0.1699	23.62

Family	Species	Realm	Threat	Latitude	Elevation	Anomaly	Size	Shape	Orientation
Rhinocryptidae	*Scelorchilus rubecula*	Australasia	0	-0.0566	3.2389	2.48	0.6120	0.0400	36.10
Rhinocryptidae	*Scytalopus acutirostris*	IndoMalay	0	-0.0030	-	3.13	0.7403	0.1763	60.80
Rhinocryptidae	*Scytalopus affinis*	Palearctic	0	-0.7203	-	3.94	0.8015	0.2440	1.95
Rhinocryptidae	*Scytalopus altirostris*	Neotropics	1	15.2567	1.2155	3.37	0.6370	0.4115	89.69
Rhinocryptidae	*Scytalopus argentifrons*	IndoMalay	1	-0.1152	-	2.66	0.6859	0.4403	76.79
Rhinocryptidae	*Scytalopus atratus*	Palearctic	0	-0.7861	11.3146	3.56	0.8292	0.4985	6.02
Rhinocryptidae	*Scytalopus bolivianus*	Neotropics	0	-0.1039	5.1538	3.38	0.7704	0.3706	28.34
Rhinocryptidae	*Scytalopus canus*	Neotropics	0	0.0538	8.1024	3.68	0.7524	0.3005	65.47
Rhinocryptidae	*Scytalopus caracae*	Neotropics	0	-0.1018	2.6206	3.95	0.8324	0.6689	18.68
Rhinocryptidae	*Scytalopus chocoensis*	Neotropics	0	-0.0454	2.7497	2.98	0.7151	0.2044	67.63
Rhinocryptidae	*Scytalopus diamantinensis*	Nearctic	0	9.3935	8.1603	4.26	0.7712	0.6836	49.28
Rhinocryptidae	*Scytalopus femoralis*	Nearctic	1	4.7157	4.3860	4.02	0.6302	0.1042	62.59
Rhinocryptidae	*Scytalopus fuscus*	Nearctic	1	0.0000	9.2813	3.84	0.5788	0.0000	0.00
Rhinocryptidae	*Scytalopus griseicollis*	Neotropics	0	0.0081	-	3.11	0.7335	0.2635	50.92
Rhinocryptidae	*Scytalopus iraiensis*	Neotropics	0	-0.6461	3.5769	3.25	0.6891	0.3945	74.80
Rhinocryptidae	*Scytalopus latebricola*	Neotropics	0	-0.6452	2.6839	3.17	0.7723	0.5065	86.87
Rhinocryptidae	*Scytalopus latrans*	Afrotropics	0	-0.4183	-	3.49	0.6824	0.1340	12.39
Rhinocryptidae	*Scytalopus macropus*	Afrotropics	0	0.1428	0.0000	3.70	0.7158	0.5339	8.11
Rhinocryptidae	*Scytalopus meridanus*	Neotropics	0	3.1100	11.1090	3.91	0.7251	0.3506	79.67
Rhinocryptidae	*Scytalopus micropterus*	Neotropics	0	-0.3700	8.4636	3.45	0.8311	0.5435	49.68
Rhinocryptidae	*Scytalopus novacapitalis*	Australasia	1	-0.3175	5.3985	2.58	0.6221	0.1501	33.56
Rhinocryptidae	*Scytalopus opaca*	Neotropics	0	-0.0600	-	3.33	0.7663	0.2630	37.28
Rhinocryptidae	*Scytalopus pachecoi*	Neotropics	0	0.1429	-	4.09	0.7769	0.4537	53.11
Rhinocryptidae	*Scytalopus panamensis*	Neotropics	0	-0.1877	-	3.99	0.8385	0.6553	19.77
Rhinocryptidae	*Scytalopus parkeri*	Australasia	0	-0.2234	2.1342	2.50	0.5788	0.0000	45.57
Rhinocryptidae	*Scytalopus parvirostris*	IndoMalay	0	0.0591	9.4203	2.79	0.7096	0.0869	54.46
Rhinocryptidae	*Scytalopus robbinsi*	IndoMalay	0	0.1443	9.0052	3.57	0.7452	0.5108	4.14
Rhinocryptidae	*Scytalopus rodriguezi*	IndoMalay	1	0.6630	4.5171	3.14	0.6120	0.9259	90.00
Rhinocryptidae	*Scytalopus sanctaemartae*	Afrotropics	0	-0.1411	-	3.73	0.8321	0.5947	70.38
Rhinocryptidae	*Scytalopus schulenbergi*	Afrotropics	0	-0.0471	-	3.28	0.7286	0.1899	58.44
Rhinocryptidae	*Scytalopus simonsi*	Afrotropics	0	-0.2173	16.8784	3.57	0.8508	0.4616	24.67
Rhinocryptidae	*Scytalopus speluncae*	Palearctic	0	-0.8487	11.3852	4.61	0.8808	0.1001	1.27
Rhinocryptidae	*Scytalopus spillmanni*	IndoMalay	1	-0.0324	2.5143	2.64	0.6562	0.4695	60.26
Rhinocryptidae	*Scytalopus stilesi*	IndoMalay	1	-0.1141	1.8667	2.64	0.6221	0.1467	25.68
Rhinocryptidae	*Scytalopus superciliaris*	Neotropics	1	-0.4203	8.2770	4.17	0.7087	0.3456	64.64
Rhinocryptidae	*Scytalopus unicolor*	Neotropics	0	-0.4244	6.1089	2.98	0.8266	0.5873	87.02
Rhinocryptidae	*Scytalopus urubambae*	IndoMalay	0	0.0199	-	2.84	0.7893	0.5111	9.27
Rhinocryptidae	*Scytalopus vicinior*	Neotropics	0	0.2453	8.3486	4.35	0.7112	0.3615	22.80
Rhinocryptidae	*Scytalopus zimmeri*	Australasia	1	-0.5699	0.0000	3.06	0.7909	0.7784	21.63
Rhinocryptidae	*Teledromas fuscus*	Neotropics	0	0.0168	-	3.17	0.7621	0.4646	59.43
Rhipiduridae	*Rhipidura albicollis*	Neotropics	0	-0.0284	4.4607	3.56	0.7449	0.6250	32.81
Rhipiduridae	*Rhipidura albolimbata*	Neotropics	0	0.0373	-	3.99	0.7792	0.4946	16.50
Rhipiduridae	*Rhipidura atra*	Neotropics	0	-0.2740	7.7955	3.56	0.8397	0.2385	49.97
Rhipiduridae	*Rhipidura aureola*	Neotropics	0	-0.1794	10.3763	3.42	0.8069	0.4099	46.49
Rhipiduridae	*Rhipidura brachyrhyncha*	Neotropics	0	-0.5465	-	2.89	0.7683	0.3563	26.43
Rhipiduridae	*Rhipidura cockerelli*	Palearctic	0	-0.8428	18.6136	4.18	0.8249	0.1741	1.92
Rhipiduridae	*Rhipidura cyaniceps*	Palearctic	0	-0.6283	-	4.21	0.8557	0.2290	2.98
Rhipiduridae	*Rhipidura dahli*	Australasia	0	-0.6857	-	3.33	0.8167	0.4114	3.91
Rhipiduridae	*Rhipidura dedemi*	Neotropics	0	-0.2576	13.1104	3.68	0.7792	0.3885	75.87
Rhipiduridae	*Rhipidura diluta*	Palearctic	0	-19.8457	13.1032	3.79	0.7510	0.1976	4.70
Rhipiduridae	*Rhipidura drownei*	IndoMalay	0	-4.4310	7.1267	4.42	0.7310	0.2032	34.10
Rhipiduridae	*Rhipidura euryura*	Palearctic	0	-0.8412	11.1958	3.72	0.8068	0.4862	22.40
Rhipiduridae	*Rhipidura fuliginosa*	IndoMalay	0	0.0047	8.6543	4.14	0.7490	0.1588	18.05
Rhipiduridae	*Rhipidura fuscorufa*	IndoMalay	0	-0.0187	5.8499	2.64	0.6921	0.1543	65.29
Rhipiduridae	*Rhipidura hyperythra*	IndoMalay	0	-0.1338	10.2669	3.46	0.8095	0.3630	7.44
Rhipiduridae	*Rhipidura hypoxantha*	Palearctic	0	-0.4195	7.5814	4.71	0.8626	0.1583	1.02
Rhipiduridae	*Rhipidura javanica*	Neotropics	1	1.7383	5.9330	3.41	0.6478	0.5506	65.68
Rhipiduridae	*Rhipidura leucophrys*	Neotropics	1	0.3017	9.0244	3.68	0.7019	0.3562	61.23
Rhipiduridae	*Rhipidura leucothorax*	Neotropics	1	-0.1327	3.4898	3.11	0.7363	0.3043	69.38
Rhipiduridae	*Rhipidura maculipectus*	Neotropics	0	-0.3419	-	3.81	0.7261	0.4338	33.73
Rhipiduridae	*Rhipidura nigrocinnamomea*	Neotropics	0	-0.0491	6.0104	4.34	0.6948	0.6587	4.19
Rhipiduridae	*Rhipidura opistherythra*	Neotropics	0	-0.3212	5.4458	3.21	0.7977	0.3409	42.95
Rhipiduridae	*Rhipidura perlata*	Neotropics	1	-0.3222	3.1601	3.38	0.6370	0.3279	15.11
Rhipiduridae	*Rhipidura phasiana*	Neotropics	0	-0.6796	6.6364	3.84	0.6428	0.1722	15.30
Rhipiduridae	*Rhipidura phoenicura*	Neotropics	0	0.0332	9.8159	2.99	0.6662	0.3023	30.73
Rhipiduridae	*Rhipidura rufidorsa*	Neotropics	0	-0.0193	-	3.62	0.7639	0.7023	26.80
Rhipiduridae	*Rhipidura rufifrons*	Neotropics	0	0.0053	6.1750	3.27	0.7500	0.2300	49.37
Rhipiduridae	*Rhipidura rufiventris*	Neotropics	0	0.4698	1.5059	3.82	0.7965	0.5552	24.21
Rhipiduridae	*Rhipidura superciliaris*	Neotropics	0	-0.1749	8.3688	3.93	0.7730	0.8265	35.64
Rhipiduridae	*Rhipidura superflua*	Neotropics	1	-1.9072	2.3249	3.25	0.6120	1.0000	0.00
Rhipiduridae	*Rhipidura tenebrosa*	Neotropics	0	-0.0147	-	4.25	0.7859	0.5917	60.66
Rhipiduridae	*Rhipidura teysmanni*	Neotropics	1	-4.1626	3.5338	3.97	0.6662	0.3160	89.00
Rhipiduridae	*Rhipidura threnothorax*	Neotropics	0	0.0635	6.6293	4.10	0.8228	0.7127	20.62
Rhipiduridae	*Rhipidura verreauxi*	Neotropics	0	0.4322	11.8339	4.12	0.6523	0.3358	36.62

Family	Species	Realm	Threat	Latitude	Elevation	Anomaly	Size	Shape	Orientation
Rhynochetidae	*Rhynochetos jubatus*	Nearctic	0	-0.4527	11.7270	4.22	0.8341	0.6360	8.94
Rostratulidae	*Rostratula australis*	Neotropics	0	0.0315	-	3.10	0.7480	0.4281	69.21
Rostratulidae	*Rostratula benghalensis*	Neotropics	0	-13.0591	6.6565	3.92	0.7128	0.2381	65.15
Rostratulidae	*Rostratula semicollaris*	Neotropics	0	0.0894	-	4.05	0.8046	0.4548	2.49
Sapayoaidae	*Sapayoa aenigma*	IndoMalay	0	-0.0501	-	2.82	0.7890	0.5930	11.00
Scolopacidae	*Actitis hypoleucos*	Palearctic	0	-0.9744	-	4.74	0.8717	0.1976	3.76
Scolopacidae	*Actitis macularius*	Nearctic	0	-0.7797	6.2668	4.30	0.8547	0.3169	11.89
Scolopacidae	*Aphriza virgata*	Nearctic	0	-0.8704	5.5494	4.10	0.7809	-	-
Scolopacidae	*Aphriza virgata*	Nearctic	0	-0.8704	5.5494	4.10	0.7809	0.2917	0.44
Scolopacidae	*Arenaria interpres*	Australasia	0	-0.4051	-	3.39	0.8349	0.6089	2.08
Scolopacidae	*Arenaria melanocephala*	Afrotropics	0	-0.1123	-	3.86	0.8193	0.2982	58.09
Scolopacidae	*Bartramia longicauda*	Neotropics	1	0.0000	3.6259	3.05	0.6120	0.3926	86.44
Scolopacidae	*Calidris acuminata*	Palearctic	0	-0.9696	8.4671	4.17	0.8557	0.1632	2.73
Scolopacidae	*Calidris alba*	Palearctic	0	-0.8812	-	4.16	0.8326	0.3947	13.25
Scolopacidae	*Calidris alpina*	Afrotropics	0	-0.1090	15.3929	3.79	0.8339	0.5108	65.98
Scolopacidae	*Calidris bairdii*	IndoMalay	0	-0.1160	-	3.54	0.7898	0.2779	14.49
Scolopacidae	*Calidris canutus*	Palearctic	0	-0.8560	-	4.14	0.8385	0.2475	7.11
Scolopacidae	*Calidris ferruginea*	Afrotropics	0	3.3170	6.5609	3.47	0.7626	0.5537	61.35
Scolopacidae	*Calidris fuscicollis*	Nearctic	0	-0.5136	-	5.68	0.8438	0.0457	0.54
Scolopacidae	*Calidris himantopus*	Nearctic	0	2.6365	8.3115	4.38	0.7781	0.4521	14.44
Scolopacidae	*Calidris maritima*	Nearctic	0	-0.4088	2.6305	5.02	0.7836	0.1641	7.30
Scolopacidae	*Calidris mauri*	Afrotropics	0	-0.1224	-	3.13	0.7500	0.4691	69.63
Scolopacidae	*Calidris melanotos*	Afrotropics	1	0.1767	0.4110	3.18	0.6478	0.4801	66.65
Scolopacidae	*Calidris minuta*	Palearctic	0	-0.4249	-	6.24	0.7554	0.0641	4.35
Scolopacidae	*Calidris minutilla*	Nearctic	0	-0.3204	0.4325	6.22	0.7932	0.0522	0.63
Scolopacidae	*Calidris ptilocnemis*	Palearctic	0	-0.8981	-	5.78	0.8331	0.0516	0.73
Scolopacidae	*Calidris pusilla*	Nearctic	0	-1.0621	10.3126	5.58	0.8149	0.0841	1.43
Scolopacidae	*Calidris ruficollis*	Nearctic	0	-0.3984	0.7113	6.12	0.8023	0.0440	0.98
Scolopacidae	*Calidris subminuta*	Palearctic	0	-0.2999	-	6.78	0.7913	0.0694	1.66
Scolopacidae	*Calidris temminckii*	Nearctic	0	-0.6450	-	6.06	0.7948	0.1401	2.30
Scolopacidae	*Calidris tenuirostris*	Nearctic	0	-0.5232	4.4952	5.74	0.7632	0.1606	8.97
Scolopacidae	*Catoptrophorus semipalmatus*	Palearctic	0	-0.7843	-	3.95	0.6859	0.1037	6.42
Scolopacidae	*Eurynorhynchus pygmeus*	Afrotropics	0	0.1339	-	3.37	0.7761	0.4881	71.10
Scolopacidae	*Gallinago andina*	Neotropics	0	0.9639	-	3.73	0.7623	0.8580	29.90
Scolopacidae	*Gallinago gallinago*	Neotropics	0	0.1099	-	4.18	0.7761	0.7891	18.14
Scolopacidae	*Gallinago hardwickii*	Neotropics	0	0.1658	-	3.94	0.8136	0.3903	14.43
Scolopacidae	*Gallinago imperialis*	Neotropics	0	0.0163	-	3.83	0.7767	0.6881	66.24
Scolopacidae	*Gallinago jamesoni*	Neotropics	0	0.1833	-	4.13	0.7873	0.6586	6.35
Scolopacidae	*Gallinago macrodactyla*	Neotropics	0	0.0096	-	4.10	0.8113	0.3439	2.94
Scolopacidae	*Gallinago media*	Neotropics	0	-0.0692	-	4.06	0.8322	0.7560	2.63
Scolopacidae	*Gallinago megala*	Neotropics	0	-0.0435	-	4.08	0.8005	0.5519	19.40
Scolopacidae	*Gallinago nemoricola*	Neotropics	0	-0.0769	-	4.14	0.8152	0.7187	80.66
Scolopacidae	*Gallinago nigripennis*	Neotropics	1	-5.1917	5.7593	3.33	0.6876	0.1741	72.25
Scolopacidae	*Gallinago nobilis*	Neotropics	0	-0.1031	-	3.84	0.8398	0.4306	35.04
Scolopacidae	*Gallinago paraguaiae*	Neotropics	0	0.1334	-	3.88	0.7829	0.3278	20.26
Scolopacidae	*Gallinago solitaria*	Palearctic	0	-0.5460	9.4691	4.06	0.8746	0.3061	7.78
Scolopacidae	*Gallinago stenura*	IndoMalay	0	-0.2033	-	3.48	0.7747	0.9491	64.22
Scolopacidae	*Gallinago stricklandii*	Afrotropics	0	-0.4295	-	3.61	0.7697	0.5538	13.34
Scolopacidae	*Gallinago undulata*	Palearctic	0	-0.3521	-	3.59	0.8044	0.2429	20.55
Scolopacidae	*Heteroscelus brevipes*	Neotropics	0	-0.1395	-	3.84	0.8539	0.4196	34.99
Scolopacidae	*Heteroscelus incanus*	Neotropics	0	-0.2496	-	3.77	0.8198	0.4210	35.02
Scolopacidae	*Limicola falcinellus*	Neotropics	0	-0.2071	-	3.73	0.8578	0.4638	40.47
Scolopacidae	*Limnodromus griseus*	Neotropics	0	0.0686	11.6993	3.62	0.7500	0.3672	77.26
Scolopacidae	*Limnodromus scolopaceus*	Neotropics	0	-1.5560	8.2403	3.68	0.7464	0.3781	86.14
Scolopacidae	*Limnodromus semipalmatus*	Neotropics	0	-1.1408	7.3743	4.06	0.7787	0.3991	62.60
Scolopacidae	*Limosa fedoa*	Neotropics	0	0.0907	-	3.94	0.7464	0.3480	0.02
Scolopacidae	*Limosa haemastica*	Neotropics	0	0.2458	1.4369	3.50	0.7050	0.2218	5.05
Scolopacidae	*Limosa lapponica*	Neotropics	0	-0.2906	11.4790	3.54	0.7112	0.3495	72.54
Scolopacidae	*Limosa limosa*	Neotropics	0	-0.6013	-	3.13	0.8037	0.5458	46.41
Scolopacidae	*Lymnocryptes minimus*	IndoMalay	1	-0.4293	0.7266	2.76	0.5985	0.5754	48.33
Scolopacidae	*Numenius americanus*	Afrotropics	0	0.0195	-	3.29	0.8222	0.2715	17.58
Scolopacidae	*Numenius arquata*	Afrotropics	0	-0.1259	-	3.40	0.7874	0.3417	71.15
Scolopacidae	*Numenius borealis*	Afrotropics	0	-0.0226	-	3.30	0.7963	0.6678	3.75
Scolopacidae	*Numenius madagascariensis*	Afrotropics	0	-0.0302	-	3.27	0.8175	0.2583	15.57
Scolopacidae	*Numenius minutus*	Afrotropics	0	0.0053	13.3725	3.28	0.8219	0.3258	13.59
Scolopacidae	*Numenius phaeopus*	Afrotropics	0	0.0088	6.2017	3.29	0.8151	0.3600	15.31
Scolopacidae	*Numenius tahitiensis*	Afrotropics	0	0.0085	-	3.28	0.8110	0.4312	11.87
Scolopacidae	*Numenius tenuirostris*	Afrotropics	0	-0.0606	-	3.62	0.8511	0.7197	39.69
Scolopacidae	*Philomachus pugnax*	Neotropics	0	0.1465	2.7883	3.12	0.7449	0.3021	53.34
Scolopacidae	*Scolopax bukidnonensis*	IndoMalay	0	0.0092	2.5775	2.84	0.7803	0.6177	17.94
Scolopacidae	*Scolopax rochussenii*	Neotropics	0	-0.1101	-	3.43	0.7352	0.1569	19.59
Scolopacidae	*Scolopax rusticola*	Palearctic	0	-0.8504	0.0000	4.20	0.8273	0.1762	8.06
Scolopacidae	*Scolopax saturata*	Palearctic	0	-0.9885	6.5300	4.19	0.8049	0.1738	5.45
Scolopacidae	*Steganopus tricolor*	IndoMalay	0	-0.0668	-	3.76	0.7651	0.2346	14.87

Family	Species	Realm	Threat	Latitude	Elevation	Anomaly	Size	Shape	Orientation
Scolopacidae	*Tringa erythropus*	Neotropics	0	0.1014	-	3.93	0.8207	0.4594	4.36
Scolopacidae	*Tringa flavipes*	Neotropics	0	-88.0117	8.8459	4.15	0.6631	0.8501	50.06
Scolopacidae	*Tringa guttifer*	Neotropics	0	0.2719	9.4796	3.25	0.7019	0.2082	76.04
Scolopacidae	*Tringa melanoleuca*	Neotropics	0	0.1095	-	3.25	0.7422	0.6256	9.27
Scolopacidae	*Tringa nebularia*	Neotropics	0	-0.2014	-	3.74	0.7498	0.1839	46.90
Scolopacidae	*Tringa ochropus*	Neotropics	0	-0.1708	-	3.95	0.8326	0.4377	1.37
Scolopacidae	*Tringa solitaria*	Neotropics	0	0.0171	-	4.11	0.7128	0.3235	30.13
Scolopacidae	*Tringa stagnatilis*	Neotropics	0	-0.0929	-	4.04	0.7664	0.4380	34.43
Scolopacidae	*Tringa totanus*	Neotropics	0	0.0510	-	3.05	0.7272	0.3077	72.49
Scolopacidae	*Tryngites subruficollis*	Neotropics	0	0.0576	8.1212	4.13	0.7711	0.3465	54.48
Scolopacidae	*Xenus cinereus*	IndoMalay	0	-0.3738	6.0334	3.13	0.8258	0.3895	36.30
Scopidae	*Scopus umbretta*	Afrotropics	0	-0.1345	6.9844	3.37	0.8015	0.6837	81.58
Sittidae	*Sitta azurea*	Neotropics	0	-12.1933	6.5853	3.82	0.6859	0.2149	64.67
Sittidae	*Sitta canadensis*	Neotropics	0	-0.8329	10.9603	2.17	0.7614	0.2330	89.81
Sittidae	*Sitta carolinensis*	Neotropics	0	-0.0126	11.5331	4.10	0.6690	0.3712	32.19
Sittidae	*Sitta cashmirensis*	Neotropics	0	4.4216	6.0345	3.41	0.7151	0.1518	57.69
Sittidae	*Sitta castanea*	Neotropics	0	-2.1796	1.2462	3.86	0.7172	0.4420	76.47
Sittidae	*Sitta europaea*	Neotropics	0	0.2145	8.2559	3.19	0.7192	0.1523	65.98
Sittidae	*Sitta formosa*	Neotropics	0	-0.5346	1.6807	2.75	0.7030	0.5454	61.78
Sittidae	*Sitta himalayensis*	Neotropics	0	-14.3769	6.5585	3.38	0.6428	0.2590	58.87
Sittidae	*Sitta krueperi*	Neotropics	0	-2.9687	8.6690	3.97	0.7344	0.1641	39.36
Sittidae	*Sitta ledanti*	Neotropics	1	0.6594	3.1201	3.21	0.6120	0.4082	71.57
Sittidae	*Sitta leucopsis*	Neotropics	1	0.0000	1.8680	3.28	0.6120	1.0000	90.00
Sittidae	*Sitta magna*	Neotropics	0	0.7410	4.6036	3.19	0.6221	0.5865	19.14
Sittidae	*Sitta nagaensis*	Neotropics	0	1.2255	3.8113	4.06	0.7060	0.1280	35.42
Sittidae	*Sitta neumayer*	Neotropics	0	7.7095	6.3032	4.05	0.7008	0.1369	34.88
Sittidae	*Sitta oenochlamys*	Neotropics	0	-0.2882	10.0426	2.83	0.7572	0.1880	43.91
Sittidae	*Sitta pusilla*	Neotropics	0	9.0717	10.2053	3.16	0.7144	0.1775	66.45
Sittidae	*Sitta pygmaea*	Neotropics	0	0.3705	4.2381	3.18	0.6784	0.2684	82.23
Sittidae	*Sitta solangiae*	Neotropics	0	8.9258	10.3980	3.70	0.7008	0.1520	73.84
Sittidae	*Sitta tephronota*	Neotropics	0	-22.4742	6.8804	3.53	0.6428	0.4658	54.92
Sittidae	*Sitta victoriae*	Neotropics	0	-84.3697	4.3888	4.16	0.6478	0.4787	37.03
Sittidae	*Sitta villosa*	Neotropics	0	1.1477	8.7442	2.99	0.6876	0.1244	61.69
Sittidae	*Sitta whiteheadi*	Neotropics	0	-12.5982	9.0773	4.02	0.6716	0.3093	80.66
Sittidae	*Sitta yunnanensis*	Australasia	0	-0.3285	-	3.29	0.8033	0.3017	34.94
Sittidae	*Tichodroma muraria*	Afrotropics	0	0.0285	8.6293	3.44	0.8229	0.5328	24.27
Steatornithidae	*Steatornis caripensis*	IndoMalay	0	-0.0903	-	3.12	0.8300	0.5435	35.37
Strigidae	*Aegolius acadicus*	Nearctic	0	-0.6817	6.8117	4.10	0.8351	0.4377	5.90
Strigidae	*Aegolius funereus*	Palearctic	0	-0.9570	-	4.86	0.8613	0.1578	0.82
Strigidae	*Aegolius harrisii*	Neotropics	0	-0.3603	8.3957	2.99	0.7790	0.4843	37.71
Strigidae	*Aegolius ridgwayi*	Neotropics	0	-0.0315	8.9683	3.06	0.6302	0.2782	40.02
Strigidae	*Asio abyssinicus*	Afrotropics	0	0.8218	8.6242	3.33	0.7165	0.2315	69.18
Strigidae	*Asio capensis*	Afrotropics	1	0.0698	2.3964	3.25	0.5788	0.0000	65.24
Strigidae	*Asio flammeus*	Neotropics	0	-0.1808	1.8608	3.74	0.8440	0.5955	50.65
Strigidae	*Asio madagascariensis*	Australasia	0	-0.5565	-	3.54	0.7371	0.5081	43.38
Strigidae	*Asio otus*	Afrotropics	0	1.7294	2.2448	3.50	0.7396	0.2846	54.14
Strigidae	*Asio stygius*	Afrotropics	0	-0.2471	6.9844	3.60	0.8388	0.7312	75.11
Strigidae	*Athene brama*	Australasia	0	-0.2456	7.3732	2.81	0.7096	0.2287	40.20
Strigidae	*Athene cunicularia*	Neotropics	0	-0.0100	1.9424	3.90	0.7794	0.4720	2.17
Strigidae	*Athene cunicularia*	Neotropics	0	-0.0100	1.9424	3.90	0.7794	-	-
Strigidae	*Athene noctua*	Afrotropics	0	-0.1342	6.5395	3.04	0.7069	0.1318	73.92
Strigidae	*Bubo africanus*	Neotropics	0	0.1801	-	3.92	0.8096	0.5767	73.53
Strigidae	*Bubo ascalaphus*	Neotropics	0	-0.0096	-	3.57	0.7778	0.2956	12.60
Strigidae	*Bubo bengalensis*	Neotropics	1	0.3849	3.4540	3.12	0.6804	0.3683	86.62
Strigidae	*Bubo bubo*	Neotropics	0	0.0427	-	4.06	0.7997	0.4135	18.52
Strigidae	*Bubo capensis*	Neotropics	0	-0.1817	-	2.97	0.7464	0.2581	46.93
Strigidae	*Bubo coromandus*	Neotropics	0	-0.2156	-	3.80	0.7787	0.3610	27.30
Strigidae	*Bubo lacteus*	Afrotropics	0	0.0603	-	3.61	0.8123	0.1440	5.76
Strigidae	*Bubo leucostictus*	Afrotropics	0	-0.0771	-	3.69	0.8169	0.2978	51.99
Strigidae	*Bubo nipalensis*	Afrotropics	0	-0.1521	8.5404	3.60	0.8631	0.8430	45.51
Strigidae	*Bubo philippensis*	Palearctic	0	-0.4700	-	4.04	0.8195	0.3508	3.74
Strigidae	*Bubo poensis*	IndoMalay	0	-0.2001	-	3.53	0.8196	0.7240	7.22
Strigidae	*Bubo scandiaca*	Palearctic	0	-0.6651	10.8239	4.50	0.8769	0.2960	0.96
Strigidae	*Bubo shelleyi*	Afrotropics	0	-0.1053	12.9209	3.62	0.8034	0.3490	64.81
Strigidae	*Bubo sumatranus*	IndoMalay	0	-0.1061	-	3.49	0.8004	0.4203	8.55
Strigidae	*Bubo virginianus*	Afrotropics	0	-0.1202	13.5054	3.61	0.8465	0.5278	64.04
Strigidae	*Bubo vosseleri*	Afrotropics	0	-0.0659	-	3.23	0.8100	0.2781	14.07
Strigidae	*Glaucidium albertinum*	Neotropics	0	-0.2343	10.5719	3.04	0.6804	0.2128	30.89
Strigidae	*Glaucidium bolivianum*	Neotropics	0	0.0349	10.7561	3.66	0.7700	0.2737	59.90
Strigidae	*Glaucidium brasilianum*	Neotropics	0	0.0841	4.9528	2.90	0.6562	0.3764	44.76
Strigidae	*Glaucidium brodiei*	Neotropics	0	-0.0833	5.0057	3.00	0.6784	0.1996	16.87
Strigidae	*Glaucidium capense*	Neotropics	0	-0.0597	-	3.69	0.7396	0.4252	36.17
Strigidae	*Glaucidium castaneum*	Neotropics	0	-0.1915	-	3.75	0.8558	0.5011	41.48
Strigidae	*Glaucidium castanonotum*	Neotropics	0	-0.3053	-	2.41	0.5788	0.0000	0.00

Family	Species	Realm	Threat	Latitude	Elevation	Anomaly	Size	Shape	Orientation
Strigidae	*Glaucidium castanopterum*	Neotropics	0	0.2896	2.8558	3.54	0.7512	0.4633	58.73
Strigidae	*Glaucidium costaricanum*	Neotropics	0	-0.0438	-	3.00	0.7240	0.5896	50.14
Strigidae	*Glaucidium cuculoides*	Neotropics	0	-0.6486	8.6532	2.47	0.5985	0.0000	0.00
Strigidae	*Glaucidium gnoma*	Neotropics	0	-0.1607	-	3.40	0.7976	0.3625	44.39
Strigidae	*Glaucidium griseiceps*	Neotropics	0	-0.3382	15.8069	3.13	0.8368	0.6954	58.82
Strigidae	*Glaucidium hardyi*	Neotropics	0	-0.1801	-	3.77	0.8556	0.4564	38.56
Strigidae	*Glaucidium jardinii*	Afrotropics	1	-0.2833	4.7749	3.47	0.7367	0.5735	64.78
Strigidae	*Glaucidium minutissimum*	Palearctic	1	-0.0705	6.3154	3.85	0.7277	0.1569	12.24
Strigidae	*Glaucidium mooreorum*	Australasia	0	0.0444	3.3882	2.96	0.7851	0.4421	33.50
Strigidae	*Glaucidium nanum*	Australasia	0	-0.1132	-	2.77	0.7676	0.3045	24.48
Strigidae	*Glaucidium nubicola*	Australasia	0	-0.0888	6.9481	2.82	0.7382	-	-
Strigidae	*Glaucidium nubicola*	Australasia	0	-0.0888	6.9481	2.82	0.7382	0.1520	23.84
Strigidae	*Glaucidium parkeri*	Australasia	0	-0.3792	-	2.57	0.6370	0.3880	82.91
Strigidae	*Glaucidium passerinum*	Australasia	0	-0.6063	-	3.40	0.8219	0.4083	4.55
Strigidae	*Glaucidium perlatum*	Australasia	0	-0.4587	-	2.33	0.7331	0.2569	44.63
Strigidae	*Glaucidium peruanum*	Australasia	0	-0.0622	-	2.56	0.6523	0.1875	30.24
Strigidae	*Glaucidium radiatum*	Australasia	0	-0.3812	-	3.08	0.7611	0.4628	24.38
Strigidae	*Glaucidium sanchezi*	Australasia	0	-0.5183	-	3.11	0.7550	0.3058	76.35
Strigidae	*Glaucidium siju*	Australasia	0	-0.4082	-	3.26	0.8066	0.5130	43.41
Strigidae	*Glaucidium sjostedti*	Australasia	0	-0.2637	-	2.95	0.7852	0.3180	62.02
Strigidae	*Glaucidium tephronotum*	Australasia	0	-0.0838	-	2.82	0.7352	0.1445	26.12
Strigidae	*Gymnoglaux lawrencii*	IndoMalay	0	-0.1527	-	3.18	0.7786	0.4449	6.42
Strigidae	*Heteroglaux blewitti*	Neotropics	0	-0.4969	2.8484	3.20	0.7697	0.4511	26.44
Strigidae	*Jubula lettii*	IndoMalay	0	0.0061	-	2.84	0.7882	0.5121	10.87
Strigidae	*Ketupa blakistoni*	Neotropics	0	-3.2793	6.1465	4.02	0.7300	0.1361	38.10
Strigidae	*Ketupa flavipes*	Neotropics	0	0.1460	4.1511	3.10	0.6891	0.1117	70.21
Strigidae	*Ketupa ketupu*	Neotropics	0	-4.3343	8.1775	3.91	0.7151	0.1678	55.74
Strigidae	*Ketupa zeylonensis*	Neotropics	0	0.0838	6.0175	3.29	0.7205	0.2131	63.98
Strigidae	*Lophostrix cristata*	Palearctic	0	-0.7015	-	4.52	0.8469	0.2017	2.13
Strigidae	*Megascops albogularis*	Neotropics	0	0.2580	1.5616	2.92	0.6221	0.4134	35.53
Strigidae	*Megascops asio*	Neotropics	0	-0.2478	9.8206	3.05	0.6740	0.1930	30.53
Strigidae	*Megascops atricapilla*	Neotropics	0	0.0796	12.5781	3.65	0.7727	0.4030	78.80
Strigidae	*Megascops barbarus*	Neotropics	0	0.0796	5.7122	3.03	0.6891	0.1214	67.06
Strigidae	*Megascops choliba*	Palearctic	1	-0.8273	-	4.05	0.8007	0.1890	2.05
Strigidae	*Megascops clarkii*	Neotropics	0	-0.0009	8.8890	3.36	0.7515	0.2313	60.51
Strigidae	*Megascops cooperi*	Australasia	0	-0.0612	-	2.79	0.7639	0.3683	23.78
Strigidae	*Megascops guatemalae*	IndoMalay	0	-0.0227	-	2.62	0.7078	0.6480	74.09
Strigidae	*Megascops hoyi*	Neotropics	0	0.0904	7.6016	3.34	0.7172	0.3340	76.46
Strigidae	*Megascops hoyi*	Neotropics	0	0.0904	7.6016	3.34	0.7172	-	-
Strigidae	*Megascops ingens*	Neotropics	0	5.3810	11.5283	3.94	0.7158	0.2747	63.99
Strigidae	*Megascops kennicottii*	Neotropics	0	-0.1592	16.0454	3.81	0.7891	0.5224	85.87
Strigidae	*Megascops koepckeae*	Neotropics	0	0.9586	6.3910	3.56	0.7256	0.2968	75.10
Strigidae	*Megascops marshalli*	Neotropics	0	0.0815	9.1855	3.54	0.7498	-	-
Strigidae	*Megascops marshalli*	Neotropics	0	0.0815	9.1855	3.54	0.7498	0.4001	86.88
Strigidae	*Megascops nudipes*	Neotropics	0	0.0228	9.7312	3.66	0.7604	0.4273	78.99
Strigidae	*Megascops petersoni*	Afrotropics	0	-0.0575	9.0206	3.29	0.8232	0.3504	14.98
Strigidae	*Megascops petersoni*	Afrotropics	0	-0.0575	9.0206	3.29	0.8232	-	-
Strigidae	*Megascops roboratus*	Nearctic	0	-0.7604	7.5193	4.21	0.8552	0.3261	13.93
Strigidae	*Megascops sanctaecatarinae*	Palearctic	0	-0.6817	-	3.46	0.8264	0.3851	12.55
Strigidae	*Megascops seductus*	Afrotropics	0	-0.1260	-	3.49	0.8541	0.5888	38.82
Strigidae	*Megascops trichopsis*	Neotropics	0	-0.2756	4.2357	3.59	0.8593	0.6779	61.70
Strigidae	*Megascops watsonii*	Australasia	0	-0.1070	-	2.84	0.7277	0.7410	49.93
Strigidae	*Micrathene whitneyi*	Neotropics	0	0.7858	9.3799	3.60	0.7030	0.5896	19.54
Strigidae	*Nesasio solomonensis*	Afrotropics	0	-0.0097	-	3.28	0.8057	0.3975	8.98
Strigidae	*Ninox connivens*	Afrotropics	0	-0.0965	-	3.55	0.7805	0.6808	10.84
Strigidae	*Ninox ios*	Afrotropics	0	-0.1284	4.3000	3.04	0.7245	0.1484	75.29
Strigidae	*Ninox jacquinoti*	Afrotropics	0	-0.1590	-	3.18	0.7495	0.3101	65.29
Strigidae	*Ninox novaeseelandiae*	Afrotropics	0	-0.0702	-	3.18	0.7614	0.3874	70.15
Strigidae	*Ninox ochracea*	Afrotropics	0	-0.1429	-	3.06	0.7318	0.1862	74.90
Strigidae	*Ninox odiosa*	Neotropics	0	-0.1492	-	3.88	0.8127	0.4540	20.79
Strigidae	*Ninox philippensis*	Neotropics	0	-0.0092	-	3.96	0.7848	0.7137	2.07
Strigidae	*Ninox punctulata*	Neotropics	1	0.0490	5.4745	3.01	0.6842	0.2048	59.70
Strigidae	*Ninox rudolfi*	Neotropics	0	0.1528	-	4.21	0.7672	0.5857	18.26
Strigidae	*Ninox rufa*	Neotropics	0	0.0072	-	4.06	0.7593	0.5003	17.58
Strigidae	*Ninox scutulata*	Neotropics	1	-0.0926	3.7412	3.18	0.7438	0.2833	73.42
Strigidae	*Ninox squamipila*	Neotropics	0	0.0374	-	3.98	0.7739	0.5646	15.30
Strigidae	*Ninox strenua*	Neotropics	0	-0.6305	3.5796	3.02	0.7245	0.3471	29.16
Strigidae	*Ninox sumbaensis*	Neotropics	0	-0.1520	-	3.85	0.8051	0.6452	40.56
Strigidae	*Ninox superciliaris*	Neotropics	0	-0.0272	-	4.12	0.7871	0.3500	35.71
Strigidae	*Ninox theomacha*	Australasia	1	-0.1415	0.0000	2.07	0.6120	0.3994	15.26
Strigidae	*Ninox variegata*	Australasia	0	-0.7187	-	3.09	0.7905	0.6437	52.63
Strigidae	*Otus alfredi*	Neotropics	0	-0.1305	15.3617	4.04	0.7760	0.2422	51.48
Strigidae	*Otus angelinae*	Neotropics	0	-0.7402	13.8854	3.26	0.7606	0.1225	71.72
Strigidae	*Otus bakkamoena*	Neotropics	0	-0.0474	4.3610	4.00	0.6948	0.5240	50.61

Family	Species	Realm	Threat	Latitude	Elevation	Anomaly	Size	Shape	Orientation
Strigidae	*Otus brookii*	Australasia	0	-0.7476	-	3.15	0.6948	0.6300	77.46
Strigidae	*Otus brucei*	Afrotropics	1	-0.1383	3.7065	3.01	0.6935	0.1246	71.95
Strigidae	*Otus flammeolus*	IndoMalay	0	-0.0979	-	2.66	0.6842	0.4928	80.15
Strigidae	*Otus fuliginosus*	Afrotropics	0	-0.1119	7.9869	3.64	0.8275	0.5224	42.94
Strigidae	*Otus icterorhynchus*	Australasia	0	-0.0749	-	2.49	0.5985	0.0448	40.11
Strigidae	*Otus ireneae*	Afrotropics	0	-0.0028	-	3.24	0.8121	0.2101	12.52
Strigidae	*Otus leucotis*	Palearctic	0	-0.4816	-	3.45	0.8317	0.4448	66.37
Strigidae	*Otus longicornis*	Afrotropics	0	-0.0197	-	3.21	0.7172	0.1718	75.22
Strigidae	*Otus magicus*	IndoMalay	0	-0.0100	-	2.82	0.7609	0.5817	2.48
Strigidae	*Otus manadensis*	Australasia	0	-0.1614	-	3.13	0.7648	0.3410	0.82
Strigidae	*Otus megalotis*	Australasia	0	0.0328	-	2.48	0.6370	0.1926	10.82
Strigidae	*Otus mindorensis*	IndoMalay	0	-1.8472	6.1385	2.92	0.6690	0.7647	63.57
Strigidae	*Otus mirus*	IndoMalay	1	-0.1010	1.8078	2.67	0.6935	0.4176	83.39
Strigidae	*Otus rufescens*	Afrotropics	0	-0.1757	-	3.58	0.8351	0.5505	63.31
Strigidae	*Otus rutilus*	Australasia	0	-0.1595	-	2.56	0.6523	0.1875	30.24
Strigidae	*Otus sagittatus*	Palearctic	1	-0.1569	5.5601	3.35	0.7382	0.3084	22.11
Strigidae	*Otus scops*	Afrotropics	0	0.0837	-	3.56	0.7567	0.5042	88.12
Strigidae	*Otus senegalensis*	Afrotropics	0	0.0536	-	3.22	0.8058	0.2891	10.41
Strigidae	*Otus silvicola*	Palearctic	0	-0.6617	5.6117	4.35	0.8588	0.3252	1.39
Strigidae	*Otus spilocephalus*	Afrotropics	0	8.0878	-	3.33	0.7389	0.4275	24.42
Strigidae	*Otus sunia*	Australasia	0	0.2687	-	2.48	0.6221	0.5774	71.57
Strigidae	*Otus thilohoffmanni*	Australasia	0	-0.3810	-	3.31	0.8170	0.5150	45.69
Strigidae	*Pseudoscops clamator*	IndoMalay	0	-0.1076	-	2.65	0.6716	0.7172	75.17
Strigidae	*Pseudoscops grammicus*	IndoMalay	1	0.3829	0.6020	2.52	0.5985	0.0024	45.41
Strigidae	*Pulsatrix koeniswaldiana*	Australasia	0	-0.0092	-	2.82	0.7151	0.2151	28.00
Strigidae	*Pulsatrix melanota*	Australasia	1	0.5236	1.3227	2.65	0.6948	0.4610	2.59
Strigidae	*Pulsatrix perspicillata*	Afrotropics	0	0.0108	8.9801	3.26	0.8151	0.2640	13.22
Strigidae	*Scotopelia bouvieri*	Neotropics	0	-0.3951	2.2317	3.10	0.7078	0.2307	81.84
Strigidae	*Scotopelia peli*	Nearctic	0	-0.5661	-	6.01	0.6562	0.1622	82.24
Strigidae	*Scotopelia ussheri*	Neotropics	0	-0.2481	-	3.67	0.7179	0.3587	74.36
Strigidae	*Strix albitarsis*	Afrotropics	0	-0.3459	-	3.42	0.7645	0.4127	50.60
Strigidae	*Strix aluco*	Nearctic	0	-0.7233	15.1201	4.19	0.8042	0.5543	57.46
Strigidae	*Strix butleri*	Nearctic	0	-0.6660	12.2305	3.29	0.7849	0.3022	54.25
Strigidae	*Strix chacoensis*	Nearctic	0	-0.6679	14.6058	4.19	0.7795	0.5858	34.02
Strigidae	*Strix fulvescens*	Nearctic	0	-0.5903	12.1170	4.30	0.8274	0.1574	15.21
Strigidae	*Strix huhula*	Afrotropics	0	-0.1293	-	3.81	0.8128	0.1162	2.26
Strigidae	*Strix hylophila*	Afrotropics	0	-0.1742	7.2064	3.40	0.7942	0.8162	30.34
Strigidae	*Strix leptogrammica*	IndoMalay	0	-0.1565	5.1264	3.18	0.8388	0.6119	22.13
Strigidae	*Strix nebulosa*	IndoMalay	0	-0.0280	-	2.64	0.7327	0.2318	67.52
Strigidae	*Strix nigrolineata*	IndoMalay	1	0.1863	5.9768	2.84	0.6935	0.3632	50.49
Strigidae	*Strix occidentalis*	Australasia	0	-0.0791	3.5790	2.72	0.7151	0.5029	56.34
Strigidae	*Strix ocellata*	Neotropics	0	0.0094	-	2.46	0.6876	0.3855	3.42
Strigidae	*Strix rufipes*	Neotropics	0	-0.4269	-	2.47	0.5985	0.0000	0.00
Strigidae	*Strix seloputo*	Neotropics	0	0.4459	-	2.41	0.5788	0.0000	0.00
Strigidae	*Strix uralensis*	Neotropics	0	-0.3315	1.4166	2.54	0.6997	0.3393	19.74
Strigidae	*Strix varia*	Nearctic	0	-0.7317	5.2473	4.41	0.8183	0.7176	22.82
Strigidae	*Strix virgata*	Neotropics	1	-0.1800	7.5112	3.67	0.7735	0.3560	72.13
Strigidae	*Strix woodfordii*	Neotropics	0	-0.1971	4.9457	3.83	0.8536	0.4938	36.58
Strigidae	*Surnia ulula*	Neotropics	0	-0.2333	-	3.75	0.8206	0.6419	20.43
Strigidae	*Uroglaux dimorpha*	Neotropics	0	-0.1934	3.6639	3.98	0.8402	0.6236	26.67
Strigidae	*Xenoglaux loweryi*	Neotropics	0	-0.1032	-	4.05	0.7859	0.7654	31.10
Struthionidae	*Struthio camelus*	Nearctic	0	-0.4918	3.1546	4.02	0.7844	0.2708	41.53
Sturnidae	*Acridotheres albocinctus*	IndoMalay	0	-0.1790	2.2812	3.26	0.7245	0.5225	0.29
Sturnidae	*Acridotheres cinereus*	Australasia	0	-0.1231	-	2.61	0.6716	0.1833	38.82
Sturnidae	*Acridotheres cristatellus*	IndoMalay	0	-0.4502	-	3.24	0.8155	0.4305	4.72
Sturnidae	*Acridotheres fuscus*	IndoMalay	0	-0.0582	-	3.31	0.8095	0.6610	19.35
Sturnidae	*Acridotheres ginginianus*	IndoMalay	0	-0.2141	-	3.59	0.7977	0.5293	10.02
Sturnidae	*Acridotheres grandis*	IndoMalay	0	-0.1020	-	3.06	0.7985	0.6104	41.21
Sturnidae	*Acridotheres tristis*	IndoMalay	0	-0.4016	-	3.43	0.8436	0.5296	23.30
Sturnidae	*Ampeliceps coronatus*	IndoMalay	0	-0.0964	-	3.07	0.7920	0.5191	44.10
Sturnidae	*Aplonis brunneicapillus*	Australasia	1	-0.0829	2.6045	2.48	0.6120	-	-
Sturnidae	*Aplonis brunneicapillus*	Australasia	1	-0.0829	2.6045	2.48	0.6120	0.0400	36.10
Sturnidae	*Aplonis cantoroides*	Australasia	0	-0.0867	-	2.76	0.7740	0.3024	16.79
Sturnidae	*Aplonis grandis*	Australasia	0	-0.0740	-	2.48	0.6120	0.0400	36.10
Sturnidae	*Aplonis metallica*	Australasia	0	-0.1677	-	2.79	0.7771	0.5375	39.63
Sturnidae	*Aplonis minor*	Australasia	0	-0.1582	-	2.69	0.7464	0.5543	34.11
Sturnidae	*Aplonis mysolensis*	Australasia	0	0.0608	-	2.49	0.6740	0.7743	40.32
Sturnidae	*Aplonis mysolensis*	Australasia	0	0.0608	-	2.49	0.6740	-	-
Sturnidae	*Aplonis mystacea*	Australasia	0	-0.0773	0.9028	2.86	0.7256	0.2342	26.74
Sturnidae	*Aplonis panayensis*	IndoMalay	0	-0.0850	-	2.82	0.8023	-	-
Sturnidae	*Aplonis panayensis*	IndoMalay	0	-0.0850	-	2.82	0.8023	0.7197	28.53
Sturnidae	*Aplonis striata*	Australasia	0	-0.0974	-	2.58	0.6302	0.1134	34.50
Sturnidae	*Basilornis celebensis*	Neotropics	0	-0.0162	10.5177	3.45	0.7519	0.2432	62.77
Sturnidae	*Basilornis corythaix*	Neotropics	0	-0.0728	-	3.67	0.7872	0.1561	29.39

Family	Species	Realm	Threat	Latitude	Elevation	Anomaly	Size	Shape	Orientation
Sturnidae	*Basilornis galeatus*	Neotropics	0	8.7210	4.8939	4.03	0.7359	0.2551	38.89
Sturnidae	*Basilornis mirandus*	Neotropics	0	-0.3610	7.2946	3.32	0.6842	0.2672	74.40
Sturnidae	*Buphagus africanus*	Neotropics	0	0.0065	-	4.05	0.8244	0.8205	4.68
Sturnidae	*Buphagus erythrorhynchus*	Nearctic	0	-0.6661	-	4.26	0.8299	0.2110	11.12
Sturnidae	*Cinnyricinclus femoralis*	Australasia	0	-0.5472	-	3.38	0.8354	0.5351	8.00
Sturnidae	*Cinnyricinclus leucogaster*	Australasia	0	-0.0816	2.0583	2.75	0.7120	0.1604	26.20
Sturnidae	*Cinnyricinclus sharpii*	Australasia	0	-0.7370	-	3.60	0.7977	0.2357	2.52
Sturnidae	*Coccycolius iris*	Neotropics	1	-0.1995	0.0000	4.29	0.7256	0.5620	4.81
Sturnidae	*Cosmopsarus regius*	Australasia	0	-0.5908	-	2.75	0.7510	0.4983	43.88
Sturnidae	*Cosmopsarus unicolor*	Palearctic	0	-0.6579	0.3487	3.35	0.8030	0.5910	53.94
Sturnidae	*Creatophora cinerea*	Neotropics	0	-69.4989	6.7459	4.14	0.6891	0.4584	28.06
Sturnidae	*Enodes erythrophris*	Nearctic	0	-0.5423	11.3446	3.23	0.7818	0.4778	57.93
Sturnidae	*Gracula ptilogenys*	IndoMalay	0	-0.3470	-	3.29	0.8259	0.6197	1.73
Sturnidae	*Gracula religiosa*	Nearctic	0	-0.6288	14.0020	3.54	0.7956	0.5812	53.58
Sturnidae	*Grafisia torquata*	Neotropics	0	-0.0347	3.1810	3.39	0.7495	0.2399	35.13
Sturnidae	*Lamprotornis acuticaudus*	Afrotropics	0	7.5248	9.8932	3.43	0.7008	0.3646	87.05
Sturnidae	*Lamprotornis australis*	IndoMalay	0	-0.0538	-	2.92	0.8058	0.4974	57.52
Sturnidae	*Lamprotornis caudatus*	Neotropics	0	0.0901	8.6606	3.60	0.7618	0.3459	88.07
Sturnidae	*Lamprotornis chalcurus*	Afrotropics	0	-0.0022	-	3.20	0.7455	0.6803	29.74
Sturnidae	*Lamprotornis chalybaeus*	Afrotropics	0	0.0433	6.9505	3.47	0.8112	0.1602	5.07
Sturnidae	*Lamprotornis chloropterus*	Afrotropics	0	-0.0913	10.1521	3.82	0.7973	0.6720	26.84
Sturnidae	*Lamprotornis corruscus*	Afrotropics	0	-0.0792	-	3.37	0.8104	0.1283	7.78
Sturnidae	*Lamprotornis cupreocauda*	Afrotropics	0	-0.0788	8.2888	3.61	0.8165	0.6680	68.89
Sturnidae	*Lamprotornis elisabeth*	Afrotropics	0	-0.1132	7.4654	3.43	0.8350	0.6122	31.75
Sturnidae	*Lamprotornis hildebrandti*	Afrotropics	0	0.0264	-	3.40	0.8183	0.1567	5.66
Sturnidae	*Lamprotornis mevesii*	Afrotropics	0	0.0000	-	3.40	0.6598	0.7578	59.35
Sturnidae	*Lamprotornis nitens*	Afrotropics	0	-0.0835	-	3.58	0.8496	0.6655	36.72
Sturnidae	*Lamprotornis pulcher*	Afrotropics	0	0.1769	-	3.88	0.7272	0.3476	9.95
Sturnidae	*Lamprotornis purpureiceps*	Palearctic	0	-0.7835	-	5.14	0.8694	0.0656	0.47
Sturnidae	*Lamprotornis purpureus*	Nearctic	0	-0.8577	-	3.63	0.8007	0.2948	28.24
Sturnidae	*Lamprotornis purpuroptera*	Palearctic	0	-0.7017	14.5330	5.11	0.8586	0.0739	1.12
Sturnidae	*Lamprotornis shelleyi*	Australasia	0	-0.0129	-	2.72	0.7461	0.2002	13.85
Sturnidae	*Lamprotornis splendidus*	Australasia	0	-0.0129	-	2.48	0.6302	0.2423	87.23
Sturnidae	*Lamprotornis superbus*	Australasia	0	-0.3519	-	3.06	0.7901	0.5771	52.44
Sturnidae	*Leucopsar rothschildi*	Neotropics	0	-0.0605	-	4.07	0.8338	0.7716	9.96
Sturnidae	*Mino anais*	Neotropics	1	0.0000	3.3899	3.28	0.5985	0.5774	45.00
Sturnidae	*Mino dumontii*	Neotropics	0	-16.5787	6.6074	4.06	0.6906	0.2473	54.83
Sturnidae	*Onychognathus albirostris*	Neotropics	0	-4.7734	-	3.78	0.6859	0.1456	71.90
Sturnidae	*Onychognathus blythii*	IndoMalay	0	0.0209	-	2.85	0.7911	0.5774	22.11
Sturnidae	*Onychognathus frater*	IndoMalay	0	-0.1186	6.4903	3.19	0.8114	0.4383	8.86
Sturnidae	*Onychognathus fulgidus*	Australasia	0	-0.5497	-	3.42	0.8342	0.5092	9.20
Sturnidae	*Onychognathus morio*	Neotropics	0	-0.2996	-	3.77	0.8227	0.7549	13.53
Sturnidae	*Onychognathus nabouroup*	Neotropics	0	-0.2810	-	3.68	0.8195	0.3100	38.03
Sturnidae	*Onychognathus salvadorii*	Neotropics	0	0.1377	-	3.15	0.7441	0.3680	65.60
Sturnidae	*Onychognathus tenuirostris*	Neotropics	0	-0.0394	-	4.10	0.8081	0.5004	8.32
Sturnidae	*Onychognathus tristramii*	Neotropics	0	0.2553	-	1.51	0.6221	0.0002	1.05
Sturnidae	*Onychognathus walleri*	Neotropics	0	-0.6470	0.0000	3.03	0.5985	0.0856	5.43
Sturnidae	*Poeoptera kenricki*	Afrotropics	1	1.1881	10.3797	3.25	0.6740	0.3758	37.43
Sturnidae	*Poeoptera lugubris*	Afrotropics	1	0.0227	4.3712	3.19	0.6961	0.2387	14.20
Sturnidae	*Poeoptera stuhlmanni*	IndoMalay	0	-0.0960	-	3.50	0.7799	0.2693	9.62
Sturnidae	*Sarcops calvus*	Neotropics	0	-0.2967	13.5617	3.09	0.7862	0.1321	86.15
Sturnidae	*Saroglossa aurata*	Neotropics	0	-0.0770	-	4.15	0.7305	0.2852	66.51
Sturnidae	*Saroglossa spiloptera*	Neotropics	0	-0.0327	-	4.29	0.7698	0.5690	74.97
Sturnidae	*Scissirostrum dubium*	Australasia	0	-0.2788	0.0000	3.08	0.7897	0.4503	49.32
Sturnidae	*Speculipastor bicolor*	IndoMalay	0	-0.4511	6.5766	3.34	0.7464	0.4319	7.38
Sturnidae	*Spreo albicapillus*	Afrotropics	1	7.5448	2.5101	3.24	0.6120	1.0000	0.00
Sturnidae	*Spreo bicolor*	Afrotropics	0	-0.0278	10.7521	3.58	0.7772	0.6131	30.46
Sturnidae	*Spreo fischeri*	Afrotropics	0	-0.0067	-	3.21	0.7888	0.1946	10.76
Sturnidae	*Streptocitta albicollis*	Neotropics	0	-0.4173	2.0215	2.52	0.7877	0.5112	29.43
Sturnidae	*Sturnus albofrontatus*	Palearctic	0	-0.4535	-	3.34	0.8054	0.7138	3.21
Sturnidae	*Sturnus burmannicus*	Afrotropics	0	-0.3574	-	4.14	0.7899	0.6714	48.52
Sturnidae	*Sturnus cineraceus*	Afrotropics	1	2.4785	2.5061	3.61	0.6824	0.5725	5.80
Sturnidae	*Sturnus contra*	Afrotropics	0	-0.0027	-	3.07	0.6906	0.1462	48.84
Sturnidae	*Sturnus malabaricus*	Afrotropics	0	1.2912	6.0512	3.34	0.7128	0.2382	30.36
Sturnidae	*Sturnus melanopterus*	Afrotropics	0	2.1744	-	3.95	0.7438	0.3979	55.88
Sturnidae	*Sturnus nigricollis*	Neotropics	0	-0.1222	-	3.55	0.7775	0.7214	69.59
Sturnidae	*Sturnus pagodarum*	Neotropics	0	0.0360	0.3140	3.69	0.8104	0.5670	18.81
Sturnidae	*Sturnus philippensis*	Neotropics	0	-0.1750	-	3.25	0.7488	0.5019	46.76
Sturnidae	*Sturnus roseus*	Neotropics	0	-0.2665	-	3.62	0.8205	0.4471	50.64
Sturnidae	*Sturnus sericeus*	Neotropics	0	0.0967	-	3.82	0.7693	0.1892	12.49
Sturnidae	*Sturnus sinensis*	Neotropics	0	-0.3181	-	3.62	0.8403	0.7904	83.60
Sturnidae	*Sturnus sturninus*	Neotropics	0	0.1464	-	3.99	0.8188	0.6667	20.45
Sturnidae	*Sturnus unicolor*	Neotropics	1	-0.8208	0.7799	2.61	0.7050	0.6214	74.10
Sturnidae	*Sturnus vulgaris*	Neotropics	0	-0.4639	-	3.49	0.8167	0.8382	31.44

Family	Species	Realm	Threat	Latitude	Elevation	Anomaly	Size	Shape	Orientation
Sylviidae	*Abroscopus albogularis*	IndoMalay	0	-0.4080	3.2130	3.27	0.8044	0.5060	4.71
Sylviidae	*Abroscopus schisticeps*	IndoMalay	0	-0.1570	-	3.61	0.7845	0.3539	10.70
Sylviidae	*Abroscopus superciliaris*	IndoMalay	0	-0.0610	5.6510	3.01	0.8132	0.4002	62.24
Sylviidae	*Achaetops pycnopygius*	Afrotropics	0	-0.2834	-	3.89	0.7672	0.3312	74.46
Sylviidae	*Acrocephalus aedon*	Palearctic	0	-0.8030	-	4.49	0.8363	0.3632	4.81
Sylviidae	*Acrocephalus agricola*	Palearctic	0	-0.9620	0.0000	4.56	0.8443	0.4046	0.03
Sylviidae	*Acrocephalus arundinaceus*	Palearctic	0	-0.5587	6.2064	4.21	0.8633	0.1682	2.80
Sylviidae	*Acrocephalus australis*	Australasia	0	-0.6304	-	3.33	0.8205	0.4811	10.44
Sylviidae	*Acrocephalus bistrigiceps*	Palearctic	0	-0.8212	-	3.99	0.8150	0.6386	38.22
Sylviidae	*Acrocephalus concinens*	Palearctic	0	-0.6135	-	3.74	0.8034	0.2372	0.84
Sylviidae	*Acrocephalus dumetorum*	Palearctic	0	-0.8917	3.4141	4.53	0.8475	0.3307	1.39
Sylviidae	*Acrocephalus gracilirostris*	Afrotropics	0	-0.1254	-	3.60	0.8307	0.5199	73.10
Sylviidae	*Acrocephalus griseldis*	Palearctic	1	-0.4119	0.0000	4.31	0.7151	0.3800	45.01
Sylviidae	*Acrocephalus melanopogon*	Palearctic	0	-0.8347	-	4.04	0.8109	0.1776	0.10
Sylviidae	*Acrocephalus newtoni*	Afrotropics	0	-0.0757	-	3.18	0.7614	0.3874	70.15
Sylviidae	*Acrocephalus orinus*	Palearctic	0	4.2844	-	4.51	0.6859	0.2546	45.32
Sylviidae	*Acrocephalus paludicola*	Palearctic	1	-0.7225	0.8434	4.97	0.8122	0.1204	5.62
Sylviidae	*Acrocephalus palustris*	Palearctic	0	-0.5438	-	4.33	0.8342	0.3942	1.09
Sylviidae	*Acrocephalus rufescens*	Afrotropics	0	-0.0529	-	3.42	0.8012	0.5559	33.12
Sylviidae	*Acrocephalus schoenobaenus*	Palearctic	0	-0.7044	-	4.84	0.8530	0.3038	4.31
Sylviidae	*Acrocephalus scirpaceus*	Palearctic	0	-0.4030	4.2720	3.94	0.8605	0.6396	77.04
Sylviidae	*Acrocephalus sorghophilus*	IndoMalay	1	0.1314	-	2.68	0.6120	0.9615	90.00
Sylviidae	*Acrocephalus stentoreus*	Palearctic	0	-0.3889	9.0503	3.58	0.8322	0.2701	24.04
Sylviidae	*Acrocephalus tangorum*	Palearctic	1	-0.7626	0.0000	4.45	0.7618	0.3830	19.44
Sylviidae	*Amphilais seebohmi*	Afrotropics	0	-0.0954	-	3.15	0.7469	0.3218	71.38
Sylviidae	*Artisornis metopias*	Australasia	0	-0.5019	-	3.44	0.8342	0.5234	6.07
Sylviidae	*Artisornis moreaui*	Australasia	0	-0.0246	-	2.71	0.7179	0.5352	59.02
Sylviidae	*Bathmocercus cerviniventris*	Australasia	0	0.0853	-	2.71	0.7185	0.5623	60.71
Sylviidae	*Bathmocercus rufus*	Australasia	0	0.1012	-	2.48	0.6370	0.1926	10.82
Sylviidae	*Bernieria apperti*	IndoMalay	0	0.0686	4.7858	2.96	0.7179	0.2219	48.41
Sylviidae	*Bernieria cinereiceps*	IndoMalay	0	0.0527	4.1978	2.84	0.7657	0.3986	0.16
Sylviidae	*Bernieria madagascariensis*	IndoMalay	0	-0.0316	-	2.64	0.7305	0.2218	67.86
Sylviidae	*Bernieria tenebrosa*	IndoMalay	0	0.0636	-	2.86	0.7811	0.4518	2.78
Sylviidae	*Bernieria zosterops*	Neotropics	0	-0.0018	-	4.09	0.8287	0.7475	23.58
Sylviidae	*Bowdleria punctata*	Afrotropics	0	-0.0020	-	3.32	0.8114	0.4254	17.21
Sylviidae	*Bradypterus accentor*	IndoMalay	0	-0.0673	13.1065	3.56	0.7382	0.1951	10.94
Sylviidae	*Bradypterus alfredi*	Nearctic	1	-0.8968	-	4.14	0.6662	0.0265	0.15
Sylviidae	*Bradypterus alishanensis*	Palearctic	1	-0.8453	-	3.82	0.7812	0.0337	0.02
Sylviidae	*Bradypterus baboecala*	Palearctic	0	-0.7440	-	4.16	0.7844	0.3360	35.02
Sylviidae	*Bradypterus bangwaensis*	Afrotropics	0	-0.3416	-	4.08	0.7943	0.5760	58.06
Sylviidae	*Bradypterus barratti*	Afrotropics	0	-0.3561	-	4.21	0.7978	0.6329	9.40
Sylviidae	*Bradypterus carpalis*	Afrotropics	0	-0.0131	8.1617	3.34	0.8082	0.4684	49.63
Sylviidae	*Bradypterus castaneus*	Afrotropics	0	-0.0761	-	3.52	0.8466	0.5814	32.31
Sylviidae	*Bradypterus caudatus*	IndoMalay	0	0.0000	3.7731	2.72	0.5788	0.0000	45.00
Sylviidae	*Bradypterus cinnamomeus*	Afrotropics	0	0.5979	7.5559	3.47	0.7327	0.1938	63.08
Sylviidae	*Bradypterus davidi*	IndoMalay	0	-0.8950	9.4257	2.61	0.6478	0.3444	72.46
Sylviidae	*Bradypterus grandis*	Afrotropics	0	-0.1344	-	3.58	0.8200	0.6060	86.40
Sylviidae	*Bradypterus graueri*	Afrotropics	0	0.5779	8.0921	3.23	0.6690	0.2470	44.41
Sylviidae	*Bradypterus lopezi*	Afrotropics	0	-0.2856	-	3.35	0.7428	0.1965	68.37
Sylviidae	*Bradypterus luteoventris*	Afrotropics	0	3.6364	-	3.41	0.7318	0.4627	81.71
Sylviidae	*Bradypterus major*	Australasia	0	0.4940	-	2.70	0.7040	0.6342	1.60
Sylviidae	*Bradypterus mandelli*	IndoMalay	0	-0.0072	-	2.65	0.6921	0.1079	66.01
Sylviidae	*Bradypterus montis*	Afrotropics	0	0.0068	11.3414	3.39	0.7769	0.4435	73.16
Sylviidae	*Bradypterus palliseri*	Palearctic	0	-0.5724	4.7296	4.43	0.7906	0.3167	25.61
Sylviidae	*Bradypterus seebohmi*	Afrotropics	0	-111.5010	2.2363	3.21	0.6662	0.2010	45.05
Sylviidae	*Bradypterus sylvaticus*	Afrotropics	1	-4.6848	4.0357	3.44	0.6842	0.3172	89.47
Sylviidae	*Bradypterus tacsanowskius*	Afrotropics	0	-0.0249	4.2642	3.47	0.7757	0.7898	15.13
Sylviidae	*Bradypterus thoracicus*	IndoMalay	0	-0.5188	7.2635	3.32	0.8005	0.3533	4.84
Sylviidae	*Bradypterus timorensis*	Palearctic	0	-0.0608	7.1336	4.49	0.7510	0.5341	25.60
Sylviidae	*Bradypterus victorini*	IndoMalay	0	-0.2943	-	3.31	0.7839	0.4284	3.59
Sylviidae	*Buettikoferella bivittata*	Neotropics	0	0.2414	0.2464	2.96	0.6824	0.2779	87.21
Sylviidae	*Cettia acanthizoides*	IndoMalay	0	-0.0900	9.6317	3.18	0.7552	0.4392	37.22
Sylviidae	*Cettia brunnescens*	IndoMalay	0	-0.0410	9.2407	3.97	0.7441	0.1556	5.89
Sylviidae	*Cettia brunnifrons*	Palearctic	0	-0.5897	1.9782	3.59	0.6974	0.3674	49.13
Sylviidae	*Cettia canturians*	Neotropics	0	0.3975	1.1976	4.03	0.8129	0.4802	6.20
Sylviidae	*Cettia carolinae*	Neotropics	0	-0.2200	2.9226	3.64	0.8424	0.7212	65.77
Sylviidae	*Cettia cetti*	Neotropics	0	-0.0335	0.2979	4.09	0.7818	0.2868	11.77
Sylviidae	*Cettia flavolivacea*	Afrotropics	0	-0.1178	-	3.36	0.7432	0.5572	14.43
Sylviidae	*Cettia fortipes*	Afrotropics	0	-0.1314	0.0000	3.11	0.6631	0.3188	60.28
Sylviidae	*Cettia haddeni*	Afrotropics	1	-6.7251	2.9321	3.82	0.7040	0.4921	5.07
Sylviidae	*Cettia major*	Afrotropics	0	-9.3909	-	4.21	0.7277	0.4555	54.04
Sylviidae	*Cettia pallidipes*	Afrotropics	0	0.0216	0.0000	2.79	0.7030	-	-
Sylviidae	*Cettia pallidipes*	Afrotropics	0	0.0216	0.0000	2.79	0.7030	0.1922	57.20
Sylviidae	*Cettia parens*	Afrotropics	0	-0.0470	0.0000	3.32	0.6824	0.3254	76.76

Family	Species	Realm	Threat	Latitude	Elevation	Anomaly	Size	Shape	Orientation
Sylviidae	*Cettia ruficapilla*	Afrotropics	0	-0.3450	0.0000	3.60	0.7588	0.5824	54.90
Sylviidae	*Cettia seebohmi*	Afrotropics	0	-0.3281	0.0000	3.72	0.7773	0.2468	59.57
Sylviidae	*Cettia vulcania*	Australasia	0	-0.6098	-	3.42	0.8298	0.5053	11.87
Sylviidae	*Chaetornis striata*	IndoMalay	0	0.0297	-	2.82	0.7770	0.5790	9.21
Sylviidae	*Chloropeta gracilirostris*	Neotropics	0	-0.1165	2.2443	3.96	0.8265	0.5702	15.54
Sylviidae	*Chloropeta similis*	Neotropics	0	-0.1349	6.7640	3.94	0.8448	0.4664	43.12
Sylviidae	*Cincloramphus cruralis*	IndoMalay	0	-0.7430	2.1355	3.29	0.7428	0.4629	29.19
Sylviidae	*Cincloramphus mathewsi*	IndoMalay	0	-0.3495	-	3.27	0.8143	0.9418	87.51
Sylviidae	*Crossleyia xanthophrys*	Neotropics	0	-0.0554	-	4.01	0.8032	0.4319	4.11
Sylviidae	*Cryptosylvicola randrianasoloi*	Afrotropics	0	0.0423	-	3.24	0.7887	0.3623	3.56
Sylviidae	*Dromaeocercus brunneus*	Afrotropics	0	0.0152	6.5167	3.34	0.7774	0.6893	87.67
Sylviidae	*Eremiornis carteri*	Australasia	0	0.6505	-	2.48	0.6478	0.1611	2.45
Sylviidae	*Eremomela badiceps*	Australasia	0	0.1417	10.0653	2.48	0.6120	0.0000	0.00
Sylviidae	*Eremomela flavicrissalis*	Australasia	0	0.2369	-	2.48	0.6428	0.4850	33.00
Sylviidae	*Eremomela gregalis*	IndoMalay	0	-0.1155	-	3.41	0.8353	0.1475	36.61
Sylviidae	*Eremomela icteropygialis*	Afrotropics	0	-0.0641	-	3.57	0.8532	0.7075	38.17
Sylviidae	*Eremomela pusilla*	Australasia	0	-0.1400	-	2.72	0.7040	0.1388	25.10
Sylviidae	*Eremomela scotops*	Australasia	0	0.1914	0.5080	2.69	0.6986	0.3166	19.26
Sylviidae	*Eremomela turneri*	Australasia	1	-0.0170	2.1776	2.85	0.7300	0.1582	19.83
Sylviidae	*Eremomela usticollis*	Australasia	0	-0.2289	5.9267	2.85	0.7356	0.1565	23.70
Sylviidae	*Graminicola bengalensis*	Neotropics	0	0.0000	7.0217	4.02	0.6478	0.5057	63.89
Sylviidae	*Graueria vittata*	Neotropics	0	4.3832	6.3605	4.01	0.6948	0.2280	33.41
Sylviidae	*Hemitesia neumanni*	Neotropics	0	-0.4264	-	3.49	0.8072	0.6692	74.44
Sylviidae	*Hippolais caligata*	Neotropics	0	-0.1156	1.6097	4.01	0.7599	0.3326	21.81
Sylviidae	*Hippolais languida*	Neotropics	0	1.7742	-	3.72	0.7318	0.6608	75.17
Sylviidae	*Hippolais olivetorum*	Neotropics	0	0.5110	-	3.97	0.7775	0.3582	22.38
Sylviidae	*Hippolais opaca*	Neotropics	0	-0.0389	-	4.22	0.8044	0.5521	15.29
Sylviidae	*Hippolais pallida*	IndoMalay	1	0.0000	1.5930	3.59	0.6221	0.1614	7.24
Sylviidae	*Hippolais rama*	Afrotropics	1	1.0612	3.1058	3.39	0.6662	0.2241	75.99
Sylviidae	*Hylia prasina*	Afrotropics	0	-0.2568	-	3.88	0.8212	0.7964	86.28
Sylviidae	*Hyliota australis*	IndoMalay	0	0.1182	6.9208	2.97	0.7096	0.2315	48.12
Sylviidae	*Hyliota flavigaster*	IndoMalay	0	-0.2247	3.4566	3.60	0.8082	0.7730	33.62
Sylviidae	*Hyliota usambara*	Afrotropics	0	0.2659	-	3.19	0.7531	0.4194	61.74
Sylviidae	*Hyliota violacea*	Afrotropics	0	-0.1170	12.3433	3.57	0.8437	0.6598	60.42
Sylviidae	*Locustella certhiola*	Palearctic	0	-0.8776	13.4192	4.36	0.8266	0.6924	5.25
Sylviidae	*Locustella fasciolata*	Palearctic	0	9.2104	-	4.38	0.6302	0.7471	0.00
Sylviidae	*Locustella fluviatilis*	Nearctic	0	-0.8592	-	4.00	0.8072	0.2977	19.80
Sylviidae	*Locustella lanceolata*	IndoMalay	0	0.0010	4.4006	2.65	0.7030	0.4071	59.43
Sylviidae	*Locustella lanceolata*	IndoMalay	0	0.0010	4.4006	2.65	0.7030	-	-
Sylviidae	*Locustella luscinioides*	Australasia	0	-0.4042	8.4088	2.89	0.7861	0.5078	80.85
Sylviidae	*Locustella naevia*	Australasia	0	-0.5673	-	3.15	0.7925	0.6438	69.97
Sylviidae	*Locustella ochotensis*	Australasia	0	-0.8443	-	2.64	0.7604	0.1218	6.18
Sylviidae	*Locustella pleskei*	Australasia	0	-0.5028	-	3.07	0.7008	0.1711	57.74
Sylviidae	*Locustella pryeri*	Australasia	0	0.0226	1.7603	3.41	0.7841	0.3666	1.21
Sylviidae	*Macrosphenus concolor*	Palearctic	0	-0.2848	-	3.91	0.7918	0.3031	5.41
Sylviidae	*Macrosphenus flavicans*	Palearctic	0	-1.1263	-	4.93	0.8491	0.2950	0.44
Sylviidae	*Macrosphenus kempi*	Palearctic	0	-0.7762	-	4.62	0.8371	0.3915	5.76
Sylviidae	*Macrosphenus kretschmeri*	Palearctic	0	-0.6657	6.3081	4.96	0.8375	0.1943	2.01
Sylviidae	*Macrosphenus pulitzeri*	Palearctic	0	-0.7252	4.4468	4.00	0.8424	0.1806	0.44
Sylviidae	*Megalurulus grosvenori*	Australasia	0	-0.4787	-	3.38	0.8026	0.3404	22.98
Sylviidae	*Megalurulus llaneae*	Australasia	0	-0.6660	-	2.74	0.7535	0.2098	6.50
Sylviidae	*Megalurulus mariei*	Australasia	0	-0.6833	-	3.33	0.8120	0.3409	3.57
Sylviidae	*Megalurulus rubiginosus*	Neotropics	0	-0.0011	-	2.94	0.6662	0.1816	26.71
Sylviidae	*Megalurulus whitneyi*	Neotropics	0	-0.0080	2.3409	3.47	0.7490	0.3104	27.98
Sylviidae	*Megalurus albolimbatus*	Neotropics	0	-0.1391	-	3.89	0.8396	0.5481	35.65
Sylviidae	*Megalurus gramineus*	Neotropics	0	-0.0059	-	2.99	0.7179	0.7061	62.67
Sylviidae	*Megalurus palustris*	Afrotropics	0	-0.1112	-	3.30	0.8074	0.5037	28.20
Sylviidae	*Megalurus timoriensis*	Australasia	0	-0.5334	-	3.38	0.8353	0.5597	2.69
Sylviidae	*Melocichla mentalis*	Australasia	0	-0.0890	-	2.80	0.7623	0.2601	25.45
Sylviidae	*Nesillas lantzii*	Afrotropics	0	7.0489	-	3.28	0.7546	0.6115	5.91
Sylviidae	*Nesillas typica*	Afrotropics	1	-0.9773	7.6520	3.44	0.6631	0.4598	85.71
Sylviidae	*Orthotomus atrogularis*	Nearctic	0	-0.4392	-	4.39	0.7933	0.1413	10.24
Sylviidae	*Orthotomus castaneiceps*	Nearctic	0	-0.8303	-	4.12	0.8023	0.5714	8.20
Sylviidae	*Orthotomus cinereiceps*	Nearctic	0	-0.5835	-	4.31	0.8153	0.1905	12.13
Sylviidae	*Orthotomus cuculatus*	Nearctic	0	-0.7012	9.2243	3.81	0.8135	0.3649	42.02
Sylviidae	*Orthotomus derbianus*	Neotropics	0	-0.4304	3.4799	2.92	0.7300	0.1853	29.35
Sylviidae	*Orthotomus frontalis*	Australasia	0	-0.0714	3.6798	2.80	0.7458	0.1622	24.70
Sylviidae	*Orthotomus heterolaemus*	Australasia	0	-0.6486	-	3.37	0.8282	0.4761	6.22
Sylviidae	*Orthotomus nigriceps*	Afrotropics	0	7.1523	7.9980	3.27	0.7318	0.2663	16.01
Sylviidae	*Orthotomus ruficeps*	Afrotropics	0	5.0518	9.1706	3.45	0.7272	0.5688	81.62
Sylviidae	*Orthotomus samarensis*	Neotropics	0	0.1444	7.9203	3.90	0.7498	0.2727	55.26
Sylviidae	*Orthotomus sericeus*	Neotropics	0	-27.2918	7.8556	4.13	0.6859	0.3550	10.85
Sylviidae	*Orthotomus sutorius*	Neotropics	1	-0.5588	12.6626	3.61	0.7008	0.2655	23.80
Sylviidae	*Oxylabes madagascariensis*	IndoMalay	0	-0.0264	-	2.64	0.7251	0.2150	68.34

Family	Species	Realm	Threat	Latitude	Elevation	Anomaly	Size	Shape	Orientation
Sylviidae	*Phylloscopus affinis*	Afrotropics	0	-0.1176	-	3.72	0.7512	0.7224	21.06
Sylviidae	*Phylloscopus armandii*	Neotropics	1	-0.1242	13.3870	4.07	0.7409	0.2495	60.67
Sylviidae	*Phylloscopus bonelli*	Neotropics	0	-2.4510	7.4227	4.21	0.7500	0.3996	71.87
Sylviidae	*Phylloscopus borealis*	Neotropics	0	-0.3695	10.7012	3.01	0.8141	0.3952	81.18
Sylviidae	*Phylloscopus borealoides*	Afrotropics	0	-0.3958	-	3.56	0.8318	0.7451	86.42
Sylviidae	*Phylloscopus budongoensis*	Afrotropics	0	0.1614	11.4019	3.23	0.7938	0.1152	10.05
Sylviidae	*Phylloscopus cantator*	Afrotropics	0	-0.0039	-	3.16	0.7759	0.0969	10.15
Sylviidae	*Phylloscopus cebuensis*	Afrotropics	0	-0.1291	6.8822	3.68	0.7785	0.2018	38.14
Sylviidae	*Phylloscopus chloronotus*	Afrotropics	0	-0.1202	-	3.62	0.8520	0.5714	45.12
Sylviidae	*Phylloscopus claudiae*	Afrotropics	0	0.0323	-	3.47	0.7908	0.7057	68.61
Sylviidae	*Phylloscopus collybita*	Palearctic	0	18.3134	1.2679	4.03	0.7698	0.3998	7.77
Sylviidae	*Phylloscopus coronatus*	Palearctic	0	-1.2369	-	4.24	0.8276	0.5084	25.40
Sylviidae	*Phylloscopus davisoni*	Palearctic	0	-0.9891	11.0429	4.37	0.7841	0.8484	18.68
Sylviidae	*Phylloscopus emeiensis*	Palearctic	0	-1.0311	18.5928	4.37	0.8257	0.5140	0.27
Sylviidae	*Phylloscopus forresti*	Palearctic	0	-1.1916	17.7427	4.42	0.8149	0.3557	22.95
Sylviidae	*Phylloscopus fuligiventer*	Palearctic	0	-2.7243	-	4.20	0.7771	0.2081	9.29
Sylviidae	*Phylloscopus fuscatus*	Palearctic	0	-0.6279	-	4.10	0.7933	0.5852	27.60
Sylviidae	*Phylloscopus goodsoni*	Palearctic	0	-0.6025	14.4175	3.83	0.7636	0.1863	17.89
Sylviidae	*Phylloscopus griseolus*	Palearctic	0	-0.6055	11.9927	4.13	0.8563	0.1790	4.86
Sylviidae	*Phylloscopus hainanus*	Palearctic	0	-0.6753	6.1084	4.62	0.8565	0.2996	3.94
Sylviidae	*Phylloscopus herberti*	Palearctic	0	-1.7055	11.8827	4.10	0.7822	0.4074	18.02
Sylviidae	*Phylloscopus humei*	Afrotropics	0	-0.0054	-	3.25	0.8153	0.2923	13.57
Sylviidae	*Phylloscopus ibericus*	Afrotropics	0	-0.3798	-	3.63	0.7599	0.6220	5.71
Sylviidae	*Phylloscopus inornatus*	Neotropics	0	-0.2144	-	3.69	0.7802	0.3872	71.37
Sylviidae	*Phylloscopus kansuensis*	Neotropics	0	-0.2714	7.4321	4.11	0.7653	0.5329	84.75
Sylviidae	*Phylloscopus laetus*	Neotropics	0	-0.6629	-	2.66	0.7614	0.8677	55.28
Sylviidae	*Phylloscopus laurae*	Neotropics	0	5.9979	3.1885	4.17	0.7359	0.3898	71.91
Sylviidae	*Phylloscopus maculipennis*	Neotropics	0	3.3030	4.4647	4.22	0.7245	0.3632	37.91
Sylviidae	*Phylloscopus magnirostris*	Neotropics	0	-0.2624	11.3996	3.25	0.8046	0.2399	86.34
Sylviidae	*Phylloscopus makirensis*	Neotropics	0	-0.5649	10.6884	2.71	0.7792	0.1705	82.21
Sylviidae	*Phylloscopus neglectus*	Neotropics	0	-0.5281	5.8463	2.07	0.7626	0.2465	87.45
Sylviidae	*Phylloscopus occipitalis*	Neotropics	0	-0.1962	11.8540	3.86	0.7858	0.2774	62.37
Sylviidae	*Phylloscopus ogilviegranti*	Neotropics	0	0.0159	11.4712	3.92	0.7522	0.2560	41.37
Sylviidae	*Phylloscopus olivaceus*	Neotropics	0	-0.1823	9.0170	3.56	0.7977	0.2227	83.98
Sylviidae	*Phylloscopus poliocephalus*	Australasia	0	-0.7571	-	3.10	0.7799	0.3018	10.87
Sylviidae	*Phylloscopus presbytes*	Australasia	0	-0.7258	-	2.79	0.7862	0.2055	7.61
Sylviidae	*Phylloscopus proregulus*	Australasia	0	-0.5628	-	3.13	0.7845	0.3107	7.87
Sylviidae	*Phylloscopus reguloides*	Australasia	0	-0.5643	-	2.73	0.7782	0.2697	2.21
Sylviidae	*Phylloscopus ricketti*	Australasia	0	-0.5794	-	2.75	0.7493	0.7019	35.34
Sylviidae	*Phylloscopus ruficapilla*	Australasia	0	-0.1888	-	2.58	0.6302	0.1134	34.50
Sylviidae	*Phylloscopus sarasinorum*	Afrotropics	0	-0.0065	-	3.22	0.7803	0.1053	9.48
Sylviidae	*Phylloscopus schwarzi*	Afrotropics	0	-0.0002	-	3.23	0.8072	0.2770	10.94
Sylviidae	*Phylloscopus sibilatrix*	Afrotropics	0	0.3572	3.4888	3.21	0.7300	0.1347	6.62
Sylviidae	*Phylloscopus sindianus*	Afrotropics	0	0.0782	13.8422	3.51	0.8060	0.5702	26.48
Sylviidae	*Phylloscopus subaffinis*	Afrotropics	0	0.0139	-	3.54	0.7898	0.6561	39.09
Sylviidae	*Phylloscopus subviridis*	Afrotropics	0	-0.1630	-	3.14	0.7389	0.1324	76.00
Sylviidae	*Phylloscopus tenellipes*	Afrotropics	0	0.0520	-	3.14	0.7444	0.4128	78.41
Sylviidae	*Phylloscopus trivirgatus*	Afrotropics	0	-0.1360	12.2848	3.40	0.7704	0.3937	89.40
Sylviidae	*Phylloscopus trochiloides*	Afrotropics	0	-0.0345	-	3.42	0.7286	0.3181	83.45
Sylviidae	*Phylloscopus trochilus*	Afrotropics	0	4.7461	12.8814	3.34	0.7359	0.6742	59.88
Sylviidae	*Phylloscopus tytleri*	Afrotropics	0	0.0868	-	3.24	0.8102	0.2438	14.74
Sylviidae	*Phylloscopus umbrovirens*	Afrotropics	1	-6.8378	-	3.09	0.5788	0.0000	90.00
Sylviidae	*Phylloscopus xanthoschistos*	Afrotropics	0	0.8555	-	3.21	0.6961	0.3838	43.75
Sylviidae	*Phylloscopus yunnanensis*	Afrotropics	0	0.4124	6.7922	3.23	0.6997	0.4750	37.54
Sylviidae	*Poliolais lopezi*	Afrotropics	0	-12.4117	4.9162	3.44	0.7151	0.1304	87.23
Sylviidae	*Randia pseudozosterops*	IndoMalay	0	-0.0810	-	2.75	0.7344	0.1658	40.05
Sylviidae	*Schoenicola platyurus*	IndoMalay	0	0.0743	-	2.84	0.7870	0.4328	0.46
Sylviidae	*Seicercus affinis*	Neotropics	0	-0.5053	2.3329	2.77	0.8095	0.5451	40.02
Sylviidae	*Seicercus burkii*	Neotropics	0	-0.2407	4.9735	3.68	0.8525	0.5881	47.09
Sylviidae	*Seicercus castaniceps*	Afrotropics	0	0.5025	15.0506	3.54	0.7528	0.4647	80.56
Sylviidae	*Seicercus grammiceps*	Neotropics	0	0.0590	9.0814	3.60	0.7647	0.3648	76.16
Sylviidae	*Seicercus montis*	Neotropics	0	0.0768	-	4.05	0.8033	0.4706	5.68
Sylviidae	*Seicercus omeiensis*	Afrotropics	0	-6.5865	9.6641	3.44	0.6842	0.2893	78.38
Sylviidae	*Seicercus omeiensis*	Afrotropics	0	-6.5865	9.6641	3.44	0.6842	0.2893	78.38
Sylviidae	*Seicercus poliogenys*	IndoMalay	1	-0.2444	-	3.57	0.7528	0.4714	21.14
Sylviidae	*Seicercus soror*	Afrotropics	0	-0.0611	7.2119	3.53	0.8474	0.5595	33.28
Sylviidae	*Seicercus tephrocephalus*	Neotropics	0	-0.3550	-	3.73	0.8547	0.5961	55.54
Sylviidae	*Seicercus valentini*	Afrotropics	1	-0.1193	0.0000	3.65	0.8554	0.7096	64.06
Sylviidae	*Seicercus whistleri*	Neotropics	0	0.1144	-	3.24	0.7205	0.3004	81.13
Sylviidae	*Sphenoeacus afer*	Neotropics	0	-0.4633	6.6473	2.21	0.7656	0.1966	88.83
Sylviidae	*Sylvia althaea*	Neotropics	0	-0.3884	2.3348	2.48	0.7151	0.5877	69.66
Sylviidae	*Sylvia boehmi*	Neotropics	0	0.0525	-	3.73	0.8084	0.2568	17.98
Sylviidae	*Sylvia borin*	Neotropics	0	0.6727	5.1743	3.79	0.7808	0.8121	82.65
Sylviidae	*Sylvia buryi*	Neotropics	0	-0.1554	-	3.74	0.8385	0.4800	35.57

Family	Species	Realm	Threat	Latitude	Elevation	Anomaly	Size	Shape	Orientation
Sylviidae	*Sylvia cantillans*	Neotropics	1	0.2526	-	3.99	0.7291	0.5136	56.53
Sylviidae	*Sylvia communis*	Neotropics	1	-0.8165	0.7749	2.53	0.7185	0.8604	87.23
Sylviidae	*Sylvia conspicillata*	Neotropics	0	-0.1877	-	3.27	0.7179	0.1502	69.55
Sylviidae	*Sylvia curruca*	Neotropics	0	-0.1646	-	3.88	0.8276	0.4951	54.59
Sylviidae	*Sylvia deserticola*	Neotropics	0	-0.6071	2.6867	3.07	0.7914	0.6550	85.54
Sylviidae	*Sylvia hortensis*	Neotropics	0	0.1330	-	3.95	0.7996	0.7522	57.75
Sylviidae	*Sylvia layardi*	Neotropics	0	-0.2861	6.9841	3.52	0.7228	0.1545	68.57
Sylviidae	*Sylvia leucomelaena*	Neotropics	0	-0.2504	3.2690	3.39	0.7385	0.2201	67.24
Sylviidae	*Sylvia lugens*	Neotropics	0	-0.1116	6.2235	3.51	0.7875	0.3152	26.25
Sylviidae	*Sylvia melanocephala*	Afrotropics	0	0.1214	-	3.58	0.8188	0.2943	12.39
Sylviidae	*Sylvia melanothorax*	Afrotropics	0	-0.1640	-	4.13	0.8044	0.7805	22.61
Sylviidae	*Sylvia minula*	Afrotropics	0	0.0258	-	3.41	0.7763	0.4889	25.58
Sylviidae	*Sylvia mystacea*	Afrotropics	0	-0.5497	-	3.59	0.7740	0.4856	30.38
Sylviidae	*Sylvia nana*	Afrotropics	0	-0.0134	-	3.19	0.7692	0.5017	45.36
Sylviidae	*Sylvia nisoria*	IndoMalay	0	0.0334	-	3.24	0.7841	0.5185	17.66
Sylviidae	*Sylvia rueppelli*	IndoMalay	0	-0.0429	3.0906	2.62	0.6986	0.4794	89.83
Sylviidae	*Sylvia sarda*	IndoMalay	0	-1.7829	-	3.21	0.7922	0.5018	72.83
Sylviidae	*Sylvia subcaerulea*	IndoMalay	0	-0.0057	3.0005	2.67	0.6740	0.3048	89.42
Sylviidae	*Sylvia undata*	IndoMalay	0	-0.0007	-	2.84	0.7889	0.4829	6.35
Sylviidae	*Sylvietta brachyura*	IndoMalay	0	-0.2766	5.3823	2.73	0.6876	0.1532	11.00
Sylviidae	*Sylvietta denti*	IndoMalay	0	-0.1073	2.0670	2.81	0.6631	0.5719	16.26
Sylviidae	*Sylvietta isabellina*	IndoMalay	0	0.5208	5.6282	2.51	0.5788	0.0000	45.00
Sylviidae	*Sylvietta leucophrys*	IndoMalay	0	0.4380	4.9012	2.63	0.5788	0.0000	90.00
Sylviidae	*Sylvietta philippae*	IndoMalay	0	0.0551	-	2.78	0.7455	0.3577	0.99
Sylviidae	*Sylvietta rufescens*	IndoMalay	0	0.0647	1.6409	2.86	0.7732	0.4579	3.29
Sylviidae	*Sylvietta ruficapilla*	IndoMalay	0	-0.1110	-	2.71	0.6974	0.1132	11.18
Sylviidae	*Sylvietta virens*	IndoMalay	0	-0.0465	-	3.11	0.8076	0.4259	55.91
Sylviidae	*Sylvietta whytii*	IndoMalay	0	0.0296	-	2.80	0.7416	0.5137	17.18
Sylviidae	*Tesia castaneocoronata*	Neotropics	0	-0.0020	8.4474	3.47	0.7626	0.4717	89.11
Sylviidae	*Tesia cyaniventer*	IndoMalay	1	-0.1397	-	3.38	0.8072	0.8968	59.37
Sylviidae	*Tesia everetti*	Neotropics	0	-0.2759	-	3.43	0.8236	0.3903	70.32
Sylviidae	*Tesia olivea*	IndoMalay	0	-0.7502	7.0370	3.31	0.7654	0.4032	15.69
Sylviidae	*Tesia superciliaris*	IndoMalay	0	-0.4698	10.5053	3.25	0.7607	0.4958	5.59
Sylviidae	*Thamnornis chloropetoides*	Neotropics	0	-0.1651	-	3.82	0.8319	0.5362	48.78
Sylviidae	*Tickellia hodgsoni*	Afrotropics	0	-0.2288	11.8134	3.54	0.8578	0.6458	36.97
Sylviidae	*Trichocichla rufa*	Australasia	0	-0.5080	0.0000	3.36	0.8334	0.5790	8.26
Sylviidae	*Urosphena squameiceps*	IndoMalay	0	0.3277	4.8868	2.85	0.7030	0.3657	33.12
Sylviidae	*Urosphena subulata*	IndoMalay	0	0.0426	2.3241	2.82	0.7281	0.3972	74.30
Sylviidae	*Urosphena whiteheadi*	IndoMalay	0	0.0460	7.5210	2.78	0.7318	0.4940	65.16
Thamnophilidae	*Batara cinerea*	Neotropics	0	-0.0462	5.5911	3.62	0.7773	0.4722	80.41
Thamnophilidae	*Biatas nigropectus*	Afrotropics	0	-0.1286	6.0609	3.11	0.7348	0.1765	73.52
Thamnophilidae	*Cercomacra brasiliana*	IndoMalay	0	-0.1161	-	3.23	0.8398	0.5209	24.40
Thamnophilidae	*Cercomacra carbonaria*	IndoMalay	1	0.6203	1.6289	2.65	0.5788	0.0000	0.00
Thamnophilidae	*Cercomacra cinerascens*	Afrotropics	0	-0.1054	12.2198	3.53	0.8379	0.4988	61.98
Thamnophilidae	*Cercomacra ferdinandi*	Afrotropics	0	-0.0769	6.0192	3.18	0.7614	0.3874	70.15
Thamnophilidae	*Cercomacra laeta*	IndoMalay	0	-0.0691	-	2.66	0.6986	0.4661	74.93
Thamnophilidae	*Cercomacra manu*	Australasia	0	-0.0512	3.2675	2.50	0.6716	0.2680	24.42
Thamnophilidae	*Cercomacra melanaria*	IndoMalay	0	0.0000	-	2.64	0.7327	0.2318	67.52
Thamnophilidae	*Cercomacra nigrescens*	Neotropics	1	-0.1410	7.2286	3.04	0.6740	0.1618	30.33
Thamnophilidae	*Cercomacra nigricans*	Neotropics	0	0.1799	-	4.10	0.8305	0.7117	31.92
Thamnophilidae	*Cercomacra parkeri*	Neotropics	1	0.0217	5.6013	3.03	0.7151	0.2444	63.65
Thamnophilidae	*Cercomacra serva*	IndoMalay	0	-0.3746	-	3.93	0.7623	0.2223	6.78
Thamnophilidae	*Cercomacra tyrannina*	Palearctic	0	-0.7669	-	4.25	0.7704	0.3617	35.54
Thamnophilidae	*Clytoctantes alixii*	Australasia	0	-0.9456	0.0000	3.39	0.7256	0.3836	0.31
Thamnophilidae	*Clytoctantes atrogularis*	IndoMalay	0	-0.5003	-	3.28	0.8316	0.4569	3.90
Thamnophilidae	*Cymbilaimus lineatus*	Neotropics	0	-0.0796	8.1726	3.60	0.7234	0.2997	17.02
Thamnophilidae	*Cymbilaimus sanctaemariae*	Neotropics	0	0.2599	8.3837	3.25	0.7050	0.2150	73.23
Thamnophilidae	*Dichrozona cincta*	Australasia	0	-0.4569	-	3.34	0.8350	0.5942	7.20
Thamnophilidae	*Drymophila caudata*	Neotropics	0	-0.3405	0.6295	2.53	0.6974	0.1632	17.43
Thamnophilidae	*Drymophila devillei*	Neotropics	0	0.0195	-	3.44	0.7669	0.3782	34.94
Thamnophilidae	*Drymophila ferruginea*	Neotropics	0	-0.1709	-	3.36	0.7385	0.1907	72.32
Thamnophilidae	*Drymophila genei*	Nearctic	0	-0.8023	-	4.35	0.8245	0.2391	3.12
Thamnophilidae	*Drymophila malura*	Neotropics	1	-0.8534	4.7402	3.18	0.6921	0.0856	66.33
Thamnophilidae	*Drymophila ochropyga*	Neotropics	1	-10.5649	6.6469	3.91	0.6690	0.1918	59.87
Thamnophilidae	*Drymophila rubricollis*	Neotropics	0	0.6978	-	3.88	0.7524	0.4483	15.53
Thamnophilidae	*Drymophila squamata*	Neotropics	0	-0.1541	1.9418	3.91	0.8431	0.6112	41.01
Thamnophilidae	*Dysithamnus leucostictus*	Australasia	0	-0.2128	-	2.58	0.6302	0.1134	34.50
Thamnophilidae	*Dysithamnus mentalis*	IndoMalay	0	-0.4917	10.0261	2.69	0.6986	0.0638	7.97
Thamnophilidae	*Dysithamnus plumbeus*	Australasia	0	-0.1424	1.5115	2.71	0.7185	0.5623	60.71
Thamnophilidae	*Dysithamnus puncticeps*	Australasia	0	-0.1811	7.0676	2.50	0.6716	0.2680	24.42
Thamnophilidae	*Dysithamnus stictothorax*	IndoMalay	1	0.1920	5.9821	2.66	0.5985	0.5771	46.13
Thamnophilidae	*Dysithamnus striaticeps*	Australasia	0	-0.0830	-	2.82	0.7466	0.6252	22.18
Thamnophilidae	*Dysithamnus xanthopterus*	Australasia	0	-0.0035	-	2.48	0.5788	0.0000	0.00
Thamnophilidae	*Epinecrophylla erythrura*	IndoMalay	0	-0.0214	-	2.75	0.7367	0.1616	40.89

Family	Species	Realm	Threat	Latitude	Elevation	Anomaly	Size	Shape	Orientation
Thamnophilidae	*Epinecrophylla fjeldsaai*	Australasia	0	-0.2312	-	2.72	0.7096	0.5293	48.75
Thamnophilidae	*Epinecrophylla fulviventris*	Neotropics	0	0.0555	10.1878	3.68	0.7688	0.4167	77.63
Thamnophilidae	*Epinecrophylla haematonota*	Neotropics	0	-0.0786	7.3641	2.98	0.6842	0.1857	62.72
Thamnophilidae	*Epinecrophylla leucophthalma*	Neotropics	0	-2.0873	12.2533	4.00	0.7458	0.1477	39.20
Thamnophilidae	*Epinecrophylla ornata*	Australasia	0	-0.3880	-	3.30	0.8107	0.4848	47.42
Thamnophilidae	*Epinecrophylla spodionota*	Palearctic	0	-0.8202	-	4.06	0.8173	0.4007	44.27
Thamnophilidae	*Formicivora erythronotos*	Palearctic	0	-0.4946	5.6593	4.55	0.8331	0.5038	7.22
Thamnophilidae	*Formicivora grantsaui*	IndoMalay	1	0.0584	1.3351	2.52	0.5985	0.0024	45.41
Thamnophilidae	*Formicivora grisea*	Australasia	0	-0.2142	2.0634	2.72	0.7136	0.5225	55.13
Thamnophilidae	*Formicivora iheringi*	Palearctic	0	-0.5826	-	3.36	0.7954	0.6974	20.67
Thamnophilidae	*Formicivora melanogaster*	Palearctic	0	-1.0334	7.9097	3.97	0.7812	0.2541	3.82
Thamnophilidae	*Formicivora rufa*	IndoMalay	0	0.0409	-	2.84	0.7493	0.5194	89.28
Thamnophilidae	*Formicivora serrana*	Palearctic	0	-0.4405	-	3.51	0.8057	0.5098	0.19
Thamnophilidae	*Frederickena viridis*	Afrotropics	0	0.0098	-	3.24	0.8113	0.2121	12.98
Thamnophilidae	*Gymnocichla nudiceps*	Neotropics	1	2.8310	0.5739	3.68	0.6221	0.3473	45.31
Thamnophilidae	*Gymnopithys leucaspis*	Nearctic	1	-0.3869	-	4.50	0.7637	0.2092	28.93
Thamnophilidae	*Gymnopithys lunulatus*	IndoMalay	1	0.0494	-	3.47	0.8021	0.1229	33.55
Thamnophilidae	*Gymnopithys rufigula*	Nearctic	0	-0.8324	-	4.71	0.8442	0.1744	0.57
Thamnophilidae	*Gymnopithys salvini*	Afrotropics	1	-0.1542	12.8033	3.73	0.8079	0.5376	71.38
Thamnophilidae	*Herpsilochmus atricapillus*	Neotropics	0	-0.1866	-	3.91	0.8452	0.7370	39.39
Thamnophilidae	*Herpsilochmus axillaris*	Neotropics	0	-0.2330	-	3.14	0.7687	0.2931	44.92
Thamnophilidae	*Herpsilochmus dorsimaculatus*	Neotropics	0	6.4967	3.0395	3.56	0.6598	0.4727	39.61
Thamnophilidae	*Herpsilochmus dugandi*	Neotropics	0	-0.2597	-	3.12	0.7736	0.2735	35.10
Thamnophilidae	*Herpsilochmus gentryi*	Neotropics	0	-3.7126	-	4.13	0.7769	0.2999	29.13
Thamnophilidae	*Herpsilochmus longirostris*	Neotropics	1	-0.2152	4.8802	2.96	0.7030	0.3301	52.21
Thamnophilidae	*Herpsilochmus motacilloides*	Neotropics	0	-0.0210	8.6011	3.59	0.7548	0.3652	85.50
Thamnophilidae	*Herpsilochmus parkeri*	Neotropics	0	0.0371	2.7141	4.15	0.8112	0.4203	8.98
Thamnophilidae	*Herpsilochmus pectoralis*	Neotropics	0	0.1995	-	3.92	0.6842	0.4269	16.57
Thamnophilidae	*Herpsilochmus pileatus*	Neotropics	0	0.0779	-	4.14	0.7828	0.7932	37.26
Thamnophilidae	*Herpsilochmus roraimae*	Neotropics	0	0.0254	-	3.90	0.7702	0.9548	44.07
Thamnophilidae	*Herpsilochmus rufimarginatus*	Neotropics	1	-0.8484	0.4770	2.35	0.6428	0.3332	78.06
Thamnophilidae	*Herpsilochmus sellowi*	Neotropics	0	-0.1474	6.8195	3.70	0.8316	0.9416	62.74
Thamnophilidae	*Herpsilochmus stictocephalus*	Neotropics	0	-0.1794	-	4.26	0.6370	0.5600	79.98
Thamnophilidae	*Herpsilochmus stictocephalus*	Neotropics	0	-0.1794	-	4.26	0.6370	-	-
Thamnophilidae	*Herpsilochmus sticturus*	Neotropics	0	-0.0817	-	4.19	0.8076	0.6936	6.15
Thamnophilidae	*Hylophylax naevioides*	Neotropics	0	-0.1216	3.6773	4.00	0.8373	0.7179	26.91
Thamnophilidae	*Hylophylax naevius*	Neotropics	0	-0.1079	-	3.36	0.7531	0.2365	31.17
Thamnophilidae	*Hylophylax punctulatus*	Neotropics	0	-0.0487	7.5823	3.03	0.6935	0.2167	62.92
Thamnophilidae	*Hypocnemis cantator*	Neotropics	0	0.0597	-	3.97	0.8193	0.7251	31.50
Thamnophilidae	*Hypocnemis flavescens*	Neotropics	0	-0.1076	-	3.40	0.7576	0.2046	32.27
Thamnophilidae	*Hypocnemis ochrogyna*	Neotropics	0	0.1168	-	4.18	0.7952	0.4274	16.87
Thamnophilidae	*Hypocnemis peruviana*	Neotropics	0	-0.0255	-	3.14	0.7356	0.2806	47.33
Thamnophilidae	*Hypocnemis striata*	Neotropics	0	0.6005	-	3.59	0.7266	0.5530	41.46
Thamnophilidae	*Hypocnemis subflava*	Neotropics	0	-0.0976	1.1560	4.07	0.8140	0.7032	18.64
Thamnophilidae	*Hypocnemoides maculicauda*	Neotropics	0	-0.5002	7.7573	2.84	0.7611	0.5371	24.77
Thamnophilidae	*Hypocnemoides melanopogon*	Neotropics	0	-0.1429	2.2917	3.62	0.7654	0.4065	81.87
Thamnophilidae	*Hypoedaleus guttatus*	Neotropics	0	0.0787	-	3.78	0.7685	0.3034	2.32
Thamnophilidae	*Mackenziaena leachii*	IndoMalay	0	0.3352	-	2.75	0.6562	0.7113	15.53
Thamnophilidae	*Mackenziaena severa*	Australasia	0	-0.0802	3.3618	2.71	0.7158	0.5488	58.89
Thamnophilidae	*Megastictus margaritatus*	IndoMalay	0	-0.4159	-	2.71	0.7112	0.1623	10.99
Thamnophilidae	*Microrhopias quixensis*	Afrotropics	0	0.0881	-	3.56	0.8364	0.1939	6.91
Thamnophilidae	*Myrmeciza atrothorax*	Neotropics	0	0.2618	2.9364	3.31	0.7550	0.6958	9.08
Thamnophilidae	*Myrmeciza berlepschi*	Neotropics	0	-0.2691	-	3.15	0.7087	0.3145	88.63
Thamnophilidae	*Myrmeciza castanea*	Neotropics	0	-0.2537	-	2.53	0.6986	0.2608	17.52
Thamnophilidae	*Myrmeciza disjuncta*	Neotropics	1	-0.2652	1.3939	3.21	0.6891	0.1645	64.40
Thamnophilidae	*Myrmeciza exsul*	Neotropics	0	-0.0918	-	2.46	0.6921	0.2089	5.23
Thamnophilidae	*Myrmeciza ferruginea*	Neotropics	0	-0.2817	-	3.68	0.8485	0.6984	82.66
Thamnophilidae	*Myrmeciza fortis*	Neotropics	0	-0.0793	7.1775	3.90	0.8483	0.3653	35.09
Thamnophilidae	*Myrmeciza goeldii*	Neotropics	0	-0.2279	5.1107	3.77	0.8482	0.3549	38.71
Thamnophilidae	*Myrmeciza griseiceps*	Neotropics	0	-0.4726	-	2.47	0.5985	0.0000	0.00
Thamnophilidae	*Myrmeciza hemimelaena*	Neotropics	0	0.1330	-	3.82	0.7452	0.3252	12.13
Thamnophilidae	*Myrmeciza hyperythra*	Neotropics	0	-0.2077	0.3837	3.01	0.7205	0.5853	55.44
Thamnophilidae	*Myrmeciza immaculata*	Neotropics	0	-0.0938	3.9753	3.81	0.8289	0.4244	17.70
Thamnophilidae	*Myrmeciza laemosticta*	Neotropics	0	-0.2160	3.3810	3.95	0.8341	0.5680	20.63
Thamnophilidae	*Myrmeciza longipes*	Neotropics	0	0.0768	2.5109	3.28	0.7731	0.3256	44.94
Thamnophilidae	*Myrmeciza loricata*	Neotropics	0	0.0617	7.5242	3.62	0.7602	0.3580	77.08
Thamnophilidae	*Myrmeciza melanoceps*	Neotropics	0	-1.2814	7.4591	4.25	0.6523	0.7906	0.00
Thamnophilidae	*Myrmeciza nigricauda*	Neotropics	0	0.0566	6.0552	4.11	0.6631	0.3787	35.79
Thamnophilidae	*Myrmeciza pelzelni*	Neotropics	0	3.9612	9.1285	3.62	0.7314	0.1747	89.74
Thamnophilidae	*Myrmeciza ruficauda*	Neotropics	0	0.0000	3.5833	4.53	0.5985	0.5774	45.00
Thamnophilidae	*Myrmeciza squamosa*	Neotropics	0	0.1957	5.7537	4.23	0.7165	0.3867	33.00
Thamnophilidae	*Myrmoborus leucophrys*	Neotropics	0	-0.0350	-	3.64	0.7913	0.2298	31.32
Thamnophilidae	*Myrmoborus lugubris*	Neotropics	0	-0.2107	7.9815	3.72	0.8558	0.6131	47.42
Thamnophilidae	*Myrmoborus melanurus*	IndoMalay	1	0.5562	0.0000	2.92	0.6302	0.2100	82.64

Family	Species	Realm	Threat	Latitude	Elevation	Anomaly	Size	Shape	Orientation
Thamnophilidae	*Myrmoborus myotherinus*	IndoMalay	1	0.0615	2.9576	3.12	0.6370	0.2826	64.05
Thamnophilidae	*Myrmochanes hemileucus*	Neotropics	0	-0.1429	3.7098	3.86	0.8381	0.5887	33.38
Thamnophilidae	*Myrmorchilus strigilatus*	Neotropics	0	0.0621	7.4537	2.47	0.5985	0.0000	0.00
Thamnophilidae	*Myrmornis torquata*	Neotropics	0	-0.0143	-	3.99	0.7952	0.5889	16.51
Thamnophilidae	*Myrmotherula ambigua*	Neotropics	0	-0.1930	-	3.14	0.7136	0.2465	87.80
Thamnophilidae	*Myrmotherula assimilis*	Neotropics	0	-0.1268	7.9027	3.77	0.8402	0.3933	36.74
Thamnophilidae	*Myrmotherula axillaris*	Afrotropics	0	0.0166	-	3.29	0.8102	0.3445	12.41
Thamnophilidae	*Myrmotherula behni*	Afrotropics	0	-0.0612	-	3.47	0.8422	0.5057	34.41
Thamnophilidae	*Myrmotherula brachyura*	Neotropics	0	5.6857	3.9204	3.54	0.7104	0.3554	87.41
Thamnophilidae	*Myrmotherula cherriei*	Neotropics	0	-0.2431	7.7930	3.62	0.8475	0.8310	40.65
Thamnophilidae	*Myrmotherula fluminensis*	Neotropics	0	0.0552	9.3654	3.55	0.7645	0.3448	73.42
Thamnophilidae	*Myrmotherula grisea*	Neotropics	0	-1.1815	8.8267	4.07	0.7087	0.1595	35.76
Thamnophilidae	*Myrmotherula gularis*	Neotropics	0	0.3245	5.3986	3.22	0.7172	0.2435	82.37
Thamnophilidae	*Myrmotherula guttata*	Neotropics	0	0.6431	7.3432	4.35	0.7428	0.3318	55.76
Thamnophilidae	*Myrmotherula gutturalis*	Neotropics	0	-0.2235	-	2.99	0.8118	0.5234	66.75
Thamnophilidae	*Myrmotherula hauxwelli*	Neotropics	0	-4.5829	4.2074	4.02	0.7310	0.1394	38.97
Thamnophilidae	*Myrmotherula ignota*	Neotropics	0	0.1126	-	3.06	0.7363	0.7929	66.89
Thamnophilidae	*Myrmotherula iheringi*	Neotropics	0	-0.2950	-	3.13	0.7890	0.3506	33.99
Thamnophilidae	*Myrmotherula klagesi*	Neotropics	0	-0.0457	-	4.05	0.8348	0.8605	9.21
Thamnophilidae	*Myrmotherula longicauda*	Neotropics	0	0.0347	6.9732	3.58	0.7526	0.3476	83.91
Thamnophilidae	*Myrmotherula longipennis*	Neotropics	0	-4.4852	9.5838	4.07	0.7286	0.1323	33.20
Thamnophilidae	*Myrmotherula menetriesii*	Neotropics	1	0.1880	4.8894	3.09	0.5985	0.0000	0.00
Thamnophilidae	*Myrmotherula minor*	Neotropics	0	-0.0994	12.1123	3.74	0.7791	0.3368	71.01
Thamnophilidae	*Myrmotherula multostriata*	Neotropics	0	0.0682	8.9526	3.46	0.7528	0.3613	81.95
Thamnophilidae	*Myrmotherula pacifica*	Neotropics	0	-0.1184	-	3.95	0.8392	0.5142	42.41
Thamnophilidae	*Myrmotherula schisticolor*	Neotropics	0	0.0333	-	3.88	0.8181	0.5845	62.89
Thamnophilidae	*Myrmotherula sclateri*	Neotropics	0	0.2590	-	4.04	0.8219	0.5988	2.92
Thamnophilidae	*Myrmotherula snowi*	Neotropics	0	-0.1221	-	3.79	0.8478	0.3718	33.80
Thamnophilidae	*Myrmotherula sunensis*	IndoMalay	1	0.1720	5.0552	2.76	0.6221	0.9901	90.00
Thamnophilidae	*Myrmotherula surinamensis*	IndoMalay	0	0.1492	6.8245	2.90	0.7261	0.4632	53.95
Thamnophilidae	*Myrmotherula unicolor*	Palearctic	0	-0.2993	14.1682	3.56	0.8380	0.6408	23.59
Thamnophilidae	*Myrmotherula urosticta*	IndoMalay	0	0.0616	5.5600	2.78	0.7228	0.1112	55.00
Thamnophilidae	*Neoctantes niger*	Afrotropics	0	-0.0197	-	3.35	0.7688	0.6746	23.27
Thamnophilidae	*Percnostola arenarum*	Afrotropics	0	-0.2905	-	3.86	0.8021	0.6547	36.62
Thamnophilidae	*Percnostola lophotes*	Palearctic	0	-0.5342	0.0000	4.13	0.7507	0.2252	8.20
Thamnophilidae	*Percnostola rufifrons*	Palearctic	0	-0.4801	-	4.30	0.8811	0.4300	9.36
Thamnophilidae	*Phaenostictus mcleannani*	Australasia	0	-0.3148	8.9752	2.84	0.7305	0.1778	24.25
Thamnophilidae	*Phlegopsis borbae*	Neotropics	0	0.0730	7.3201	3.60	0.7768	0.4245	86.51
Thamnophilidae	*Phlegopsis erythroptera*	Neotropics	0	0.0369	-	4.04	0.8221	0.5604	65.19
Thamnophilidae	*Phlegopsis nigromaculata*	Neotropics	0	-0.1772	-	3.01	0.7228	0.2798	60.16
Thamnophilidae	*Pithys albifrons*	Neotropics	0	-0.8553	4.3343	3.44	0.7069	0.5957	25.95
Thamnophilidae	*Pithys castaneus*	Neotropics	0	-30.3203	-	4.21	0.6906	0.2535	21.71
Thamnophilidae	*Pygiptila stellaris*	Australasia	0	-2.2103	0.0000	2.60	0.7223	0.0864	9.74
Thamnophilidae	*Pyriglena atra*	Neotropics	0	-0.1567	-	3.80	0.8171	0.3913	59.87
Thamnophilidae	*Pyriglena leuconota*	Neotropics	0	0.3147	3.3566	4.00	0.8151	0.7963	67.48
Thamnophilidae	*Pyriglena leucoptera*	Neotropics	0	-0.2584	6.5487	3.10	0.7710	0.2700	29.85
Thamnophilidae	*Rhegmatorhina berlepschi*	IndoMalay	1	-0.1477	5.2625	2.58	0.6221	0.2766	70.83
Thamnophilidae	*Rhegmatorhina cristata*	IndoMalay	0	0.1382	5.2873	2.77	0.7240	0.0979	53.76
Thamnophilidae	*Rhegmatorhina gymnops*	IndoMalay	0	-0.0249	-	2.64	0.7291	0.2179	67.85
Thamnophilidae	*Rhegmatorhina hoffmannsi*	IndoMalay	1	0.2477	7.5724	3.19	0.7352	0.6013	84.02
Thamnophilidae	*Rhegmatorhina melanosticta*	Palearctic	0	-0.3869	13.4816	4.08	0.7884	0.4086	39.22
Thamnophilidae	*Rhopornis ardesiacus*	Neotropics	0	-0.0519	-	3.89	0.7730	0.3846	5.71
Thamnophilidae	*Sakesphorus bernardi*	Australasia	0	-0.1384	-	2.91	0.7469	0.4984	68.10
Thamnophilidae	*Sakesphorus bernardi*	Australasia	0	-0.1384	-	2.91	0.7469	-	-
Thamnophilidae	*Sakesphorus canadensis*	Afrotropics	0	-0.1676	4.5844	3.04	0.7256	0.1560	76.49
Thamnophilidae	*Sakesphorus cristatus*	Nearctic	0	-0.7606	-	4.26	0.8115	0.6924	60.10
Thamnophilidae	*Sakesphorus luctuosus*	Neotropics	0	-0.1232	6.2064	4.08	0.7750	0.4310	65.77
Thamnophilidae	*Sakesphorus melanonotus*	Palearctic	0	-0.4417	10.5487	4.13	0.8595	0.4018	47.02
Thamnophilidae	*Sakesphorus melanothorax*	Australasia	0	-0.5079	0.0000	3.00	0.7602	0.4725	6.71
Thamnophilidae	*Schistocichla brunneiceps*	Australasia	0	-0.0763	-	2.78	0.7670	0.2731	23.50
Thamnophilidae	*Schistocichla caurensis*	IndoMalay	0	-0.1448	-	3.64	0.8005	0.3376	21.67
Thamnophilidae	*Schistocichla humaythae*	IndoMalay	0	-0.0414	-	2.87	0.8050	0.8126	45.99
Thamnophilidae	*Schistocichla leucostigma*	Australasia	0	-0.3372	-	3.28	0.8406	0.8166	7.62
Thamnophilidae	*Schistocichla rufifacies*	Australasia	0	-0.0623	-	2.79	0.7664	0.2240	23.09
Thamnophilidae	*Schistocichla saturata*	Australasia	0	-0.0275	-	2.73	0.7335	0.1966	31.69
Thamnophilidae	*Schistocichla schistacea*	IndoMalay	0	-0.0392	-	2.63	0.6784	0.6648	80.50
Thamnophilidae	*Sclateria naevia*	Australasia	0	-0.0270	-	3.02	0.7985	0.6452	26.82
Thamnophilidae	*Stymphalornis acutirostris*	Neotropics	0	0.0260	2.4548	3.25	0.7654	0.3106	50.45
Thamnophilidae	*Taraba major*	Afrotropics	0	0.0027	13.2225	3.46	0.7639	0.3011	75.07
Thamnophilidae	*Terenura callinota*	Neotropics	0	-13.6630	5.1300	3.55	0.7396	0.2298	65.21
Thamnophilidae	*Terenura humeralis*	Neotropics	0	0.0552	-	4.06	0.7760	0.4888	1.89
Thamnophilidae	*Terenura maculata*	Neotropics	0	-0.5188	-	3.20	0.7861	0.4294	34.82
Thamnophilidae	*Terenura sharpei*	Neotropics	0	0.0254	-	4.05	0.8319	0.7463	12.84
Thamnophilidae	*Terenura sicki*	Neotropics	0	-0.1848	-	3.83	0.8198	0.5100	31.73

Family	Species	Realm	Threat	Latitude	Elevation	Anomaly	Size	Shape	Orientation
Thamnophilidae	*Terenura spodioptila*	Neotropics	0	-0.4641	-	2.83	0.7914	0.2505	41.30
Thamnophilidae	*Thamnistes anabatinus*	Neotropics	0	-0.4441	-	3.42	0.8274	0.6914	56.27
Thamnophilidae	*Thamnomanes ardesiacus*	Neotropics	0	-0.5022	-	2.52	0.7745	0.4694	87.80
Thamnophilidae	*Thamnomanes caesius*	Neotropics	0	-0.6644	1.3416	3.18	0.6784	0.3206	70.67
Thamnophilidae	*Thamnomanes saturninus*	Nearctic	0	-0.7250	-	3.91	0.8337	0.2884	41.24
Thamnophilidae	*Thamnomanes schistogynus*	Neotropics	0	-0.6431	-	2.18	0.7803	0.5339	89.06
Thamnophilidae	*Thamnophilus aethiops*	Afrotropics	0	-0.0373	6.0578	3.48	0.7994	0.8336	80.05
Thamnophilidae	*Thamnophilus amazonicus*	Palearctic	0	-0.3551	10.4984	3.77	0.8432	0.8929	72.29
Thamnophilidae	*Thamnophilus ambiguus*	Neotropics	0	-0.5495	-	3.29	0.7905	0.6059	19.84
Thamnophilidae	*Thamnophilus aroyae*	Neotropics	0	-0.1877	3.6279	3.95	0.8339	0.5814	28.43
Thamnophilidae	*Thamnophilus atrinucha*	Neotropics	0	-0.0682	-	3.12	0.7413	0.4826	54.00
Thamnophilidae	*Thamnophilus bridgesi*	Neotropics	0	-0.0494	-	4.00	0.8380	0.7149	26.80
Thamnophilidae	*Thamnophilus caerulescens*	Neotropics	0	-0.0127	-	4.11	0.8055	0.6800	53.70
Thamnophilidae	*Thamnophilus cryptoleucus*	Neotropics	0	0.1213	-	4.06	0.7738	0.7400	71.42
Thamnophilidae	*Thamnophilus divisorius*	Neotropics	0	-0.2218	-	3.72	0.8315	0.6872	38.82
Thamnophilidae	*Thamnophilus doliatus*	Neotropics	0	-0.0741	-	4.01	0.8260	0.5847	11.76
Thamnophilidae	*Thamnophilus insignis*	Afrotropics	0	-0.4917	-	3.82	0.7953	0.5816	35.02
Thamnophilidae	*Thamnophilus murinus*	Palearctic	0	-0.6732	-	4.35	0.8625	0.2845	5.22
Thamnophilidae	*Thamnophilus nigriceps*	Australasia	0	-0.2496	-	3.17	0.8051	0.8124	29.43
Thamnophilidae	*Thamnophilus nigrocinereus*	Palearctic	0	-0.5968	-	4.38	0.8472	0.1908	0.71
Thamnophilidae	*Thamnophilus palliatus*	Australasia	0	-0.6812	0.0000	3.14	0.8157	0.2385	13.68
Thamnophilidae	*Thamnophilus pelzelni*	Australasia	0	-0.4551	-	2.33	0.7331	0.2569	44.63
Thamnophilidae	*Thamnophilus praecox*	Australasia	0	-0.4520	-	3.40	0.8140	0.4178	37.16
Thamnophilidae	*Thamnophilus punctatus*	Australasia	0	-0.4730	-	3.43	0.8337	0.4912	10.89
Thamnophilidae	*Thamnophilus ruficapillus*	Neotropics	0	-0.0562	0.9973	3.99	0.7781	0.2819	37.00
Thamnophilidae	*Thamnophilus schistaceus*	Australasia	0	-0.1290	-	2.63	0.7104	0.2531	33.53
Thamnophilidae	*Thamnophilus stictocephalus*	Australasia	0	0.0116	-	2.85	0.7455	0.2974	20.76
Thamnophilidae	*Thamnophilus sticturus*	Australasia	0	-0.0337	5.2611	2.73	0.7406	0.2050	28.94
Thamnophilidae	*Thamnophilus tenuepunctatus*	Neotropics	0	0.8001	8.5845	3.91	0.6997	0.0664	42.97
Thamnophilidae	*Thamnophilus torquatus*	Neotropics	0	0.0133	2.9490	3.65	0.7748	0.4319	85.33
Thamnophilidae	*Thamnophilus unicolor*	Neotropics	1	-0.4679	4.9162	3.57	0.6221	0.2290	36.14
Thamnophilidae	*Thamnophilus zarumae*	Neotropics	0	0.1212	-	3.93	0.7937	0.6310	69.07
Thamnophilidae	*Willisornis poecilinotus*	Afrotropics	0	-0.2028	8.6321	3.50	0.8617	0.3637	17.44
Thamnophilidae	*Xenornis setifrons*	Neotropics	0	-0.4151	11.1566	3.61	0.8933	0.3186	5.09
Thinocoridae	*Attagis gayi*	Neotropics	0	0.0000	3.4008	4.15	0.6302	0.6369	0.00
Thinocoridae	*Attagis malouinus*	Neotropics	0	0.1964	7.6895	3.52	0.7256	0.2131	81.02
Thinocoridae	*Thinocorus orbignyianus*	Neotropics	0	-0.2537	-	3.29	0.7604	-	-
Thinocoridae	*Thinocorus orbignyianus*	Neotropics	0	-0.2537	-	3.29	0.7604	0.3395	59.56
Thinocoridae	*Thinocorus rumicivorus*	Neotropics	0	-0.3795	5.7524	3.07	0.7396	0.2895	31.42
Thraupidae	*Anisognathus igniventris*	Neotropics	0	2.7289	5.5042	3.72	0.7599	0.4062	72.76
Thraupidae	*Anisognathus lacrymosus*	Neotropics	0	0.0606	8.7325	3.55	0.7472	0.2762	79.28
Thraupidae	*Anisognathus melanogenys*	Neotropics	0	0.0730	6.0085	3.32	0.6120	0.9804	90.00
Thraupidae	*Anisognathus melanogenys*	Neotropics	0	0.0730	6.0085	3.32	0.6120	-	-
Thraupidae	*Anisognathus notabilis*	Neotropics	0	0.2433	7.0277	3.02	0.6891	0.1390	71.71
Thraupidae	*Anisognathus somptuosus*	Neotropics	0	0.0035	7.5858	3.57	0.7645	0.4381	74.24
Thraupidae	*Bangsia arcaei*	Afrotropics	1	3.0895	-	3.45	0.7971	0.4784	88.04
Thraupidae	*Bangsia aureocincta*	Afrotropics	1	0.0564	0.0000	3.56	0.8166	0.1787	6.15
Thraupidae	*Bangsia edwardsi*	Afrotropics	1	-0.1549	-	3.58	0.8162	0.4982	68.48
Thraupidae	*Bangsia melanochlamys*	IndoMalay	0	-0.2928	11.8832	3.23	0.7700	0.7523	24.39
Thraupidae	*Bangsia rothschildi*	Palearctic	0	-0.6331	5.6895	3.28	0.7935	0.2872	11.10
Thraupidae	*Buthraupis aureodorsalis*	Palearctic	0	-0.7138	10.8223	4.26	0.8454	0.2647	9.60
Thraupidae	*Buthraupis eximia*	Afrotropics	0	-0.4316	-	3.72	0.7929	0.6599	16.55
Thraupidae	*Calochaetes coccineus*	Australasia	1	-0.4414	2.0854	2.42	0.7165	0.2944	41.11
Thraupidae	*Calyptophilus frugivorus*	Australasia	0	0.0522	1.9118	2.59	0.7482	0.5154	14.90
Thraupidae	*Catamblyrhynchus diadema*	Neotropics	1	0.1743	2.0124	3.02	0.6221	0.0912	32.25
Thraupidae	*Chlorochrysa calliparaea*	IndoMalay	0	0.1178	13.6868	2.90	0.7323	0.4098	58.01
Thraupidae	*Chlorochrysa nitidissima*	Palearctic	1	-0.6896	0.0000	4.13	0.8520	0.2039	10.71
Thraupidae	*Chlorochrysa phoenicotis*	Palearctic	0	-0.4777	6.4855	3.87	0.8501	0.5125	30.39
Thraupidae	*Chlorophanes spiza*	Neotropics	0	-0.1965	-	3.75	0.8555	0.5295	42.22
Thraupidae	*Chlorophonia callophrys*	Neotropics	0	-0.2827	6.9223	3.69	0.8610	0.4593	44.28
Thraupidae	*Chlorophonia cyanea*	Neotropics	0	-0.2151	-	3.97	0.8425	0.6655	36.39
Thraupidae	*Chlorophonia flavirostris*	IndoMalay	0	0.0947	14.9514	2.89	0.7367	0.4301	37.97
Thraupidae	*Chlorophonia occipitalis*	Neotropics	0	-20.6477	6.0561	3.82	0.6221	0.2106	78.31
Thraupidae	*Chlorophonia pyrrhophrys*	Neotropics	1	0.1661	7.0612	3.09	0.6859	0.2429	61.98
Thraupidae	*Chlorornis riefferii*	Neotropics	0	-0.1817	9.1007	3.04	0.6804	0.2128	30.89
Thraupidae	*Chlorospingus canigularis*	Neotropics	0	-0.1725	-	3.54	0.8083	0.4204	49.91
Thraupidae	*Chlorospingus flavigularis*	Neotropics	0	-0.1725	6.4085	2.96	0.6740	0.1787	57.41
Thraupidae	*Chlorospingus flavovirens*	Neotropics	0	0.0372	9.4934	3.60	0.7413	0.2034	20.70
Thraupidae	*Chlorospingus inornatus*	Neotropics	0	0.0239	5.8139	3.52	0.7416	0.2770	73.78
Thraupidae	*Chlorospingus ophthalmicus*	IndoMalay	0	-0.0689	3.4577	3.22	0.8185	0.4970	6.78
Thraupidae	*Chlorospingus parvirostris*	IndoMalay	0	-0.0898	4.5201	2.98	0.8123	0.3882	60.81
Thraupidae	*Chlorospingus pileatus*	IndoMalay	0	0.0411	-	2.83	0.7870	0.4666	4.93
Thraupidae	*Chlorospingus semifuscus*	IndoMalay	1	-0.0161	2.8341	2.62	0.7040	0.6488	86.25
Thraupidae	*Chlorospingus tacarcunae*	IndoMalay	0	-0.1506	-	3.27	0.8073	0.4952	3.28

Family	Species	Realm	Threat	Latitude	Elevation	Anomaly	Size	Shape	Orientation
Thraupidae	*Chrysothlypis chrysomelas*	IndoMalay	0	-0.5526	-	3.29	0.8028	0.5146	3.09
Thraupidae	*Chrysothlypis salmoni*	Australasia	0	-0.0597	6.4474	2.78	0.7645	0.2066	22.88
Thraupidae	*Cissopis leverianus*	Australasia	0	-0.5595	9.7854	2.91	0.8016	0.3027	5.92
Thraupidae	*Cnemoscopus rubrirostris*	Australasia	0	-0.5631	-	3.09	0.7472	0.2463	62.05
Thraupidae	*Compsospiza baeri*	Palearctic	0	-0.7733	-	4.28	0.8523	0.4316	1.92
Thraupidae	*Compsospiza garleppi*	IndoMalay	0	-0.2602	10.3184	3.54	0.7745	0.3747	11.92
Thraupidae	*Compsothraupis loricata*	IndoMalay	1	-0.0901	4.7610	3.26	0.7446	0.5456	9.79
Thraupidae	*Conirostrum albifrons*	Afrotropics	0	0.8617	8.5754	3.22	0.6997	0.4775	44.35
Thraupidae	*Conirostrum bicolor*	IndoMalay	1	0.4157	-	2.76	0.6221	0.9901	90.00
Thraupidae	*Conirostrum cinereum*	Afrotropics	0	-0.0610	-	3.28	0.8126	0.3285	13.19
Thraupidae	*Conirostrum ferrugineiventre*	Australasia	0	-0.0735	-	2.71	0.7823	0.3600	32.41
Thraupidae	*Conirostrum leucogenys*	Neotropics	0	0.2504	-	3.10	0.7030	0.3007	83.26
Thraupidae	*Conirostrum margaritae*	Neotropics	0	-0.2351	-	3.53	0.7495	0.1730	61.59
Thraupidae	*Conirostrum rufum*	Neotropics	1	-0.2631	3.6665	4.00	0.7710	0.9012	5.10
Thraupidae	*Conirostrum sitticolor*	Nearctic	0	-0.2488	-	3.96	0.8112	0.4374	43.50
Thraupidae	*Conirostrum speciosum*	Neotropics	0	-0.1598	4.7025	3.75	0.8380	0.4646	38.20
Thraupidae	*Conirostrum tamarugense*	Neotropics	0	-0.1829	7.1160	3.69	0.8361	0.1788	30.25
Thraupidae	*Conothraupis mesoleuca*	Neotropics	1	24.2651	14.3275	4.05	0.6631	0.2153	83.19
Thraupidae	*Conothraupis speculigera*	Neotropics	0	0.1104	2.8835	3.09	0.7378	0.2960	55.16
Thraupidae	*Creurgops dentatus*	Neotropics	0	-0.5436	-	2.86	0.7681	0.8327	36.97
Thraupidae	*Creurgops verticalis*	Neotropics	0	-0.0894	8.7008	3.42	0.7712	0.7274	40.26
Thraupidae	*Cyanerpes caeruleus*	Australasia	0	-0.5533	-	3.34	0.8379	0.5827	7.73
Thraupidae	*Cyanerpes cyaneus*	Palearctic	0	-0.8011	10.9211	3.56	0.8292	0.3318	17.93
Thraupidae	*Cyanerpes lucidus*	Afrotropics	0	-0.0667	6.0514	3.18	0.7611	0.3932	69.79
Thraupidae	*Cyanerpes nitidus*	IndoMalay	0	-0.6776	10.2304	3.39	0.8192	0.3518	3.75
Thraupidae	*Cyanicterus cyanicterus*	Afrotropics	0	-0.1382	11.3894	3.47	0.8516	0.6009	35.05
Thraupidae	*Cypsnagra hirundinacea*	IndoMalay	1	0.2262	0.3218	2.68	0.6120	0.3820	58.28
Thraupidae	*Dacnis albiventris*	Neotropics	0	-0.0359	1.4595	4.05	0.8246	0.6342	21.86
Thraupidae	*Dacnis berlepschi*	Neotropics	0	-0.0026	-	3.14	0.7510	0.5262	62.57
Thraupidae	*Dacnis cayana*	Neotropics	0	0.1323	8.1433	3.69	0.7428	0.3205	74.56
Thraupidae	*Dacnis flaviventer*	Neotropics	0	0.1702	6.1813	3.06	0.6302	0.2782	40.02
Thraupidae	*Dacnis hartlaubi*	Neotropics	0	0.0134	9.2540	3.70	0.7524	0.2466	21.29
Thraupidae	*Dacnis lineata*	Neotropics	0	-0.4417	8.5798	3.14	0.7748	0.4812	29.73
Thraupidae	*Dacnis nigripes*	Neotropics	0	144.9738	1.8160	3.12	0.6302	0.1050	72.69
Thraupidae	*Dacnis venusta*	Nearctic	0	-0.4542	10.7757	3.52	0.7936	0.3063	42.47
Thraupidae	*Dacnis viguieri*	Neotropics	0	2.8336	6.6116	3.69	0.7310	0.1631	81.78
Thraupidae	*Delothraupis castaneoventris*	Neotropics	0	-0.1942	-	3.86	0.8512	0.5520	34.40
Thraupidae	*Diglossa albilatera*	Australasia	0	0.2273	-	2.48	0.6370	0.1926	10.82
Thraupidae	*Diglossa baritula*	Neotropics	0	0.1494	-	4.02	0.8153	0.5769	3.92
Thraupidae	*Diglossa brunneiventris*	Afrotropics	0	-0.1551	8.9190	3.54	0.8609	0.7482	37.83
Thraupidae	*Diglossa caerulescens*	IndoMalay	0	-0.0482	-	3.11	0.8271	0.5569	35.38
Thraupidae	*Diglossa carbonaria*	IndoMalay	0	-0.1930	-	3.26	0.7913	0.3472	23.14
Thraupidae	*Diglossa cyanea*	Afrotropics	0	0.0452	-	3.22	0.8006	0.1800	9.99
Thraupidae	*Diglossa duidae*	IndoMalay	0	-0.0721	-	2.65	0.7128	0.2680	74.97
Thraupidae	*Diglossa glauca*	Australasia	0	-0.2595	-	3.02	0.8048	0.3876	60.58
Thraupidae	*Diglossa gloriosa*	IndoMalay	0	-0.1099	-	3.34	0.8038	0.6271	73.43
Thraupidae	*Diglossa gloriosissima*	Australasia	0	-0.0803	-	2.59	0.6891	0.1686	0.56
Thraupidae	*Diglossa humeralis*	Afrotropics	0	-0.0765	-	3.18	0.7614	0.3874	70.15
Thraupidae	*Diglossa indigotica*	IndoMalay	0	-0.2954	4.8967	3.24	0.8388	0.7953	9.38
Thraupidae	*Diglossa lafresnayii*	IndoMalay	0	-0.4524	-	3.39	0.8364	0.7843	54.23
Thraupidae	*Diglossa major*	Afrotropics	0	-0.1322	-	3.38	0.8287	0.4051	31.13
Thraupidae	*Diglossa mystacalis*	IndoMalay	0	-0.6024	4.0717	3.43	0.8444	0.4698	11.14
Thraupidae	*Diglossa plumbea*	Australasia	0	0.6196	-	2.52	0.5788	0.0000	90.00
Thraupidae	*Diglossa sittoides*	Australasia	0	-0.1346	-	2.73	0.7069	0.4847	47.70
Thraupidae	*Diglossa venezuelensis*	IndoMalay	0	-0.0702	-	3.11	0.8319	0.4771	33.07
Thraupidae	*Dubusia taeniata*	Neotropics	0	0.1566	3.3968	4.21	0.8046	0.5777	3.46
Thraupidae	*Eucometis penicillata*	IndoMalay	1	-0.0068	-	2.66	0.6997	0.4736	75.00
Thraupidae	*Euphonia affinis*	Nearctic	0	-0.1348	8.9320	3.85	0.7843	0.2251	37.23
Thraupidae	*Euphonia anneae*	Australasia	0	-0.0806	12.4639	2.82	0.7382	0.1595	23.93
Thraupidae	*Euphonia cayennensis*	Neotropics	0	-0.4119	2.9350	2.30	0.7172	0.2592	87.29
Thraupidae	*Euphonia chalybea*	Australasia	0	0.1216	-	3.08	0.7120	0.4590	7.98
Thraupidae	*Euphonia chlorotica*	Australasia	0	-0.2407	5.3820	2.85	0.7310	0.1620	23.47
Thraupidae	*Euphonia concinna*	Neotropics	1	-0.0066	2.5657	4.14	0.7019	0.2856	82.02
Thraupidae	*Euphonia cyanocephala*	Australasia	1	-0.0066	5.7871	2.48	0.6690	0.5741	71.12
Thraupidae	*Euphonia elegantissima*	Neotropics	0	-0.0759	-	3.27	0.7425	0.5097	66.46
Thraupidae	*Euphonia finschi*	IndoMalay	0	-0.0197	-	3.04	0.6961	0.2268	62.91
Thraupidae	*Euphonia fulvicrissa*	IndoMalay	0	-0.0181	-	2.82	0.7558	0.6112	0.50
Thraupidae	*Euphonia gouldi*	IndoMalay	0	-0.0684	-	2.66	0.7446	0.3330	88.92
Thraupidae	*Euphonia hirundinacea*	IndoMalay	0	0.1899	3.6645	2.76	0.6302	0.7374	46.14
Thraupidae	*Euphonia imitans*	IndoMalay	0	-0.1581	8.3018	3.23	0.8266	0.5932	65.19
Thraupidae	*Euphonia jamaica*	Neotropics	0	-1.0614	-	2.47	0.5985	0.0000	0.00
Thraupidae	*Euphonia laniirostris*	Australasia	1	-0.1478	5.1625	2.58	0.6302	0.1134	34.50
Thraupidae	*Euphonia luteicapilla*	IndoMalay	0	0.0547	2.0641	2.84	0.7869	0.4519	4.68
Thraupidae	*Euphonia mesochrysa*	Neotropics	0	-0.2048	6.8429	3.74	0.8243	0.7255	27.33

Family	Species	Realm	Threat	Latitude	Elevation	Anomaly	Size	Shape	Orientation
Thraupidae	*Euphonia minuta*	Nearctic	1	-0.5693	-	4.34	0.8387	0.1299	9.86
Thraupidae	*Euphonia musica*	Nearctic	0	-0.8564	-	4.12	0.8303	0.5778	5.88
Thraupidae	*Euphonia pectoralis*	Neotropics	1	0.6123	9.4851	3.27	0.6120	0.0000	0.00
Thraupidae	*Euphonia plumbea*	Neotropics	0	-0.0321	8.9734	3.50	0.7413	0.2206	33.03
Thraupidae	*Euphonia rufiventris*	Neotropics	0	-0.1307	6.0580	3.01	0.6690	0.2040	31.26
Thraupidae	*Euphonia saturata*	Neotropics	1	0.7248	8.6932	3.55	0.6763	0.2744	19.46
Thraupidae	*Euphonia trinitatis*	Neotropics	0	-0.0397	-	3.51	0.7770	0.2665	25.83
Thraupidae	*Euphonia violacea*	Neotropics	0	-0.0050	4.8206	2.98	0.6935	0.1602	16.35
Thraupidae	*Euphonia xanthogaster*	Neotropics	0	-0.0639	-	3.88	0.7885	0.4467	30.61
Thraupidae	*Hemispingus atropileus*	Neotropics	0	-0.5337	6.2349	2.97	0.7750	0.4600	34.49
Thraupidae	*Hemispingus calophrys*	Neotropics	0	0.0938	1.3776	4.06	0.8192	0.7582	9.13
Thraupidae	*Hemispingus frontalis*	Neotropics	0	-20.3641	4.8358	4.09	0.7256	0.3455	51.69
Thraupidae	*Hemispingus goeringi*	Neotropics	0	-17.2469	3.9528	3.44	0.7128	0.2485	78.29
Thraupidae	*Hemispingus melanotis*	Neotropics	0	-17.2469	7.8369	2.97	0.6859	0.1680	63.63
Thraupidae	*Hemispingus parodii*	Neotropics	0	0.0456	7.9170	3.06	0.7277	0.5257	77.81
Thraupidae	*Hemispingus reyi*	Neotropics	0	0.0548	9.0906	3.74	0.7709	0.3840	88.90
Thraupidae	*Hemispingus rufosuperciliaris*	Neotropics	0	0.0300	5.6121	3.46	0.7524	0.2862	89.22
Thraupidae	*Hemispingus superciliaris*	Neotropics	0	0.2371	3.6241	3.76	0.7763	0.7162	69.83
Thraupidae	*Hemispingus trifasciatus*	Neotropics	0	0.1796	6.7770	4.32	0.7096	0.2566	7.62
Thraupidae	*Hemispingus verticalis*	Afrotropics	0	-0.1067	5.7116	3.40	0.8212	0.3118	24.34
Thraupidae	*Hemispingus xanthophthalmus*	Neotropics	0	-0.0129	5.6568	3.70	0.7683	0.1656	33.47
Thraupidae	*Hemithraupis flavicollis*	Neotropics	0	-0.0145	-	3.97	0.8419	0.5512	34.12
Thraupidae	*Hemithraupis guira*	Neotropics	0	-0.1946	-	3.70	0.7947	0.4319	44.90
Thraupidae	*Hemithraupis ruficapilla*	IndoMalay	1	-0.0814	2.7331	2.97	0.6997	0.4579	17.45
Thraupidae	*Heterospingus rubrifrons*	Neotropics	0	0.1130	6.8942	3.65	0.7389	0.2845	71.65
Thraupidae	*Heterospingus xanthopygius*	Neotropics	0	0.2352	-	4.12	0.7803	0.6244	20.50
Thraupidae	*Iridophanes pulcherrimus*	Afrotropics	0	-0.0195	-	3.27	0.8161	0.3076	13.53
Thraupidae	*Iridosornis analis*	Afrotropics	0	-0.1671	-	3.18	0.7710	0.1198	7.99
Thraupidae	*Iridosornis jelskii*	Afrotropics	0	4.1439	7.4210	3.40	0.7406	0.7541	66.89
Thraupidae	*Iridosornis porphyrocephalus*	Afrotropics	0	0.2284	5.1454	3.09	0.7488	0.2839	11.74
Thraupidae	*Iridosornis reinhardti*	Afrotropics	0	0.0149	-	3.22	0.8004	0.1896	13.17
Thraupidae	*Lamprospiza melanoleuca*	Afrotropics	1	0.0000	6.8682	3.15	0.6120	0.3611	79.10
Thraupidae	*Lanio aurantius*	Afrotropics	0	-0.0717	-	3.88	0.7974	0.5493	18.08
Thraupidae	*Lanio fulvus*	Afrotropics	0	-0.1472	-	4.23	0.7950	0.5105	32.55
Thraupidae	*Lanio leucothorax*	Afrotropics	0	0.2698	-	3.60	0.8133	0.1301	4.05
Thraupidae	*Lanio versicolor*	Afrotropics	0	0.0043	-	3.43	0.8104	0.1311	8.01
Thraupidae	*Mitrospingus cassinii*	IndoMalay	0	-0.0283	4.3707	2.64	0.7310	0.2278	67.50
Thraupidae	*Mitrospingus oleagineus*	IndoMalay	0	-0.0246	2.2915	2.84	0.7909	0.5866	19.97
Thraupidae	*Nemosia pileata*	Afrotropics	0	-0.0413	-	3.23	0.8041	0.2213	11.14
Thraupidae	*Nemosia rourei*	Afrotropics	1	-0.1506	0.0000	3.64	0.8575	0.6732	41.60
Thraupidae	*Neothraupis fasciata*	Afrotropics	0	-0.2454	-	3.14	0.7192	0.3179	68.92
Thraupidae	*Nephelornis oneilli*	Afrotropics	0	4.0358	9.3458	3.45	0.7144	0.2767	83.47
Thraupidae	*Nesospingus speculiferus*	IndoMalay	0	-0.0758	-	2.91	0.8024	0.7044	41.45
Thraupidae	*Orchesticus abeillei*	Neotropics	0	-0.0451	9.8558	3.93	0.7793	0.3053	57.49
Thraupidae	*Oreomanes fraseri*	Neotropics	0	0.1047	1.7580	4.09	0.8255	0.8738	30.99
Thraupidae	*Orthogonys chloricterus*	Neotropics	0	-0.2148	2.6393	3.42	0.7803	0.2304	26.22
Thraupidae	*Paroaria baeri*	Australasia	0	-0.0550	4.5518	2.56	0.6523	0.1875	30.24
Thraupidae	*Paroaria capitata*	Australasia	0	-0.3997	-	2.91	0.8035	0.6809	6.88
Thraupidae	*Paroaria coronata*	Australasia	0	-0.0807	1.0089	2.48	0.5985	0.1888	68.65
Thraupidae	*Paroaria dominicana*	IndoMalay	0	-0.0143	-	2.64	0.7261	0.2111	67.49
Thraupidae	*Paroaria gularis*	Australasia	0	-0.5158	-	3.35	0.8368	0.5729	6.17
Thraupidae	*Paroaria nigrogenis*	Australasia	0	-0.7543	-	2.79	0.7165	0.5396	12.90
Thraupidae	*Phaenicophilus palmarum*	Australasia	0	0.1504	6.3479	2.76	0.7096	0.2658	23.71
Thraupidae	*Phaenicophilus poliocephalus*	Australasia	0	-0.0647	-	2.82	0.7444	0.1769	24.41
Thraupidae	*Pipraeidea melanonota*	Afrotropics	1	0.1022	9.3949	3.17	0.7542	0.6187	49.35
Thraupidae	*Pyrrhocoma ruficeps*	Neotropics	0	0.0233	6.6353	3.68	0.7572	0.3775	73.51
Thraupidae	*Ramphocelus bresilius*	Nearctic	1	-0.3441	1.3488	3.12	0.7766	0.1614	47.57
Thraupidae	*Ramphocelus carbo*	Neotropics	0	-0.3932	1.0981	3.02	0.7874	0.7134	74.44
Thraupidae	*Ramphocelus costaricensis*	Australasia	0	-0.5900	4.1327	2.54	0.7416	0.1344	2.86
Thraupidae	*Ramphocelus dimidiatus*	Australasia	1	-0.2142	0.0000	2.51	0.6784	0.3246	2.97
Thraupidae	*Ramphocelus flammigerus*	Australasia	1	-0.4458	3.5393	2.61	0.7500	0.3987	9.62
Thraupidae	*Ramphocelus passerinii*	Nearctic	0	-0.6651	0.1921	3.23	0.7314	0.1092	44.30
Thraupidae	*Ramphocelus sanguinolentus*	Australasia	0	-0.5640	-	3.65	0.7937	0.2143	12.54
Thraupidae	*Rhodinocichla rosea*	Neotropics	1	-0.7217	5.9726	3.09	0.5985	0.0000	0.00
Thraupidae	*Schistochlamys melanopis*	Australasia	0	-0.5730	-	2.83	0.7904	0.3000	7.43
Thraupidae	*Sericossypha albocristata*	Afrotropics	0	-0.0753	-	3.16	0.7550	0.4276	70.75
Thraupidae	*Spindalis dominicensis*	Australasia	0	0.8448	-	2.50	0.6370	0.3679	33.40
Thraupidae	*Spindalis nigricephala*	Australasia	0	-0.0574	-	2.79	0.7569	0.2019	22.53
Thraupidae	*Spindalis portoricensis*	Australasia	0	0.0975	-	2.74	0.7179	0.1673	17.50
Thraupidae	*Spindalis zena*	Neotropics	0	-4.6281	6.9636	3.59	0.7300	0.2921	86.68
Thraupidae	*Stephanophorus diadematus*	Palearctic	0	-0.8913	-	3.96	0.7928	0.2215	8.37
Thraupidae	*Tachyphonus coronatus*	Afrotropics	0	-0.0260	8.4242	3.48	0.7917	0.2952	67.16
Thraupidae	*Tachyphonus cristatus*	Palearctic	0	-0.7814	-	4.25	0.8668	0.6217	13.39
Thraupidae	*Tachyphonus delatrii*	Afrotropics	0	0.2848	-	3.28	0.7344	0.7522	47.06

Family	Species	Realm	Threat	Latitude	Elevation	Anomaly	Size	Shape	Orientation
Thraupidae	*Tachyphonus luctuosus*	Afrotropics	0	-0.1488	5.1304	3.84	0.8359	0.1825	3.26
Thraupidae	*Tachyphonus phoenicius*	Afrotropics	0	-0.0812	8.4899	3.52	0.8554	0.6821	36.96
Thraupidae	*Tachyphonus rufiventer*	IndoMalay	0	-0.2607	-	3.44	0.8449	0.5480	7.15
Thraupidae	*Tachyphonus rufus*	Palearctic	0	-0.7676	-	4.29	0.8623	0.3582	4.31
Thraupidae	*Tachyphonus surinamus*	Afrotropics	0	0.1189	8.2353	3.53	0.8346	0.2014	5.29
Thraupidae	*Tangara argyrofenges*	Neotropics	0	-0.5387	-	2.91	0.7727	0.4106	24.16
Thraupidae	*Tangara arthus*	IndoMalay	0	-0.1744	8.8413	3.17	0.8312	0.7860	31.98
Thraupidae	*Tangara cabanisi*	Palearctic	0	-0.7012	8.3854	4.84	0.8652	0.0629	0.48
Thraupidae	*Tangara callophrys*	Neotropics	0	-0.0543	3.2993	3.38	0.7862	0.3762	32.47
Thraupidae	*Tangara cayana*	Nearctic	0	-0.6616	7.3193	3.92	0.7969	0.2964	47.39
Thraupidae	*Tangara chilensis*	IndoMalay	0	-0.1314	-	3.39	0.8044	0.6938	84.73
Thraupidae	*Tangara chrysotis*	Neotropics	0	-0.7281	6.6292	2.27	0.7681	0.2284	87.34
Thraupidae	*Tangara cyanicollis*	IndoMalay	0	-0.0043	1.7117	2.94	0.7698	0.3248	63.39
Thraupidae	*Tangara cyanocephala*	Palearctic	0	-0.3471	5.3799	4.93	0.8573	0.1811	3.40
Thraupidae	*Tangara cyanoptera*	Nearctic	0	-0.6245	9.6258	4.13	0.8371	0.5214	12.06
Thraupidae	*Tangara cyanotis*	Neotropics	0	-0.1376	5.9162	3.82	0.8473	0.3702	37.47
Thraupidae	*Tangara cyanoventris*	Afrotropics	0	-0.1492	14.7396	3.45	0.8469	0.6605	32.10
Thraupidae	*Tangara desmaresti*	IndoMalay	1	0.0918	6.1167	3.11	0.6428	0.3485	68.99
Thraupidae	*Tangara dowii*	IndoMalay	0	-0.0700	7.1970	2.92	0.6428	0.4185	88.40
Thraupidae	*Tangara fastuosa*	Australasia	0	-0.5105	-	3.39	0.8085	0.5790	58.19
Thraupidae	*Tangara florida*	Afrotropics	0	-0.2105	12.2356	3.78	0.8485	0.8109	54.56
Thraupidae	*Tangara fucosa*	Neotropics	0	-0.1286	-	3.56	0.7550	0.1737	61.79
Thraupidae	*Tangara guttata*	Neotropics	1	-0.5086	-	2.38	0.7558	0.7659	54.69
Thraupidae	*Tangara gyrola*	Neotropics	0	-0.5944	-	2.64	0.7937	0.4019	83.80
Thraupidae	*Tangara heinei*	Nearctic	0	-0.4897	7.9859	4.01	0.8367	0.6465	59.43
Thraupidae	*Tangara icterocephala*	Neotropics	0	0.0733	3.1355	3.94	0.8193	0.6548	26.23
Thraupidae	*Tangara inornata*	Nearctic	0	-0.8211	-	4.22	0.8364	0.7087	8.67
Thraupidae	*Tangara johannae*	Neotropics	0	-0.3517	7.3956	3.34	0.8275	0.5325	44.30
Thraupidae	*Tangara labradorides*	IndoMalay	1	0.4535	4.0259	2.76	0.6370	0.9166	89.25
Thraupidae	*Tangara larvata*	IndoMalay	0	-0.0900	-	3.08	0.7802	0.3840	40.87
Thraupidae	*Tangara lavinia*	Palearctic	0	-1.1256	-	4.15	0.8289	0.4618	16.03
Thraupidae	*Tangara mexicana*	IndoMalay	0	-0.4779	-	3.27	0.8232	0.6078	14.83
Thraupidae	*Tangara meyerdeschauenseei*	IndoMalay	0	-0.0861	-	3.24	0.8179	0.4149	4.44
Thraupidae	*Tangara nigrocincta*	IndoMalay	1	-0.4467	5.4825	2.71	0.7128	0.1436	11.08
Thraupidae	*Tangara nigroviridis*	IndoMalay	0	-0.2250	-	3.15	0.8119	0.7760	32.57
Thraupidae	*Tangara palmeri*	IndoMalay	0	-0.1114	-	3.61	0.8192	0.6897	0.73
Thraupidae	*Tangara parzudakii*	Palearctic	0	-0.9152	4.1129	3.46	0.7240	0.2590	62.58
Thraupidae	*Tangara peruviana*	Palearctic	0	-1.0739	-	4.41	0.8332	0.3243	1.61
Thraupidae	*Tangara phillipsi*	IndoMalay	0	-0.5243	-	3.27	0.7964	0.6140	12.20
Thraupidae	*Tangara preciosa*	IndoMalay	0	-0.6741	-	3.25	0.7728	0.3890	17.99
Thraupidae	*Tangara punctata*	Palearctic	0	-1.0526	-	4.16	0.8162	0.4673	17.61
Thraupidae	*Tangara ruficervix*	Palearctic	0	-0.4648	-	3.56	0.7778	0.5893	3.23
Thraupidae	*Tangara rufigenis*	Palearctic	0	-0.5761	-	4.27	0.8784	0.2371	6.45
Thraupidae	*Tangara rufigula*	Neotropics	1	-0.8544	0.0249	2.42	0.6120	0.3739	73.86
Thraupidae	*Tangara schrankii*	Neotropics	0	0.0634	1.2726	3.85	0.7817	0.2864	5.46
Thraupidae	*Tangara seledon*	Neotropics	0	-0.3533	9.7056	3.66	0.8349	0.6173	40.07
Thraupidae	*Tangara varia*	Neotropics	0	0.0015	2.1896	4.00	0.8246	0.6145	19.51
Thraupidae	*Tangara vassorii*	Neotropics	0	-0.1498	2.7152	4.18	0.7798	0.5708	16.79
Thraupidae	*Tangara velia*	Neotropics	0	-0.5151	5.2097	3.03	0.8028	0.4653	84.45
Thraupidae	*Tangara viridicollis*	Neotropics	0	0.0093	0.1758	3.16	0.7078	0.2660	34.38
Thraupidae	*Tangara vitriolina*	Palearctic	0	-0.6525	-	4.90	0.8668	0.0614	0.45
Thraupidae	*Tangara xanthocephala*	IndoMalay	0	-0.0936	-	3.14	0.8367	0.6530	30.02
Thraupidae	*Tangara xanthogastra*	Afrotropics	1	-0.0425	7.1207	3.36	0.6562	0.1001	72.51
Thraupidae	*Tersina viridis*	Neotropics	0	-0.2524	7.9014	3.08	0.8010	0.5045	34.55
Thraupidae	*Thlypopsis fulviceps*	Neotropics	1	0.1119	2.0317	3.22	0.6598	0.5541	69.81
Thraupidae	*Thlypopsis inornata*	Neotropics	0	0.0329	-	2.97	0.7136	0.4320	54.70
Thraupidae	*Thlypopsis ornata*	Neotropics	0	0.4384	2.6395	2.93	0.6120	0.9804	90.00
Thraupidae	*Thlypopsis pectoralis*	Neotropics	0	-0.0902	4.4153	3.91	0.7659	0.3558	10.96
Thraupidae	*Thlypopsis ruficeps*	Neotropics	0	0.1058	-	3.92	0.8252	0.8066	18.90
Thraupidae	*Thlypopsis sordida*	Neotropics	0	0.4134	5.2441	3.44	0.7461	0.2658	52.58
Thraupidae	*Thraupis abbas*	Neotropics	0	0.0360	5.2458	3.01	0.7300	0.4810	72.14
Thraupidae	*Thraupis bonariensis*	Neotropics	0	-0.1410	-	3.19	0.7335	0.4288	19.96
Thraupidae	*Thraupis cyanocephala*	Neotropics	0	0.2006	2.1831	2.95	0.7040	0.1946	71.00
Thraupidae	*Thraupis cyanoptera*	Neotropics	0	-0.1993	4.1221	3.25	0.7327	0.2367	70.14
Thraupidae	*Thraupis episcopus*	Neotropics	0	-0.1783	-	3.31	0.7663	0.2855	38.34
Thraupidae	*Thraupis glaucocolpa*	Neotropics	0	0.0676	-	3.08	0.7356	0.2831	52.34
Thraupidae	*Thraupis ornata*	Neotropics	0	-0.1707	2.9509	4.03	0.8373	0.7710	17.05
Thraupidae	*Thraupis palmarum*	Neotropics	1	0.0000	1.2948	4.05	0.5788	0.0000	90.00
Thraupidae	*Thraupis sayaca*	Neotropics	0	0.1830	-	4.05	0.8186	0.8272	32.83
Thraupidae	*Trichothraupis melanops*	Nearctic	0	-0.5316	-	4.06	0.8183	0.5181	33.28
Thraupidae	*Wetmorethraupis sterrhopteron*	IndoMalay	0	-0.0922	6.3342	3.15	0.8320	0.6261	19.08
Thraupidae	*Xenodacnis parina*	Neotropics	0	0.1330	-	4.05	0.8295	0.6113	11.77
Threskiornithidae	*Bostrychia carunculata*	Palearctic	0	-1.0265	-	4.92	0.8547	0.0425	0.20

Family	Species	Realm	Threat	Latitude	Elevation	Anomaly	Size	Shape	Orientation
Threskiornithidae	*Bostrychia hagedash*	Palearctic	0	-0.6720	-	4.55	0.7776	0.3501	5.27
Threskiornithidae	*Bostrychia olivacea*	Palearctic	0	-0.7042	1.1376	4.93	0.8592	0.1614	0.06
Threskiornithidae	*Bostrychia rara*	Palearctic	0	-4.3473	16.0408	3.88	0.7584	0.6992	67.50
Threskiornithidae	*Cercibis oxycerca*	IndoMalay	1	-0.4381	-	2.71	0.6961	0.2131	11.26
Threskiornithidae	*Eudocimus albus*	IndoMalay	0	-0.1266	-	3.38	0.8170	0.4294	8.97
Threskiornithidae	*Eudocimus albus*	IndoMalay	0	-0.1266	-	3.38	0.8170	-	-
Threskiornithidae	*Eudocimus ruber*	Afrotropics	0	-0.2570	4.3924	3.55	0.8392	0.8422	21.99
Threskiornithidae	*Geronticus calvus*	Neotropics	1	-0.3156	4.1807	3.85	0.7916	0.6029	7.05
Threskiornithidae	*Geronticus eremita*	Neotropics	0	-2.1342	11.9008	4.16	0.7552	0.5397	82.04
Threskiornithidae	*Lophotibis cristata*	Palearctic	0	-0.9150	-	4.42	0.7770	0.4627	2.96
Threskiornithidae	*Mesembrinibis cayennensis*	Australasia	0	-0.1014	-	2.78	0.7709	0.3038	24.05
Threskiornithidae	*Nipponia nippon*	Australasia	0	-0.8479	-	2.91	0.7789	0.2308	12.85
Threskiornithidae	*Phimosus infuscatus*	Neotropics	0	-0.1002	7.6190	3.61	0.7474	0.4794	48.55
Threskiornithidae	*Platalea ajaja*	Neotropics	0	0.1585	8.3275	3.15	0.7385	0.2146	57.97
Threskiornithidae	*Platalea alba*	Neotropics	0	-0.0378	3.5291	3.97	0.7995	0.4001	30.62
Threskiornithidae	*Platalea flavipes*	Neotropics	0	-0.2769	-	3.61	0.8249	0.5186	29.99
Threskiornithidae	*Platalea leucorodia*	Neotropics	0	-0.0105	-	3.97	0.8424	0.6538	27.40
Threskiornithidae	*Platalea minor*	Neotropics	0	-0.0504	-	3.47	0.7619	0.2686	32.10
Threskiornithidae	*Platalea regia*	Neotropics	0	-0.0413	8.7686	3.61	0.7477	0.5152	86.48
Threskiornithidae	*Plegadis chihi*	Neotropics	0	3.9576	5.5368	4.39	0.6716	0.4359	3.94
Threskiornithidae	*Plegadis falcinellus*	Neotropics	0	-0.1586	4.9165	3.94	0.8377	0.7560	43.13
Threskiornithidae	*Plegadis ridgwayi*	Neotropics	0	0.1087	2.6931	3.34	0.7228	0.4822	54.30
Threskiornithidae	*Pseudibis davisoni*	Palearctic	0	-0.3678	8.6751	3.88	0.8281	0.3440	5.45
Threskiornithidae	*Pseudibis papillosa*	IndoMalay	0	-0.1109	-	3.29	0.8263	0.4424	10.08
Threskiornithidae	*Thaumatibis gigantea*	Neotropics	0	-6.7055	3.6104	3.78	0.7371	0.2715	56.27
Threskiornithidae	*Theristicus caerulescens*	Neotropics	0	0.0827	-	3.93	0.7963	0.4632	31.50
Threskiornithidae	*Theristicus caudatus*	Neotropics	0	-0.2085	-	2.88	0.7618	0.2797	43.51
Threskiornithidae	*Theristicus melanopis*	Neotropics	0	-0.0450	7.1138	4.10	0.7503	0.3791	27.94
Threskiornithidae	*Threskiornis aethiopicus*	Neotropics	1	-0.5104	2.4122	2.69	0.7087	0.3195	38.37
Threskiornithidae	*Threskiornis bernieri*	Neotropics	0	-31.2898	1.6951	4.09	0.6370	0.3250	79.55
Threskiornithidae	*Threskiornis melanocephalus*	Neotropics	0	-0.5196	-	2.76	0.7841	0.4901	46.23
Threskiornithidae	*Threskiornis molucca*	Neotropics	0	0.1327	-	4.03	0.8107	0.7163	11.23
Threskiornithidae	*Threskiornis spinicollis*	Neotropics	0	0.1611	7.2560	3.55	0.7606	0.3454	71.16
Timaliidae	*Actinodura egertoni*	IndoMalay	0	-0.0446	9.4598	3.56	0.7533	0.4844	8.24
Timaliidae	*Actinodura morrisoniana*	IndoMalay	0	-0.8744	9.4257	2.61	0.6478	0.3444	72.46
Timaliidae	*Actinodura nipalensis*	IndoMalay	0	0.3474	11.0298	3.95	0.7256	0.1386	11.09
Timaliidae	*Actinodura ramsayi*	IndoMalay	0	-0.5275	8.3725	3.16	0.7639	0.4513	23.44
Timaliidae	*Actinodura sodangorum*	IndoMalay	1	0.1034	8.1052	2.79	0.6370	0.5768	46.69
Timaliidae	*Actinodura souliei*	Palearctic	0	-0.1126	8.8892	3.33	0.7327	0.3333	85.00
Timaliidae	*Actinodura waldeni*	IndoMalay	0	-0.0739	8.7748	3.44	0.7599	0.7643	7.26
Timaliidae	*Alcippe brunnea*	Australasia	1	-0.3717	1.1451	2.50	0.6690	0.2790	23.71
Timaliidae	*Alcippe brunneicauda*	Palearctic	0	-0.4231	-	3.32	0.7720	0.5317	13.43
Timaliidae	*Alcippe castaneceps*	IndoMalay	0	0.0519	2.3001	2.85	0.7666	0.3619	1.27
Timaliidae	*Alcippe chrysotis*	IndoMalay	0	-0.0204	13.7593	3.24	0.7920	0.5076	51.80
Timaliidae	*Alcippe cinerea*	Palearctic	0	-0.4533	9.9407	3.46	0.7925	0.4711	7.67
Timaliidae	*Alcippe cinereiceps*	IndoMalay	0	-0.0040	10.0395	3.50	0.7515	0.5140	23.09
Timaliidae	*Alcippe danisi*	Palearctic	0	-0.3903	9.9689	3.48	0.7855	0.5602	33.13
Timaliidae	*Alcippe dubia*	IndoMalay	0	-0.0275	3.6671	2.95	0.6876	0.1785	48.05
Timaliidae	*Alcippe formosana*	IndoMalay	0	-0.1398	-	3.36	0.7969	0.6193	3.85
Timaliidae	*Alcippe grotei*	IndoMalay	0	-0.8796	7.7712	2.61	0.6478	0.3444	72.46
Timaliidae	*Alcippe klossi*	IndoMalay	0	-0.2117	3.4585	2.91	0.7444	0.5471	68.91
Timaliidae	*Alcippe ludlowi*	IndoMalay	0	0.4213	9.3202	2.88	0.6428	0.6755	18.16
Timaliidae	*Alcippe manipurensis*	Palearctic	0	0.3825	8.7662	4.09	0.7069	0.3944	13.46
Timaliidae	*Alcippe morrisonia*	IndoMalay	0	-0.0899	7.7650	3.31	0.7291	0.3008	24.87
Timaliidae	*Alcippe nipalensis*	IndoMalay	0	-0.4822	-	3.26	0.8147	0.5936	24.43
Timaliidae	*Alcippe peracensis*	IndoMalay	0	0.0137	-	3.54	0.7611	0.3619	11.21
Timaliidae	*Alcippe poioicephala*	IndoMalay	0	-0.0836	8.0287	2.83	0.7300	0.2162	62.78
Timaliidae	*Alcippe pyrrhoptera*	IndoMalay	0	-0.1919	-	3.16	0.8045	0.4148	7.76
Timaliidae	*Alcippe ruficapilla*	IndoMalay	0	-0.3786	-	2.76	0.6562	0.3688	20.08
Timaliidae	*Alcippe rufogularis*	Palearctic	0	-0.8178	8.3443	3.46	0.7676	0.4695	51.48
Timaliidae	*Alcippe striaticollis*	IndoMalay	0	-0.0687	3.9848	3.12	0.7693	0.2778	43.06
Timaliidae	*Alcippe variegaticeps*	Palearctic	0	7.0187	11.9526	4.02	0.7611	0.5178	24.37
Timaliidae	*Alcippe vinipectus*	Palearctic	1	-0.1103	6.6226	3.35	0.7382	0.3084	22.11
Timaliidae	*Babax koslowi*	Afrotropics	1	-0.2539	4.2275	3.06	0.6370	0.1526	88.79
Timaliidae	*Babax lanceolatus*	Palearctic	0	-0.6120	-	5.40	0.8415	0.0354	0.84
Timaliidae	*Babax waddelli*	Australasia	0	-0.4774	3.4886	2.33	0.7331	0.2569	44.63
Timaliidae	*Chamaea fasciata*	Neotropics	0	-0.0088	-	2.99	0.6428	0.2290	28.42
Timaliidae	*Chrysomma poecilotis*	Afrotropics	0	-0.0582	-	3.42	0.8500	0.5602	29.09
Timaliidae	*Chrysomma sinense*	Afrotropics	0	-0.0442	-	3.26	0.7938	0.2306	11.76
Timaliidae	*Conostoma oemodium*	Neotropics	0	-0.1550	-	3.79	0.8399	0.7196	24.39
Timaliidae	*Crocias albonotatus*	Australasia	0	-0.0911	13.1805	2.82	0.7416	0.1493	24.21
Timaliidae	*Crocias langbianis*	Neotropics	1	-0.2826	2.8800	3.36	0.7128	0.4290	80.59
Timaliidae	*Cutia legalleni*	IndoMalay	0	-0.4813	-	3.37	0.8465	0.6205	40.47
Timaliidae	*Cutia nipalensis*	Palearctic	0	-0.7520	12.5373	4.93	0.8539	0.2099	0.17

Family	Species	Realm	Threat	Latitude	Elevation	Anomaly	Size	Shape	Orientation
Timaliidae	*Dasycrotapha speciosa*	Nearctic	0	-0.4046	11.0941	3.98	0.7813	0.3254	51.76
Timaliidae	*Dumetia hyperythra*	Australasia	0	-0.0732	8.6276	2.71	0.7158	0.5198	57.16
Timaliidae	*Erpornis zantholeuca*	Palearctic	0	-1.0338	18.9230	4.29	0.8263	0.2028	13.31
Timaliidae	*Gampsorhynchus rufulus*	Palearctic	0	-0.2678	5.7314	5.30	0.8416	0.2585	0.61
Timaliidae	*Gampsorhynchus torquatus*	Palearctic	0	-0.3793	-	4.75	0.8179	0.2989	5.24
Timaliidae	*Garrulax affinis*	Palearctic	1	-10.4283	11.4761	4.20	0.7310	0.1365	12.09
Timaliidae	*Garrulax albogularis*	Afrotropics	0	-0.1079	5.3645	3.41	0.7660	0.2064	73.13
Timaliidae	*Garrulax annamensis*	Neotropics	0	0.1160	11.3317	3.30	0.7488	0.2374	60.37
Timaliidae	*Garrulax austeni*	Neotropics	0	-0.3161	11.2732	3.60	0.8571	0.6777	82.49
Timaliidae	*Garrulax berthemyi*	Palearctic	0	-1.0860	17.8948	4.40	0.8232	0.2137	15.81
Timaliidae	*Garrulax bicolor*	Palearctic	0	-1.0445	-	5.26	0.8441	0.2514	2.29
Timaliidae	*Garrulax bieti*	Neotropics	0	-0.4918	-	1.90	0.7507	0.4343	79.32
Timaliidae	*Garrulax caerulatus*	Neotropics	0	0.0647	-	3.70	0.8106	0.2827	62.26
Timaliidae	*Garrulax calvus*	Afrotropics	0	-0.0748	8.2391	3.55	0.8516	0.6521	43.28
Timaliidae	*Garrulax canorus*	Palearctic	0	-0.3939	10.1257	3.81	0.8887	0.3762	7.02
Timaliidae	*Garrulax castanotis*	Neotropics	0	-0.3073	-	3.01	0.8108	0.4484	46.32
Timaliidae	*Garrulax chinensis*	Australasia	0	-0.2668	-	2.21	0.6740	0.5984	20.11
Timaliidae	*Garrulax chrysopterus*	Australasia	1	2.0943	2.3344	2.46	0.5428	-	-
Timaliidae	*Garrulax cineraceus*	Australasia	0	-0.3591	-	3.13	0.8213	0.6341	60.78
Timaliidae	*Garrulax cinereifrons*	Australasia	0	-0.6499	0.0000	3.37	0.8270	0.4545	10.33
Timaliidae	*Garrulax courtoisi*	Australasia	1	-0.5016	5.6520	2.27	0.6784	0.1982	40.03
Timaliidae	*Garrulax davidi*	Australasia	0	-0.2723	4.1885	2.49	0.6662	0.3153	20.62
Timaliidae	*Garrulax delesserti*	Australasia	1	-0.2015	3.6321	2.58	0.6221	0.1501	33.56
Timaliidae	*Garrulax elliotii*	Australasia	0	-0.2448	8.2746	2.81	0.7996	0.5572	42.40
Timaliidae	*Garrulax erythrocephalus*	IndoMalay	0	-0.2497	-	3.06	0.8277	0.9183	40.00
Timaliidae	*Garrulax ferrarius*	IndoMalay	0	-0.1102	2.7778	2.66	0.7519	0.3766	89.96
Timaliidae	*Garrulax formosus*	IndoMalay	0	0.1892	3.0362	2.77	0.6690	0.4914	79.63
Timaliidae	*Garrulax galbanus*	IndoMalay	0	-0.0828	-	3.34	0.7923	0.7097	66.21
Timaliidae	*Garrulax gularis*	IndoMalay	0	-0.0726	-	3.36	0.7875	0.7003	60.45
Timaliidae	*Garrulax gularis*	IndoMalay	0	-0.0726	-	3.36	0.7875	-	-
Timaliidae	*Garrulax henrici*	IndoMalay	0	-0.2085	4.8828	3.10	0.8282	0.4056	39.61
Timaliidae	*Garrulax imbricatus*	IndoMalay	0	0.1529	3.4350	2.77	0.6784	0.5917	79.40
Timaliidae	*Garrulax konkakinhensis*	IndoMalay	0	-0.0448	6.3927	3.38	0.7825	0.5037	80.54
Timaliidae	*Garrulax leucolophus*	IndoMalay	0	-0.4370	5.0764	2.69	0.7211	0.0968	10.17
Timaliidae	*Garrulax lineatus*	Neotropics	0	-0.1599	-	3.87	0.8472	0.6429	40.39
Timaliidae	*Garrulax lugubris*	IndoMalay	0	-0.0985	4.1845	3.32	0.7643	0.8921	49.28
Timaliidae	*Garrulax lunulatus*	IndoMalay	0	-0.0566	6.6905	3.04	0.7683	0.6099	85.16
Timaliidae	*Garrulax maesi*	Neotropics	0	0.2725	-	1.51	0.6221	0.0002	1.05
Timaliidae	*Garrulax maximus*	Palearctic	0	-0.5471	12.3025	3.63	0.7818	0.4637	1.11
Timaliidae	*Garrulax melanostigma*	Palearctic	0	-0.5224	12.8891	3.74	0.7875	0.2609	0.62
Timaliidae	*Garrulax merulinus*	IndoMalay	0	0.5491	3.5012	2.88	0.6428	0.7899	47.45
Timaliidae	*Garrulax milleti*	IndoMalay	0	-0.3778	6.9646	3.18	0.7030	0.6094	72.72
Timaliidae	*Garrulax milnei*	Palearctic	0	-0.0643	5.8030	3.38	0.7744	0.4045	5.16
Timaliidae	*Garrulax mitratus*	IndoMalay	1	0.0545	6.8691	2.77	0.7251	0.0985	53.61
Timaliidae	*Garrulax monileger*	Palearctic	1	-37.6812	10.0299	3.56	0.6690	0.7142	43.19
Timaliidae	*Garrulax morrisonianus*	IndoMalay	0	0.2448	-	3.69	0.7474	0.2812	0.70
Timaliidae	*Garrulax ngoclinhensis*	IndoMalay	0	0.2638	-	2.94	0.6906	0.4596	68.41
Timaliidae	*Garrulax nuchalis*	Palearctic	0	-0.5325	5.4033	3.28	0.8022	0.6734	21.09
Timaliidae	*Garrulax ocellatus*	IndoMalay	0	-0.4386	6.4417	2.89	0.7211	0.6250	19.29
Timaliidae	*Garrulax palliatus*	IndoMalay	0	-0.1880	-	3.12	0.7947	0.8758	16.35
Timaliidae	*Garrulax pectoralis*	IndoMalay	0	0.1848	9.0323	3.34	0.7356	0.4286	56.57
Timaliidae	*Garrulax peninsulae*	Palearctic	0	-0.6928	-	3.35	0.7895	0.5311	11.48
Timaliidae	*Garrulax perspicillatus*	IndoMalay	1	0.3187	4.3966	2.76	0.6370	0.9166	89.25
Timaliidae	*Garrulax ruficeps*	Palearctic	0	-1.3323	12.2504	3.91	0.7878	0.3024	30.21
Timaliidae	*Garrulax ruficollis*	IndoMalay	0	0.0009	5.5206	3.13	0.7251	0.1698	66.76
Timaliidae	*Garrulax rufifrons*	Palearctic	0	-0.8806	14.6660	3.78	0.7825	0.4817	44.56
Timaliidae	*Garrulax rufogularis*	IndoMalay	0	-0.1653	12.2075	3.97	0.7609	0.1568	9.98
Timaliidae	*Garrulax sannio*	IndoMalay	0	-0.1151	2.6767	3.04	0.6221	0.3895	5.29
Timaliidae	*Garrulax squamatus*	Palearctic	0	-0.3514	11.0665	3.39	0.7305	0.6106	63.62
Timaliidae	*Garrulax strepitans*	IndoMalay	0	-0.4015	3.7749	3.17	0.7069	0.3584	60.57
Timaliidae	*Garrulax striatus*	IndoMalay	0	0.0228	-	3.31	0.7580	0.4361	30.96
Timaliidae	*Garrulax subunicolor*	Palearctic	0	-10.6768	14.4122	4.47	0.7314	0.4412	8.21
Timaliidae	*Garrulax sukatschewi*	IndoMalay	0	0.8952	5.5855	3.85	0.6859	0.3611	0.00
Timaliidae	*Garrulax variegatus*	IndoMalay	0	-0.0336	6.3923	3.23	0.8028	0.3560	34.47
Timaliidae	*Garrulax vassali*	Palearctic	0	-0.5068	13.3113	4.31	0.7661	0.3799	24.47
Timaliidae	*Garrulax virgatus*	IndoMalay	0	0.0124	-	2.80	0.6997	0.4996	63.49
Timaliidae	*Garrulax yersini*	Palearctic	0	6.2281	9.5802	3.48	0.7441	0.4510	35.34
Timaliidae	*Gypsophila crispifrons*	Neotropics	0	-0.3915	-	3.70	0.8042	0.6408	6.20
Timaliidae	*Heterophasia annectens*	Australasia	1	-0.3903	4.3281	2.49	0.6662	0.3153	20.62
Timaliidae	*Heterophasia auricularis*	Australasia	0	-0.0967	6.1447	2.78	0.7716	0.3088	23.58
Timaliidae	*Heterophasia capistrata*	Australasia	0	-0.0954	2.5447	2.78	0.7716	0.3088	23.58
Timaliidae	*Heterophasia desgodinsi*	Australasia	1	-0.2829	2.4989	2.49	0.6662	0.3153	20.62
Timaliidae	*Heterophasia gracilis*	Neotropics	0	-0.0325	15.4383	3.59	0.7898	0.3753	47.16
Timaliidae	*Heterophasia melanoleuca*	Neotropics	0	-5.2994	6.3056	3.46	0.6221	0.5728	71.79

Family	Species	Realm	Threat	Latitude	Elevation	Anomaly	Size	Shape	Orientation
Timaliidae	*Heterophasia picaoides*	Neotropics	0	0.0043	-	3.71	0.8118	0.4539	21.62
Timaliidae	*Heterophasia pulchella*	Neotropics	1	0.0000	2.4328	3.02	0.5788	0.0000	45.00
Timaliidae	*Illadopsis albipectus*	Nearctic	0	-0.7485	-	4.16	0.8358	0.4878	7.55
Timaliidae	*Illadopsis cleaveri*	Nearctic	0	0.0763	7.3153	3.94	0.7493	0.6895	43.89
Timaliidae	*Illadopsis cleaveri*	Nearctic	0	0.0763	7.3153	3.94	0.7493	-	-
Timaliidae	*Illadopsis fulvescens*	Neotropics	0	-0.2513	-	2.87	0.7112	0.6348	42.32
Timaliidae	*Illadopsis puveli*	Neotropics	0	-0.1470	-	3.71	0.7626	0.4671	7.11
Timaliidae	*Illadopsis pyrrhoptera*	Nearctic	0	-0.6682	8.6909	4.13	0.8196	0.5206	41.85
Timaliidae	*Illadopsis rufescens*	Neotropics	0	0.0450	2.9826	4.03	0.8342	0.7665	2.45
Timaliidae	*Illadopsis rufipennis*	Neotropics	0	-0.1052	-	3.39	0.7797	0.3089	33.53
Timaliidae	*Jabouilleia danjoui*	Neotropics	0	0.4311	1.8481	3.24	0.6906	0.2869	1.94
Timaliidae	*Kakamega poliothorax*	IndoMalay	0	-0.0882	-	3.00	0.8192	0.5246	47.30
Timaliidae	*Kenopia striata*	Neotropics	0	-4.7656	8.1150	3.72	0.7340	0.3022	64.09
Timaliidae	*Kupeornis chapini*	IndoMalay	0	-0.0515	2.8157	2.62	0.7008	0.6308	85.20
Timaliidae	*Kupeornis gilberti*	IndoMalay	0	0.0148	-	2.84	0.7880	0.5118	10.58
Timaliidae	*Kupeornis rufocinctus*	IndoMalay	0	0.2432	-	2.52	0.5985	0.0024	45.41
Timaliidae	*Leiothrix argentauris*	Afrotropics	0	0.0245	5.6298	3.26	0.7911	0.6601	48.58
Timaliidae	*Leiothrix lutea*	Palearctic	0	-0.6880	8.0376	4.45	0.8808	0.2526	0.55
Timaliidae	*Leonardina woodi*	Afrotropics	0	-0.0836	-	3.39	0.7647	0.1321	10.96
Timaliidae	*Liocichla bugunorum*	Neotropics	0	-0.0154	-	3.97	0.8425	0.5846	35.31
Timaliidae	*Liocichla omeiensis*	Neotropics	0	-0.1150	-	4.15	0.8079	0.4188	10.49
Timaliidae	*Liocichla phoenicea*	Neotropics	1	-0.2121	9.8054	2.94	0.7323	0.2391	52.25
Timaliidae	*Liocichla steerii*	Neotropics	0	-0.1017	-	3.98	0.8091	0.4236	9.65
Timaliidae	*Lioptilus nigricapillus*	Neotropics	1	0.6133	7.8480	3.13	0.6986	0.4004	88.30
Timaliidae	*Macronous bornensis*	Australasia	0	-0.0838	3.2425	2.71	0.7185	0.5623	60.71
Timaliidae	*Macronous flavicollis*	Australasia	0	-0.3219	1.8378	2.50	0.6716	0.2680	24.42
Timaliidae	*Macronous gularis*	IndoMalay	0	-0.1147	-	3.14	0.8067	0.5237	11.24
Timaliidae	*Macronous kelleyi*	Australasia	0	0.3821	8.1346	2.52	0.5788	0.0000	90.00
Timaliidae	*Macronous ptilosus*	Australasia	0	-0.7496	-	2.46	0.5985	0.1035	28.88
Timaliidae	*Macronous striaticeps*	Australasia	1	-0.0064	3.9569	2.48	0.6370	0.1926	10.82
Timaliidae	*Malacocincla abbotti*	Palearctic	0	1.4312	5.3062	4.15	0.7855	0.3715	15.04
Timaliidae	*Malacocincla cinereiceps*	Palearctic	0	1.5657	-	4.34	0.8173	0.4353	12.03
Timaliidae	*Malacocincla malaccensis*	Palearctic	1	0.6454	2.3520	3.65	0.7040	0.3352	2.57
Timaliidae	*Malacocincla perspicillata*	Palearctic	0	-0.5982	-	4.90	0.8308	0.2674	6.25
Timaliidae	*Malacocincla sepiaria*	Palearctic	0	-0.8407	4.7810	5.00	0.8659	0.2082	2.73
Timaliidae	*Malacopteron affine*	Afrotropics	0	-0.0655	-	3.58	0.8074	0.5147	15.20
Timaliidae	*Malacopteron albogulare*	Afrotropics	0	1.3103	10.1347	3.49	0.7864	0.3868	21.15
Timaliidae	*Malacopteron cinereum*	Afrotropics	0	-0.9709	1.8648	3.45	0.7040	0.5939	82.82
Timaliidae	*Malacopteron magnirostre*	Afrotropics	0	-0.2328	8.6375	3.66	0.8262	0.9316	25.05
Timaliidae	*Malacopteron magnum*	Afrotropics	0	0.0749	13.8656	3.55	0.7687	0.6165	84.81
Timaliidae	*Malacopteron palawanense*	Afrotropics	0	0.2024	6.6700	3.54	0.8357	0.2511	0.81
Timaliidae	*Malia grata*	Neotropics	0	-0.2645	0.9550	3.45	0.8359	0.7554	67.52
Timaliidae	*Micromacronus leytensis*	Afrotropics	0	-0.0395	-	3.66	0.8305	0.8688	45.64
Timaliidae	*Micromacronus sordidus*	Afrotropics	0	0.0197	-	3.50	0.8155	0.1487	6.17
Timaliidae	*Minla cyanouroptera*	Afrotropics	0	0.1061	12.8868	3.51	0.8420	0.3179	6.86
Timaliidae	*Minla ignotincta*	Afrotropics	0	-0.1977	18.0494	3.65	0.8082	0.8069	72.00
Timaliidae	*Minla strigula*	Neotropics	0	-0.1918	6.7825	4.07	0.7128	0.1351	33.31
Timaliidae	*Myzornis pyrrhoura*	Afrotropics	0	-0.0012	-	3.44	0.8180	0.1628	7.71
Timaliidae	*Napothera brevicaudata*	Afrotropics	0	-0.0191	-	3.51	0.8216	0.7556	27.97
Timaliidae	*Napothera crassa*	Afrotropics	0	-3.4727	5.9974	3.55	0.7565	0.5927	69.27
Timaliidae	*Napothera epilepidota*	Afrotropics	0	0.1238	-	3.55	0.7512	0.4618	82.90
Timaliidae	*Neomixis flavoviridis*	Afrotropics	0	-0.1551	-	3.29	0.8241	0.3697	14.47
Timaliidae	*Neomixis striatigula*	Afrotropics	0	-0.1587	5.8600	3.31	0.8030	0.1403	7.38
Timaliidae	*Neomixis tenella*	Afrotropics	0	-11.7231	-	3.28	0.7323	0.3497	19.47
Timaliidae	*Neomixis viridis*	Afrotropics	0	-0.0543	-	3.46	0.8415	0.4826	26.94
Timaliidae	*Panurus biarmicus*	Afrotropics	0	-0.0615	-	3.61	0.8595	0.7539	46.01
Timaliidae	*Paradoxornis alphonsianus*	Australasia	0	0.9634	6.4461	2.63	0.6478	0.0000	0.00
Timaliidae	*Paradoxornis atrosuperciliaris*	IndoMalay	0	-0.1187	7.6060	3.24	0.8194	0.7225	37.87
Timaliidae	*Paradoxornis brunneus*	IndoMalay	0	-0.6559	4.0672	3.51	0.8394	0.3372	28.12
Timaliidae	*Paradoxornis conspicillatus*	IndoMalay	1	0.2947	2.1405	2.76	0.6120	0.9804	90.00
Timaliidae	*Paradoxornis davidianus*	Afrotropics	0	-0.1522	-	3.04	0.7291	0.1519	76.24
Timaliidae	*Paradoxornis flavirostris*	Neotropics	0	-0.2237	13.2774	3.70	0.6974	0.4772	58.92
Timaliidae	*Paradoxornis fulvifrons*	Neotropics	0	-0.1664	-	3.65	0.8054	0.5325	56.66
Timaliidae	*Paradoxornis gularis*	Australasia	0	-0.5793	0.0000	2.87	0.7659	0.3400	2.56
Timaliidae	*Paradoxornis guttaticollis*	Nearctic	0	-0.4774	8.0388	3.91	0.8468	0.4604	59.68
Timaliidae	*Paradoxornis heudei*	Palearctic	1	-0.9498	-	4.76	0.8238	0.2780	11.16
Timaliidae	*Paradoxornis margaritae*	Afrotropics	0	-0.1884	15.0676	3.77	0.8065	0.4275	64.05
Timaliidae	*Paradoxornis nipalensis*	Neotropics	0	-0.5831	-	2.51	0.8039	0.5022	57.88
Timaliidae	*Paradoxornis paradoxus*	Australasia	0	-0.0793	6.7899	2.81	0.7498	0.1725	24.64
Timaliidae	*Paradoxornis przewalskii*	IndoMalay	0	-0.0845	-	2.66	0.7030	0.4461	81.12
Timaliidae	*Paradoxornis unicolor*	Australasia	0	-0.2624	-	2.88	0.7172	0.2151	19.32
Timaliidae	*Paradoxornis verreauxi*	Australasia	0	-0.2451	-	2.58	0.6302	0.1134	34.50
Timaliidae	*Paradoxornis webbianus*	Australasia	0	-0.0362	-	2.48	0.6690	0.5741	71.12
Timaliidae	*Paradoxornis zappeyi*	IndoMalay	0	-0.0386	-	2.83	0.7901	0.4692	52.46

Family	Species	Realm	Threat	Latitude	Elevation	Anomaly	Size	Shape	Orientation
Timaliidae	*Parophasma galinieri*	Australasia	0	-0.0759	12.7494	2.82	0.7406	0.1803	23.94
Timaliidae	*Pellorneum albiventre*	Afrotropics	0	0.0916	10.1732	3.57	0.7574	0.6585	86.51
Timaliidae	*Pellorneum capistratum*	Palearctic	0	-1.0161	8.8112	4.01	0.7973	0.2123	16.10
Timaliidae	*Pellorneum fuscocapillus*	Palearctic	0	-0.5162	10.3126	4.17	0.8770	0.3668	7.18
Timaliidae	*Pellorneum palustre*	Palearctic	0	-0.5309	9.8570	4.73	0.8669	0.1951	0.30
Timaliidae	*Pellorneum ruficeps*	Palearctic	0	-0.3633	14.8722	3.54	0.8028	0.4389	1.37
Timaliidae	*Phyllanthus atripennis*	Afrotropics	0	-21.8700	-	3.49	0.7560	0.3956	24.12
Timaliidae	*Pnoepyga albiventer*	Neotropics	0	0.0970	8.0284	3.69	0.7172	0.2904	89.54
Timaliidae	*Pnoepyga formosana*	Neotropics	1	-0.4729	3.6147	2.97	0.7631	0.3486	19.68
Timaliidae	*Pnoepyga immaculata*	Neotropics	0	-0.1976	7.7655	3.68	0.8340	0.5578	38.58
Timaliidae	*Pnoepyga pusilla*	Neotropics	0	0.1359	-	4.05	0.8304	0.6731	14.30
Timaliidae	*Pomatorhinus erythrocnemis*	Australasia	0	-0.2801	3.2168	2.81	0.7751	0.3624	52.69
Timaliidae	*Pomatorhinus erythrogenys*	Australasia	0	-0.1539	2.1849	2.83	0.7790	0.5037	51.90
Timaliidae	*Pomatorhinus ferruginosus*	Australasia	0	-0.5522	-	3.34	0.8379	0.5824	7.72
Timaliidae	*Pomatorhinus gravivox*	Afrotropics	0	-0.0670	7.2630	3.46	0.8516	0.5446	32.54
Timaliidae	*Pomatorhinus hypoleucos*	Palearctic	0	-0.8366	3.0430	4.69	0.8611	0.0519	0.37
Timaliidae	*Pomatorhinus mcclellandi*	Palearctic	0	-0.5182	7.5892	4.27	0.8660	0.5301	5.45
Timaliidae	*Pomatorhinus melanurus*	Neotropics	1	-0.8018	3.6945	1.75	0.6842	0.5717	76.64
Timaliidae	*Pomatorhinus montanus*	Palearctic	0	-0.8200	3.0188	4.61	0.8638	0.0680	0.24
Timaliidae	*Pomatorhinus musicus*	Neotropics	0	-0.3852	2.8288	2.57	0.8131	0.6312	78.60
Timaliidae	*Pomatorhinus ochraceiceps*	Palearctic	0	-0.5738	6.7145	4.39	0.8623	0.3352	0.54
Timaliidae	*Pomatorhinus ochraceiceps*	Palearctic	0	-0.5738	6.7145	4.39	0.8623	-	-
Timaliidae	*Pomatorhinus ruficollis*	Neotropics	0	-0.3593	11.6715	2.90	0.8117	0.3466	83.97
Timaliidae	*Pseudoalcippe abyssinica*	Afrotropics	0	-0.4827	-	3.37	0.7356	0.3378	82.98
Timaliidae	*Pteruthius aenobarbus*	Neotropics	0	-0.0777	10.7153	3.06	0.6631	0.3326	30.61
Timaliidae	*Pteruthius flaviscapis*	Australasia	1	0.1507	-	3.16	0.6428	0.4853	84.41
Timaliidae	*Pteruthius melanotis*	Australasia	0	-0.0820	-	3.32	0.7291	0.9682	55.60
Timaliidae	*Pteruthius rufiventer*	Australasia	0	-0.6815	-	3.18	0.7929	0.8068	30.87
Timaliidae	*Ptilocichla falcata*	Neotropics	0	0.0960	8.6916	3.65	0.7552	0.3680	69.18
Timaliidae	*Ptilocichla leucogrammica*	Neotropics	0	-5.2010	5.9139	4.05	0.7144	0.1660	37.39
Timaliidae	*Ptilocichla mindanensis*	IndoMalay	0	0.0150	7.9081	2.84	0.7584	0.4258	60.43
Timaliidae	*Ptyrticus turdinus*	IndoMalay	0	-0.1367	-	3.12	0.7995	0.4484	39.28
Timaliidae	*Rhopocichla atriceps*	Afrotropics	0	-0.1307	-	3.67	0.8493	0.7838	61.34
Timaliidae	*Rimator albostriatus*	Neotropics	0	0.0055	9.9563	3.78	0.7469	0.2636	71.93
Timaliidae	*Rimator malacoptilus*	Neotropics	0	0.3217	3.5815	3.00	0.7192	0.1641	71.08
Timaliidae	*Rimator pasquieri*	Neotropics	0	-0.5881	6.1590	3.25	0.7916	0.5238	32.75
Timaliidae	*Robsonius rabori*	Neotropics	0	-0.1963	4.6803	3.98	0.8407	0.6140	26.87
Timaliidae	*Robsonius sorsogonensis*	Neotropics	0	-0.1647	-	3.90	0.8447	0.4789	33.00
Timaliidae	*Spelaeornis badeigularis*	Neotropics	1	0.0185	6.1025	2.88	0.6120	0.5587	47.92
Timaliidae	*Spelaeornis chocolatinus*	Neotropics	0	0.2833	9.8731	3.03	0.6478	0.3341	24.93
Timaliidae	*Spelaeornis formosus*	Nearctic	0	-0.3837	6.0781	4.19	0.7944	0.2903	57.14
Timaliidae	*Spelaeornis kinneari*	Nearctic	0	-0.6549	-	3.47	0.7957	0.2920	33.08
Timaliidae	*Spelaeornis longicaudatus*	Nearctic	0	-0.6356	-	2.96	0.6859	0.1454	57.74
Timaliidae	*Spelaeornis oatesi*	Neotropics	0	-0.0737	6.5444	3.04	0.6662	0.2278	35.23
Timaliidae	*Spelaeornis reptatus*	Neotropics	0	-0.0480	-	4.08	0.8012	0.4864	35.05
Timaliidae	*Spelaeornis troglodytoides*	Neotropics	0	-0.3060	-	3.10	0.7797	0.3089	33.59
Timaliidae	*Sphenocichla humei*	Neotropics	0	-0.1950	7.0326	3.02	0.6740	0.2182	28.75
Timaliidae	*Sphenocichla roberti*	Neotropics	0	-0.3678	7.6389	3.02	0.7008	0.1991	62.83
Timaliidae	*Stachyris ambigua*	Afrotropics	1	-0.2919	2.8387	3.36	0.6784	0.1938	80.97
Timaliidae	*Stachyris capitalis*	Afrotropics	0	-0.0957	4.1003	3.18	0.7078	0.1528	73.67
Timaliidae	*Stachyris chrysaea*	Afrotropics	1	-2.6890	6.6789	3.32	0.6562	0.2333	45.71
Timaliidae	*Stachyris dennistouni*	Afrotropics	1	0.0000	3.6828	3.05	0.5788	0.0000	90.00
Timaliidae	*Stachyris erythroptera*	Afrotropics	0	0.0557	-	3.31	0.7223	0.2531	49.99
Timaliidae	*Stachyris grammiceps*	Nearctic	0	-0.9237	14.7755	4.05	0.8259	0.4031	30.81
Timaliidae	*Stachyris herberti*	Nearctic	0	-0.6184	15.7660	3.97	0.8045	0.3262	50.48
Timaliidae	*Stachyris hypogrammica*	Nearctic	0	-0.6641	9.4541	4.21	0.8334	0.7136	37.06
Timaliidae	*Stachyris latistriata*	Neotropics	0	2.8172	4.1626	3.52	0.7256	0.1372	81.17
Timaliidae	*Stachyris leucotis*	Neotropics	0	-0.1060	10.7267	3.91	0.7958	0.5147	47.23
Timaliidae	*Stachyris maculata*	Neotropics	0	0.0276	-	4.02	0.7969	0.2692	35.85
Timaliidae	*Stachyris melanothorax*	Neotropics	0	-0.2664	-	3.45	0.8360	0.7549	57.08
Timaliidae	*Stachyris nigriceps*	Neotropics	0	-0.7134	-	2.26	0.7546	0.3763	74.73
Timaliidae	*Stachyris nigricollis*	Neotropics	0	4.1080	5.0622	4.14	0.7524	0.2537	62.33
Timaliidae	*Stachyris nigrocapitata*	Neotropics	0	-13.6255	4.4469	3.99	0.7087	0.3912	87.77
Timaliidae	*Stachyris nigrorum*	Neotropics	0	-0.2249	6.1921	3.01	0.8142	0.4247	81.46
Timaliidae	*Stachyris nonggangensis*	Neotropics	0	-0.2808	7.7890	3.87	0.7869	0.3338	70.93
Timaliidae	*Stachyris oglei*	Neotropics	0	-0.0061	10.2598	3.71	0.7060	0.1346	53.41
Timaliidae	*Stachyris plateni*	Neotropics	0	-0.2447	-	3.17	0.6804	0.2826	73.06
Timaliidae	*Stachyris poliocephala*	Neotropics	0	4.1264	13.7259	4.06	0.7751	0.2800	50.34
Timaliidae	*Stachyris pygmaea*	Afrotropics	0	-0.5353	-	3.69	0.7836	0.5946	43.41
Timaliidae	*Stachyris pyrrhops*	Neotropics	0	-4.4270	3.9341	4.03	0.7112	0.1894	38.59
Timaliidae	*Stachyris ruficeps*	Neotropics	0	-10.7769	5.7704	4.21	0.7425	0.6657	36.27
Timaliidae	*Stachyris rufifrons*	Neotropics	1	0.7189	-	2.47	0.5788	0.0000	0.00
Timaliidae	*Stachyris striata*	Neotropics	0	0.1955	3.1149	2.47	0.6598	0.6633	20.37
Timaliidae	*Stachyris striolata*	Australasia	0	-0.0876	-	2.78	0.7659	0.2594	23.90

Family	Species	Realm	Threat	Latitude	Elevation	Anomaly	Size	Shape	Orientation
Timaliidae	*Stachyris thoracica*	Neotropics	0	-29.3942	6.2557	3.31	0.6876	0.1422	68.48
Timaliidae	*Stachyris whiteheadi*	Neotropics	1	-16.6625	7.8129	3.59	0.6478	0.3298	65.46
Timaliidae	*Strophocincla cachinnans*	Neotropics	0	-0.1484	-	3.86	0.8458	0.4426	45.01
Timaliidae	*Strophocincla fairbanki*	Neotropics	0	-0.1460	-	3.87	0.8424	0.4808	39.48
Timaliidae	*Timalia pileata*	Afrotropics	0	0.0439	8.8854	3.24	0.8010	0.2715	15.75
Timaliidae	*Trichastoma bicolor*	Neotropics	0	-0.0145	-	4.01	0.8291	0.7317	14.37
Timaliidae	*Trichastoma buettikoferi*	Neotropics	0	-0.0953	1.3221	3.50	0.7300	0.4838	82.83
Timaliidae	*Trichastoma celebense*	Neotropics	0	0.0359	2.8173	3.30	0.7677	0.6335	45.66
Timaliidae	*Trichastoma pyrrogenys*	Afrotropics	0	-0.2801	-	3.61	0.8614	0.7855	54.73
Timaliidae	*Trichastoma rostratum*	Afrotropics	1	-0.1572	0.4175	3.07	0.6974	0.2263	64.52
Timaliidae	*Trichastoma tickelli*	IndoMalay	0	-0.3844	-	3.44	0.7989	0.4240	25.17
Timaliidae	*Turdinus atrigularis*	Neotropics	0	-0.2537	4.9061	3.11	0.7820	0.3317	33.76
Timaliidae	*Turdinus macrodactylus*	Neotropics	0	-0.0169	6.0605	4.09	0.8214	0.6066	2.92
Timaliidae	*Turdinus marmorata*	Neotropics	0	-0.1839	-	3.88	0.8505	0.6340	36.44
Timaliidae	*Turdinus rufipectus*	Neotropics	0	-0.1585	-	3.82	0.8497	0.4684	36.44
Timaliidae	*Turdoides affinis*	Neotropics	0	-0.0911	-	3.89	0.8351	0.3792	34.71
Timaliidae	*Turdoides altirostris*	Afrotropics	0	0.0206	-	3.27	0.7906	0.4662	55.47
Timaliidae	*Turdoides aylmeri*	Afrotropics	0	-0.1403	-	3.47	0.8185	0.8603	79.52
Timaliidae	*Turdoides bicolor*	Afrotropics	0	0.1882	-	4.27	0.7755	0.4214	3.49
Timaliidae	*Turdoides caudata*	Afrotropics	0	0.0443	-	3.22	0.8010	0.2363	13.41
Timaliidae	*Turdoides earlei*	Afrotropics	0	-0.0113	-	3.31	0.7975	0.3989	56.30
Timaliidae	*Turdoides fulva*	Afrotropics	0	-0.0943	-	3.66	0.8495	0.7240	31.77
Timaliidae	*Turdoides gularis*	Afrotropics	0	0.0084	-	3.34	0.8307	0.3451	17.74
Timaliidae	*Turdoides gymnogenys*	Afrotropics	0	0.0699	-	3.36	0.7950	0.5547	48.72
Timaliidae	*Turdoides hartlaubii*	Afrotropics	0	0.0865	-	3.28	0.8206	0.2931	12.19
Timaliidae	*Turdoides hindei*	Afrotropics	0	0.0329	-	3.50	0.7710	0.5308	60.79
Timaliidae	*Turdoides hypoleuca*	Afrotropics	0	0.8058	-	3.32	0.7165	0.7210	76.35
Timaliidae	*Turdoides jardineii*	Afrotropics	0	-0.1696	-	4.10	0.8021	0.5553	9.28
Timaliidae	*Turdoides leucocephala*	Afrotropics	0	-0.3499	-	3.91	0.7548	0.3914	64.41
Timaliidae	*Turdoides leucopygia*	Afrotropics	0	-0.0313	6.0790	3.60	0.8476	0.6510	29.74
Timaliidae	*Turdoides longirostris*	Afrotropics	0	-0.0368	-	3.62	0.8041	0.3688	3.42
Timaliidae	*Turdoides malcolmi*	Australasia	0	-0.1959	3.3972	2.49	0.6662	0.3153	20.62
Timaliidae	*Turdoides melanops*	Australasia	0	-0.1267	2.5158	2.58	0.6740	0.1664	3.70
Timaliidae	*Turdoides nipalensis*	IndoMalay	0	-0.1511	3.0873	2.91	0.8212	0.3423	25.78
Timaliidae	*Turdoides plebejus*	Australasia	0	-0.1511	2.2427	2.48	0.6302	0.2423	87.23
Timaliidae	*Turdoides rubiginosa*	Australasia	1	0.1031	1.9827	2.48	0.6221	0.5774	71.57
Timaliidae	*Turdoides rufescens*	Australasia	0	0.1503	1.9518	2.48	0.6370	0.1926	10.82
Timaliidae	*Turdoides sharpei*	Australasia	0	-0.1436	6.1685	2.48	0.6120	0.0400	36.10
Timaliidae	*Turdoides squamiceps*	Australasia	0	-0.3388	-	3.15	0.7918	0.5846	53.46
Timaliidae	*Turdoides squamulata*	Australasia	0	-0.0832	1.1715	2.76	0.7597	0.3753	25.96
Timaliidae	*Turdoides striata*	Australasia	0	-0.5110	-	3.36	0.8374	0.5678	6.24
Timaliidae	*Turdoides subrufa*	Australasia	0	-0.4200	-	3.28	0.8325	0.5132	13.44
Timaliidae	*Turdoides tenebrosa*	Australasia	0	-0.1069	-	2.58	0.7327	0.1403	14.58
Timaliidae	*Xiphirhynchus superciliaris*	Australasia	0	-0.4573	-	3.25	0.8280	0.6316	8.51
Timaliidae	*Yuhina bakeri*	Australasia	0	-0.1399	4.0285	2.61	0.7300	0.1200	25.61
Timaliidae	*Yuhina brunneiceps*	Afrotropics	0	0.7378	-	3.22	0.7008	0.4993	44.06
Timaliidae	*Yuhina castaniceps*	Afrotropics	0	-0.1701	9.7362	3.95	0.8046	0.6488	50.25
Timaliidae	*Yuhina diademata*	Neotropics	0	-0.0111	-	4.02	0.8001	0.6274	24.04
Timaliidae	*Yuhina everetti*	Neotropics	0	-0.0701	5.7199	3.66	0.7590	0.2687	69.04
Timaliidae	*Yuhina flavicollis*	Neotropics	0	1.8786	7.7347	3.68	0.7552	0.3460	68.59
Timaliidae	*Yuhina gularis*	Neotropics	0	-0.1291	-	3.39	0.7556	0.3788	6.39
Timaliidae	*Yuhina humilis*	Palearctic	0	-0.9476	-	3.84	0.7818	0.7056	35.23
Timaliidae	*Yuhina nigrimenta*	Palearctic	0	-0.8698	4.9364	3.70	0.7340	0.4601	53.43
Timaliidae	*Yuhina occipitalis*	IndoMalay	0	0.1553	-	2.90	0.7310	0.3479	58.56
Timaliidae	*Yuhina torqueola*	Neotropics	0	-0.0657	4.9036	2.98	0.6804	0.1468	63.57
Tinamidae	*Crypturellus atrocapillus*	Afrotropics	0	0.0768	-	3.25	0.7945	0.3868	5.00
Tinamidae	*Crypturellus bartletti*	Afrotropics	1	0.1904	2.7455	3.05	0.7356	0.2844	12.62
Tinamidae	*Crypturellus boucardi*	IndoMalay	1	0.4311	4.5370	2.90	0.5985	0.0000	90.00
Tinamidae	*Crypturellus brevirostris*	Afrotropics	0	-0.1277	7.7170	3.05	0.7069	0.0945	74.79
Tinamidae	*Crypturellus casiquiare*	Palearctic	0	9.1106	-	3.98	0.7634	0.8360	26.13
Tinamidae	*Crypturellus cinnamomeus*	Palearctic	0	-24.4768	-	4.70	0.7136	0.6676	20.99
Tinamidae	*Crypturellus duidae*	Palearctic	1	-0.7689	8.2478	3.82	0.7378	0.4263	34.55
Tinamidae	*Crypturellus erythropus*	Neotropics	0	-0.2013	6.3245	3.75	0.8549	0.6349	49.65
Tinamidae	*Crypturellus kerriae*	Neotropics	0	-0.1935	-	3.85	0.8513	0.6359	40.95
Tinamidae	*Crypturellus noctivagus*	Neotropics	0	-0.0966	5.8135	3.67	0.8130	0.3902	40.78
Tinamidae	*Crypturellus parvirostris*	IndoMalay	0	-0.0857	-	3.03	0.7863	0.7185	55.60
Tinamidae	*Crypturellus strigulosus*	Afrotropics	0	-0.1363	-	3.38	0.7700	0.4170	34.85
Tinamidae	*Crypturellus tataupa*	Afrotropics	0	3.7589	8.7528	3.36	0.7600	0.4996	69.35
Tinamidae	*Crypturellus transfasciatus*	Afrotropics	1	8.6808	4.9465	3.43	0.7096	0.3569	81.40
Tinamidae	*Crypturellus undulatus*	Afrotropics	0	-0.1180	8.8343	3.07	0.7281	0.1436	74.31
Tinamidae	*Crypturellus variegatus*	Neotropics	0	-4.7201	3.8429	4.07	0.7256	0.1779	36.38
Tinamidae	*Eudromia elegans*	Afrotropics	0	0.2803	8.9712	3.30	0.7816	0.3870	9.10
Tinamidae	*Eudromia formosa*	Afrotropics	0	0.1406	-	3.53	0.7989	0.1298	6.75
Tinamidae	*Nothoprocta cinerascens*	Neotropics	0	-0.0069	-	2.46	0.6876	0.3855	3.42

Family	Species	Realm	Threat	Latitude	Elevation	Anomaly	Size	Shape	Orientation
Tinamidae	*Nothoprocta curvirostris*	Afrotropics	0	-0.0734	0.7134	3.18	0.7614	0.3874	70.15
Tinamidae	*Nothoprocta ornata*	Neotropics	1	-0.8592	8.9070	2.47	0.5985	0.0000	0.00
Tinamidae	*Nothoprocta pentlandii*	Neotropics	0	3.0419	4.7808	2.41	0.5788	0.0000	0.00
Tinamidae	*Nothoprocta perdicaria*	Australasia	1	-0.4621	3.5749	2.40	0.7192	0.2733	41.29
Tinamidae	*Nothoprocta taczanowskii*	Australasia	1	-0.4756	9.6954	2.25	0.6997	0.1926	33.57
Tinamidae	*Nothura boraquira*	Afrotropics	0	-0.1041	15.9848	3.61	0.8491	0.3229	2.82
Tinamidae	*Nothura chacoensis*	Neotropics	0	-0.5773	2.7882	2.50	0.8114	0.4022	56.02
Tinamidae	*Nothura darwinii*	Palearctic	0	-1.0230	-	4.49	0.8363	0.2076	2.70
Tinamidae	*Nothura maculosa*	Afrotropics	0	-0.0624	-	3.51	0.8612	0.5952	28.30
Tinamidae	*Nothura minor*	IndoMalay	0	-0.2610	-	3.28	0.8446	0.4142	31.07
Tinamidae	*Rhynchotus maculicollis*	Afrotropics	0	-0.1178	14.1405	3.62	0.8325	0.6307	74.14
Tinamidae	*Rhynchotus rufescens*	Nearctic	0	-0.9266	6.4457	4.10	0.8178	0.7894	42.62
Tinamidae	*Taoniscus nanus*	Palearctic	0	-0.6158	8.7769	3.88	0.7539	0.1580	13.38
Tinamidae	*Tinamotis ingoufi*	Afrotropics	0	-0.1921	5.5691	3.32	0.7647	0.1600	70.30
Tinamidae	*Tinamotis pentlandii*	Afrotropics	0	-0.1403	12.0868	3.54	0.8452	0.5205	35.82
Todidae	*Todus angustirostris*	Australasia	0	-0.5595	-	2.60	0.7455	0.0957	0.92
Todidae	*Todus mexicanus*	Neotropics	1	0.2447	0.3041	1.51	0.6221	0.0002	1.05
Todidae	*Todus multicolor*	Afrotropics	0	-0.1290	-	3.59	0.8467	0.7724	73.78
Todidae	*Todus subulatus*	Neotropics	0	-0.1879	4.3854	3.45	0.7643	0.3417	25.72
Todidae	*Todus todus*	Neotropics	0	0.1911	4.1535	3.01	0.7310	0.1972	74.57
Trochilidae	*Abeillia abeillei*	Neotropics	0	-0.0464	6.6949	3.60	0.7340	0.2162	20.79
Trochilidae	*Adelomyia melanogenys*	Neotropics	0	-0.1439	8.1474	3.68	0.7794	0.4314	75.41
Trochilidae	*Aglaeactis aliciae*	Neotropics	0	0.5870	2.1287	2.41	0.5788	0.0000	0.00
Trochilidae	*Aglaeactis castelnaudii*	Neotropics	1	0.0000	3.6887	3.60	0.5788	0.0000	90.00
Trochilidae	*Aglaeactis cupripennis*	Neotropics	0	-15.0275	4.9847	4.12	0.6690	0.2324	46.99
Trochilidae	*Aglaeactis pamela*	Neotropics	0	0.0523	10.4750	3.63	0.7556	0.3548	88.21
Trochilidae	*Aglaiocercus berlepschi*	Neotropics	0	13.4051	3.2049	4.02	0.6690	0.2280	22.99
Trochilidae	*Aglaiocercus coelestis*	Neotropics	1	-0.0764	2.1667	3.68	0.6221	0.5865	19.14
Trochilidae	*Aglaiocercus kingi*	Neotropics	0	0.3760	6.4995	3.05	0.7019	0.0965	67.17
Trochilidae	*Amazilia amabilis*	Neotropics	0	-0.5818	6.9621	2.94	0.7672	0.3751	27.77
Trochilidae	*Amazilia amazilia*	Neotropics	0	-0.0133	2.2623	3.18	0.7519	0.6317	66.24
Trochilidae	*Amazilia beryllina*	Neotropics	0	-0.0976	3.8990	3.32	0.7305	0.1576	70.03
Trochilidae	*Amazilia boucardi*	Neotropics	0	-0.0889	8.3283	3.77	0.7718	0.2100	33.31
Trochilidae	*Amazilia brevirostris*	Neotropics	1	-0.1665	-	3.09	0.6221	0.1604	34.06
Trochilidae	*Amazilia candida*	Neotropics	0	-0.1511	1.1335	4.08	0.7663	0.4254	19.01
Trochilidae	*Amazilia castaneiventris*	Neotropics	0	0.0313	3.2972	3.44	0.7604	0.4046	20.52
Trochilidae	*Amazilia chionogaster*	Neotropics	1	-0.1479	10.0093	3.77	0.6763	0.4342	89.70
Trochilidae	*Amazilia cyanifrons*	Neotropics	0	1.5763	7.6714	3.85	0.7678	0.2714	52.53
Trochilidae	*Amazilia cyanocephala*	Neotropics	0	1.1027	7.8355	3.33	0.6961	0.2643	54.03
Trochilidae	*Amazilia cyanura*	Neotropics	0	-0.0484	6.2964	3.59	0.7542	0.2174	26.70
Trochilidae	*Amazilia decora*	Neotropics	0	0.1094	-	3.56	0.7050	0.1966	17.42
Trochilidae	*Amazilia edward*	Neotropics	0	0.0177	-	3.02	0.6428	0.1982	31.64
Trochilidae	*Amazilia fimbriata*	Neotropics	0	-0.0565	-	2.91	0.6842	0.2730	6.47
Trochilidae	*Amazilia franciae*	Neotropics	0	-0.2241	3.3564	3.91	0.8478	0.5668	29.17
Trochilidae	*Amazilia lactea*	Neotropics	0	0.4287	6.4671	3.31	0.7495	0.2633	77.76
Trochilidae	*Amazilia leucogaster*	Neotropics	0	-0.3460	-	3.83	0.7956	0.3636	24.36
Trochilidae	*Amazilia luciae*	Neotropics	0	-0.1097	-	3.43	0.7477	0.1722	40.04
Trochilidae	*Amazilia rosenbergi*	Neotropics	1	-0.1459	5.3982	3.46	0.6428	0.3515	4.44
Trochilidae	*Amazilia rutila*	Neotropics	0	0.2942	-	2.94	0.7030	0.2154	69.17
Trochilidae	*Amazilia saucerrottei*	Neotropics	0	-0.0124	-	3.42	0.7607	0.2688	22.13
Trochilidae	*Amazilia tobaci*	Neotropics	0	-0.2354	5.1863	3.39	0.7474	0.6462	16.29
Trochilidae	*Amazilia tzacatl*	Neotropics	0	-0.6086	5.5628	4.17	0.7586	0.6320	28.95
Trochilidae	*Amazilia versicolor*	Neotropics	0	-0.0045	5.0539	3.37	0.7847	0.4152	36.37
Trochilidae	*Amazilia violiceps*	Neotropics	0	-0.1366	4.8362	3.84	0.8300	0.4796	53.20
Trochilidae	*Amazilia viridicauda*	Nearctic	0	-0.4473	-	3.91	0.7651	0.2204	52.10
Trochilidae	*Amazilia viridifrons*	Neotropics	0	-14.9240	9.9683	4.25	0.7474	0.2236	56.69
Trochilidae	*Amazilia viridigaster*	Neotropics	0	0.8853	6.2578	3.55	0.7112	0.1438	9.41
Trochilidae	*Amazilia yucatanensis*	Neotropics	0	0.0572	8.1708	4.17	0.7522	0.4195	3.32
Trochilidae	*Androdon aequatorialis*	Neotropics	0	0.0652	4.4866	2.99	0.7198	0.2813	74.73
Trochilidae	*Anopetia gounellei*	Neotropics	0	0.0244	1.1972	3.72	0.7642	0.5864	59.12
Trochilidae	*Anthocephala floriceps*	Neotropics	1	-0.3525	8.4421	3.18	0.6763	0.0952	73.60
Trochilidae	*Anthracothorax dominicus*	Neotropics	0	0.1443	-	2.46	0.6906	0.2797	2.64
Trochilidae	*Anthracothorax mango*	Neotropics	0	-0.8576	5.6344	2.47	0.5985	0.0000	0.00
Trochilidae	*Anthracothorax nigricollis*	Neotropics	0	-0.1877	2.9782	3.84	0.8465	0.5559	48.43
Trochilidae	*Anthracothorax prevostii*	Neotropics	0	-0.1136	2.4809	3.41	0.7725	0.2312	20.47
Trochilidae	*Anthracothorax veraguensis*	Neotropics	0	-0.0093	-	2.93	0.6662	0.2443	24.93
Trochilidae	*Anthracothorax viridigula*	Neotropics	0	-0.0438	0.5159	3.68	0.7709	0.4703	39.39
Trochilidae	*Anthracothorax viridis*	Neotropics	0	0.4364	2.4478	2.41	0.5788	0.0000	0.00
Trochilidae	*Aphantochroa cirrochloris*	Neotropics	0	-0.4092	-	3.38	0.7846	0.5905	53.07
Trochilidae	*Archilochus alexandri*	IndoMalay	0	-0.1098	8.5230	2.85	0.7069	0.1324	52.64
Trochilidae	*Archilochus colubris*	IndoMalay	0	-0.0465	6.7061	3.78	0.7791	0.2524	15.09
Trochilidae	*Atthis ellioti*	Australasia	1	-1.2217	1.5144	2.28	0.5788	0.0000	35.75
Trochilidae	*Atthis heloisa*	Australasia	1	-0.4705	3.8277	3.14	0.6961	0.3300	75.19
Trochilidae	*Augastes lumachella*	Neotropics	0	-0.5426	6.8087	2.86	0.7643	0.4890	25.22

Family	Species	Realm	Threat	Latitude	Elevation	Anomaly	Size	Shape	Orientation
Trochilidae	*Augastes scutatus*	Neotropics	0	-0.2534	-	2.88	0.7512	0.2054	47.75
Trochilidae	*Avocettula recurvirostris*	IndoMalay	0	-0.0541	4.0374	2.91	0.8049	0.5684	30.88
Trochilidae	*Boissonneaua flavescens*	Afrotropics	1	0.0324	5.2764	3.07	0.7488	0.3051	4.22
Trochilidae	*Boissonneaua jardini*	Afrotropics	0	0.0609	5.9783	3.28	0.8060	0.4306	1.66
Trochilidae	*Boissonneaua matthewsii*	Afrotropics	0	-0.0158	9.1872	3.31	0.8199	0.3994	15.21
Trochilidae	*Calliphlox amethystina*	Palearctic	0	-0.7273	-	6.10	0.7877	0.0257	0.15
Trochilidae	*Calliphlox bryantae*	Palearctic	0	-1.0551	-	5.01	0.7574	0.1675	3.52
Trochilidae	*Calliphlox evelynae*	Palearctic	0	-1.4669	-	6.24	0.8212	0.0432	0.66
Trochilidae	*Calliphlox evelynae*	Palearctic	0	-1.4669	-	6.24	0.8212	-	-
Trochilidae	*Calliphlox mitchellii*	Palearctic	1	-0.8549	5.1736	5.25	0.7932	0.0283	0.29
Trochilidae	*Calothorax lucifer*	Neotropics	0	-0.1988	6.3731	3.05	0.6740	0.1907	33.57
Trochilidae	*Calypte anna*	Neotropics	0	0.0087	7.6216	3.03	0.7008	0.1697	70.72
Trochilidae	*Calypte costae*	Australasia	0	-0.6732	-	2.91	0.7474	0.3429	32.38
Trochilidae	*Campylopterus curvipennis*	Afrotropics	0	-0.1787	8.6905	3.90	0.8215	0.9252	45.58
Trochilidae	*Campylopterus duidae*	Afrotropics	0	-0.0577	-	3.41	0.8457	0.8212	8.15
Trochilidae	*Campylopterus ensipennis*	Afrotropics	0	0.0002	-	3.31	0.8213	0.4444	13.03
Trochilidae	*Campylopterus excellens*	Afrotropics	0	0.2798	-	3.17	0.7631	0.3375	23.65
Trochilidae	*Campylopterus falcatus*	Afrotropics	0	-0.0701	-	3.05	0.6961	0.1594	51.54
Trochilidae	*Campylopterus hemileucurus*	Afrotropics	0	-0.0115	-	3.29	0.8214	0.3357	16.42
Trochilidae	*Campylopterus hyperythrus*	Afrotropics	0	-0.7787	-	2.92	0.7096	0.1514	15.47
Trochilidae	*Campylopterus largipennis*	Afrotropics	0	-0.1771	9.4325	3.43	0.8188	0.7796	86.20
Trochilidae	*Campylopterus phainopeplus*	Afrotropics	0	-0.0564	-	3.46	0.8227	0.1754	9.09
Trochilidae	*Campylopterus rufus*	Afrotropics	0	0.1434	11.3112	3.30	0.7574	0.2572	13.44
Trochilidae	*Campylopterus villaviscensio*	Australasia	0	-0.0099	-	2.82	0.7452	0.2344	25.98
Trochilidae	*Chaetocercus astreans*	Palearctic	0	-0.8136	-	3.74	0.8001	0.4976	48.30
Trochilidae	*Chaetocercus berlepschi*	Palearctic	0	-0.6644	-	3.59	0.7990	0.4277	8.58
Trochilidae	*Chaetocercus bombus*	IndoMalay	0	-0.3780	3.3659	3.39	0.8072	0.3424	0.55
Trochilidae	*Chaetocercus heliodor*	Australasia	0	-0.5523	2.3947	2.50	0.5788	0.0000	45.57
Trochilidae	*Chaetocercus jourdanii*	Palearctic	0	-0.6358	11.6344	3.86	0.7992	0.3770	7.99
Trochilidae	*Chaetocercus mulsant*	IndoMalay	0	-0.0465	-	3.39	0.7898	0.2818	24.00
Trochilidae	*Chalcostigma heteropogon*	Australasia	0	-0.0947	-	2.78	0.7595	0.2055	23.87
Trochilidae	*Chalcostigma olivaceum*	IndoMalay	1	-0.1642	1.3013	3.39	0.7895	0.7870	32.04
Trochilidae	*Chalcostigma ruficeps*	Neotropics	0	-0.0239	-	3.98	0.8386	0.8538	38.51
Trochilidae	*Chalcostigma stanleyi*	Neotropics	0	-0.0491	2.9371	3.94	0.8107	0.3919	23.87
Trochilidae	*Chalybura buffonii*	Neotropics	0	-0.1779	4.5875	3.87	0.8341	0.5104	49.81
Trochilidae	*Chalybura urochrysia*	Neotropics	0	0.0681	-	4.15	0.7998	0.3737	17.60
Trochilidae	*Chlorestes notata*	Neotropics	0	-0.1355	-	3.97	0.8288	0.5528	8.05
Trochilidae	*Chlorostilbon alice*	IndoMalay	0	-0.0432	4.5807	3.31	0.7986	0.6631	78.44
Trochilidae	*Chlorostilbon assimilis*	IndoMalay	0	0.2883	8.7448	2.86	0.6935	0.2561	64.85
Trochilidae	*Chlorostilbon auriceps*	IndoMalay	0	0.0015	2.3149	2.77	0.7136	0.1260	55.84
Trochilidae	*Chlorostilbon canivetii*	IndoMalay	0	0.1714	-	2.52	0.5985	0.0024	45.41
Trochilidae	*Chlorostilbon gibsoni*	IndoMalay	0	-0.0264	-	2.83	0.7918	0.6013	13.79
Trochilidae	*Chlorostilbon lucidus*	IndoMalay	0	0.0709	4.8302	2.77	0.7261	0.1034	53.83
Trochilidae	*Chlorostilbon maugaeus*	Neotropics	0	0.1148	5.9029	3.59	0.7628	0.3257	74.29
Trochilidae	*Chlorostilbon melanorhynchus*	Neotropics	0	0.0630	6.6230	3.50	0.7542	0.4150	84.30
Trochilidae	*Chlorostilbon mellisugus*	Neotropics	0	-0.0604	6.3542	3.53	0.7544	0.3314	74.91
Trochilidae	*Chlorostilbon olivaresi*	Neotropics	1	-0.2858	3.5493	3.00	0.6740	0.1624	58.99
Trochilidae	*Chlorostilbon poortmani*	Neotropics	0	0.0995	3.6400	2.89	0.5788	0.0000	90.00
Trochilidae	*Chlorostilbon ricordii*	Neotropics	0	-0.1495	8.1439	3.67	0.7855	0.3073	53.76
Trochilidae	*Chlorostilbon russatus*	Neotropics	0	-5.8046	3.6000	3.65	0.7327	0.2886	62.16
Trochilidae	*Chlorostilbon stenurus*	Neotropics	0	-0.1327	8.5123	3.03	0.6662	0.2621	35.03
Trochilidae	*Chlorostilbon swainsonii*	Neotropics	0	-0.0943	7.2397	3.00	0.6948	0.1369	65.99
Trochilidae	*Chrysolampis mosquitus*	Neotropics	0	0.3879	2.0580	3.97	0.8021	0.7682	74.67
Trochilidae	*Chrysuronia oenone*	Australasia	0	-0.1860	3.5047	3.10	0.8077	0.1886	33.92
Trochilidae	*Clytolaema rubricauda*	Afrotropics	0	-0.2813	7.8638	3.60	0.8557	0.6758	52.21
Trochilidae	*Coeligena bonapartei*	Nearctic	0	-0.6924	-	4.13	0.8309	0.6346	11.43
Trochilidae	*Coeligena coeligena*	Nearctic	0	-0.5904	-	4.36	0.8286	0.4048	7.36
Trochilidae	*Coeligena helianthea*	Neotropics	0	-0.1470	2.9405	3.88	0.8431	0.5596	48.80
Trochilidae	*Coeligena iris*	Neotropics	0	0.1189	1.8537	3.45	0.7482	0.2285	55.31
Trochilidae	*Coeligena lutetiae*	Neotropics	0	-0.1359	5.3885	2.46	0.6876	0.3855	3.42
Trochilidae	*Coeligena orina*	Neotropics	0	-0.3062	3.4651	3.75	0.8547	0.6892	50.08
Trochilidae	*Coeligena phalerata*	Neotropics	0	-0.2797	1.3871	2.53	0.6986	0.2608	17.52
Trochilidae	*Coeligena prunellei*	Neotropics	0	-0.0746	-	3.29	0.7849	0.3002	16.28
Trochilidae	*Coeligena torquata*	Neotropics	0	0.5277	5.6344	2.47	0.5985	0.0000	0.00
Trochilidae	*Coeligena violifer*	Neotropics	1	-0.4126	2.8984	2.46	0.6716	0.4366	13.45
Trochilidae	*Coeligena wilsoni*	Neotropics	0	-0.9642	4.5531	2.47	0.5985	0.0000	0.00
Trochilidae	*Colibri coruscans*	Neotropics	1	29.4471	9.5756	3.62	0.6598	0.3511	54.35
Trochilidae	*Colibri delphinae*	Neotropics	0	0.0537	8.0295	3.63	0.7697	0.3788	77.45
Trochilidae	*Colibri serrirostris*	Neotropics	0	-2.0023	13.1285	3.97	0.7378	0.1721	39.46
Trochilidae	*Colibri thalassinus*	Neotropics	0	0.2237	6.3268	3.04	0.7019	0.1015	66.91
Trochilidae	*Cynanthus latirostris*	Palearctic	0	-0.7907	5.0982	3.99	0.8286	0.2187	2.33
Trochilidae	*Cynanthus sordidus*	Neotropics	1	-11.0769	-	3.82	0.7217	0.4780	20.52
Trochilidae	*Damophila julie*	Neotropics	0	-0.6463	5.9451	3.64	0.7112	0.2926	17.87
Trochilidae	*Discosura conversii*	Neotropics	0	-0.4857	6.6375	3.80	0.7416	0.2994	56.89

Family	Species	Realm	Threat	Latitude	Elevation	Anomaly	Size	Shape	Orientation
Trochilidae	*Discosura langsdorffi*	Neotropics	0	0.1356	6.1341	4.08	0.6662	0.2905	42.51
Trochilidae	*Discosura letitiae*	Neotropics	1	-3.2078	4.9290	3.10	0.6370	0.3277	79.39
Trochilidae	*Discosura longicaudus*	Neotropics	0	-0.0020	10.3969	3.35	0.7406	0.1870	62.94
Trochilidae	*Discosura popelairii*	Neotropics	0	0.6717	7.7364	2.98	0.6876	0.1378	66.36
Trochilidae	*Doricha enicura*	Neotropics	1	-0.5953	-	2.34	0.7515	0.0573	0.12
Trochilidae	*Doryfera johannae*	Neotropics	0	-0.1591	5.4072	3.93	0.8242	0.7294	58.13
Trochilidae	*Doryfera ludovicae*	Afrotropics	0	0.0044	-	3.54	0.7060	0.2757	79.21
Trochilidae	*Elvira chionura*	Neotropics	0	0.1259	13.3455	3.85	0.7713	0.6779	60.31
Trochilidae	*Elvira cupreiceps*	Neotropics	0	-0.6176	-	3.03	0.8105	0.8810	81.04
Trochilidae	*Ensifera ensifera*	Neotropics	0	0.0788	11.3435	3.48	0.7413	0.2711	33.38
Trochilidae	*Eriocnemis alinae*	Afrotropics	0	-0.2771	-	3.91	0.7746	0.3167	60.71
Trochilidae	*Eriocnemis cupreoventris*	Australasia	0	-0.5103	-	3.66	0.8016	0.2725	0.08
Trochilidae	*Eriocnemis cupreoventris*	Australasia	0	-0.5103	-	3.66	0.8016	-	-
Trochilidae	*Eriocnemis derbyi*	Afrotropics	0	-1.9043	-	3.76	0.7860	0.4656	2.79
Trochilidae	*Eriocnemis glaucopoides*	Afrotropics	0	-0.0258	-	3.28	0.8170	0.3443	14.57
Trochilidae	*Eriocnemis godini*	Afrotropics	0	-0.0162	-	3.33	0.7820	0.5099	34.01
Trochilidae	*Eriocnemis isabellae*	Afrotropics	0	-0.2202	-	3.50	0.7449	0.3589	42.32
Trochilidae	*Eriocnemis luciani*	Afrotropics	0	-0.0867	4.4701	3.72	0.8446	0.6416	54.75
Trochilidae	*Eriocnemis mirabilis*	Afrotropics	0	0.0300	-	3.49	0.8299	0.1780	4.73
Trochilidae	*Eriocnemis mosquera*	Afrotropics	0	-0.1783	-	3.63	0.8269	0.7453	12.46
Trochilidae	*Eriocnemis nigrivestis*	Afrotropics	1	-0.1783	6.9937	3.32	0.6370	0.0445	16.95
Trochilidae	*Eriocnemis vestita*	Afrotropics	0	-0.2284	-	4.00	0.7965	0.6730	0.89
Trochilidae	*Eugenes fulgens*	Afrotropics	0	0.1056	-	3.29	0.7925	0.2557	12.29
Trochilidae	*Eulampis holosericeus*	Afrotropics	0	0.4709	9.8064	3.66	0.7179	0.7828	31.10
Trochilidae	*Eulidia yarrellii*	Afrotropics	0	-0.0112	1.5971	3.54	0.7374	0.2663	81.28
Trochilidae	*Eupetomena macroura*	Palearctic	0	-0.8196	-	5.66	0.8264	0.1215	0.29
Trochilidae	*Eupherusa cyanophrys*	IndoMalay	0	-0.1761	3.8799	3.26	0.8459	0.6350	15.63
Trochilidae	*Eupherusa eximia*	Australasia	0	-0.4908	-	2.36	0.7245	0.2482	43.75
Trochilidae	*Eupherusa poliocerca*	Australasia	1	-0.5088	0.4396	2.20	0.6921	0.1358	3.36
Trochilidae	*Eutoxeres aquila*	Australasia	0	-0.0480	8.3233	2.83	0.7305	0.1663	22.31
Trochilidae	*Eutoxeres condamini*	Australasia	0	-0.5205	-	3.36	0.8358	0.6079	3.37
Trochilidae	*Florisuga fusca*	IndoMalay	0	-0.0512	2.1514	2.69	0.6370	0.4217	50.01
Trochilidae	*Florisuga mellivora*	IndoMalay	0	-0.0435	4.3683	2.82	0.7696	0.5261	8.90
Trochilidae	*Glaucis aeneus*	IndoMalay	0	0.0033	-	2.74	0.7842	0.6784	12.42
Trochilidae	*Glaucis dohrnii*	Australasia	0	-0.6515	-	3.24	0.7030	0.1604	35.35
Trochilidae	*Glaucis hirsutus*	Afrotropics	0	0.1804	-	3.34	0.8182	0.4611	34.42
Trochilidae	*Goethalsia bella*	Afrotropics	1	10.5505	3.5974	3.42	0.6948	0.3084	83.92
Trochilidae	*Goldmania violiceps*	Neotropics	0	3.6334	9.2069	3.89	0.7228	0.2542	65.95
Trochilidae	*Haplophaedia assimilis*	IndoMalay	0	0.0565	2.9488	2.91	0.7096	0.2272	61.21
Trochilidae	*Haplophaedia aureliae*	IndoMalay	0	-0.1244	9.5443	3.60	0.7659	0.2489	4.35
Trochilidae	*Haplophaedia lugens*	Afrotropics	0	-0.1671	-	3.53	0.8215	0.9441	61.39
Trochilidae	*Heliactin bilophus*	Nearctic	0	-0.2977	0.0000	3.50	0.6842	0.3584	48.04
Trochilidae	*Heliangelus amethysticollis*	Australasia	0	-0.5009	1.8615	3.46	0.8296	0.5674	5.52
Trochilidae	*Heliangelus exortis*	Neotropics	1	0.0812	5.9471	3.65	0.7211	0.1995	51.25
Trochilidae	*Heliangelus mavors*	Neotropics	1	0.0000	5.9950	3.05	0.6120	0.9804	90.00
Trochilidae	*Heliangelus micraster*	Neotropics	0	-3.9478	9.9751	4.01	0.6961	0.0830	34.26
Trochilidae	*Heliangelus regalis*	Neotropics	1	0.1248	5.9900	3.34	0.6763	0.2983	74.82
Trochilidae	*Heliangelus strophianus*	Neotropics	0	-0.4992	11.0344	3.37	0.6921	0.1835	86.89
Trochilidae	*Heliangelus viola*	Neotropics	0	2.3072	11.5378	4.10	0.7639	0.2398	42.33
Trochilidae	*Heliangelus zusii*	Neotropics	0	-3.9770	5.9620	3.97	0.7314	0.1639	41.41
Trochilidae	*Heliodoxa aurescens*	Neotropics	0	-0.0123	7.3355	3.61	0.7804	0.3319	53.54
Trochilidae	*Heliodoxa branickii*	Neotropics	0	-0.4630	-	3.05	0.7813	0.3633	34.02
Trochilidae	*Heliodoxa gularis*	IndoMalay	0	-0.0106	-	2.64	0.7261	0.2111	67.49
Trochilidae	*Heliodoxa imperatrix*	IndoMalay	0	0.0046	1.6651	2.84	0.7848	0.4856	5.86
Trochilidae	*Heliodoxa jacula*	IndoMalay	0	0.0106	2.0271	2.84	0.7887	0.5174	10.28
Trochilidae	*Heliodoxa leadbeateri*	IndoMalay	0	-0.1014	9.5698	3.20	0.8128	0.9283	71.41
Trochilidae	*Heliodoxa rubinoides*	IndoMalay	0	0.0220	-	3.12	0.7576	0.7059	54.81
Trochilidae	*Heliodoxa schreibersii*	IndoMalay	0	0.0616	2.0941	2.84	0.7818	0.4794	3.61
Trochilidae	*Heliodoxa xanthogonys*	IndoMalay	0	-0.0574	5.7247	3.01	0.7996	0.4686	71.00
Trochilidae	*Heliomaster constantii*	Palearctic	0	0.0911	9.1935	3.83	0.7335	0.3671	16.26
Trochilidae	*Heliomaster furcifer*	IndoMalay	0	-0.2215	3.4755	2.87	0.6876	0.4193	59.50
Trochilidae	*Heliomaster longirostris*	Neotropics	0	-0.1197	-	3.89	0.8439	0.5008	30.21
Trochilidae	*Heliomaster squamosus*	Neotropics	0	-0.0643	-	3.72	0.8211	0.7946	52.04
Trochilidae	*Heliothryx auritus*	Neotropics	0	-0.0805	8.1424	3.71	0.7852	0.2152	47.55
Trochilidae	*Heliothryx barroti*	Australasia	1	-0.0907	4.2201	2.79	0.7707	0.2954	24.22
Trochilidae	*Hylocharis chrysura*	Afrotropics	0	0.1694	-	3.42	0.7939	0.2384	7.21
Trochilidae	*Hylocharis cyanus*	Afrotropics	0	0.1094	5.7785	3.57	0.8085	0.2337	8.08
Trochilidae	*Hylocharis eliciae*	Afrotropics	1	0.0000	1.2753	3.36	0.6598	0.7653	32.81
Trochilidae	*Hylocharis grayi*	Australasia	0	-0.5439	-	3.14	0.8223	0.3586	8.17
Trochilidae	*Hylocharis humboldtii*	Australasia	0	-0.5368	-	3.32	0.8342	0.5225	11.26
Trochilidae	*Hylocharis leucotis*	Afrotropics	0	0.0201	-	3.32	0.8219	0.4026	19.67
Trochilidae	*Hylocharis sapphirina*	Afrotropics	0	-3.3971	-	3.73	0.7696	0.2334	10.89
Trochilidae	*Hylocharis xantusii*	Palearctic	0	-0.2334	13.4421	4.10	0.8358	0.1922	1.31
Trochilidae	*Klais guimeti*	Palearctic	0	-0.9125	11.1796	3.95	0.7789	0.2890	16.34

Family	Species	Realm	Threat	Latitude	Elevation	Anomaly	Size	Shape	Orientation
Trochilidae	*Lafresnaya lafresnayi*	IndoMalay	0	-0.1377	-	2.64	0.6523	0.4138	2.64
Trochilidae	*Lampornis amethystinus*	Neotropics	0	-0.5603	-	2.69	0.7642	0.6975	80.02
Trochilidae	*Lampornis calolaemus*	Neotropics	0	-0.4196	-	3.44	0.7916	0.6325	78.56
Trochilidae	*Lampornis castaneoventris*	Neotropics	0	-0.2793	11.4222	3.21	0.7602	0.2302	58.56
Trochilidae	*Lampornis clemenciae*	Neotropics	0	0.1214	-	4.11	0.7763	0.4343	30.92
Trochilidae	*Lampornis hemileucus*	Neotropics	0	0.0703	-	4.06	0.7950	0.5224	23.52
Trochilidae	*Lampornis sybillae*	Neotropics	0	0.1263	11.6247	3.78	0.7716	0.6076	50.42
Trochilidae	*Lampornis viridipallens*	Neotropics	0	1.1345	11.7715	3.86	0.7435	0.2142	53.01
Trochilidae	*Lamprolaima rhami*	Neotropics	0	-0.4151	5.4625	3.40	0.7803	0.4216	68.55
Trochilidae	*Lepidopyga coeruleogularis*	Afrotropics	0	0.1412	8.3651	3.40	0.7803	0.4661	22.93
Trochilidae	*Lepidopyga goudoti*	Afrotropics	0	-0.0732	6.1695	3.71	0.8034	0.5877	6.43
Trochilidae	*Lepidopyga lilliae*	Palearctic	0	-0.8888	14.5834	4.09	0.8088	0.2195	25.59
Trochilidae	*Lesbia nuna*	Neotropics	0	-0.2304	4.6851	3.78	0.8476	0.7495	37.52
Trochilidae	*Lesbia victoriae*	Neotropics	1	0.1005	4.5626	3.18	0.7136	0.3042	88.33
Trochilidae	*Leucippus baeri*	IndoMalay	0	-0.0090	-	3.27	0.7957	0.5564	58.90
Trochilidae	*Leucippus chlorocercus*	Palearctic	0	-0.4116	-	3.51	0.7966	0.1585	2.68
Trochilidae	*Leucippus fallax*	Australasia	1	-0.8368	-	2.95	0.7797	0.1965	6.99
Trochilidae	*Leucippus taczanowskii*	IndoMalay	0	-0.2057	7.8569	2.64	0.6842	0.8662	65.43
Trochilidae	*Leucochloris albicollis*	Neotropics	0	-0.0827	10.4795	3.54	0.7510	0.2157	30.50
Trochilidae	*Loddigesia mirabilis*	Australasia	0	-0.3246	-	2.17	0.6804	0.6686	14.77
Trochilidae	*Lophornis adorabilis*	Neotropics	0	-0.1175	4.6456	4.36	0.6921	0.4969	3.12
Trochilidae	*Lophornis brachylophus*	Neotropics	0	0.0970	-	3.33	0.7647	0.2476	36.48
Trochilidae	*Lophornis chalybeus*	Neotropics	1	-5.2165	5.8115	4.02	0.6891	0.2823	37.37
Trochilidae	*Lophornis delattrei*	Neotropics	0	-0.1263	-	4.02	0.8372	0.7048	9.25
Trochilidae	*Lophornis gouldii*	Neotropics	1	0.0000	1.9670	3.32	0.5985	0.5771	46.13
Trochilidae	*Lophornis helenae*	Australasia	0	-0.0723	7.1261	2.81	0.7482	0.1527	24.89
Trochilidae	*Lophornis magnificus*	Neotropics	0	-0.2467	10.6311	3.44	0.8134	0.5666	48.13
Trochilidae	*Lophornis ornatus*	Palearctic	0	-0.9051	6.3500	4.71	0.8519	0.3933	5.99
Trochilidae	*Lophornis pavoninus*	Palearctic	0	-0.6811	-	4.61	0.8204	0.2120	6.38
Trochilidae	*Lophornis stictolophus*	Palearctic	0	-0.3760	-	4.62	0.8335	0.8058	19.09
Trochilidae	*Mellisuga helenae*	Australasia	0	-0.0680	-	2.83	0.7367	0.1524	25.09
Trochilidae	*Mellisuga minima*	Australasia	0	-0.0801	-	2.80	0.7425	0.1705	24.62
Trochilidae	*Metallura aeneocauda*	Australasia	0	-0.0844	-	2.79	0.7539	0.1843	23.37
Trochilidae	*Metallura baroni*	Australasia	0	0.1388	4.5518	2.56	0.6523	0.1875	30.24
Trochilidae	*Metallura eupogon*	Australasia	0	-0.1698	11.4959	2.79	0.6370	0.3627	22.80
Trochilidae	*Metallura iracunda*	Australasia	0	-0.0695	11.0281	2.83	0.7406	0.2010	24.98
Trochilidae	*Metallura odomae*	Australasia	0	-0.1670	8.5830	2.52	0.6428	0.2419	46.04
Trochilidae	*Metallura phoebe*	Australasia	0	-0.2940	-	2.17	0.6804	0.6686	14.77
Trochilidae	*Metallura theresiae*	Australasia	0	-0.3387	1.5266	3.28	0.7941	0.4284	21.41
Trochilidae	*Metallura tyrianthina*	Australasia	0	-0.6317	-	3.07	0.8021	0.2959	2.58
Trochilidae	*Metallura williami*	Australasia	0	-0.5484	-	3.43	0.8153	0.4616	24.80
Trochilidae	*Microchera albocoronata*	Australasia	0	-0.5391	-	3.12	0.7474	0.2825	59.46
Trochilidae	*Microstilbon burmeisteri*	Afrotropics	0	0.1198	9.7593	3.31	0.7619	0.8042	13.32
Trochilidae	*Myrmia micrura*	Neotropics	0	-0.0981	8.2495	3.04	0.6784	0.1941	29.99
Trochilidae	*Myrtis fanny*	IndoMalay	0	-0.5246	8.5164	2.72	0.6948	0.1004	11.03
Trochilidae	*Ocreatus underwoodii*	Neotropics	0	-0.0525	1.8130	3.98	0.8313	0.4426	41.53
Trochilidae	*Opisthoprora euryptera*	Neotropics	0	0.0255	9.6268	3.63	0.7669	0.3744	75.97
Trochilidae	*Oreonympha nobilis*	IndoMalay	0	0.0488	-	2.90	0.7385	0.4014	51.91
Trochilidae	*Oreotrochilus adela*	Neotropics	0	0.0732	-	3.07	0.7344	0.1654	67.25
Trochilidae	*Oreotrochilus chimborazo*	Neotropics	0	0.0107	-	3.99	0.8349	0.7055	16.23
Trochilidae	*Oreotrochilus estella*	Neotropics	0	-0.0657	10.1537	3.51	0.7556	0.3639	31.39
Trochilidae	*Oreotrochilus leucopleurus*	Nearctic	0	-0.2891	4.7984	4.05	0.6961	0.5281	70.66
Trochilidae	*Oreotrochilus melanogaster*	Neotropics	0	-0.3104	6.1701	3.04	0.6763	0.1815	29.74
Trochilidae	*Orthorhyncus cristatus*	Neotropics	0	-19.8811	5.9989	3.27	0.6921	0.1102	68.19
Trochilidae	*Oxypogon guerinii*	Australasia	0	-0.0839	-	2.78	0.7705	0.2936	23.75
Trochilidae	*Panterpe insignis*	Afrotropics	1	0.0268	1.3353	3.01	0.5985	0.1021	61.50
Trochilidae	*Patagona gigas*	Palearctic	0	-0.5741	6.0009	4.34	0.8347	0.3148	14.00
Trochilidae	*Phaeochroa cuvierii*	Neotropics	1	0.0000	0.9546	3.83	0.6370	0.6952	31.30
Trochilidae	*Phaethornis aethopyga*	IndoMalay	0	-0.0632	-	3.38	0.7979	0.5381	81.56
Trochilidae	*Phaethornis anthophilus*	IndoMalay	0	-0.1301	8.0215	3.23	0.7678	0.5010	50.37
Trochilidae	*Phaethornis atrimentalis*	IndoMalay	1	0.1265	2.2110	3.30	0.7314	0.7725	12.56
Trochilidae	*Phaethornis augusti*	Palearctic	0	-0.6753	-	4.35	0.8378	0.5112	2.82
Trochilidae	*Phaethornis bourcieri*	Palearctic	0	-1.3055	11.8947	4.50	0.7852	0.3442	6.22
Trochilidae	*Phaethornis eurynome*	Palearctic	0	-0.5776	-	4.51	0.8476	0.2576	0.92
Trochilidae	*Phaethornis griseogularis*	IndoMalay	0	-0.1347	-	3.58	0.8086	0.3859	16.25
Trochilidae	*Phaethornis guy*	Palearctic	0	-0.3790	3.4469	3.33	0.8013	0.6175	1.65
Trochilidae	*Phaethornis hispidus*	IndoMalay	0	-0.1093	-	3.33	0.8272	0.4549	24.21
Trochilidae	*Phaethornis idaliae*	Palearctic	0	-1.0013	-	4.00	0.7789	0.7471	78.03
Trochilidae	*Phaethornis koepckeae*	IndoMalay	0	-0.1325	-	3.40	0.7998	0.5663	3.18
Trochilidae	*Phaethornis longirostris*	Palearctic	0	-0.6545	-	3.64	0.8230	0.5447	9.79
Trochilidae	*Phaethornis longuemareus*	IndoMalay	0	-0.1382	-	3.13	0.8343	0.6849	29.44
Trochilidae	*Phaethornis malaris*	IndoMalay	0	0.0048	-	2.83	0.7880	0.5092	7.29
Trochilidae	*Phaethornis nattereri*	Australasia	0	0.3411	-	2.63	0.6478	0.0000	0.00
Trochilidae	*Phaethornis philippii*	IndoMalay	0	-0.0280	-	2.75	0.7389	0.1614	40.79

Family	Species	Realm	Threat	Latitude	Elevation	Anomaly	Size	Shape	Orientation
Trochilidae	*Phaethornis pretrei*	IndoMalay	0	-0.1889	-	3.51	0.7978	0.2160	17.51
Trochilidae	*Phaethornis ruber*	IndoMalay	0	0.3601	-	3.24	0.8122	0.5422	0.65
Trochilidae	*Phaethornis rupurumii*	Palearctic	0	-0.8255	-	2.90	0.6690	0.6105	70.81
Trochilidae	*Phaethornis squalidus*	Neotropics	0	0.0139	-	3.68	0.7783	0.2794	37.35
Trochilidae	*Phaethornis striigularis*	Nearctic	0	-0.9216	-	4.29	0.8414	0.2294	8.42
Trochilidae	*Phaethornis stuarti*	Palearctic	0	-0.1078	-	5.16	0.8496	0.1396	2.52
Trochilidae	*Phaethornis subochraceus*	Palearctic	1	3.4274	7.5706	4.15	0.7228	0.5517	31.63
Trochilidae	*Phaethornis superciliosus*	Neotropics	0	-0.0029	-	4.07	0.7960	0.5937	10.54
Trochilidae	*Phaethornis syrmatophorus*	Palearctic	0	-0.6052	4.5899	4.52	0.8451	0.2757	6.32
Trochilidae	*Phaethornis yaruqui*	IndoMalay	0	-0.0080	5.3036	2.67	0.7503	0.2491	86.29
Trochilidae	*Phlogophilus harterti*	Neotropics	0	-0.0653	5.6307	3.41	0.7725	0.1585	23.41
Trochilidae	*Phlogophilus hemileucurus*	Neotropics	0	0.0711	1.1751	3.67	0.7435	0.1899	29.27
Trochilidae	*Polyonymus caroli*	Afrotropics	0	-19.4049	-	3.81	0.7179	0.4002	16.35
Trochilidae	*Polytmus guainumbi*	IndoMalay	0	-0.8862	8.3794	2.61	0.6478	0.3444	72.46
Trochilidae	*Polytmus milleri*	IndoMalay	0	0.5457	7.7521	4.00	0.6935	0.1755	16.21
Trochilidae	*Polytmus theresiae*	IndoMalay	0	-0.2463	10.8687	3.31	0.8218	0.9092	35.73
Trochilidae	*Pterophanes cyanopterus*	Neotropics	0	-0.0630	-	3.47	0.7623	0.2669	31.20
Trochilidae	*Ramphodon naevius*	Palearctic	0	-0.5696	-	3.58	0.7706	0.3556	3.86
Trochilidae	*Ramphomicron dorsale*	Neotropics	0	-0.4232	6.3823	3.06	0.7648	0.2318	25.94
Trochilidae	*Ramphomicron microrhynchum*	Neotropics	0	-0.0599	5.4267	3.86	0.7458	0.2409	52.07
Trochilidae	*Rhodopis vesper*	Afrotropics	0	-0.0180	-	3.46	0.8061	0.1275	4.36
Trochilidae	*Sappho sparganura*	Afrotropics	0	0.0773	5.4452	3.21	0.8009	0.2055	11.78
Trochilidae	*Schistes geoffroyi*	IndoMalay	0	-0.4488	9.9676	2.72	0.6859	0.0948	10.27
Trochilidae	*Selasphorus ardens*	Neotropics	0	-0.1846	-	3.68	0.7693	0.5778	88.15
Trochilidae	*Selasphorus flammula*	Neotropics	0	0.0059	-	4.11	0.7968	0.7679	5.63
Trochilidae	*Selasphorus platycercus*	Neotropics	0	0.1015	-	3.49	0.7172	0.4630	6.04
Trochilidae	*Selasphorus rufus*	Neotropics	0	0.0375	-	3.57	0.7399	0.5274	38.43
Trochilidae	*Selasphorus sasin*	Nearctic	0	-0.5963	15.6555	4.08	0.8306	0.4704	54.24
Trochilidae	*Selasphorus scintilla*	Afrotropics	0	-0.1629	-	3.53	0.8194	0.3919	20.88
Trochilidae	*Sephanoides sephaniodes*	Neotropics	0	-0.1269	-	3.92	0.8458	0.4842	32.12
Trochilidae	*Stellula calliope*	Palearctic	0	-3.9603	9.0019	4.26	0.7772	0.2310	0.71
Trochilidae	*Stephanoxis lalandi*	IndoMalay	0	0.0011	-	2.64	0.7305	0.2218	67.89
Trochilidae	*Sternoclyta cyanopectus*	Palearctic	0	-0.9225	-	4.22	0.8123	0.4506	9.69
Trochilidae	*Taphrolesbia griseiventris*	Afrotropics	0	-0.4119	-	3.57	0.7755	0.4647	37.42
Trochilidae	*Taphrospilus hypostictus*	Afrotropics	0	-0.3046	8.1206	3.73	0.7724	0.4375	45.77
Trochilidae	*Thalurania fannyi*	Neotropics	0	-0.0755	1.3214	3.42	0.7782	0.2191	28.01
Trochilidae	*Thalurania furcata*	Neotropics	0	-0.1707	1.9510	3.88	0.8504	0.6085	35.27
Trochilidae	*Thalurania glaucopis*	Nearctic	0	-0.7444	0.2968	4.26	0.8514	0.2940	13.22
Trochilidae	*Thalurania watertonii*	Neotropics	1	0.3231	6.6016	2.47	0.6716	0.1415	5.68
Trochilidae	*Thaumastura cora*	Neotropics	0	0.0692	-	4.04	0.8263	0.8507	24.65
Trochilidae	*Threnetes leucurus*	Neotropics	0	-0.0016	8.5007	3.66	0.7711	0.4070	81.37
Trochilidae	*Threnetes niger*	Neotropics	0	0.0319	-	2.96	0.7040	0.2939	77.18
Trochilidae	*Threnetes ruckeri*	Neotropics	0	0.1665	8.5810	3.53	0.7477	0.2621	82.67
Trochilidae	*Topaza pella*	Neotropics	0	-0.0309	2.7026	3.98	0.8175	0.5010	23.06
Trochilidae	*Topaza pyra*	Neotropics	0	-0.0851	-	3.97	0.8331	0.5139	25.62
Trochilidae	*Trochilus polytmus*	Neotropics	0	-0.0700	-	3.99	0.8422	0.5587	35.01
Trochilidae	*Trochilus scitulus*	Neotropics	0	-0.1788	-	3.38	0.7913	0.3327	69.28
Trochilidae	*Urochroa bougueri*	Neotropics	0	-0.0628	1.8369	3.36	0.7606	0.5018	33.30
Trochilidae	*Urosticte benjamini*	IndoMalay	0	0.0504	8.8131	2.77	0.7223	0.1018	53.79
Trochilidae	*Urosticte ruficrissa*	IndoMalay	0	0.0958	-	3.19	0.7705	0.5998	86.35
Troglodytidae	*Campylorhynchus albobrunneus*	Neotropics	0	0.0846	7.1025	3.64	0.6302	0.4147	18.92
Troglodytidae	*Campylorhynchus brunneicapillus*	Neotropics	0	-1.0820	2.6517	3.70	0.6763	0.5004	24.73
Troglodytidae	*Campylorhynchus chiapensis*	Neotropics	0	0.0884	10.8748	3.75	0.7272	0.2476	51.12
Troglodytidae	*Campylorhynchus fasciatus*	Neotropics	0	-0.0388	8.6656	3.51	0.7569	0.2125	31.16
Troglodytidae	*Campylorhynchus griseus*	Neotropics	0	0.1074	7.8573	4.41	0.6876	0.6222	13.84
Troglodytidae	*Campylorhynchus gularis*	Neotropics	0	-0.0466	1.4068	4.04	0.8351	0.7293	0.42
Troglodytidae	*Campylorhynchus jocosus*	Neotropics	1	0.4549	18.0764	3.24	0.5985	0.5771	46.13
Troglodytidae	*Campylorhynchus megalopterus*	Neotropics	0	-0.2775	6.1407	3.58	0.6662	0.1917	28.49
Troglodytidae	*Campylorhynchus nuchalis*	Neotropics	0	-5.5741	2.6847	3.32	0.6824	0.1322	69.70
Troglodytidae	*Campylorhynchus rufinucha*	Neotropics	0	-0.2980	-	3.01	0.7792	0.2598	41.09
Troglodytidae	*Campylorhynchus turdinus*	Neotropics	0	-0.0423	-	4.02	0.8180	0.6495	3.47
Troglodytidae	*Campylorhynchus yucatanicus*	Neotropics	0	0.0139	8.5351	3.25	0.7512	0.5467	86.95
Troglodytidae	*Campylorhynchus zonatus*	Neotropics	0	-0.1566	-	3.83	0.8397	0.6799	35.51
Troglodytidae	*Cinnycerthia fulva*	Neotropics	0	-0.5189	2.0719	2.26	0.6740	0.3574	56.60
Troglodytidae	*Cinnycerthia olivascens*	Neotropics	1	-25.2812	2.5081	4.02	0.6906	0.6748	51.58
Troglodytidae	*Cinnycerthia peruana*	Neotropics	0	-0.5790	8.0255	2.25	0.7691	0.2192	88.33
Troglodytidae	*Cinnycerthia unirufa*	Australasia	0	-0.6223	-	3.34	0.8307	0.3898	6.82
Troglodytidae	*Cistothorus apolinari*	Afrotropics	0	-17.2685	-	3.67	0.7586	0.5108	17.28
Troglodytidae	*Cistothorus meridae*	Afrotropics	0	0.0409	-	3.26	0.7737	0.3689	77.52
Troglodytidae	*Cistothorus palustris*	Afrotropics	0	-0.0880	-	3.45	0.8436	0.5652	29.73
Troglodytidae	*Cistothorus platensis*	Afrotropics	0	-0.7949	-	3.42	0.7158	0.6075	21.53

Family	Species	Realm	Threat	Latitude	Elevation	Anomaly	Size	Shape	Orientation
Troglodytidae	*Cyphorhinus arada*	IndoMalay	0	-0.1041	0.0000	3.11	0.8012	0.3831	70.94
Troglodytidae	*Cyphorhinus phaeocephalus*	IndoMalay	1	0.0800	1.0273	2.85	0.7828	0.4026	4.42
Troglodytidae	*Cyphorhinus thoracicus*	IndoMalay	0	-0.0391	-	2.97	0.7864	0.4865	64.44
Troglodytidae	*Ferminia cerverai*	Neotropics	0	-0.3305	10.9624	3.54	0.8531	0.5586	51.50
Troglodytidae	*Henicorhina leucophrys*	Neotropics	0	-3.5728	4.3711	4.07	0.7217	0.1320	33.39
Troglodytidae	*Henicorhina leucoptera*	Neotropics	0	-0.2541	5.4319	3.27	0.7136	0.1511	65.90
Troglodytidae	*Henicorhina leucosticta*	Neotropics	0	-6.0326	4.3348	3.96	0.7266	0.1682	44.18
Troglodytidae	*Henicorhina negreti*	Afrotropics	0	11.3196	-	3.43	0.7019	0.3874	83.36
Troglodytidae	*Hylorchilus navai*	Neotropics	0	0.1148	-2.8683	2.88	0.6740	0.2295	75.99
Troglodytidae	*Hylorchilus sumichrasti*	Neotropics	0	-0.0298	13.1929	3.84	0.7736	0.2248	34.70
Troglodytidae	*Microcerculus bambla*	Nearctic	0	-0.5224	14.7902	4.16	0.7980	0.5704	57.21
Troglodytidae	*Microcerculus marginatus*	Neotropics	0	-0.1879	-	3.74	0.7528	0.2208	40.12
Troglodytidae	*Microcerculus philomela*	Neotropics	0	0.1889	7.4171	3.50	0.6921	0.1874	28.08
Troglodytidae	*Microcerculus ustulatus*	Australasia	0	-0.2519	-	3.18	0.6690	0.5976	82.69
Troglodytidae	*Odontorchilus branickii*	Neotropics	0	0.0232	12.1865	3.71	0.7705	0.3901	82.31
Troglodytidae	*Odontorchilus cinereus*	Neotropics	0	-0.0160	8.7493	3.63	0.7136	0.4357	27.05
Troglodytidae	*Salpinctes obsoletus*	Nearctic	0	-0.9327	-	4.23	0.8461	0.2867	8.16
Troglodytidae	*Thryomanes bewickii*	IndoMalay	0	-0.0171	-	2.67	0.7488	0.2439	86.29
Troglodytidae	*Thryorchilus browni*	Australasia	0	-0.4944	2.7864	2.61	0.6842	0.3092	41.12
Troglodytidae	*Thryothorus atrogularis*	Australasia	0	-0.0747	-	2.76	0.7724	0.2722	21.79
Troglodytidae	*Thryothorus coraya*	Australasia	0	-0.0811	-	2.78	0.6842	0.2108	17.03
Troglodytidae	*Thryothorus eisenmanni*	Australasia	0	-0.0411	2.3278	2.62	0.7087	0.1186	17.02
Troglodytidae	*Thryothorus euophrys*	Australasia	0	-0.2664	-	2.87	0.7234	0.3980	81.75
Troglodytidae	*Thryothorus fasciatoventris*	Neotropics	1	-0.2924	2.3524	3.35	0.7507	0.3001	24.33
Troglodytidae	*Thryothorus felix*	Neotropics	0	-0.2064	4.4303	3.77	0.8523	0.5624	41.27
Troglodytidae	*Thryothorus genibarbis*	Neotropics	1	0.0126	2.6209	3.58	0.6662	0.2206	52.27
Troglodytidae	*Thryothorus griseus*	Neotropics	0	-0.8211	4.9321	3.81	0.7356	0.1904	49.85
Troglodytidae	*Thryothorus guarayanus*	Neotropics	0	-0.1792	-	3.84	0.8531	0.6086	36.67
Troglodytidae	*Thryothorus leucopogon*	Neotropics	0	-0.5545	4.8986	3.12	0.7895	0.4470	67.61
Troglodytidae	*Thryothorus leucotis*	Neotropics	0	-29.3703	8.9159	3.97	0.6997	0.5783	78.85
Troglodytidae	*Thryothorus longirostris*	IndoMalay	0	-0.0954	-	3.78	0.7828	0.3007	7.00
Troglodytidae	*Thryothorus ludovicianus*	Palearctic	0	-0.9857	-	4.80	0.8533	0.4037	0.03
Troglodytidae	*Thryothorus maculipectus*	Palearctic	0	0.1645	12.8397	3.97	0.7493	0.1738	2.31
Troglodytidae	*Thryothorus modestus*	Palearctic	0	-0.3359	7.0196	3.72	0.7758	0.3369	4.43
Troglodytidae	*Thryothorus mystacalis*	IndoMalay	0	-0.7218	6.7349	2.61	0.6562	0.4622	64.29
Troglodytidae	*Thryothorus nicefori*	Afrotropics	1	1.3152	2.4816	3.23	0.6302	0.4924	53.13
Troglodytidae	*Thryothorus nigricapillus*	Afrotropics	0	-0.5056	8.2714	3.26	0.7300	0.3135	59.12
Troglodytidae	*Thryothorus pleurostictus*	Afrotropics	0	-0.1176	-	3.47	0.7371	0.5652	88.75
Troglodytidae	*Thryothorus rufalbus*	Afrotropics	0	0.0804	2.2131	3.04	0.6948	0.1214	55.17
Troglodytidae	*Thryothorus rutilus*	Afrotropics	0	0.2024	9.9377	3.20	0.7452	0.4281	70.53
Troglodytidae	*Thryothorus sclateri*	Afrotropics	0	0.0687	9.9850	3.39	0.7939	0.2881	17.50
Troglodytidae	*Thryothorus semibadius*	Afrotropics	0	0.1901	6.2224	3.53	0.7747	0.7064	88.48
Troglodytidae	*Thryothorus sinaloa*	Afrotropics	0	-0.1583	7.9579	3.34	0.7809	0.4073	79.68
Troglodytidae	*Thryothorus spadix*	Afrotropics	0	0.1845	5.6004	3.14	0.7889	0.2936	19.69
Troglodytidae	*Thryothorus superciliaris*	Afrotropics	0	0.0464	4.3906	3.28	0.8154	0.3151	15.98
Troglodytidae	*Thryothorus thoracicus*	Afrotropics	0	-0.2334	7.4394	3.48	0.8052	0.4411	79.86
Troglodytidae	*Troglodytes aedon*	Neotropics	0	0.0441	-	3.42	0.7517	0.2212	33.67
Troglodytidae	*Troglodytes cobbi*	Neotropics	0	0.0994	6.7361	3.41	0.7526	0.2152	48.10
Troglodytidae	*Troglodytes monticola*	Neotropics	1	40.8858	4.4175	3.84	0.6428	0.6028	65.97
Troglodytidae	*Troglodytes ochraceus*	Neotropics	0	0.0350	-	3.13	0.7526	0.5605	62.05
Troglodytidae	*Troglodytes rufociliatus*	Neotropics	0	0.5222	-	3.54	0.7399	0.1258	23.70
Troglodytidae	*Troglodytes rufulus*	Neotropics	0	-0.0363	-	3.77	0.7799	0.2922	16.78
Troglodytidae	*Troglodytes solstitialis*	Neotropics	0	-1.1946	-	3.61	0.7452	0.4160	3.23
Troglodytidae	*Troglodytes troglodytes*	Neotropics	0	-0.9649	4.0911	3.22	0.7087	0.3389	66.32
Troglodytidae	*Uropsila leucogastra*	Australasia	0	-0.0375	6.4049	2.56	0.6523	0.1875	30.24
Trogonidae	*Apalharpactes mackloti*	IndoMalay	0	0.0461	7.9003	2.77	0.7245	0.1030	53.82
Trogonidae	*Apalharpactes reinwardtii*	IndoMalay	1	0.0069	8.8220	2.75	0.6370	0.3167	20.59
Trogonidae	*Apaloderma aequatoriale*	Afrotropics	0	0.0722	-	3.22	0.7731	0.3751	2.14
Trogonidae	*Apaloderma narina*	Afrotropics	0	-0.0405	14.6527	3.44	0.8476	0.6870	35.47
Trogonidae	*Apaloderma vittatum*	Afrotropics	0	-0.0360	11.3384	3.33	0.7722	0.4635	28.59
Trogonidae	*Euptilotis neoxenus*	Afrotropics	0	-0.1112	-	3.49	0.8191	0.5897	78.17
Trogonidae	*Harpactes ardens*	IndoMalay	0	-0.4525	-	3.07	0.8235	0.8496	38.22
Trogonidae	*Harpactes diardii*	IndoMalay	0	-0.3667	-	2.71	0.7104	0.1572	11.52
Trogonidae	*Harpactes duvaucelii*	Afrotropics	0	-0.0439	9.6967	3.53	0.8572	0.6415	28.30
Trogonidae	*Harpactes erythrocephalus*	Afrotropics	0	-0.0292	7.2363	3.31	0.8272	0.3498	15.50
Trogonidae	*Harpactes fasciatus*	IndoMalay	0	-0.6786	-	3.41	0.8375	0.2812	18.10
Trogonidae	*Harpactes kasumba*	Afrotropics	0	0.0116	6.2036	3.52	0.8565	0.5834	33.65
Trogonidae	*Harpactes oreskios*	Afrotropics	0	-0.1238	-	3.08	0.7449	0.1239	68.40
Trogonidae	*Harpactes orrhophaeus*	IndoMalay	0	-0.2044	-	3.42	0.8376	0.3328	19.72
Trogonidae	*Harpactes wardi*	Palearctic	0	-0.7078	-	4.59	0.8187	0.1686	3.82
Trogonidae	*Harpactes whiteheadi*	Nearctic	0	-0.7183	5.8051	4.18	0.8478	0.2768	15.59
Trogonidae	*Pharomachrus antisianus*	IndoMalay	0	-0.2585	-	3.75	0.8286	0.4354	13.86
Trogonidae	*Pharomachrus auriceps*	Australasia	0	0.1175	-	3.35	0.7363	0.4140	13.03
Trogonidae	*Pharomachrus fulgidus*	Australasia	0	0.2015	-	3.21	0.6906	0.3997	20.61

Family	Species	Realm	Threat	Latitude	Elevation	Anomaly	Size	Shape	Orientation
Trogonidae	*Pharomachrus mocinno*	Nearctic	0	-0.8331	1.6885	3.86	0.7756	0.4941	9.06
Trogonidae	*Pharomachrus pavoninus*	Neotropics	0	-0.1857	8.8722	3.75	0.7852	0.3006	31.80
Trogonidae	*Priotelus roseigaster*	IndoMalay	0	-0.0623	6.9699	3.31	0.7853	0.8403	60.63
Trogonidae	*Priotelus temnurus*	IndoMalay	0	-0.1097	-	3.09	0.7861	0.6027	53.46
Trogonidae	*Trogon aurantiiventris*	Neotropics	0	-0.0197	-	3.01	0.6370	0.2651	32.26
Trogonidae	*Trogon bairdii*	Neotropics	0	-0.3431	-	3.79	0.7505	0.1760	49.93
Trogonidae	*Trogon caligatus*	Neotropics	0	-0.1062	6.8624	3.05	0.7019	0.3665	73.67
Trogonidae	*Trogon chionurus*	Neotropics	0	-0.4057	-	3.18	0.7128	0.2371	80.33
Trogonidae	*Trogon citreolus*	Neotropics	0	-0.1419	-	3.22	0.7087	0.4083	56.92
Trogonidae	*Trogon clathratus*	Neotropics	0	-0.2095	-	3.26	0.7403	0.6623	87.25
Trogonidae	*Trogon collaris*	Neotropics	0	-0.3314	-	2.53	0.6961	0.1557	17.00
Trogonidae	*Trogon comptus*	Neotropics	0	-0.2862	6.0901	3.27	0.7807	0.7011	62.18
Trogonidae	*Trogon curucui*	Neotropics	0	-0.0815	5.1831	3.56	0.7982	0.3035	59.04
Trogonidae	*Trogon elegans*	Neotropics	0	-0.1365	5.5014	3.25	0.7843	0.5086	33.26
Trogonidae	*Trogon massena*	Palearctic	0	-0.9374	-	4.27	0.8355	0.1733	5.45
Trogonidae	*Trogon melanocephalus*	IndoMalay	0	0.0753	9.4248	3.49	0.7661	0.3756	22.43
Trogonidae	*Trogon melanurus*	Afrotropics	0	0.0266	-	3.30	0.8208	0.2496	20.85
Trogonidae	*Trogon mesurus*	Neotropics	0	-0.2138	-	3.41	0.7996	0.2991	51.63
Trogonidae	*Trogon mexicanus*	Neotropics	0	-0.2361	-	3.78	0.8535	0.6458	45.31
Trogonidae	*Trogon personatus*	Neotropics	0	-0.0057	-	3.48	0.7848	0.2470	29.62
Trogonidae	*Trogon ramonianus*	Neotropics	0	-0.6985	5.7820	2.92	0.6842	0.2255	14.25
Trogonidae	*Trogon rufus*	Neotropics	1	-0.5220	3.7764	2.75	0.6302	0.4335	20.41
Trogonidae	*Trogon surrucura*	Neotropics	0	-0.0052	9.6280	3.65	0.7558	0.1629	23.71
Trogonidae	*Trogon violaceus*	IndoMalay	0	-0.0834	-	3.19	0.8119	0.6040	33.17
Trogonidae	*Trogon viridis*	Australasia	0	-0.0897	-	2.81	0.7452	0.1721	24.28
Turdidae	*Alethe choloensis*	Australasia	0	-0.4024	-	3.21	0.7692	0.2093	59.70
Turdidae	*Alethe diademata*	Afrotropics	1	-0.7457	5.5650	3.57	0.6631	0.7721	83.32
Turdidae	*Alethe fuelleborni*	Afrotropics	0	0.0103	6.5054	3.27	0.8187	0.2885	14.19
Turdidae	*Alethe poliocephala*	Afrotropics	0	-0.0568	-	3.33	0.7392	0.2991	78.33
Turdidae	*Alethe poliophrys*	Afrotropics	0	0.1158	-	3.21	0.8009	0.2246	10.74
Turdidae	*Brachypteryx hyperythra*	Neotropics	0	-0.1922	-	4.08	0.7240	0.4072	31.32
Turdidae	*Brachypteryx leucophrys*	Neotropics	0	0.0158	-	3.99	0.8277	0.5948	37.48
Turdidae	*Brachypteryx montana*	Neotropics	0	-0.6824	-	3.13	0.6948	0.7477	19.35
Turdidae	*Brachypteryx stellata*	Afrotropics	1	-0.1483	4.9142	3.02	0.7144	0.1282	74.30
Turdidae	*Cataponera turdoides*	Neotropics	1	-0.1585	1.3458	2.92	0.7261	0.2563	48.39
Turdidae	*Catharus aurantiirostris*	Neotropics	0	-0.3677	5.4241	3.77	0.8120	0.6573	14.29
Turdidae	*Catharus bicknelli*	Afrotropics	0	-0.4626	4.6727	3.67	0.8968	0.3133	1.06
Turdidae	*Catharus dryas*	Australasia	0	-0.1287	11.0610	2.83	0.7512	0.2407	26.30
Turdidae	*Catharus frantzii*	Australasia	1	-0.1031	0.8927	2.78	0.7602	0.3846	32.18
Turdidae	*Catharus fuscater*	Australasia	1	0.0151	2.2121	2.70	0.7352	0.1736	12.66
Turdidae	*Catharus fuscescens*	Neotropics	0	-0.1185	7.1918	3.66	0.7697	0.4270	77.70
Turdidae	*Catharus gracilirostris*	Neotropics	0	-0.2202	10.5363	3.62	0.7911	0.2422	73.80
Turdidae	*Catharus guttatus*	Neotropics	0	0.0251	10.6261	3.77	0.7640	0.6056	83.36
Turdidae	*Catharus mexicanus*	Neotropics	0	-0.2322	6.0809	3.77	0.7858	0.2790	72.59
Turdidae	*Catharus minimus*	Australasia	0	-0.1447	7.3483	2.76	0.6948	0.6992	64.99
Turdidae	*Catharus occidentalis*	Neotropics	0	-0.9471	-	1.75	0.6302	0.0928	52.35
Turdidae	*Catharus ustulatus*	Neotropics	0	-0.4292	-	1.84	0.7469	0.4479	75.05
Turdidae	*Chlamydochaera jefferyi*	Nearctic	0	-0.6668	6.5090	5.60	0.7474	0.3979	18.36
Turdidae	*Cichlopsis leucogenys*	Neotropics	0	-0.1059	5.4210	3.87	0.8383	0.4402	41.74
Turdidae	*Cochoa azurea*	Afrotropics	0	0.2909	-	3.31	0.7300	0.2392	16.11
Turdidae	*Cochoa beccarii*	Neotropics	0	-0.5338	2.4123	3.01	0.8053	0.6850	45.81
Turdidae	*Cochoa purpurea*	Neotropics	0	-0.0418	2.6832	4.00	0.8428	0.7035	36.79
Turdidae	*Cochoa viridis*	Neotropics	0	-0.0822	2.1006	3.94	0.7721	0.6087	12.82
Turdidae	*Entomodestes coracinus*	Nearctic	0	-0.5165	-	4.34	0.8309	0.1762	9.88
Turdidae	*Entomodestes leucotis*	Nearctic	0	-0.2636	15.3417	3.96	0.7762	0.2253	42.04
Turdidae	*Geomalia heinrichi*	Palearctic	0	-0.7139	12.6996	3.75	0.7911	0.2974	6.17
Turdidae	*Heinrichia calligyna*	IndoMalay	0	-0.2534	7.0594	3.17	0.8430	0.3350	31.18
Turdidae	*Hylocichla mustelina*	Afrotropics	0	0.2857	-	3.44	0.8069	-	-
Turdidae	*Hylocichla mustelina*	Afrotropics	0	0.2857	-	3.44	0.8069	0.3318	11.80
Turdidae	*Myadestes coloratus*	Afrotropics	0	-0.6239	7.0537	3.39	0.7574	0.3649	38.18
Turdidae	*Myadestes elisabeth*	Palearctic	0	-0.9797	-	4.36	0.8052	0.6151	17.08
Turdidae	*Myadestes genibarbis*	Afrotropics	0	-0.2277	-	3.12	0.6690	0.4189	44.45
Turdidae	*Myadestes melanops*	Palearctic	0	-0.5784	11.0335	3.50	0.8096	0.2597	3.27
Turdidae	*Myadestes obscurus*	Afrotropics	0	0.0715	3.5729	3.50	0.7777	0.4213	63.93
Turdidae	*Myadestes occidentalis*	Afrotropics	0	-0.6270	-	3.38	0.7653	0.4215	43.56
Turdidae	*Myadestes ralloides*	Palearctic	0	-1.1069	14.3557	4.18	0.8466	0.1494	2.79
Turdidae	*Myadestes townsendi*	Afrotropics	0	-0.2216	6.7916	3.18	0.7490	0.3198	66.95
Turdidae	*Myadestes unicolor*	Palearctic	0	-0.4465	10.9771	3.79	0.8485	0.2113	3.45
Turdidae	*Myophonus borneensis*	Neotropics	0	-0.5293	2.5821	2.41	0.5788	0.0000	0.00
Turdidae	*Myophonus caeruleus*	Neotropics	0	0.1303	9.9388	3.29	0.6997	0.2474	49.93
Turdidae	*Myophonus castaneus*	Neotropics	0	-0.1258	7.4537	2.47	0.5985	0.0000	0.00
Turdidae	*Myophonus glaucinus*	Neotropics	0	-0.0059	11.0186	3.71	0.7650	0.4362	84.18
Turdidae	*Myophonus horsfieldii*	Nearctic	0	-0.6810	9.8196	4.10	0.8180	0.4587	39.74
Turdidae	*Myophonus insularis*	Nearctic	0	-0.7729	-	4.28	0.8297	0.6701	5.51

Family	Species	Realm	Threat	Latitude	Elevation	Anomaly	Size	Shape	Orientation
Turdidae	*Myophonus melanurus*	Neotropics	0	-0.1942	-	3.88	0.8504	0.6560	33.77
Turdidae	*Neocossyphus poensis*	Afrotropics	0	-0.1520	-	3.67	0.7618	0.3782	2.47
Turdidae	*Neocossyphus rufus*	Afrotropics	0	0.0391	-	3.21	0.7827	0.3087	19.35
Turdidae	*Psophocichla litsitsirupa*	Afrotropics	0	-0.2557	-	3.43	0.7296	0.2903	76.75
Turdidae	*Sialia currucoides*	Australasia	0	-0.0631	-	2.82	0.7382	0.1462	22.87
Turdidae	*Sialia mexicana*	Palearctic	0	-0.5311	-	4.68	0.8592	0.1764	0.24
Turdidae	*Sialia sialis*	IndoMalay	0	0.0412	8.7587	2.76	0.6997	0.0902	51.31
Turdidae	*Stizorhina fraseri*	Neotropics	0	-0.1710	4.6371	3.35	0.6859	0.1570	67.41
Turdidae	*Turdus albicollis*	IndoMalay	1	0.0011	2.7326	2.62	0.7096	0.6073	74.68
Turdidae	*Turdus albocinctus*	Neotropics	0	0.1236	-	4.01	0.8234	0.5786	0.50
Turdidae	*Turdus amaurochalinus*	Neotropics	0	-0.1469	-	3.73	0.8425	0.4785	36.37
Turdidae	*Turdus assimilis*	Neotropics	0	-0.0268	-	4.01	0.8244	0.5182	5.41
Turdidae	*Turdus aurantius*	Neotropics	0	0.1146	-	3.34	0.7613	0.8106	44.84
Turdidae	*Turdus boulboul*	Neotropics	0	-0.0597	-	4.08	0.7968	0.5664	14.26
Turdidae	*Turdus cardis*	Neotropics	0	-0.3320	-	3.18	0.7597	0.3694	40.91
Turdidae	*Turdus chiguanco*	Neotropics	0	-0.0294	-	3.33	0.6523	0.2546	10.40
Turdidae	*Turdus chrysolaus*	Neotropics	0	0.0342	11.0389	2.46	0.6876	0.3855	3.42
Turdidae	*Turdus dissimilis*	Neotropics	0	-0.1643	-	2.41	0.5788	0.0000	0.00
Turdidae	*Turdus falcklandii*	Neotropics	0	-0.3372	-	2.53	0.6974	0.1632	17.43
Turdidae	*Turdus feae*	Neotropics	0	0.0396	-	2.46	0.6876	0.3855	3.42
Turdidae	*Turdus flavipes*	Neotropics	0	-0.2241	-	2.47	0.5985	0.0000	0.00
Turdidae	*Turdus fulviventris*	Neotropics	0	0.0227	2.9941	3.98	0.8333	0.7624	6.43
Turdidae	*Turdus fumigatus*	Neotropics	0	-0.1175	3.2838	3.95	0.8436	0.5504	20.67
Turdidae	*Turdus fuscater*	Neotropics	0	-0.1089	-	4.00	0.8333	0.6357	3.43
Turdidae	*Turdus grayi*	Neotropics	0	-0.1712	-	3.82	0.8524	0.4948	40.49
Turdidae	*Turdus haplochrous*	Neotropics	0	0.2536	1.8881	3.74	0.7449	0.8842	33.55
Turdidae	*Turdus hauxwelli*	Neotropics	0	-0.1139	-	4.00	0.7963	0.5067	28.59
Turdidae	*Turdus helleri*	Neotropics	0	0.7230	-	3.77	0.7764	0.4014	1.30
Turdidae	*Turdus hortulorum*	Afrotropics	1	-0.1457	14.7787	3.77	0.8464	0.8523	87.68
Turdidae	*Turdus ignobilis*	Neotropics	1	0.0465	-	2.54	0.5788	0.0000	0.00
Turdidae	*Turdus iliacus*	Neotropics	0	0.0230	3.3948	3.80	0.7485	0.2371	26.34
Turdidae	*Turdus infuscatus*	Neotropics	1	-0.2025	11.6889	3.02	0.6690	0.2193	30.39
Turdidae	*Turdus jamaicensis*	Neotropics	0	0.2181	4.9536	3.25	0.7318	0.2942	54.88
Turdidae	*Turdus kessleri*	Neotropics	0	-0.0011	-	3.97	0.8021	-	-
Turdidae	*Turdus kessleri*	Neotropics	0	-0.0011	-	3.97	0.8021	0.6260	2.40
Turdidae	*Turdus lawrencii*	Neotropics	1	-0.2504	4.3348	2.81	0.6948	0.3191	39.69
Turdidae	*Turdus leucomelas*	Neotropics	0	-0.0391	3.1281	3.98	0.8201	0.4936	3.34
Turdidae	*Turdus leucops*	Neotropics	1	2.3229	8.4163	3.39	0.7261	0.2260	66.38
Turdidae	*Turdus libonyanus*	Neotropics	1	-0.1827	3.0598	2.92	0.7217	0.1819	53.50
Turdidae	*Turdus ludoviciae*	Australasia	0	-0.0843	-	2.80	0.7661	0.2558	24.76
Turdidae	*Turdus maculirostris*	Australasia	0	-0.1125	-	2.87	0.7422	0.3868	18.19
Turdidae	*Turdus maranonicus*	Australasia	0	-0.2799	-	2.83	0.7466	0.1956	23.74
Turdidae	*Turdus menachensis*	Nearctic	1	-0.4547	7.2285	4.26	0.7771	0.7984	42.98
Turdidae	*Turdus merula*	Nearctic	0	-0.7825	-	2.96	0.7019	0.1199	52.44
Turdidae	*Turdus migratorius*	Nearctic	0	-0.5032	-	4.15	0.7839	0.3359	37.69
Turdidae	*Turdus mupinensis*	Nearctic	0	-0.6158	-	4.05	0.8028	0.6461	58.23
Turdidae	*Turdus naumanni*	Nearctic	0	-0.0338	4.3406	3.88	0.7488	0.5163	56.16
Turdidae	*Turdus nigrescens*	Nearctic	0	-0.4260	-	3.73	0.7455	0.3797	76.78
Turdidae	*Turdus nigriceps*	Nearctic	0	-0.4667	-	3.34	0.7348	0.2152	52.04
Turdidae	*Turdus nudigenis*	Nearctic	0	-0.7057	-	4.29	0.8317	0.5082	18.78
Turdidae	*Turdus obscurus*	Afrotropics	0	0.0378	7.6630	3.28	0.7967	0.6914	56.14
Turdidae	*Turdus obsoletus*	Afrotropics	0	0.1301	8.1681	3.35	0.7971	0.4786	52.82
Turdidae	*Turdus olivaceus*	Afrotropics	0	0.0109	8.3203	3.74	0.8078	0.1325	3.36
Turdidae	*Turdus olivater*	Afrotropics	0	0.0529	11.2584	3.28	0.8234	0.3560	12.57
Turdidae	*Turdus pallidus*	Afrotropics	0	12.3266	3.0929	3.23	0.7286	0.7200	41.36
Turdidae	*Turdus pelios*	Afrotropics	0	-0.2138	8.8110	3.69	0.8170	0.9374	49.45
Turdidae	*Turdus philomelos*	IndoMalay	1	-0.0906	9.9632	3.49	0.7327	0.7253	52.63
Turdidae	*Turdus pilaris*	IndoMalay	1	-0.6817	6.2145	3.25	0.7567	0.3360	22.08
Turdidae	*Turdus plebejus*	IndoMalay	1	-9.3417	10.3299	4.44	0.6921	0.2804	38.34
Turdidae	*Turdus plumbeus*	IndoMalay	0	0.0916	11.3782	4.10	0.7277	0.1183	14.72
Turdidae	*Turdus poliocephalus*	Palearctic	0	-0.4992	-	3.51	0.7899	0.6435	1.56
Turdidae	*Turdus reevei*	Australasia	0	-0.4575	-	3.11	0.7240	0.1562	61.96
Turdidae	*Turdus rubrocanus*	Australasia	0	-0.0512	-	2.79	0.7482	0.1769	24.72
Turdidae	*Turdus ruficollis*	IndoMalay	0	-0.0544	-	3.29	0.7993	0.4369	22.96
Turdidae	*Turdus rufitorques*	Afrotropics	0	-0.0792	3.4153	3.18	0.7614	0.3874	70.15
Turdidae	*Turdus rufiventris*	IndoMalay	0	-0.1401	3.9587	3.11	0.8106	0.6184	26.63
Turdidae	*Turdus rufopalliatus*	Afrotropics	0	-0.1494	-	3.46	0.8500	0.4536	31.08
Turdidae	*Turdus serranus*	IndoMalay	1	-0.0022	2.7697	2.84	0.7874	0.4741	4.78
Turdidae	*Turdus swalesi*	IndoMalay	0	-0.0552	-	3.04	0.8169	0.4237	59.80
Turdidae	*Turdus tephronotus*	Australasia	1	0.6873	3.5106	2.63	0.6523	0.0000	0.00
Turdidae	*Turdus torquatus*	IndoMalay	0	-0.8249	5.3855	2.61	0.6562	0.4622	64.29
Turdidae	*Turdus unicolor*	IndoMalay	0	0.0071	0.3865	2.83	0.7841	0.4780	6.49
Turdidae	*Turdus viscivorus*	Australasia	0	-0.0413	6.7530	2.71	0.7385	0.2363	24.13
Turdidae	*Zoothera andromedae*	Neotropics	0	0.2143	4.9091	3.99	0.8300	0.5259	10.78

Family	Species	Realm	Threat	Latitude	Elevation	Anomaly	Size	Shape	Orientation
Turdidae	*Zoothera camaronensis*	Neotropics	0	-0.0891	-	3.18	0.7234	0.2881	89.15
Turdidae	*Zoothera cinerea*	Neotropics	0	-0.0588	-	4.04	0.7874	0.4971	32.54
Turdidae	*Zoothera citrina*	Neotropics	0	-0.0616	2.3264	2.96	0.7096	0.2300	70.21
Turdidae	*Zoothera crossleyi*	Neotropics	0	0.0560	8.2587	3.24	0.7340	0.2550	83.39
Turdidae	*Zoothera dauma*	Neotropics	0	1.9751	-	3.83	0.7217	0.2270	87.18
Turdidae	*Zoothera dixoni*	Neotropics	0	0.0852	-	3.52	0.7751	0.4799	29.29
Turdidae	*Zoothera dohertyi*	Neotropics	0	-0.6690	12.7452	2.86	0.7695	0.1300	76.67
Turdidae	*Zoothera dumasi*	Neotropics	0	-0.1075	-	3.13	0.7389	0.3784	57.23
Turdidae	*Zoothera erythronota*	Neotropics	0	-0.4179	2.0508	3.41	0.8200	0.3169	52.82
Turdidae	*Zoothera everetti*	Neotropics	0	0.7177	11.3450	3.68	0.7500	0.3057	60.49
Turdidae	*Zoothera gurneyi*	Neotropics	0	-0.1838	-	3.92	0.8490	0.6519	28.21
Turdidae	*Zoothera guttata*	Neotropics	0	0.2544	-	3.43	0.6876	0.1588	20.20
Turdidae	*Zoothera heinei*	Neotropics	0	-0.4682	-	3.08	0.7939	0.3762	54.00
Turdidae	*Zoothera imbricata*	Nearctic	1	-0.7785	-	3.95	0.7782	0.3594	10.75
Turdidae	*Zoothera interpres*	Nearctic	0	-0.8727	-	4.16	0.8403	0.3362	10.01
Turdidae	*Zoothera joiceyi*	Nearctic	0	-0.8517	7.8732	4.30	0.7955	0.5790	11.47
Turdidae	*Zoothera lunulata*	Nearctic	0	14.3085	9.1836	3.97	0.7165	0.2942	68.84
Turdidae	*Zoothera machiki*	Nearctic	0	-0.8486	0.0000	4.32	0.8001	0.4092	1.07
Turdidae	*Zoothera margaretae*	Nearctic	0	-0.4776	-	4.25	0.7595	0.6310	28.87
Turdidae	*Zoothera marginata*	Nearctic	0	-0.5728	-	4.27	0.8281	0.1467	10.45
Turdidae	*Zoothera mendeni*	Nearctic	0	-0.7707	-	4.30	0.8155	0.2148	1.03
Turdidae	*Zoothera mollissima*	Nearctic	0	-0.6365	-	4.48	0.7739	0.6603	20.64
Turdidae	*Zoothera naevia*	Afrotropics	0	0.0715	0.0000	3.36	0.7782	0.2136	10.06
Turdidae	*Zoothera oberlaenderi*	Afrotropics	0	-0.0781	-	3.58	0.8413	0.6048	33.41
Turdidae	*Zoothera peronii*	Afrotropics	0	-0.4399	-	3.70	0.7490	0.6093	56.22
Turdidae	*Zoothera piaggiae*	Afrotropics	0	0.0891	-	3.33	0.7955	0.4920	49.08
Turdidae	*Zoothera pinicola*	Afrotropics	0	-0.2727	0.0000	3.51	0.7994	0.6923	86.66
Turdidae	*Zoothera princei*	Afrotropics	0	0.0705	-	3.33	0.7848	0.4316	64.32
Turdidae	*Zoothera schistacea*	Afrotropics	0	0.0265	-	3.45	0.8005	0.1264	7.39
Turdidae	*Zoothera sibirica*	Afrotropics	0	0.4726	-	3.44	0.7722	0.2110	0.73
Turdidae	*Zoothera spiloptera*	Afrotropics	0	-0.1340	-	3.50	0.8555	0.6499	38.76
Turdidae	*Zoothera talaseae*	Afrotropics	0	0.0000	2.3855	3.40	0.6598	0.7578	59.35
Turdidae	*Zoothera tanganjicae*	Afrotropics	0	0.0822	0.0000	3.43	0.7179	0.0826	11.06
Turdidae	*Zoothera turipavae*	Afrotropics	0	-0.0117	-	3.62	0.8179	0.6530	2.52
Turdidae	*Zoothera wardii*	Afrotropics	0	-0.0676	5.6875	3.61	0.8406	0.7173	45.19
Turnicidae	*Ortyxelos meiffrenii*	Australasia	0	-0.0443	10.1442	2.81	0.7374	0.1517	23.88
Turnicidae	*Turnix castanotus*	IndoMalay	0	0.0076	-	2.83	0.7887	0.4876	3.14
Turnicidae	*Turnix everetti*	IndoMalay	0	-0.0305	12.7676	2.76	0.7256	0.1090	51.70
Turnicidae	*Turnix hottentottus*	IndoMalay	0	-0.0740	-	3.38	0.8268	0.4428	13.44
Turnicidae	*Turnix maculosus*	IndoMalay	0	-0.0825	4.1259	3.09	0.8071	0.4378	11.17
Turnicidae	*Turnix melanogaster*	Australasia	1	-0.1464	2.4276	2.56	0.6523	0.1875	30.24
Turnicidae	*Turnix nigricollis*	IndoMalay	0	-0.1303	-	2.91	0.7441	0.6228	85.12
Turnicidae	*Turnix ocellatus*	IndoMalay	0	-0.6337	6.3293	3.22	0.8035	0.2623	26.72
Turnicidae	*Turnix olivii*	IndoMalay	0	-0.0736	10.3017	3.29	0.8061	0.5315	52.71
Turnicidae	*Turnix pyrrhothorax*	IndoMalay	0	-0.0150	-	2.82	0.8052	0.7987	13.79
Turnicidae	*Turnix suscitator*	Afrotropics	0	0.0454	9.0998	3.53	0.8322	0.1754	1.78
Turnicidae	*Turnix sylvaticus*	IndoMalay	0	-0.0068	1.0658	2.82	0.7773	0.4917	10.99
Turnicidae	*Turnix tanki*	IndoMalay	0	0.0826	1.3520	2.85	0.7519	0.2186	49.02
Turnicidae	*Turnix varius*	Australasia	0	-0.1626	5.9475	2.71	0.7185	0.5623	60.71
Turnicidae	*Turnix velox*	IndoMalay	0	0.0015	5.1090	2.87	0.7474	0.3782	55.06
Turnicidae	*Turnix worcesteri*	IndoMalay	0	0.0215	0.7488	2.86	0.7606	0.4869	18.93
Tyrannidae	*Agriornis albicauda*	Neotropics	0	0.0031	9.3989	3.70	0.7683	0.4405	89.45
Tyrannidae	*Agriornis lividus*	Neotropics	1	-0.0164	11.2711	3.97	0.7802	0.2096	54.48
Tyrannidae	*Agriornis micropterus*	Neotropics	0	-0.5510	6.0009	2.25	0.7666	0.1631	87.26
Tyrannidae	*Agriornis montanus*	Neotropics	0	-0.6552	6.3861	2.41	0.7616	0.4606	71.12
Tyrannidae	*Agriornis murinus*	Neotropics	0	-0.2175	14.1272	3.31	0.8071	0.2476	80.13
Tyrannidae	*Alectrurus risora*	Afrotropics	0	-0.0921	8.1098	3.17	0.7485	0.3092	71.28
Tyrannidae	*Alectrurus tricolor*	Neotropics	1	-0.5522	0.8380	2.71	0.7812	0.5371	68.89
Tyrannidae	*Anairetes agilis*	Neotropics	0	8.8370	9.1913	3.35	0.7087	0.1277	55.77
Tyrannidae	*Anairetes agraphia*	Neotropics	0	-7.7114	5.5022	3.93	0.7192	0.1763	52.28
Tyrannidae	*Anairetes alpinus*	Neotropics	1	0.9862	5.0393	3.97	0.7136	0.1380	35.36
Tyrannidae	*Anairetes flavirostris*	Neotropics	0	-0.2545	13.1935	3.39	0.7969	0.2743	78.61
Tyrannidae	*Anairetes nigrocristatus*	Neotropics	0	-0.4842	12.2709	3.60	0.6997	0.1908	57.56
Tyrannidae	*Anairetes parulus*	Neotropics	0	-0.2983	12.0995	2.96	0.8111	0.2704	84.87
Tyrannidae	*Anairetes reguloides*	Neotropics	0	-0.0633	13.8937	3.82	0.7165	0.1643	49.54
Tyrannidae	*Aphanotriccus audax*	Neotropics	0	-0.2380	1.2182	3.37	0.7286	0.8372	5.22
Tyrannidae	*Aphanotriccus capitalis*	Neotropics	1	-0.2692	2.0157	3.19	0.6662	0.6939	39.72
Tyrannidae	*Arundinicola leucocephala*	Australasia	0	-0.5715	-	3.34	0.8376	0.5670	8.35
Tyrannidae	*Atalotriccus pilaris*	Australasia	0	0.3802	3.3777	2.49	0.6302	0.2885	29.23
Tyrannidae	*Attila bolivianus*	Neotropics	0	0.0018	11.4754	3.62	0.7211	0.3229	20.86
Tyrannidae	*Attila cinnamomeus*	Neotropics	0	-0.1605	8.8028	3.76	0.7512	0.3686	37.35
Tyrannidae	*Attila citriniventris*	Neotropics	0	0.0157	-	4.04	0.8347	0.7597	4.13
Tyrannidae	*Attila phoenicurus*	Neotropics	0	-0.1297	-	4.08	0.8060	0.7011	60.50
Tyrannidae	*Attila rufus*	Neotropics	0	0.0128	-	4.12	0.8124	0.6752	11.71

Family	Species	Realm	Threat	Latitude	Elevation	Anomaly	Size	Shape	Orientation
Tyrannidae	*Attila spadiceus*	Neotropics	0	-0.1324	-	4.02	0.8291	0.7027	6.35
Tyrannidae	*Attila torridus*	Neotropics	0	0.5846	-	3.91	0.7898	0.5651	28.72
Tyrannidae	*Camptostoma imberbe*	Neotropics	0	-0.0142	-	4.04	0.8364	0.7753	0.87
Tyrannidae	*Camptostoma obsoletum*	Afrotropics	0	-0.1012	9.1399	3.75	0.8388	0.4860	40.97
Tyrannidae	*Capsiempis flaveola*	Neotropics	0	-0.1453	10.1445	3.84	0.7706	0.2418	40.77
Tyrannidae	*Casiornis fuscus*	Palearctic	0	-0.7395	14.2040	4.39	0.8197	0.3845	12.44
Tyrannidae	*Casiornis rufus*	Palearctic	0	1.1979	6.6513	4.29	0.8001	0.4055	6.09
Tyrannidae	*Cnemarchus erythropygius*	Neotropics	0	-0.1494	4.7400	3.84	0.8533	0.4841	36.04
Tyrannidae	*Cnemotriccus fuscatus*	Australasia	0	-0.0273	-	3.49	0.7862	0.3570	15.11
Tyrannidae	*Cnipodectes subbrunneus*	Australasia	0	-0.5542	-	3.27	0.8053	0.6098	90.00
Tyrannidae	*Cnipodectes superrufus*	Australasia	0	-0.8461	-	2.78	0.7611	0.2266	0.42
Tyrannidae	*Colonia colonus*	Australasia	0	-0.1124	-	2.90	0.7515	0.3654	20.13
Tyrannidae	*Colorhamphus parvirostris*	Australasia	0	-0.0724	-	2.49	0.6302	0.0405	36.17
Tyrannidae	*Conopias albovittatus*	Neotropics	0	-0.3700	-	3.39	0.8310	0.5324	53.48
Tyrannidae	*Conopias cinchoneti*	Neotropics	0	-0.1872	-	3.73	0.8268	0.5936	49.00
Tyrannidae	*Conopias parvus*	Neotropics	0	-0.1948	-	3.75	0.8562	0.4959	41.04
Tyrannidae	*Conopias trivirgatus*	Neotropics	1	11.0128	6.9300	3.77	0.6948	0.1607	75.53
Tyrannidae	*Contopus albogularis*	Neotropics	0	0.1761	7.0852	3.66	0.7452	0.3139	85.19
Tyrannidae	*Contopus caribaeus*	Neotropics	0	-0.0120	2.9373	4.01	0.8152	0.4669	20.06
Tyrannidae	*Contopus cinereus*	Neotropics	0	-0.2241	2.3877	3.67	0.7983	0.4672	46.71
Tyrannidae	*Contopus cooperi*	Neotropics	0	-3.8876	8.8470	4.01	0.7251	0.2147	46.75
Tyrannidae	*Contopus fumigatus*	Neotropics	0	-0.0295	-	4.00	0.8162	0.5216	9.74
Tyrannidae	*Contopus hispaniolensis*	Neotropics	0	0.0349	8.0897	3.52	0.7482	0.2697	88.76
Tyrannidae	*Contopus lugubris*	Neotropics	0	-0.2260	10.5146	3.38	0.8005	0.3918	44.21
Tyrannidae	*Contopus nigrescens*	Neotropics	0	0.0866	-	4.22	0.7809	0.3021	37.72
Tyrannidae	*Contopus ochraceus*	Neotropics	0	-0.1947	2.5337	2.96	0.7422	0.1853	55.25
Tyrannidae	*Contopus pallidus*	Neotropics	0	0.0808	-	4.03	0.7856	0.7166	32.67
Tyrannidae	*Contopus pertinax*	Neotropics	0	0.1685	-	3.56	0.7700	0.7332	7.54
Tyrannidae	*Contopus sordidulus*	Australasia	0	-0.0121	0.9975	2.96	0.7659	0.7153	17.59
Tyrannidae	*Contopus virens*	Australasia	0	-0.4215	-	3.44	0.8012	0.3449	10.39
Tyrannidae	*Corythopis delalandi*	Palearctic	0	-0.3993	13.7732	3.99	0.8570	0.3877	4.90
Tyrannidae	*Corythopis torquatus*	Neotropics	0	-0.1328	-	3.77	0.7136	0.2059	54.27
Tyrannidae	*Culicivora caudacuta*	Neotropics	0	-0.1896	2.7542	3.98	0.8424	0.6808	31.96
Tyrannidae	*Deltarhynchus flammulatus*	Neotropics	0	0.3086	-	4.08	0.8230	0.6644	21.11
Tyrannidae	*Elaenia albiceps*	Neotropics	0	-0.1713	-	2.46	0.6876	0.3855	3.42
Tyrannidae	*Elaenia chiriquensis*	Nearctic	0	-0.6184	-	4.27	0.8370	0.4057	8.96
Tyrannidae	*Elaenia cristata*	IndoMalay	0	-0.0931	-	3.35	0.8044	0.6881	78.97
Tyrannidae	*Elaenia dayi*	Neotropics	0	-0.0843	7.1988	3.48	0.7172	0.1652	47.06
Tyrannidae	*Elaenia fallax*	Neotropics	0	-0.1908	9.4656	3.66	0.8286	0.4728	40.42
Tyrannidae	*Elaenia flavogaster*	Neotropics	1	-0.1859	7.0057	3.16	0.6824	0.2110	81.37
Tyrannidae	*Elaenia frantzii*	Neotropics	1	-0.0117	3.0893	3.10	0.7096	0.3247	70.51
Tyrannidae	*Elaenia gigas*	Neotropics	0	0.0338	-	2.96	0.7251	0.5454	85.93
Tyrannidae	*Elaenia martinica*	Neotropics	0	-0.2757	4.9301	2.99	0.7591	0.2135	35.84
Tyrannidae	*Elaenia mesoleuca*	Neotropics	0	0.0899	-	3.29	0.7104	0.3295	79.43
Tyrannidae	*Elaenia obscura*	Neotropics	0	-0.4521	5.3369	2.89	0.7120	0.1615	26.08
Tyrannidae	*Elaenia pallatangae*	Australasia	0	-0.1090	-	2.74	0.7762	0.3334	13.13
Tyrannidae	*Elaenia pelzelni*	Afrotropics	0	-0.0902	6.1185	3.55	0.8435	0.6261	36.83
Tyrannidae	*Elaenia ruficeps*	Neotropics	0	-0.3650	6.7909	3.74	0.8338	0.4269	48.56
Tyrannidae	*Elaenia spectabilis*	Afrotropics	0	-0.4124	6.0287	3.50	0.8821	0.4422	8.72
Tyrannidae	*Elaenia strepera*	Afrotropics	0	-0.0920	0.8564	3.43	0.8070	0.3465	6.21
Tyrannidae	*Empidonax affinis*	Palearctic	0	-0.9540	-	4.02	0.8073	0.3618	14.90
Tyrannidae	*Empidonax albigularis*	Palearctic	0	-0.9622	-	5.21	0.8335	0.2151	4.46
Tyrannidae	*Empidonax alnorum*	Afrotropics	0	0.1820	-	3.34	0.7908	0.5782	44.39
Tyrannidae	*Empidonax atriceps*	Palearctic	0	-0.7771	-	5.52	0.8428	0.1104	0.61
Tyrannidae	*Empidonax difficilis*	Palearctic	0	-0.6906	-	5.25	0.8499	0.1175	0.20
Tyrannidae	*Empidonax flavescens*	Palearctic	0	-0.8918	-	4.77	0.8258	0.3944	2.55
Tyrannidae	*Empidonax flaviventris*	Palearctic	0	-0.8453	-	4.75	0.8693	0.2126	1.66
Tyrannidae	*Empidonax hammondii*	Palearctic	0	-0.8771	-	4.45	0.8290	0.5935	10.79
Tyrannidae	*Empidonax minimus*	Palearctic	0	-1.0235	7.3948	4.38	0.7899	0.6348	47.90
Tyrannidae	*Empidonax oberholseri*	Palearctic	0	-0.3191	9.8282	4.00	0.8268	0.2653	0.57
Tyrannidae	*Empidonax occidentalis*	Palearctic	1	-0.8489	4.4059	3.28	0.7104	0.3458	54.30
Tyrannidae	*Empidonax traillii*	Afrotropics	0	-0.0892	11.7440	3.62	0.8436	0.7051	44.05
Tyrannidae	*Empidonax virescens*	Palearctic	0	-0.8013	-	4.34	0.7599	0.7053	18.72
Tyrannidae	*Empidonax wrightii*	Palearctic	0	-0.7080	-	3.93	0.7432	0.3107	42.84
Tyrannidae	*Empidonomus aurantioatrocristatus*	Palearctic	0	-1.0500	-	4.08	0.7158	0.3019	28.25
Tyrannidae	*Empidonomus varius*	Neotropics	0	2.0690	4.6587	4.08	0.5788	0.0000	90.00
Tyrannidae	*Euscarthmus meloryphus*	Afrotropics	0	-0.4477	-	3.70	0.7712	0.5415	39.26
Tyrannidae	*Euscarthmus rufomarginatus*	Nearctic	0	-0.3638	7.3777	3.99	0.7526	0.2417	55.92
Tyrannidae	*Fluvicola albiventer*	Australasia	0	0.5012	3.5281	2.60	0.6120	0.4082	18.43
Tyrannidae	*Fluvicola albiventer*	Australasia	0	0.5012	3.5281	2.60	0.6120	-	-
Tyrannidae	*Fluvicola nengeta*	Palearctic	0	-0.4988	-	3.76	0.7985	0.7229	8.19
Tyrannidae	*Fluvicola pica*	IndoMalay	0	-0.1650	10.0391	3.14	0.8149	0.5434	54.19
Tyrannidae	*Gubernetes yetapa*	Neotropics	0	0.0486	10.3216	3.65	0.7616	0.4444	75.85

Family	Species	Realm	Threat	Latitude	Elevation	Anomaly	Size	Shape	Orientation
Tyrannidae	*Hemitriccus cinnamomeipectus*	Neotropics	0	-0.1418	-	3.91	0.8438	0.5194	41.91
Tyrannidae	*Hemitriccus diops*	Neotropics	0	-0.1753	2.9000	3.96	0.8365	0.6842	19.38
Tyrannidae	*Hemitriccus flammulatus*	Neotropics	0	0.0561	3.1315	3.24	0.7677	0.3823	49.61
Tyrannidae	*Hemitriccus furcatus*	Neotropics	0	0.2464	9.3110	3.52	0.7469	0.2788	74.01
Tyrannidae	*Hemitriccus granadensis*	Nearctic	0	-0.8408	4.3631	4.08	0.7984	0.4335	16.59
Tyrannidae	*Hemitriccus griseipectus*	IndoMalay	0	-0.1473	-	3.09	0.7818	0.3040	4.64
Tyrannidae	*Hemitriccus inornatus*	IndoMalay	0	-0.0297	-	2.83	0.7906	0.5955	11.41
Tyrannidae	*Hemitriccus minimus*	Australasia	0	-0.4657	3.7708	2.34	0.7318	0.2591	44.31
Tyrannidae	*Hemitriccus minor*	IndoMalay	0	0.0084	-	2.81	0.7937	0.5594	17.92
Tyrannidae	*Hemitriccus mirandae*	IndoMalay	0	-0.0639	-	3.25	0.8180	0.5007	5.29
Tyrannidae	*Hemitriccus nidipendulus*	IndoMalay	0	-0.0458	-	2.81	0.7939	0.5180	13.24
Tyrannidae	*Hemitriccus obsoletus*	Australasia	0	-0.1983	3.4317	2.74	0.7751	0.2720	16.55
Tyrannidae	*Hemitriccus orbitatus*	IndoMalay	0	-0.0500	-	2.82	0.7913	0.5929	11.84
Tyrannidae	*Hemitriccus rufigularis*	IndoMalay	0	-0.0602	-	3.14	0.8288	0.6042	31.31
Tyrannidae	*Hemitriccus spodiops*	Neotropics	0	0.0005	6.2934	3.52	0.7505	0.3010	88.45
Tyrannidae	*Hemitriccus striaticollis*	Neotropics	0	-5.5313	3.1982	4.03	0.6824	0.2752	38.92
Tyrannidae	*Hemitriccus zosterops*	Neotropics	0	0.0875	8.8945	3.63	0.7563	0.3467	73.51
Tyrannidae	*Hirundinea ferruginea*	IndoMalay	0	0.0238	-	3.28	0.7811	0.3950	42.30
Tyrannidae	*Hymenops perspicillatus*	Nearctic	0	-0.4548	5.9777	2.89	0.6824	0.1227	54.42
Tyrannidae	*Inezia caudata*	Neotropics	0	-0.2071	-	3.44	0.7416	0.6855	6.66
Tyrannidae	*Inezia inornata*	Neotropics	0	0.2005	3.2046	2.41	0.5788	0.0000	0.00
Tyrannidae	*Inezia subflava*	Neotropics	0	-0.0140	0.9773	3.35	0.7599	0.4664	33.60
Tyrannidae	*Inezia tenuirostris*	Neotropics	0	-0.0040	-	3.72	0.7678	0.1849	33.13
Tyrannidae	*Knipolegus aterrimus*	IndoMalay	0	-0.3802	-	3.18	0.8407	0.9532	2.48
Tyrannidae	*Knipolegus cyanirostris*	Palearctic	0	-0.7991	-	3.94	0.8257	0.5402	47.93
Tyrannidae	*Knipolegus franciscanus*	Nearctic	0	-0.3935	-	3.86	0.8382	0.3697	51.70
Tyrannidae	*Knipolegus hudsoni*	IndoMalay	0	-0.3240	3.2826	3.19	0.8432	0.3511	32.48
Tyrannidae	*Knipolegus lophotes*	Neotropics	0	-0.4968	-	3.02	0.8095	0.3967	79.45
Tyrannidae	*Knipolegus nigerrimus*	Afrotropics	0	-0.4269	5.6802	3.83	0.8756	0.8074	10.99
Tyrannidae	*Knipolegus poecilocercus*	IndoMalay	0	-0.4660	-	3.35	0.8422	0.7675	38.17
Tyrannidae	*Knipolegus poecilurus*	Afrotropics	0	-0.1511	-	3.52	0.8534	0.6151	39.66
Tyrannidae	*Knipolegus signatus*	Afrotropics	0	0.0968	-	3.26	0.8129	0.3023	12.74
Tyrannidae	*Knipolegus striaticeps*	Palearctic	0	-0.8330	-	3.28	0.7576	0.4027	30.16
Tyrannidae	*Lathrotriccus euleri*	Afrotropics	0	-0.1223	14.9998	3.63	0.8409	0.8164	83.46
Tyrannidae	*Lathrotriccus griseipectus*	Palearctic	0	-0.7005	-	4.59	0.8539	0.3036	2.80
Tyrannidae	*Legatus leucophaius*	Palearctic	0	-0.7726	-	4.64	0.8561	0.5264	9.38
Tyrannidae	*Leptopogon amaurocephalus*	Palearctic	0	-0.5026	0.0000	3.59	0.7775	0.2729	1.50
Tyrannidae	*Leptopogon rufipectus*	Palearctic	0	-0.3920	-	5.14	0.8424	0.1392	0.06
Tyrannidae	*Leptopogon superciliaris*	Australasia	0	-0.6018	0.0000	2.99	0.7931	0.4783	19.57
Tyrannidae	*Leptopogon taczanowskii*	Nearctic	0	-0.6137	2.2589	4.24	0.8261	0.1597	10.40
Tyrannidae	*Lessonia oreas*	Palearctic	0	-0.5845	7.5608	3.53	0.7256	0.4162	0.21
Tyrannidae	*Lessonia rufa*	Neotropics	0	-0.1623	-	3.85	0.8500	0.4750	41.75
Tyrannidae	*Lophotriccus eulophotes*	Palearctic	1	-0.6903	0.4538	3.24	0.6302	0.5120	62.00
Tyrannidae	*Lophotriccus galeatus*	Palearctic	0	-0.7394	-	3.73	0.7599	0.7032	24.14
Tyrannidae	*Lophotriccus pileatus*	Neotropics	1	3.3081	4.8510	3.64	0.5788	0.0000	90.00
Tyrannidae	*Lophotriccus vitiosus*	IndoMalay	0	-0.0804	-	2.96	0.8226	0.3024	7.93
Tyrannidae	*Machetornis rixosa*	Australasia	0	-0.0526	-	2.78	0.7656	0.2188	22.89
Tyrannidae	*Mecocerculus calopterus*	Afrotropics	1	1.4367	7.6707	3.18	0.6598	0.5809	59.02
Tyrannidae	*Mecocerculus hellmayri*	Afrotropics	0	0.0833	7.6893	3.14	0.7528	0.0887	12.89
Tyrannidae	*Mecocerculus leucophrys*	Afrotropics	0	-0.6788	5.4860	3.32	0.6891	0.2276	78.01
Tyrannidae	*Mecocerculus minor*	IndoMalay	0	0.0224	1.2485	2.82	0.7687	0.4907	3.90
Tyrannidae	*Mecocerculus poecilocercus*	IndoMalay	0	0.0312	1.7359	2.87	0.7701	0.3692	6.57
Tyrannidae	*Mecocerculus stictopterus*	IndoMalay	0	-0.0880	-	2.88	0.7962	0.6225	65.63
Tyrannidae	*Megarynchus pitangua*	Neotropics	0	0.5056	-	2.41	0.5788	0.0000	0.00
Tyrannidae	*Mionectes macconnelli*	Neotropics	1	-0.4095	2.5352	3.71	0.6221	0.5682	72.23
Tyrannidae	*Mionectes oleagineus*	Neotropics	0	0.0000	3.0824	3.39	0.6120	0.4058	71.81
Tyrannidae	*Mionectes olivaceus*	Neotropics	0	-0.1405	16.5572	3.91	0.7327	0.1440	50.98
Tyrannidae	*Mionectes rufiventris*	Neotropics	0	-2.9054	5.4539	3.74	0.6906	0.2738	64.25
Tyrannidae	*Mionectes striaticollis*	Neotropics	0	0.0617	14.4849	3.66	0.7701	0.4268	81.70
Tyrannidae	*Mitrephanes olivaceus*	Australasia	0	-0.0626	12.8353	2.81	0.7422	0.1715	24.22
Tyrannidae	*Mitrephanes phaeocercus*	IndoMalay	0	-0.0576	2.7907	3.17	0.7906	0.3212	37.07
Tyrannidae	*Muscigralla brevicauda*	Australasia	0	-0.4453	-	3.09	0.7600	0.3434	80.17
Tyrannidae	*Muscisaxicola albifrons*	Australasia	0	-0.4620	-	2.48	0.6370	0.1317	72.10
Tyrannidae	*Muscisaxicola albilora*	Australasia	0	-0.1792	-	2.78	0.7490	0.6790	15.97
Tyrannidae	*Muscisaxicola capistratus*	Australasia	0	-0.2806	3.8239	3.06	0.7634	0.2527	40.72
Tyrannidae	*Muscisaxicola cinereus*	Australasia	0	-0.2971	-	2.50	0.6716	0.2680	24.42
Tyrannidae	*Muscisaxicola fluviatilis*	Neotropics	0	-0.0943	-	4.09	0.7972	0.5624	18.19
Tyrannidae	*Muscisaxicola frontalis*	Neotropics	0	0.1175	-	3.88	0.7777	0.3669	64.57
Tyrannidae	*Muscisaxicola griseus*	Neotropics	0	-0.0872	-	3.96	0.8297	0.5162	23.61
Tyrannidae	*Muscisaxicola juninensis*	Neotropics	0	-0.1248	-	3.99	0.8369	0.4502	6.79
Tyrannidae	*Muscisaxicola maclovianus*	Afrotropics	1	-0.2006	0.2512	3.23	0.6302	0.4559	89.85
Tyrannidae	*Muscisaxicola maculirostris*	Afrotropics	0	-0.2192	9.2268	3.66	0.8094	0.6512	1.87
Tyrannidae	*Muscisaxicola rufivertex*	Afrotropics	0	-0.3680	10.8251	4.02	0.7855	0.4308	43.67
Tyrannidae	*Myiarchus antillarum*	Australasia	0	-0.0947	-	2.71	0.7136	0.5723	62.74

Family	Species	Realm	Threat	Latitude	Elevation	Anomaly	Size	Shape	Orientation
Tyrannidae	*Myiarchus apicalis*	IndoMalay	0	-0.0088	3.5030	2.64	0.7256	0.2114	67.51
Tyrannidae	*Myiarchus barbirostris*	IndoMalay	1	-0.0300	2.9270	3.07	0.8100	0.2894	48.41
Tyrannidae	*Myiarchus cephalotes*	Afrotropics	0	-0.1340	16.0852	3.49	0.8160	0.4806	75.69
Tyrannidae	*Myiarchus cinerascens*	Afrotropics	0	-0.0643	-	3.45	0.8072	0.3064	19.89
Tyrannidae	*Myiarchus crinitus*	Afrotropics	0	0.8616	-	3.73	0.7822	0.4414	8.95
Tyrannidae	*Myiarchus ferox*	Afrotropics	0	-0.0792	6.4512	3.50	0.8399	0.5985	33.94
Tyrannidae	*Myiarchus magnirostris*	Afrotropics	0	0.0174	-	3.32	0.8213	0.3706	18.93
Tyrannidae	*Myiarchus nuttingi*	Afrotropics	0	-0.0339	-	3.27	0.8196	0.3018	13.63
Tyrannidae	*Myiarchus panamensis*	Palearctic	0	-0.7266	-	4.29	0.8318	0.5380	19.27
Tyrannidae	*Myiarchus phaeocephalus*	Afrotropics	0	0.0668	-	3.20	0.7912	0.2322	9.91
Tyrannidae	*Myiarchus sagrae*	Palearctic	0	-0.5736	10.9204	3.50	0.7994	-	-
Tyrannidae	*Myiarchus sagrae*	Palearctic	0	-0.5736	10.9204	3.50	0.7994	0.5274	14.48
Tyrannidae	*Myiarchus semirufus*	Afrotropics	0	0.0582	6.5974	3.49	0.7840	0.3177	1.41
Tyrannidae	*Myiarchus stolidus*	Palearctic	0	-0.8020	6.2183	4.49	0.8000	0.5891	6.05
Tyrannidae	*Myiarchus swainsoni*	Afrotropics	0	0.0073	-	3.32	0.8146	0.6273	7.96
Tyrannidae	*Myiarchus tuberculifer*	Afrotropics	1	8.0614	4.1302	3.40	0.7040	0.4675	58.25
Tyrannidae	*Myiarchus tyrannulus*	IndoMalay	0	-0.6241	6.8129	3.29	0.7901	0.5192	26.03
Tyrannidae	*Myiarchus validus*	Afrotropics	0	0.0476	-	3.21	0.7957	0.2326	13.48
Tyrannidae	*Myiarchus venezuelensis*	IndoMalay	1	-0.0021	2.3243	2.65	0.7030	0.4071	59.43
Tyrannidae	*Myiarchus yucatanensis*	Palearctic	0	-0.7831	11.1892	4.37	0.7698	0.2508	33.44
Tyrannidae	*Myiobius atricaudus*	Australasia	0	0.5171	1.9575	2.60	0.6120	0.4082	18.43
Tyrannidae	*Myiobius barbatus*	Afrotropics	0	0.1675	9.4985	3.26	0.7921	0.4217	7.27
Tyrannidae	*Myiobius sulphureipygius*	Palearctic	0	-1.1464	12.5124	4.36	0.8428	0.3919	19.21
Tyrannidae	*Myiobius villosus*	Palearctic	0	-0.6387	5.1980	4.48	0.8610	0.2748	1.88
Tyrannidae	*Myiodynastes bairdii*	Neotropics	0	8.5702	10.3826	3.35	0.7416	0.1192	83.80
Tyrannidae	*Myiodynastes chrysocephalus*	Neotropics	0	2.2217	12.3891	3.98	0.7533	0.2811	36.62
Tyrannidae	*Myiodynastes hemichrysus*	Neotropics	0	2.7226	11.0614	4.10	0.7660	0.2790	49.36
Tyrannidae	*Myiodynastes luteiventris*	Neotropics	0	-0.5046	4.0010	2.00	0.7600	0.4568	81.09
Tyrannidae	*Myiodynastes maculatus*	Neotropics	0	-0.2409	14.5832	3.44	0.8050	0.2365	85.31
Tyrannidae	*Myiopagis caniceps*	Afrotropics	0	0.2378	4.1372	3.45	0.8042	0.2297	6.03
Tyrannidae	*Myiopagis cotta*	Neotropics	0	0.2061	5.9408	2.92	0.5985	0.5771	46.13
Tyrannidae	*Myiopagis flavivertex*	Neotropics	0	-0.3083	2.5720	2.48	0.6428	0.0604	13.44
Tyrannidae	*Myiopagis gaimardii*	Neotropics	0	0.0012	3.3639	2.46	0.6921	0.2089	5.23
Tyrannidae	*Myiopagis olallai*	Neotropics	0	-0.1977	11.1418	3.04	0.6804	0.2128	30.89
Tyrannidae	*Myiopagis viridicata*	Neotropics	0	-0.0592	14.8871	3.78	0.7728	0.2385	32.01
Tyrannidae	*Myiophobus cryptoxanthus*	Neotropics	0	-0.1300	8.7921	3.66	0.7363	0.1476	26.72
Tyrannidae	*Myiophobus cryptoxanthus*	Neotropics	0	-0.1300	8.7921	3.66	0.7363	-	-
Tyrannidae	*Myiophobus fasciatus*	Palearctic	0	-0.5684	7.5555	3.96	0.7895	0.3287	3.59
Tyrannidae	*Myiophobus flavicans*	Palearctic	0	-1.0259	10.1492	4.51	0.8401	0.4243	6.60
Tyrannidae	*Myiophobus inornatus*	Palearctic	0	-0.0822	9.5939	4.35	0.7595	0.2683	26.26
Tyrannidae	*Myiophobus phoenicomitra*	IndoMalay	0	-0.2552	10.2553	3.72	0.7968	0.4655	11.65
Tyrannidae	*Myiophobus roraimae*	Neotropics	0	-0.2474	-	3.73	0.8550	0.6161	51.63
Tyrannidae	*Myiornis albiventris*	Afrotropics	0	-0.0668	-	3.54	0.8617	0.6550	35.40
Tyrannidae	*Myiornis atricapillus*	IndoMalay	0	-0.0951	-	3.30	0.8016	0.5565	5.07
Tyrannidae	*Myiornis auricularis*	Australasia	0	-0.2412	-	3.02	0.7944	0.8090	5.39
Tyrannidae	*Myiotheretes fumigatus*	Australasia	0	-0.6445	-	2.57	0.6370	0.3880	82.91
Tyrannidae	*Myiotheretes pernix*	Australasia	0	-0.5259	-	3.04	0.7858	0.5337	89.78
Tyrannidae	*Myiotheretes striaticollis*	Australasia	0	-0.0168	-	2.48	0.6120	0.0400	36.10
Tyrannidae	*Myiotriccus ornatus*	Australasia	0	-0.0168	-	2.48	0.6690	0.5741	71.12
Tyrannidae	*Myiozetetes cayanensis*	Australasia	0	-0.3353	4.8505	2.50	0.6716	0.2680	24.42
Tyrannidae	*Myiozetetes granadensis*	Australasia	0	-0.4352	-	3.16	0.8086	0.6586	31.81
Tyrannidae	*Myiozetetes luteiventris*	Australasia	0	-0.3737	-	3.23	0.8062	0.5220	48.40
Tyrannidae	*Myiozetetes similis*	Australasia	0	-0.2051	-	3.03	0.7697	0.5025	8.96
Tyrannidae	*Neopipo cinnamomea*	IndoMalay	0	-0.0217	-	3.11	0.7165	0.1263	66.49
Tyrannidae	*Neoxolmis rufiventris*	Afrotropics	0	-3.1421	-	3.68	0.7441	0.2025	12.96
Tyrannidae	*Nephelomyias lintoni*	Afrotropics	0	0.1034	-	3.28	0.7542	0.1902	14.84
Tyrannidae	*Nephelomyias ochraceiventris*	Afrotropics	0	0.0847	-	3.59	0.8335	0.2480	10.13
Tyrannidae	*Nephelomyias pulcher*	Afrotropics	0	4.2171	-	3.44	0.7151	0.5538	79.54
Tyrannidae	*Ochthoeca cinnamomeiventris*	IndoMalay	1	-0.0052	1.0046	2.84	0.7869	0.4677	3.56
Tyrannidae	*Ochthoeca diadema*	IndoMalay	0	-0.6446	14.6278	3.44	0.8035	0.2818	10.71
Tyrannidae	*Ochthoeca frontalis*	IndoMalay	1	-0.0222	2.5601	2.64	0.7318	0.3285	67.68
Tyrannidae	*Ochthoeca fumicolor*	Neotropics	0	-0.2249	2.4288	3.49	0.8427	0.5057	44.61
Tyrannidae	*Ochthoeca jelskii*	Neotropics	0	0.0643	-	4.31	0.6716	0.6180	58.04
Tyrannidae	*Ochthoeca leucophrys*	Neotropics	0	-0.9918	-	3.64	0.7406	0.3495	84.29
Tyrannidae	*Ochthoeca oenanthoides*	Neotropics	0	-0.1483	-	3.35	0.7277	0.6028	5.42
Tyrannidae	*Ochthoeca piurae*	Neotropics	0	-0.0471	-	3.88	0.8300	0.4423	44.14
Tyrannidae	*Ochthoeca pulchella*	Neotropics	0	0.0351	-	4.16	0.8128	0.4248	0.36
Tyrannidae	*Ochthoeca rufipectoralis*	Neotropics	0	-0.0495	-	4.22	0.7696	0.5463	21.77
Tyrannidae	*Ochthornis littoralis*	Australasia	0	-0.6399	-	3.17	0.7985	0.4014	3.14
Tyrannidae	*Oncostoma cinereigulare*	Neotropics	0	-0.1432	0.4017	3.01	0.7211	0.6052	64.09
Tyrannidae	*Oncostoma olivaceum*	Neotropics	0	-0.0127	1.4414	4.08	0.8252	0.5743	43.67
Tyrannidae	*Onychorhynchus coronatus*	Nearctic	0	-0.4326	-	2.97	0.7374	0.0690	1.47
Tyrannidae	*Onychorhynchus mexicanus*	Nearctic	1	-0.3752	0.0000	2.95	0.6120	0.1210	62.99
Tyrannidae	*Onychorhynchus occidentalis*	Neotropics	0	-2.1348	11.6339	4.13	0.7544	0.4933	88.46

Family	Species	Realm	Threat	Latitude	Elevation	Anomaly	Size	Shape	Orientation
Tyrannidae	*Onychorhynchus swainsoni*	Neotropics	0	-0.3195	8.7041	3.09	0.6974	0.0936	83.61
Tyrannidae	*Ornithion brunneicapillus*	Palearctic	0	-0.9657	11.7609	4.38	0.8448	0.2676	0.22
Tyrannidae	*Ornithion inerme*	Palearctic	0	-0.9197	8.0548	4.14	0.7747	0.3561	29.80
Tyrannidae	*Ornithion semiflavum*	Neotropics	1	-0.1136	11.7069	3.38	0.7406	0.2903	53.06
Tyrannidae	*Phaeomyias murina*	Neotropics	0	-14.5625	-	4.18	0.7314	0.4129	38.87
Tyrannidae	*Phelpsia inornatus*	Neotropics	0	0.0456	6.7860	3.61	0.7472	0.1164	24.31
Tyrannidae	*Phyllomyias burmeisteri*	Australasia	0	0.2630	-	2.52	0.5788	0.0000	90.00
Tyrannidae	*Phyllomyias cinereiceps*	Australasia	1	0.1005	0.4286	2.48	0.6221	0.5774	71.57
Tyrannidae	*Phyllomyias fasciatus*	Australasia	0	-0.0810	7.9595	2.56	0.6523	0.1875	30.24
Tyrannidae	*Phyllomyias griseiceps*	Australasia	0	-0.0615	-	2.81	0.7631	0.2775	23.64
Tyrannidae	*Phyllomyias griseocapilla*	Australasia	0	-0.1708	4.8709	2.49	0.5985	0.0448	40.11
Tyrannidae	*Phyllomyias nigrocapillus*	Australasia	0	-0.2162	3.3572	2.89	0.7711	0.3227	54.94
Tyrannidae	*Phyllomyias plumbeiceps*	Australasia	0	0.1156	3.1304	2.48	0.6370	0.1926	10.82
Tyrannidae	*Phyllomyias reiseri*	IndoMalay	0	-0.0057	1.9313	2.84	0.7885	0.4811	6.07
Tyrannidae	*Phyllomyias sclateri*	IndoMalay	0	0.0008	2.7449	2.84	0.7896	0.5795	13.88
Tyrannidae	*Phyllomyias urichi*	Afrotropics	0	-0.1580	4.2998	3.06	0.7323	0.1629	76.07
Tyrannidae	*Phyllomyias uropygialis*	Afrotropics	0	-0.0458	2.3261	3.15	0.7172	0.2399	57.76
Tyrannidae	*Phyllomyias weedeni*	Afrotropics	0	-0.3858	-	4.21	0.7756	0.4960	47.44
Tyrannidae	*Phylloscartes ceciliae*	Neotropics	0	-0.5011	8.1165	3.63	0.7128	0.3227	15.26
Tyrannidae	*Phylloscartes ceciliae*	Neotropics	0	-0.5011	8.1165	3.63	0.7128	-	-
Tyrannidae	*Phylloscartes chapmani*	Neotropics	0	-0.2628	-	3.22	0.7854	0.3498	31.47
Tyrannidae	*Phylloscartes difficilis*	Neotropics	0	-0.0210	4.8338	4.05	0.8302	0.6238	5.61
Tyrannidae	*Phylloscartes eximius*	Neotropics	0	0.1296	3.3680	4.08	0.8150	0.5565	10.53
Tyrannidae	*Phylloscartes flaviventris*	Neotropics	0	0.4123	3.4552	3.19	0.7256	0.6362	71.29
Tyrannidae	*Phylloscartes flavovirens*	Neotropics	0	-0.1871	-	3.42	0.7957	0.4641	33.99
Tyrannidae	*Phylloscartes kronei*	Neotropics	0	0.0958	-	4.03	0.8272	0.6993	17.11
Tyrannidae	*Phylloscartes lanyoni*	Neotropics	0	0.0982	3.6544	4.03	0.8237	0.6742	15.33
Tyrannidae	*Phylloscartes nigrifrons*	Neotropics	0	-0.2934	9.7599	3.61	0.8092	0.3794	38.13
Tyrannidae	*Phylloscartes ophthalmicus*	Neotropics	0	-0.2698	7.9626	3.59	0.8356	0.6131	61.74
Tyrannidae	*Phylloscartes orbitalis*	Neotropics	0	-0.0935	0.2325	4.31	0.7256	0.5048	38.87
Tyrannidae	*Phylloscartes oustaleti*	Neotropics	0	0.0935	1.4389	3.97	0.8017	0.7477	23.60
Tyrannidae	*Phylloscartes parkeri*	Neotropics	0	0.1908	2.6438	4.04	0.8215	0.5208	3.18
Tyrannidae	*Phylloscartes paulista*	Neotropics	0	-0.4277	9.0459	2.95	0.8150	0.6084	71.22
Tyrannidae	*Phylloscartes poecilotis*	Neotropics	0	-10.2944	2.7255	4.08	0.6974	0.1298	34.26
Tyrannidae	*Phylloscartes roquettei*	Neotropics	0	0.5108	5.3294	3.36	0.6921	0.2037	74.44
Tyrannidae	*Phylloscartes superciliaris*	IndoMalay	0	-0.0898	-	2.99	0.8182	0.5495	57.48
Tyrannidae	*Phylloscartes sylviolus*	Afrotropics	1	9.8802	3.6824	3.45	0.6523	0.5624	80.69
Tyrannidae	*Phylloscartes venezuelanus*	Neotropics	1	0.0304	0.3087	3.37	0.6986	0.2394	56.31
Tyrannidae	*Phylloscartes ventralis*	Neotropics	0	-0.0845	-	3.92	0.7909	0.6441	35.45
Tyrannidae	*Phylloscartes virescens*	Neotropics	0	-0.1209	-	4.04	0.8099	0.4580	12.42
Tyrannidae	*Pitangus lictor*	Neotropics	0	-0.2338	-	3.44	0.7934	0.2701	71.41
Tyrannidae	*Pitangus sulphuratus*	Neotropics	0	-1.2152	8.5391	4.00	0.7198	0.1065	35.16
Tyrannidae	*Platyrinchus cancrominus*	Nearctic	0	-0.6401	15.0904	4.07	0.8220	0.5405	58.10
Tyrannidae	*Platyrinchus coronatus*	Neotropics	0	0.0117	13.1397	3.68	0.7281	0.2606	22.98
Tyrannidae	*Platyrinchus flavigularis*	Neotropics	0	-0.0290	-	3.85	0.7744	0.7812	49.16
Tyrannidae	*Platyrinchus leucoryphus*	Neotropics	0	0.8362	-	4.08	0.7634	0.2191	37.83
Tyrannidae	*Platyrinchus mystaceus*	Neotropics	0	0.3409	6.5237	4.27	0.7510	0.6693	31.45
Tyrannidae	*Platyrinchus platyrhynchos*	Neotropics	0	0.0541	-	3.89	0.8181	0.5310	1.22
Tyrannidae	*Platyrinchus saturatus*	Neotropics	0	-0.2372	-	4.02	0.8269	0.6015	21.67
Tyrannidae	*Poecilotriccus albifacies*	Neotropics	0	-2.3785	10.3868	4.05	0.7642	0.2324	43.34
Tyrannidae	*Poecilotriccus calopterus*	Afrotropics	0	0.0873	-	3.27	0.7374	0.4230	44.61
Tyrannidae	*Poecilotriccus capitalis*	Afrotropics	0	-0.0836	-	3.84	0.8211	0.3527	58.16
Tyrannidae	*Poecilotriccus fumifrons*	Afrotropics	0	1.3006	-	3.73	0.7666	0.3419	10.18
Tyrannidae	*Poecilotriccus latirostris*	Afrotropics	0	0.0860	-	3.48	0.8120	0.1905	3.85
Tyrannidae	*Poecilotriccus luluae*	Afrotropics	0	0.0358	-	3.19	0.7785	0.1231	10.40
Tyrannidae	*Poecilotriccus plumbeiceps*	Afrotropics	0	1.1903	7.0546	3.43	0.7144	0.4573	73.18
Tyrannidae	*Poecilotriccus pulchellus*	Afrotropics	0	4.2524	-	3.75	0.7522	0.2342	1.55
Tyrannidae	*Poecilotriccus ruficeps*	Afrotropics	0	-0.0913	-	3.19	0.7784	0.2662	9.66
Tyrannidae	*Poecilotriccus russatus*	Afrotropics	1	0.0000	-	3.39	0.6598	0.5406	4.84
Tyrannidae	*Poecilotriccus senex*	Afrotropics	0	2.0299	-	3.53	0.7572	0.4488	74.14
Tyrannidae	*Poecilotriccus sylvia*	Afrotropics	0	0.0486	-	3.40	0.8038	0.7059	9.37
Tyrannidae	*Polioxolmis rufipennis*	Afrotropics	1	-0.2296	-	2.98	0.6740	0.2195	64.67
Tyrannidae	*Polystictus pectoralis*	Palearctic	0	-0.9435	-	6.56	0.8013	0.0261	0.73
Tyrannidae	*Polystictus superciliaris*	Palearctic	0	-0.8582	-	6.33	0.8245	0.0274	0.34
Tyrannidae	*Pseudelaenia leucospodia*	Afrotropics	0	-0.2342	-	4.08	0.8107	0.7040	48.62
Tyrannidae	*Pseudocolopteryx acutipennis*	Afrotropics	0	18.1889	-	3.61	0.6523	0.3758	83.06
Tyrannidae	*Pseudocolopteryx citreola*	IndoMalay	0	-0.0510	-	3.11	0.8062	0.5528	56.04
Tyrannidae	*Pseudocolopteryx dinelliana*	IndoMalay	0	-0.1090	-	3.44	0.8111	0.8273	71.49
Tyrannidae	*Pseudocolopteryx flaviventris*	Afrotropics	0	-0.0707	-	3.31	0.7900	0.5222	35.91
Tyrannidae	*Pseudocolopteryx sclateri*	Afrotropics	0	-0.0766	-	3.53	0.8566	0.6200	34.90
Tyrannidae	*Pseudotriccus pelzelni*	IndoMalay	0	0.0782	-	2.52	0.5985	0.0024	45.41
Tyrannidae	*Pseudotriccus ruficeps*	IndoMalay	0	0.0458	2.5036	2.84	0.7861	0.4266	0.86
Tyrannidae	*Pseudotriccus simplex*	IndoMalay	0	0.1385	-	2.86	0.7452	0.4811	31.52
Tyrannidae	*Pyrocephalus rubinus*	Neotropics	0	-0.0692	2.2268	4.10	0.7941	0.5500	20.66

Family	Species	Realm	Threat	Latitude	Elevation	Anomaly	Size	Shape	Orientation
Tyrannidae	*Pyrrhomyias cinnamomeus*	IndoMalay	0	-0.1357	-	3.12	0.7869	0.3796	67.64
Tyrannidae	*Ramphotrigon fuscicauda*	Neotropics	0	-0.1759	-	3.84	0.8526	0.5003	35.79
Tyrannidae	*Ramphotrigon megacephalum*	Australasia	0	-0.7020	-	2.50	0.7128	0.4263	4.95
Tyrannidae	*Ramphotrigon ruficauda*	IndoMalay	0	-0.0915	-	2.94	0.8089	0.4251	56.74
Tyrannidae	*Rhynchocyclus brevirostris*	Neotropics	0	0.3332	-	3.41	0.6598	0.3721	44.46
Tyrannidae	*Rhynchocyclus fulvipectus*	Neotropics	0	-0.2060	-	2.50	0.7217	0.1682	18.06
Tyrannidae	*Rhynchocyclus pacificus*	Nearctic	0	-0.7561	-	4.30	0.8417	0.5228	16.63
Tyrannidae	*Rhytipterna holerythra*	Neotropics	0	-0.3329	-	3.19	0.7834	0.3218	35.99
Tyrannidae	*Rhytipterna immunda*	Afrotropics	1	-0.1378	7.1301	3.07	0.7245	0.1652	74.14
Tyrannidae	*Rhytipterna simplex*	Neotropics	1	0.0000	8.9717	3.55	0.6562	0.7008	75.30
Tyrannidae	*Satrapa icterophrys*	IndoMalay	0	-0.1036	-	2.75	0.7172	0.2263	32.39
Tyrannidae	*Sayornis nigricans*	Australasia	0	-0.0818	12.1888	2.81	0.7432	0.1663	24.39
Tyrannidae	*Sayornis phoebe*	Australasia	0	-0.0803	-	2.80	0.7495	0.1827	23.45
Tyrannidae	*Sayornis saya*	IndoMalay	0	-0.1014	-	3.38	0.8238	0.4457	14.72
Tyrannidae	*Serpophaga cinerea*	IndoMalay	1	-0.0201	6.1668	3.02	0.6842	0.1683	67.59
Tyrannidae	*Serpophaga hypoleuca*	Neotropics	0	-0.5303	1.4566	3.32	0.8051	0.5306	71.18
Tyrannidae	*Serpophaga munda*	Afrotropics	0	-0.1177	10.1236	3.32	0.7631	0.7448	33.70
Tyrannidae	*Serpophaga nigricans*	Afrotropics	1	0.9254	9.3678	3.41	0.6997	0.3655	67.12
Tyrannidae	*Serpophaga subcristata*	Australasia	0	-0.0097	-	2.71	0.7185	0.5623	60.71
Tyrannidae	*Sirystes sibilator*	Neotropics	0	0.0131	13.5075	3.37	0.7505	0.2070	60.08
Tyrannidae	*Stigmatura budytoides*	Palearctic	0	-20.7703	5.8328	3.32	0.7403	0.8604	31.27
Tyrannidae	*Stigmatura napensis*	Neotropics	0	-0.1892	-	3.80	0.8518	0.4575	38.51
Tyrannidae	*Sublegatus arenarum*	Neotropics	1	-0.2279	4.1834	3.00	0.7120	0.1766	22.24
Tyrannidae	*Sublegatus modestus*	Neotropics	1	-0.5640	5.0463	2.77	0.7428	0.4300	28.06
Tyrannidae	*Sublegatus obscurior*	Neotropics	0	-0.6607	0.1499	2.87	0.7340	0.2874	80.94
Tyrannidae	*Suiriri islerorum*	Neotropics	0	-0.4375	-	3.45	0.8092	0.9192	83.13
Tyrannidae	*Suiriri suiriri*	Neotropics	0	-0.0455	-	3.90	0.7927	0.4618	0.84
Tyrannidae	*Tachuris rubrigastra*	Neotropics	0	-0.3627	7.9814	3.20	0.7945	0.3686	47.99
Tyrannidae	*Taeniotriccus andrei*	Palearctic	0	-0.4654	12.1080	3.96	0.7461	0.4565	52.57
Tyrannidae	*Terenotriccus erythrurus*	Neotropics	1	0.0000	4.0653	3.48	0.6370	0.5533	59.04
Tyrannidae	*Todirostrum chrysocrotaphum*	Palearctic	0	9.2452	5.9817	4.43	0.8047	0.2957	1.97
Tyrannidae	*Todirostrum cinereum*	Palearctic	0	1.5244	12.5537	4.45	0.7894	0.3774	8.06
Tyrannidae	*Todirostrum maculatum*	Palearctic	0	-3.5556	6.5642	3.86	0.7256	0.3439	73.06
Tyrannidae	*Todirostrum nigriceps*	Palearctic	0	-12.0560	7.6779	4.18	0.7643	0.6430	2.41
Tyrannidae	*Todirostrum pictum*	Palearctic	0	-0.9861	-	4.64	0.8300	0.1615	6.18
Tyrannidae	*Todirostrum poliocephalum*	Neotropics	0	-0.3900	0.3067	3.20	0.7158	0.2077	78.17
Tyrannidae	*Tolmomyias assimilis*	Neotropics	0	-0.1794	-	3.91	0.8492	0.7394	34.36
Tyrannidae	*Tolmomyias flaviventris*	Neotropics	0	-0.3379	-	3.16	0.7917	0.4861	34.29
Tyrannidae	*Tolmomyias poliocephalus*	Neotropics	1	-0.4250	4.7733	3.64	0.6690	0.4340	60.57
Tyrannidae	*Tolmomyias sulphurescens*	Neotropics	0	-0.1759	1.9956	3.14	0.6891	0.1642	57.90
Tyrannidae	*Tolmomyias traylori*	Neotropics	0	0.0634	5.0414	3.48	0.7781	0.2439	59.70
Tyrannidae	*Tumbezia salvini*	Neotropics	0	0.2049	1.5783	4.02	0.8219	0.5310	4.76
Tyrannidae	*Tyrannopsis sulphurea*	Afrotropics	0	0.7882	6.0519	3.81	0.7933	0.4050	2.55
Tyrannidae	*Tyrannulus elatus*	Afrotropics	0	-0.0431	-	3.28	0.8204	0.3267	14.76
Tyrannidae	*Tyrannus albogularis*	Afrotropics	0	0.0822	8.3150	3.32	0.7913	0.6562	83.97
Tyrannidae	*Tyrannus caudifasciatus*	Afrotropics	0	-0.3221	7.7311	3.93	0.8145	0.7348	39.80
Tyrannidae	*Tyrannus couchii*	Afrotropics	0	0.0721	-	3.34	0.8033	0.6119	61.55
Tyrannidae	*Tyrannus crassirostris*	IndoMalay	0	0.0495	-	2.84	0.7883	0.4910	7.10
Tyrannidae	*Tyrannus cubensis*	Neotropics	0	-0.3315	-	3.51	0.8097	0.5528	10.09
Tyrannidae	*Tyrannus dominicensis*	Neotropics	0	-0.3239	3.6182	2.69	0.7120	0.1862	42.13
Tyrannidae	*Tyrannus forficatus*	Afrotropics	1	-0.0849	16.1866	3.61	0.8405	0.7837	30.64
Tyrannidae	*Tyrannus melancholicus*	Palearctic	0	-1.4232	-	6.09	0.8226	0.0493	0.62
Tyrannidae	*Tyrannus niveigularis*	Nearctic	0	-0.7221	-	4.31	0.8265	0.1799	9.62
Tyrannidae	*Tyrannus savana*	Palearctic	1	-0.6407	-	4.58	0.7403	0.7524	59.99
Tyrannidae	*Tyrannus tyrannus*	Nearctic	0	-0.4265	-	4.13	0.8258	0.1081	4.83
Tyrannidae	*Tyrannus verticalis*	Palearctic	0	-0.0203	-	5.17	0.8521	0.1113	0.22
Tyrannidae	*Tyrannus vociferans*	Palearctic	0	-0.7763	-	4.99	0.8575	0.1444	0.46
Tyrannidae	*Xenotriccus callizonus*	Neotropics	0	-0.0408	-	2.46	0.6876	0.3855	3.42
Tyrannidae	*Xenotriccus mexicanus*	Australasia	1	-0.2351	5.7554	2.71	0.7179	0.5352	59.02
Tyrannidae	*Xolmis cinereus*	IndoMalay	0	-0.8106	4.2146	2.61	0.6562	0.4622	64.29
Tyrannidae	*Xolmis coronatus*	IndoMalay	0	-0.6153	-	3.31	0.8251	0.5748	24.70
Tyrannidae	*Xolmis dominicanus*	IndoMalay	0	-0.1559	6.2220	3.90	0.7570	0.2023	16.10
Tyrannidae	*Xolmis irupero*	IndoMalay	1	0.2489	7.8676	2.76	0.6370	0.9166	89.25
Tyrannidae	*Xolmis pyrope*	IndoMalay	0	-0.1788	5.4561	3.02	0.7546	0.6518	54.71
Tyrannidae	*Xolmis rubetra*	Afrotropics	0	-0.2966	-	3.84	0.8202	0.9017	66.29
Tyrannidae	*Xolmis salinarum*	Afrotropics	0	-0.0311	7.9792	3.55	0.8335	0.3077	13.59
Tyrannidae	*Xolmis velatus*	Palearctic	0	3.2950	11.5280	4.11	0.7680	0.8549	86.02
Tyrannidae	*Zimmerius acer*	IndoMalay	0	-0.0925	-	3.21	0.8023	0.3481	26.31
Tyrannidae	*Zimmerius albigularis*	Palearctic	1	-0.8979	1.0768	5.00	0.8104	0.1779	2.70
Tyrannidae	*Zimmerius bolivianus*	IndoMalay	0	-0.3337	4.7294	3.58	0.8302	0.3079	19.40
Tyrannidae	*Zimmerius chrysops*	Palearctic	0	-1.0152	0.0000	4.17	0.8035	0.4757	24.98
Tyrannidae	*Zimmerius cinereicapilla*	Afrotropics	0	-0.0357	-	3.34	0.8218	0.5020	24.26
Tyrannidae	*Zimmerius gracilipes*	Australasia	1	0.0660	-	2.65	0.6784	0.0643	8.71
Tyrannidae	*Zimmerius improbus*	IndoMalay	0	-0.0934	-	3.37	0.8050	0.7987	87.36

Family	Species	Realm	Threat	Latitude	Elevation	Anomaly	Size	Shape	Orientation
Tyrannidae	*Zimmerius vilissimus*	Afrotropics	0	2.4089	13.6367	3.59	0.7331	0.4527	83.83
Tyrannidae	*Zimmerius villarejoi*	Afrotropics	0	-0.0252	10.2457	3.41	0.7834	0.1211	75.85
Tyrannidae	*Zimmerius viridiflavus*	Australasia	0	-0.3955	-	2.99	0.8067	0.5323	48.77
Tytonidae	*Phodilus badius*	Neotropics	0	-0.0514	-	3.97	0.8181	0.6491	7.06
Tytonidae	*Phodilus prigoginei*	Neotropics	0	-0.0492	-	3.99	0.7929	0.2846	35.97
Tytonidae	*Tyto alba*	Nearctic	0	-0.5730	-	4.30	0.8324	0.1199	9.71
Tytonidae	*Tyto aurantia*	Palearctic	0	-0.8400	-	5.01	0.8355	0.1392	2.38
Tytonidae	*Tyto capensis*	Palearctic	0	-0.4997	-	4.51	0.8593	0.2280	5.91
Tytonidae	*Tyto glaucops*	Neotropics	0	-0.2743	5.6344	2.47	0.5985	0.0000	0.00
Tytonidae	*Tyto longimembris*	Afrotropics	0	-0.1937	-	3.38	0.7937	0.4795	79.00
Tytonidae	*Tyto nigrobrunnea*	Afrotropics	0	-0.0097	-	3.28	0.8199	0.3426	12.91
Tytonidae	*Tyto novaehollandiae*	Neotropics	0	-0.4282	10.3414	3.79	0.8709	0.3452	54.24
Tytonidae	*Tyto rosenbergii*	Neotropics	1	0.1395	-	1.49	0.5788	0.0000	0.00
Tytonidae	*Tyto sororcula*	Neotropics	1	0.6304	8.5329	3.24	0.5985	0.5771	46.13
Tytonidae	*Tyto soumagnei*	Neotropics	0	-0.1268	8.4855	3.01	0.6804	0.1912	18.73
Tytonidae	*Tyto tenebricosa*	Neotropics	0	0.2301	10.5327	3.63	0.7205	0.2238	23.35
Upupidae	*Upupa epops*	Neotropics	0	-0.0404	2.9496	3.40	0.7849	0.3758	34.12
Upupidae	*Upupa marginata*	Neotropics	0	0.1050	2.3860	3.12	0.7425	0.4936	70.63
Urocynchramidae	*Urocynchramus pylzowi*	Neotropics	0	-0.1014	3.2390	4.05	0.8321	0.7219	7.04
Vangidae	*Artamella viridis*	Neotropics	0	0.1599	-	3.43	0.6961	0.3793	11.29
Vangidae	*Calicalicus madagascariensis*	Nearctic	0	-0.8060	-	4.41	0.7967	0.4748	56.49
Vangidae	*Calicalicus rufocarpalis*	Palearctic	0	-0.6651	20.0280	4.42	0.8243	0.3551	0.22
Vangidae	*Cyanolanius madagascarinus*	Nearctic	0	-0.5198	16.0074	3.86	0.8213	0.2438	47.60
Vangidae	*Euryceros prevostii*	Afrotropics	0	-1.4851	7.4024	3.45	0.6891	0.4686	50.45
Vangidae	*Falculea palliata*	Palearctic	0	-0.4740	-	3.45	0.7158	0.2084	12.64
Vangidae	*Hypositta corallirostris*	Neotropics	0	0.0035	-	3.80	0.7786	0.4332	5.57
Vangidae	*Hypositta perdita*	Neotropics	0	0.4107	-	4.05	0.8217	0.6733	7.21
Vangidae	*Leptopterus chabert*	Nearctic	0	2.7034	10.7326	4.30	0.7942	0.6120	20.59
Vangidae	*Mystacornis crossleyi*	IndoMalay	0	-0.0669	-	3.35	0.7614	0.7171	70.18
Vangidae	*Newtonia amphichroa*	IndoMalay	0	-0.1053	-	3.22	0.7980	0.5564	32.64
Vangidae	*Newtonia archboldi*	Neotropics	0	-0.1181	-	3.92	0.8430	0.6872	29.32
Vangidae	*Newtonia brunneicauda*	Neotropics	1	0.0000	1.2293	2.99	0.5985	0.0870	34.85
Vangidae	*Newtonia fanovanae*	Neotropics	0	-0.1407	-	3.91	0.8247	0.5982	20.86
Vangidae	*Oriolia bernieri*	Neotropics	0	0.0381	3.6526	3.22	0.7245	0.3470	46.32
Vangidae	*Pseudobias wardi*	IndoMalay	0	-0.2797	-	3.32	0.8380	0.4541	1.55
Vangidae	*Schetba rufa*	Australasia	0	-0.3828	-	2.50	0.6716	0.2680	24.42
Vangidae	*Tylas eduardi*	Australasia	0	0.0248	7.3725	2.56	0.6523	0.1875	30.24
Vangidae	*Vanga curvirostris*	IndoMalay	0	-0.0487	2.3993	3.14	0.7296	0.1842	64.36
Vangidae	*Xenopirostris damii*	Neotropics	0	-0.3595	-	3.41	0.7629	0.1763	47.04
Vangidae	*Xenopirostris polleni*	Nearctic	0	-0.6317	-	4.26	0.7926	0.7499	58.20
Vangidae	*Xenopirostris xenopirostris*	Neotropics	0	-0.2181	-	3.68	0.8592	0.5200	44.70
Viduidae	*Anomalospiza imberbis*	Afrotropics	0	-0.1515	7.0801	3.61	0.8095	0.6397	43.75
Viduidae	*Vidua camerunensis*	Neotropics	0	-0.2302	12.7366	3.62	0.7444	0.2941	26.03
Viduidae	*Vidua chalybeata*	Neotropics	0	-0.9391	7.3324	2.47	0.5985	0.0000	0.00
Viduidae	*Vidua codringtoni*	Palearctic	0	-0.7787	5.4784	4.06	0.7844	0.8349	80.25
Viduidae	*Vidua fischeri*	Neotropics	0	0.1768	-	4.08	0.8151	0.6865	14.92
Viduidae	*Vidua funerea*	Neotropics	0	-0.1713	4.9884	3.79	0.8381	0.5632	51.95
Viduidae	*Vidua funerea*	Neotropics	0	-0.1713	4.9884	3.79	0.8381	-	-
Viduidae	*Vidua hypocherina*	Neotropics	0	0.0104	6.4546	3.84	0.7723	0.6895	73.16
Viduidae	*Vidua interjecta*	Afrotropics	0	-0.2188	10.5947	3.71	0.8251	0.8800	3.62
Viduidae	*Vidua larvaticola*	Afrotropics	1	-0.4845	4.3456	3.50	0.6662	0.1456	17.42
Viduidae	*Vidua macroura*	Neotropics	0	0.1548	4.8117	3.08	0.7060	0.3090	75.41
Viduidae	*Vidua maryae*	Neotropics	0	-25.7941	9.1440	3.56	0.6891	0.3063	63.75
Viduidae	*Vidua nigeriae*	Afrotropics	1	0.0547	11.0212	3.71	0.7112	0.1685	65.26
Viduidae	*Vidua obtusa*	Palearctic	0	-0.5085	-	3.92	0.8589	0.2946	24.32
Viduidae	*Vidua paradisaea*	Nearctic	0	-0.7931	11.8630	4.27	0.8597	0.4342	14.56
Viduidae	*Vidua purpurascens*	Palearctic	0	-0.8964	9.8751	3.53	0.7865	0.2945	49.17
Viduidae	*Vidua raricola*	Palearctic	0	-0.9600	-	4.98	0.8273	0.1961	4.79
Viduidae	*Vidua regia*	Neotropics	0	0.0669	6.1107	3.06	0.6302	0.2782	40.02
Viduidae	*Vidua togoensis*	Neotropics	0	-0.2390	7.4562	3.20	0.7804	0.4161	34.51
Viduidae	*Vidua wilsoni*	Neotropics	0	0.0446	2.6383	3.89	0.7951	0.2597	23.76
Vireonidae	*Cyclarhis gujanensis*	Afrotropics	0	-0.0632	-	3.17	0.7567	0.4224	70.12
Vireonidae	*Cyclarhis nigrirostris*	Neotropics	1	-0.1242	0.0000	2.54	0.5788	0.0000	0.00
Vireonidae	*Hylophilus amaurocephalus*	IndoMalay	0	-0.1319	-	3.39	0.8346	0.3614	8.96
Vireonidae	*Hylophilus aurantiifrons*	Neotropics	0	-0.0326	2.1612	4.07	0.8318	0.8016	10.94
Vireonidae	*Hylophilus brunneiceps*	Neotropics	0	-0.2351	1.9284	3.60	0.8399	0.6567	42.94
Vireonidae	*Hylophilus decurtatus*	Australasia	0	-0.8412	-	2.79	0.7823	0.1433	7.17
Vireonidae	*Hylophilus flavipes*	Australasia	0	-0.6422	-	3.00	0.7739	0.5120	25.61
Vireonidae	*Hylophilus hypoxanthus*	Neotropics	0	0.1246	2.3188	3.98	0.7503	0.3562	85.62
Vireonidae	*Hylophilus muscicapinus*	Neotropics	0	-0.0699	1.7574	4.03	0.7833	0.8129	26.55
Vireonidae	*Hylophilus ochraceiceps*	Neotropics	0	0.1117	-	4.12	0.7861	0.4747	51.61
Vireonidae	*Hylophilus olivaceus*	Neotropics	0	-0.0504	2.0613	4.27	0.7841	0.6479	80.62
Vireonidae	*Hylophilus pectoralis*	Afrotropics	0	0.0494	-	3.30	0.8237	0.2877	16.38
Vireonidae	*Hylophilus poicilotis*	Afrotropics	0	-0.1132	-	3.76	0.7948	0.6700	24.91

Family	Species	Realm	Threat	Latitude	Elevation	Anomaly	Size	Shape	Orientation
Vireonidae	*Hylophilus sclateri*	Afrotropics	0	-0.1500	-	3.47	0.8290	0.4095	25.98
Vireonidae	*Hylophilus semicinereus*	Afrotropics	0	-0.0003	-	3.21	0.7891	0.2153	13.52
Vireonidae	*Hylophilus thoracicus*	Neotropics	0	-0.4042	4.1484	3.43	0.8150	0.7428	62.24
Vireonidae	*Vireo altiloquus*	Palearctic	0	-0.6171	-	4.98	0.8258	0.2291	6.05
Vireonidae	*Vireo atricapilla*	Neotropics	0	0.0966	5.1029	3.02	0.7300	0.4442	51.09
Vireonidae	*Vireo bellii*	Afrotropics	0	-0.1092	15.4078	3.39	0.8001	0.1797	73.97
Vireonidae	*Vireo brevipennis*	Neotropics	0	0.0588	9.8868	4.01	0.7480	0.5766	25.39
Vireonidae	*Vireo carmioli*	Palearctic	0	-0.8587	-	4.19	0.7820	0.3676	41.49
Vireonidae	*Vireo cassinii*	Afrotropics	0	0.1231	11.8292	3.42	0.8429	0.4839	12.14
Vireonidae	*Vireo crassirostris*	Palearctic	0	-0.6009	5.9936	4.67	0.8561	0.4077	9.00
Vireonidae	*Vireo flavifrons*	Palearctic	0	-0.9178	-	5.05	0.8579	0.1678	1.71
Vireonidae	*Vireo flavoviridis*	Neotropics	0	0.0409	9.9494	3.47	0.7323	0.2233	33.81
Vireonidae	*Vireo gilvus*	Neotropics	0	-0.2825	-	2.50	0.7205	0.1442	18.97
Vireonidae	*Vireo griseus*	Australasia	0	-0.1447	12.2566	2.74	0.7640	0.4622	15.39
Vireonidae	*Vireo gundlachii*	Neotropics	0	0.2750	-	3.14	0.7050	0.2584	89.65
Vireonidae	*Vireo huttoni*	Palearctic	0	-0.7002	9.5450	3.66	0.7824	0.2685	0.58
Vireonidae	*Vireo hypochryseus*	Palearctic	0	-0.9301	8.3352	5.18	0.8320	0.5037	0.74
Vireonidae	*Vireo latimeri*	Neotropics	0	-0.3550	11.4568	3.64	0.7078	0.4471	36.89
Vireonidae	*Vireo leucophrys*	Neotropics	0	-0.3358	7.3212	3.42	0.8277	0.4325	43.25
Vireonidae	*Vireo magister*	Neotropics	0	-0.1625	6.0235	3.74	0.7512	0.2033	44.14
Vireonidae	*Vireo masteri*	Neotropics	0	-0.1623	7.9742	3.67	0.7744	0.4220	76.17
Vireonidae	*Vireo modestus*	Neotropics	1	0.0541	4.7249	2.47	0.6478	0.3985	28.33
Vireonidae	*Vireo nanus*	Afrotropics	0	0.0233	6.0865	3.24	0.7818	0.4200	69.21
Vireonidae	*Vireo nelsoni*	Palearctic	0	-0.5927	11.2431	3.99	0.8069	0.5213	16.29
Vireonidae	*Vireo olivaceus*	IndoMalay	0	-0.1443	6.6724	4.16	0.7403	0.1454	27.34
Vireonidae	*Vireo osburni*	Palearctic	0	-0.6869	-	4.46	0.8567	0.2889	0.28
Vireonidae	*Vireo plumbeus*	Australasia	0	0.1153	1.7363	3.26	0.7488	0.3682	19.31
Vireonidae	*Vireo solitarius*	Australasia	1	-0.7644	0.7521	2.60	0.6120	0.4082	18.43
Vireonidae	*Vireo vicinior*	Afrotropics	0	-1.3992	6.3340	2.75	0.6842	0.1360	6.91
Vireonidae	*Vireolanius eximius*	Australasia	0	-0.2875	5.6121	3.06	0.7888	0.5819	21.80
Vireonidae	*Vireolanius leucotis*	Australasia	1	-0.4827	2.8882	3.17	0.6859	0.4215	87.77
Vireonidae	*Vireolanius melitophrys*	Afrotropics	0	-0.0740	6.3453	3.18	0.7614	0.3874	70.15
Vireonidae	*Vireolanius pulchellus*	IndoMalay	0	-0.0691	5.0077	2.66	0.7060	0.2610	75.04
Zosteropidae	*Chlorocharis emiliae*	Australasia	0	-0.3400	-	3.33	0.7887	0.3197	9.38
Zosteropidae	*Heleia crassirostris*	Australasia	0	-0.4057	4.5875	3.27	0.8307	0.7914	6.43
Zosteropidae	*Heleia muelleri*	Neotropics	0	-0.5531	-	2.13	0.7700	0.0529	2.23
Zosteropidae	*Hypocryptadius cinnamomeus*	Neotropics	0	-0.2406	-	3.64	0.7946	0.3832	68.97
Zosteropidae	*Lophozosterops dohertyi*	Australasia	0	-0.4477	-	2.64	0.6891	0.2662	38.57
Zosteropidae	*Lophozosterops goodfellowi*	Afrotropics	0	-0.0040	6.6074	3.62	0.8272	0.2934	6.59
Zosteropidae	*Lophozosterops javanicus*	Australasia	0	-0.3246	-	3.32	0.8019	0.5496	26.25
Zosteropidae	*Lophozosterops pinaiae*	Afrotropics	0	-0.1795	-	3.45	0.8457	0.5432	32.67
Zosteropidae	*Lophozosterops squamiceps*	IndoMalay	0	-0.4174	-	2.71	0.7112	0.1623	10.99
Zosteropidae	*Lophozosterops superciliaris*	Australasia	0	0.1483	-	3.36	0.7192	0.6288	29.85
Zosteropidae	*Madanga ruficollis*	Palearctic	1	0.3912	1.7994	3.66	0.7136	0.5254	16.62
Zosteropidae	*Oculocincta squamifrons*	Neotropics	0	-0.2204	3.3553	3.93	0.7767	0.5332	37.51
Zosteropidae	*Speirops brunneus*	IndoMalay	0	-0.1172	4.4034	3.96	0.7718	0.2538	18.04
Zosteropidae	*Speirops melanocephalus*	Nearctic	0	-0.6305	5.3327	4.29	0.8317	0.3409	13.85
Zosteropidae	*Tephrozosterops stalkeri*	Neotropics	1	-0.0096	0.3124	4.44	0.6370	0.9258	90.00
Zosteropidae	*Zosterops abyssinicus*	Afrotropics	0	0.0087	-	3.71	0.7906	0.5716	64.66
Zosteropidae	*Zosterops anomalus*	Afrotropics	0	-0.0124	-	3.31	0.7510	0.1860	4.76
Zosteropidae	*Zosterops atricapilla*	Afrotropics	0	-0.1839	-	4.16	0.8018	0.5419	15.52
Zosteropidae	*Zosterops atriceps*	Afrotropics	0	0.2541	-	3.36	0.7591	0.2832	4.22
Zosteropidae	*Zosterops atrifrons*	Afrotropics	0	-0.0052	-	3.37	0.8061	0.1169	8.50
Zosteropidae	*Zosterops buruensis*	Neotropics	0	-0.3936	-	2.62	0.7300	0.3200	31.84
Zosteropidae	*Zosterops ceylonensis*	Nearctic	1	-1.0095	7.4023	4.20	0.7602	0.4310	60.18
Zosteropidae	*Zosterops chloris*	Nearctic	0	-0.8646	6.9806	4.31	0.8128	0.5879	29.75
Zosteropidae	*Zosterops citrinella*	Neotropics	0	0.0685	10.1314	3.66	0.7300	0.2705	18.93
Zosteropidae	*Zosterops consobrinorum*	Neotropics	0	0.2796	8.7317	3.03	0.6478	0.3341	24.93
Zosteropidae	*Zosterops erythropleurus*	Nearctic	0	-0.5069	12.3031	3.46	0.7830	0.5266	86.18
Zosteropidae	*Zosterops everetti*	Neotropics	0	-0.2166	-	2.44	0.5788	0.0000	41.52
Zosteropidae	*Zosterops explorator*	Nearctic	0	-0.8288	-	4.23	0.8165	0.7245	6.33
Zosteropidae	*Zosterops flavifrons*	Neotropics	0	-0.0864	-	3.50	0.7859	0.2794	29.67
Zosteropidae	*Zosterops flavus*	Nearctic	0	-0.8076	10.1543	4.24	0.8441	0.5602	20.63
Zosteropidae	*Zosterops fuscicapilla*	Nearctic	0	-0.8625	-	4.05	0.8136	0.4956	24.72
Zosteropidae	*Zosterops hypoxanthus*	Neotropics	0	-0.3307	-	2.53	0.6974	0.1632	17.43
Zosteropidae	*Zosterops japonicus*	Nearctic	0	-0.5484	14.0188	3.73	0.7948	0.1694	44.99
Zosteropidae	*Zosterops kuehni*	Neotropics	0	-0.1637	-	3.74	0.7524	0.2080	42.82
Zosteropidae	*Zosterops lateralis*	Neotropics	0	0.8202	2.8909	2.41	0.5788	0.0000	0.00
Zosteropidae	*Zosterops luteus*	Neotropics	0	-0.0297	8.1545	3.63	0.7840	0.3980	53.90
Zosteropidae	*Zosterops maderaspatanus*	Neotropics	0	0.0657	-	2.61	0.6302	0.1249	76.17
Zosteropidae	*Zosterops metcalfii*	Neotropics	1	0.9391	2.9609	2.99	0.6804	0.1241	60.96
Zosteropidae	*Zosterops meyeni*	Neotropics	0	0.3794	-	2.47	0.5985	0.0000	0.00
Zosteropidae	*Zosterops minor*	Neotropics	0	-0.3532	-	2.46	0.6523	0.5852	2.23
Zosteropidae	*Zosterops montanus*	Neotropics	0	0.0266	8.4248	3.69	0.7344	0.2564	22.55

Family	Species	Realm	Threat	Latitude	Elevation	Anomaly	Size	Shape	Orientation
Zosteropidae	*Zosterops nigrorum*	Nearctic	0	-0.4320	4.7703	4.01	0.8521	0.3158	58.86
Zosteropidae	*Zosterops novaeguineae*	Neotropics	0	-0.3684	8.9070	2.47	0.5985	0.0000	0.00
Zosteropidae	*Zosterops pallidus*	Neotropics	0	-0.0834	-	3.29	0.7505	0.2921	23.54
Zosteropidae	*Zosterops palpebrosus*	Nearctic	0	-0.4063	-	4.23	0.8136	0.1322	9.81
Zosteropidae	*Zosterops poliogastrus*	Nearctic	0	-0.3951	11.9415	4.25	0.7962	0.3204	54.15
Zosteropidae	*Zosterops rendovae*	Nearctic	0	-0.6641	-	4.35	0.8195	0.2168	15.62
Zosteropidae	*Zosterops senegalensis*	Nearctic	0	-0.4028	4.2754	4.34	0.7660	0.6047	14.25
Zosteropidae	*Zosterops stalkeri*	Neotropics	0	-0.1989	-	3.45	0.7348	0.7887	71.53
Zosteropidae	*Zosterops wallacei*	Neotropics	0	0.0158	-	4.05	0.8250	0.6711	17.11
Zosteropidae	*Zosterops xanthochroa*	Neotropics	0	-0.2437	0.9611	3.64	0.7441	0.2484	21.82
